# Supplementary material for: Ubiquilin-2 liquid droplets catalyze α-synuclein fibril formation
Source: EMBO J. 2025 Oct 14;44(22):6527–55. doi: 10.1038/s44318-025-00591-1 (PMC12623503; doi:10.1038/s44318-025-00591-1)
Supplement: Supplementary file 1 — Appendix [file 44318_2025_591_MOESM1_ESM.pdf]

Appendix

Ubiquilin-2 liquid droplets catalyze α-synuclein fibril formation

Tomoki Takei, Yukiko Sasazawa, Daisuke Noshiro, Mitsuhiro Kitagawa, Tetsushi Kataura, Hiroko Hirawake-Mogi, Emi Kawauchi, Yuya Nakano, Etsu Tashiro, Tsuyoshi Saitoh, Shigeru Nishiyama, Seiichiro Ogawa, Soichiro Kakuta, Saiko Kazuno, Yoshiki Miura, Daisuke Taniguchi, Viktor I Korolchuk, Nobuo N. Noda, Shinji Saiki, Masaya Imoto, Nobutaka Hattori

Table of contents

Appendix Figure S1.
α-Syn aggregates within UBQLN2 droplets *in vitro*. 2

Appendix Figure S2.
α-Syn directly interacts with UBQLN2. 3

Appendix Figure S3.
α-Syn aggregates within UBQLN2 droplets in cultured cells. 4

Appendix Figure S4.
SO286 binds to STI1 regions of UBQLN2. 5

Appendix Figure S5.
SO286 inhibits α-syn aggregation within UBQLN2 droplets *in vitro*. 6

Appendix Figure S6.
SO286 inhibits α-syn aggregation within UBQLN2 droplets in cultured cells. 7

Appendix Table S1
Clinical information of human samples used in this study. 8

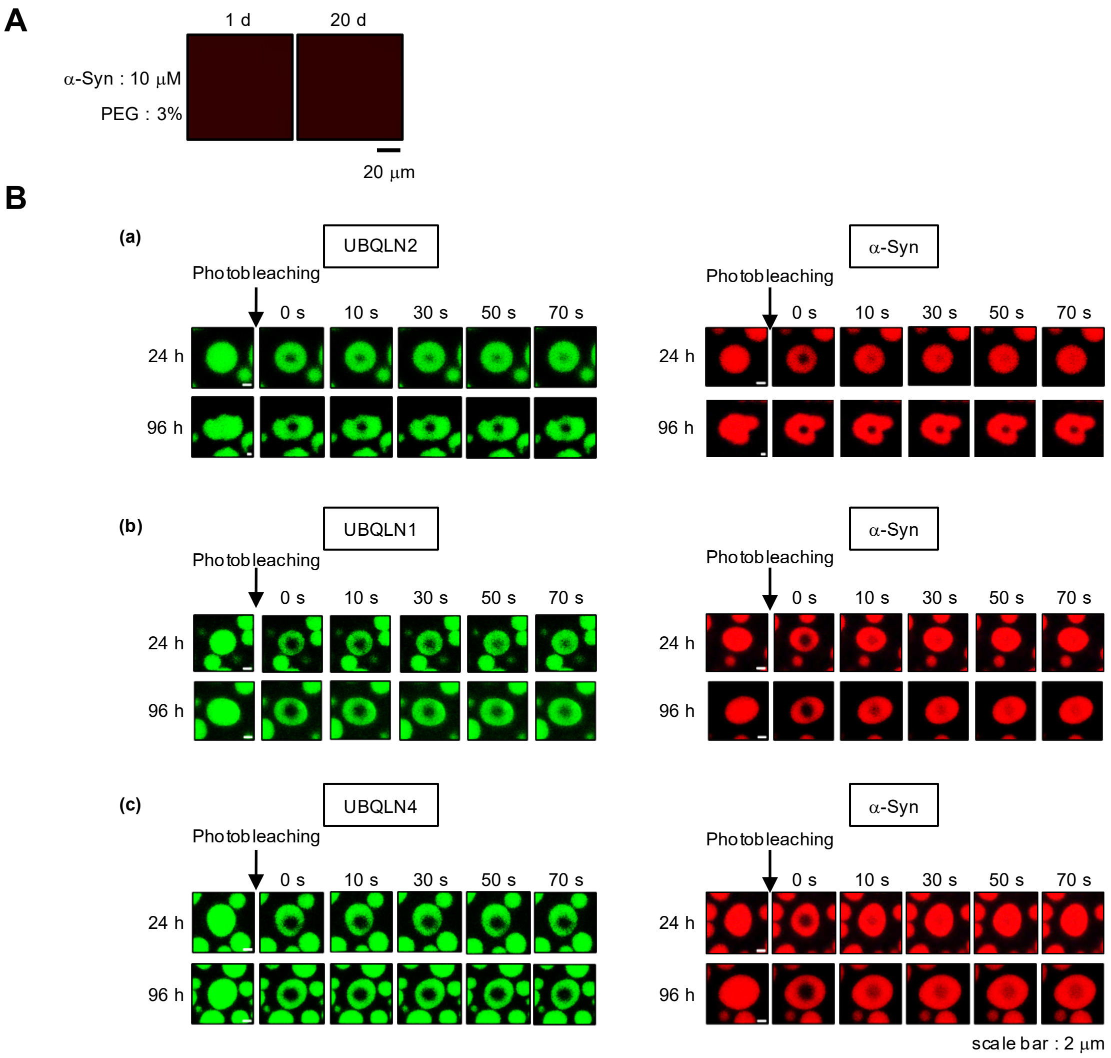

Appendix Figure S1. α-Syn aggregates within UBQLN2 droplets *in vitro*. (*A*) Fluorescence microscopy images of 10 μM of α-syn construct (1% DyLight633-labeling) in the presence of 3% PEG for the indicated time periods. (*B*) Fluorescence microscopy images of partial droplet photobleaching for UBQLN2 (*a*), UBQLN1 (*b*), and UBQLN4 (*c*) droplets incorporating α-syn.

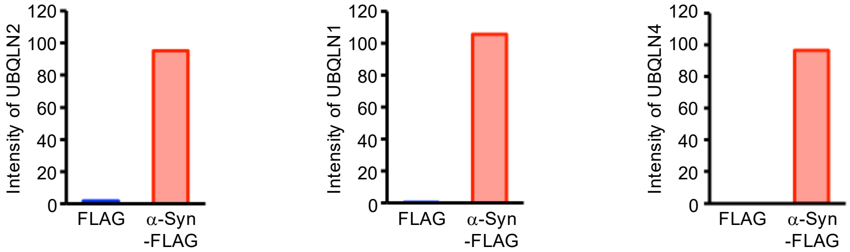

Appendix Figure S2. α-Syn directly interacts with UBQLN2. Recombinant α-syn-FLAG and each HA-UBQLN were incubated before α-syn-FLAG was pulled down with anti-FLAG beads and α-syn-FLAG was eluted with the FLAG peptide. The eluates were then immunoblotted with anti-UBQLN2, anti -UBQLN1, or anti-UBQLN4. Quantification of the band intensity of each UBQLN in Fig. EV2A which is representative data set from two independent experiments.

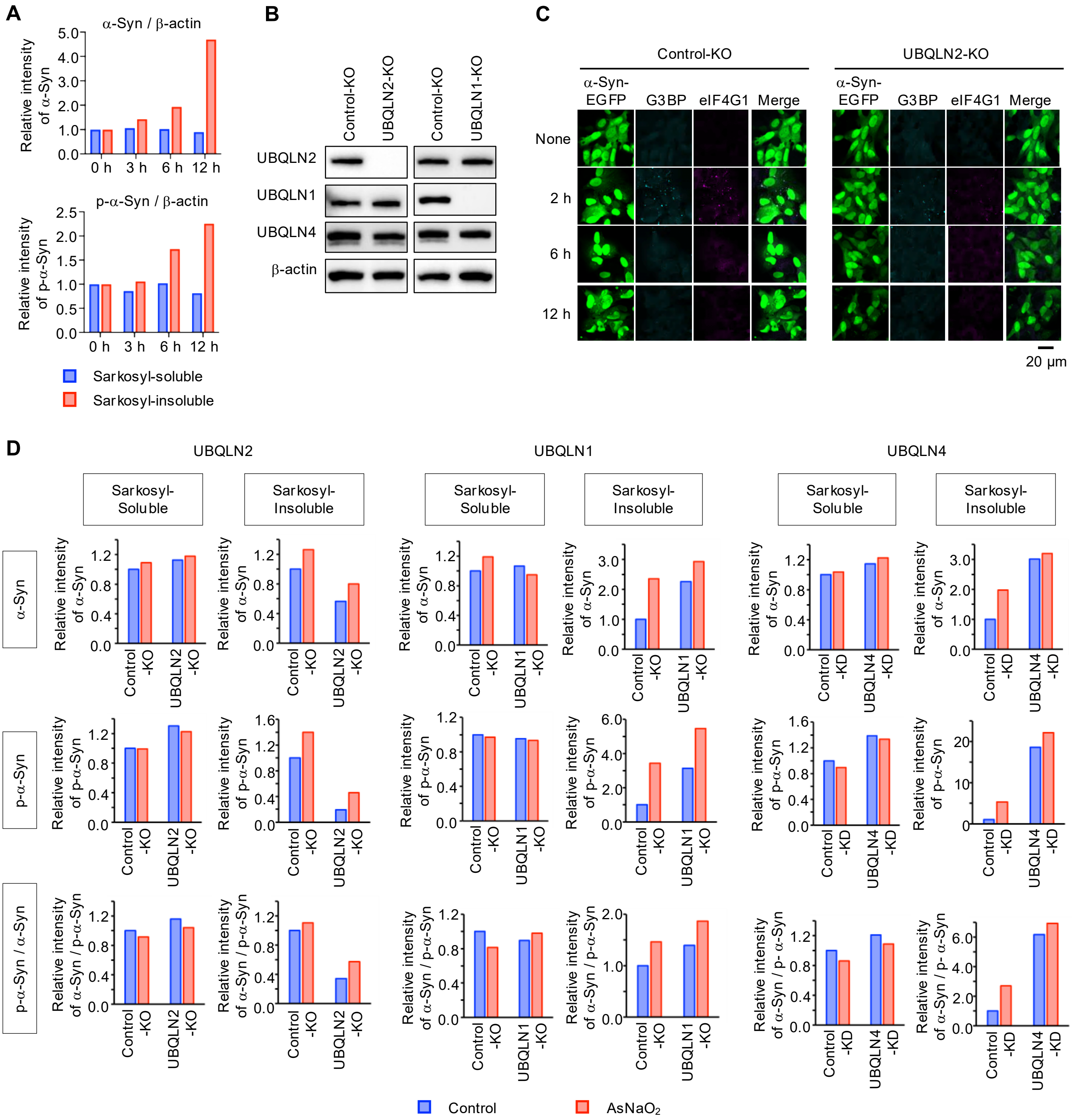

Appendix Figure S3. α-Syn aggregates within UBQLN2 droplets in cultured cells. *(A)* Quantification of the band intensities of α-syn and p-α-syn in Fig.3D which is representative data set from two independent experiments. (*B*) Immunoblot of Control-KO, UBQLN1-KO, and UBQLN2-KO α-syn (A53T)-EGFP/SH-SY5Y cell lysates. (*C*) Fluorescence microscopy of Control-KO or UBQLN2-KO α-syn (WT)-EGFP/SH-SY5Y cells were treated with 50 μM $AsNaO_2$ for 3, 6, and 12 h and immunostained with anti-G3BP and anti-eIF4G1antibodies. At least three experiments were replicated. (*D*) Quantification of the band intensities of α-syn, p-α-syn, and the ratio of p-α-syn to α-syn in Fig.3F which is representative data set from two independent experiments.

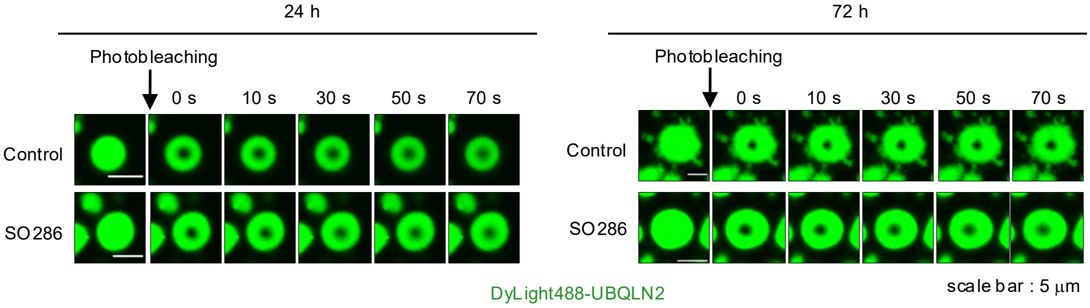

Appendix Figure S4. SO286 binds to STI1 regions of UBQLN2. Fluorescence microscopy images of partial photobleaching of UBQLN2 droplets incubated with or without SO286 at 37 ° C for 24 or 72 h.

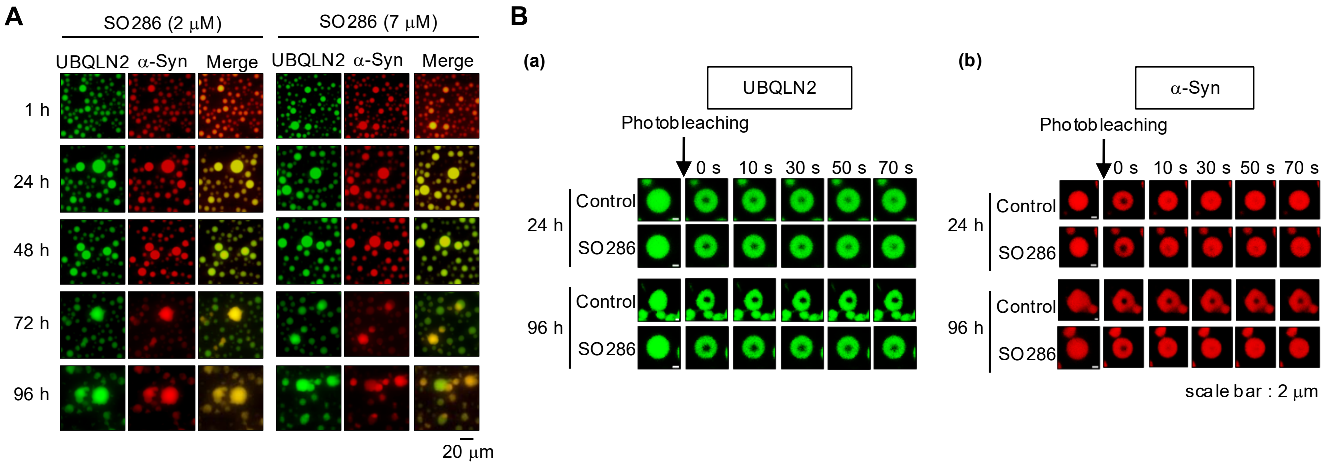

Appendix Figure S5. SO286 inhibits α-syn aggregation within UBQLN2 droplets *in vitro*. (*A*) Fluorescence microscopy images showing mixed solutions of 10 μM UBQLN2 (1% DyLight488-labeling) and 10 μM α-syn (1% DyLight633-labeling) in the presence of 2 μM and 7 μM SO286. (*B*) FRAP analysis of UBQLN2 droplets with α-syn and SO286. Fluorescence microscopy images of partial photobleaching for UBQLN2 and α-syn incubated with or without SO286 at 37 ºC for 24 or 72 h.

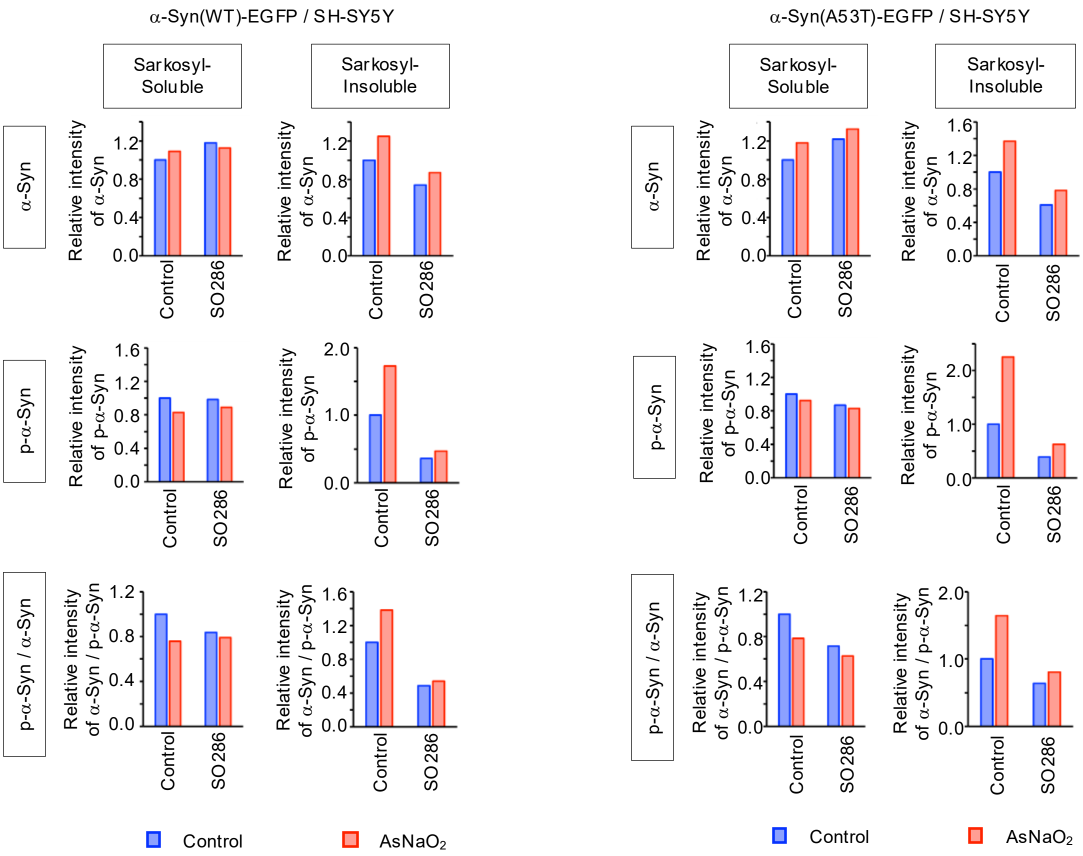

Appendix Figure S6. SO286 inhibits α-syn aggregation within UBQLN2 droplets in cultured cells. Quantification of the band intensities of α-syn, p-α-syn, and the ratio of p-α-syn to α-syn in Fig.6C and Fig. EV6B which are representative data sets from two independent experiments.

Appendix Table S1. Clinical information of human samples used in this study.

	Age at death	Sex	Disease duration (years)	Lewy body type pathology
PD1	82	Female	10	Limbic
PD2	89	Male	11	Diffuse neocortical type
PD3	83	Male	8	Limbic
PD4	61	Male	14	Limbic
