# Supplementary figures and images for: Ubiquilin-2 liquid droplets catalyze α-synuclein fibril formation (part 1 of 3)
Source: EMBO J. 2025 Oct 14;44(22):6527–55. doi: 10.1038/s44318-025-00591-1 (PMC12623503; doi:10.1038/s44318-025-00591-1)

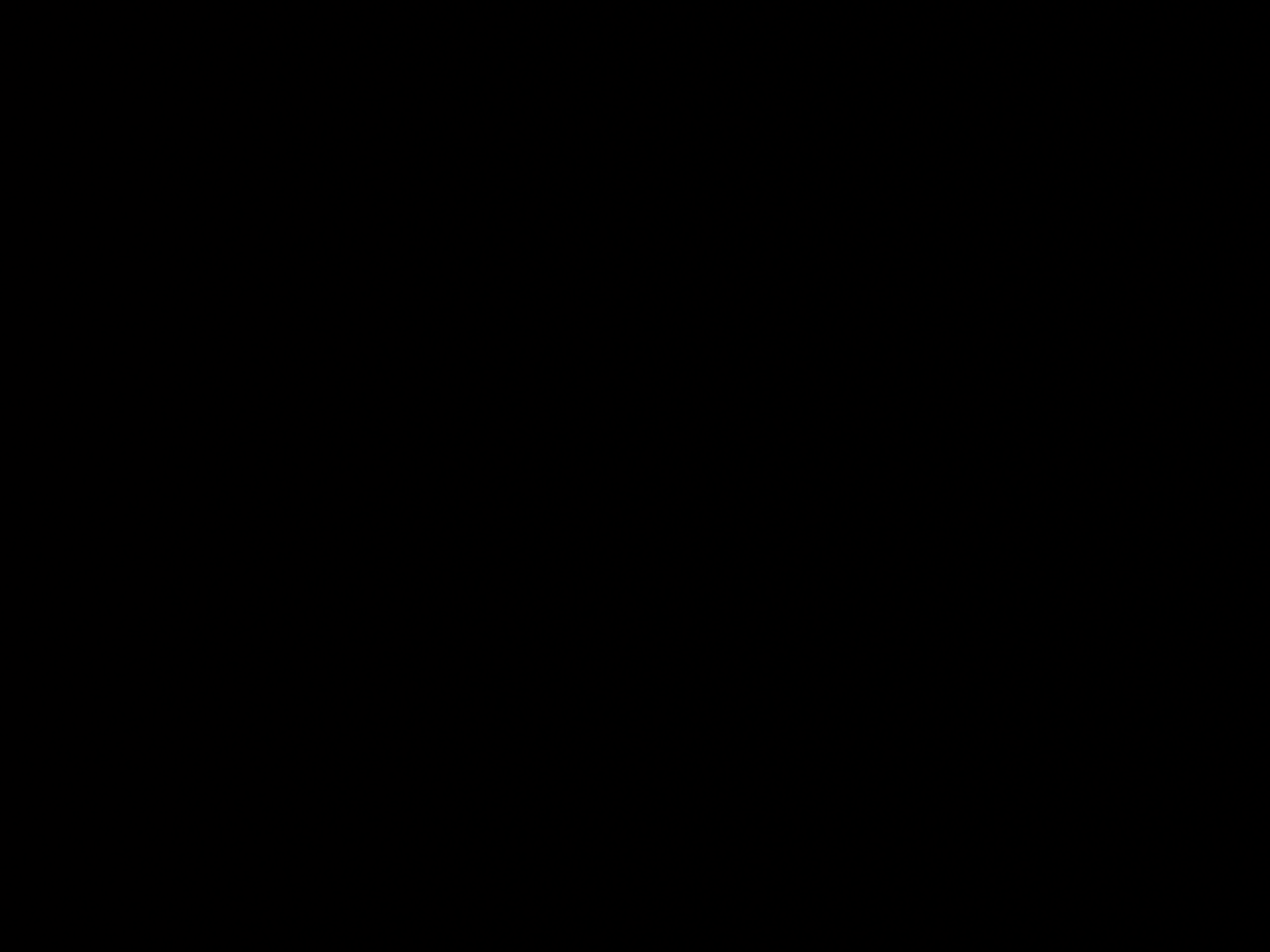

Supplement: Supplementary file 3 — Source data Fig. 1 [file 44318_2025_591_MOESM3_ESM.zip › Figure 1/1A/02_1 h_╬▒-Syn(╬▒-Syn).tif]

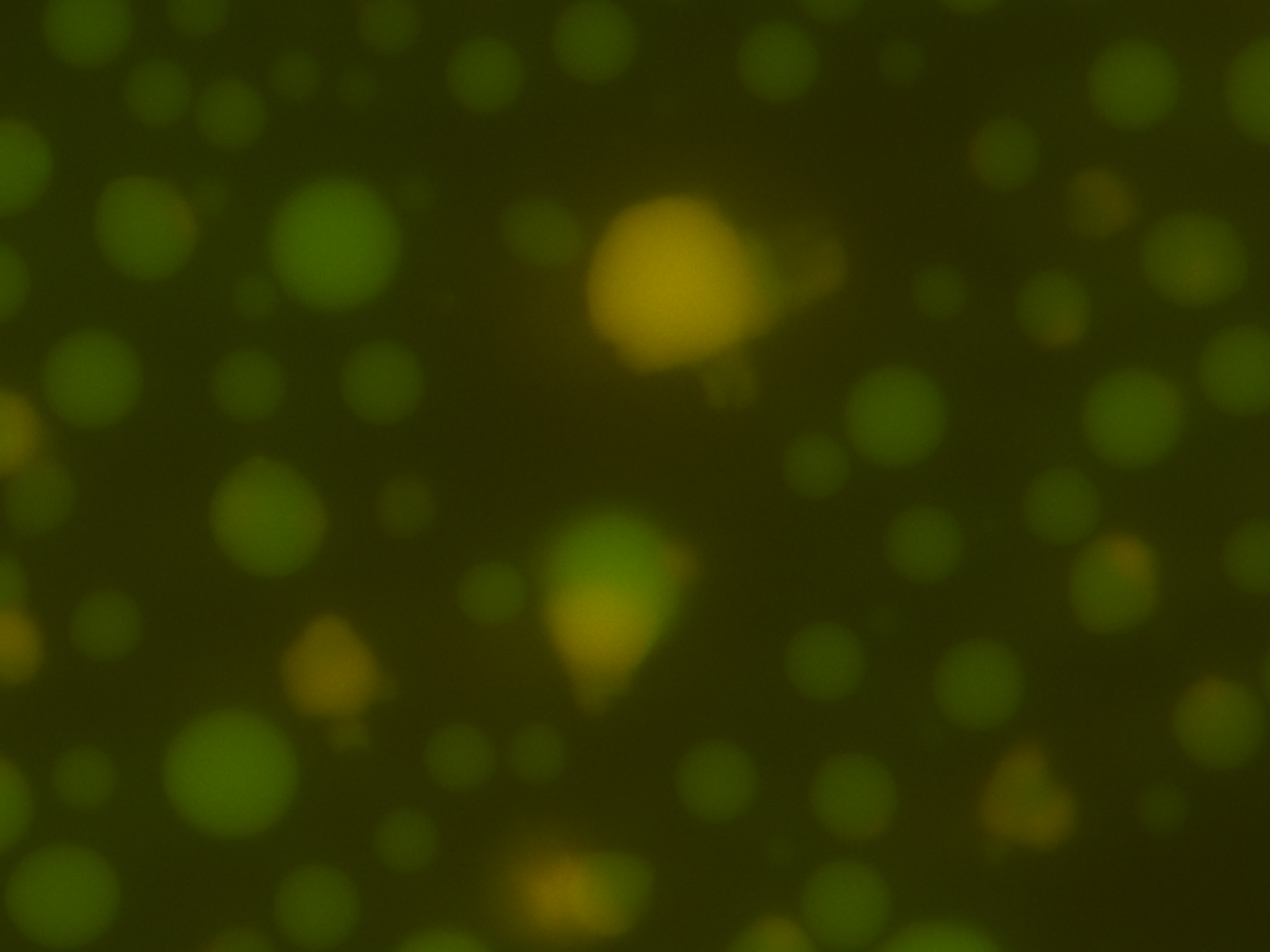

Supplement: Supplementary file 3 — Source data Fig. 1 [file 44318_2025_591_MOESM3_ESM.zip › Figure 1/1A/20_72 h_Merge(UBQLN2+╬▒-Syn).tif]

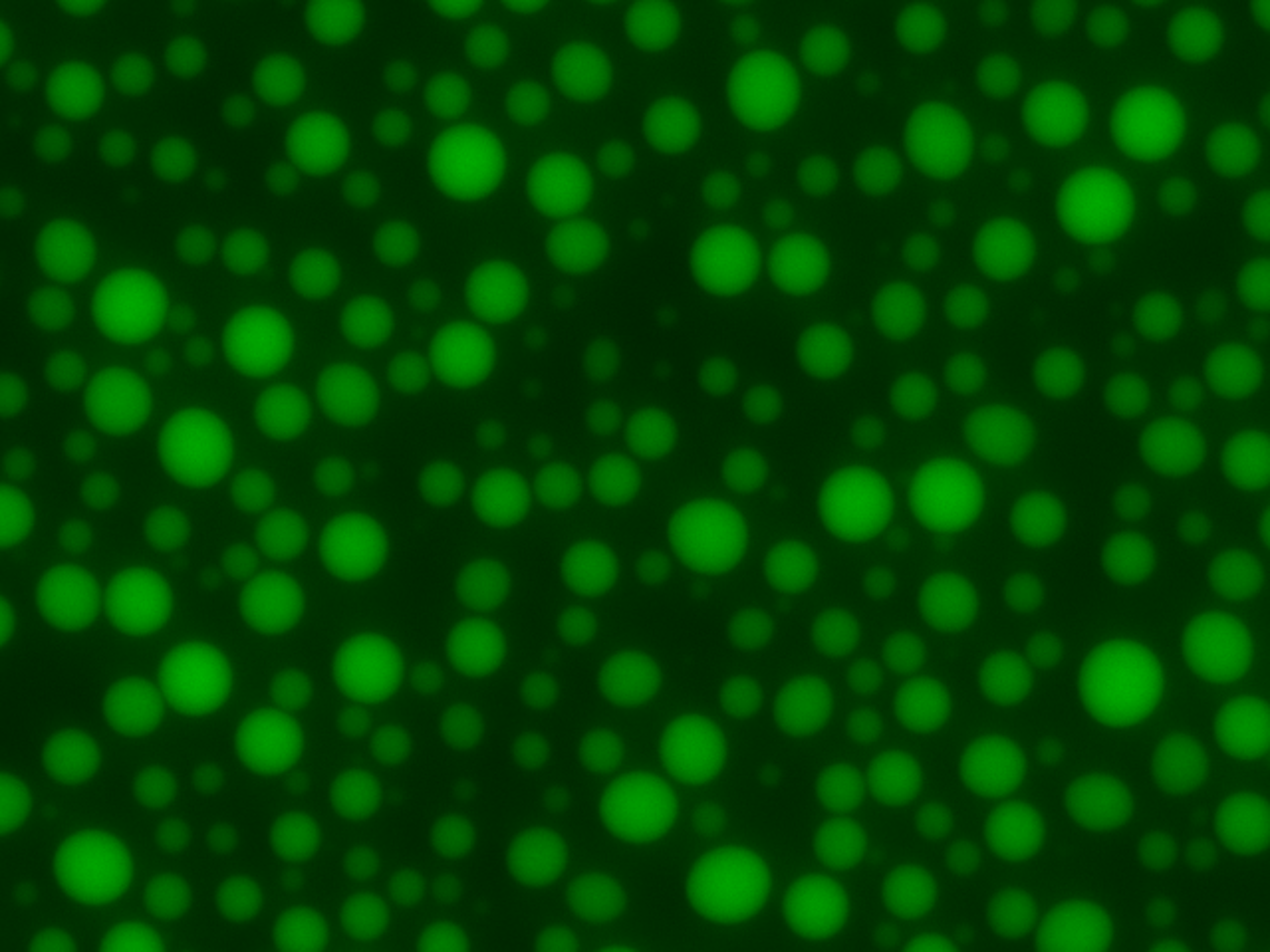

Supplement: Supplementary file 3 — Source data Fig. 1 [file 44318_2025_591_MOESM3_ESM.zip › Figure 1/1A/36_24 h_UBQLN4(UBQLN4).tif]

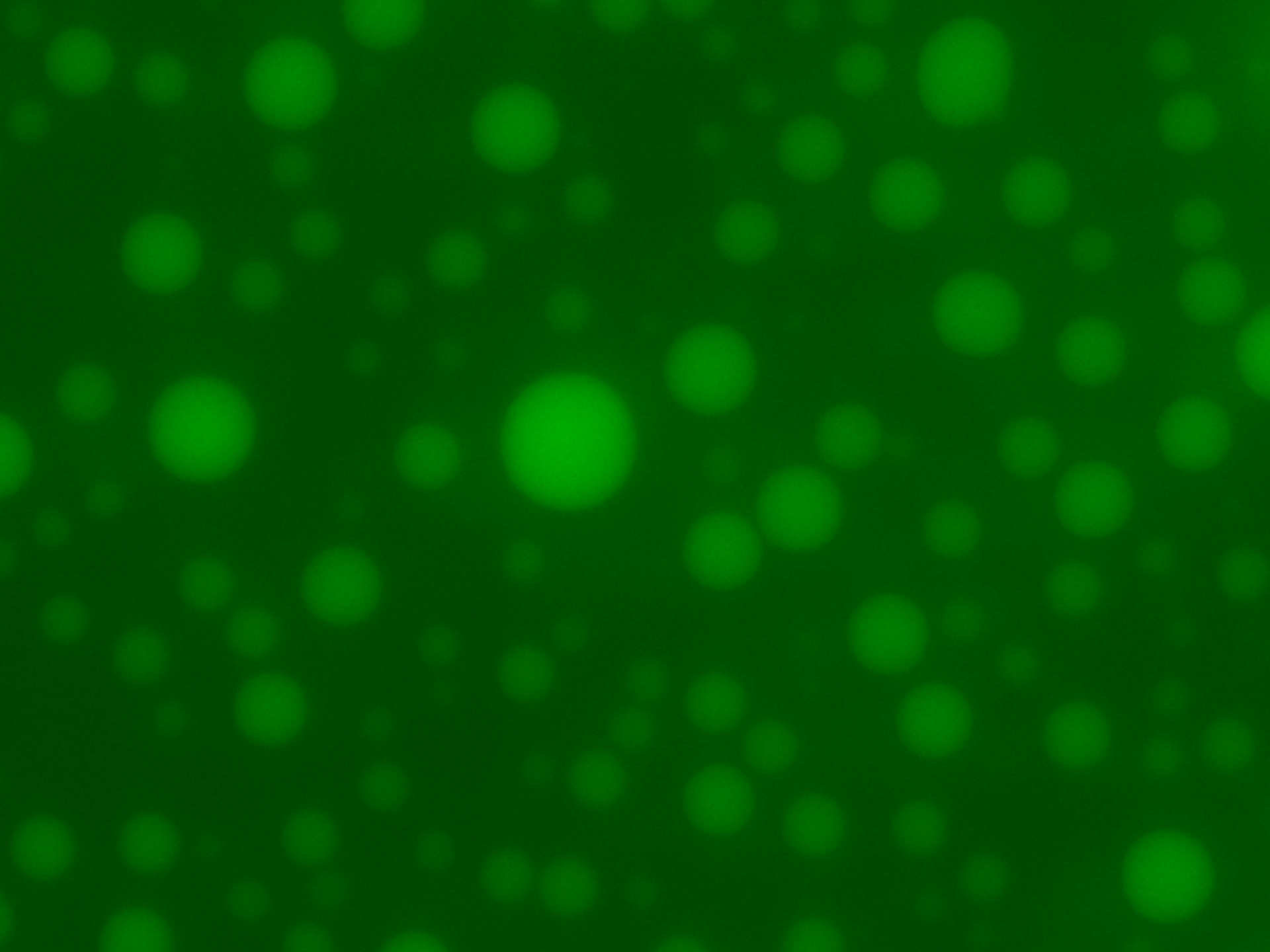

Supplement: Supplementary file 3 — Source data Fig. 1 [file 44318_2025_591_MOESM3_ESM.zip › Figure 1/1A/11_48 h_UBQLN2(UBQLN2).tif]

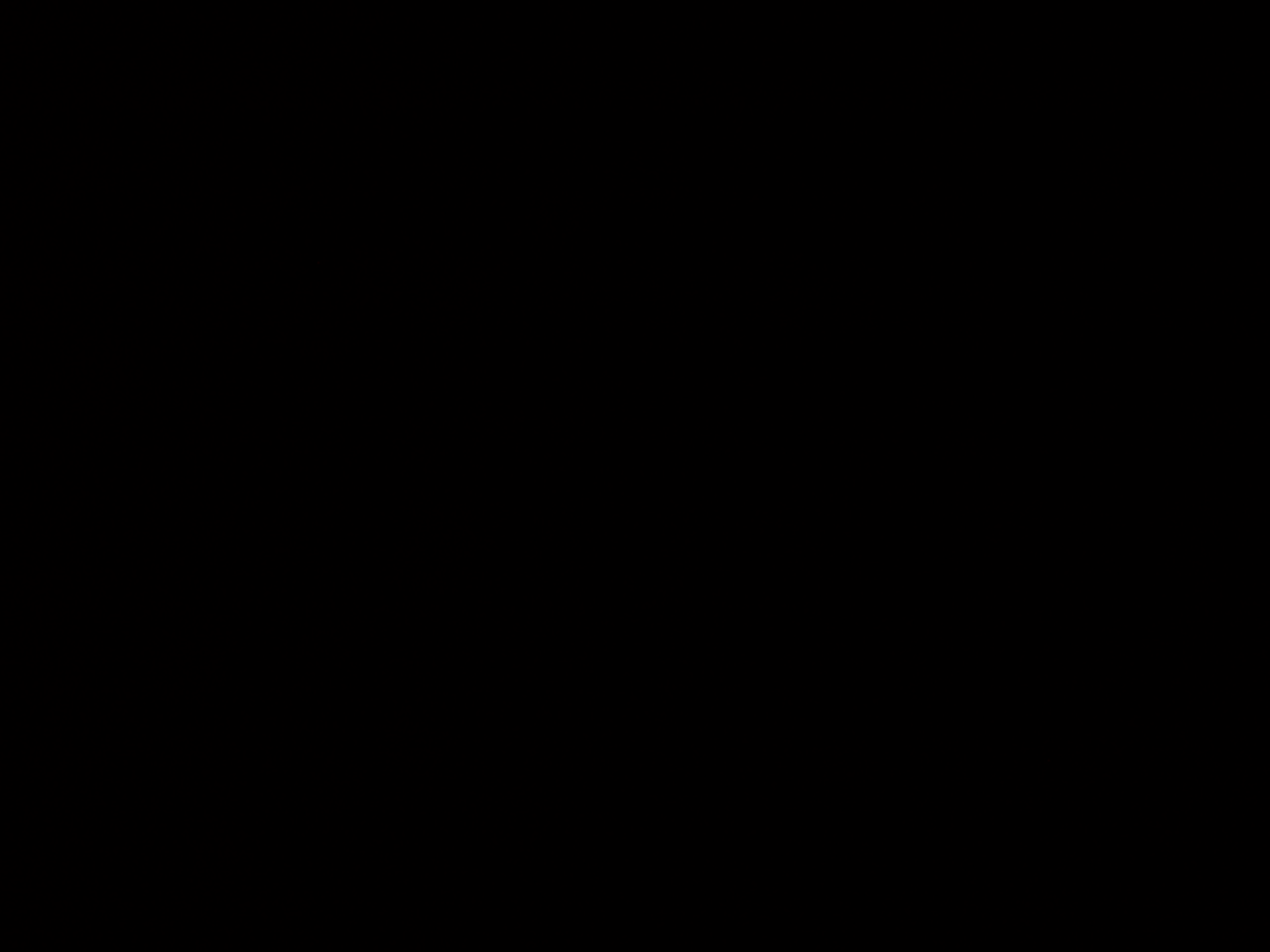

Supplement: Supplementary file 3 — Source data Fig. 1 [file 44318_2025_591_MOESM3_ESM.zip › Figure 1/1A/27_24 h_╬▒-Syn(╬▒-Syn).tif]

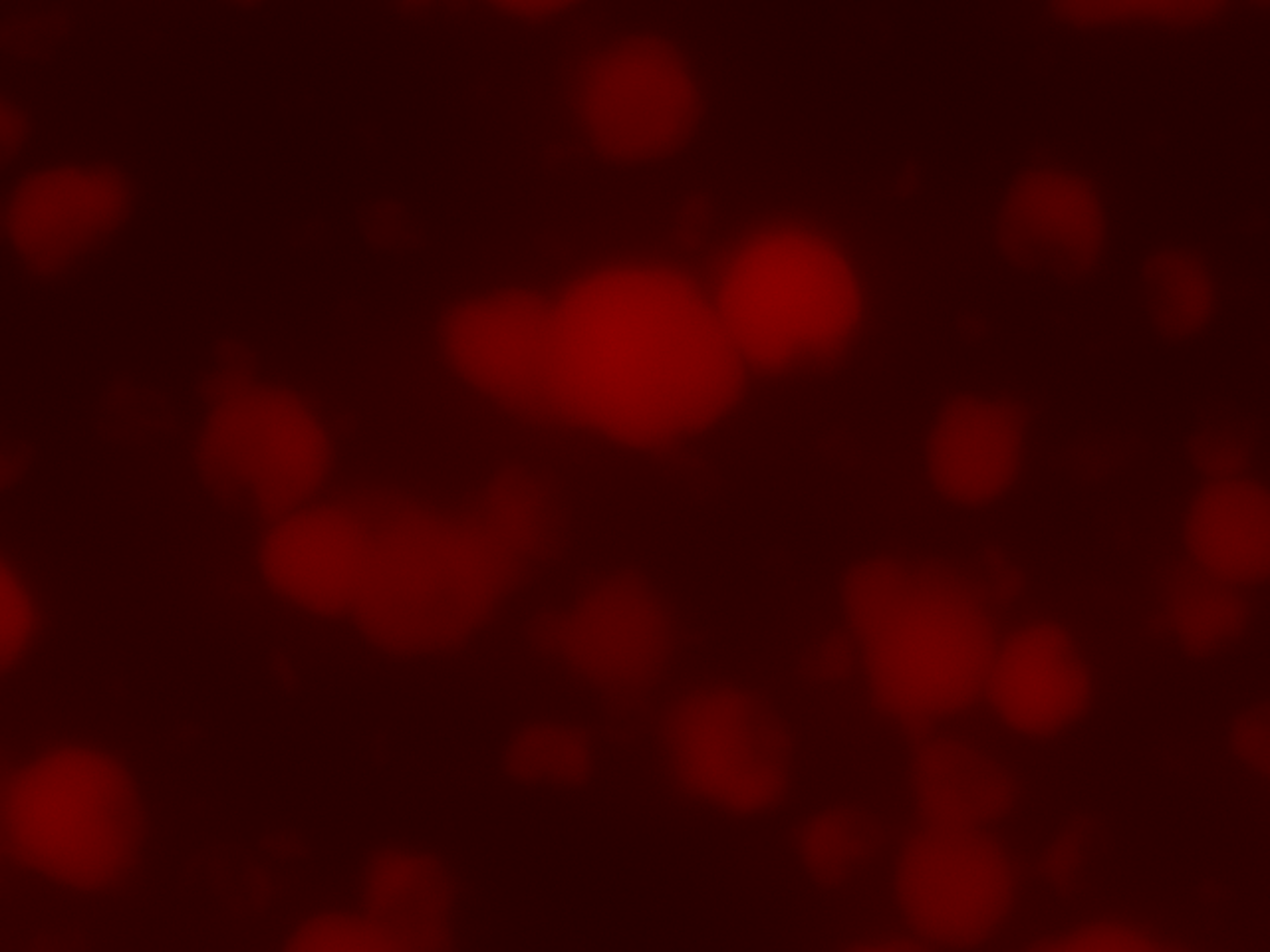

Supplement: Supplementary file 3 — Source data Fig. 1 [file 44318_2025_591_MOESM3_ESM.zip › Figure 1/1A/24_96 h_╬▒-Syn(UBQLN2+╬▒-Syn).tif]

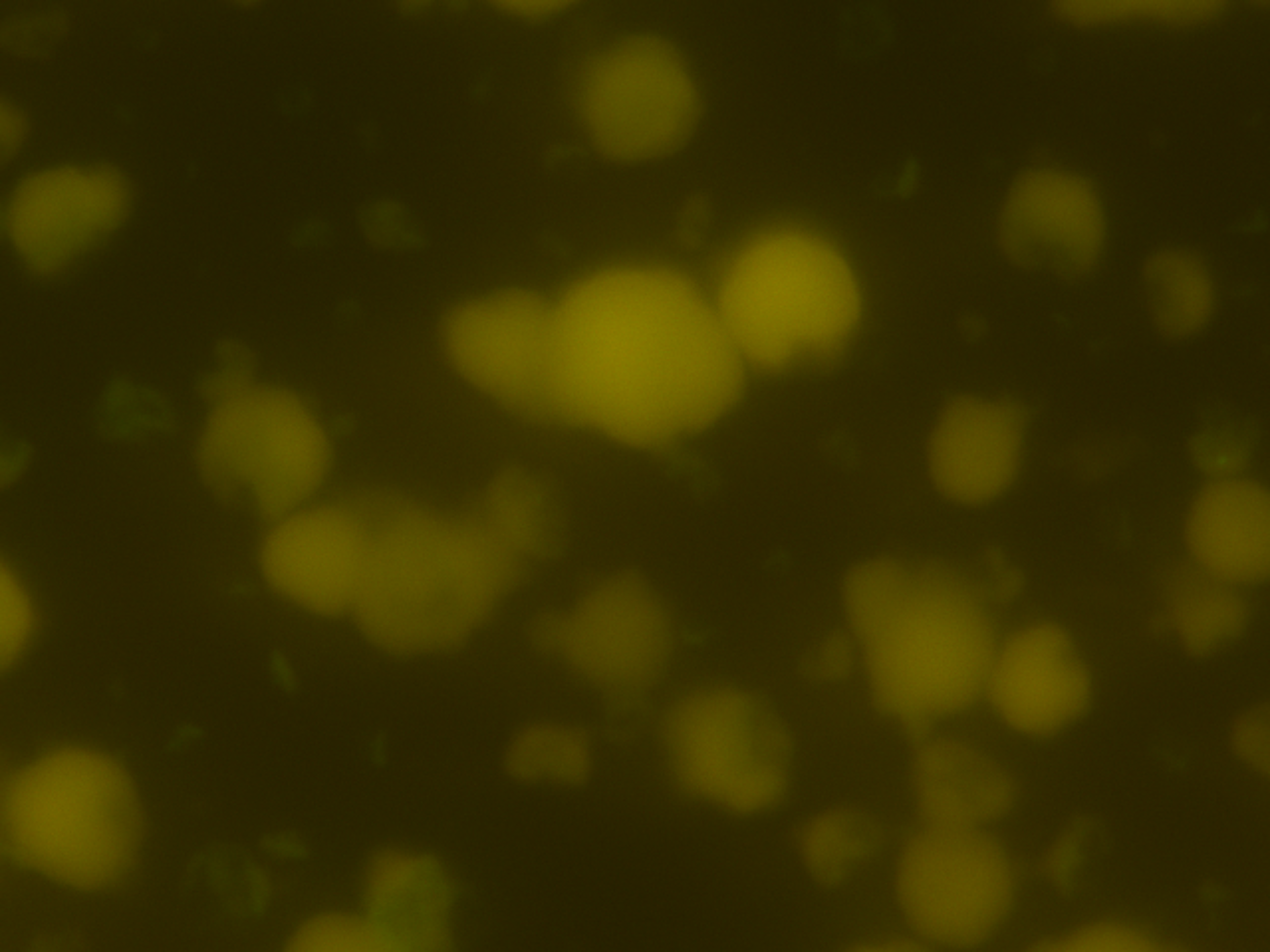

Supplement: Supplementary file 3 — Source data Fig. 1 [file 44318_2025_591_MOESM3_ESM.zip › Figure 1/1A/25_96 h_Merge(UBQLN2+╬▒-Syn).tif]

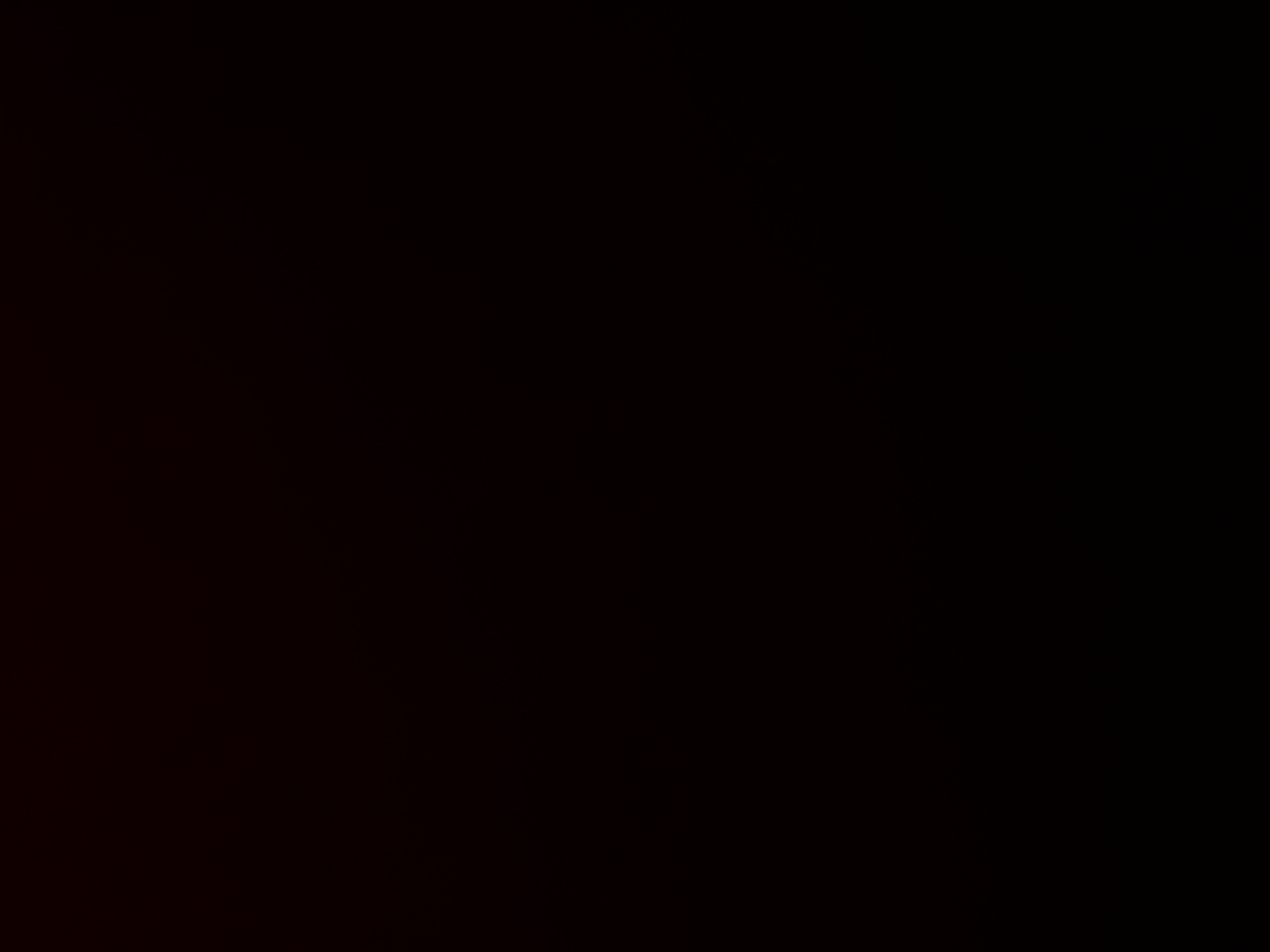

Supplement: Supplementary file 3 — Source data Fig. 1 [file 44318_2025_591_MOESM3_ESM.zip › Figure 1/1A/42_96 h_╬▒-Syn(╬▒-Syn).tif]

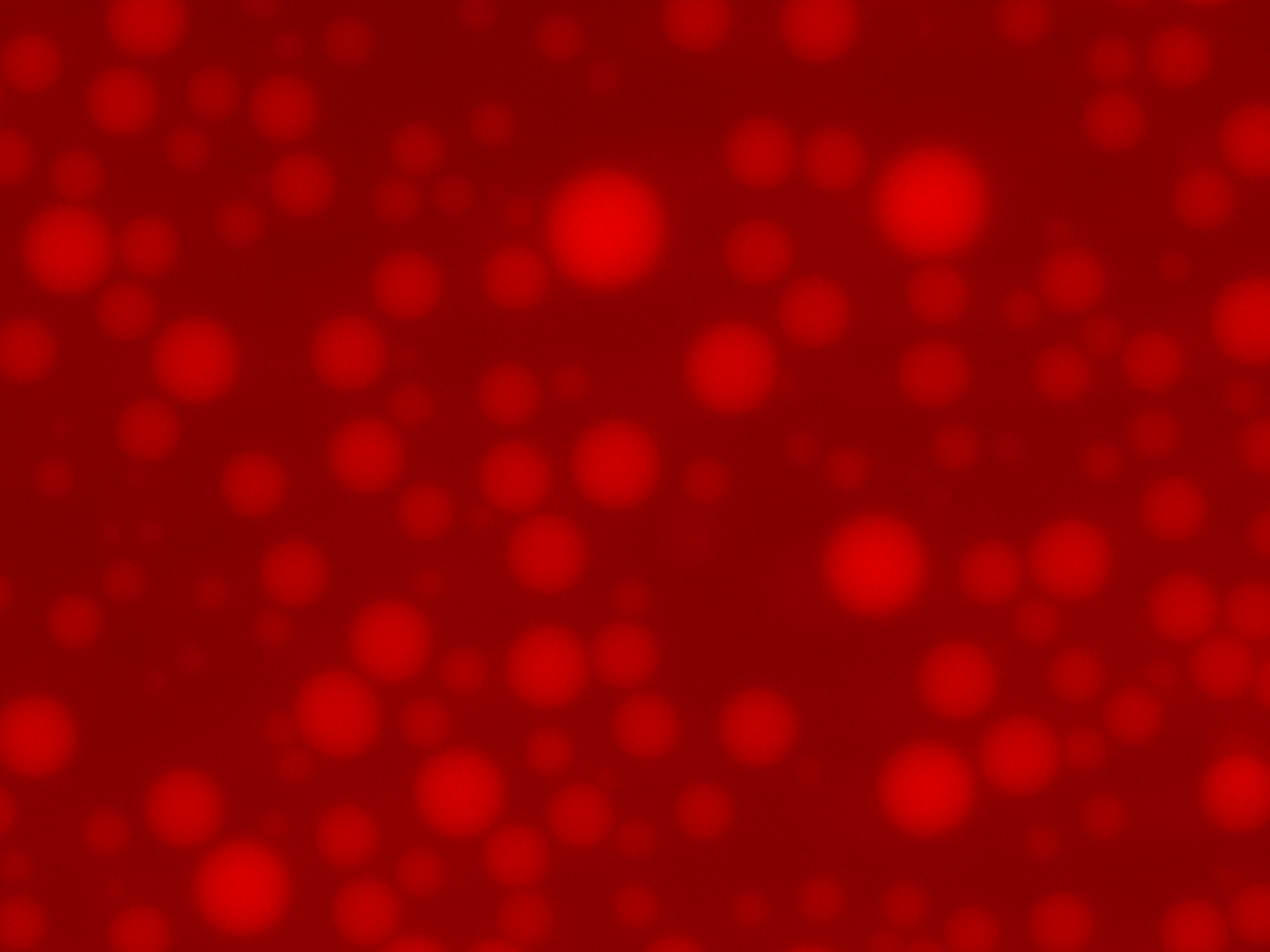

Supplement: Supplementary file 3 — Source data Fig. 1 [file 44318_2025_591_MOESM3_ESM.zip › Figure 1/1A/04_1 h_╬▒-Syn(UBQLN2+╬▒-Syn).tif]

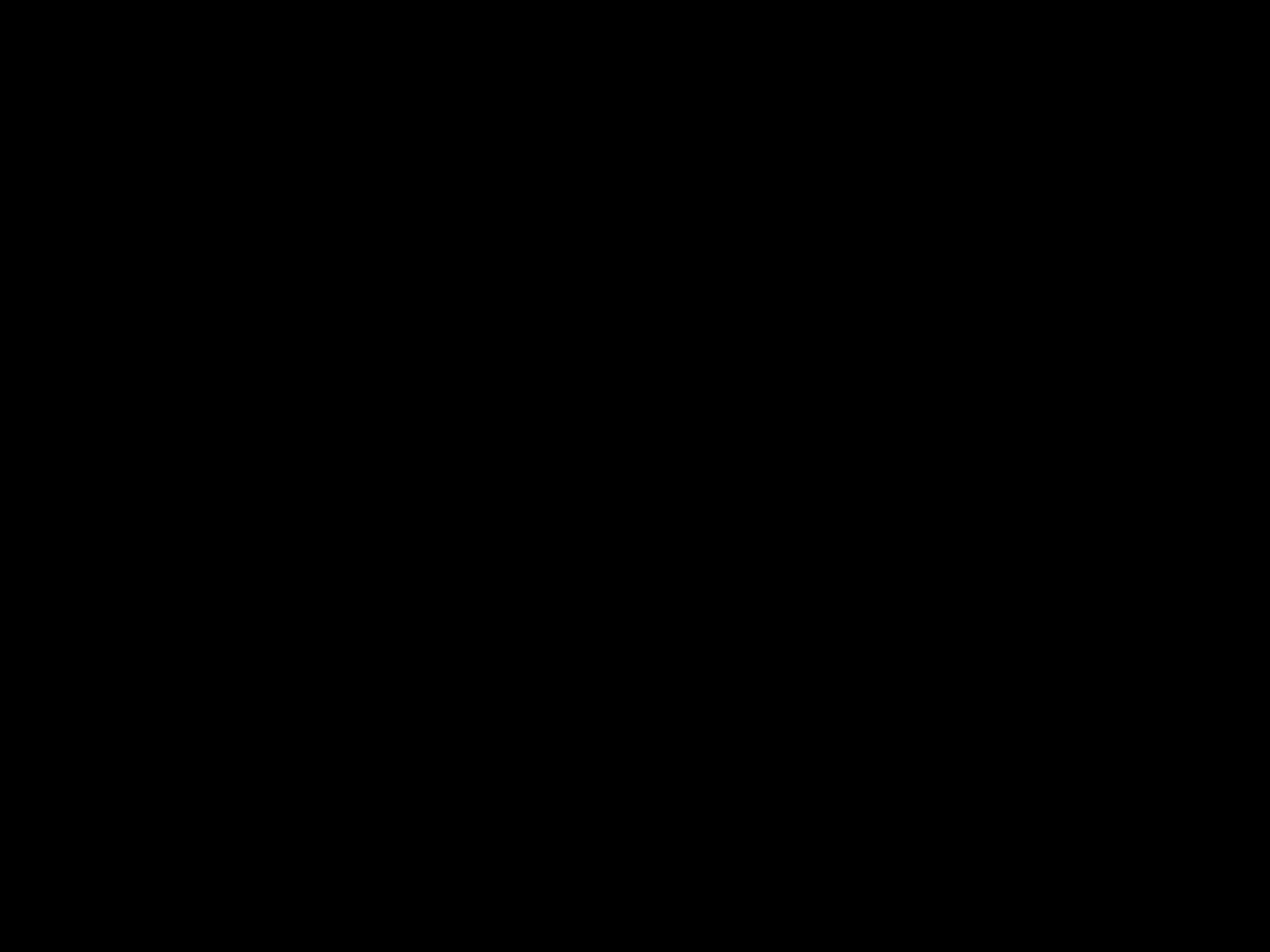

Supplement: Supplementary file 3 — Source data Fig. 1 [file 44318_2025_591_MOESM3_ESM.zip › Figure 1/1A/12_48 h_╬▒-Syn(╬▒-Syn).tif]

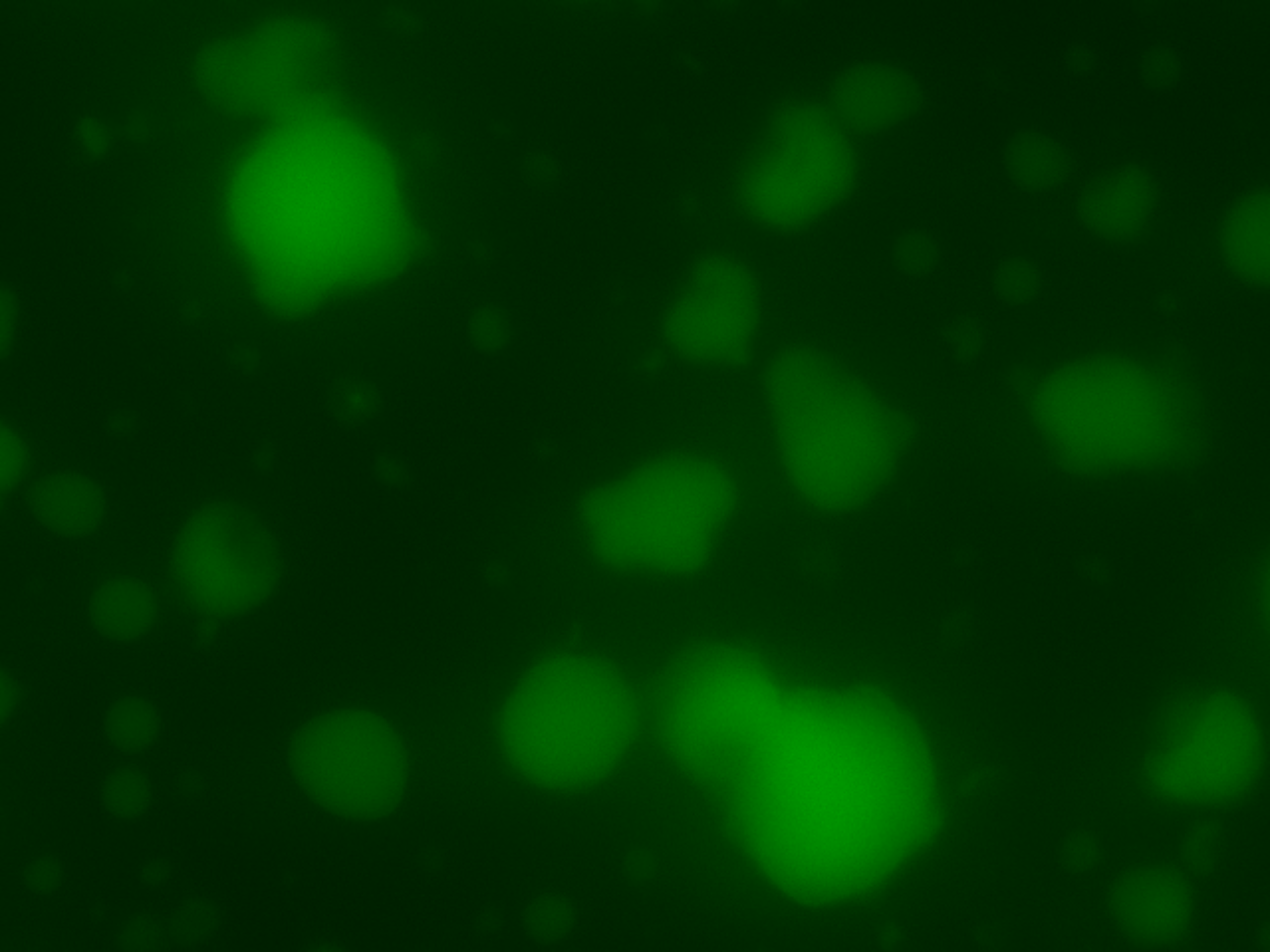

Supplement: Supplementary file 3 — Source data Fig. 1 [file 44318_2025_591_MOESM3_ESM.zip › Figure 1/1A/21_96 h_UBQLN2(UBQLN2).tif]

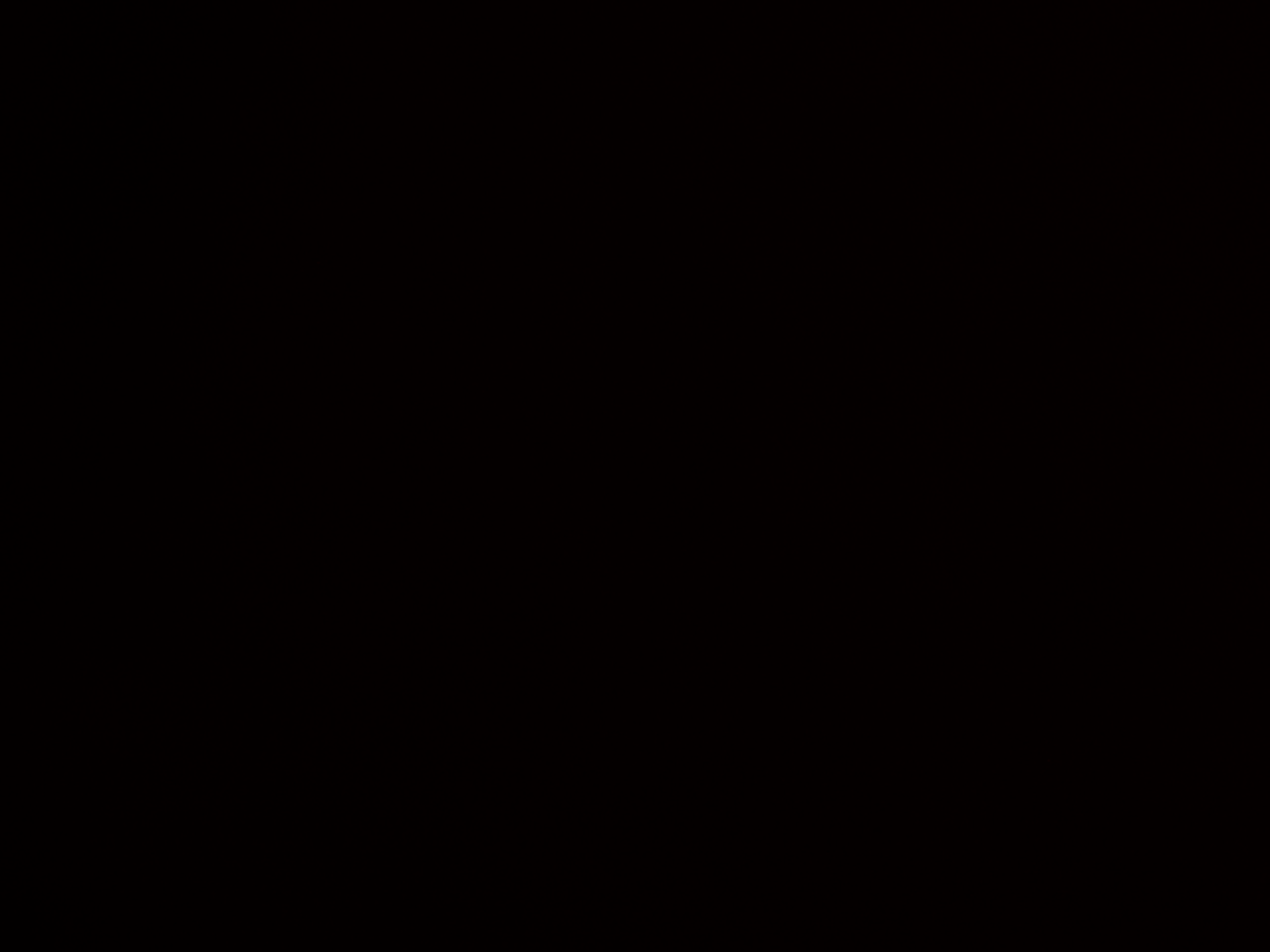

Supplement: Supplementary file 3 — Source data Fig. 1 [file 44318_2025_591_MOESM3_ESM.zip › Figure 1/1A/32_96 h_╬▒-Syn(╬▒-Syn).tif]

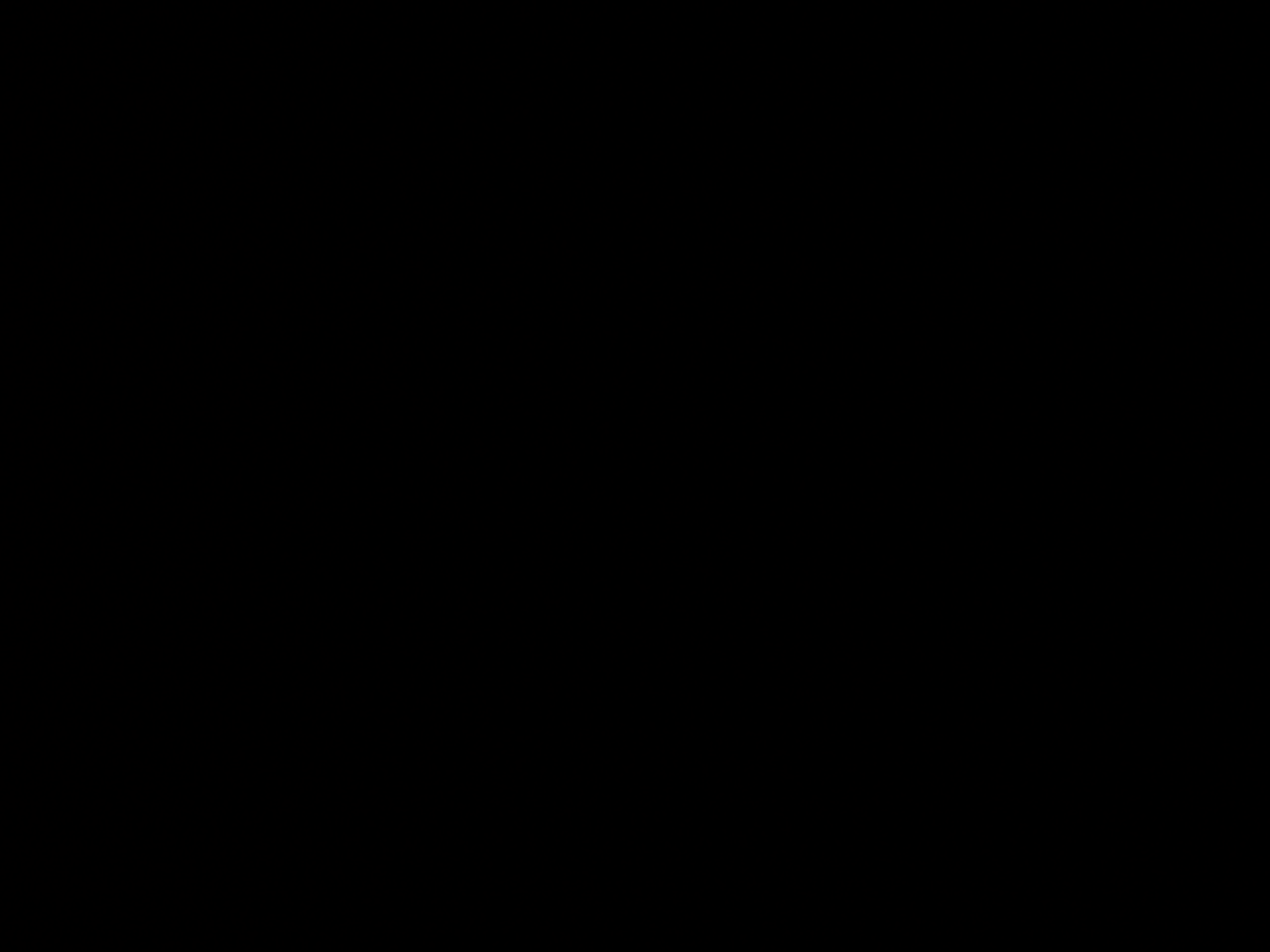

Supplement: Supplementary file 3 — Source data Fig. 1 [file 44318_2025_591_MOESM3_ESM.zip › Figure 1/1A/22_96 h_╬▒-Syn(╬▒-Syn).tif]

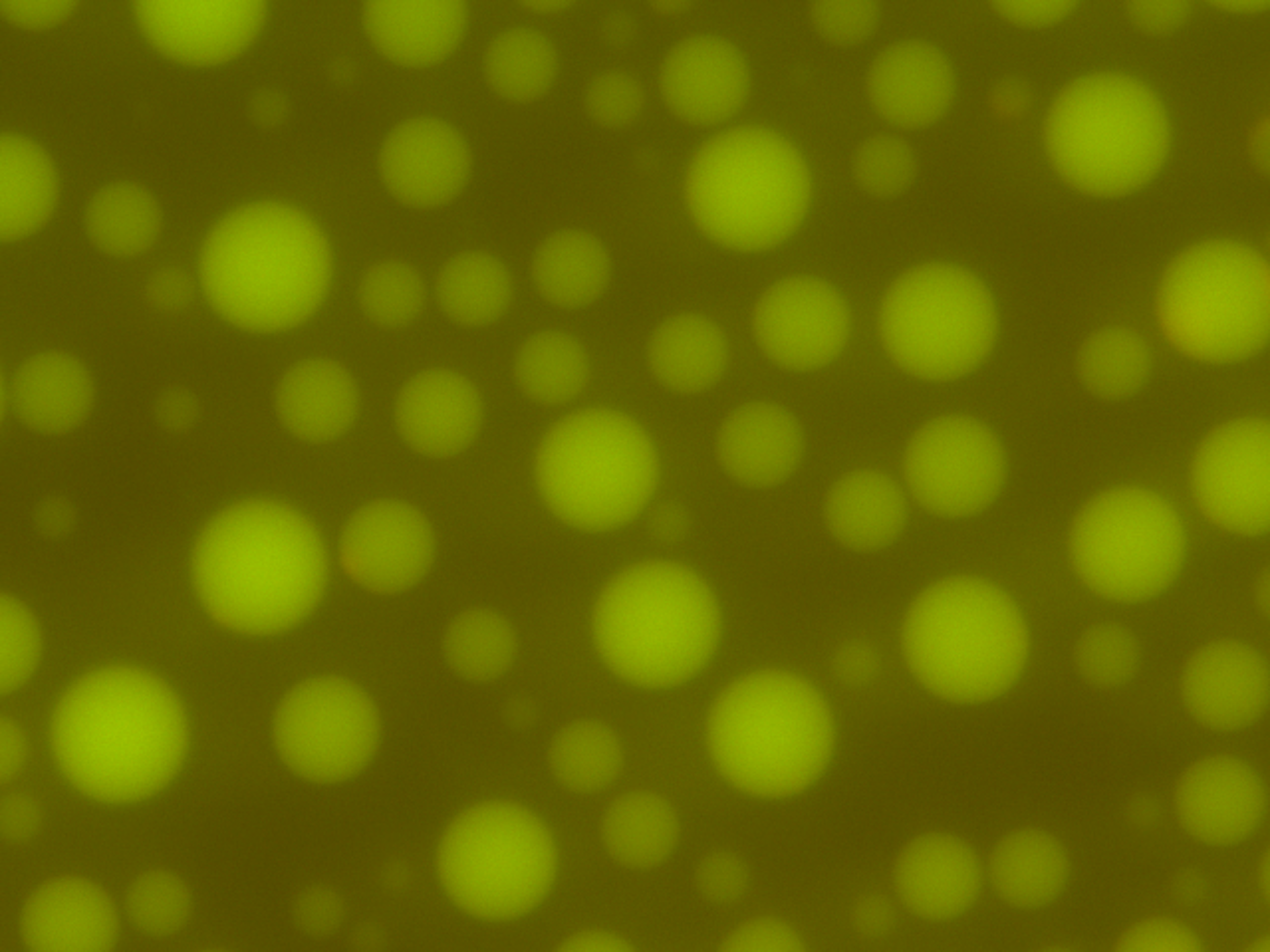

Supplement: Supplementary file 3 — Source data Fig. 1 [file 44318_2025_591_MOESM3_ESM.zip › Figure 1/1A/15_48 h_Merge(UBQLN2+╬▒-Syn).tif]

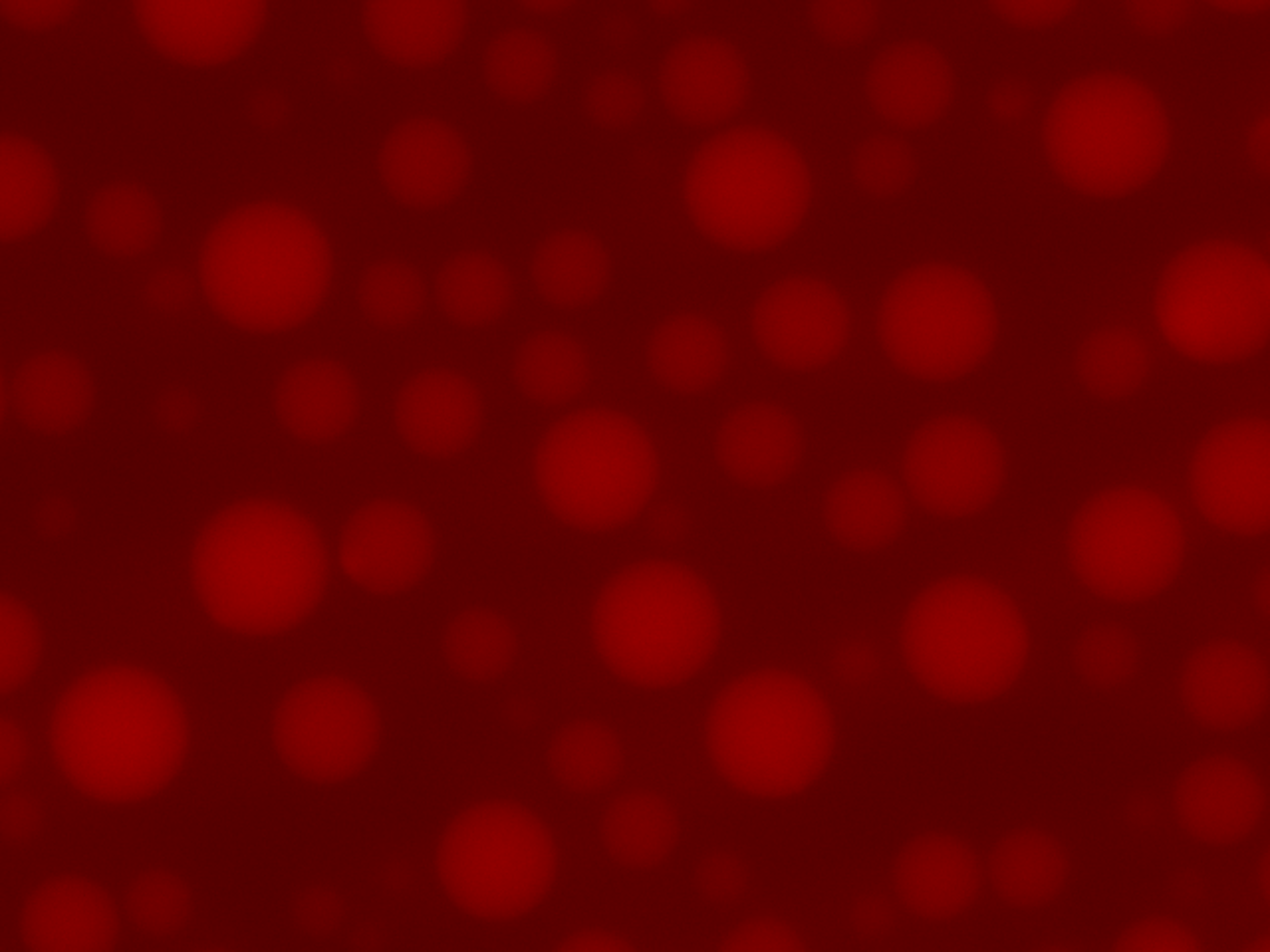

Supplement: Supplementary file 3 — Source data Fig. 1 [file 44318_2025_591_MOESM3_ESM.zip › Figure 1/1A/14_48 h_╬▒-Syn(UBQLN2+╬▒-Syn).tif]

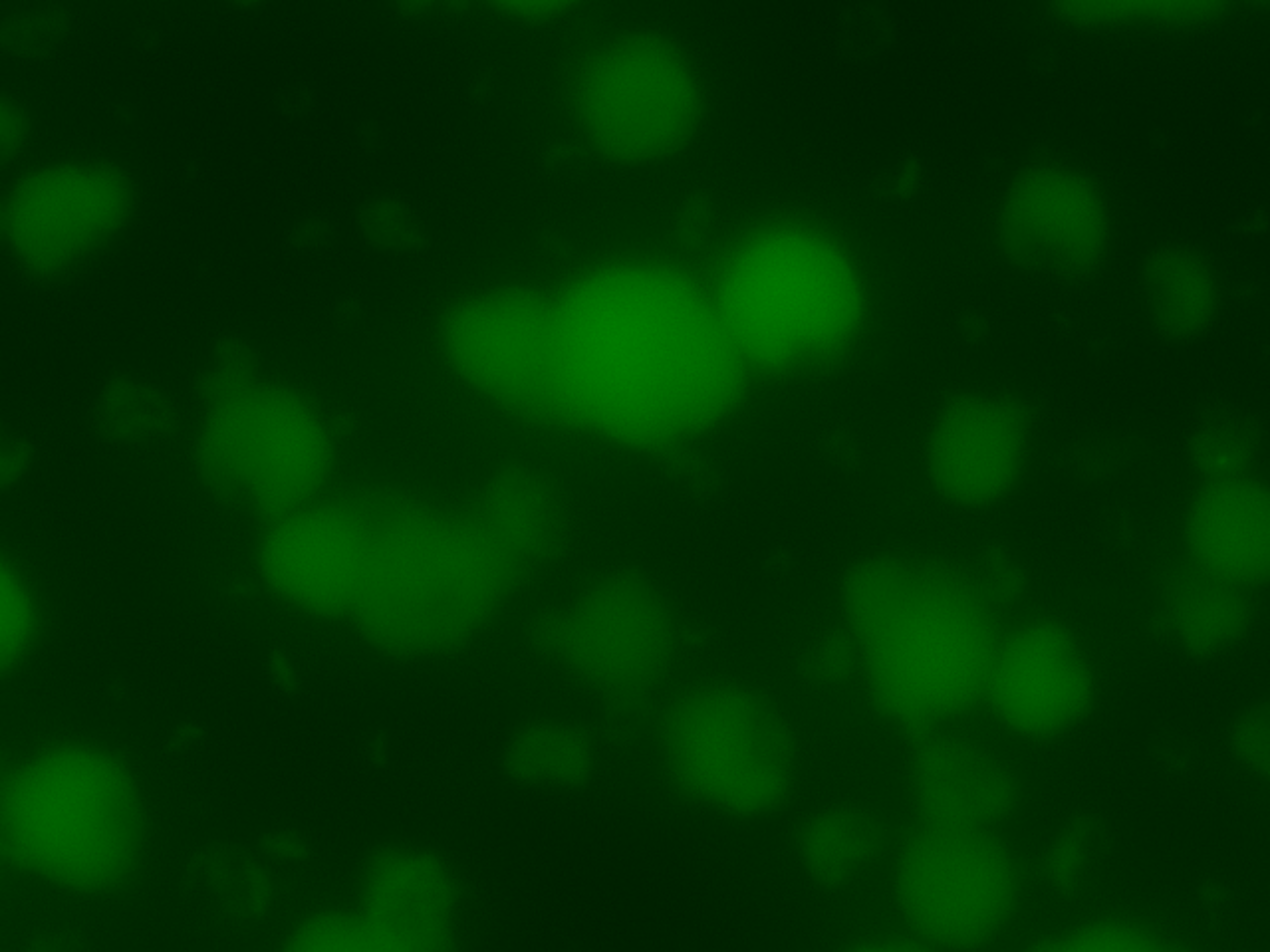

Supplement: Supplementary file 3 — Source data Fig. 1 [file 44318_2025_591_MOESM3_ESM.zip › Figure 1/1A/23_96 h_UBQLN2(UBQLN2+╬▒-Syn).tif]

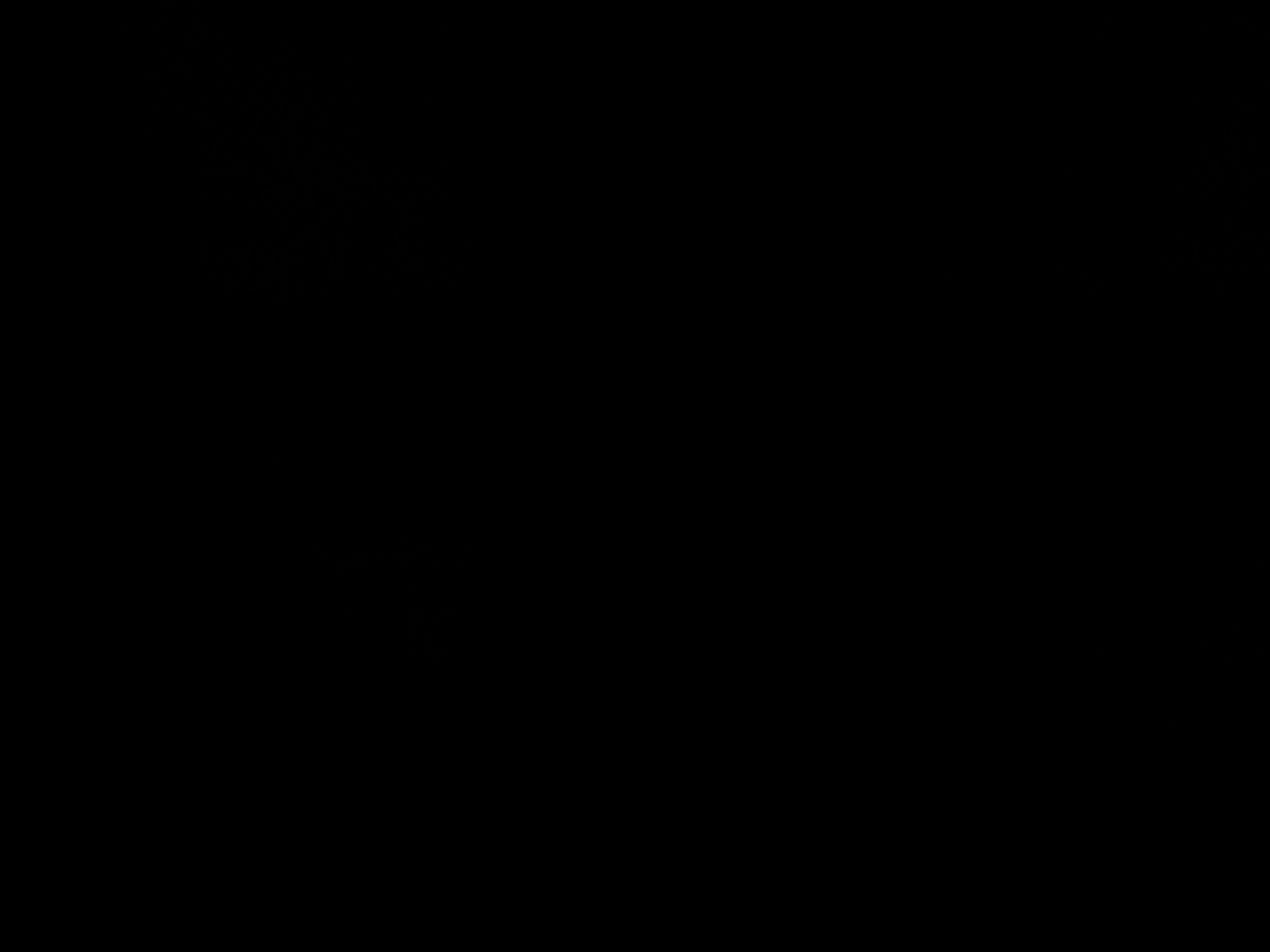

Supplement: Supplementary file 3 — Source data Fig. 1 [file 44318_2025_591_MOESM3_ESM.zip › Figure 1/1A/07_24 h_╬▒-Syn(╬▒-Syn).tif]

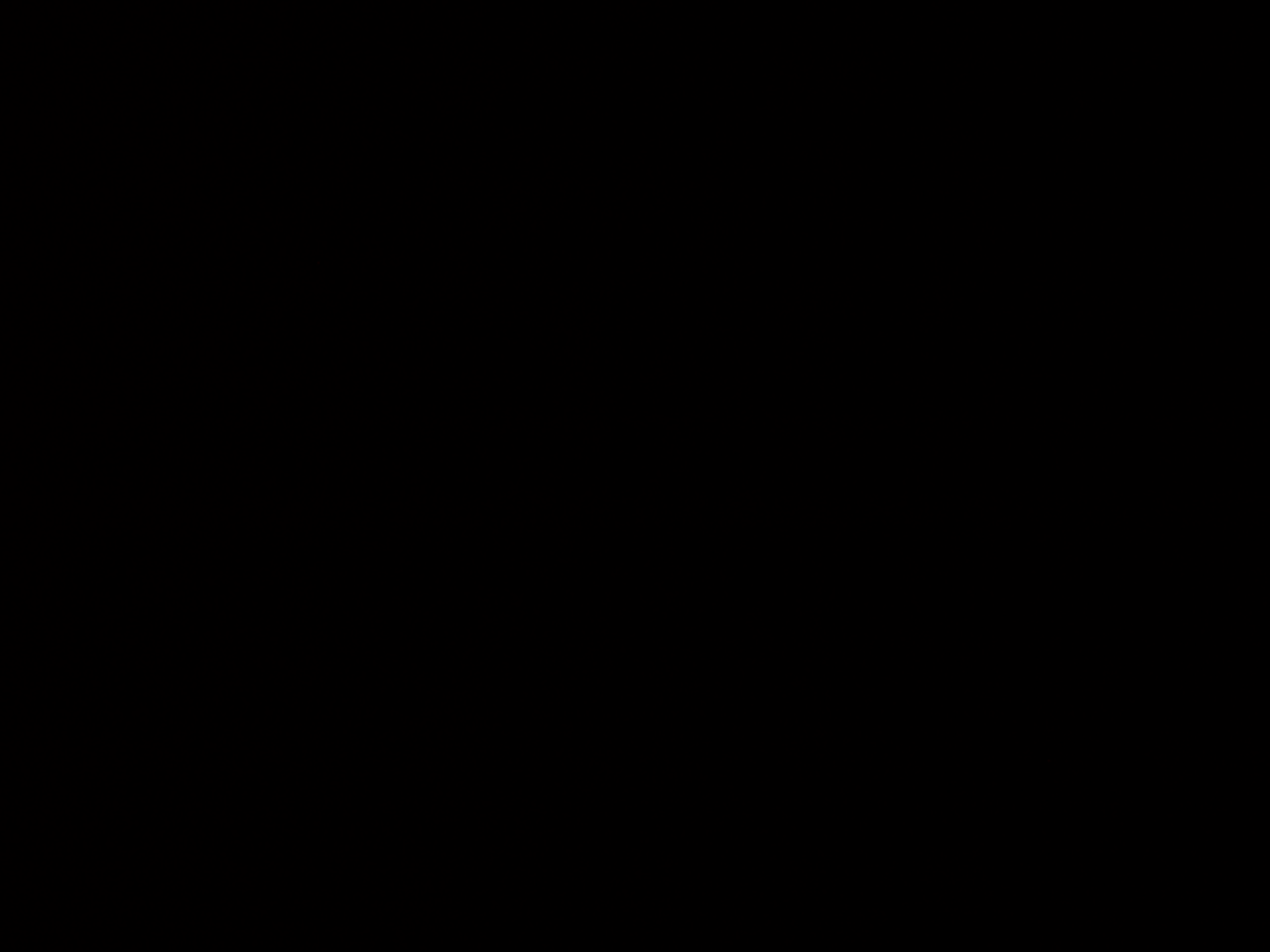

Supplement: Supplementary file 3 — Source data Fig. 1 [file 44318_2025_591_MOESM3_ESM.zip › Figure 1/1A/37_24 h_╬▒-Syn(╬▒-Syn).tif]

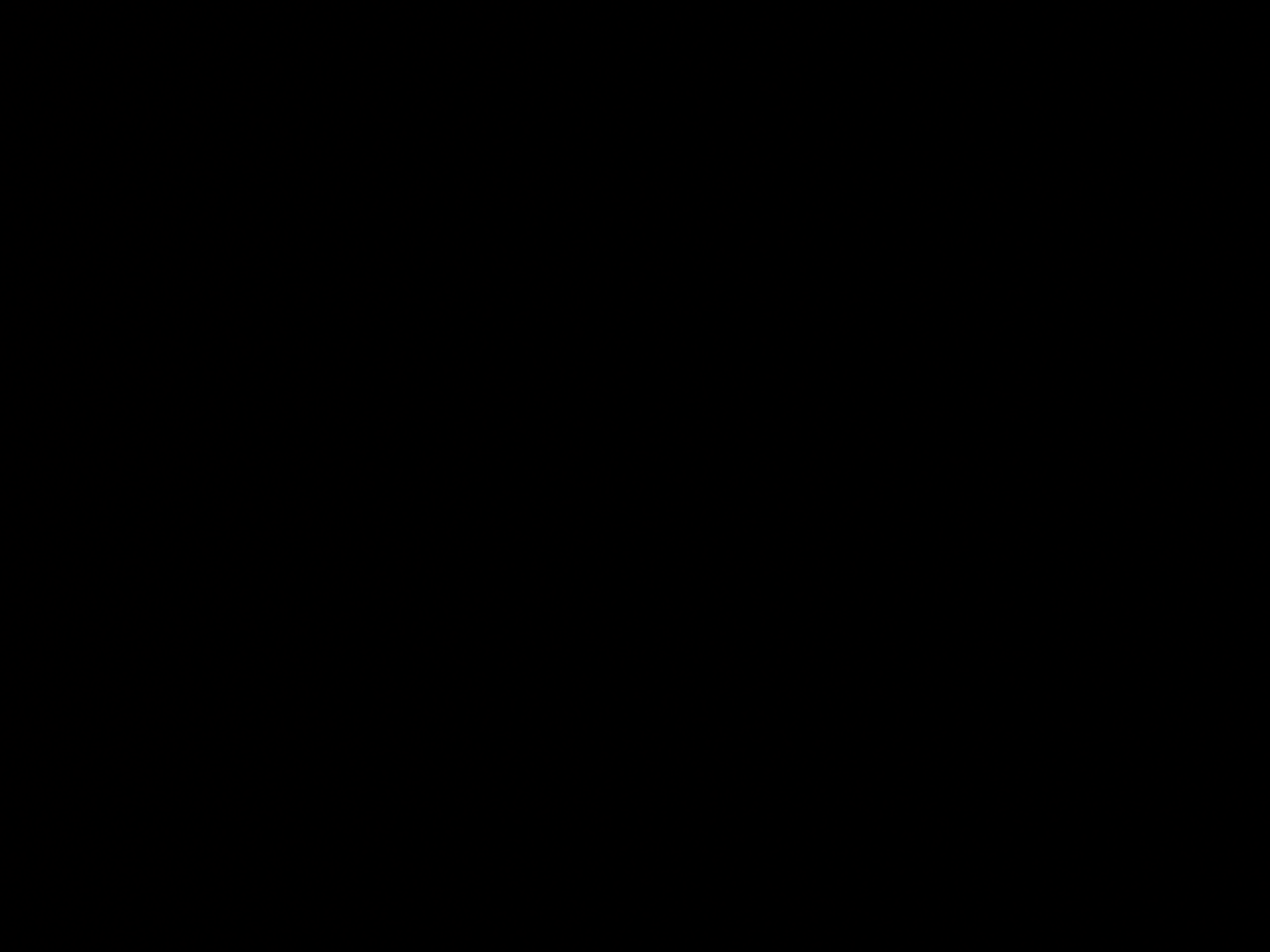

Supplement: Supplementary file 3 — Source data Fig. 1 [file 44318_2025_591_MOESM3_ESM.zip › Figure 1/1A/17_72 h_╬▒-Syn(╬▒-Syn).tif]

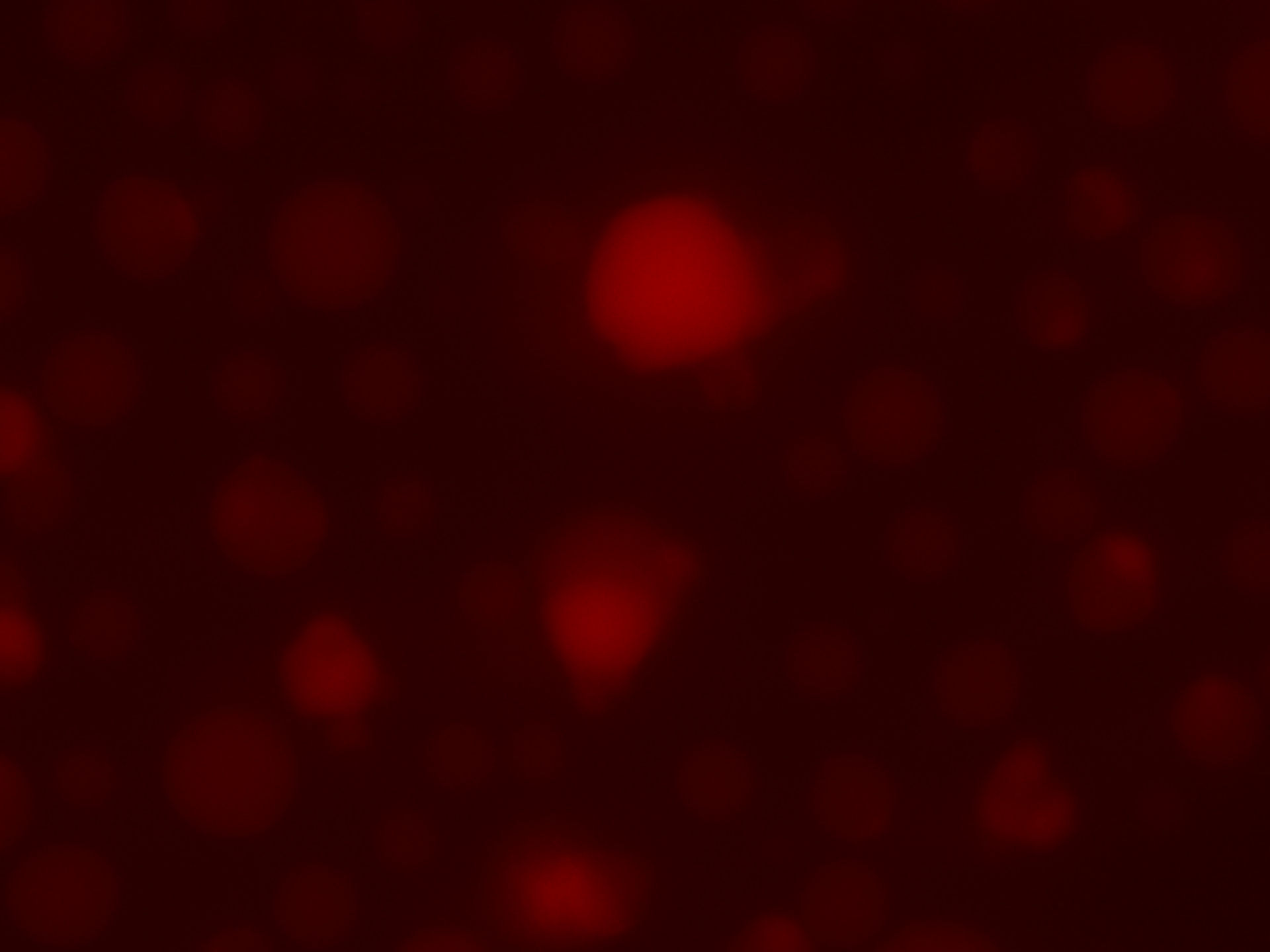

Supplement: Supplementary file 3 — Source data Fig. 1 [file 44318_2025_591_MOESM3_ESM.zip › Figure 1/1A/19_72 h_╬▒-Syn(UBQLN2+╬▒-Syn).tif]

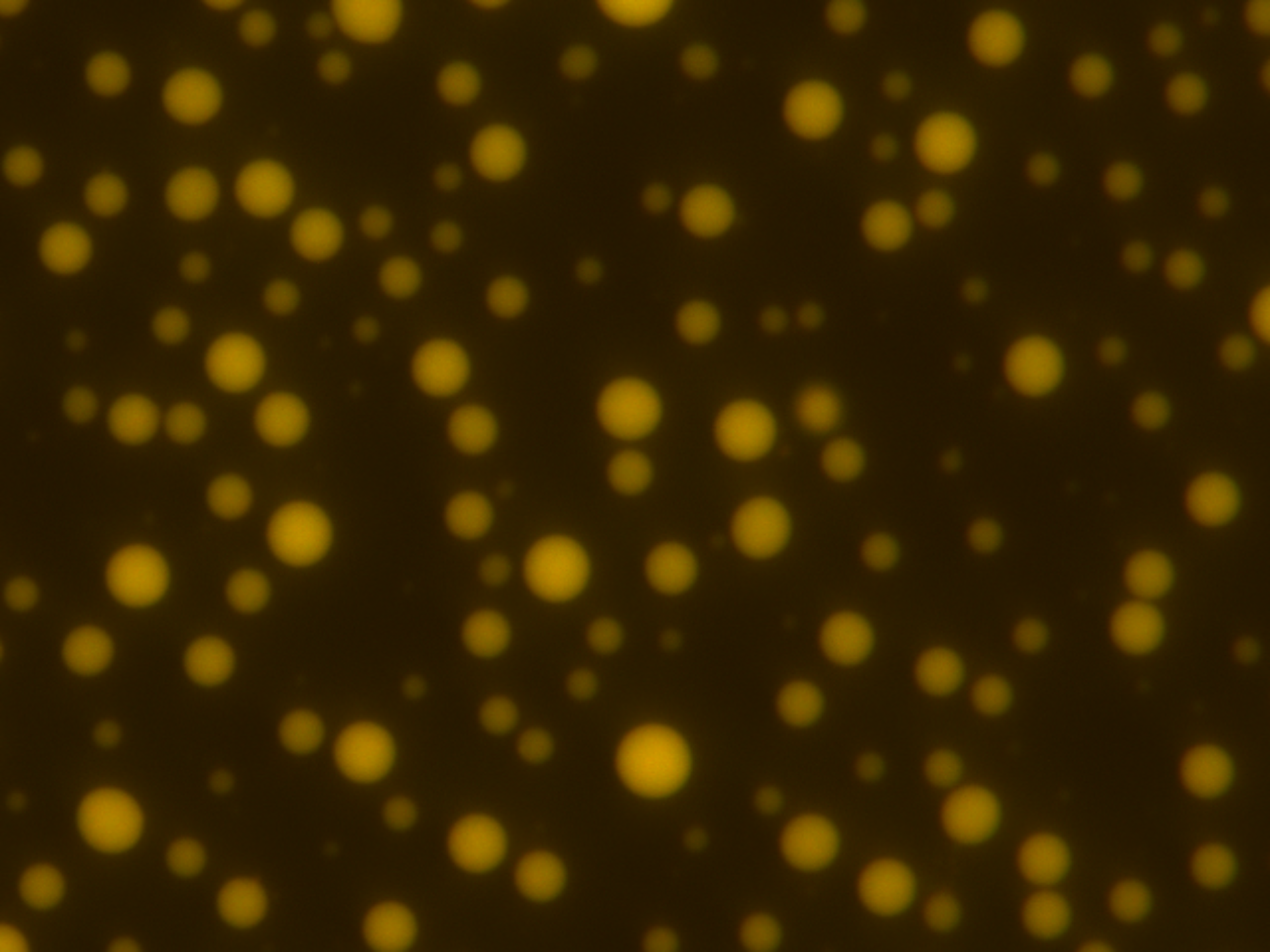

Supplement: Supplementary file 3 — Source data Fig. 1 [file 44318_2025_591_MOESM3_ESM.zip › Figure 1/1A/30_24 h_Merge(UBQLN1+╬▒-Syn).tif]

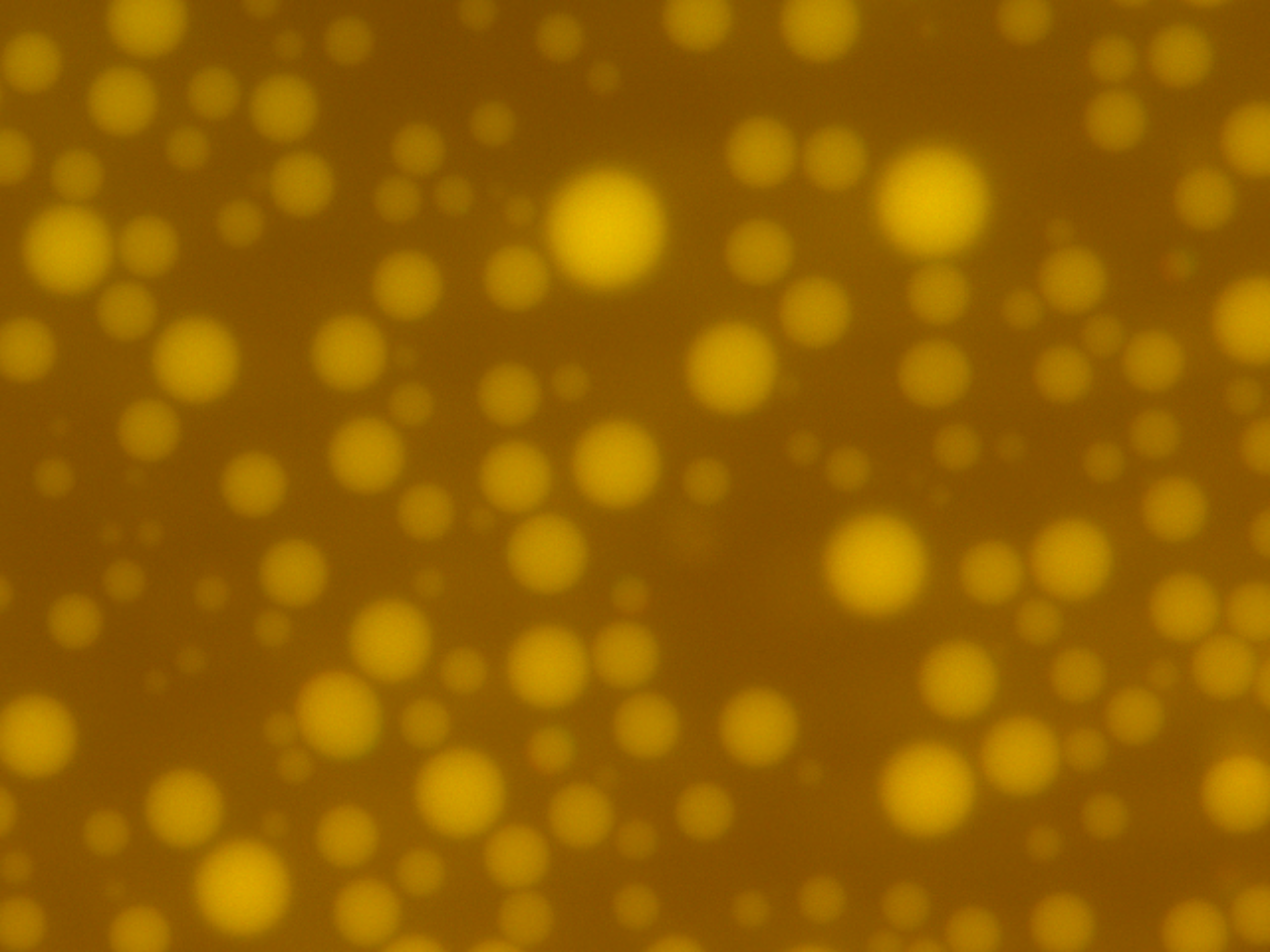

Supplement: Supplementary file 3 — Source data Fig. 1 [file 44318_2025_591_MOESM3_ESM.zip › Figure 1/1A/05_1 h_Merge(UBQLN2+╬▒-Syn).tif]

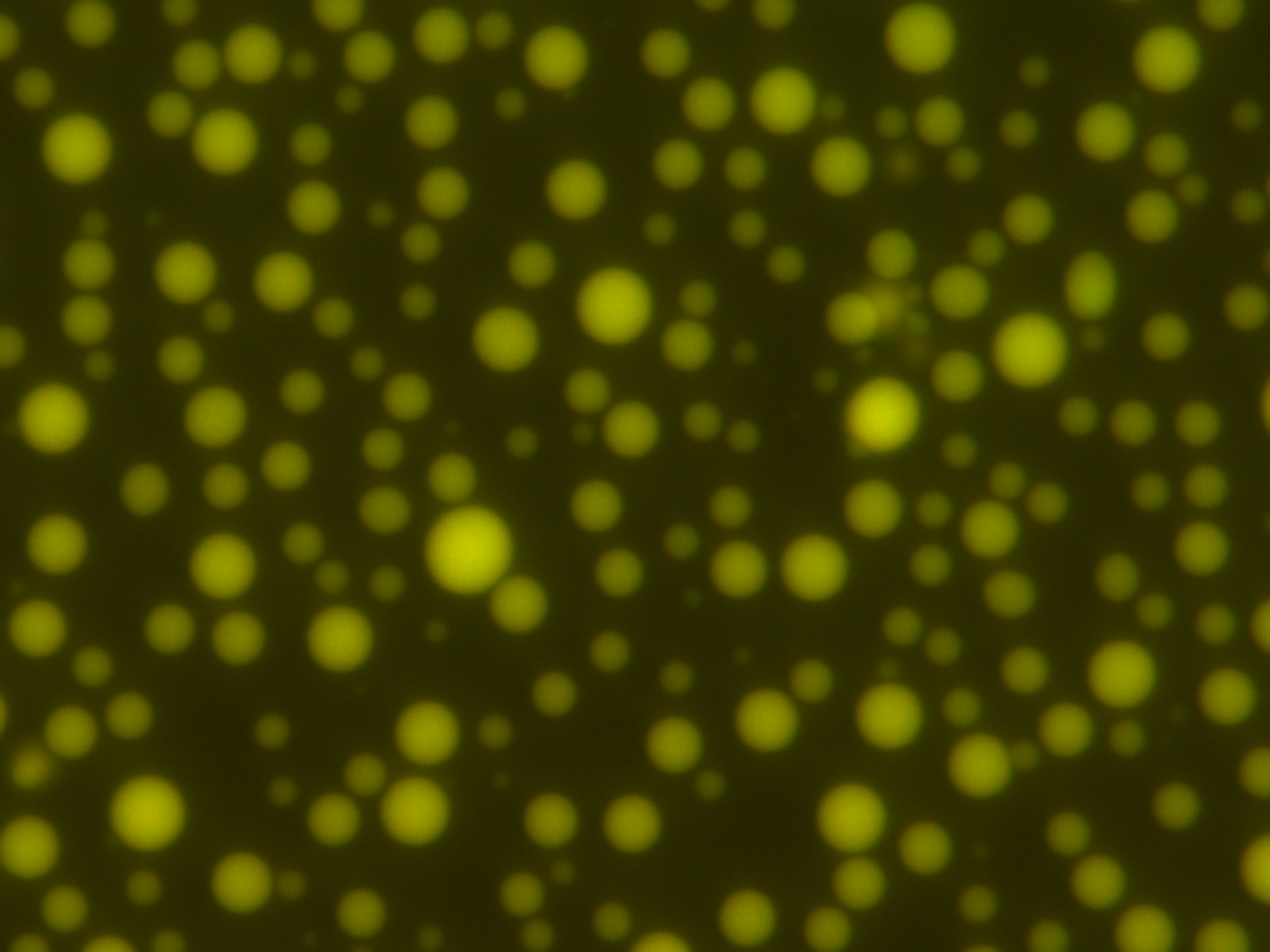

Supplement: Supplementary file 3 — Source data Fig. 1 [file 44318_2025_591_MOESM3_ESM.zip › Figure 1/1A/40_24 h_Merge(UBQLN4+╬▒-Syn).tif]

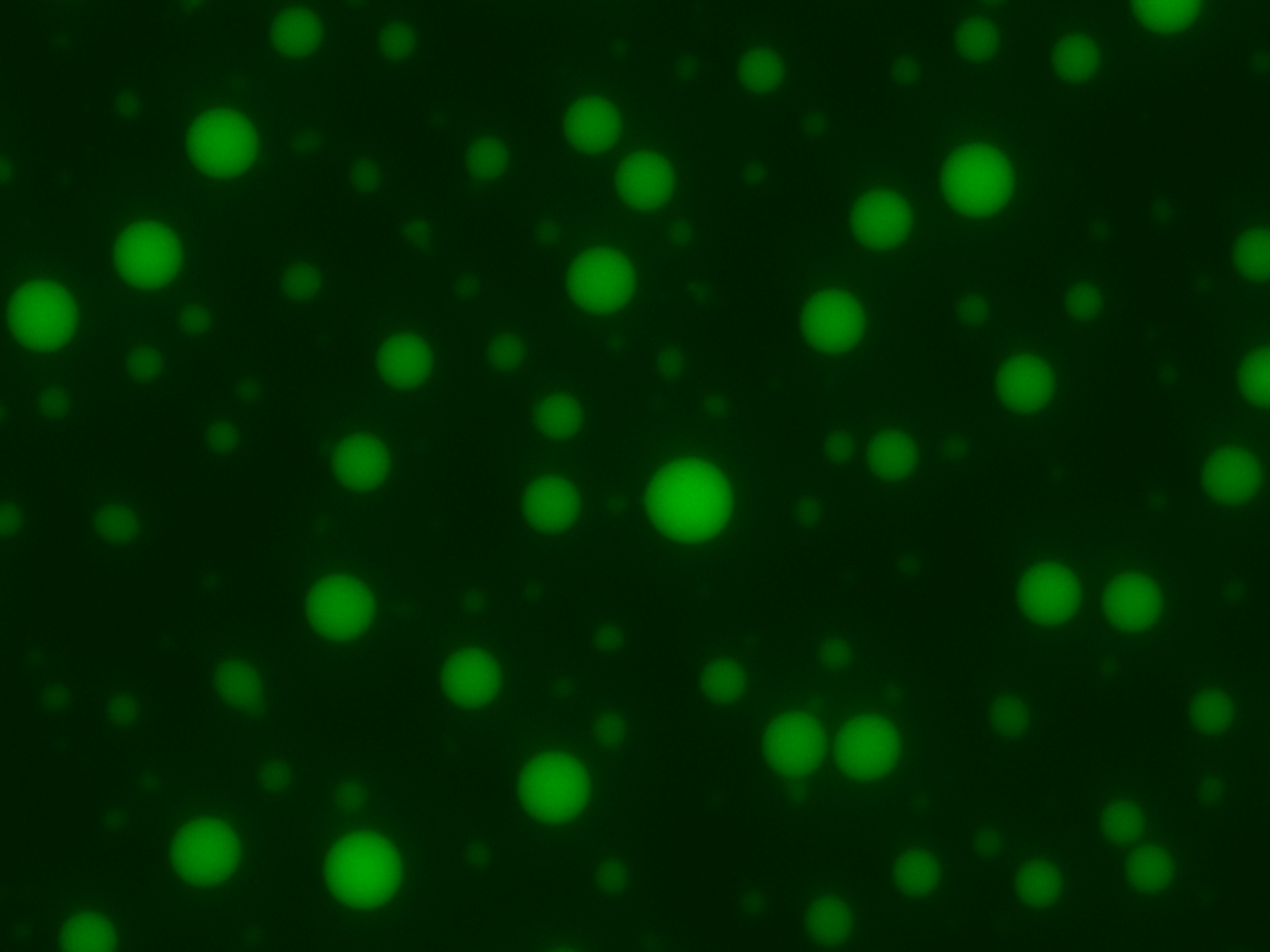

Supplement: Supplementary file 3 — Source data Fig. 1 [file 44318_2025_591_MOESM3_ESM.zip › Figure 1/1A/33_96 h_UBQLN1(UBQLN1+╬▒-Syn).tif]

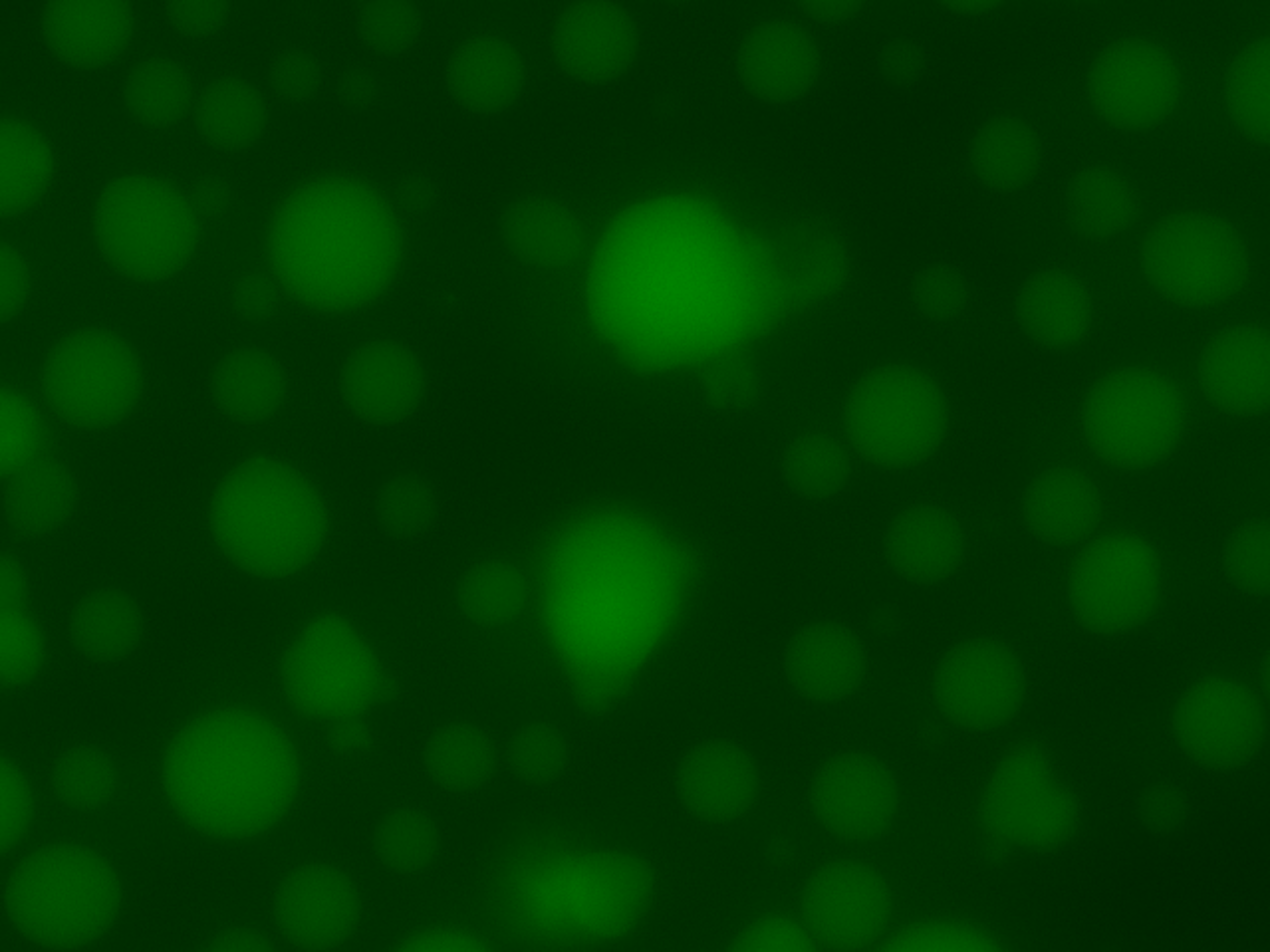

Supplement: Supplementary file 3 — Source data Fig. 1 [file 44318_2025_591_MOESM3_ESM.zip › Figure 1/1A/18_72 h_UBQLN2(UBQLN2+╬▒-Syn).tif]

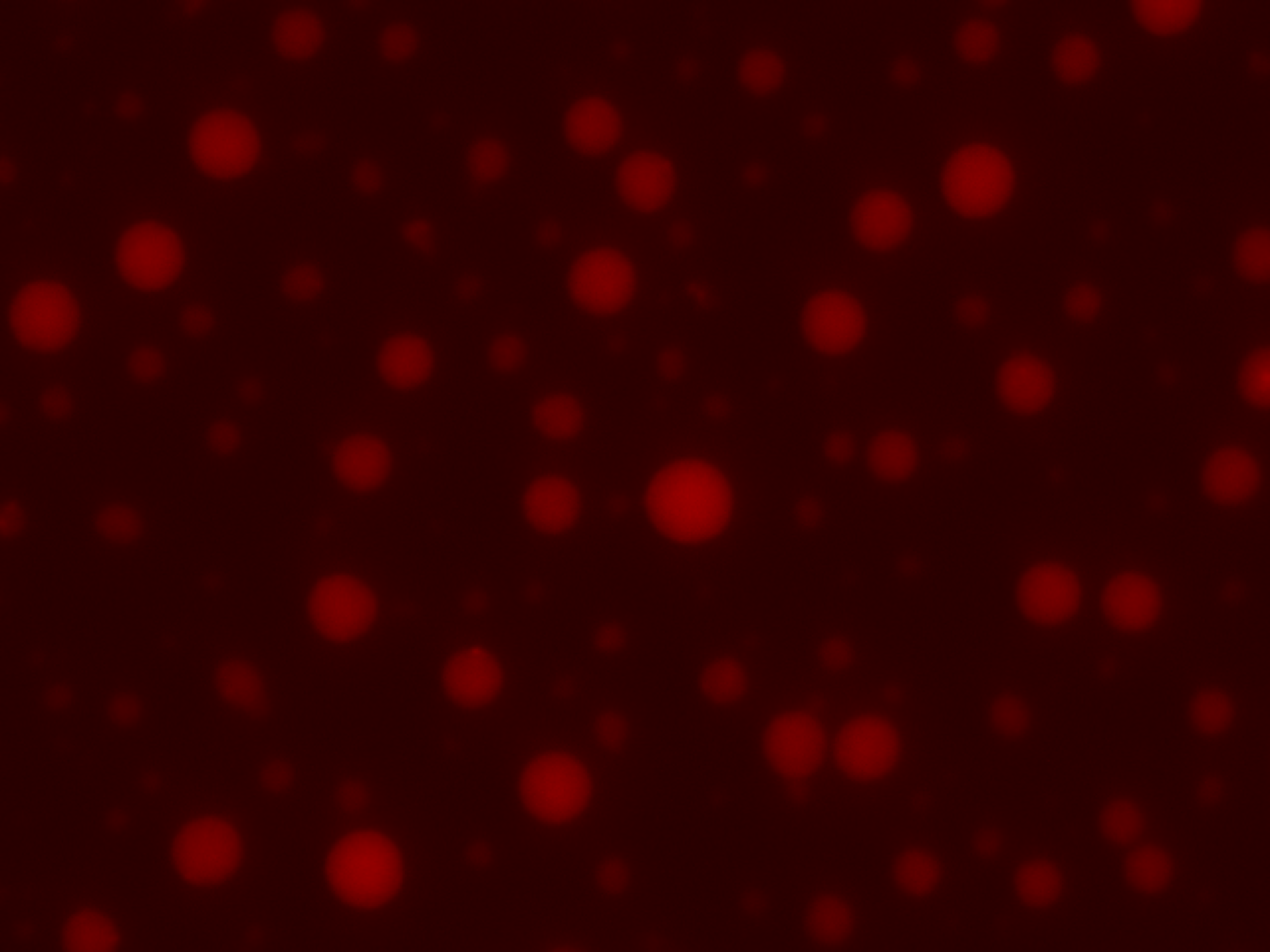

Supplement: Supplementary file 3 — Source data Fig. 1 [file 44318_2025_591_MOESM3_ESM.zip › Figure 1/1A/34_96 h_╬▒-Syn(UBQLN1+╬▒-Syn).tif]

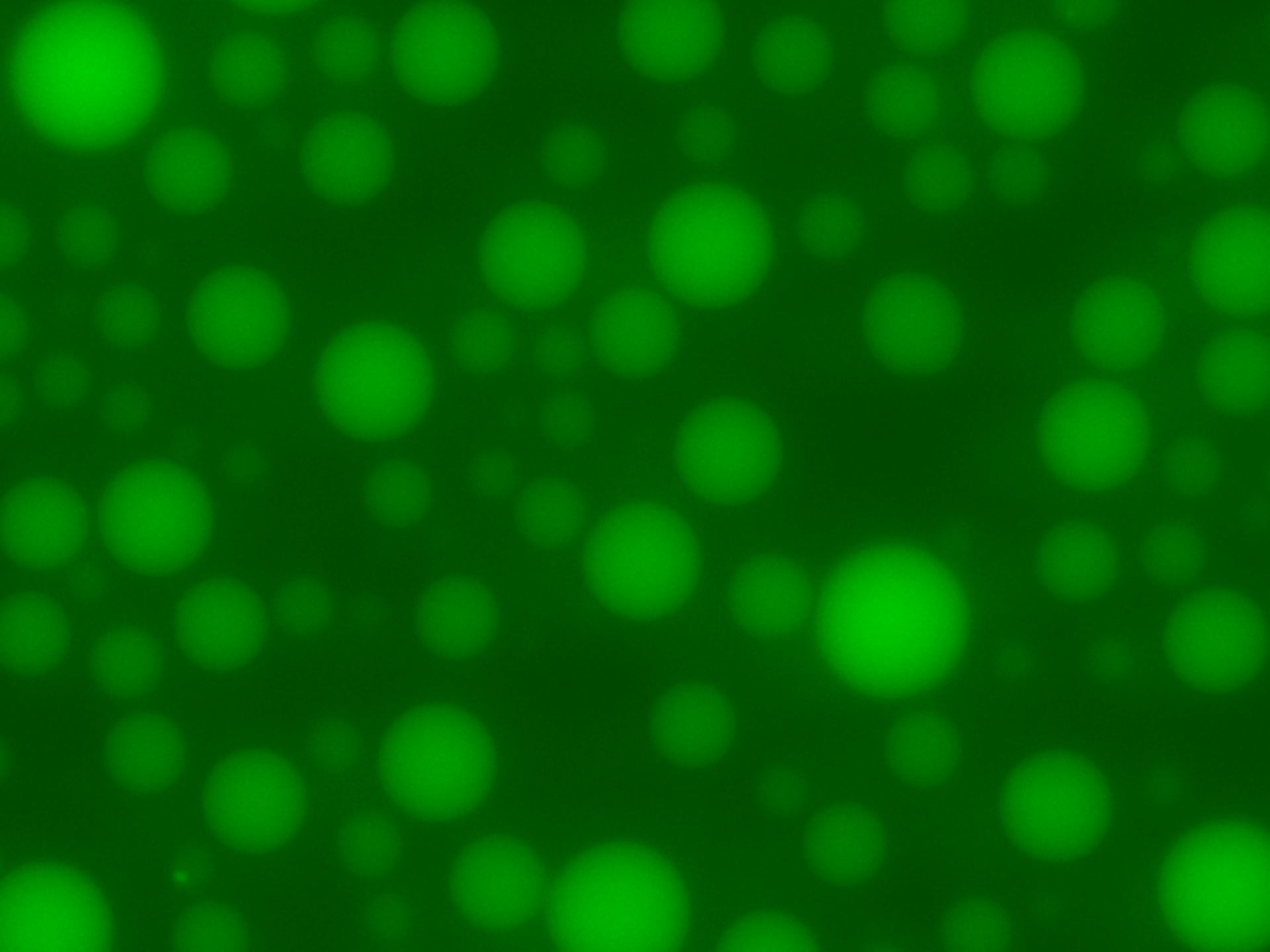

Supplement: Supplementary file 3 — Source data Fig. 1 [file 44318_2025_591_MOESM3_ESM.zip › Figure 1/1A/08_24 h_UBQLN2(UBQLN2+╬▒-Syn).tif]

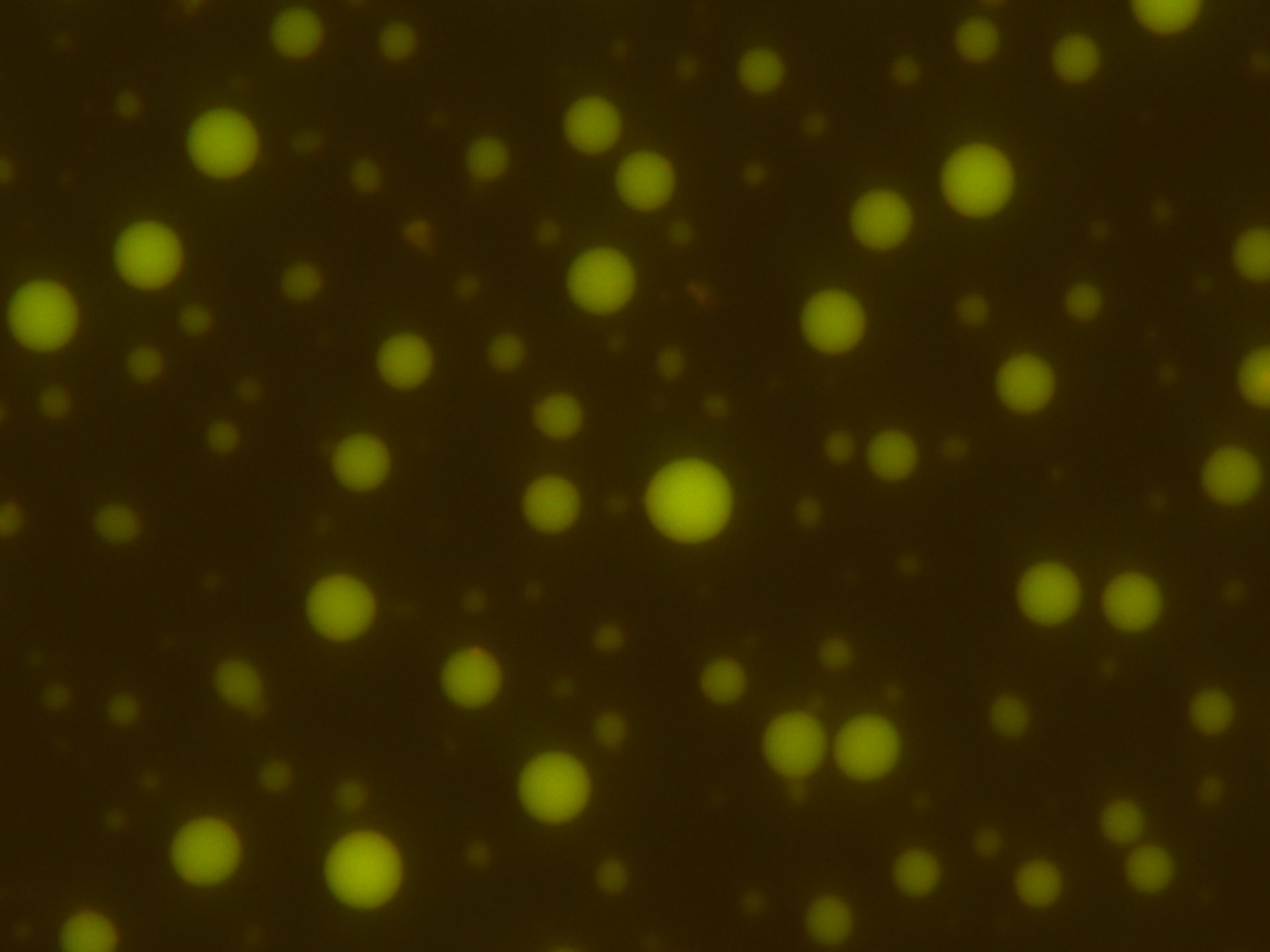

Supplement: Supplementary file 3 — Source data Fig. 1 [file 44318_2025_591_MOESM3_ESM.zip › Figure 1/1A/35_96 h_Merge(UBQLN1+╬▒-Syn).tif]

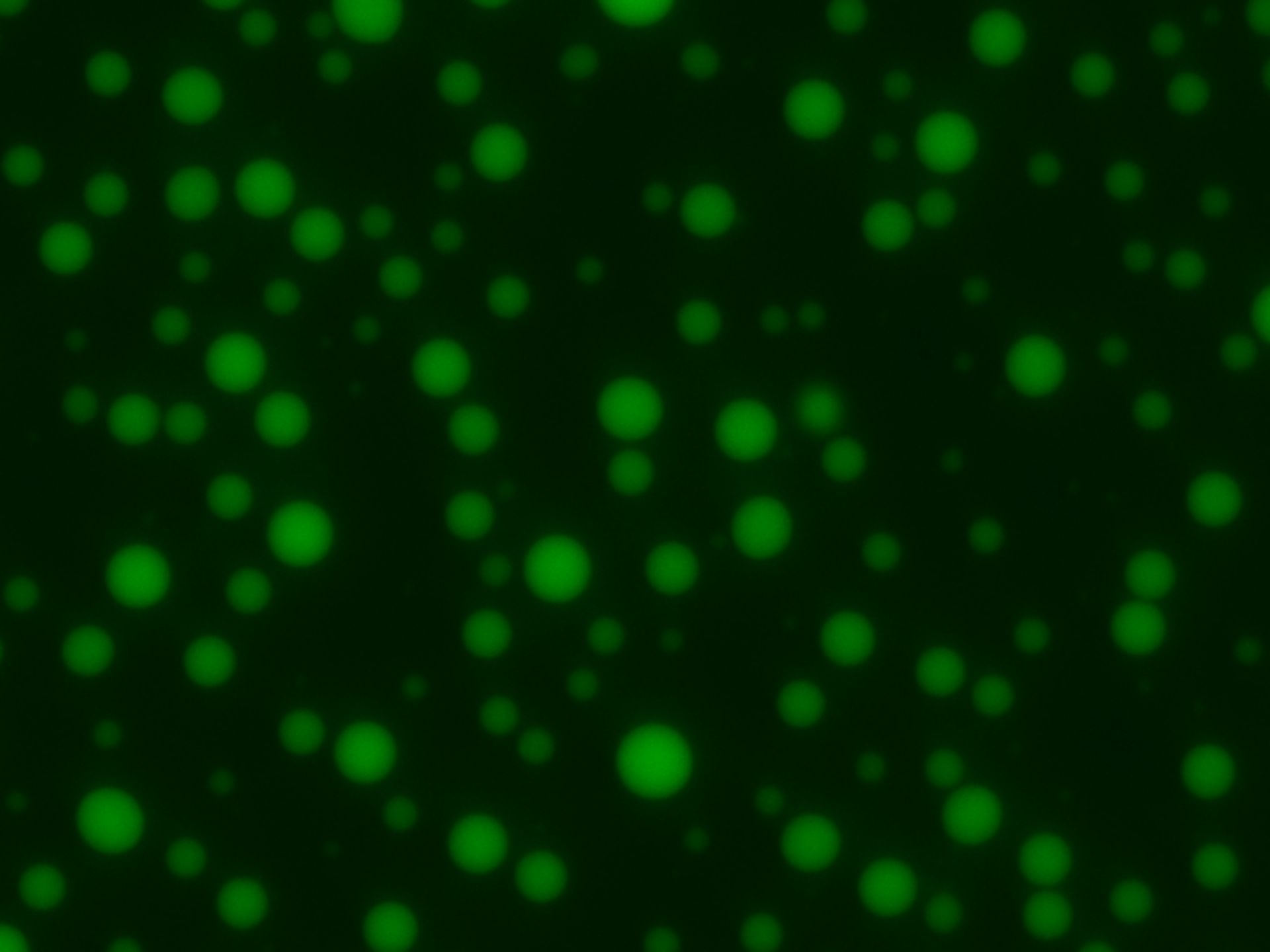

Supplement: Supplementary file 3 — Source data Fig. 1 [file 44318_2025_591_MOESM3_ESM.zip › Figure 1/1A/28_24 h_UBQLN1(UBQLN1+╬▒-Syn).tif]

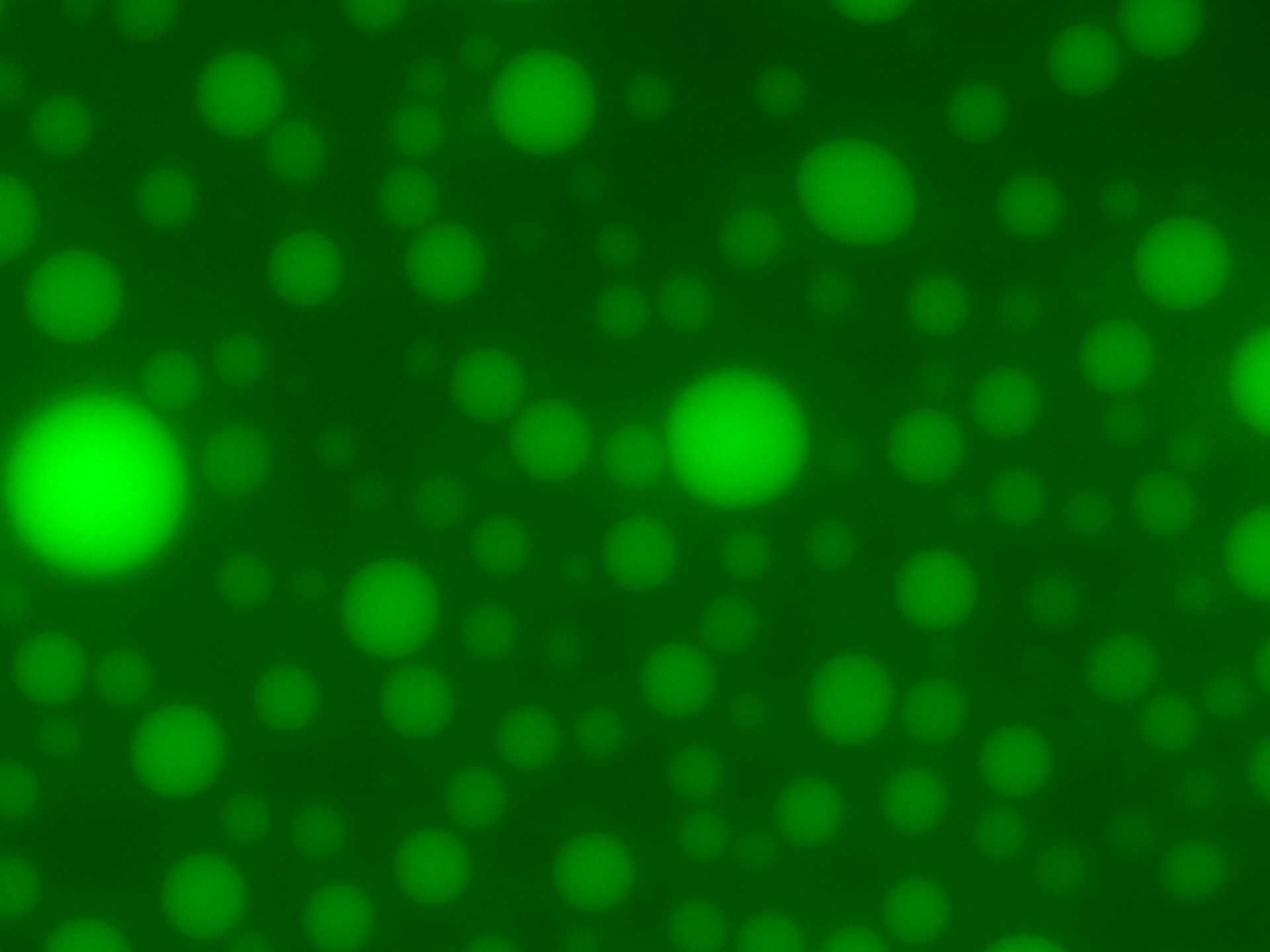

Supplement: Supplementary file 3 — Source data Fig. 1 [file 44318_2025_591_MOESM3_ESM.zip › Figure 1/1A/06_24 h_UBQLN2(UBQLN2).tif]

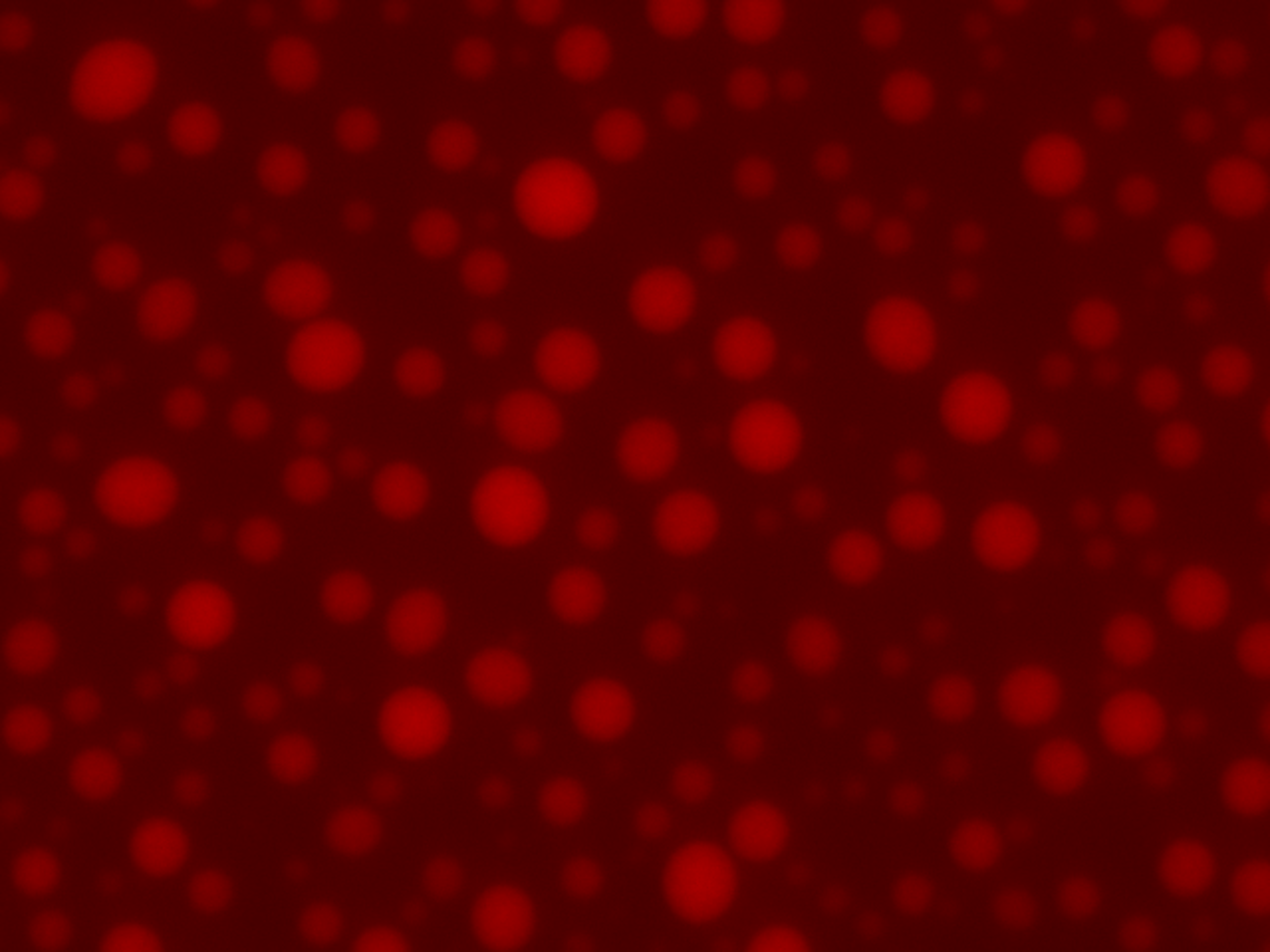

Supplement: Supplementary file 3 — Source data Fig. 1 [file 44318_2025_591_MOESM3_ESM.zip › Figure 1/1A/44_96 h_╬▒-Syn(UBQLN4+╬▒-Syn).tif]

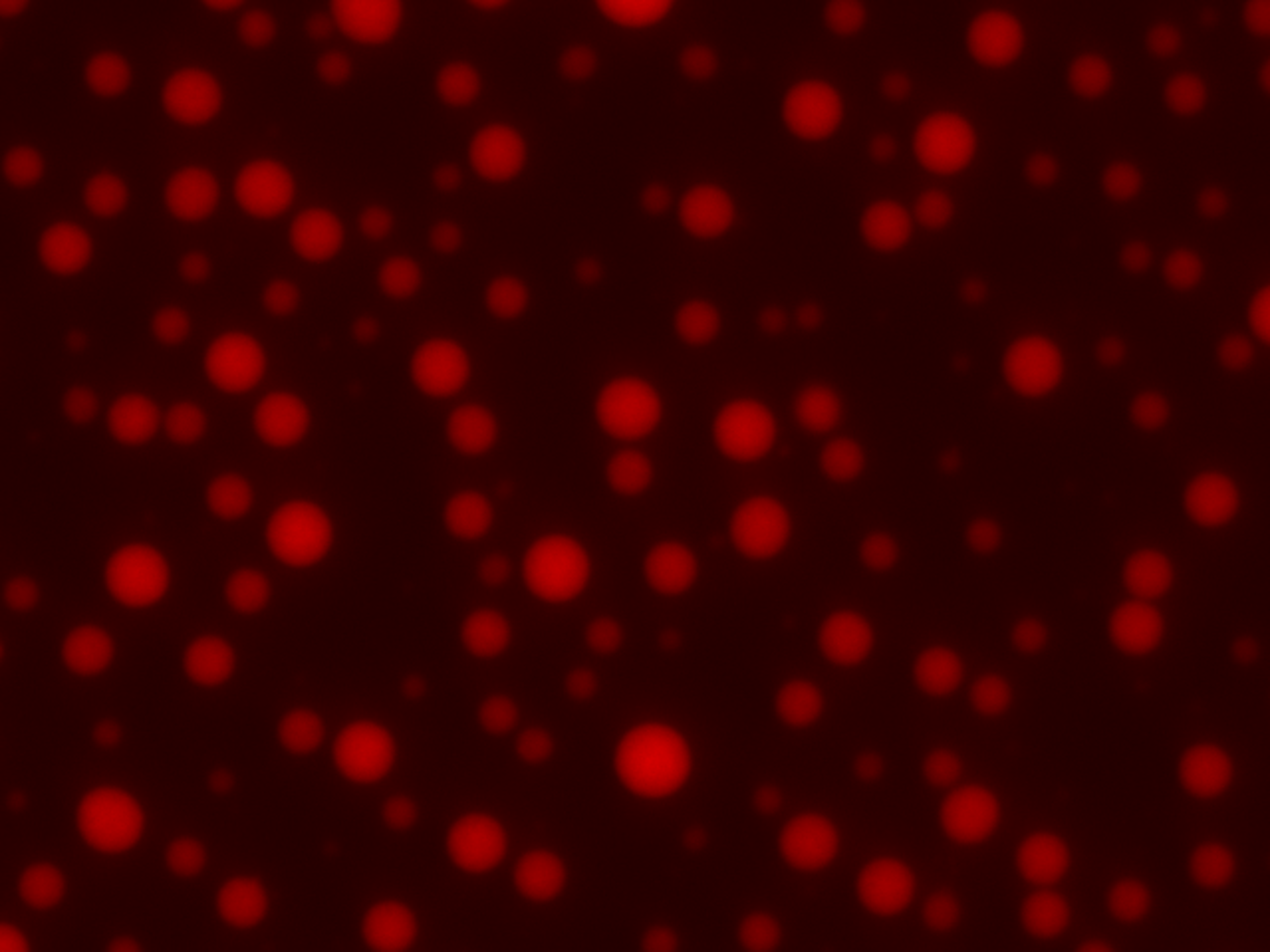

Supplement: Supplementary file 3 — Source data Fig. 1 [file 44318_2025_591_MOESM3_ESM.zip › Figure 1/1A/29_24 h_╬▒-Syn(UBQLN1+╬▒-Syn).tif]

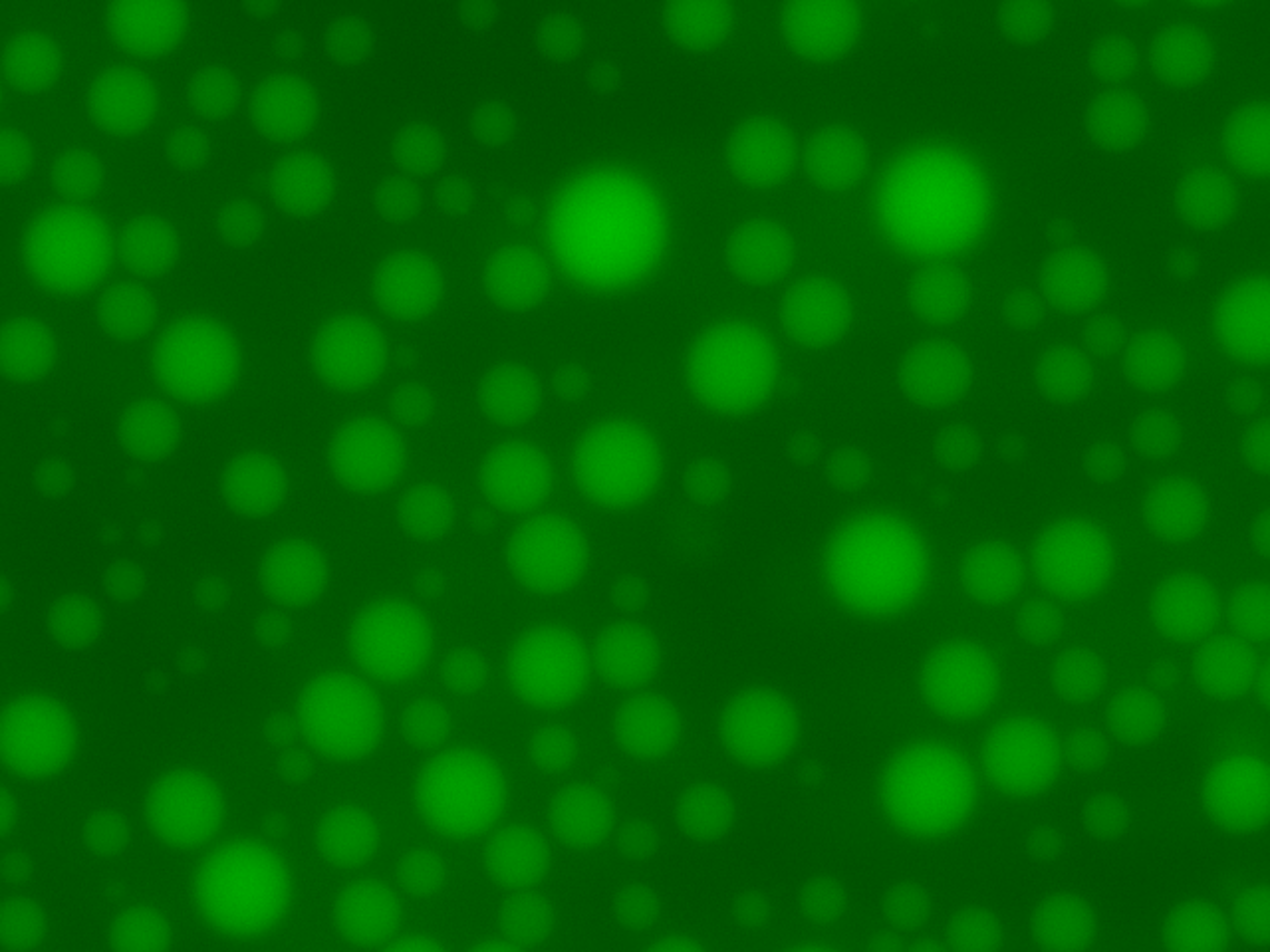

Supplement: Supplementary file 3 — Source data Fig. 1 [file 44318_2025_591_MOESM3_ESM.zip › Figure 1/1A/03_1 h_UBQLN2(UBQLN2+╬▒-Syn).tif]

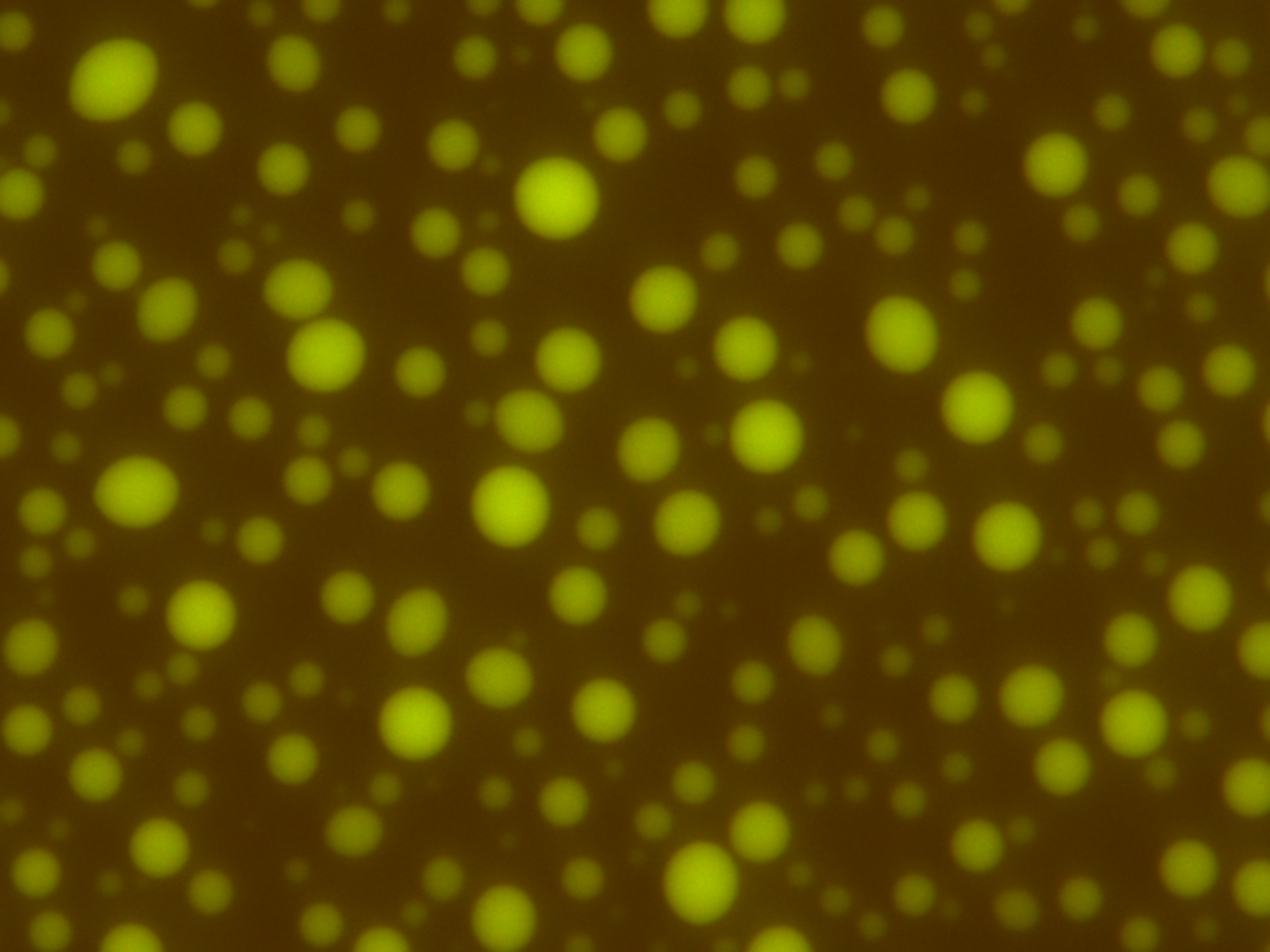

Supplement: Supplementary file 3 — Source data Fig. 1 [file 44318_2025_591_MOESM3_ESM.zip › Figure 1/1A/45_96 h_Merge(UBQLN4+╬▒-Syn).tif]

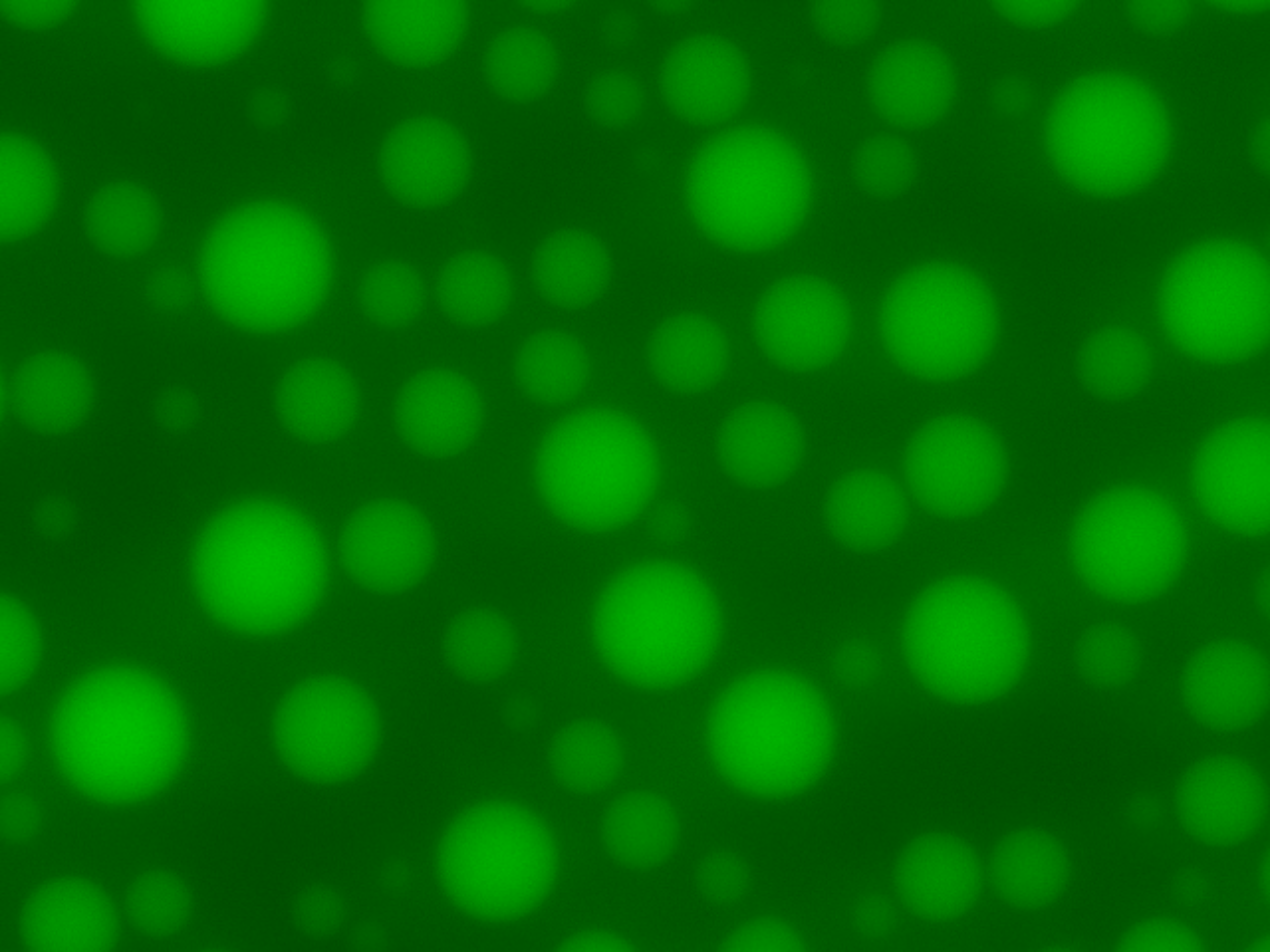

Supplement: Supplementary file 3 — Source data Fig. 1 [file 44318_2025_591_MOESM3_ESM.zip › Figure 1/1A/13_48 h_UBQLN2(UBQLN2+╬▒-Syn).tif]

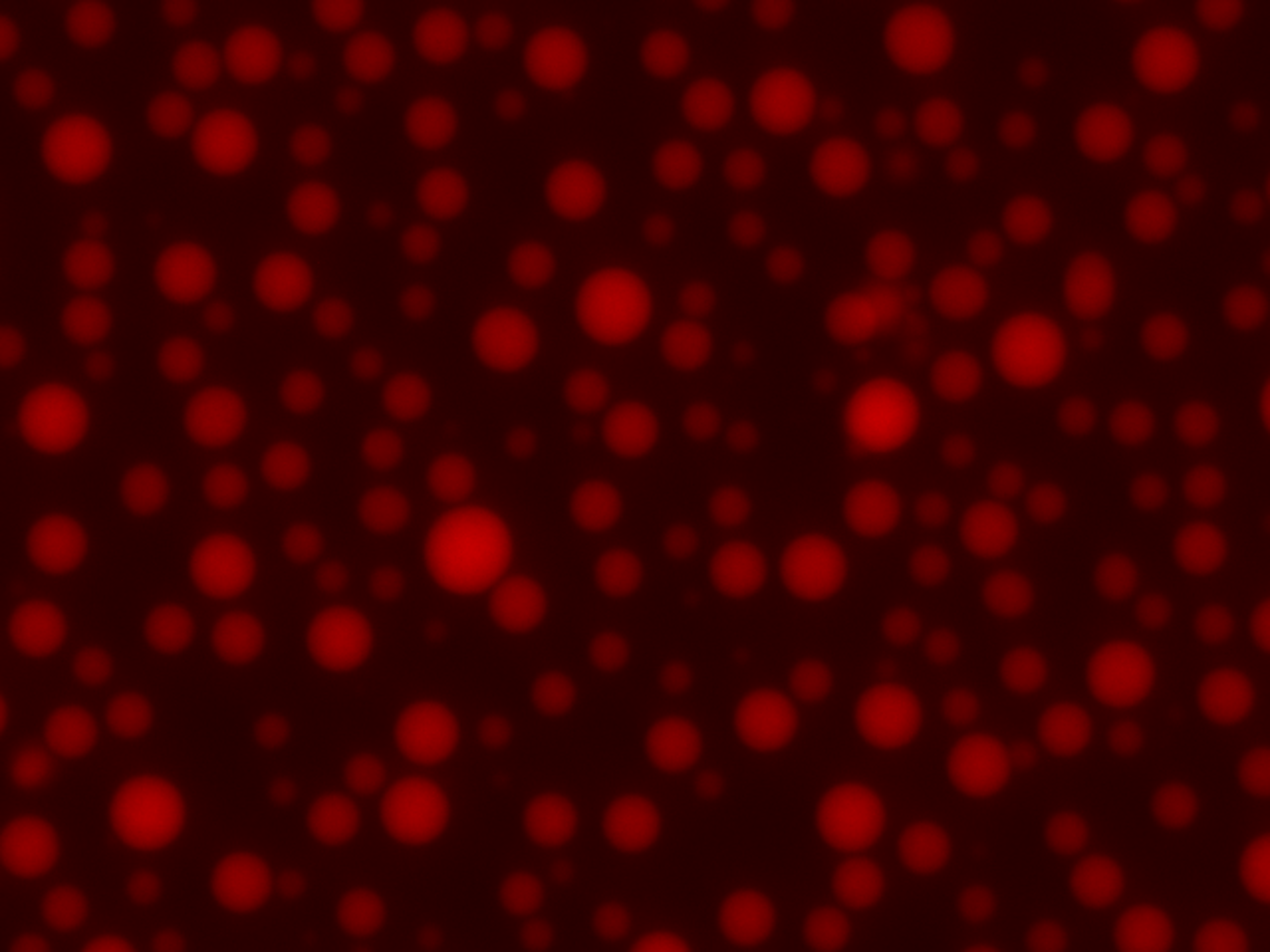

Supplement: Supplementary file 3 — Source data Fig. 1 [file 44318_2025_591_MOESM3_ESM.zip › Figure 1/1A/39_24 h_╬▒-Syn(UBQLN4+╬▒-Syn).tif]

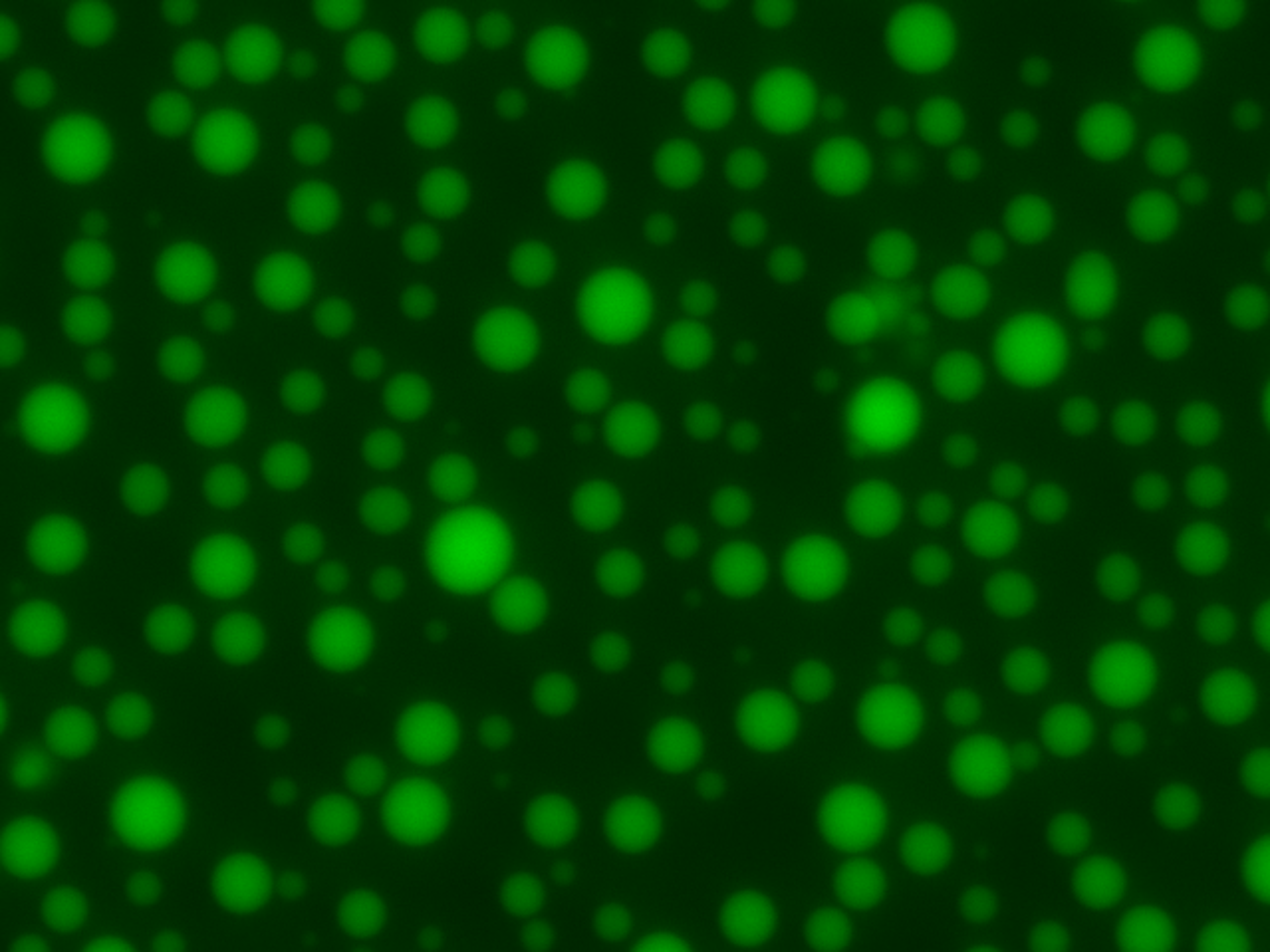

Supplement: Supplementary file 3 — Source data Fig. 1 [file 44318_2025_591_MOESM3_ESM.zip › Figure 1/1A/38_24 h_UBQLN4(UBQLN4+╬▒-Syn).tif]

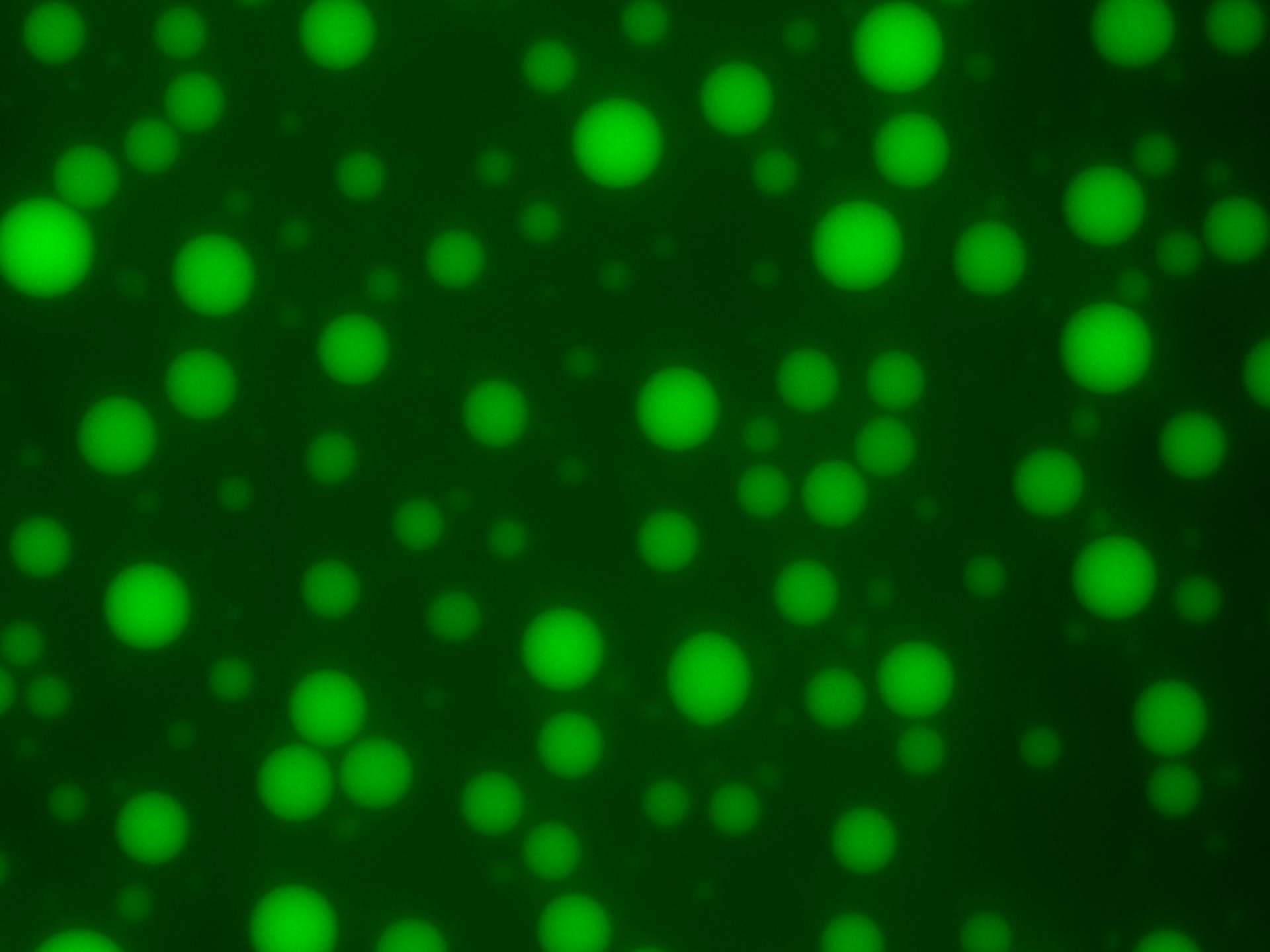

Supplement: Supplementary file 3 — Source data Fig. 1 [file 44318_2025_591_MOESM3_ESM.zip › Figure 1/1A/31_96 h_UBQLN1(UBQLN1).tif]

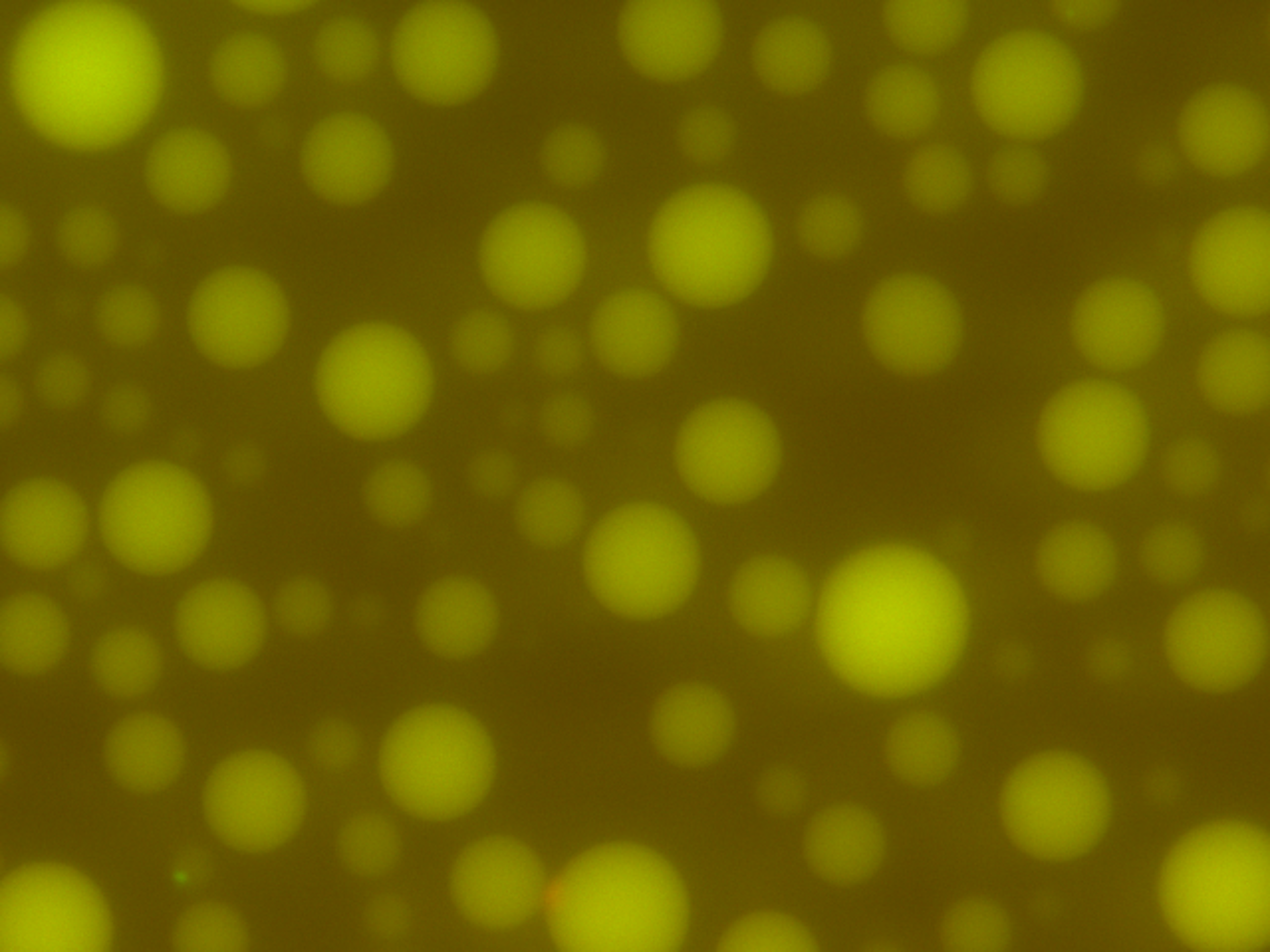

Supplement: Supplementary file 3 — Source data Fig. 1 [file 44318_2025_591_MOESM3_ESM.zip › Figure 1/1A/10_24 h_Merge(UBQLN2+╬▒-Syn).tif]

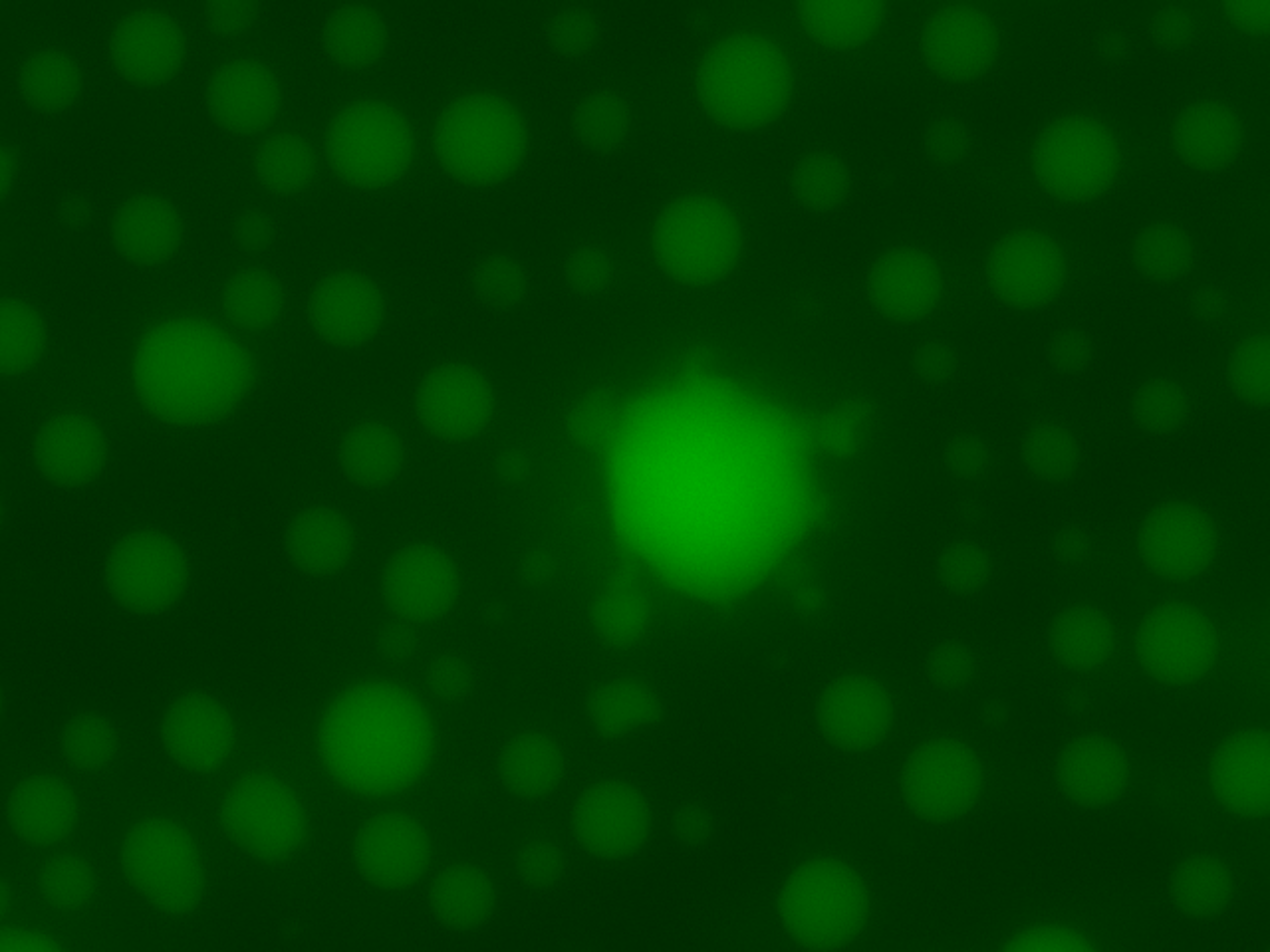

Supplement: Supplementary file 3 — Source data Fig. 1 [file 44318_2025_591_MOESM3_ESM.zip › Figure 1/1A/16_72 h_UBQLN2(UBQLN2).tif]

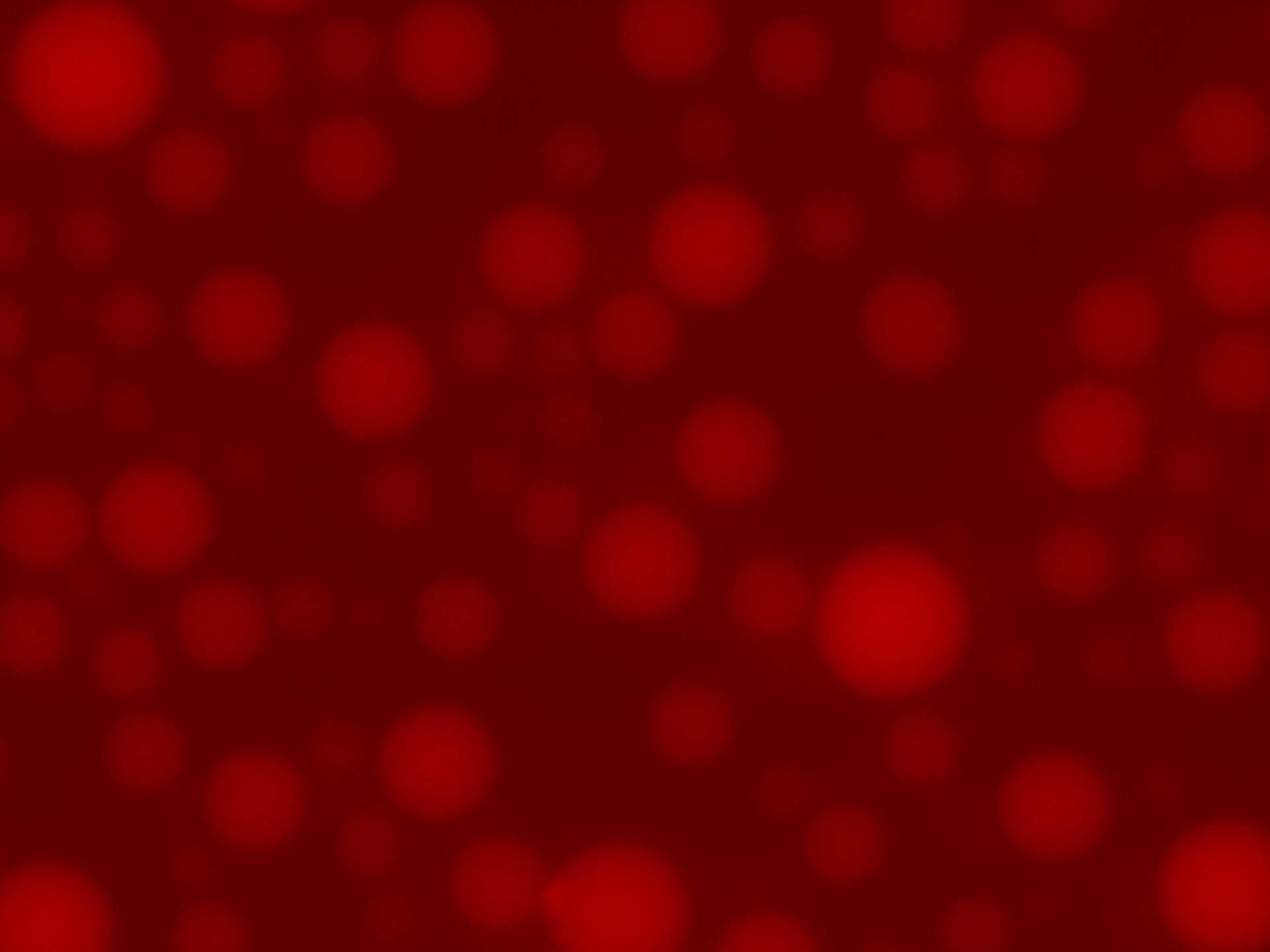

Supplement: Supplementary file 3 — Source data Fig. 1 [file 44318_2025_591_MOESM3_ESM.zip › Figure 1/1A/09_24 h_╬▒-Syn(UBQLN2+╬▒-Syn).tif]

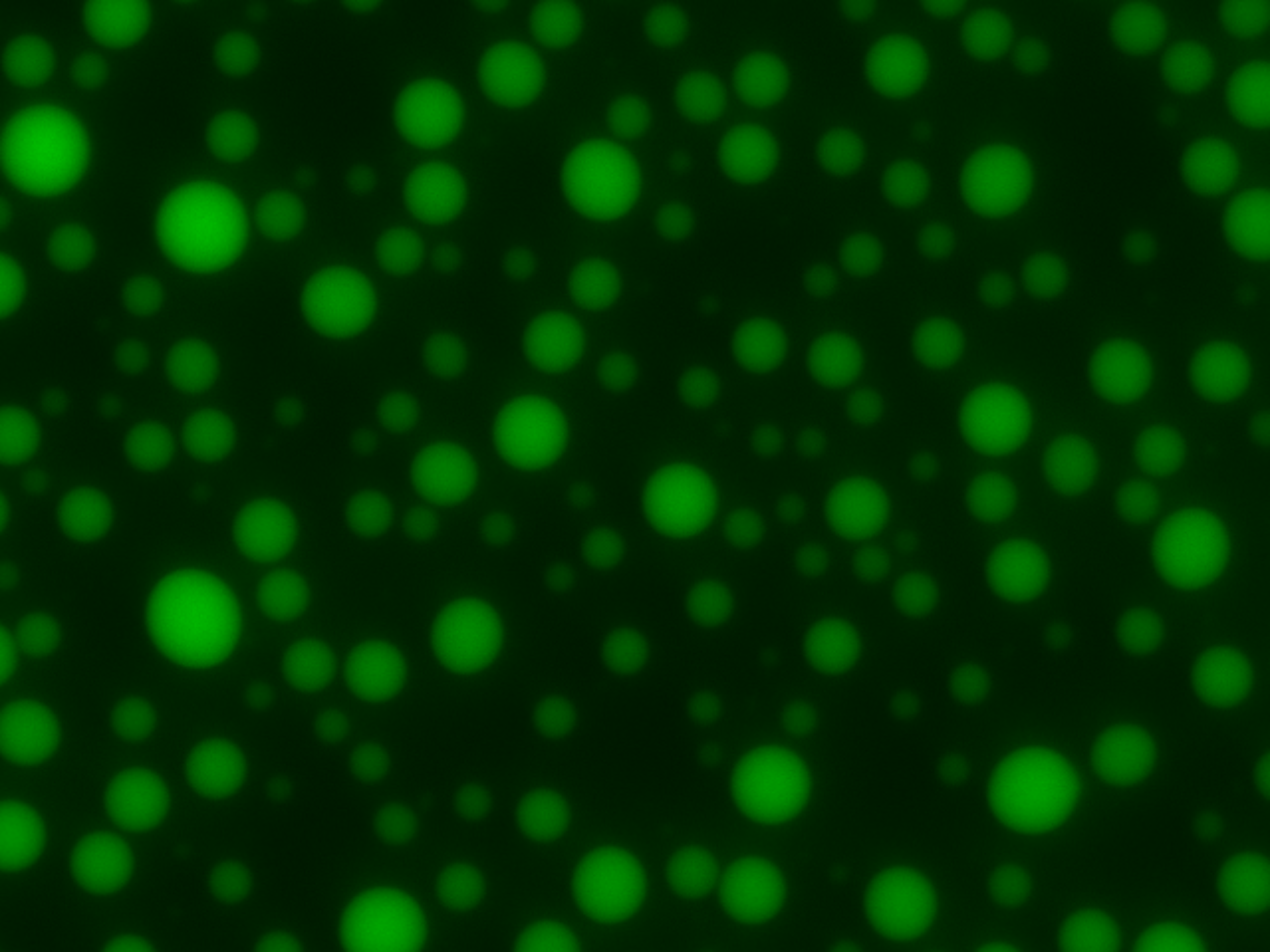

Supplement: Supplementary file 3 — Source data Fig. 1 [file 44318_2025_591_MOESM3_ESM.zip › Figure 1/1A/26_24 h_UBQLN1(UBQLN1).tif]

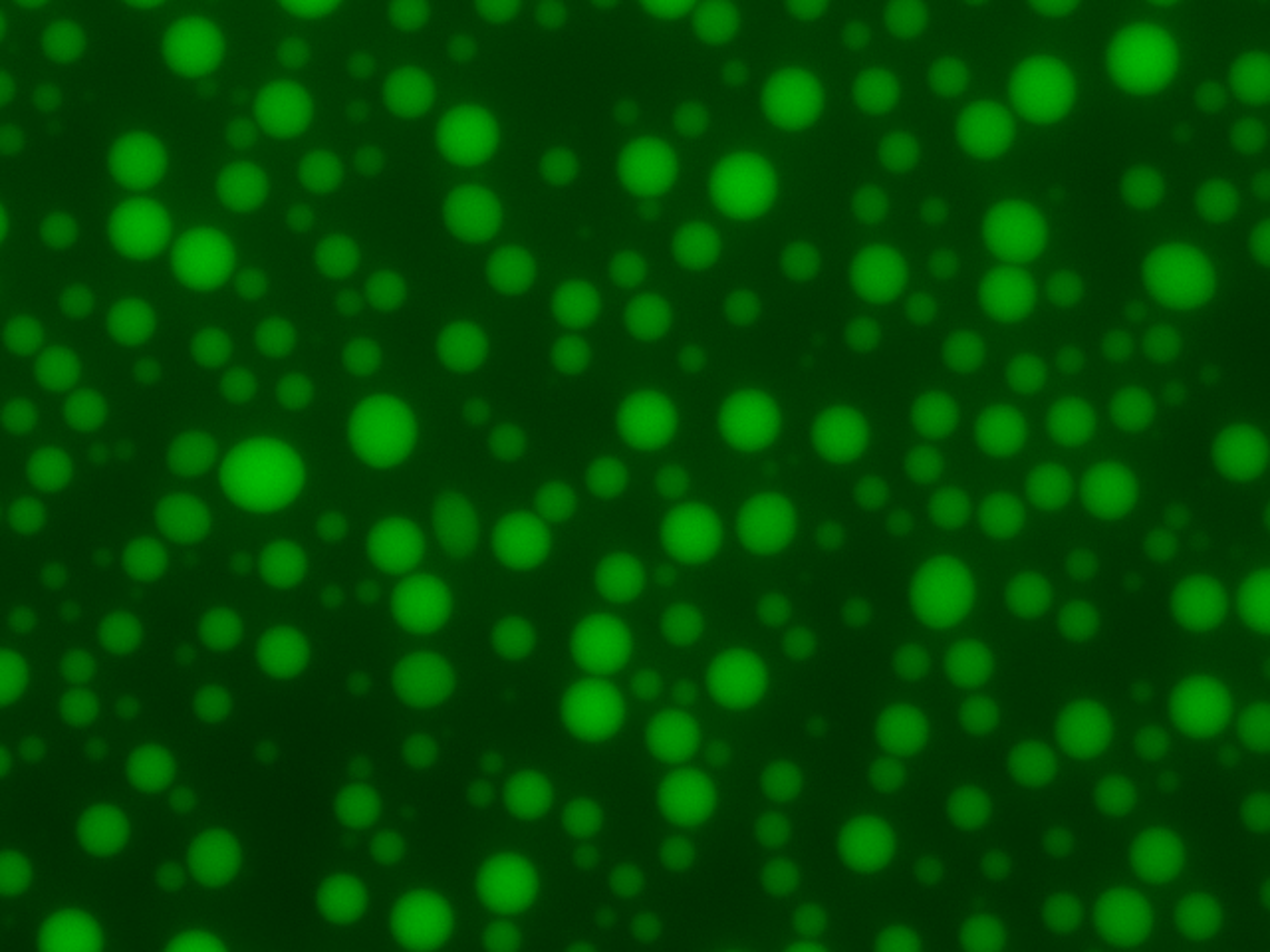

Supplement: Supplementary file 3 — Source data Fig. 1 [file 44318_2025_591_MOESM3_ESM.zip › Figure 1/1A/41_96 h_UBQLN4(UBQLN4).tif]

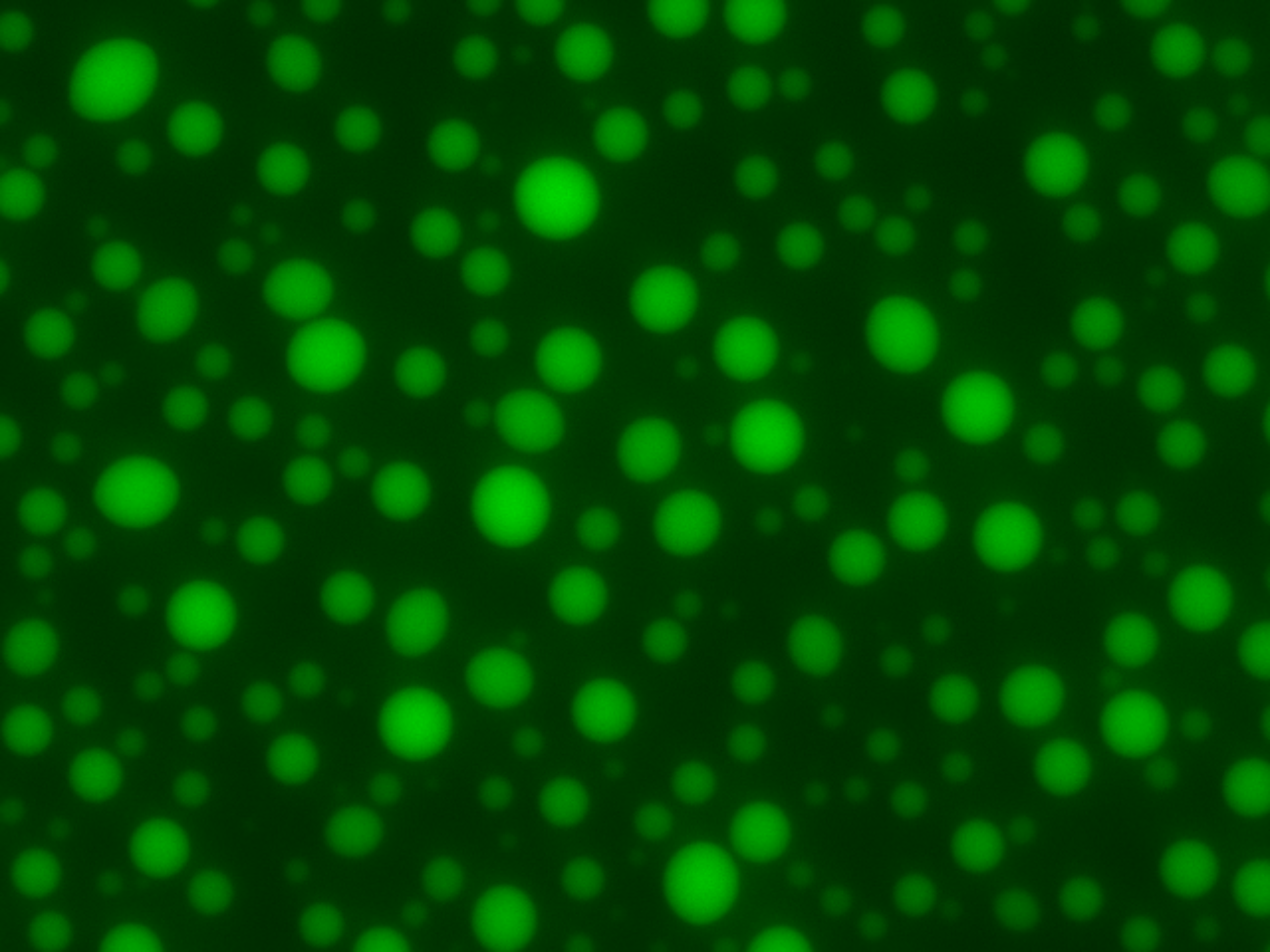

Supplement: Supplementary file 3 — Source data Fig. 1 [file 44318_2025_591_MOESM3_ESM.zip › Figure 1/1A/43_96 h_UBQLN4(UBQLN4+╬▒-Syn).tif]

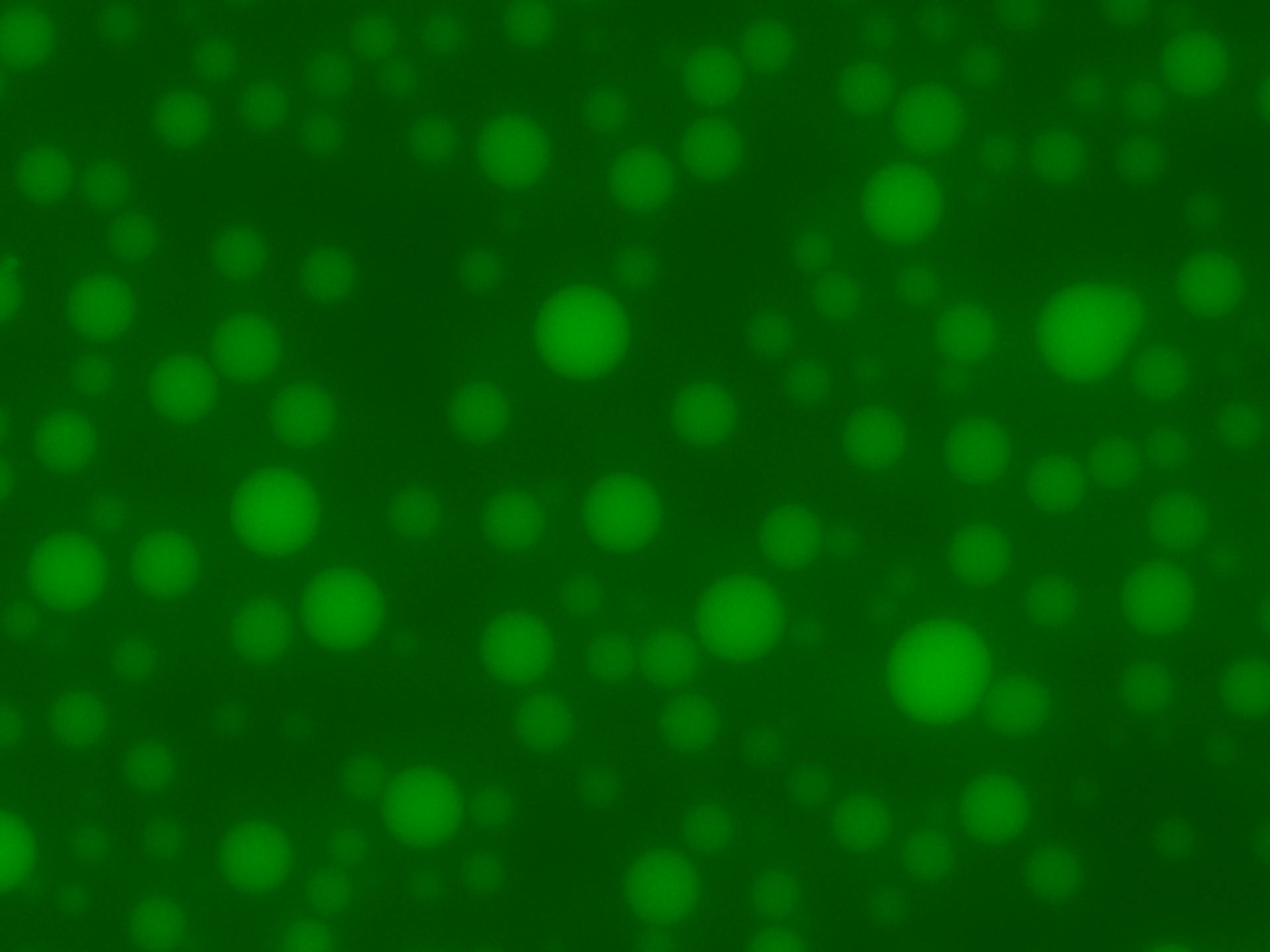

Supplement: Supplementary file 3 — Source data Fig. 1 [file 44318_2025_591_MOESM3_ESM.zip › Figure 1/1A/01_1 h_UBQLN2(UBQLN2).tif]

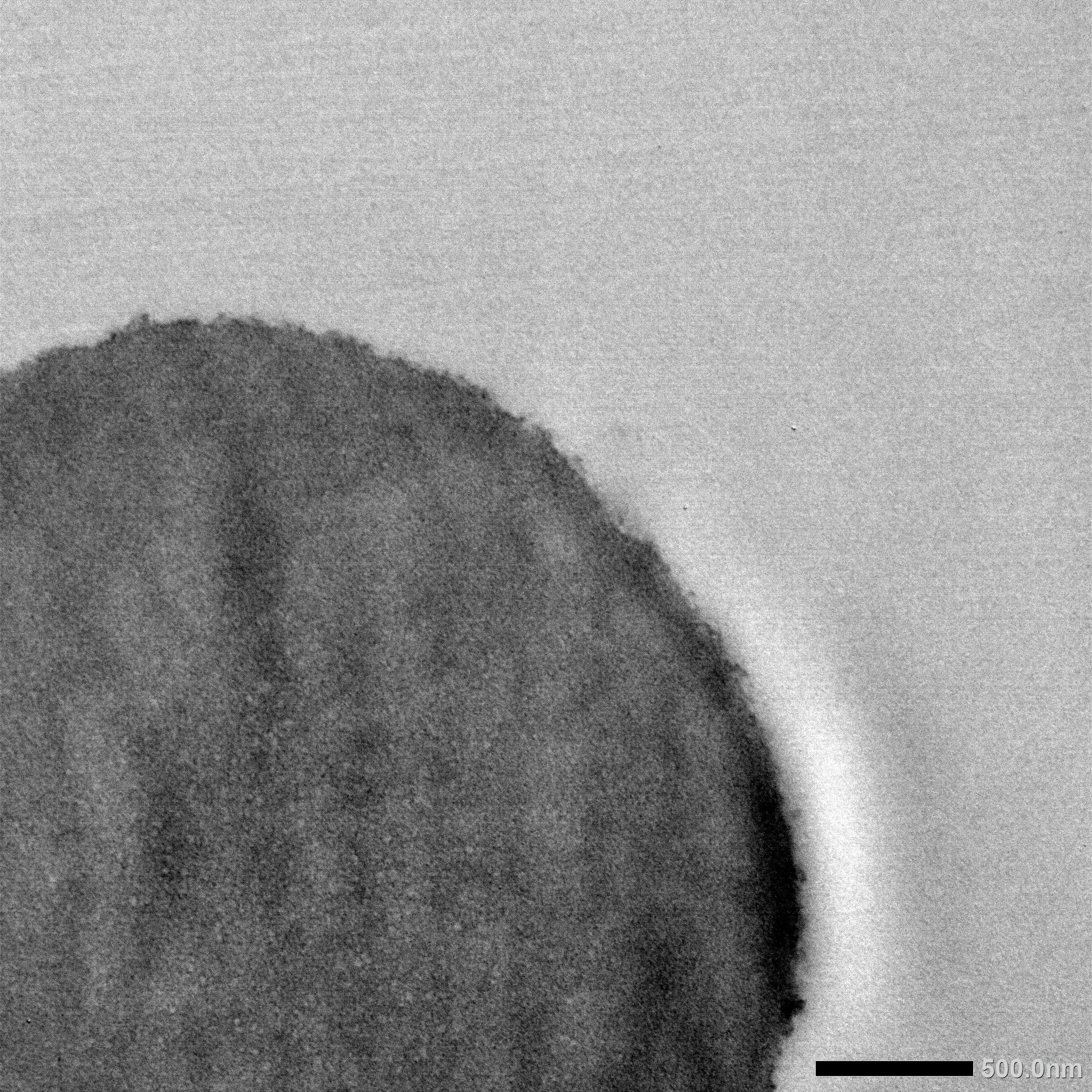

Supplement: Supplementary file 3 — Source data Fig. 1 [file 44318_2025_591_MOESM3_ESM.zip › Figure 1/1E/01_48h(#1)_01.tif]

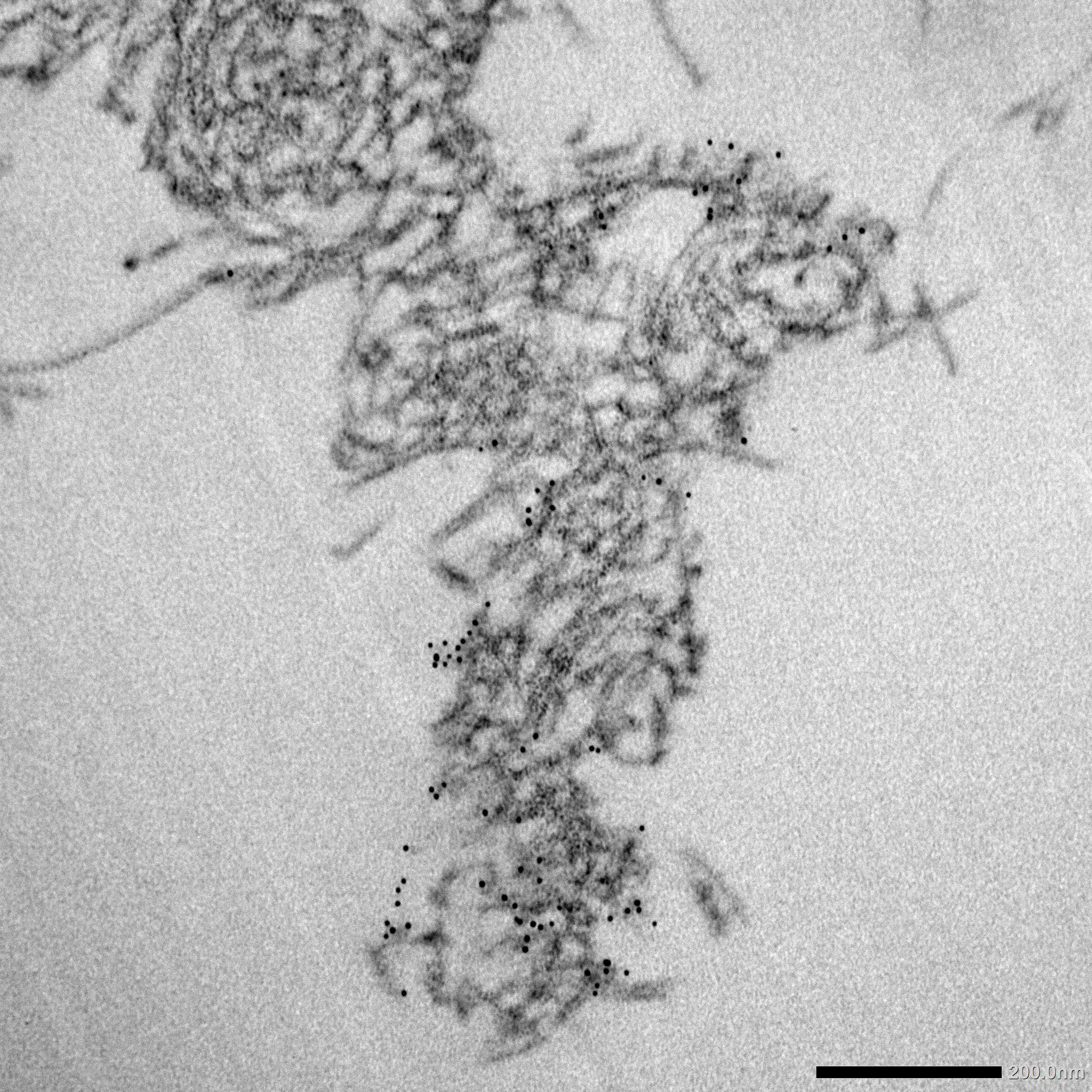

Supplement: Supplementary file 3 — Source data Fig. 1 [file 44318_2025_591_MOESM3_ESM.zip › Figure 1/1E/10_UBQLN2+╬▒-Syn_anti-╬▒-Syn.tif]

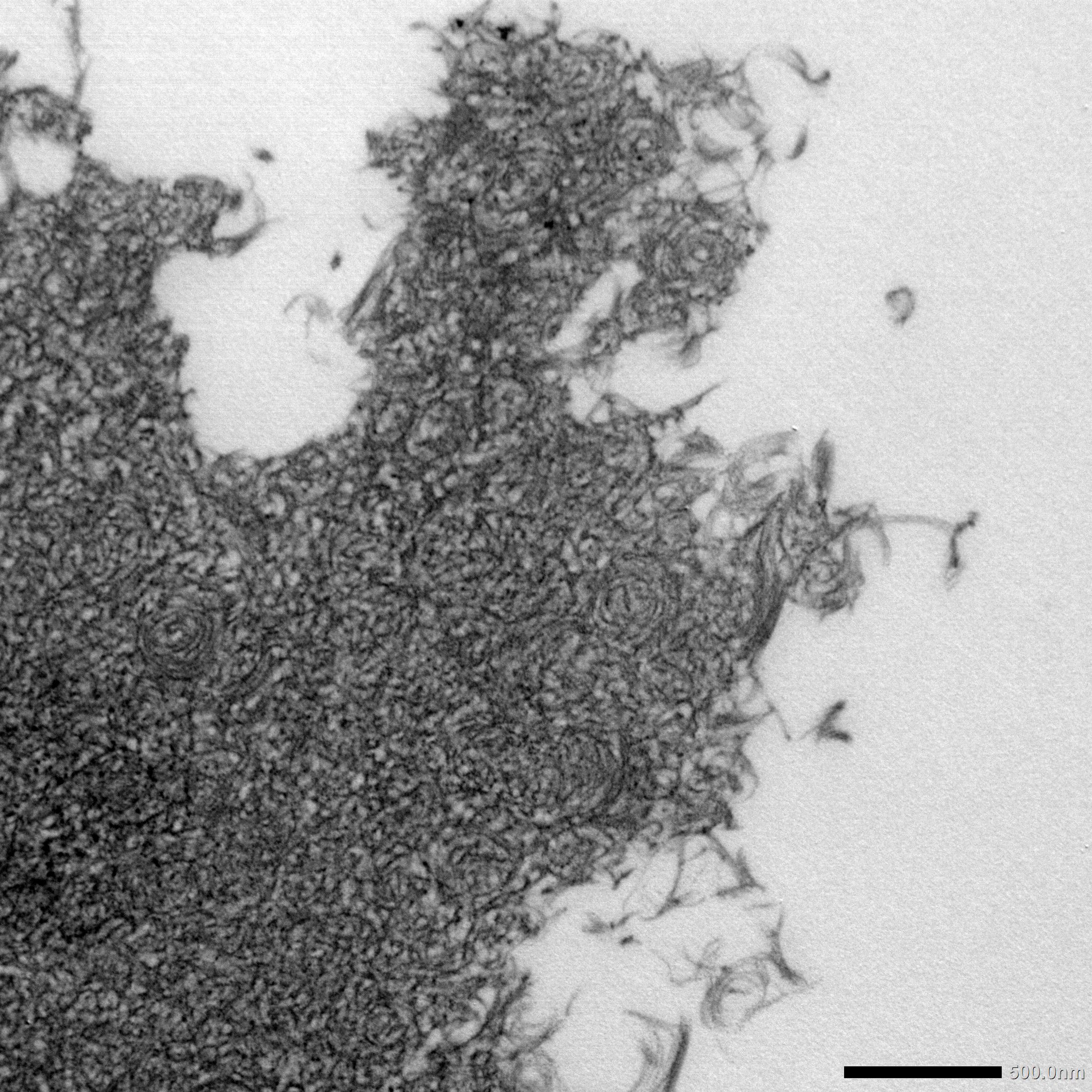

Supplement: Supplementary file 3 — Source data Fig. 1 [file 44318_2025_591_MOESM3_ESM.zip › Figure 1/1E/05_72h_01.tif]

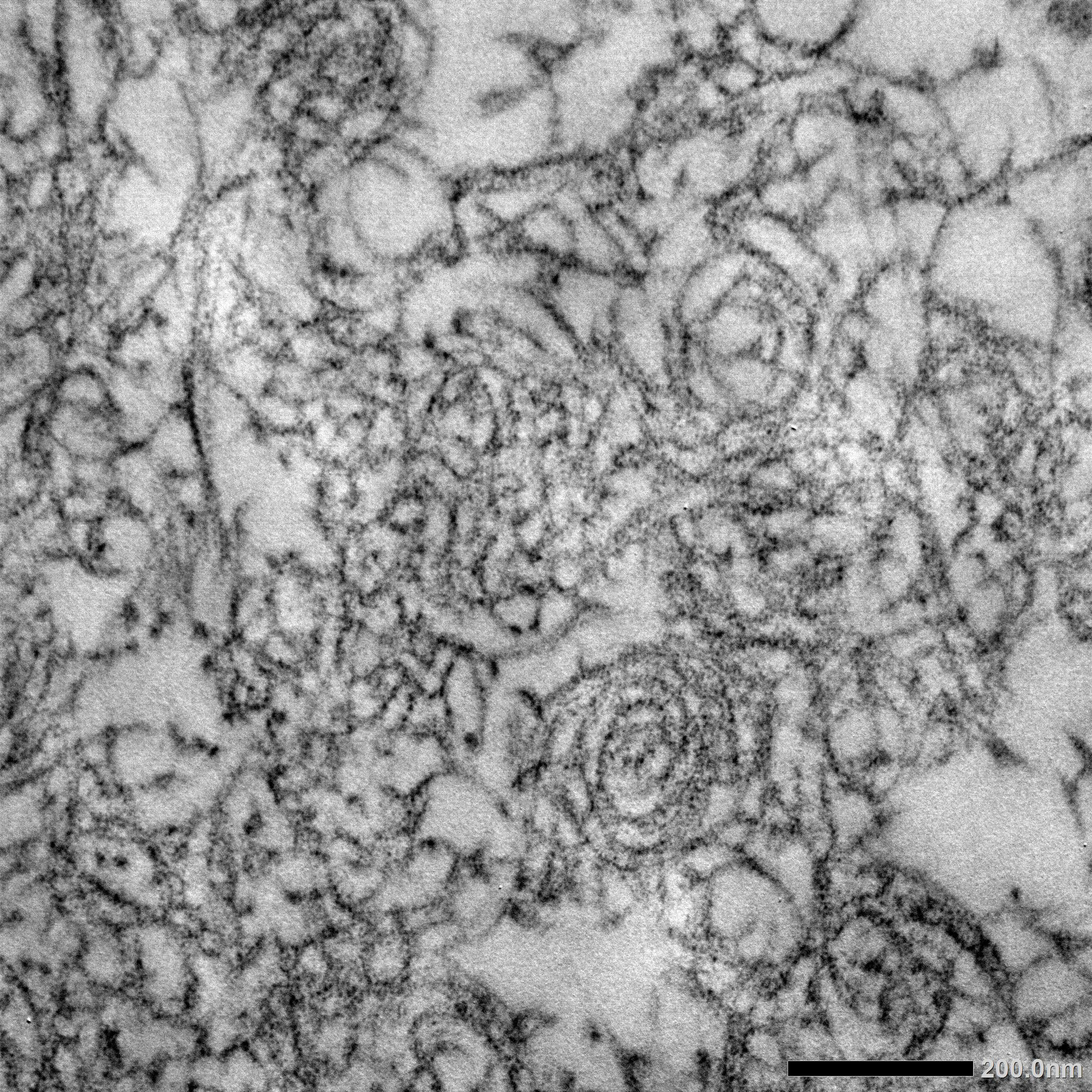

Supplement: Supplementary file 3 — Source data Fig. 1 [file 44318_2025_591_MOESM3_ESM.zip › Figure 1/1E/08_96h_02.tif]

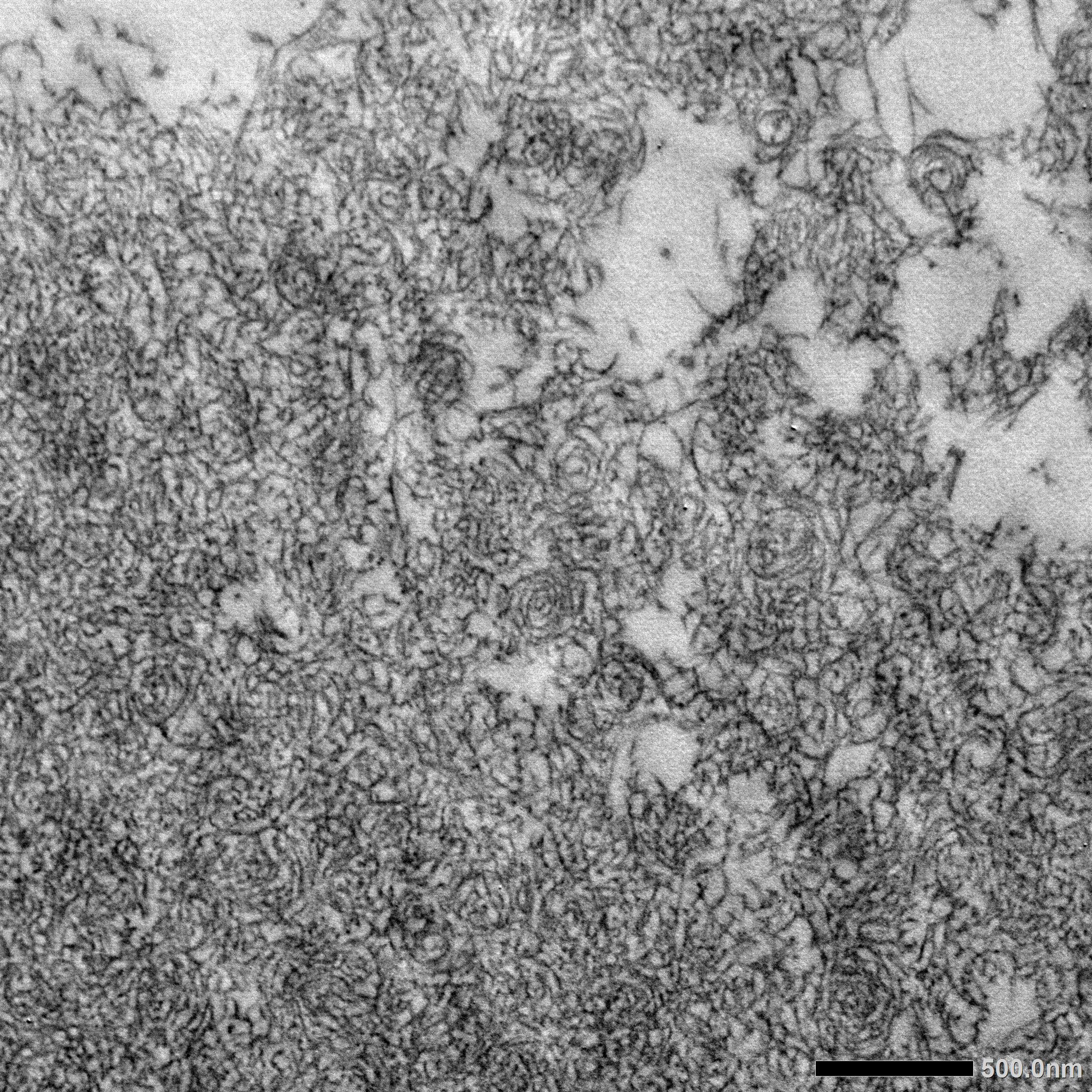

Supplement: Supplementary file 3 — Source data Fig. 1 [file 44318_2025_591_MOESM3_ESM.zip › Figure 1/1E/07_96h_01.tif]

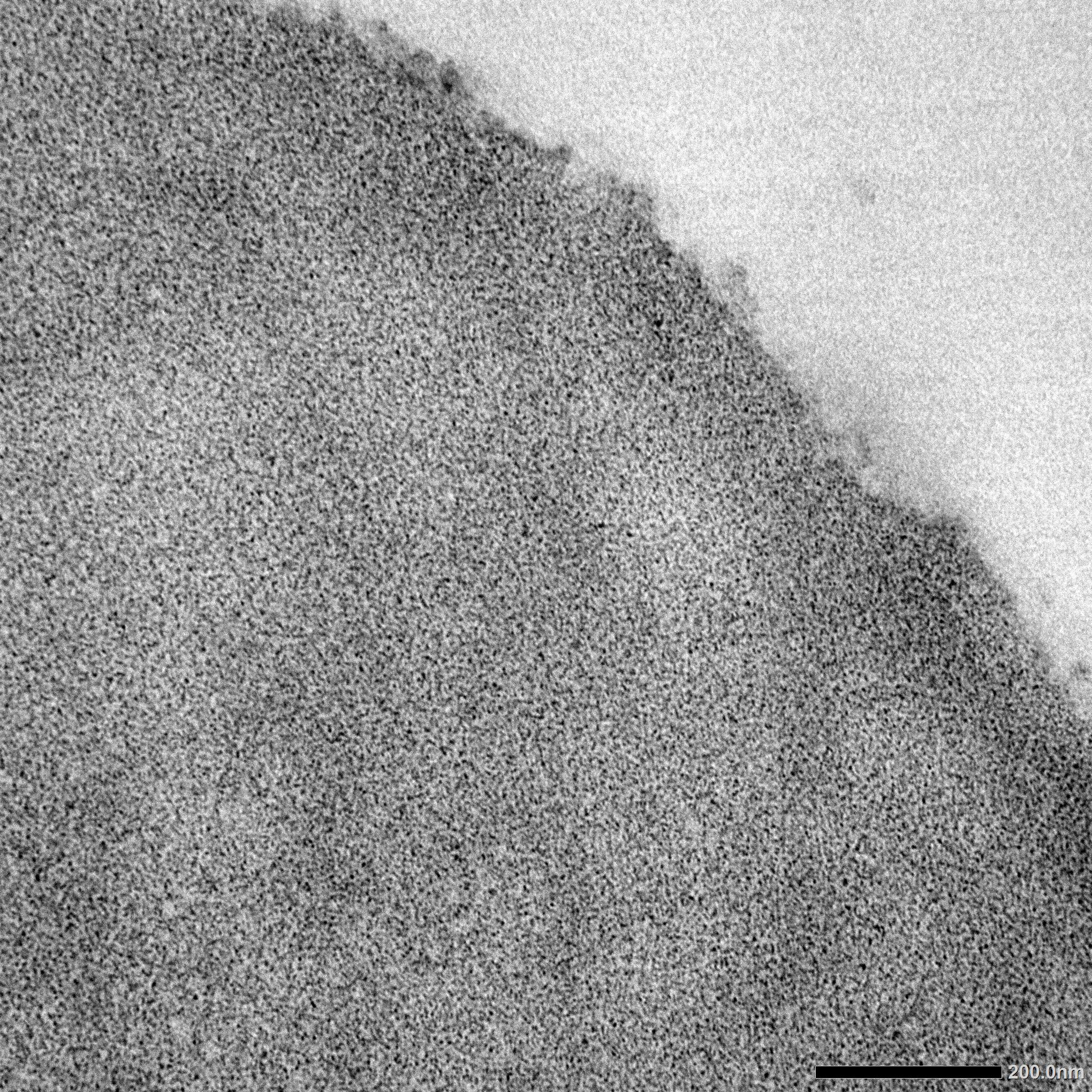

Supplement: Supplementary file 3 — Source data Fig. 1 [file 44318_2025_591_MOESM3_ESM.zip › Figure 1/1E/02_48h(#1)_02.tif]

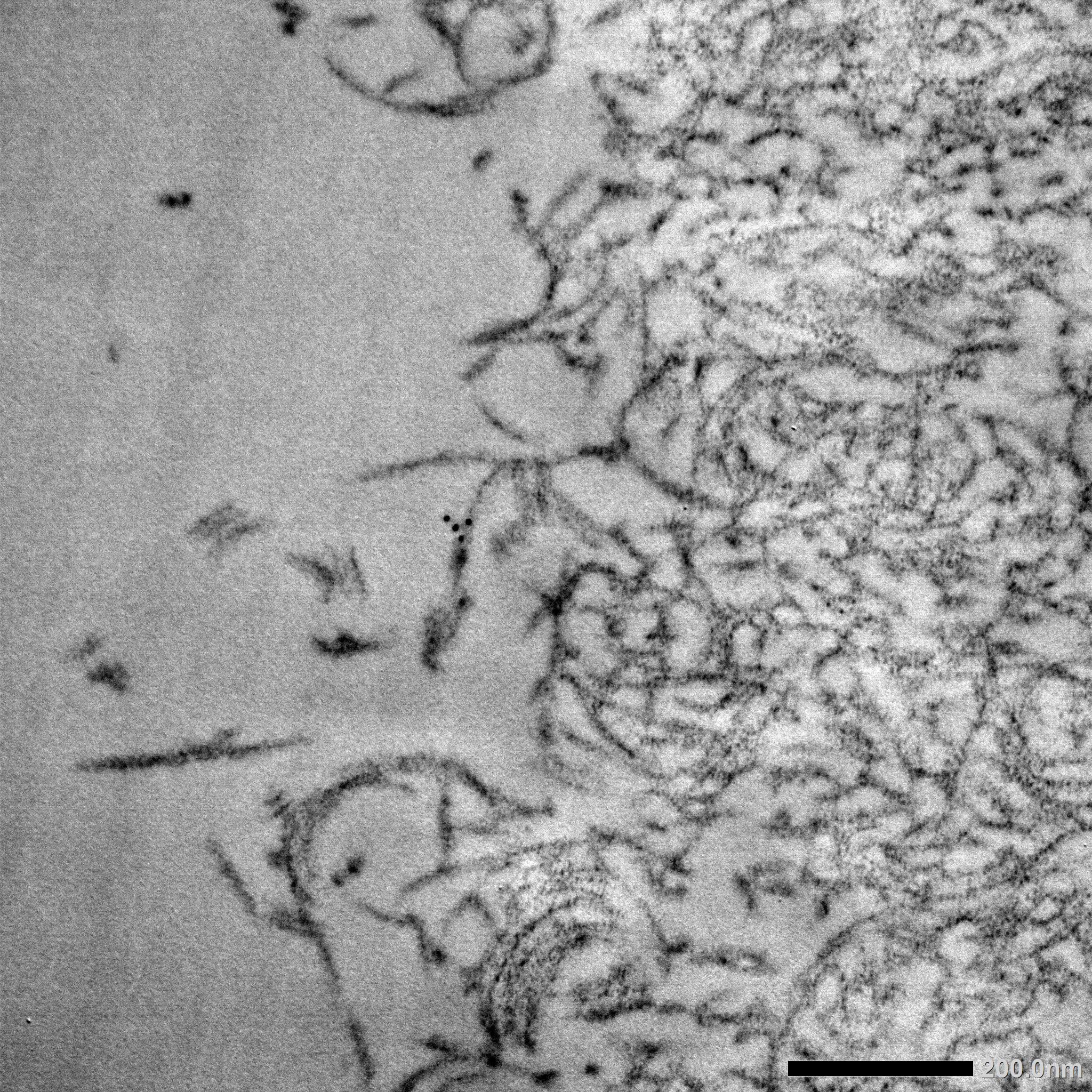

Supplement: Supplementary file 3 — Source data Fig. 1 [file 44318_2025_591_MOESM3_ESM.zip › Figure 1/1E/09_UBQLN2_anti-╬▒-Syn.tif]

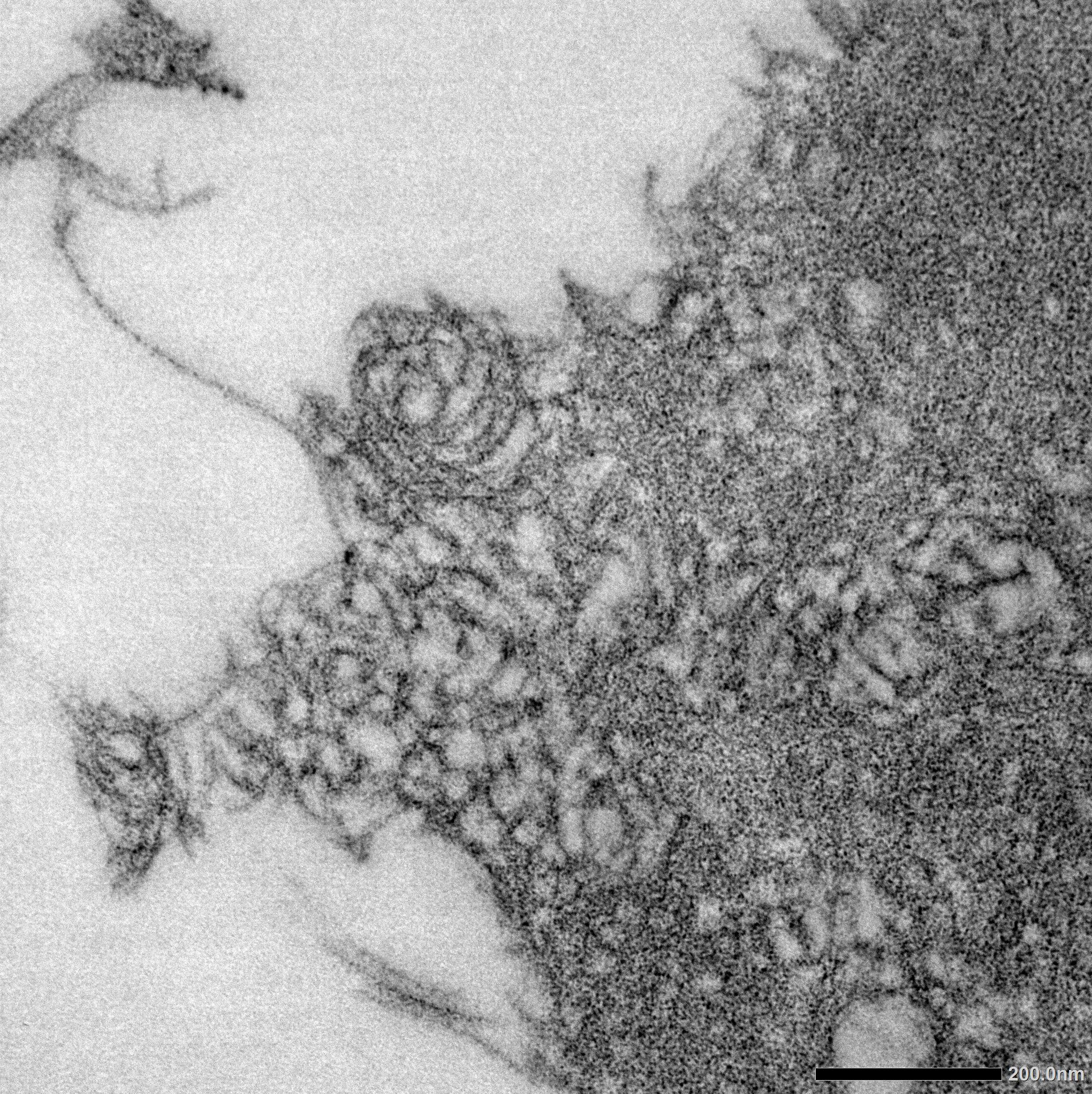

Supplement: Supplementary file 3 — Source data Fig. 1 [file 44318_2025_591_MOESM3_ESM.zip › Figure 1/1E/04_48h(#1)_02.tif]

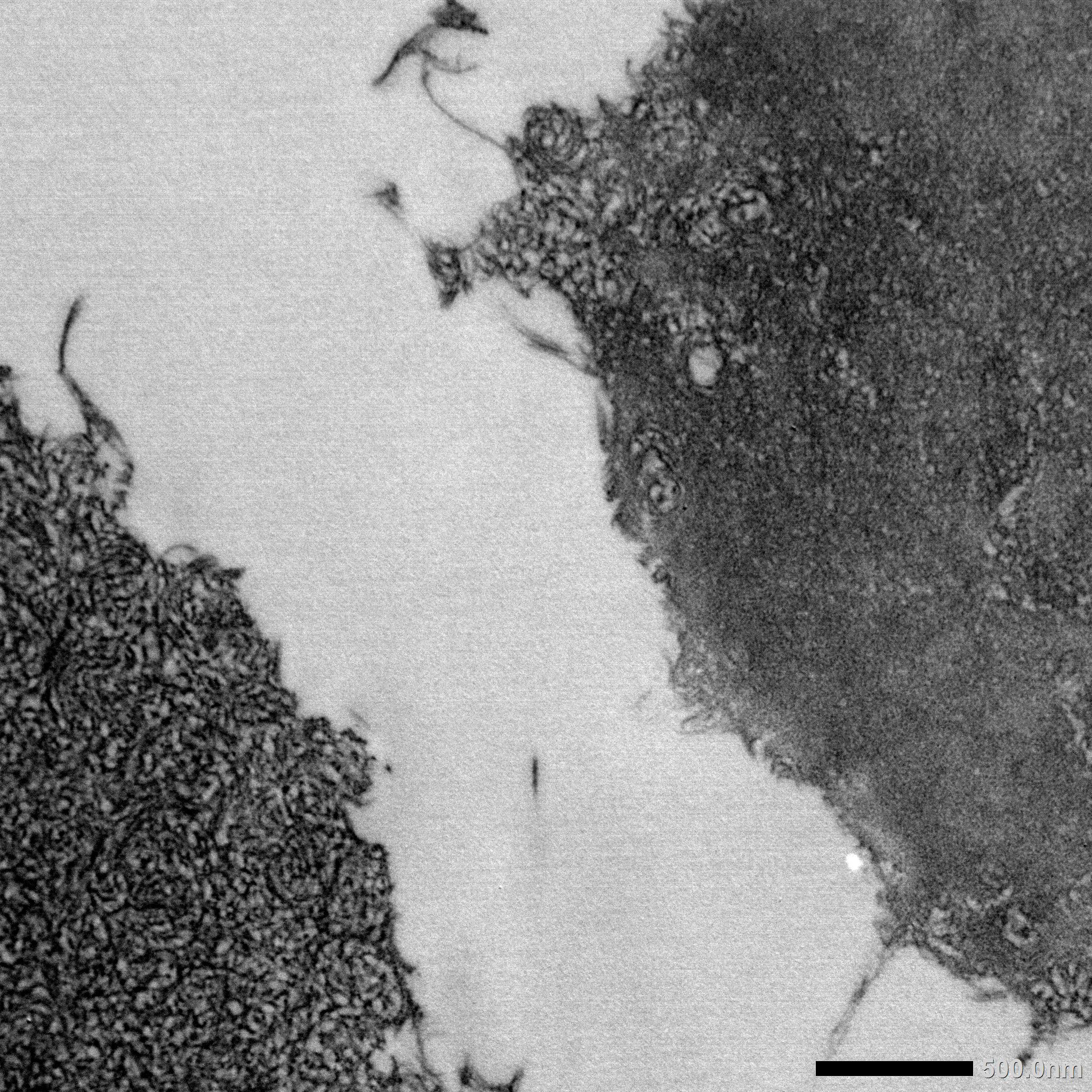

Supplement: Supplementary file 3 — Source data Fig. 1 [file 44318_2025_591_MOESM3_ESM.zip › Figure 1/1E/03_48h(#1)_01.tif]

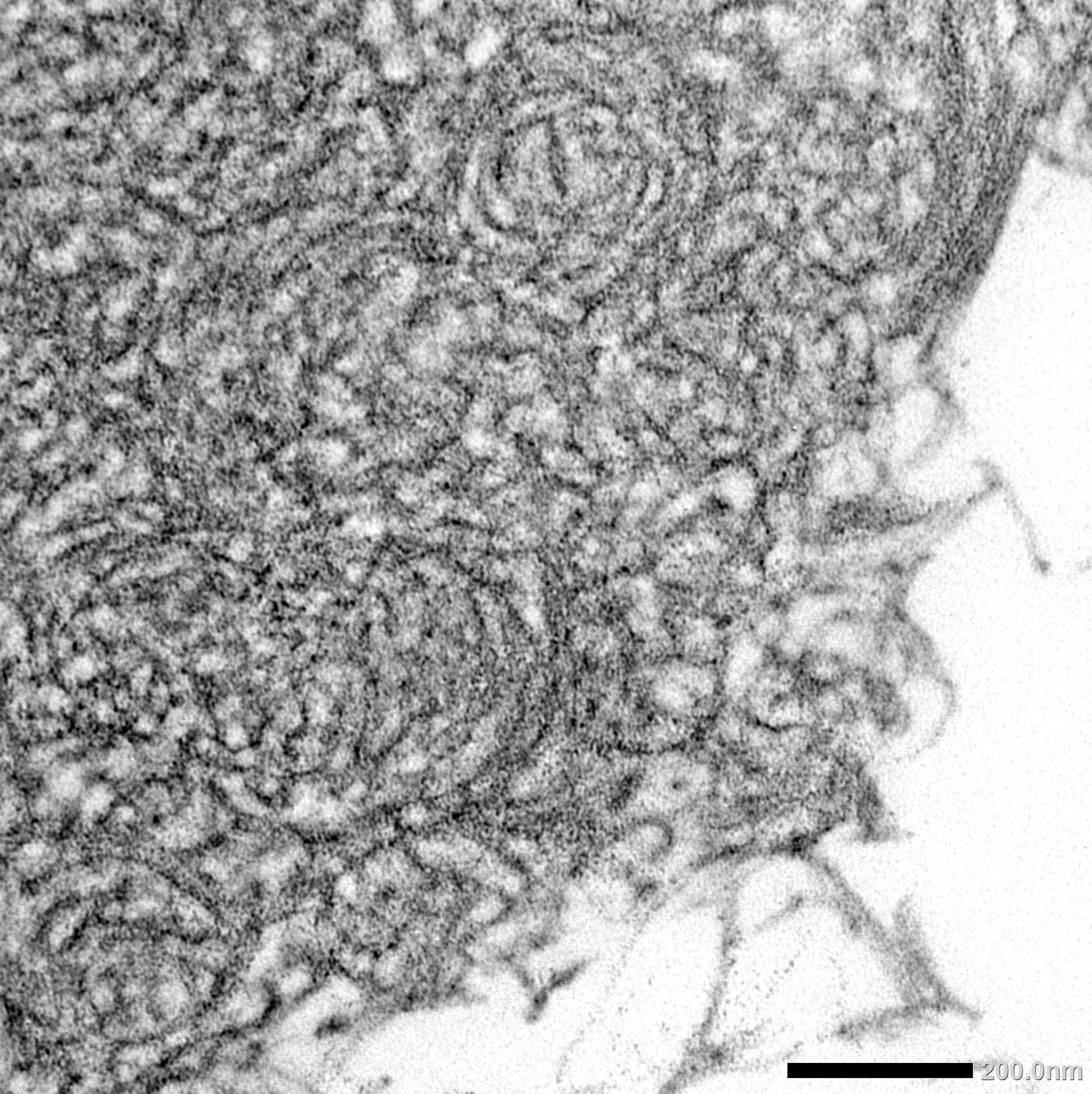

Supplement: Supplementary file 3 — Source data Fig. 1 [file 44318_2025_591_MOESM3_ESM.zip › Figure 1/1E/06_72h_02.tif]

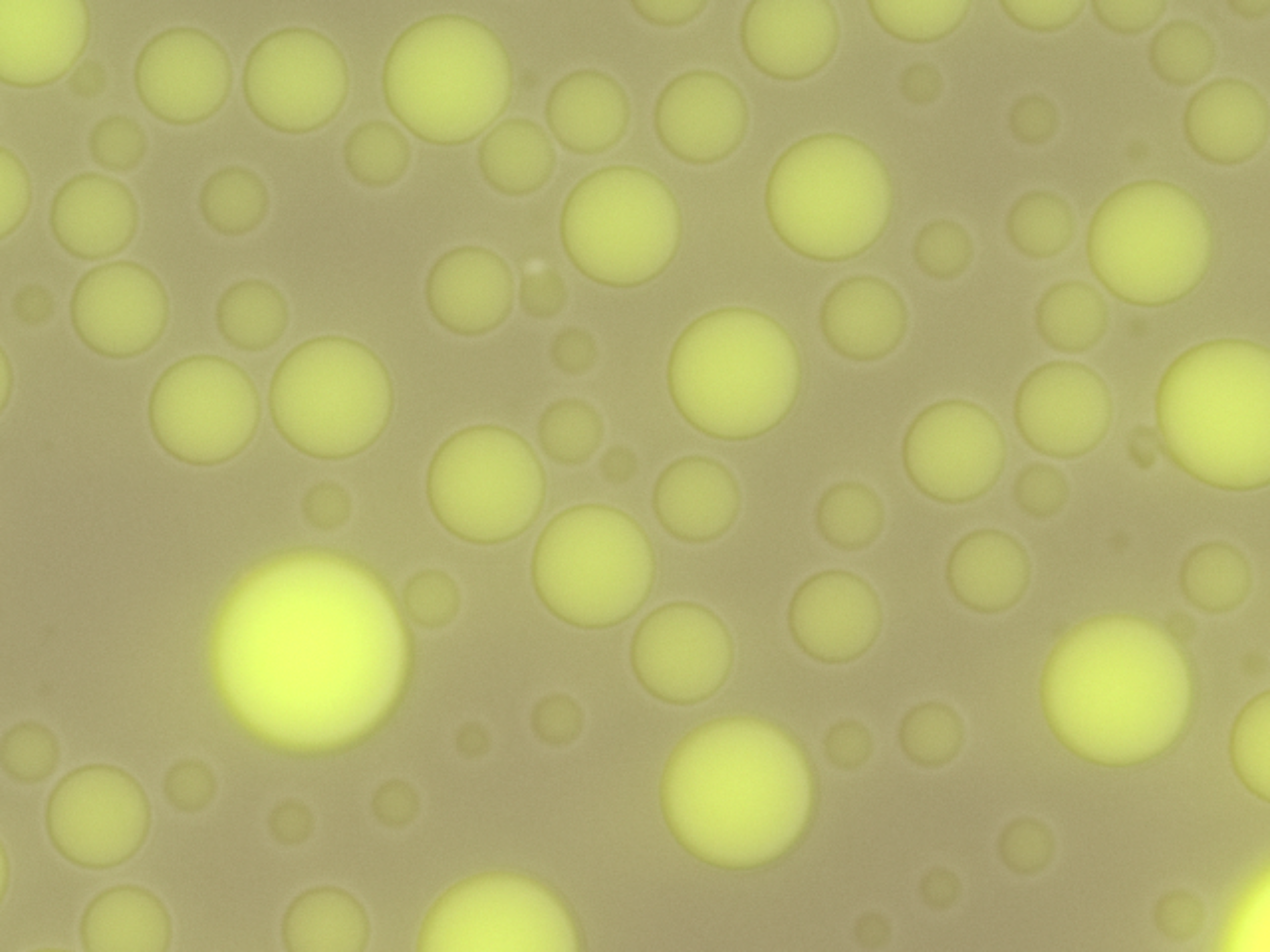

Supplement: Supplementary file 3 — Source data Fig. 1 [file 44318_2025_591_MOESM3_ESM.zip › Figure 1/1D/04_24 h_Merge.tif]

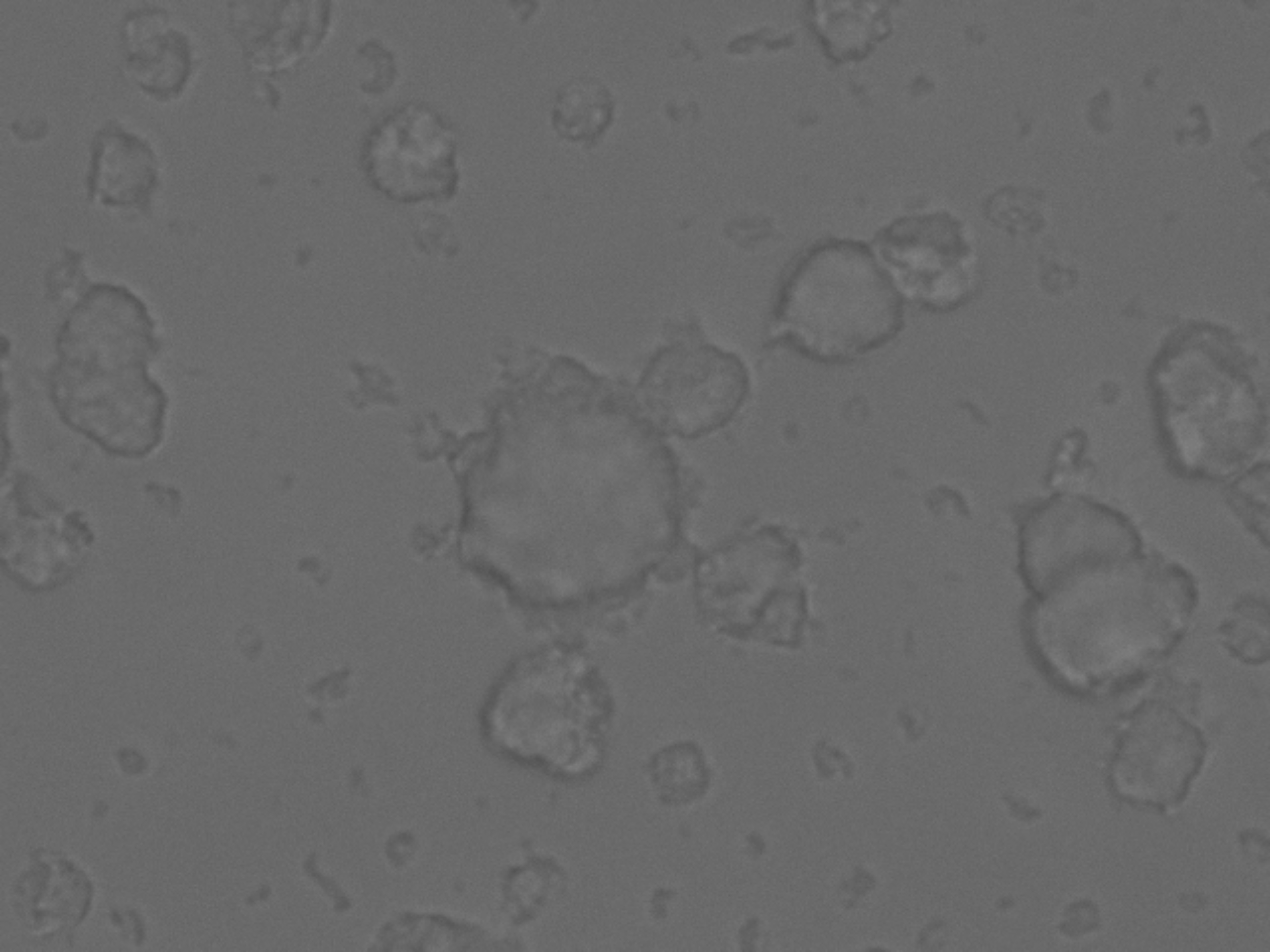

Supplement: Supplementary file 3 — Source data Fig. 1 [file 44318_2025_591_MOESM3_ESM.zip › Figure 1/1D/07_96 h_Bright field.tif]

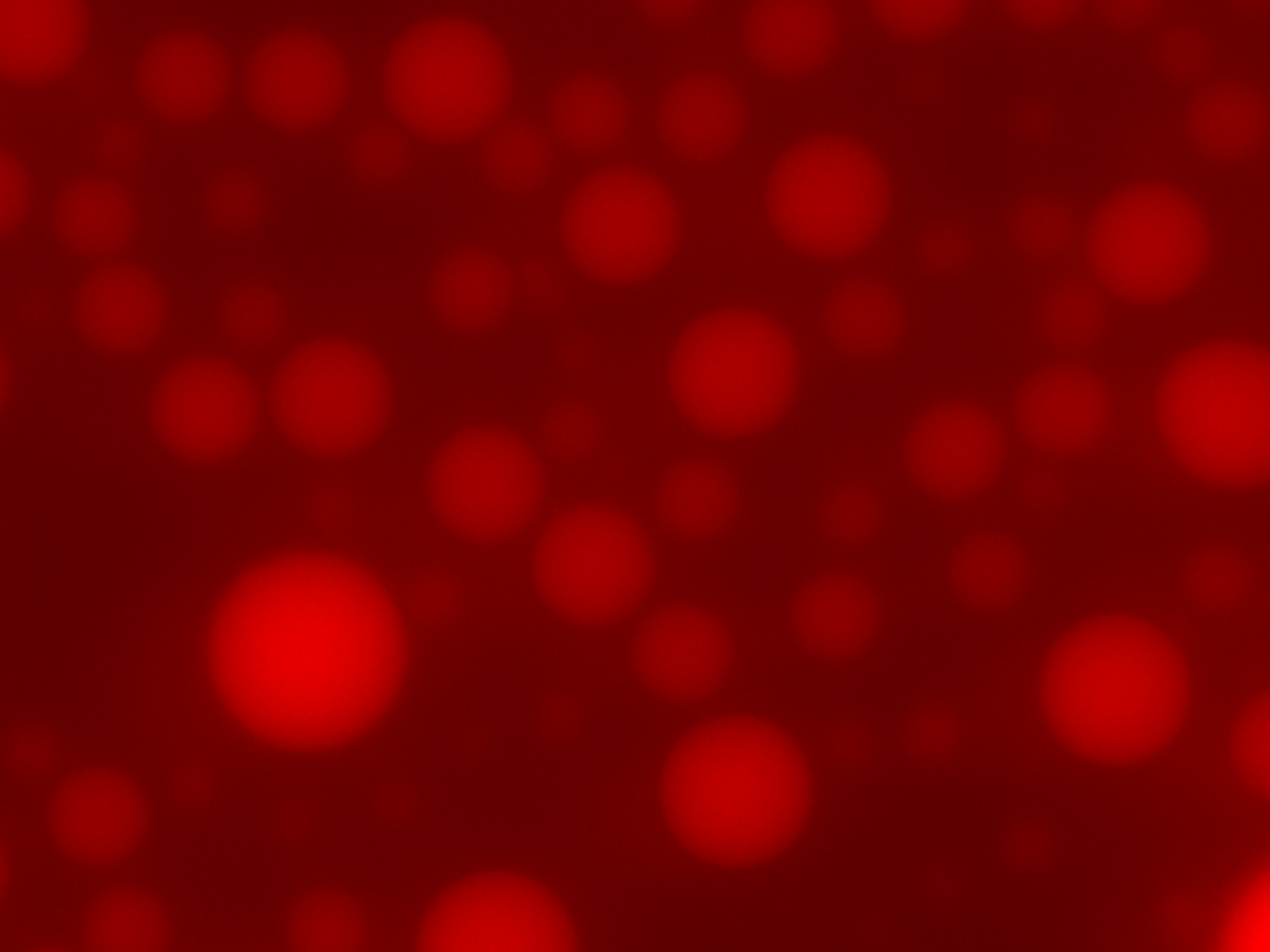

Supplement: Supplementary file 3 — Source data Fig. 1 [file 44318_2025_591_MOESM3_ESM.zip › Figure 1/1D/02_24 h_╬▒-Syn.tif]

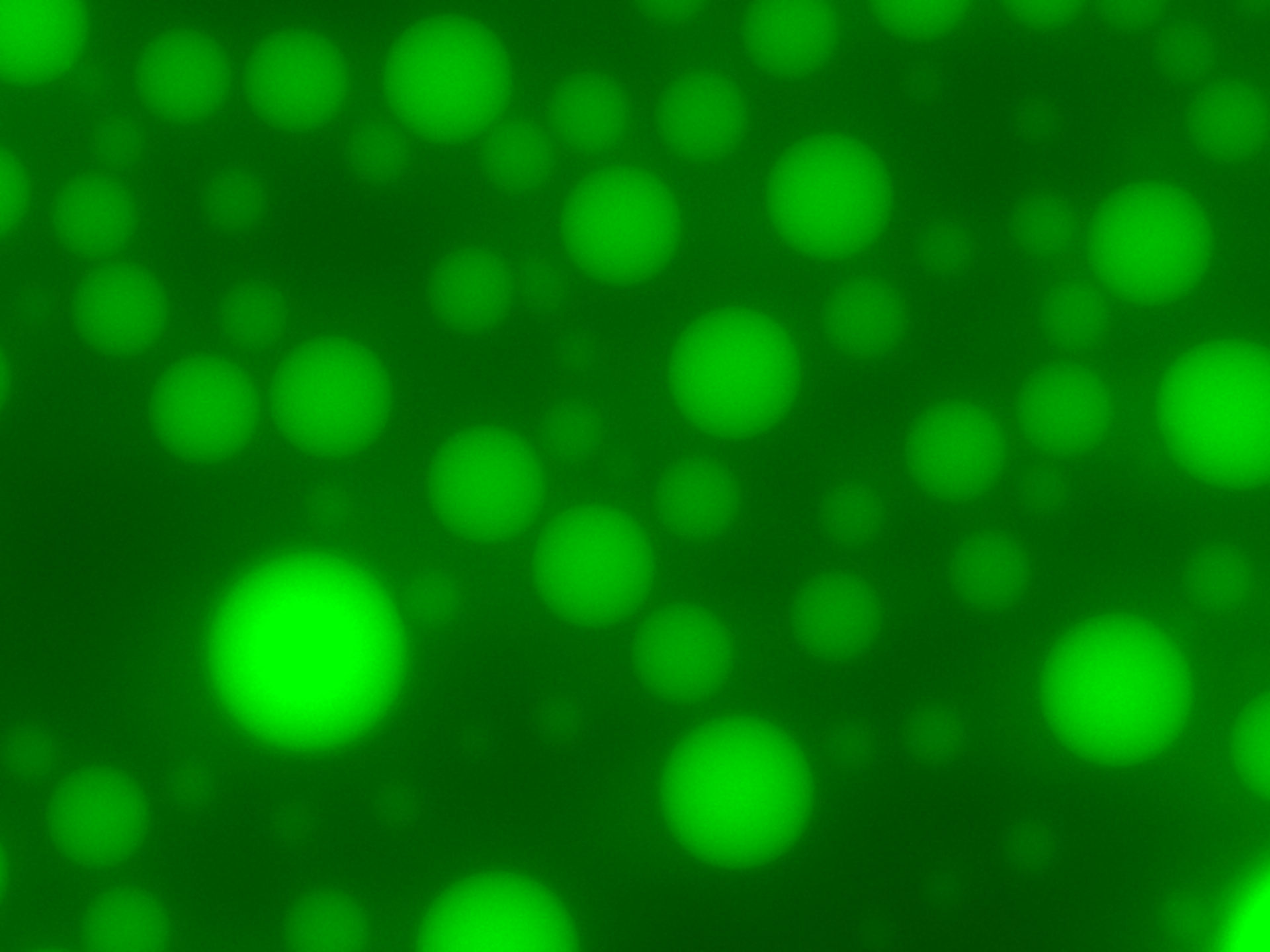

Supplement: Supplementary file 3 — Source data Fig. 1 [file 44318_2025_591_MOESM3_ESM.zip › Figure 1/1D/01_24 h_UBQLN2.tif]

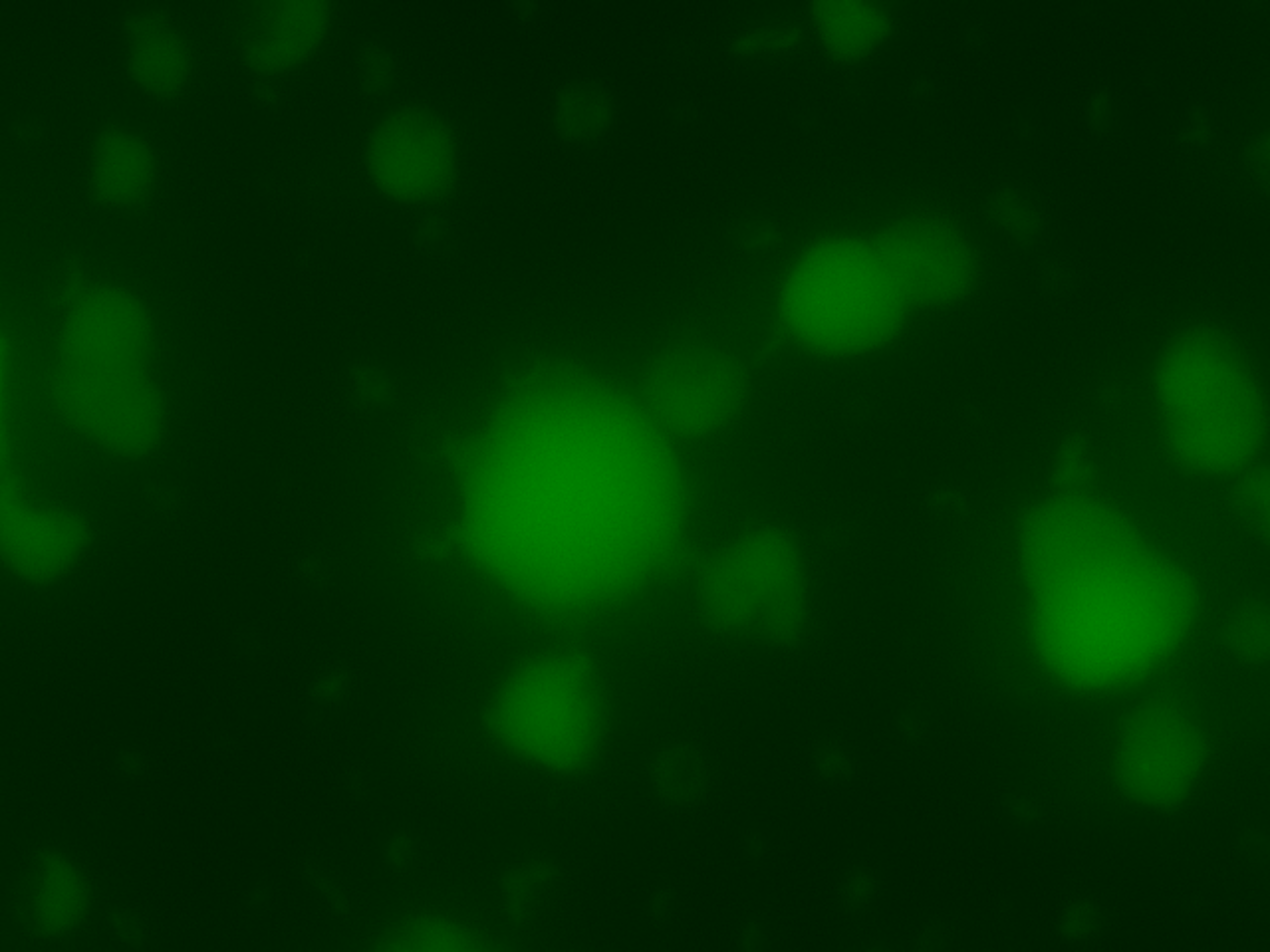

Supplement: Supplementary file 3 — Source data Fig. 1 [file 44318_2025_591_MOESM3_ESM.zip › Figure 1/1D/05_96 h_UBQLN2.tif]

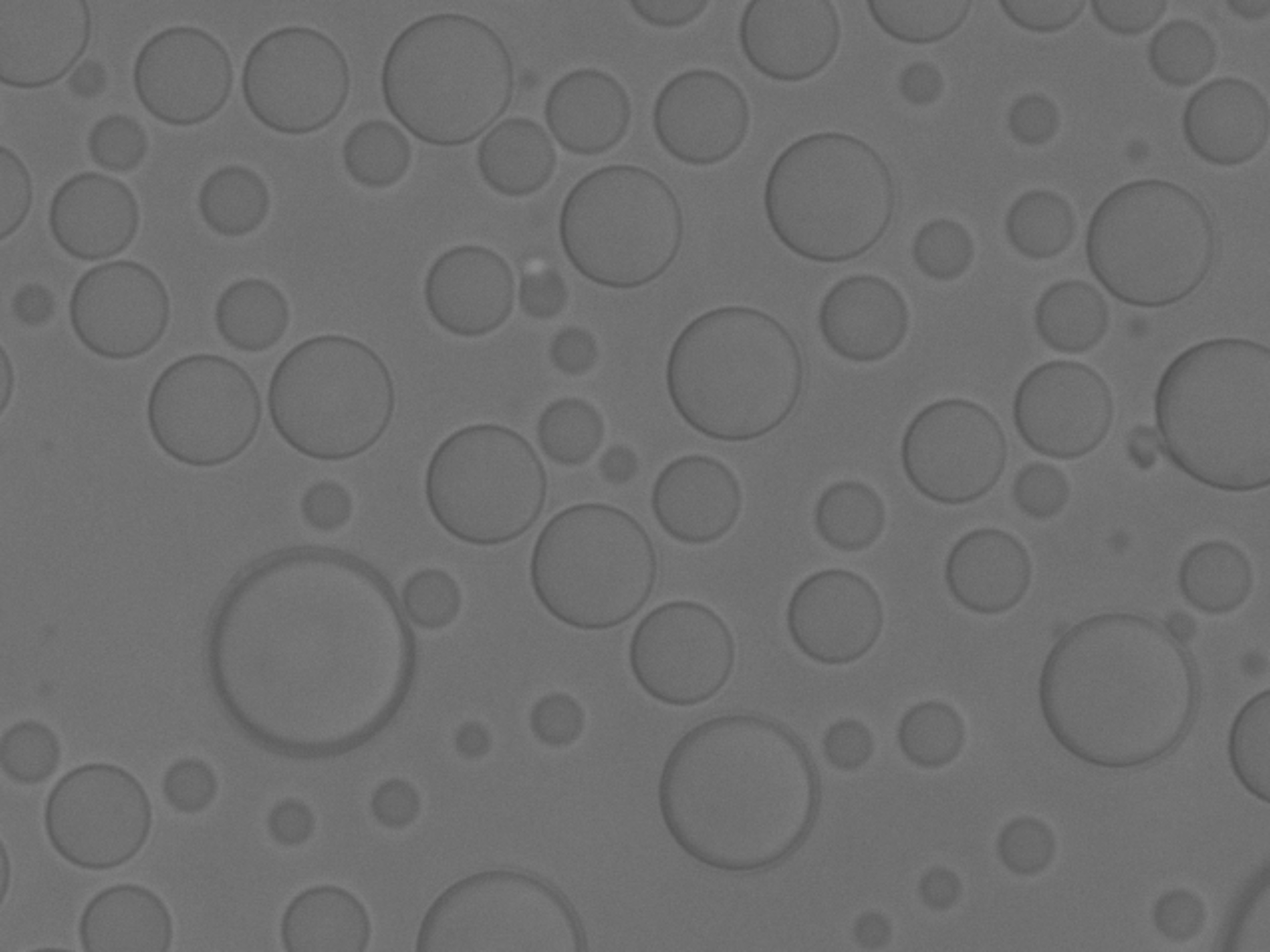

Supplement: Supplementary file 3 — Source data Fig. 1 [file 44318_2025_591_MOESM3_ESM.zip › Figure 1/1D/03_24 h_Bright field.tif]

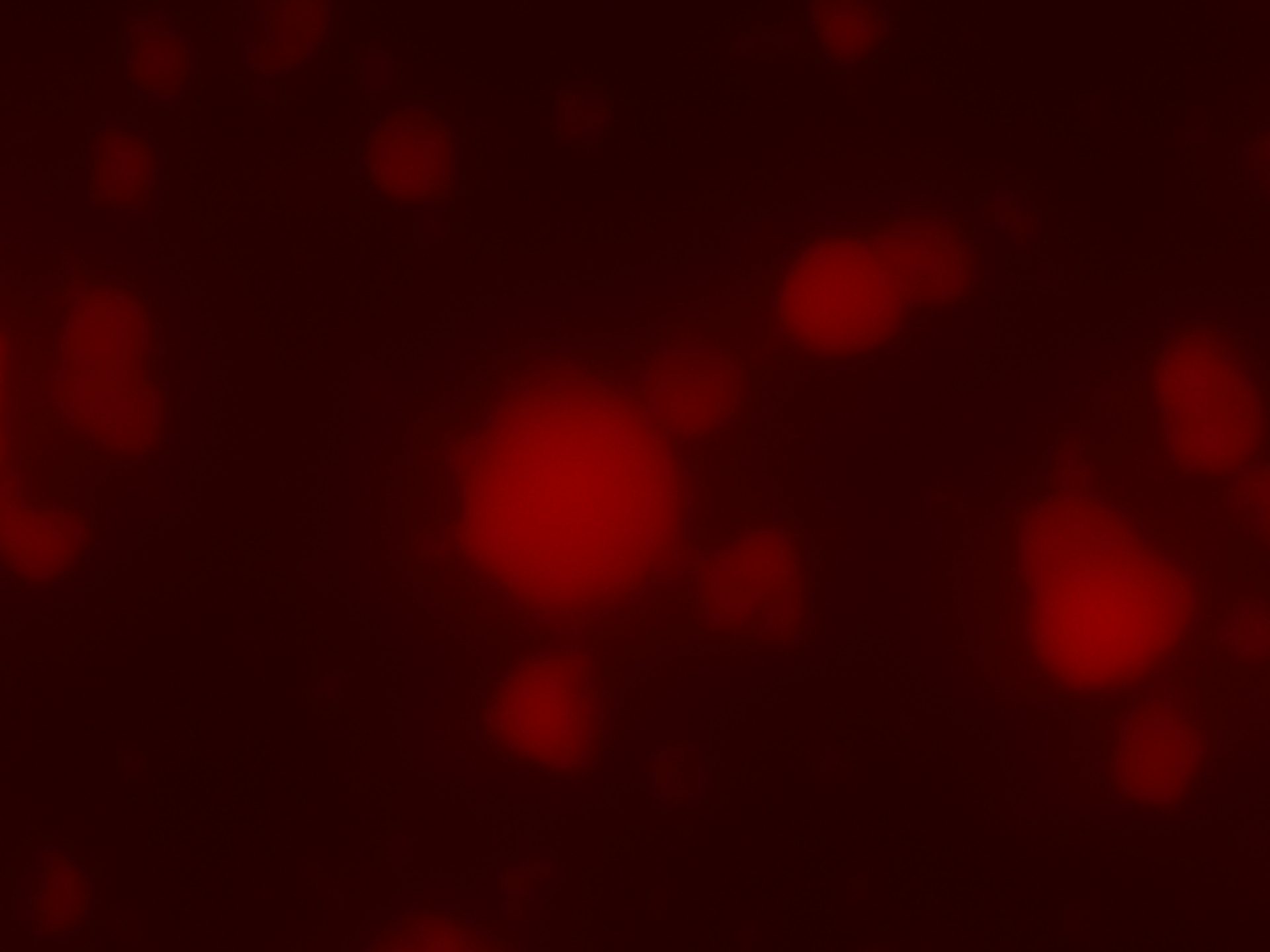

Supplement: Supplementary file 3 — Source data Fig. 1 [file 44318_2025_591_MOESM3_ESM.zip › Figure 1/1D/06_96 h_╬▒-Syn.tif]

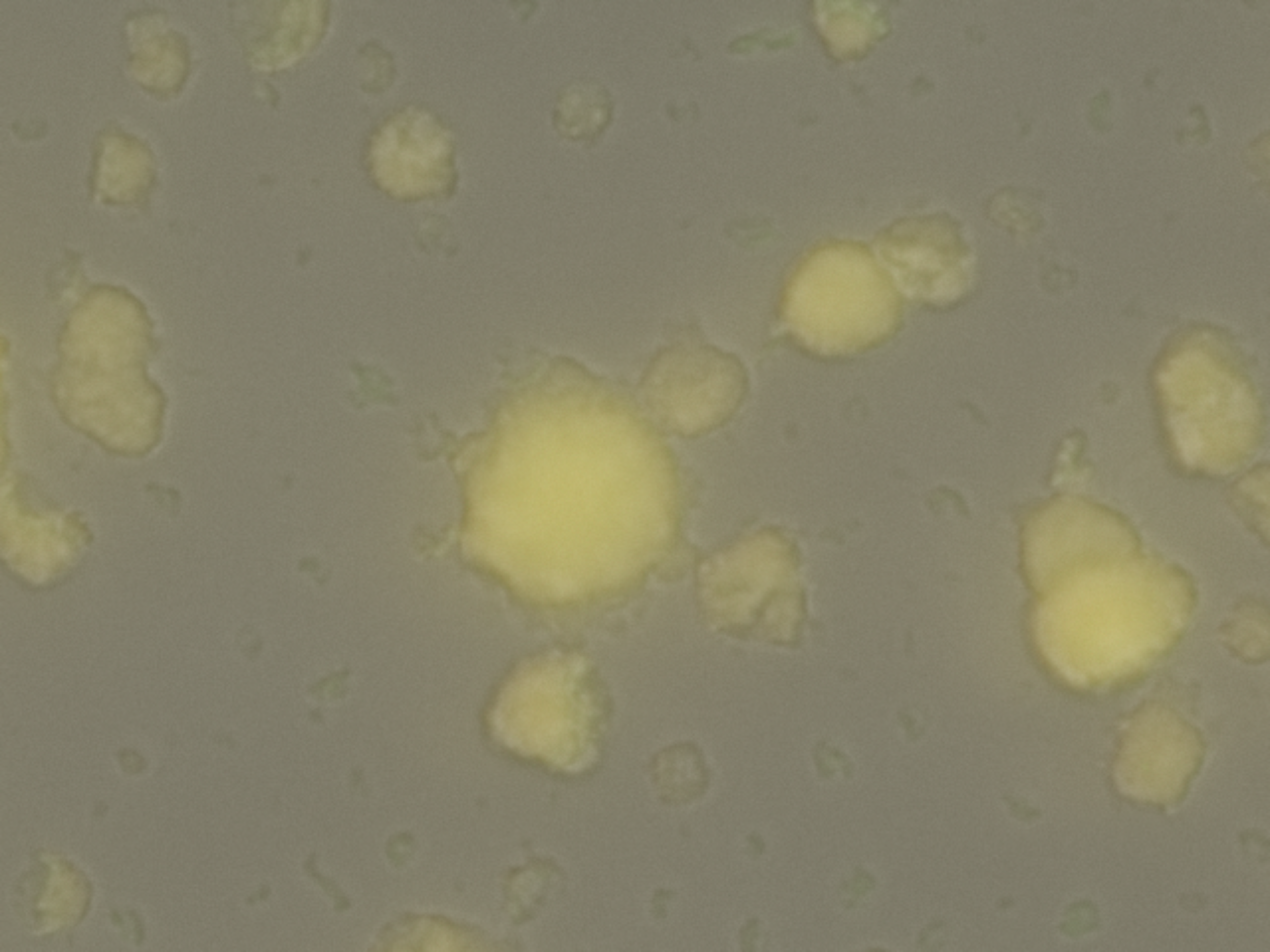

Supplement: Supplementary file 3 — Source data Fig. 1 [file 44318_2025_591_MOESM3_ESM.zip › Figure 1/1D/08_96 h_Merge.tif]

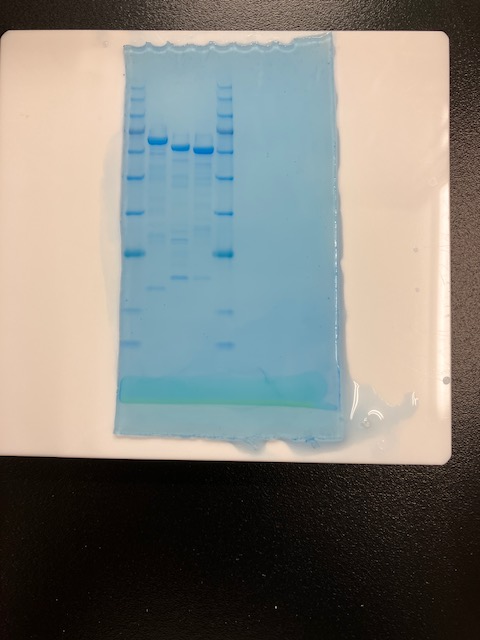

Supplement: Supplementary file 4 — Source data Fig. 2 [file 44318_2025_591_MOESM4_ESM.zip › Figure 2/2A/(b)_CBB.tif]

Source Data of Fig. 2B

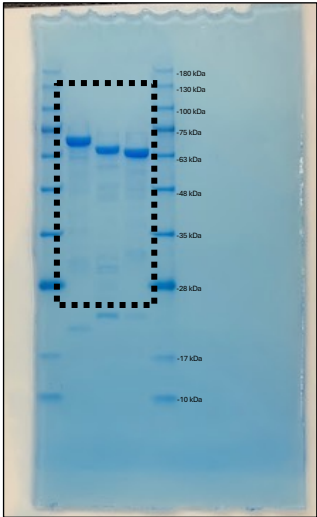

CBB staining

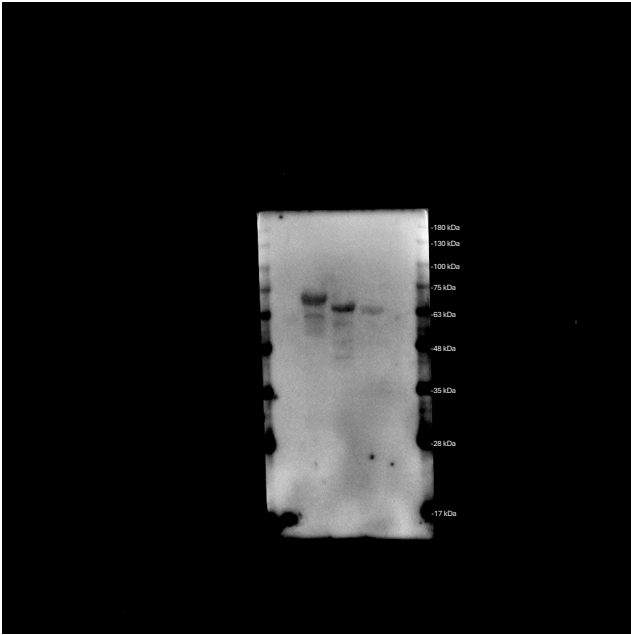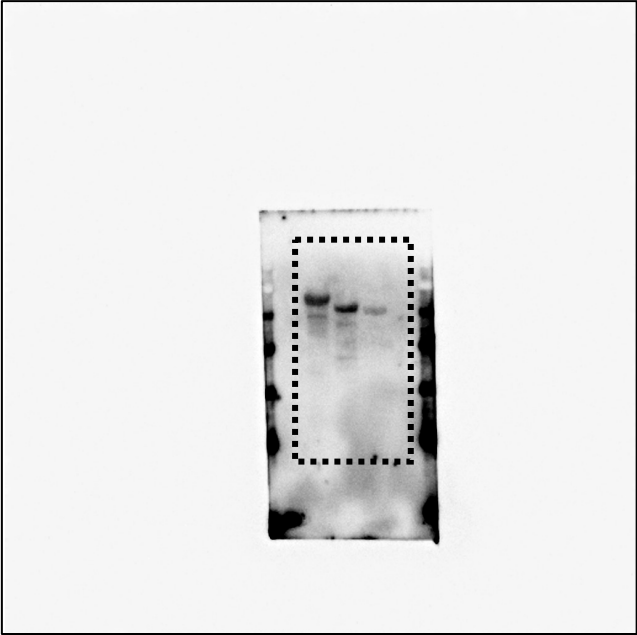

anti- $\alpha$ -Syn

Supplement: Supplementary file 4 — Source data Fig. 2 [file 44318_2025_591_MOESM4_ESM.zip › Figure 2/2A/Highight of crop area.pdf]

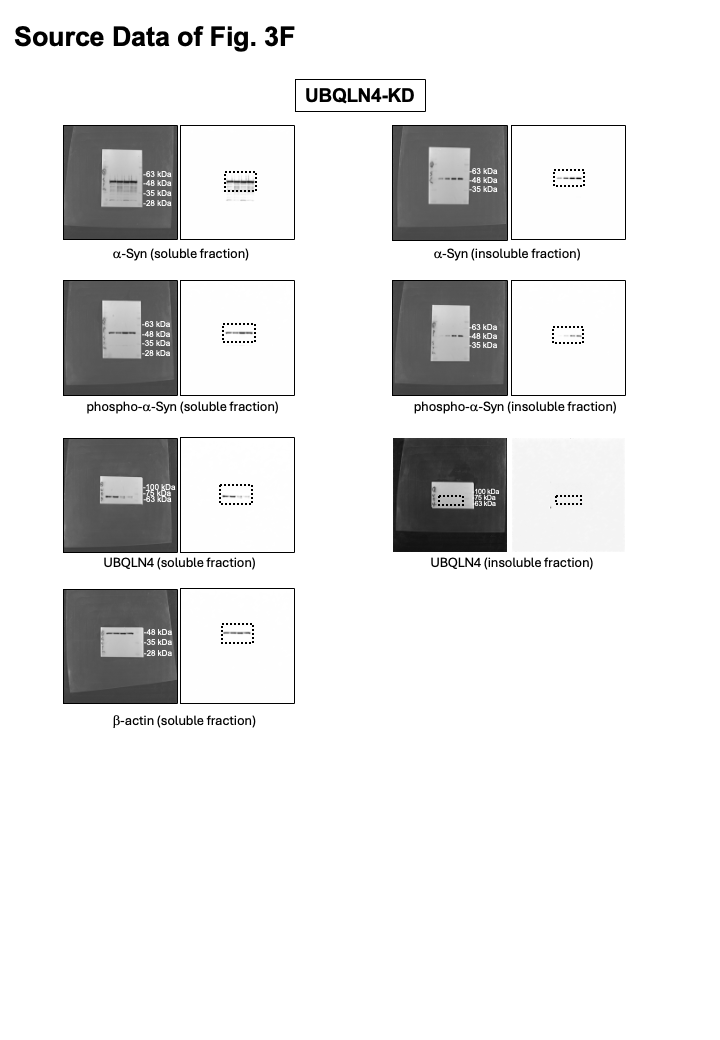

Supplement: Supplementary file 5 — Source data Fig. 3 [file 44318_2025_591_MOESM5_ESM.zip › Figure 3/3F/24_Highlight for crop area_UBQLN4_KD.tiff]

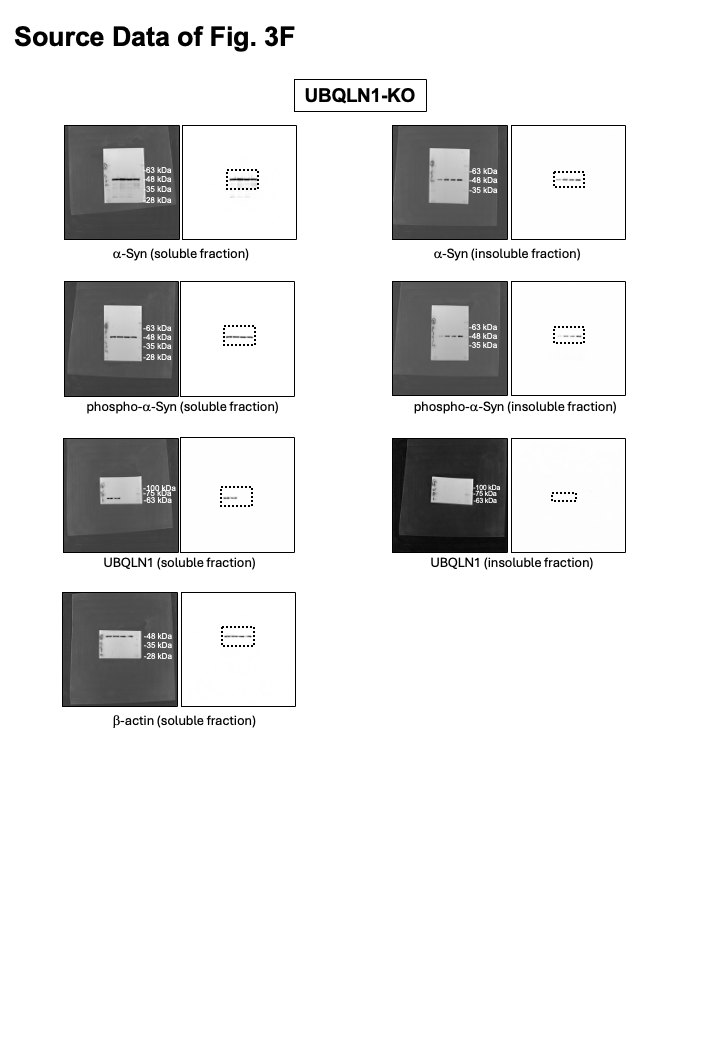

Supplement: Supplementary file 5 — Source data Fig. 3 [file 44318_2025_591_MOESM5_ESM.zip › Figure 3/3F/23_Highlight for crop area_UBQLN1_KO.tiff]

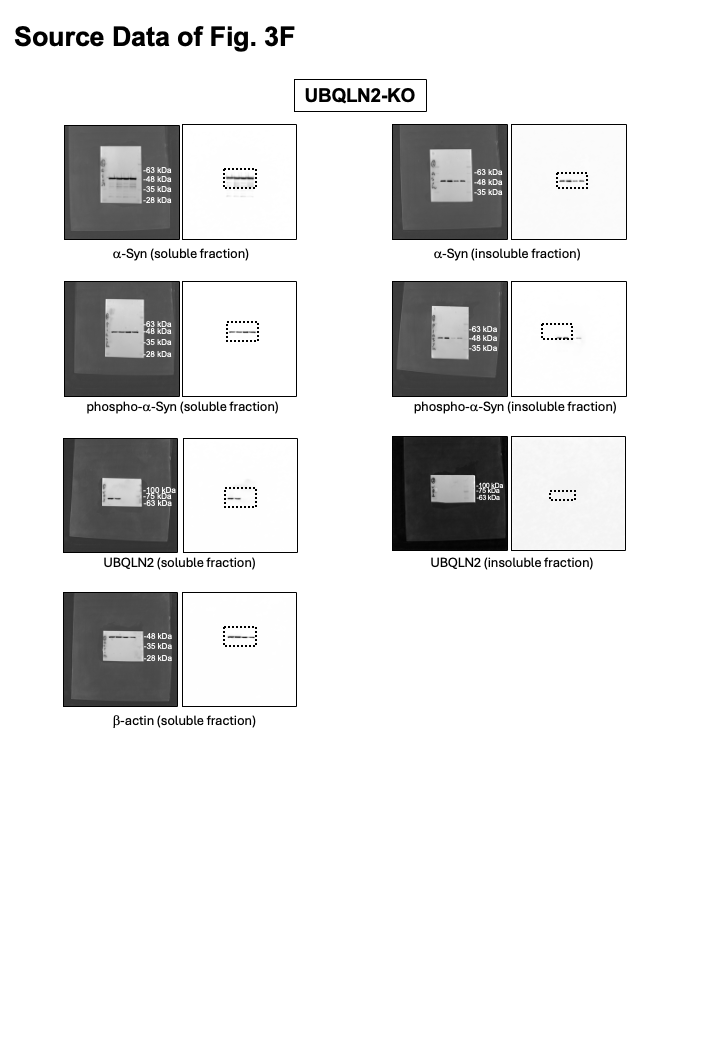

Supplement: Supplementary file 5 — Source data Fig. 3 [file 44318_2025_591_MOESM5_ESM.zip › Figure 3/3F/22_Highlight for crop area_UBQLN2_KO.tiff]

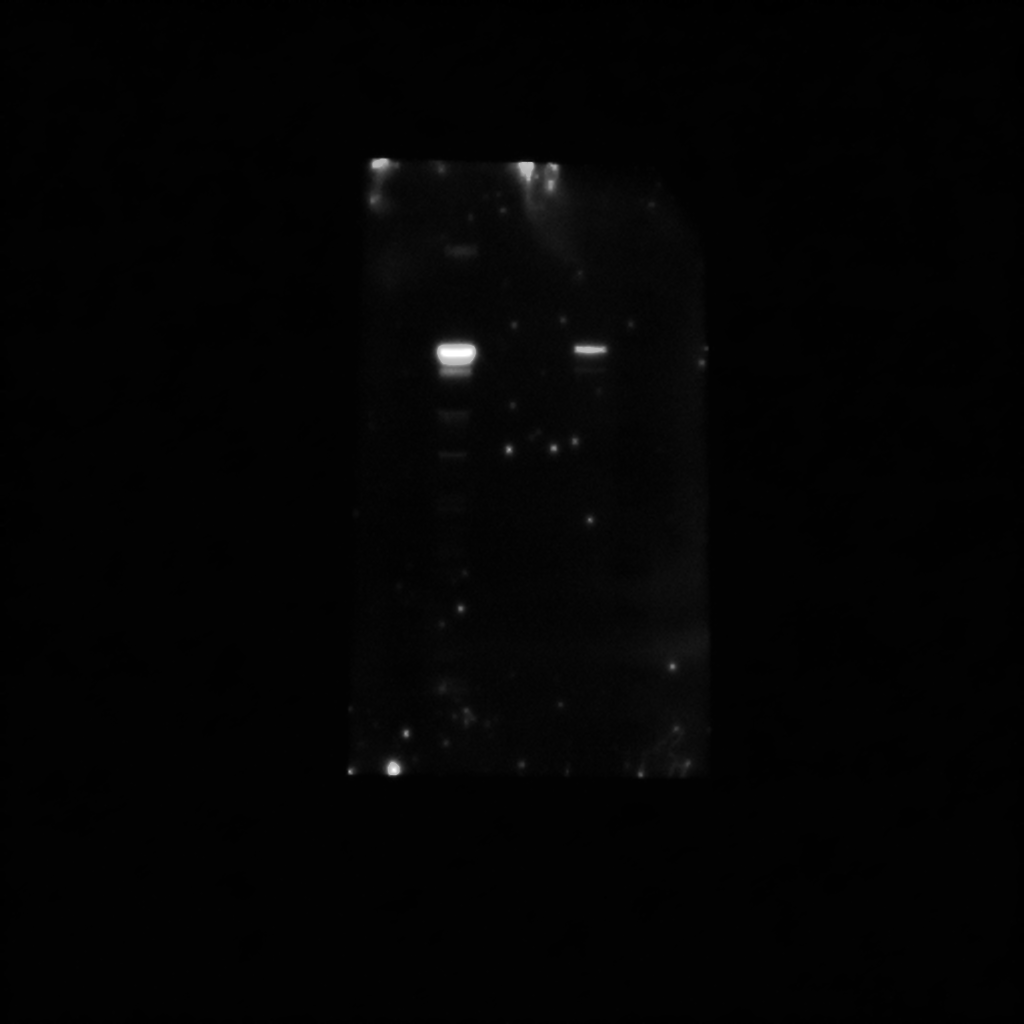

Supplement: Supplementary file 6 — Source data Fig. 4 [file 44318_2025_591_MOESM6_ESM.zip › Figure 4/4B/02_UBQLN2.Tif]

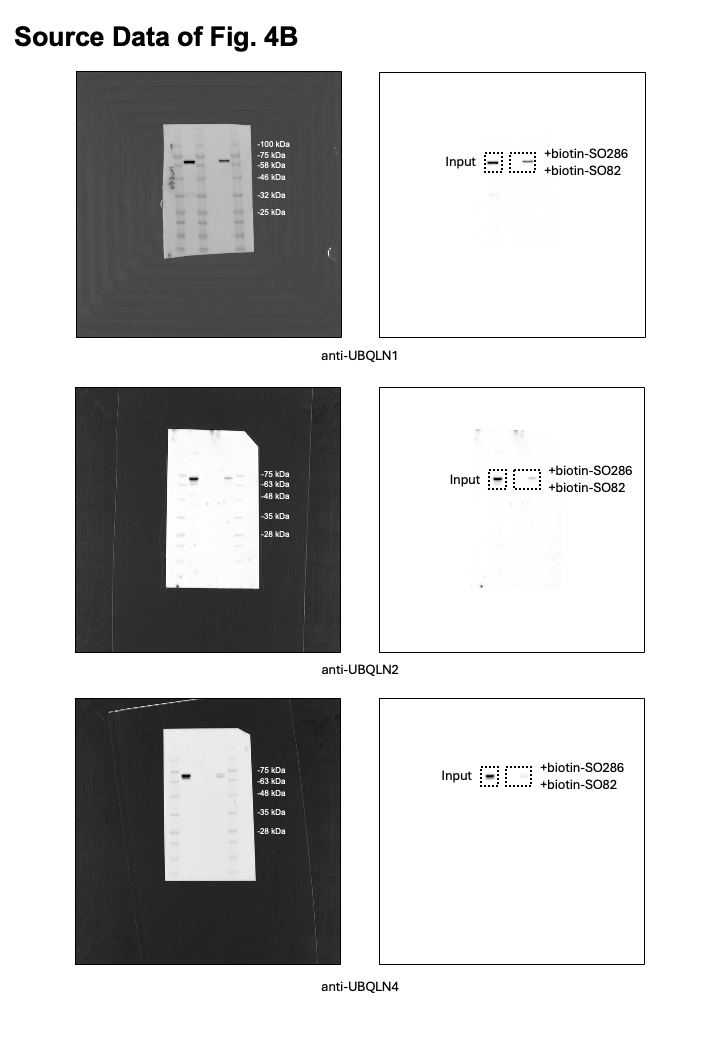

Supplement: Supplementary file 6 — Source data Fig. 4 [file 44318_2025_591_MOESM6_ESM.zip › Figure 4/4B/04_Highlight of crop area.tiff]

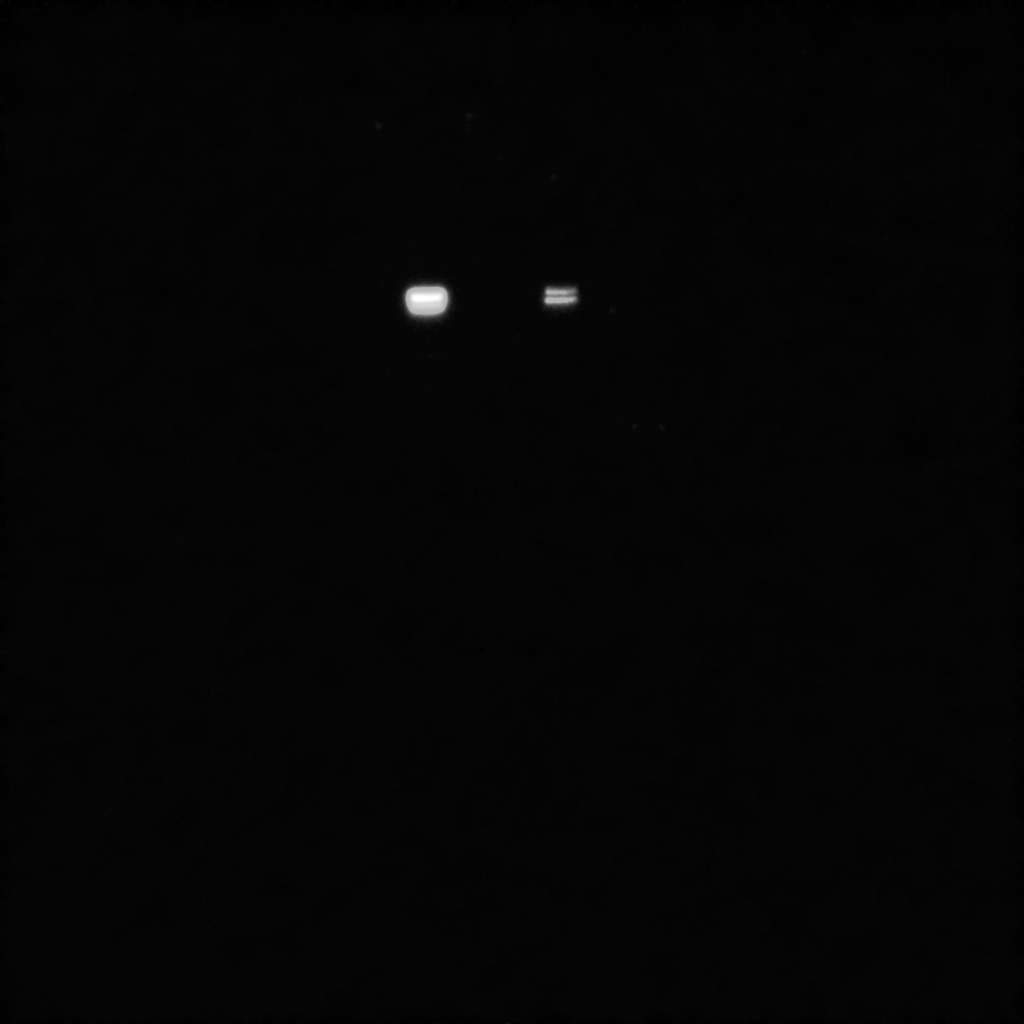

Supplement: Supplementary file 6 — Source data Fig. 4 [file 44318_2025_591_MOESM6_ESM.zip › Figure 4/4B/03_UBQLN4.Tif]

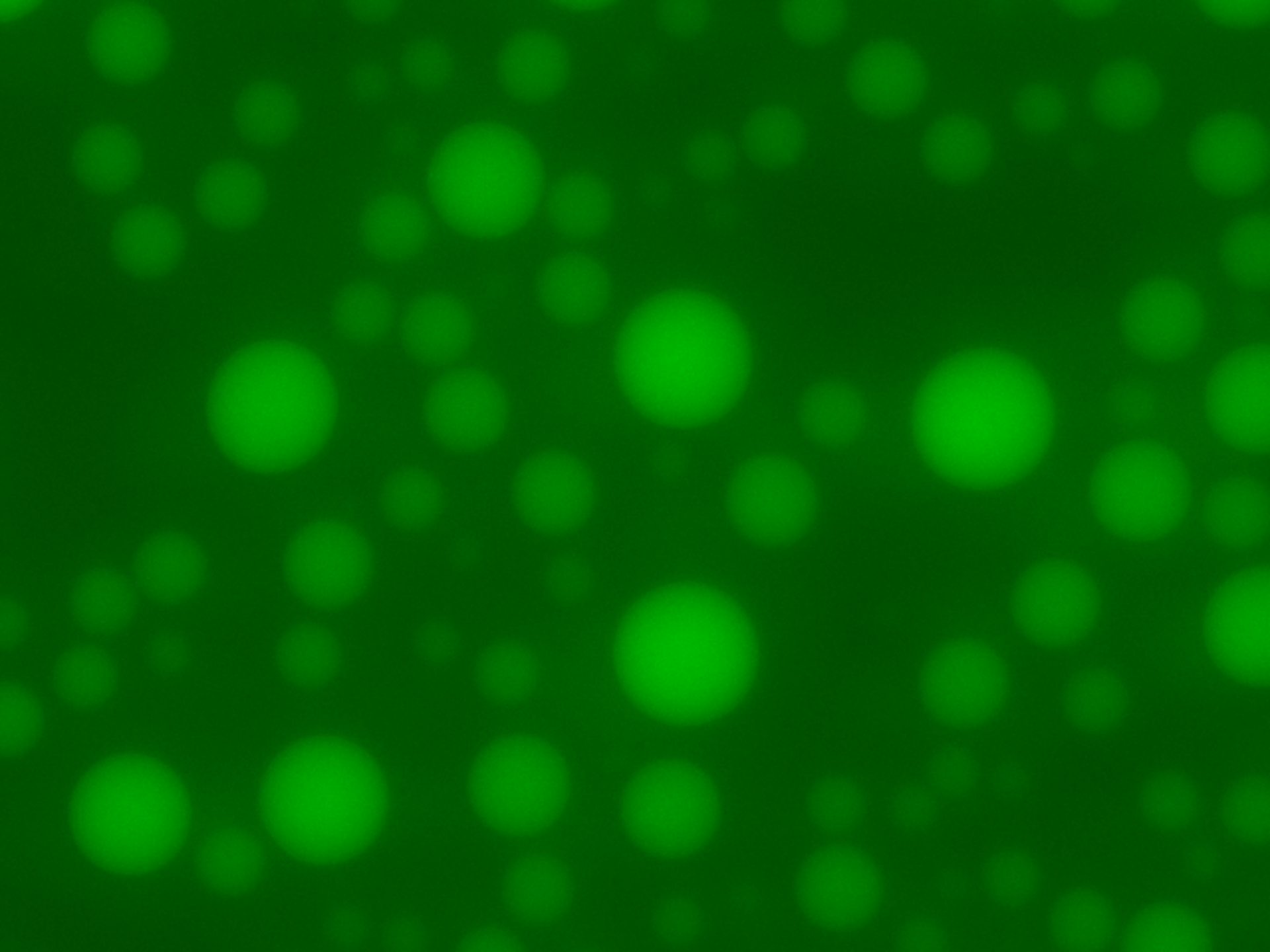

Supplement: Supplementary file 6 — Source data Fig. 4 [file 44318_2025_591_MOESM6_ESM.zip › Figure 4/4F/(a)18_48 h_SO286(20 ╬╝M).tif]

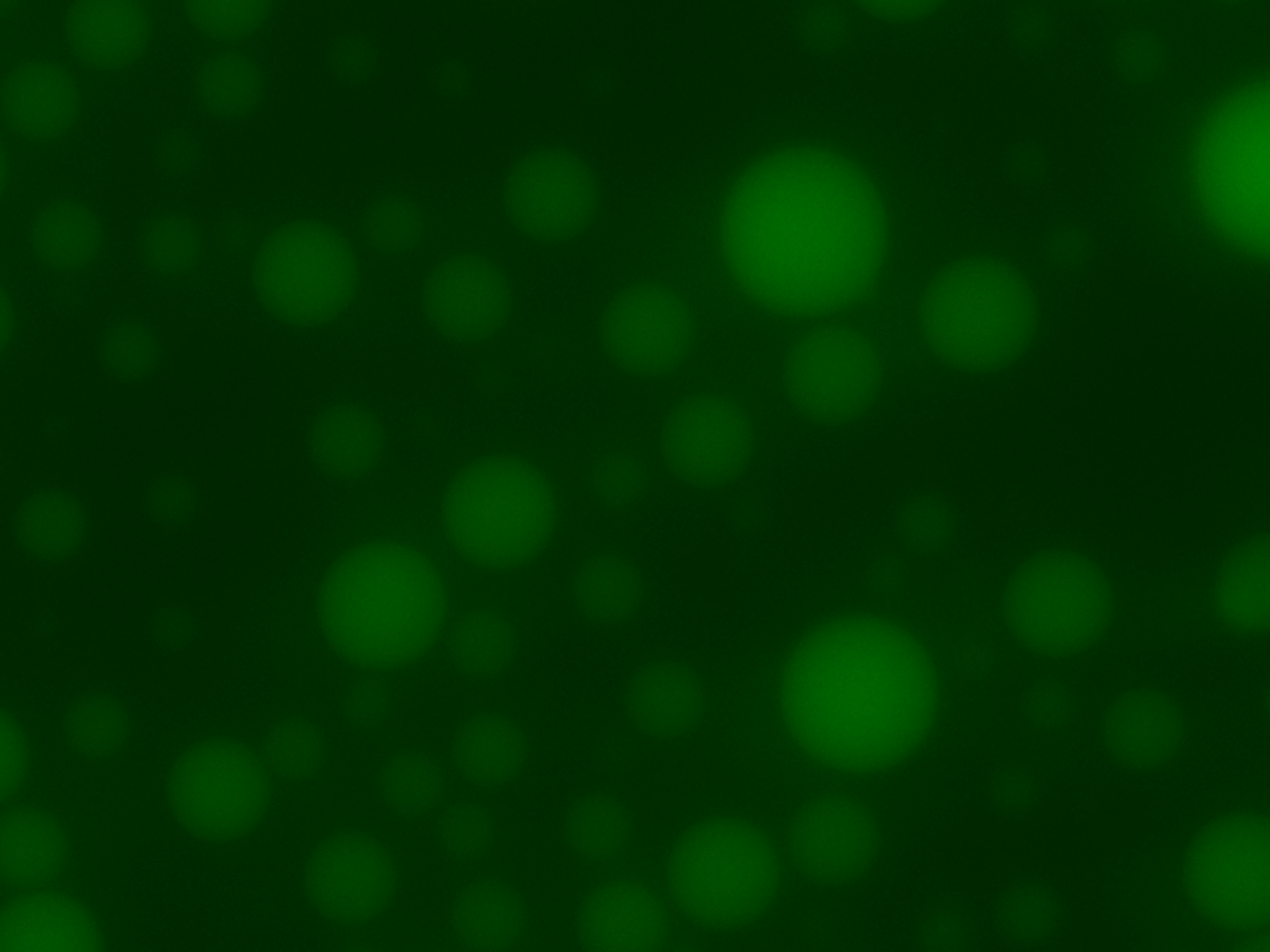

Supplement: Supplementary file 6 — Source data Fig. 4 [file 44318_2025_591_MOESM6_ESM.zip › Figure 4/4F/(a)19_72 h_SO286(20 ╬╝M).tif]

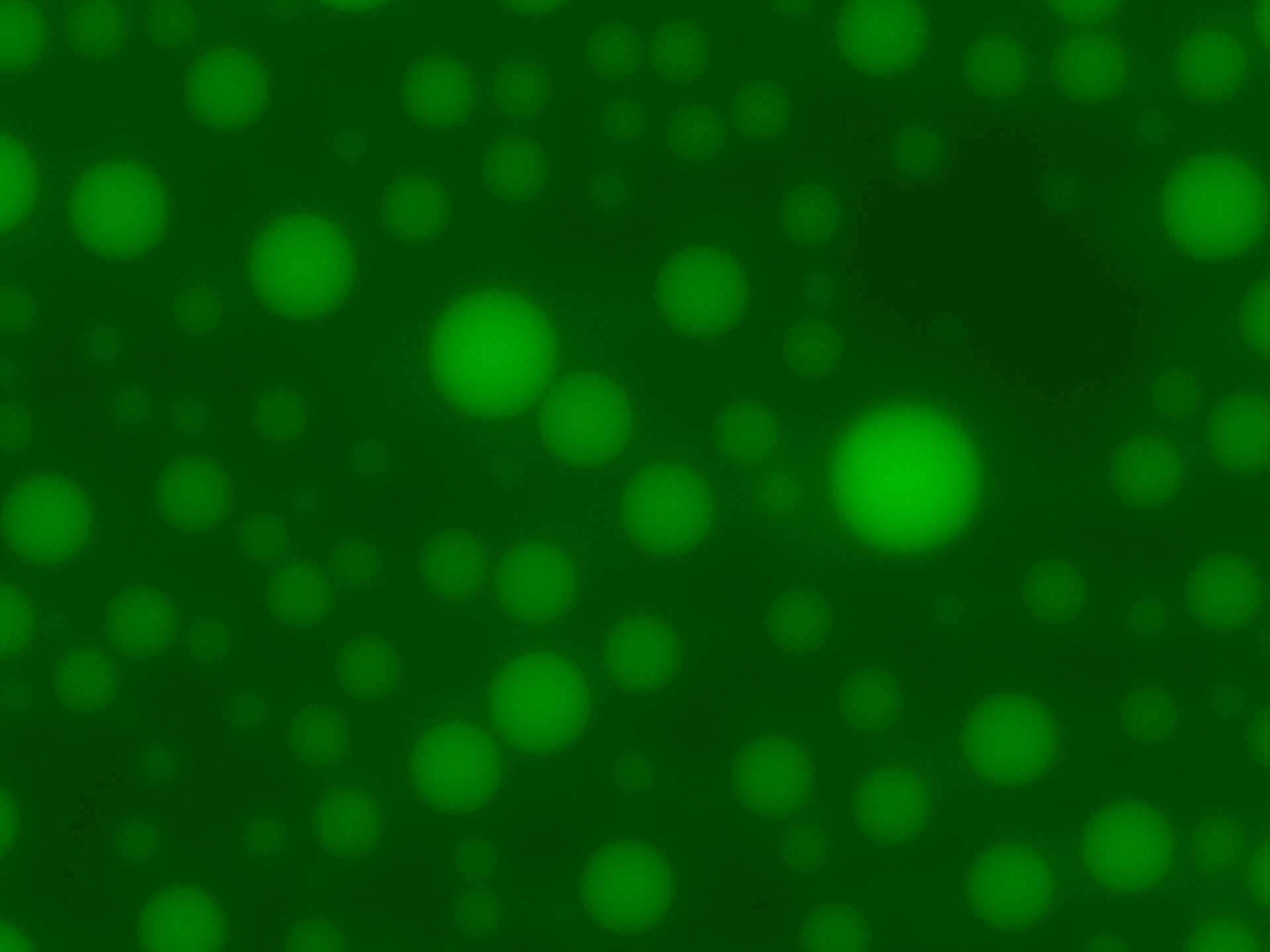

Supplement: Supplementary file 6 — Source data Fig. 4 [file 44318_2025_591_MOESM6_ESM.zip › Figure 4/4F/(a)17_24 h_SO286(20 ╬╝M).tif]

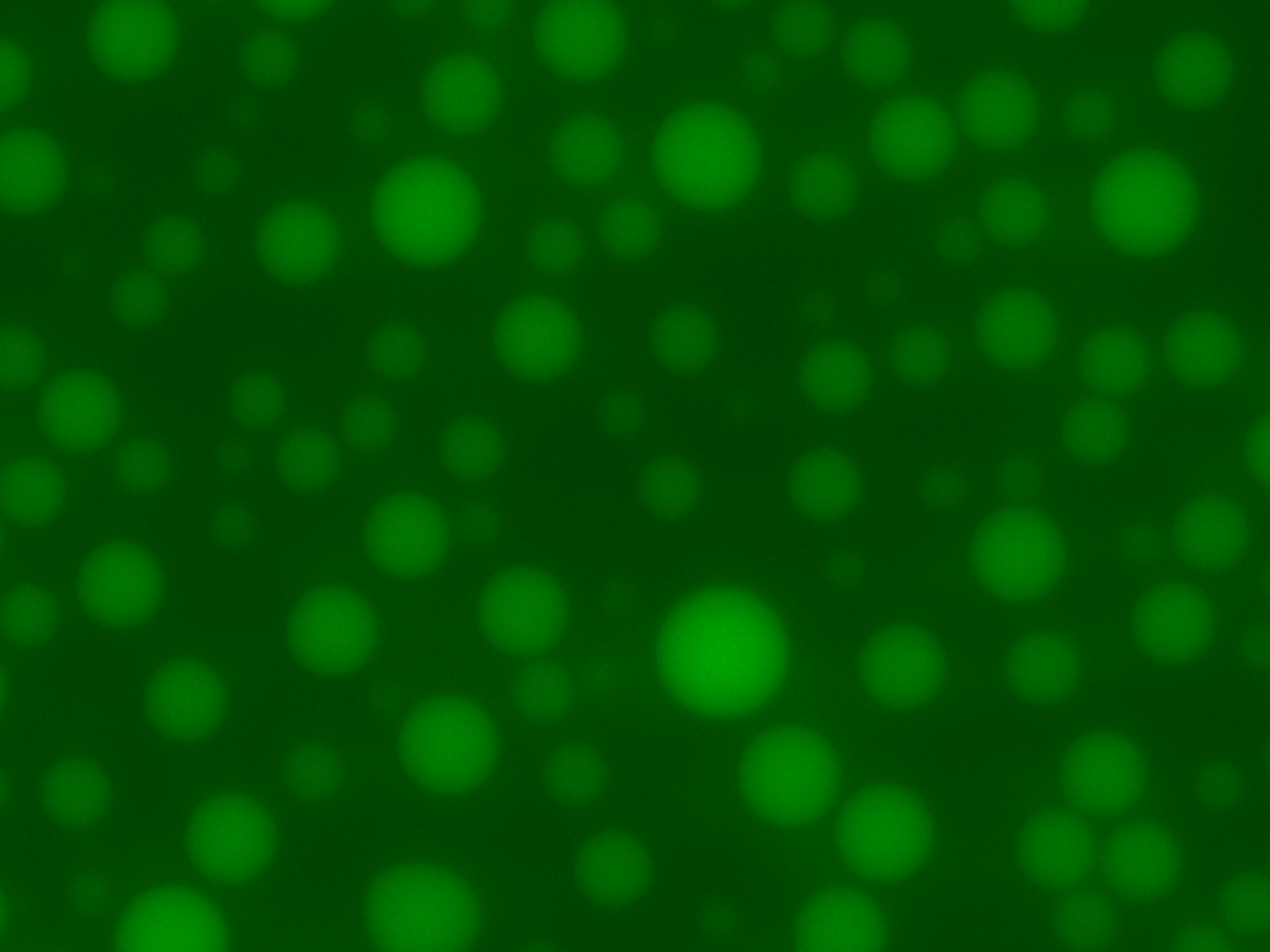

Supplement: Supplementary file 6 — Source data Fig. 4 [file 44318_2025_591_MOESM6_ESM.zip › Figure 4/4F/(a)12_24 h_SO286(7 ╬╝M).tif]

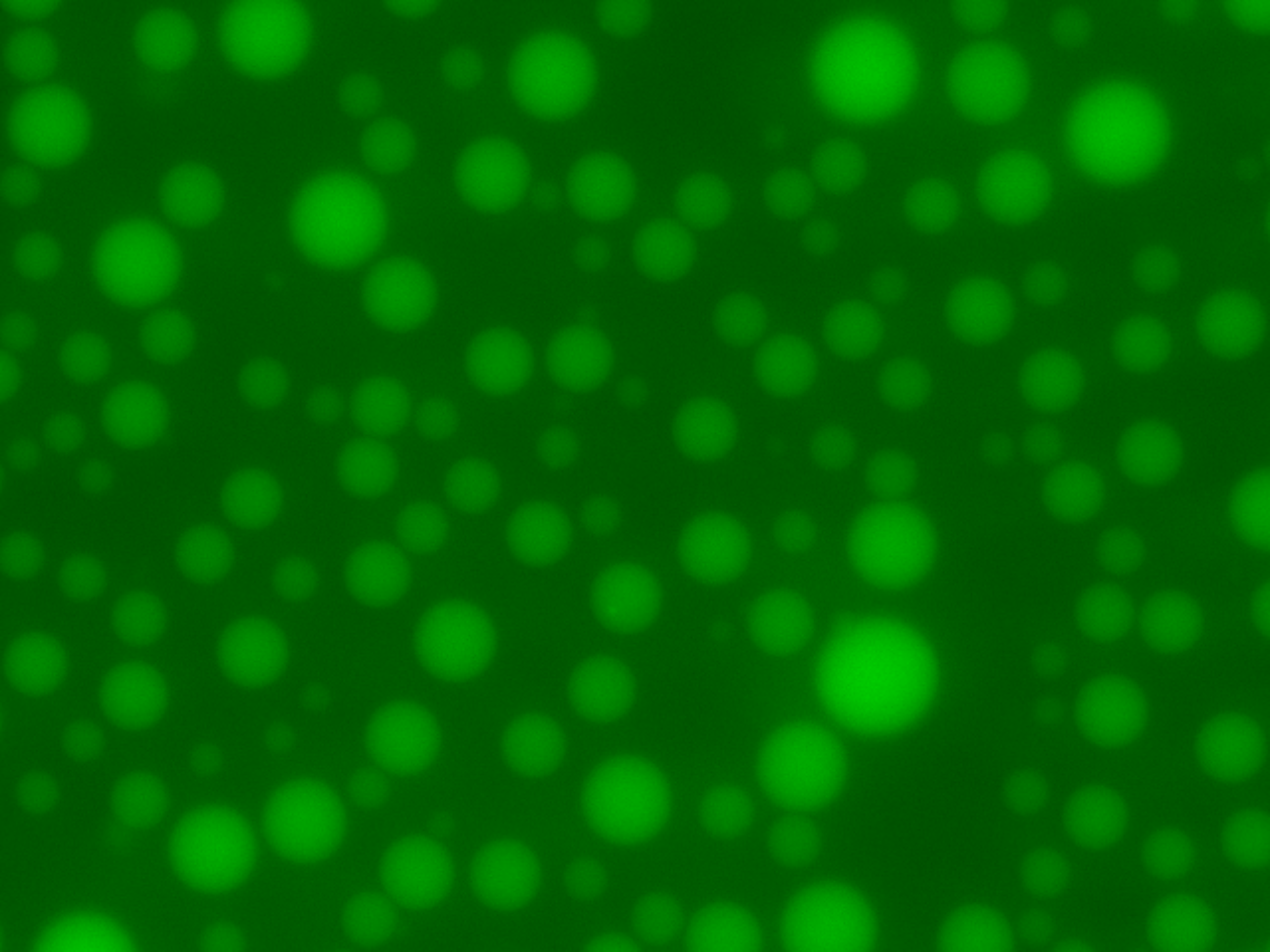

Supplement: Supplementary file 6 — Source data Fig. 4 [file 44318_2025_591_MOESM6_ESM.zip › Figure 4/4F/(a)06_1 h_SO286(2 ╬╝M).tif]

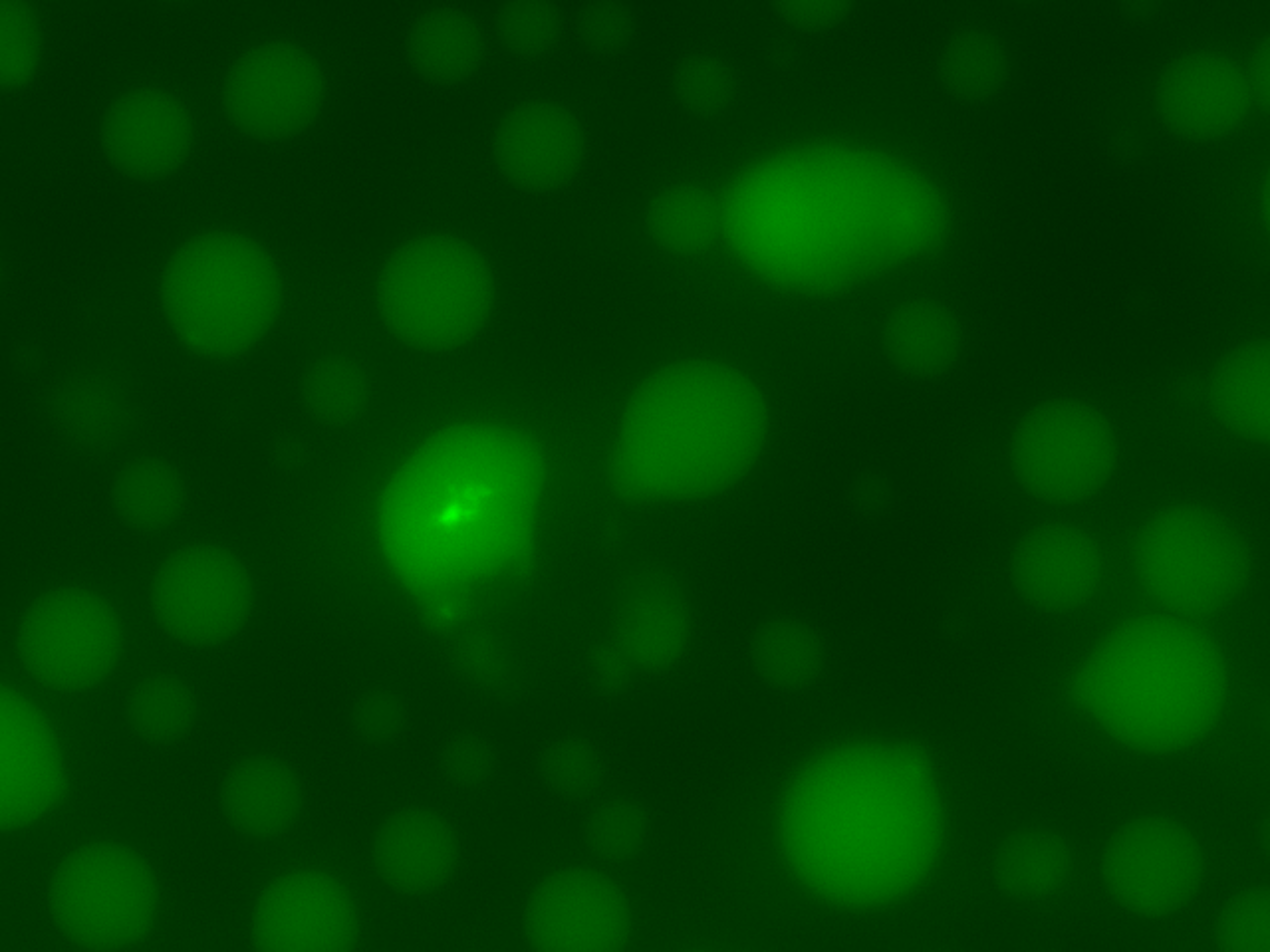

Supplement: Supplementary file 6 — Source data Fig. 4 [file 44318_2025_591_MOESM6_ESM.zip › Figure 4/4F/(a)09_72 h_SO286(2 ╬╝M).tif]

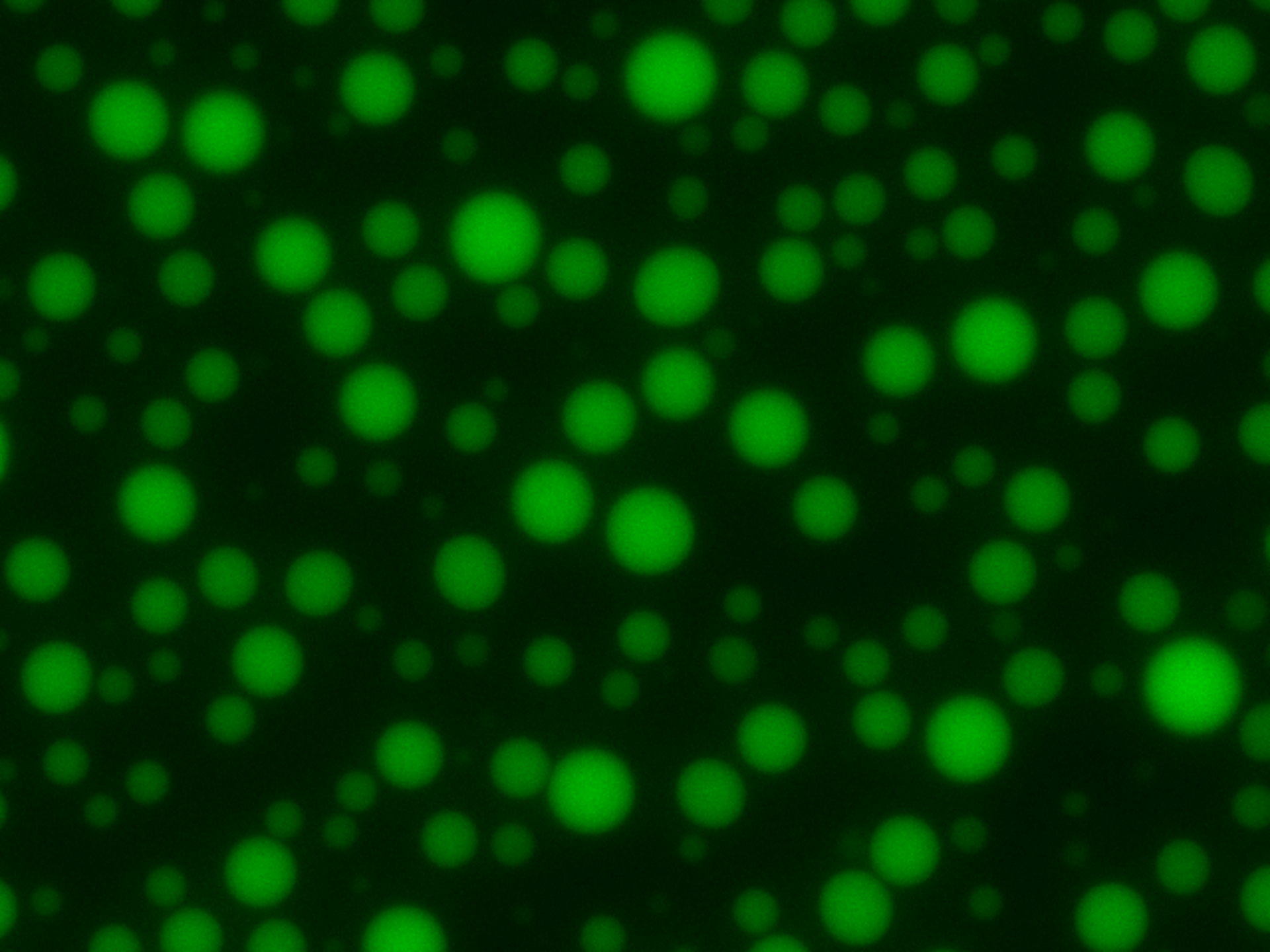

Supplement: Supplementary file 6 — Source data Fig. 4 [file 44318_2025_591_MOESM6_ESM.zip › Figure 4/4F/(b)_01_Control_24h.tif]

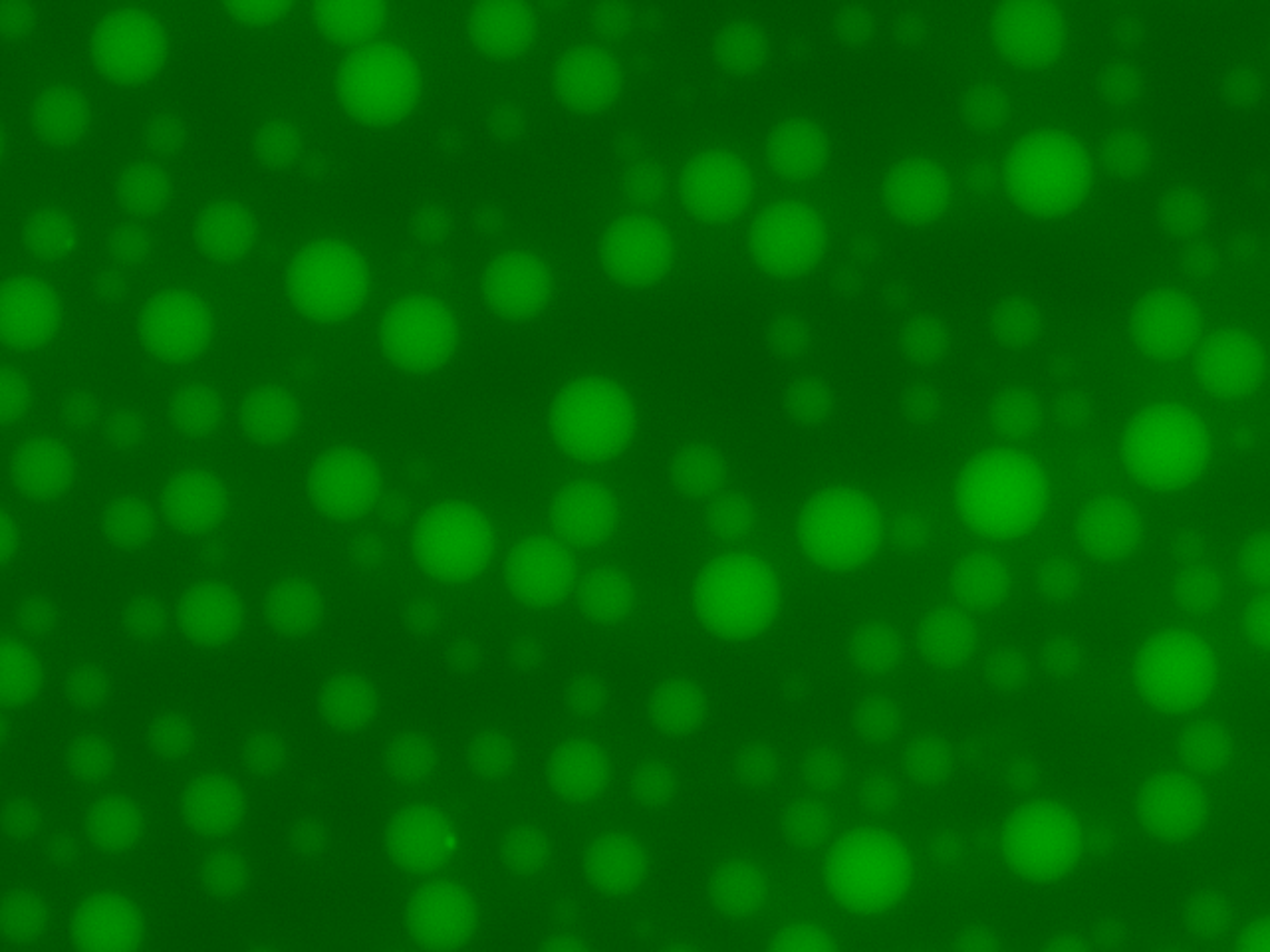

Supplement: Supplementary file 6 — Source data Fig. 4 [file 44318_2025_591_MOESM6_ESM.zip › Figure 4/4F/(a)01_1 h_None.tif]

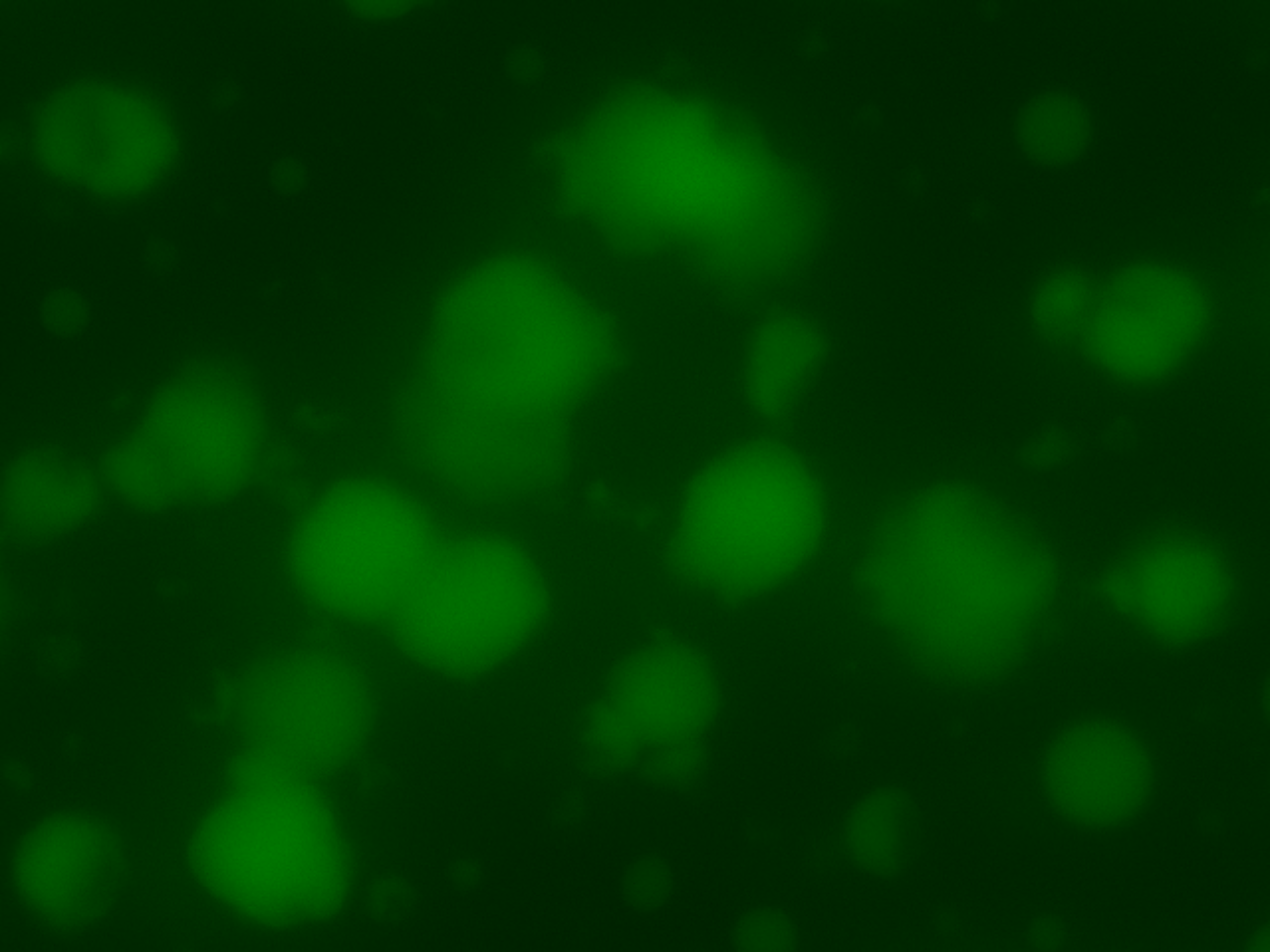

Supplement: Supplementary file 6 — Source data Fig. 4 [file 44318_2025_591_MOESM6_ESM.zip › Figure 4/4F/(a)10_96 h_SO286(2 ╬╝M).tif]

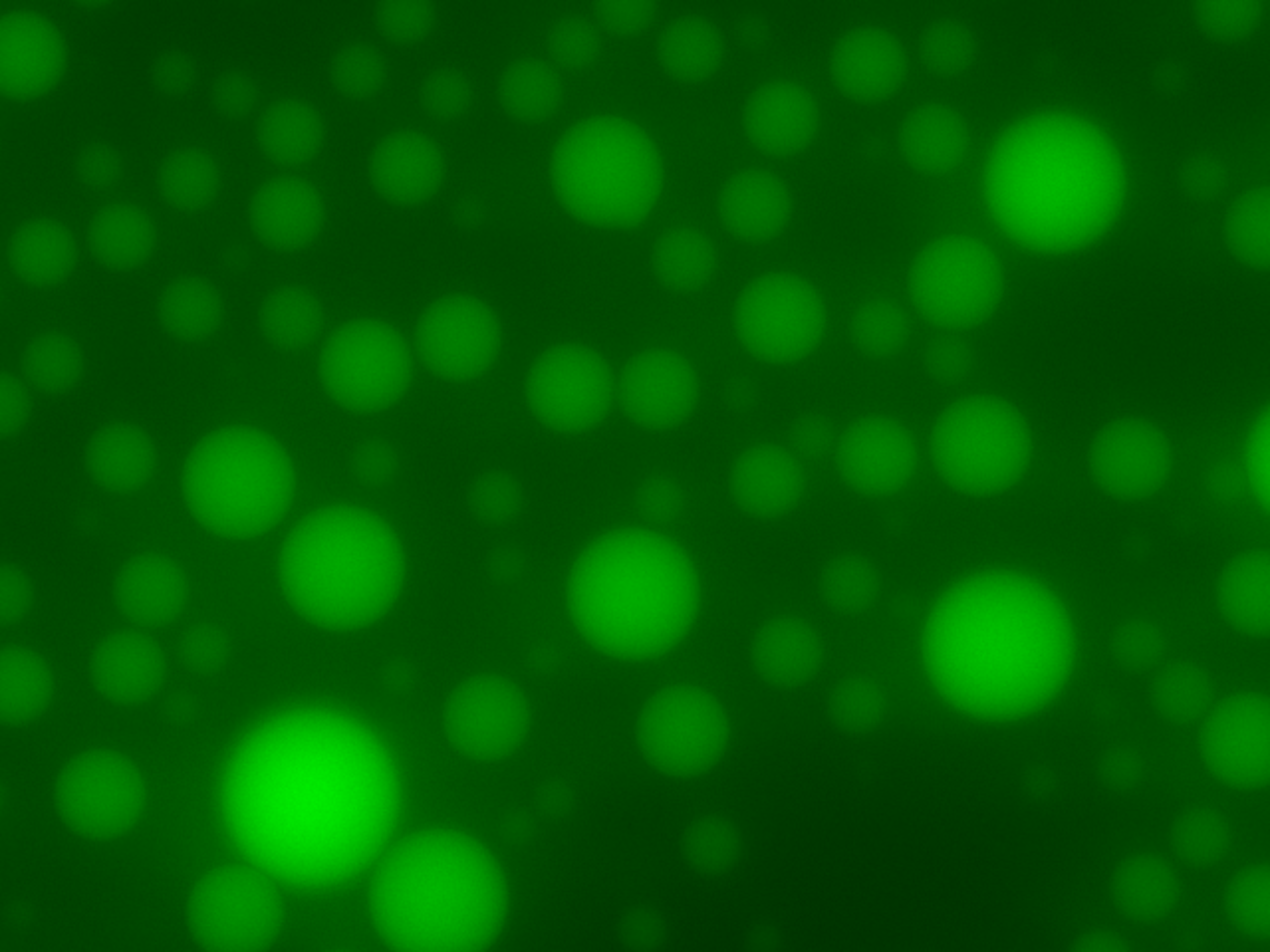

Supplement: Supplementary file 6 — Source data Fig. 4 [file 44318_2025_591_MOESM6_ESM.zip › Figure 4/4F/(a)07_24 h_SO286(2 ╬╝M).tif]

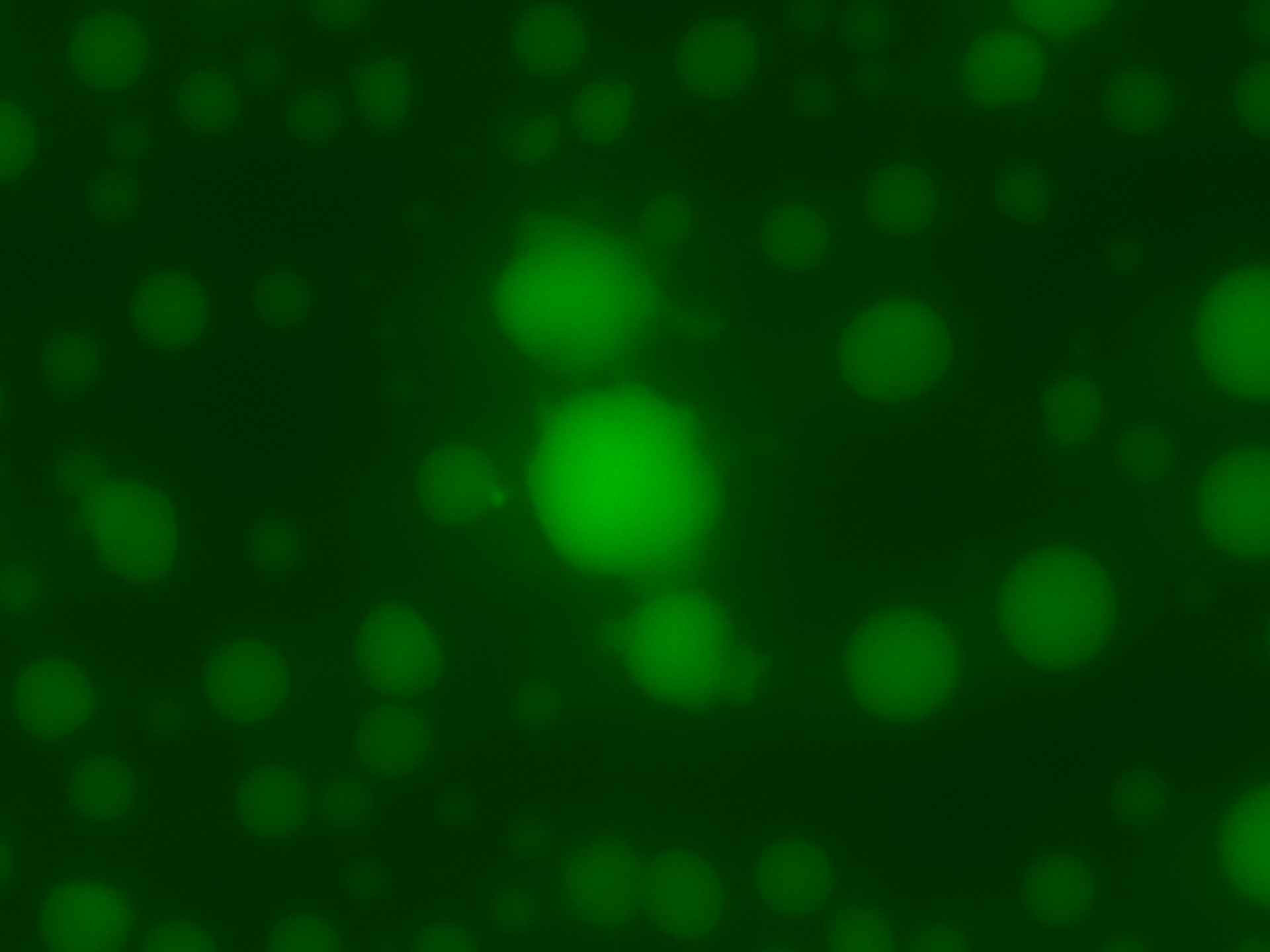

Supplement: Supplementary file 6 — Source data Fig. 4 [file 44318_2025_591_MOESM6_ESM.zip › Figure 4/4F/(a)04_72 h_None.tif]

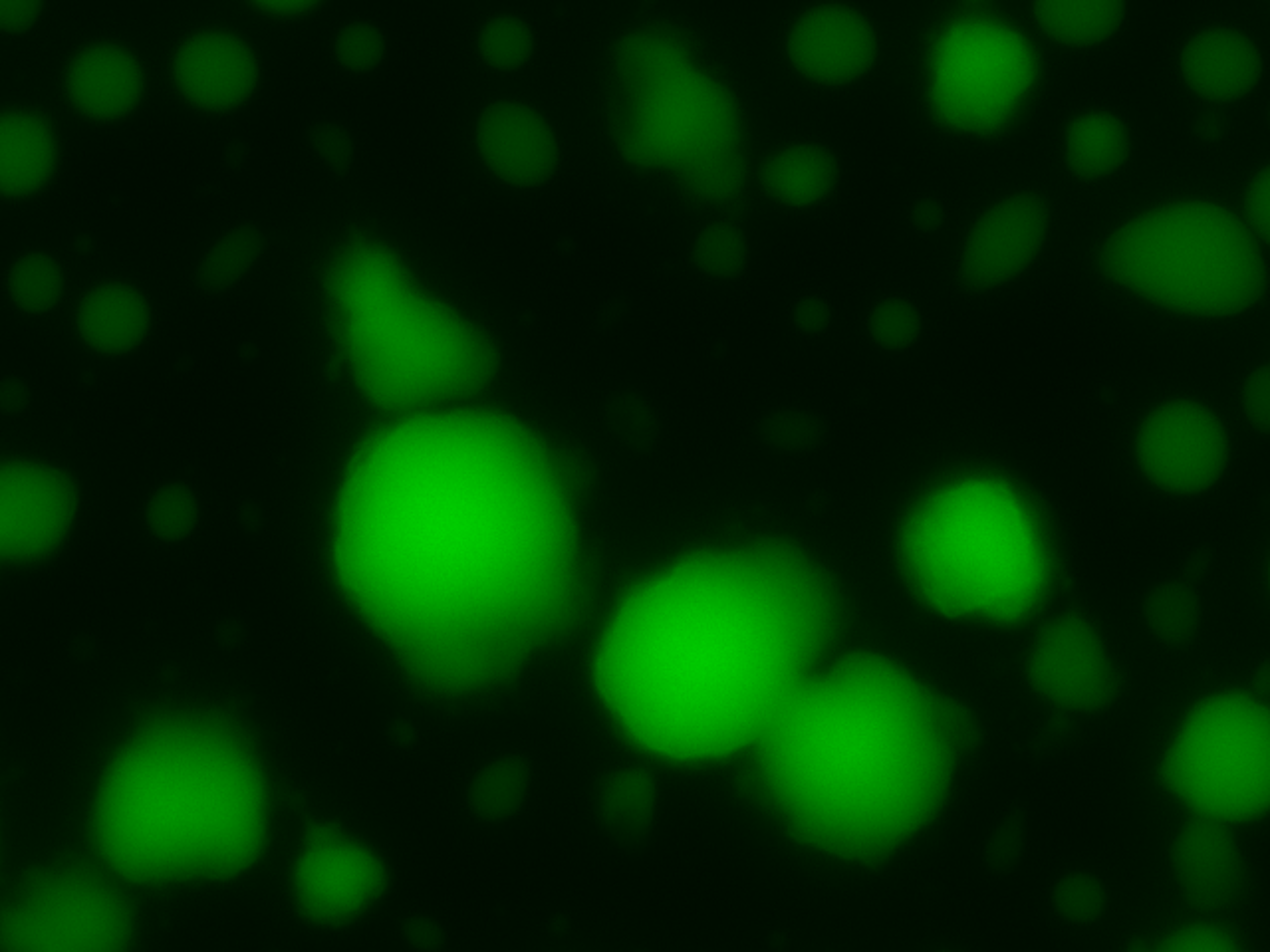

Supplement: Supplementary file 6 — Source data Fig. 4 [file 44318_2025_591_MOESM6_ESM.zip › Figure 4/4F/(b)_06_SO82_96h.tif]

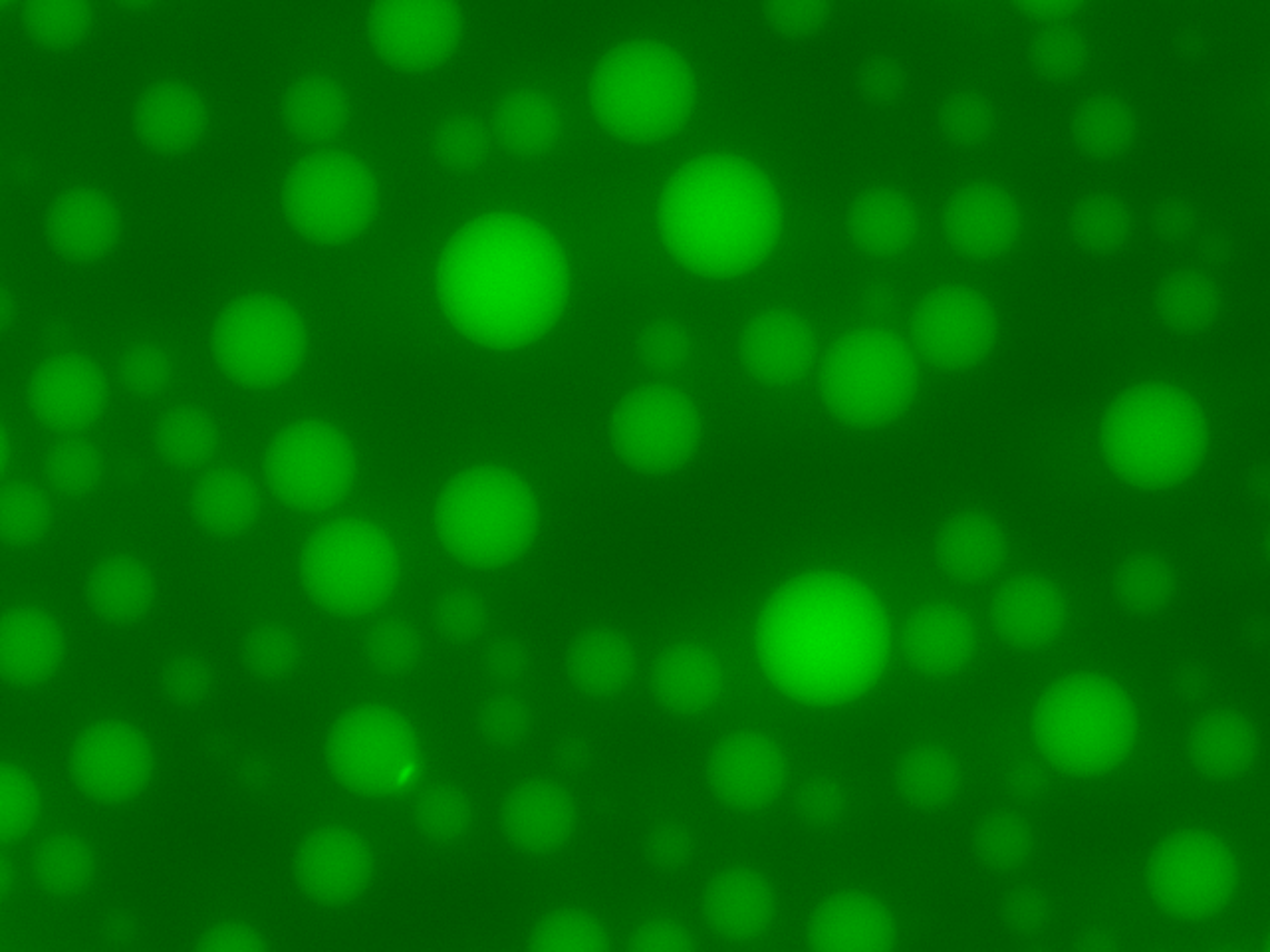

Supplement: Supplementary file 6 — Source data Fig. 4 [file 44318_2025_591_MOESM6_ESM.zip › Figure 4/4F/(a)03_48 h_None.tif]

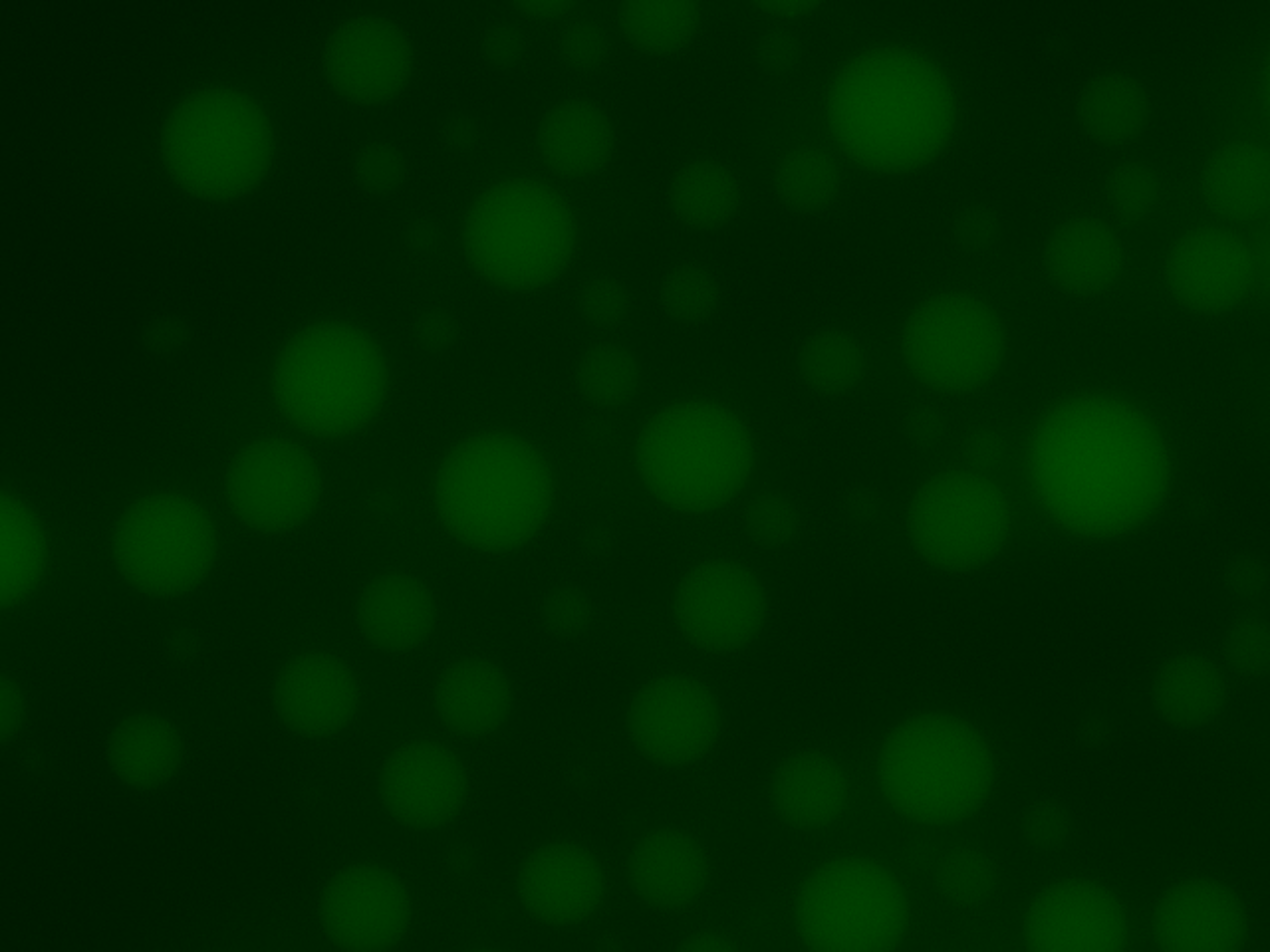

Supplement: Supplementary file 6 — Source data Fig. 4 [file 44318_2025_591_MOESM6_ESM.zip › Figure 4/4F/(a)20_96 h_SO286(20 ╬╝M).tif]

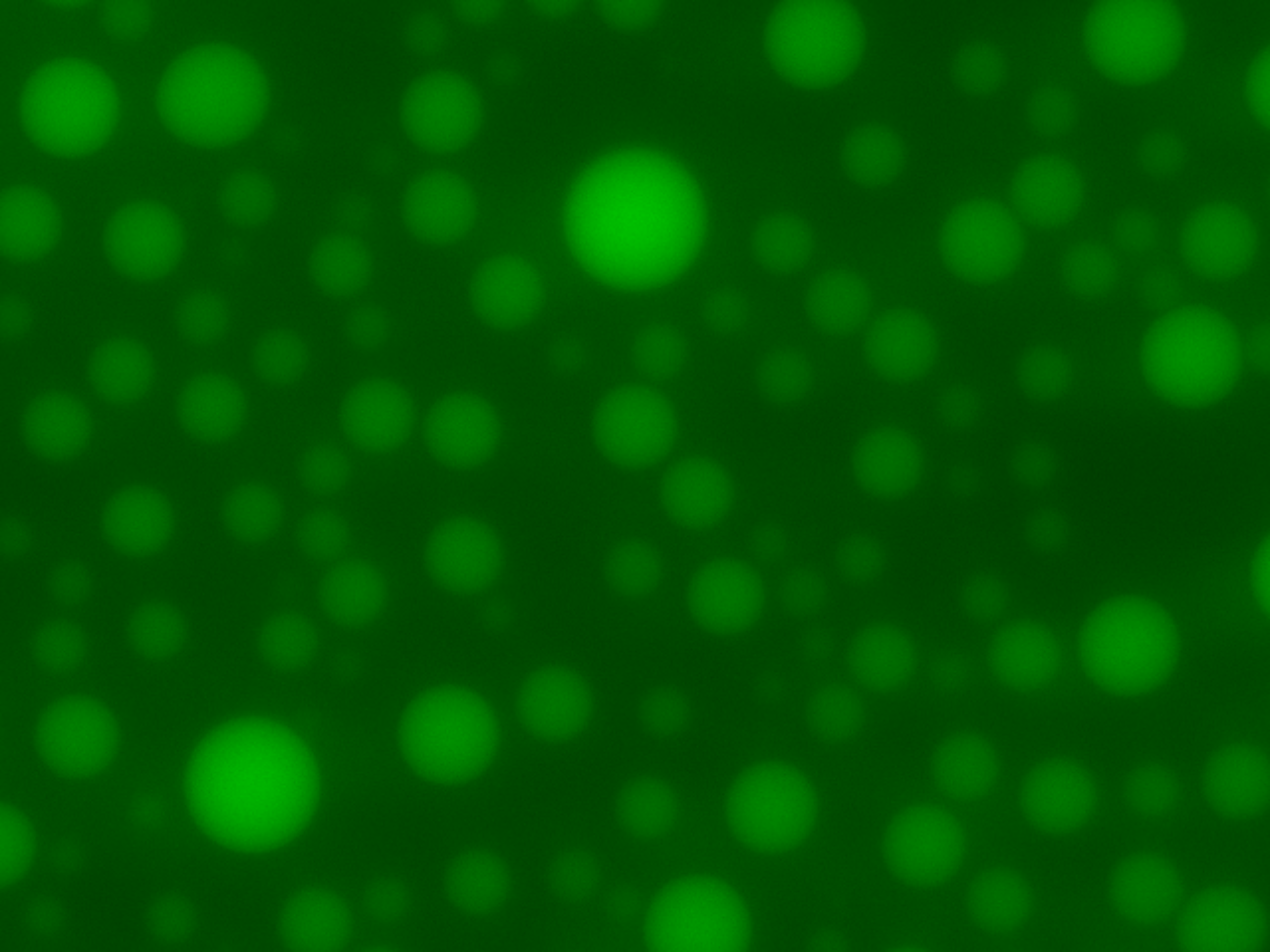

Supplement: Supplementary file 6 — Source data Fig. 4 [file 44318_2025_591_MOESM6_ESM.zip › Figure 4/4F/(a)02_24 h_None.tif]

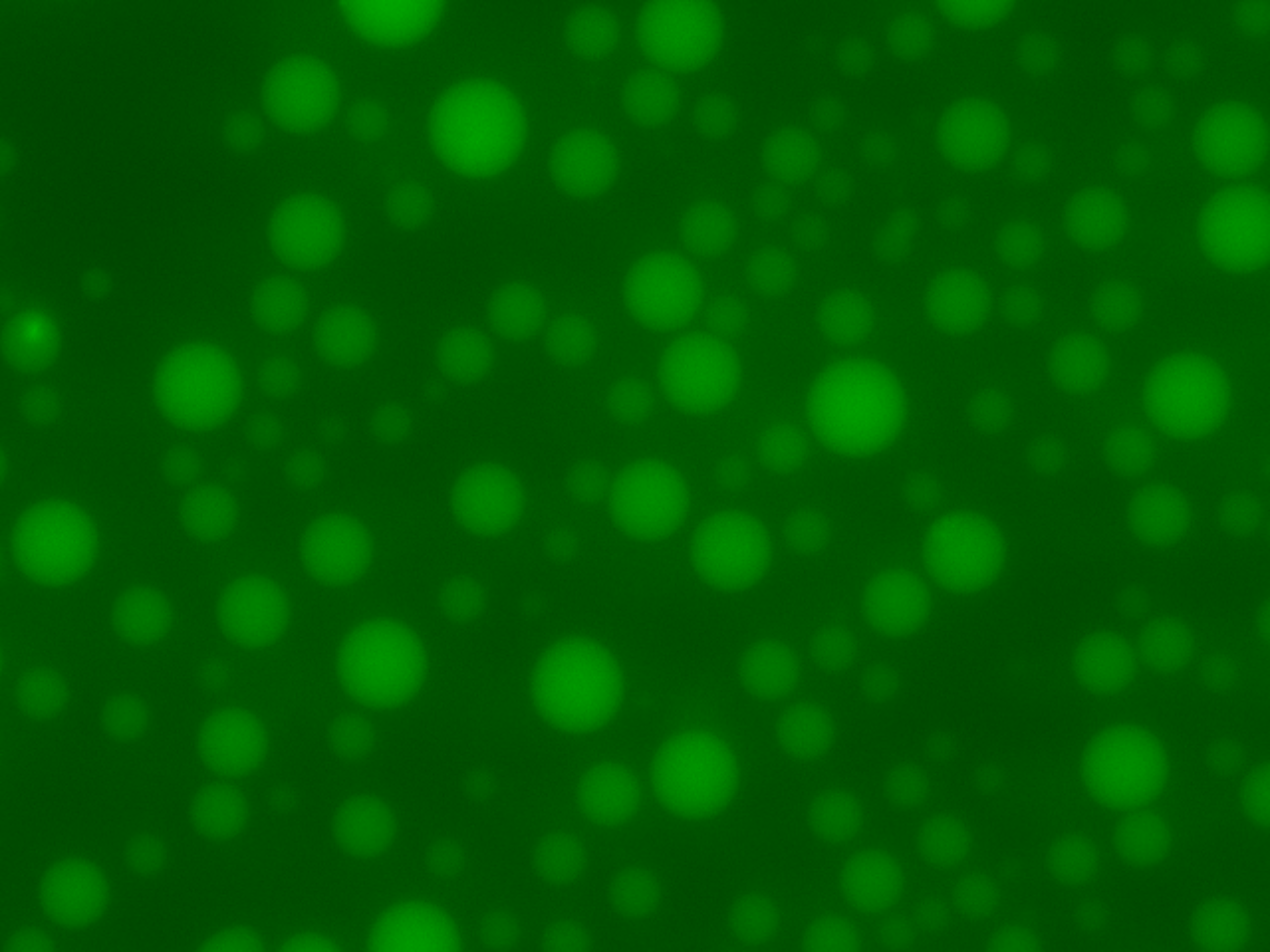

Supplement: Supplementary file 6 — Source data Fig. 4 [file 44318_2025_591_MOESM6_ESM.zip › Figure 4/4F/(a)16_1 h_SO286(20 ╬╝M).tif]

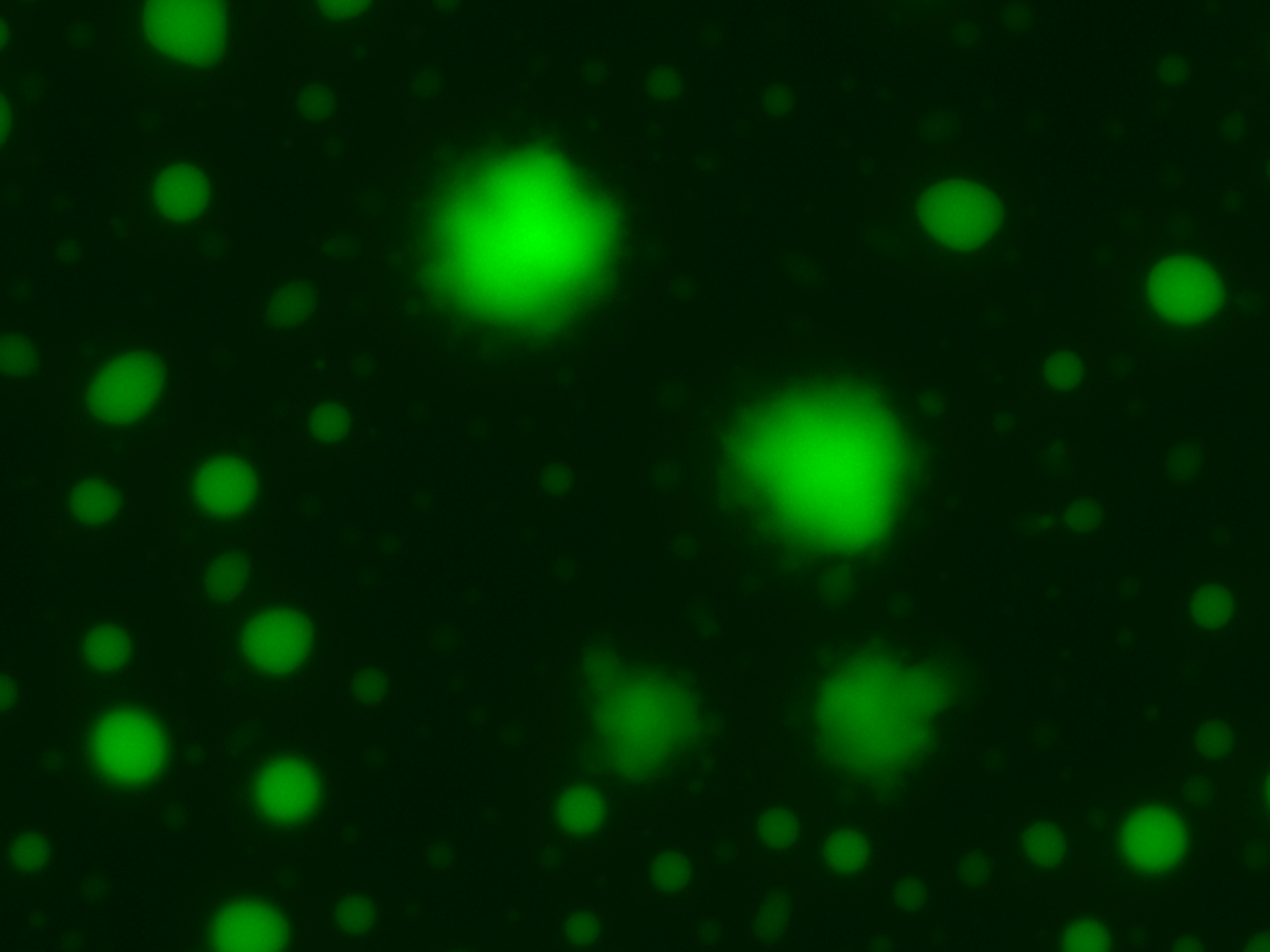

Supplement: Supplementary file 6 — Source data Fig. 4 [file 44318_2025_591_MOESM6_ESM.zip › Figure 4/4F/(b)_02_Control_96h.tif]

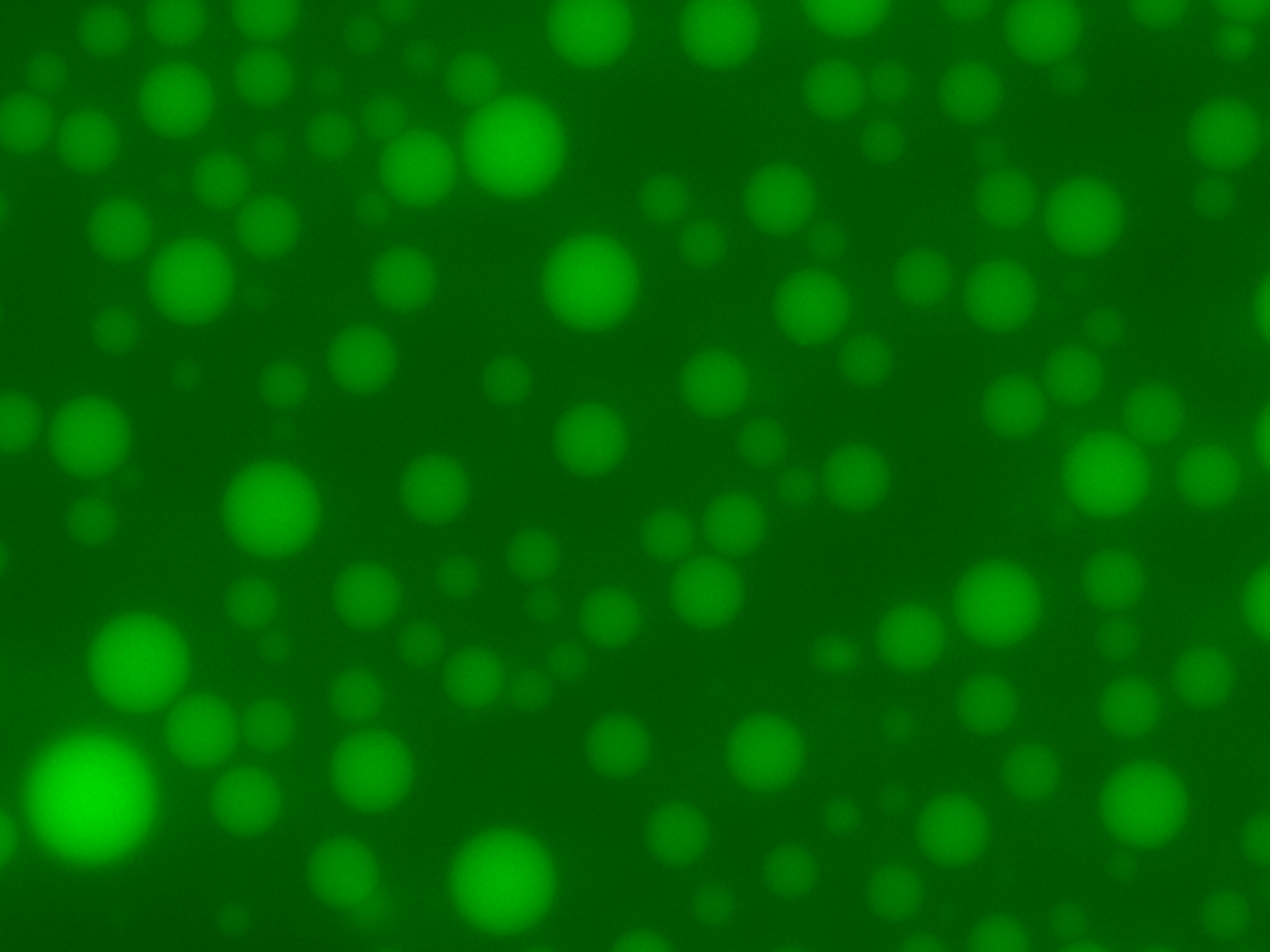

Supplement: Supplementary file 6 — Source data Fig. 4 [file 44318_2025_591_MOESM6_ESM.zip › Figure 4/4F/(a)11_1 h_SO286(7 ╬╝M).tif]

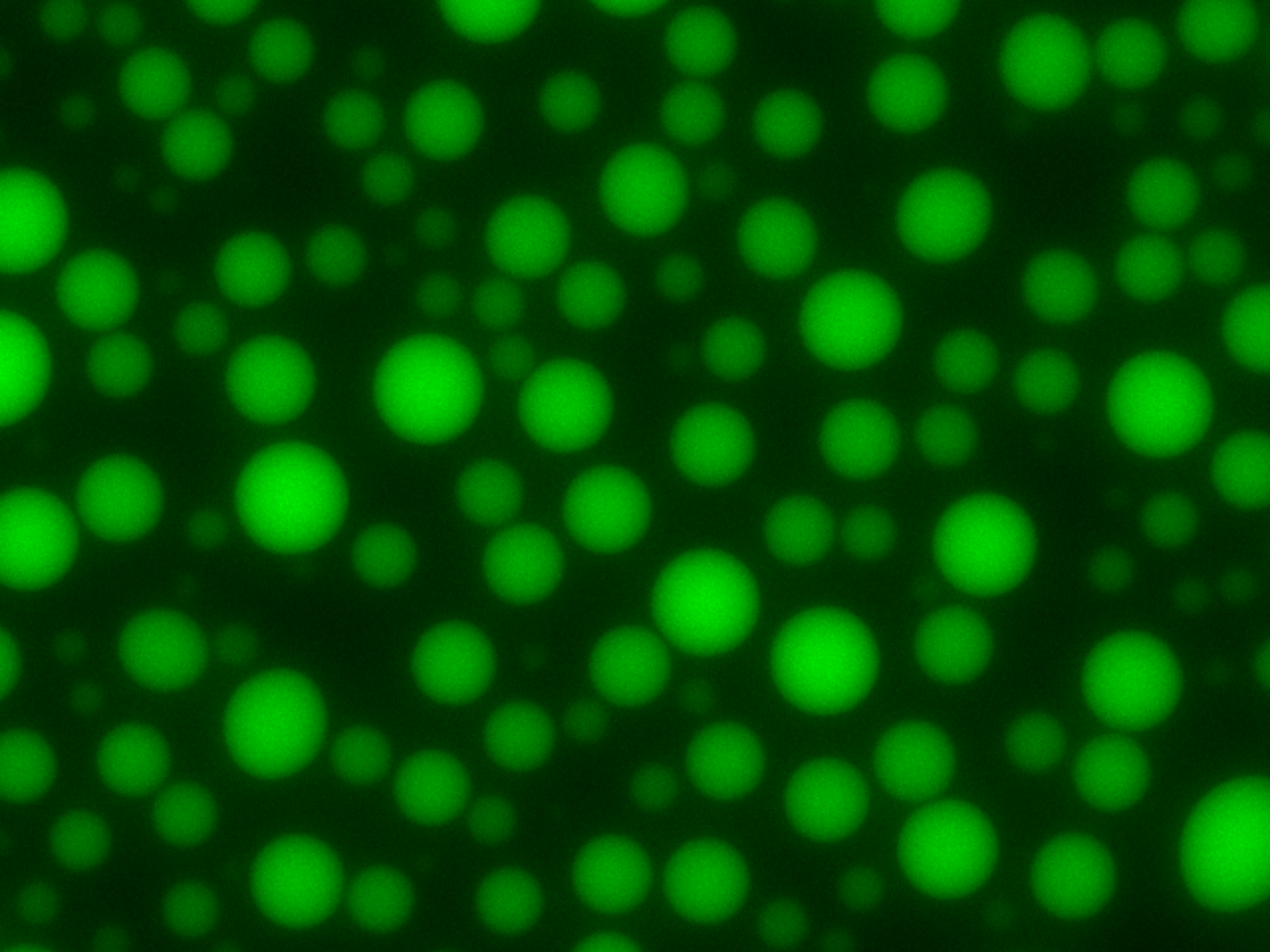

Supplement: Supplementary file 6 — Source data Fig. 4 [file 44318_2025_591_MOESM6_ESM.zip › Figure 4/4F/(b)_03_SO286_24h.tif]

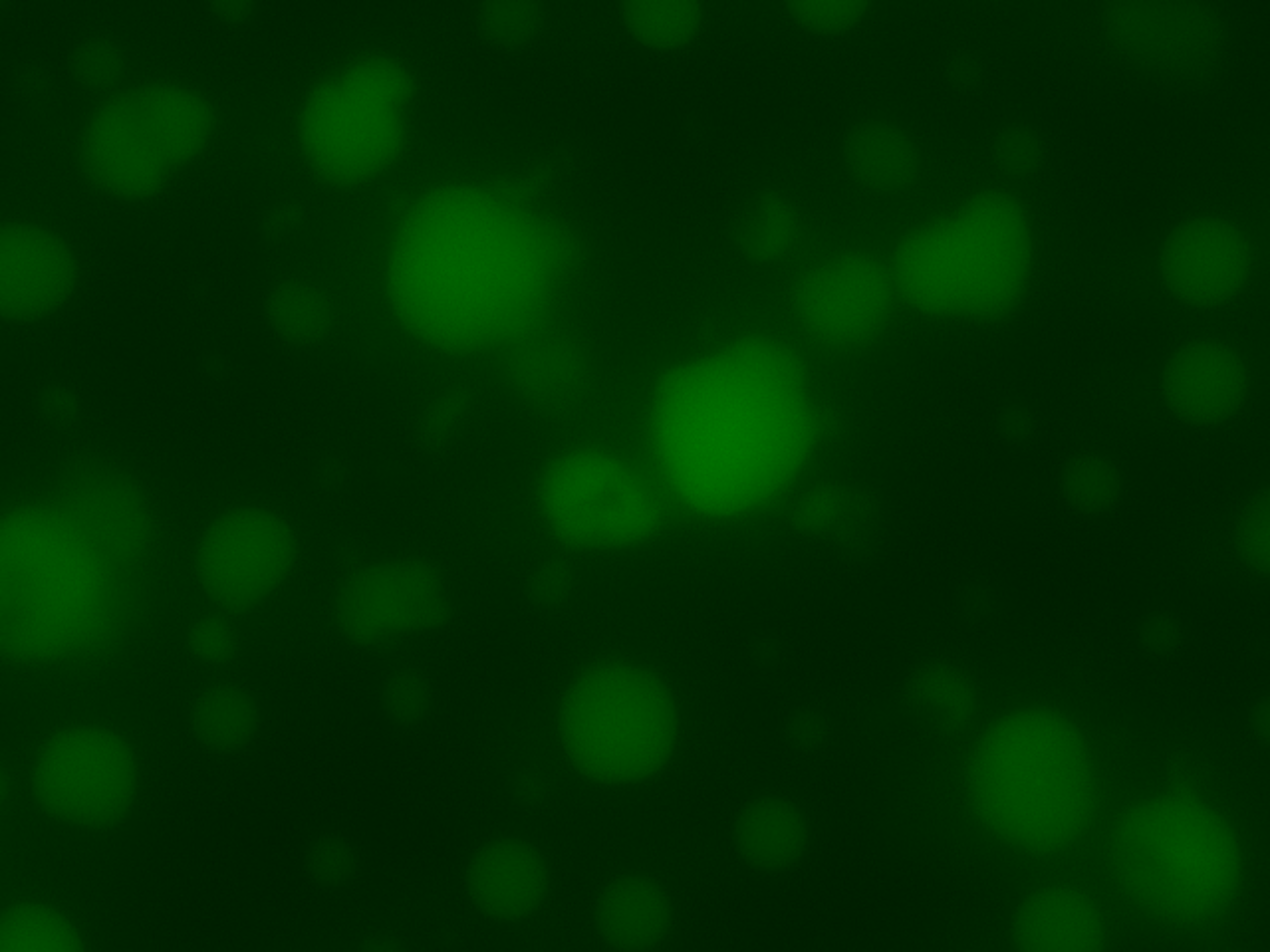

Supplement: Supplementary file 6 — Source data Fig. 4 [file 44318_2025_591_MOESM6_ESM.zip › Figure 4/4F/(a)15_96 h_SO286(7 ╬╝M).tif]

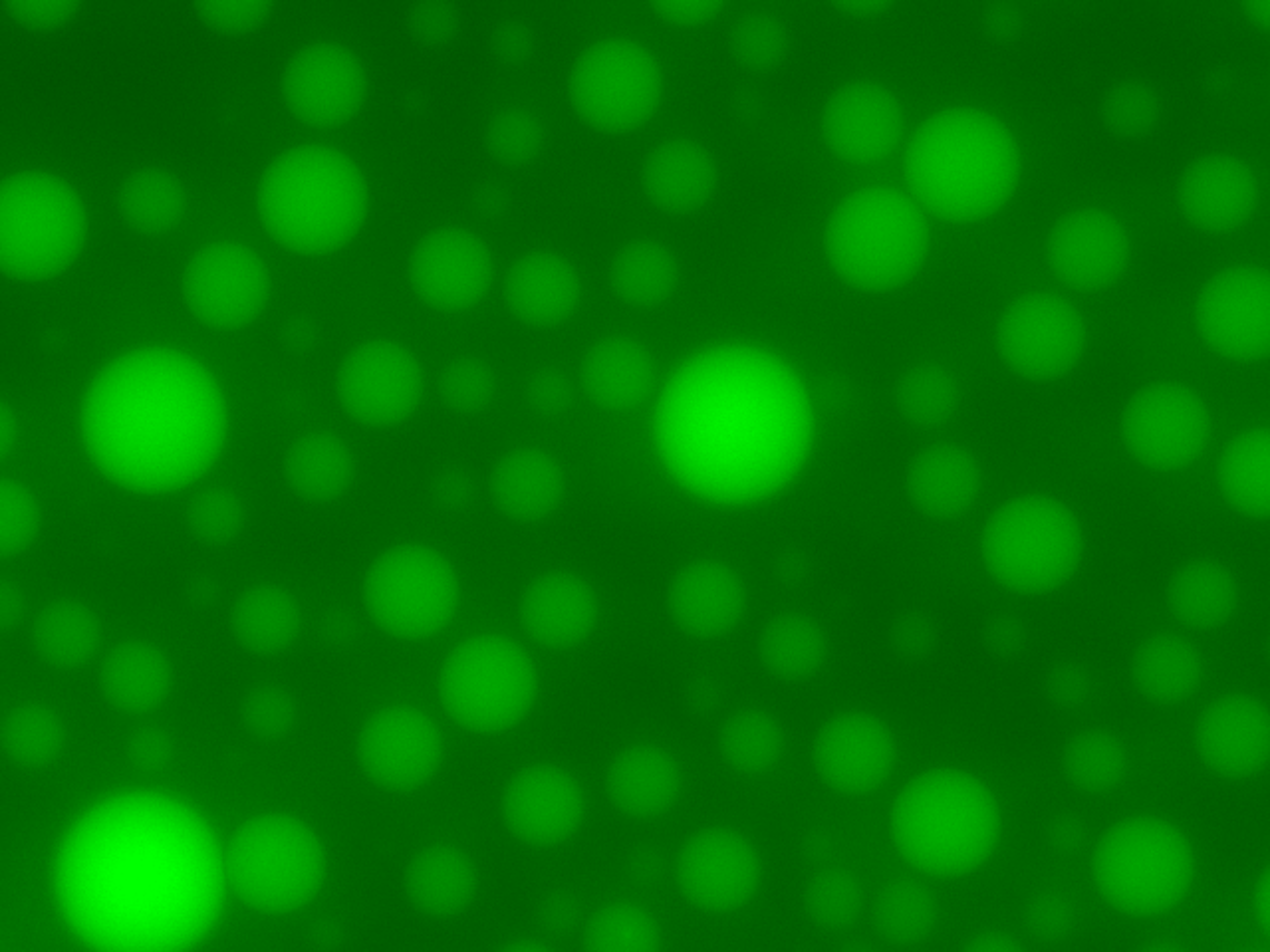

Supplement: Supplementary file 6 — Source data Fig. 4 [file 44318_2025_591_MOESM6_ESM.zip › Figure 4/4F/(a)08_48 h_SO286(2 ╬╝M).tif]

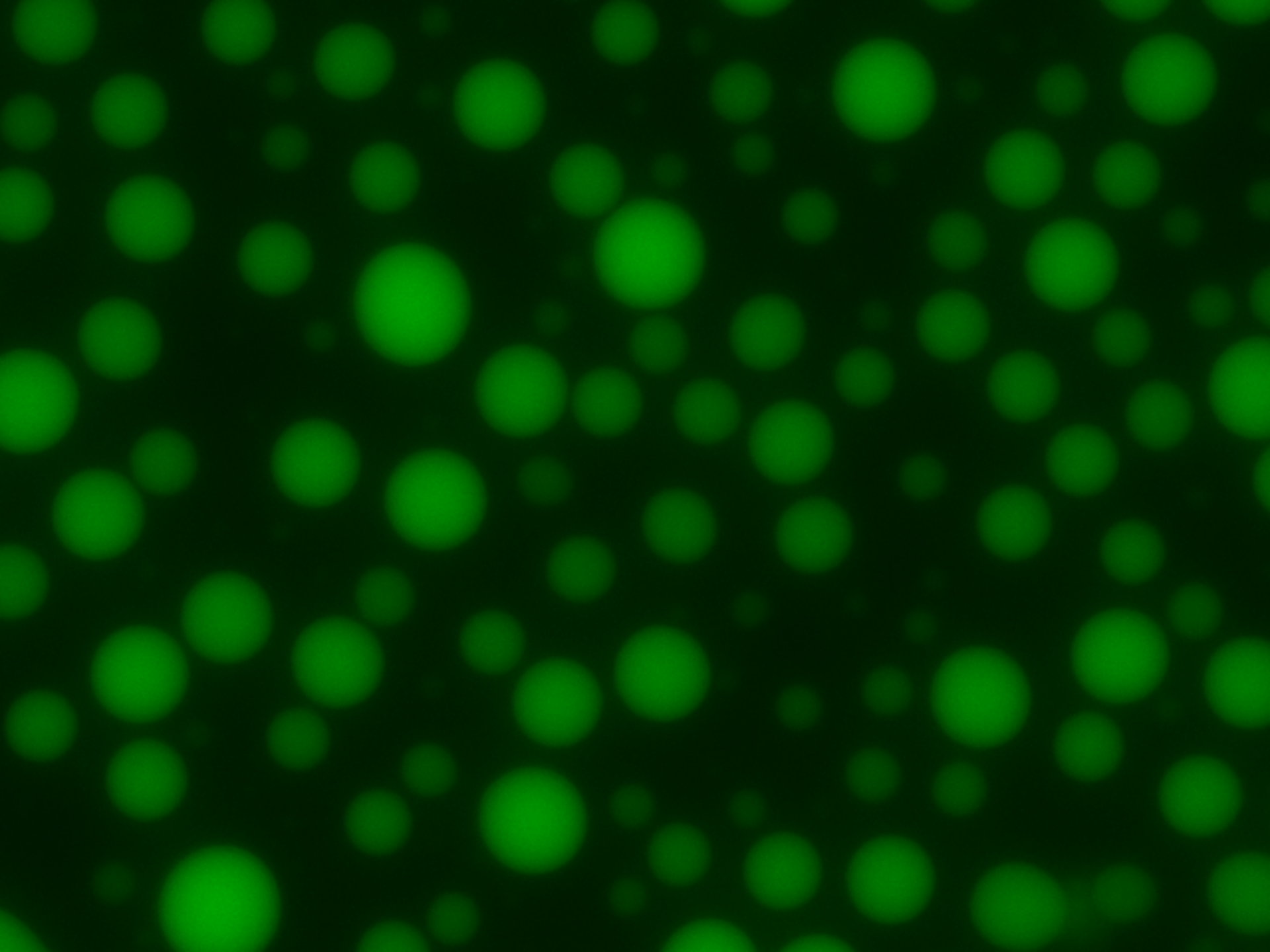

Supplement: Supplementary file 6 — Source data Fig. 4 [file 44318_2025_591_MOESM6_ESM.zip › Figure 4/4F/(b)_04_SO286_96h.tif]

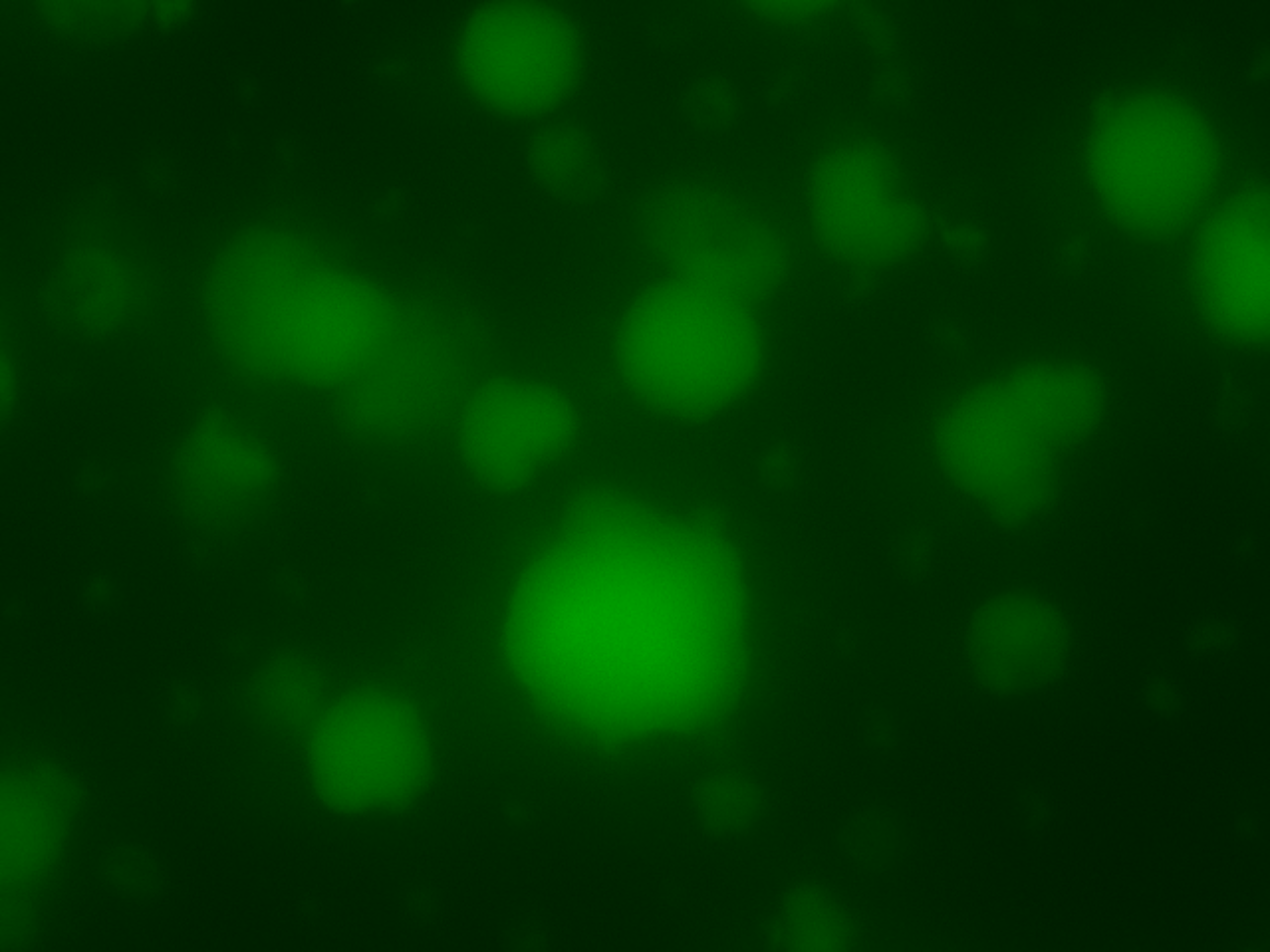

Supplement: Supplementary file 6 — Source data Fig. 4 [file 44318_2025_591_MOESM6_ESM.zip › Figure 4/4F/(a)05_96 h_None.tif]

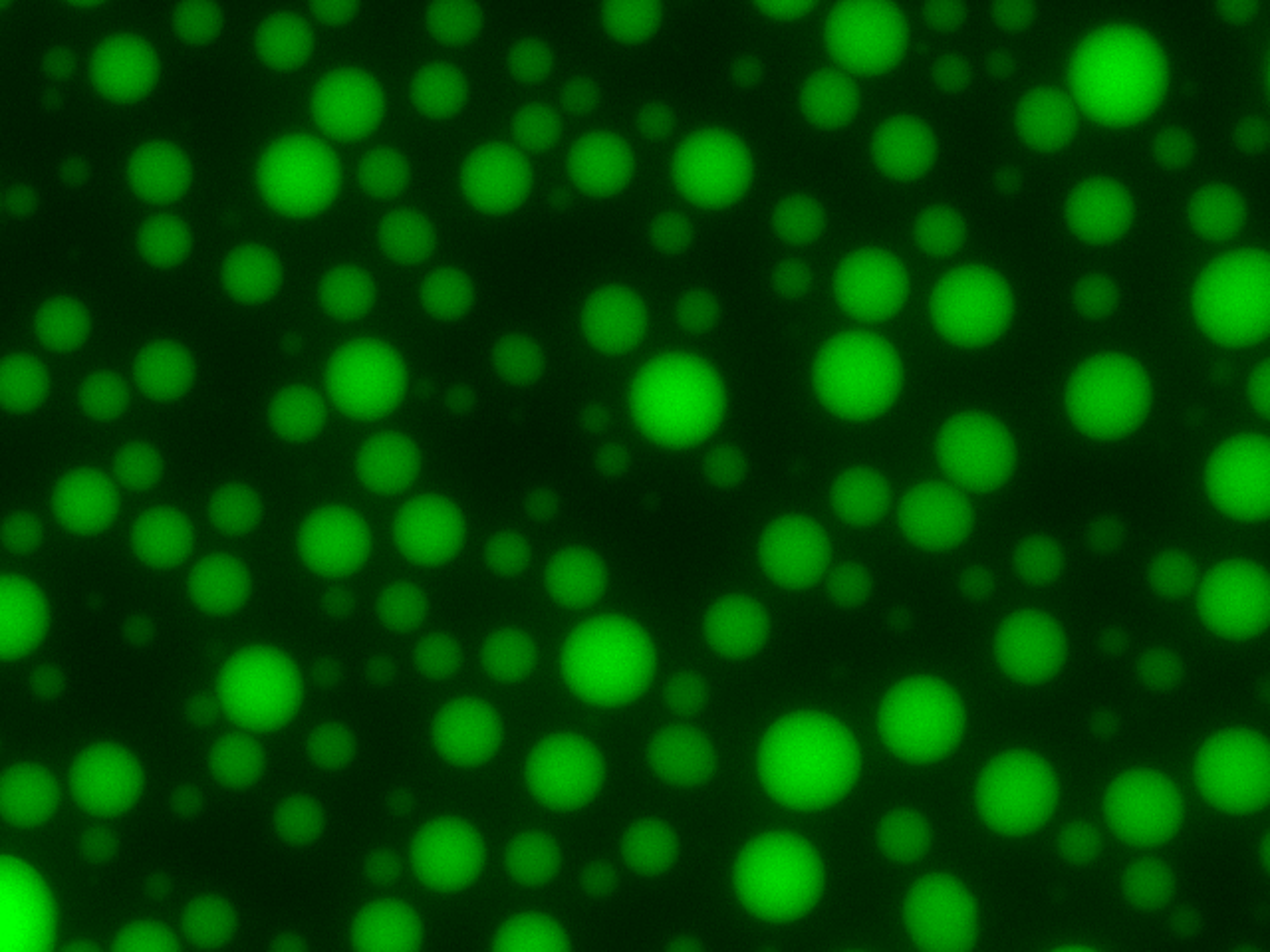

Supplement: Supplementary file 6 — Source data Fig. 4 [file 44318_2025_591_MOESM6_ESM.zip › Figure 4/4F/(b)_05_SO82_24h.tif]

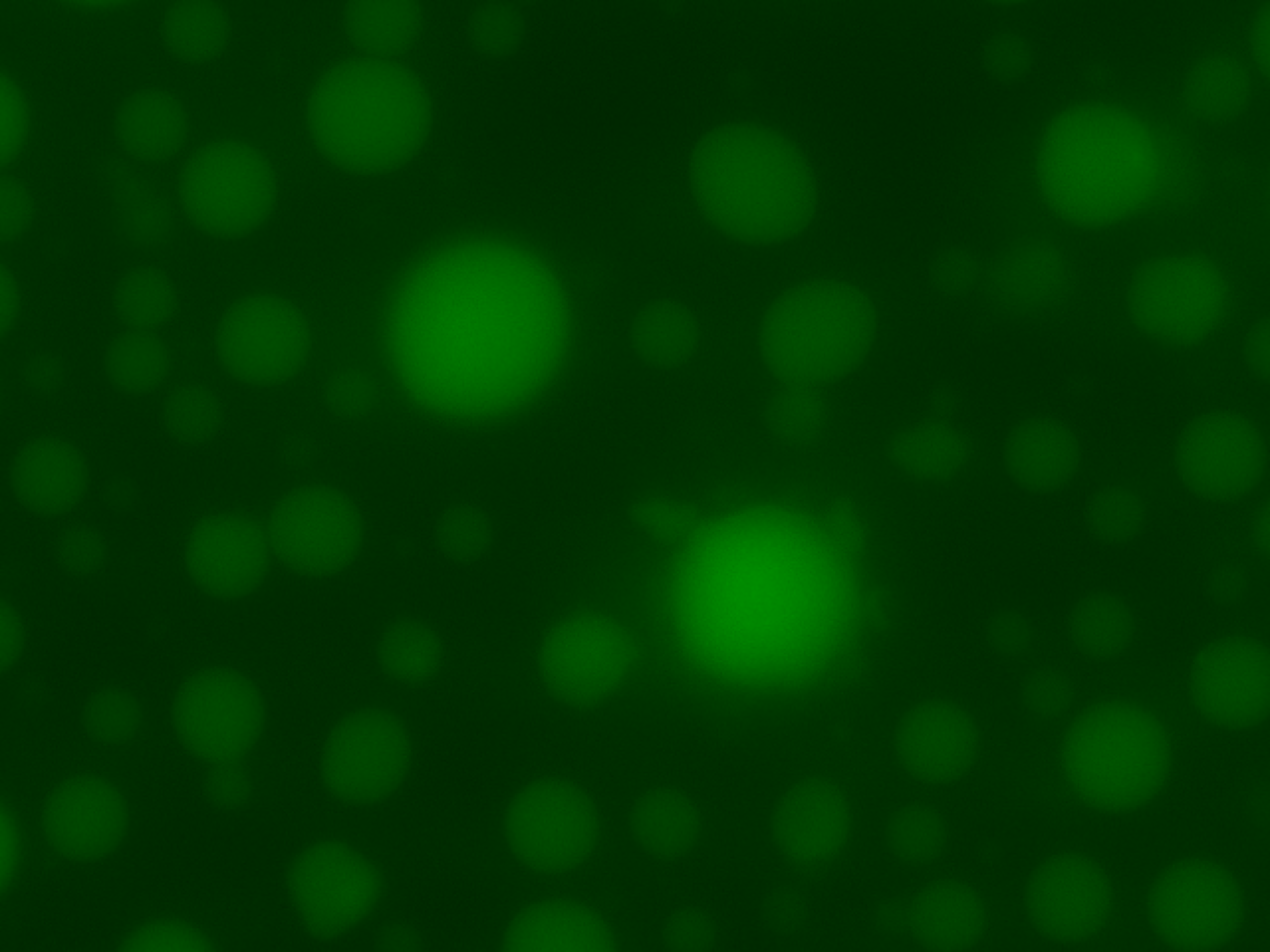

Supplement: Supplementary file 6 — Source data Fig. 4 [file 44318_2025_591_MOESM6_ESM.zip › Figure 4/4F/(a)14_72 h_SO286(7 ╬╝M).tif]

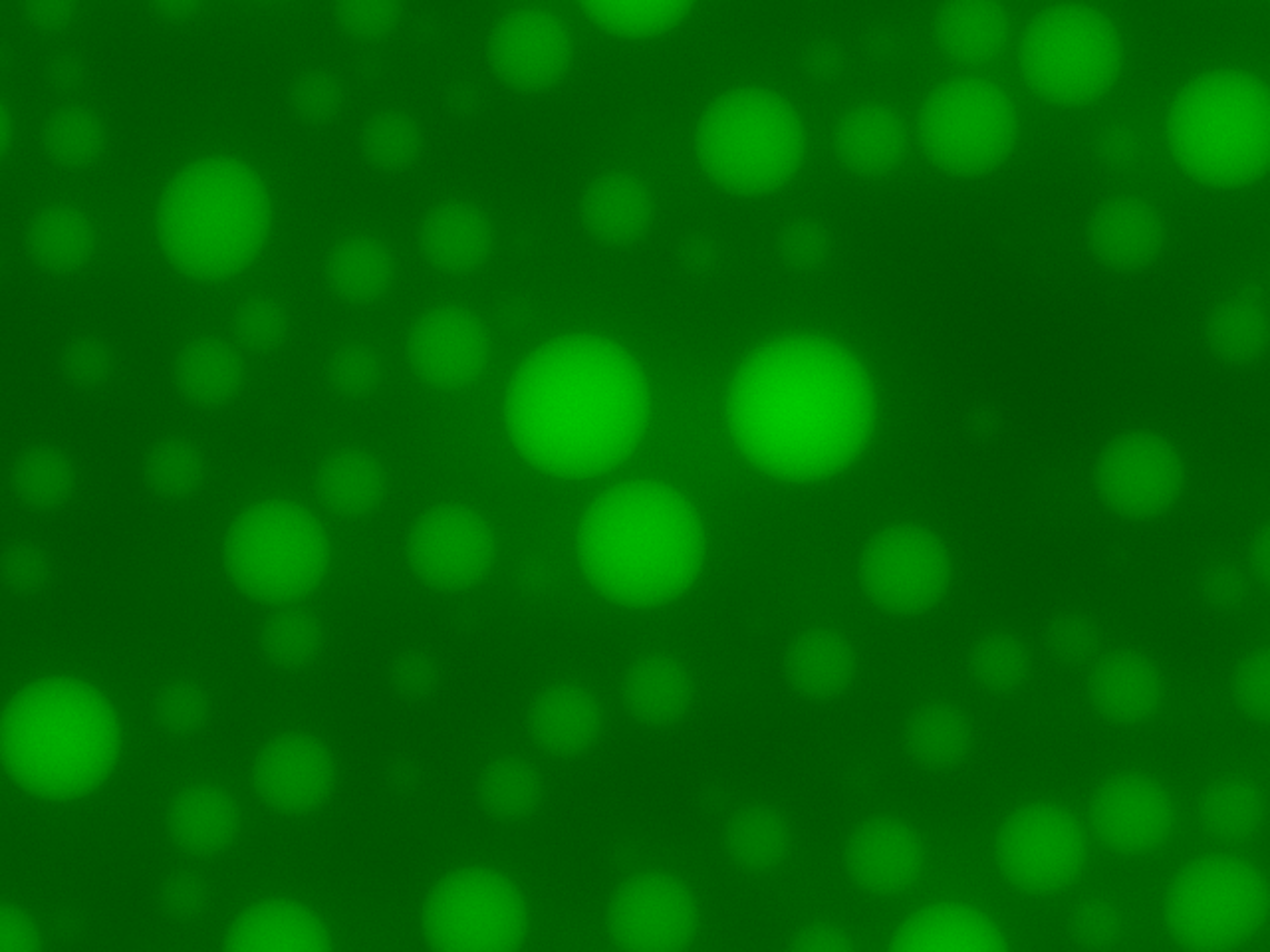

Supplement: Supplementary file 6 — Source data Fig. 4 [file 44318_2025_591_MOESM6_ESM.zip › Figure 4/4F/(a)13_48 h_SO286(7 ╬╝M).tif]

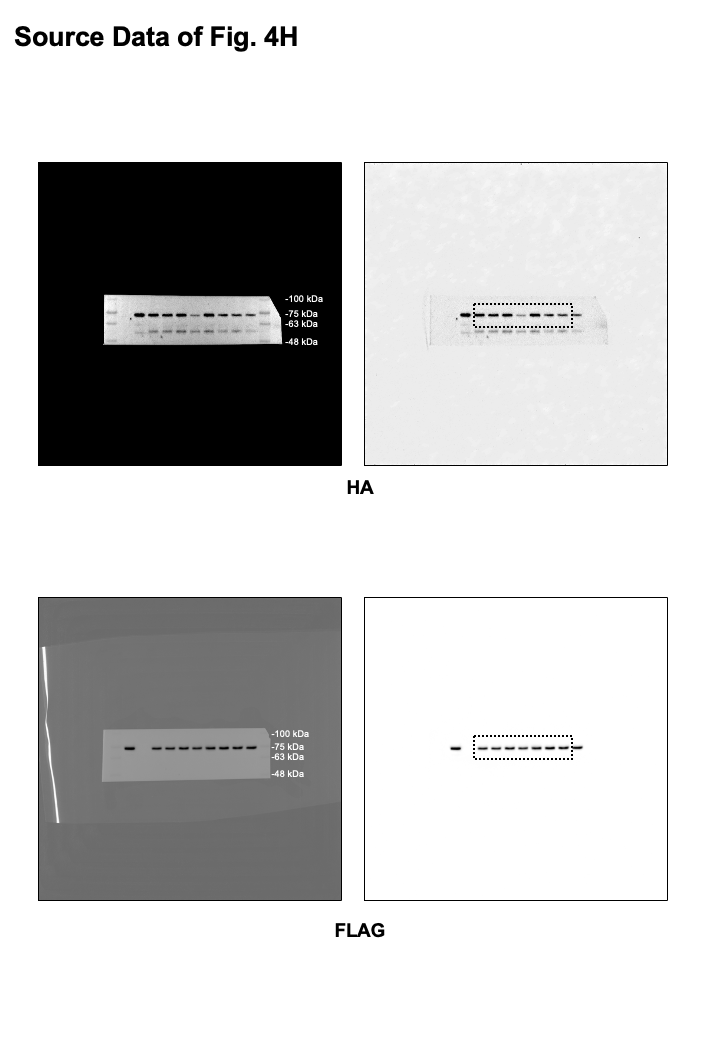

Supplement: Supplementary file 6 — Source data Fig. 4 [file 44318_2025_591_MOESM6_ESM.zip › Figure 4/4H/(a)_Highlight of crop area.tiff]

Source Data of Fig. 5A

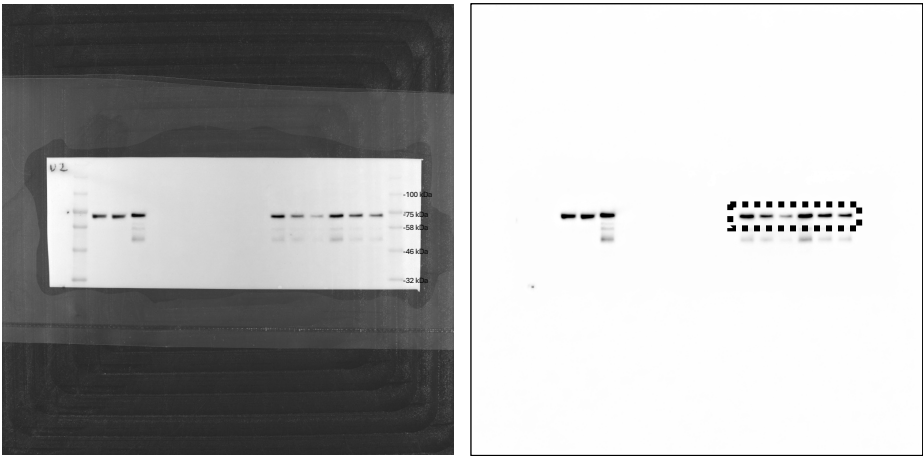

anti-UBQLN2

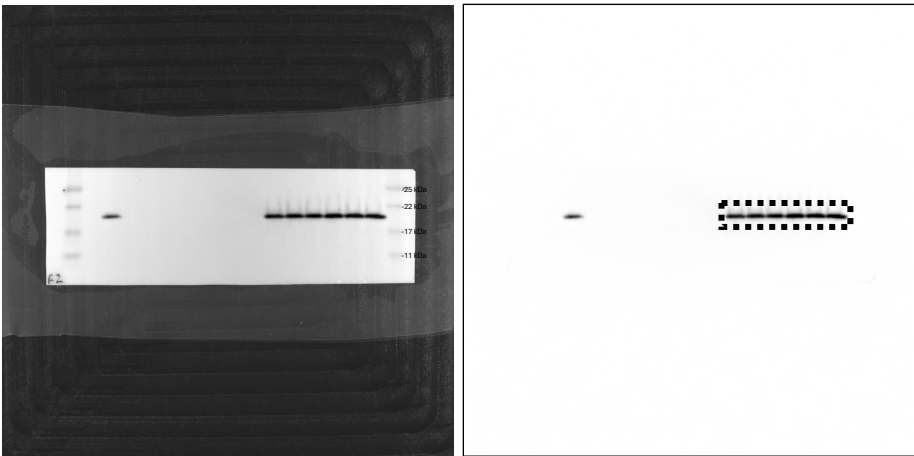

anti-FLAG

Supplement: Supplementary file 7 — Source data Fig. 5 [file 44318_2025_591_MOESM7_ESM.zip › Figure 5/5A/(a)_03_Highlight of crop area.pdf]

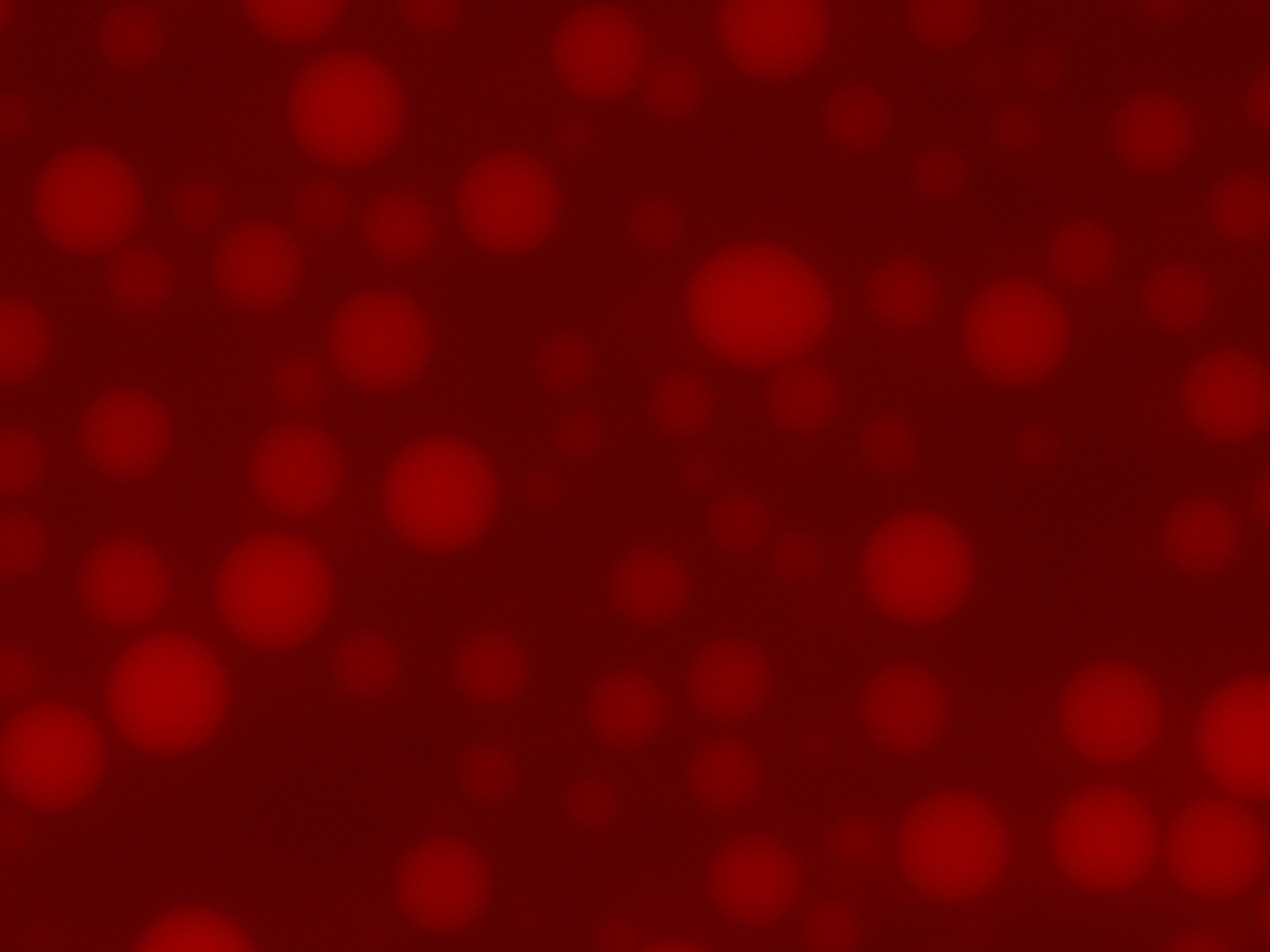

Supplement: Supplementary file 7 — Source data Fig. 5 [file 44318_2025_591_MOESM7_ESM.zip › Figure 5/5E/10_24 h_SO286_╬▒-Syn.tif]
