# Supplementary material for: Ubiquilin-2 liquid droplets catalyze α-synuclein fibril formation
Source: EMBO J. 2025 Oct 14;44(22):6527–55. doi: 10.1038/s44318-025-00591-1 (PMC12623503; doi:10.1038/s44318-025-00591-1)
Supplement: Supplementary file 5 — Source data Fig. 3 [file 44318_2025_591_MOESM5_ESM.zip › Figure 3/3D/07_Highlight of corp area.pdf]

Source Data of Fig. 3D

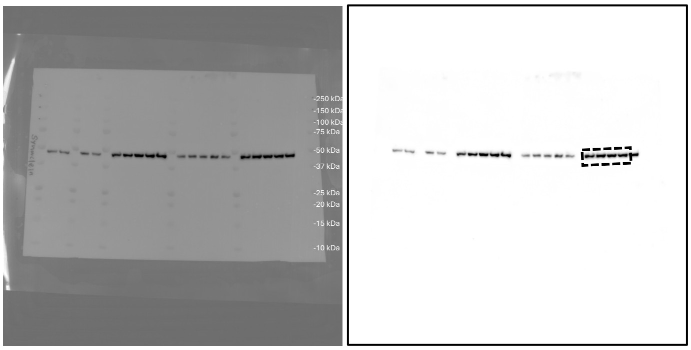

α-Syn (soluble fraction)

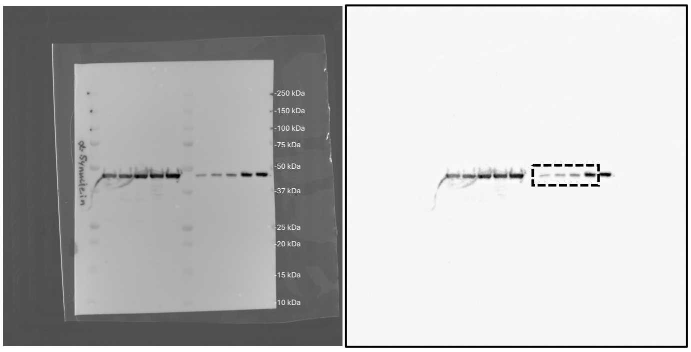

α-Syn (insoluble fraction)

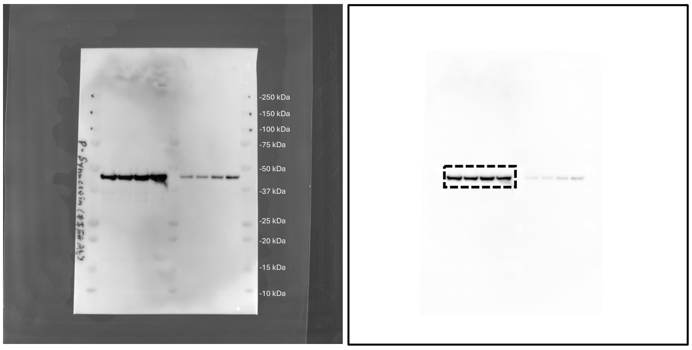

phospho-synuclein
(soluble fraction)

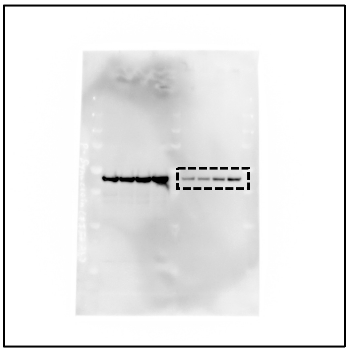

phospho-synuclein
(insoluble fraction)

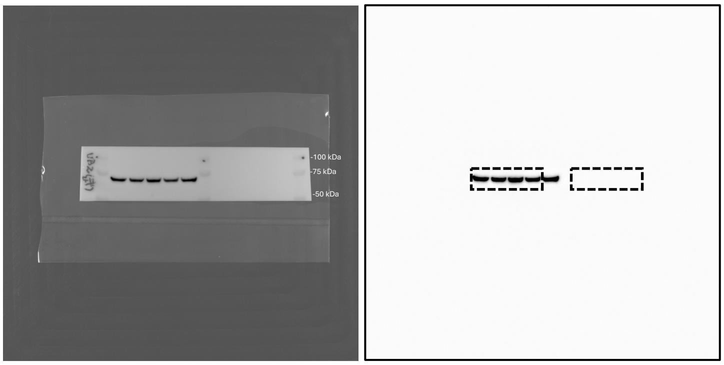

UBQLN2 (Left, soluble; right, insoluble)

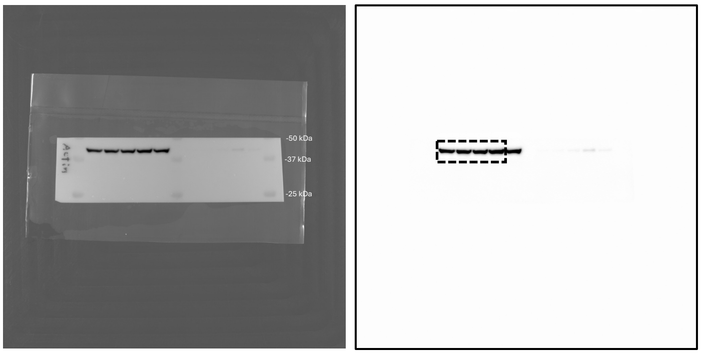

β-actin (soluble fraction)
