# Supplementary figures and images for: Ubiquilin-2 liquid droplets catalyze α-synuclein fibril formation (part 2 of 3)
Source: EMBO J. 2025 Oct 14;44(22):6527–55. doi: 10.1038/s44318-025-00591-1 (PMC12623503; doi:10.1038/s44318-025-00591-1)

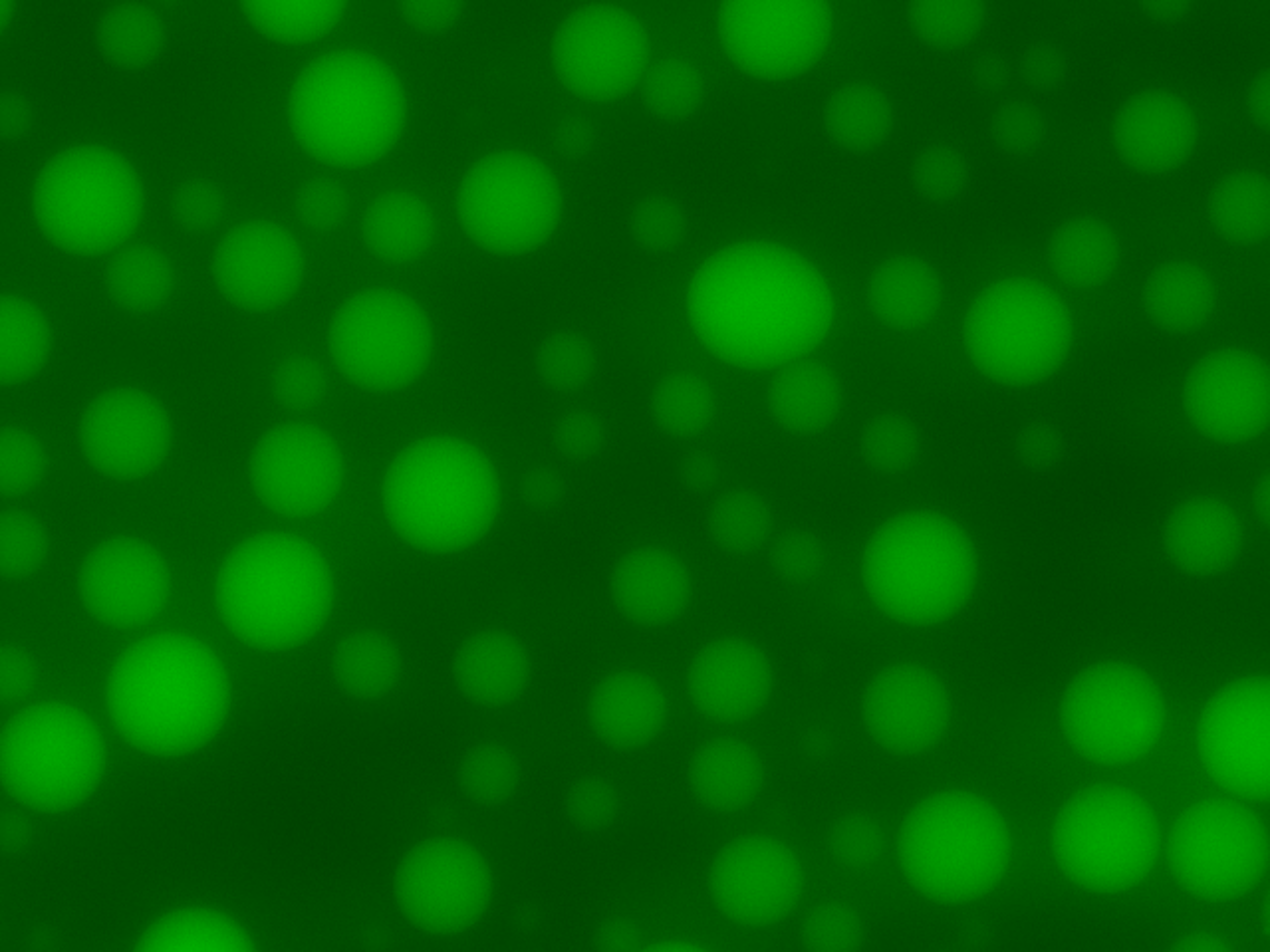

Supplement: Supplementary file 7 — Source data Fig. 5 [file 44318_2025_591_MOESM7_ESM.zip › Figure 5/5E/09_24 h_SO286_UBQLN2.tif]

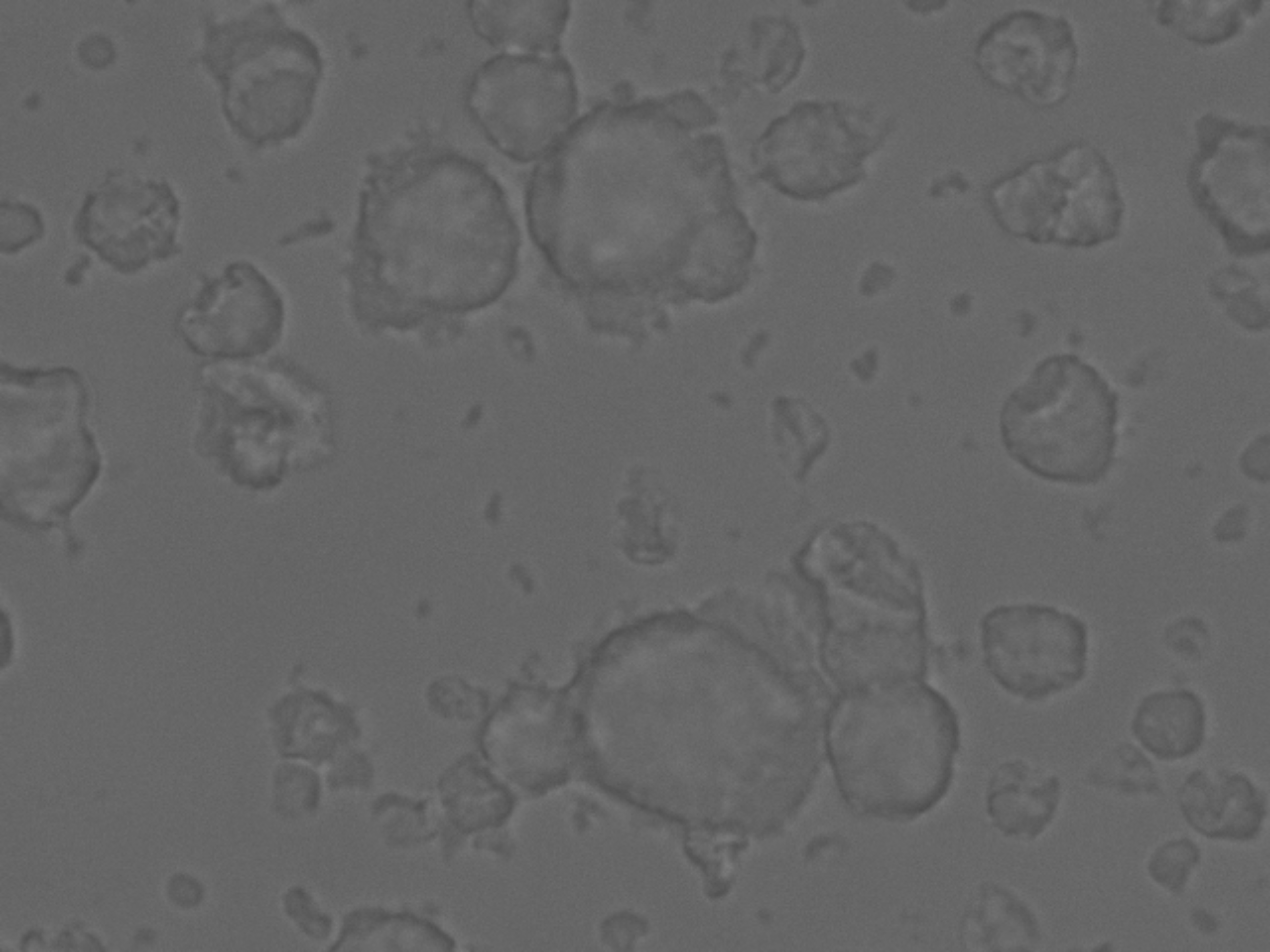

Supplement: Supplementary file 7 — Source data Fig. 5 [file 44318_2025_591_MOESM7_ESM.zip › Figure 5/5E/07_96 h_Control_Bright field.tif]

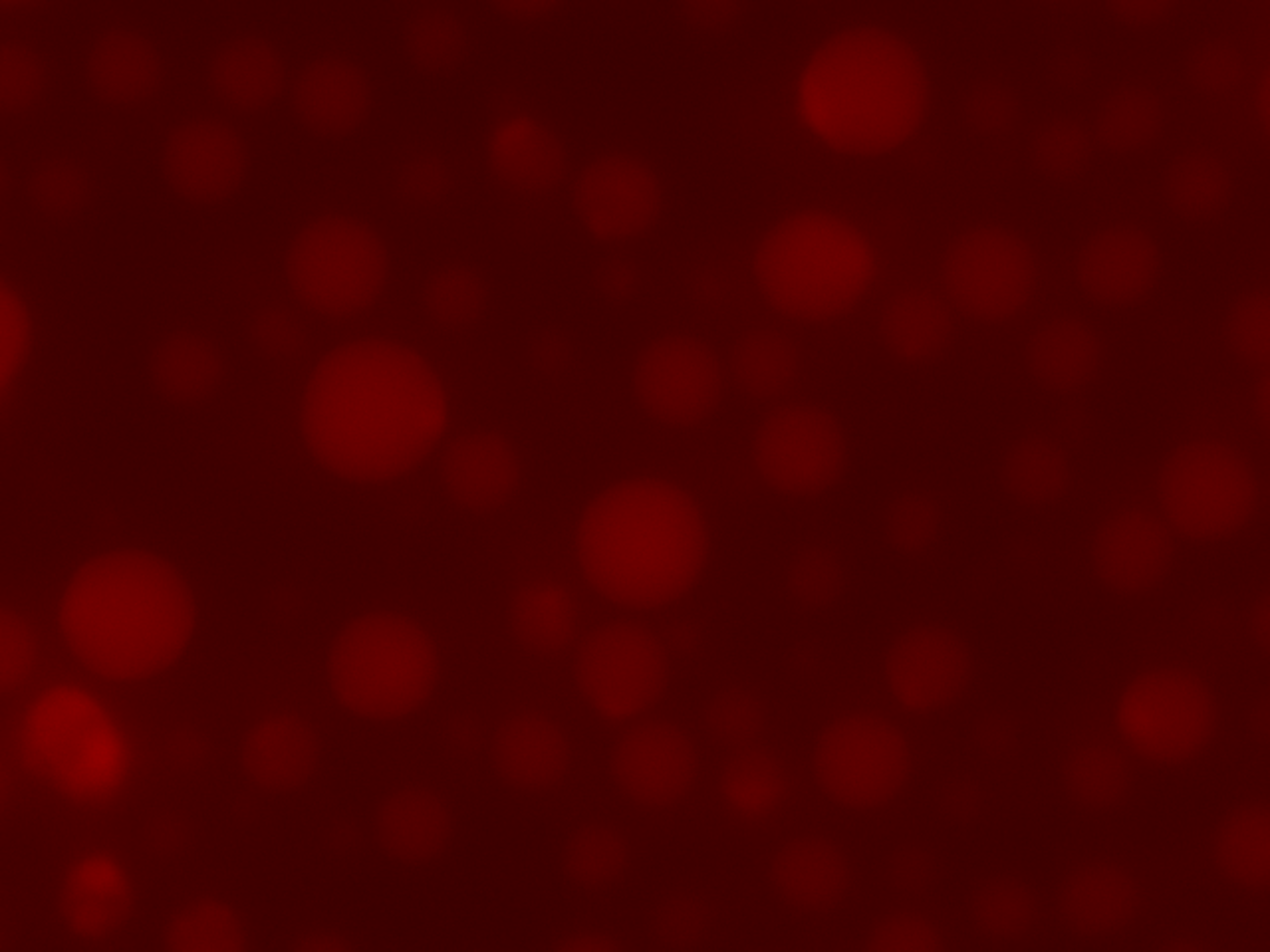

Supplement: Supplementary file 7 — Source data Fig. 5 [file 44318_2025_591_MOESM7_ESM.zip › Figure 5/5E/14_96 h_SO286_╬▒-Syn.tif]

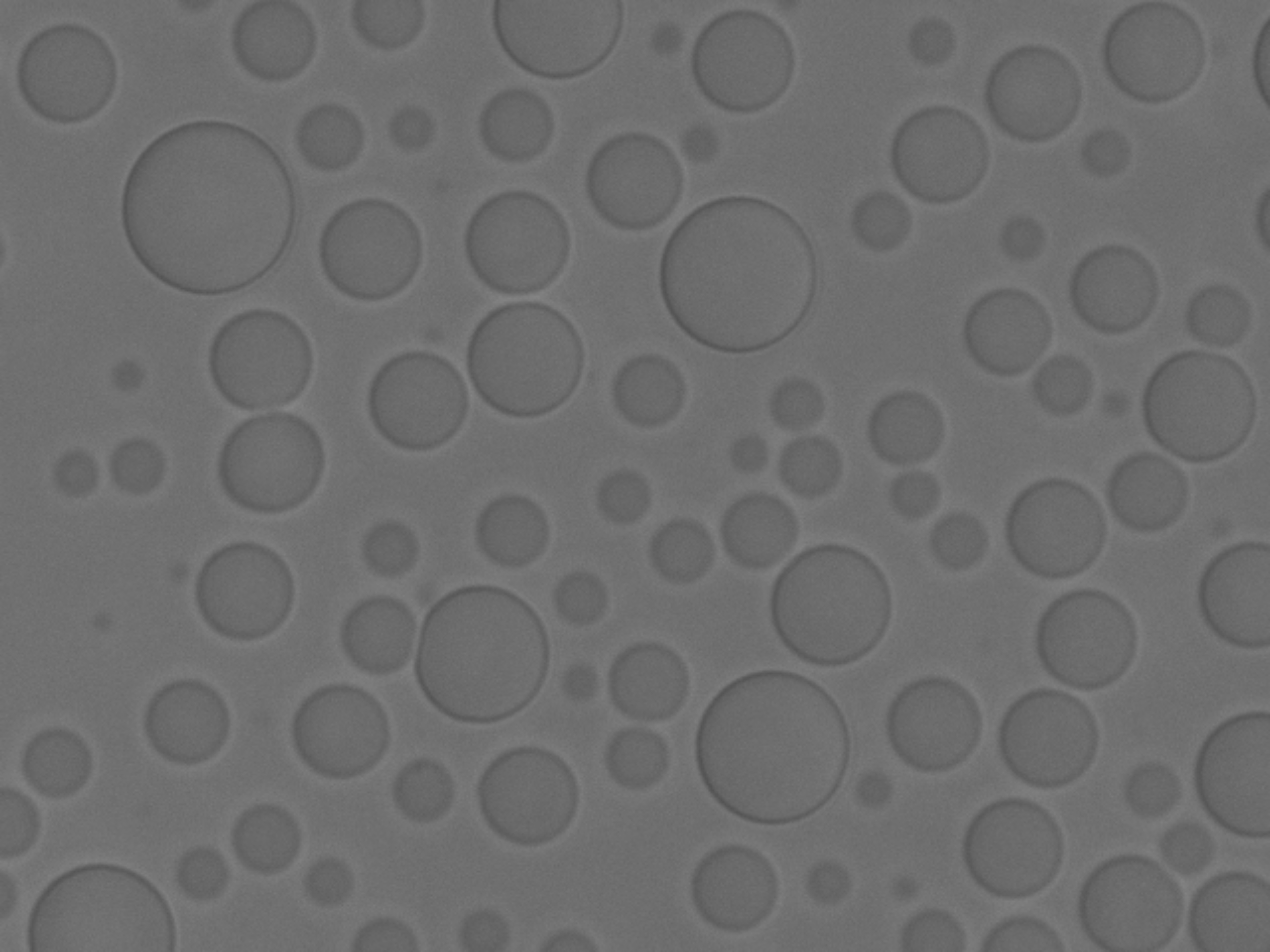

Supplement: Supplementary file 7 — Source data Fig. 5 [file 44318_2025_591_MOESM7_ESM.zip › Figure 5/5E/03_24 h_Control_Bright field.tif]

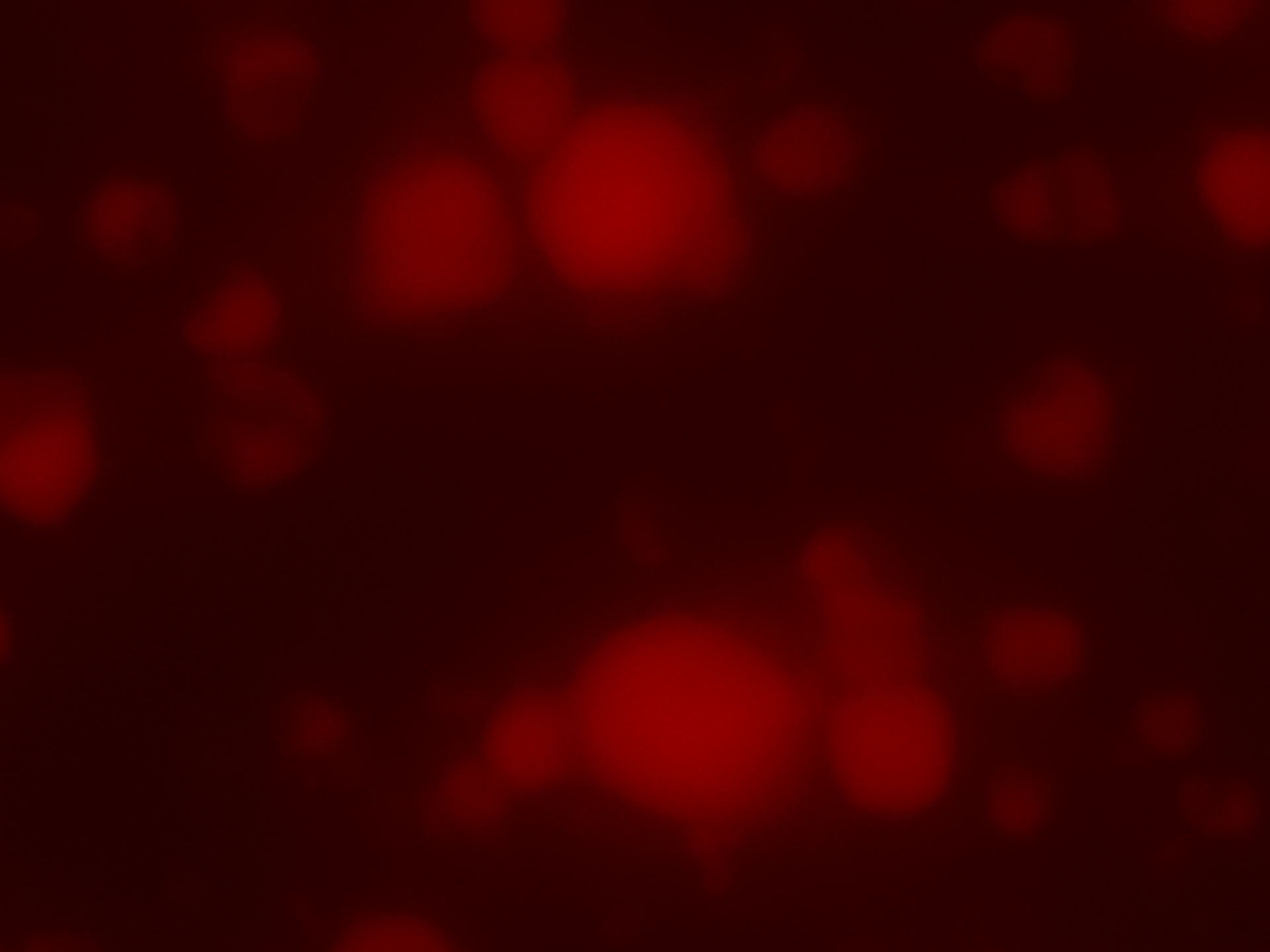

Supplement: Supplementary file 7 — Source data Fig. 5 [file 44318_2025_591_MOESM7_ESM.zip › Figure 5/5E/06_96 h_Control_╬▒-Syn.tif]

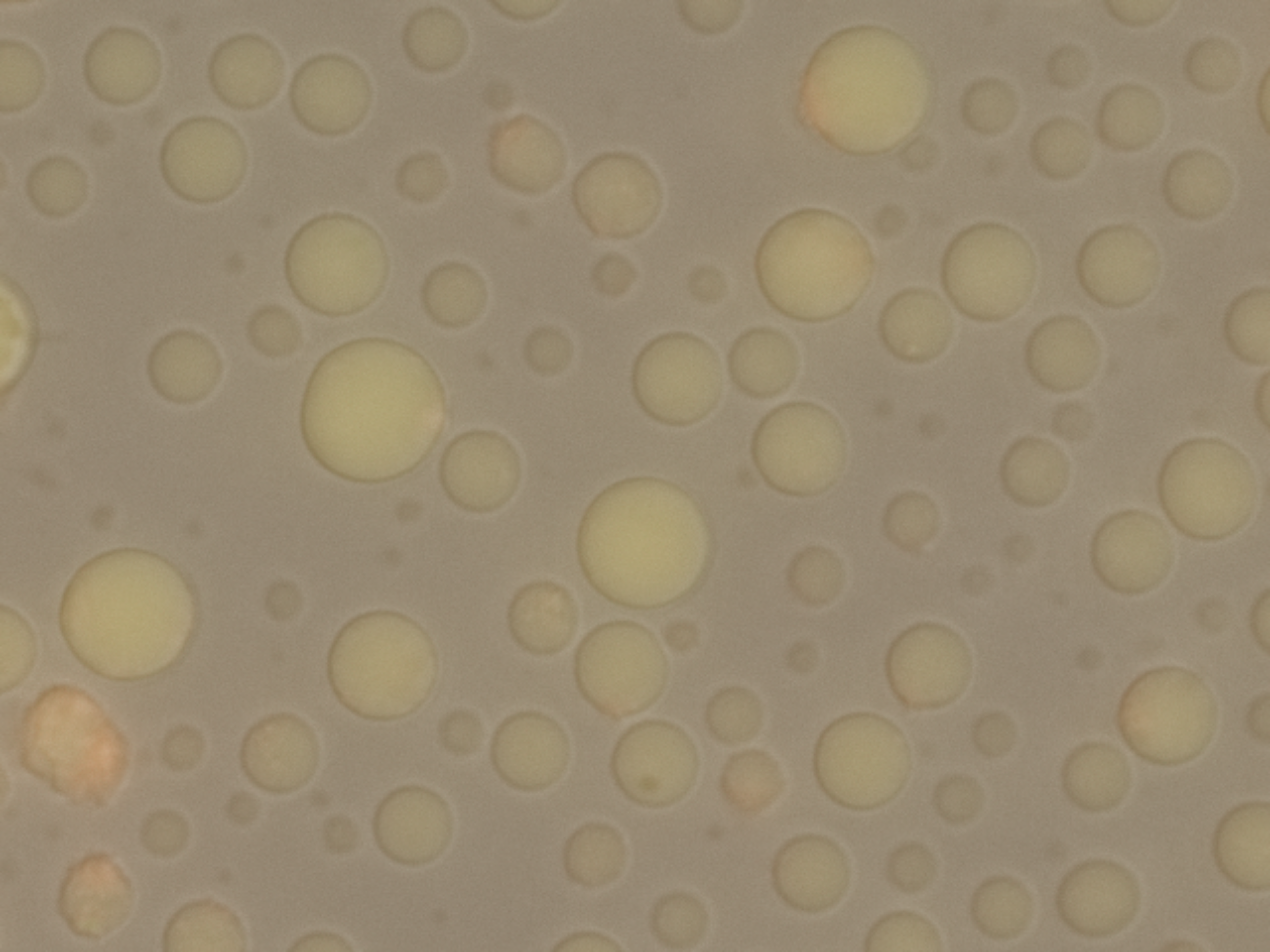

Supplement: Supplementary file 7 — Source data Fig. 5 [file 44318_2025_591_MOESM7_ESM.zip › Figure 5/5E/16_96 h_SO286_Merge.tif]

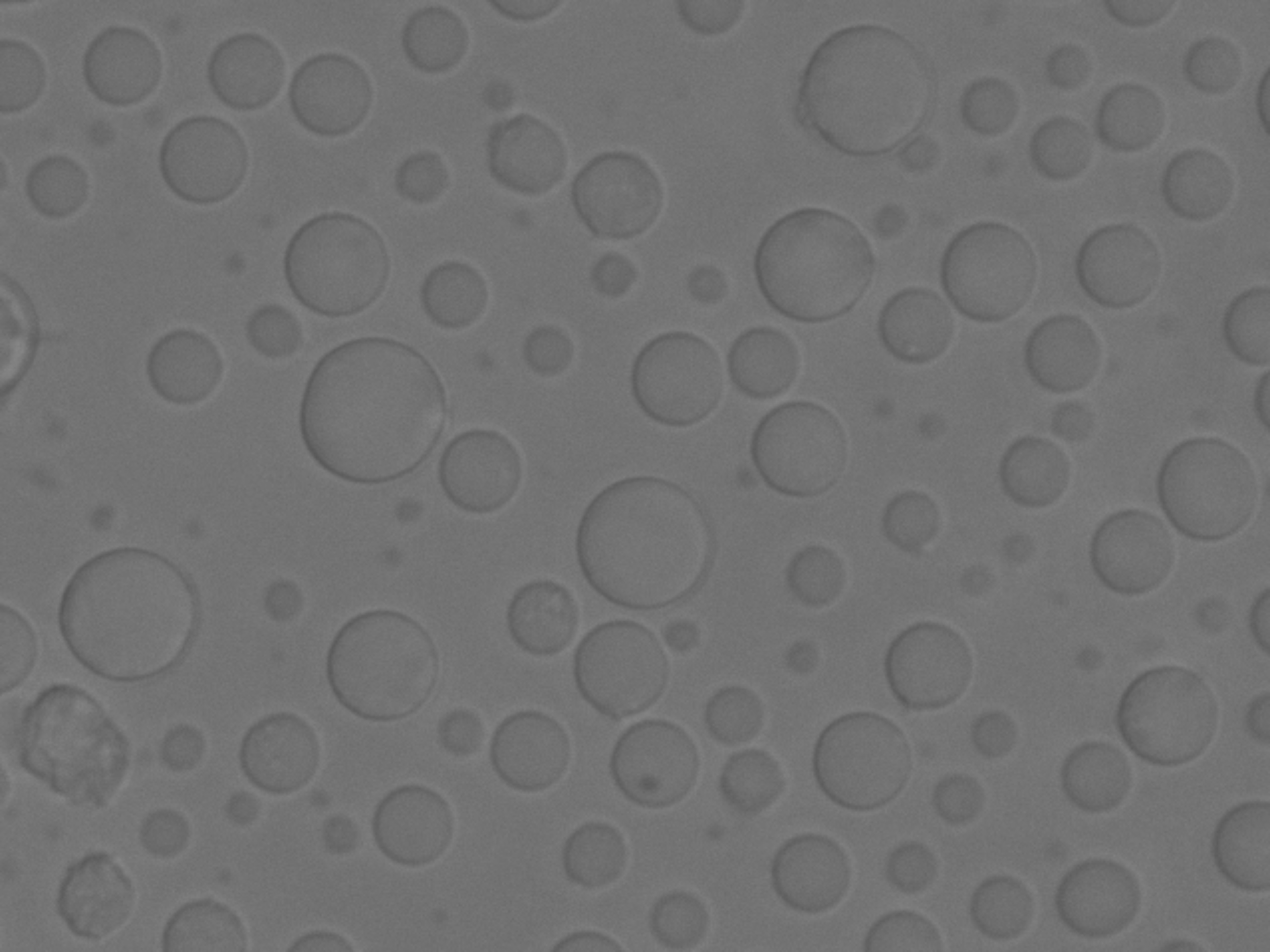

Supplement: Supplementary file 7 — Source data Fig. 5 [file 44318_2025_591_MOESM7_ESM.zip › Figure 5/5E/15_96 h_SO286_Bright field.tif]

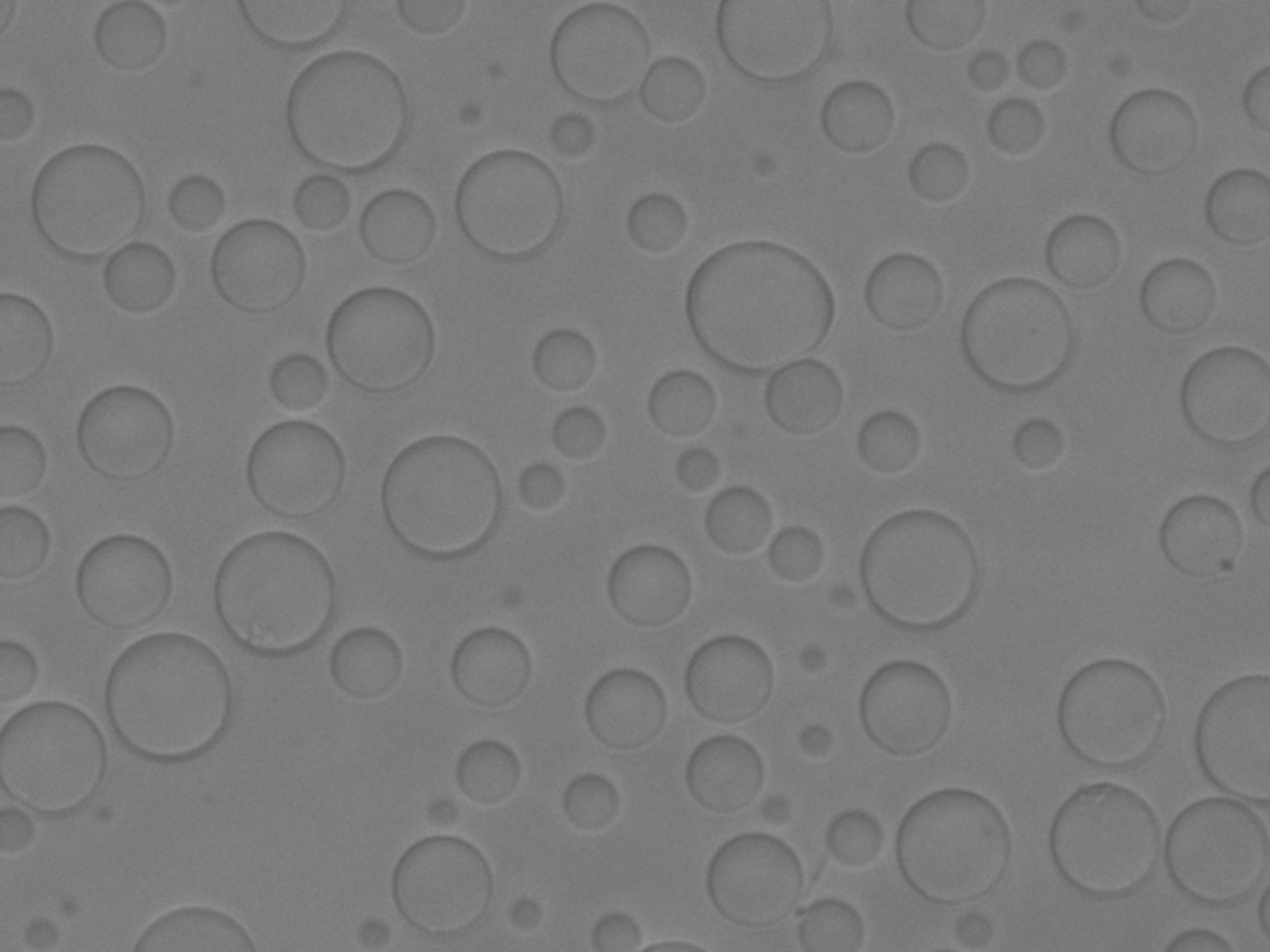

Supplement: Supplementary file 7 — Source data Fig. 5 [file 44318_2025_591_MOESM7_ESM.zip › Figure 5/5E/11_24 h_SO286_Bright field.tif]

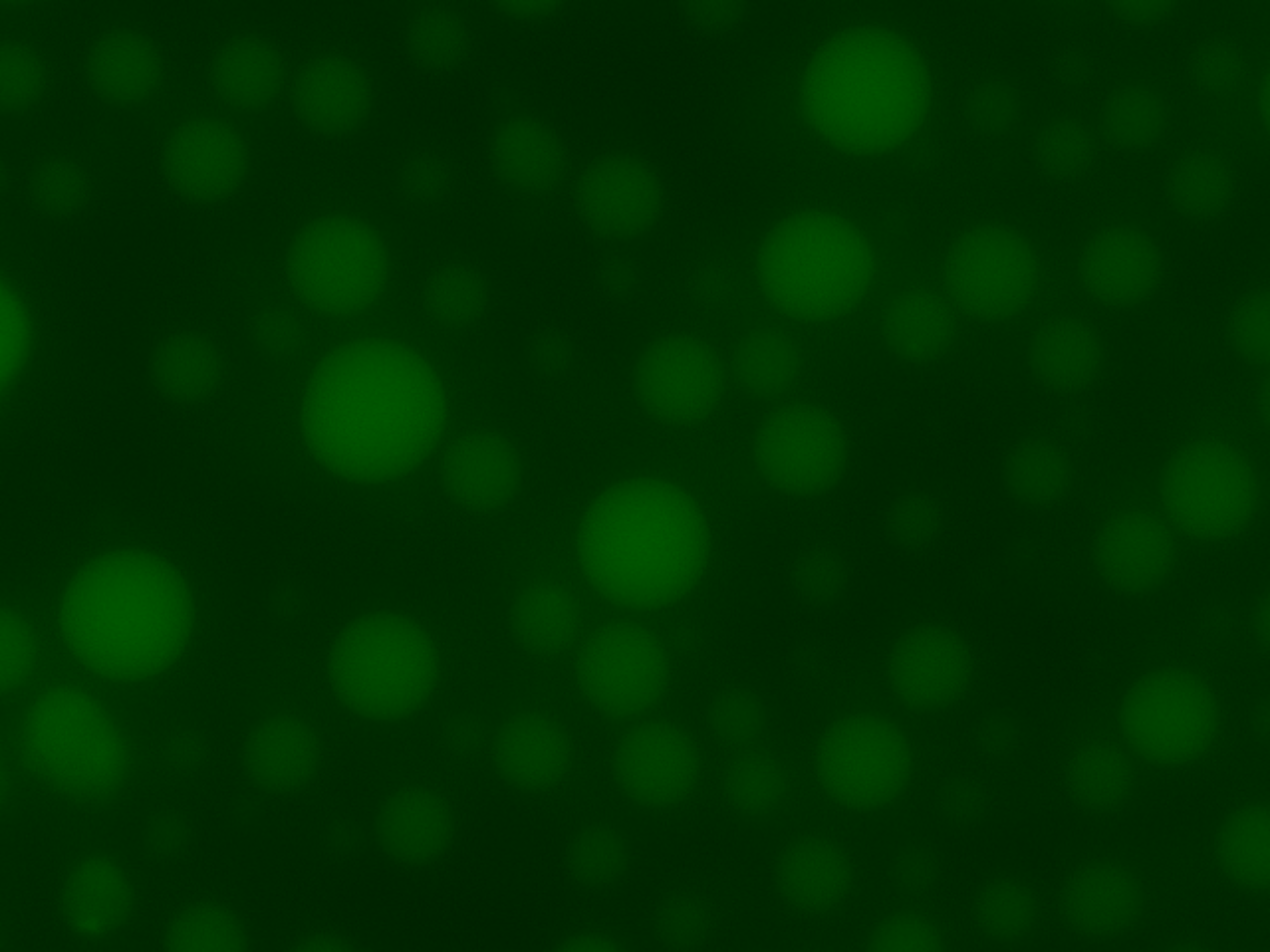

Supplement: Supplementary file 7 — Source data Fig. 5 [file 44318_2025_591_MOESM7_ESM.zip › Figure 5/5E/13_96 h_SO286_UBQLN2.tif]

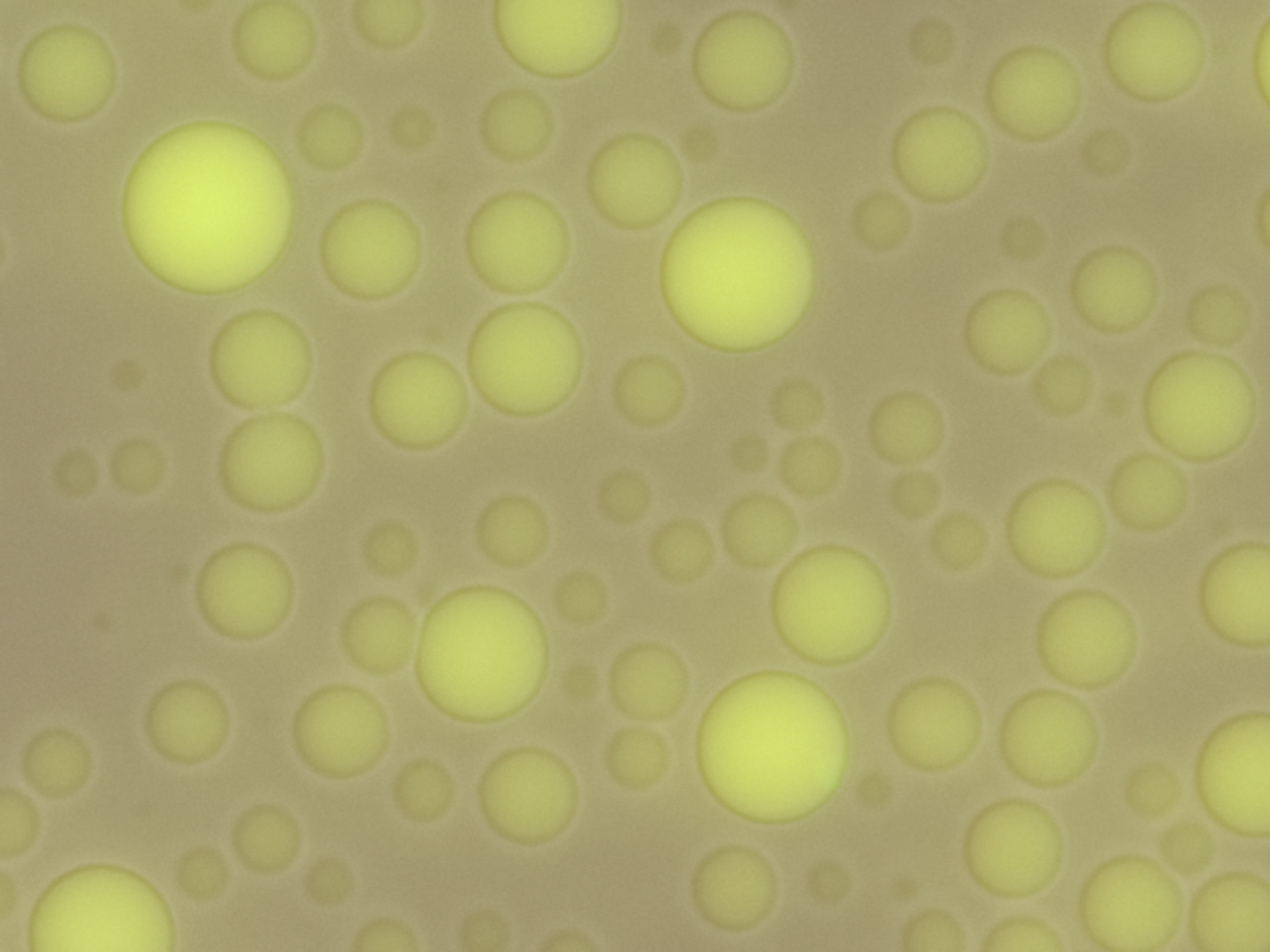

Supplement: Supplementary file 7 — Source data Fig. 5 [file 44318_2025_591_MOESM7_ESM.zip › Figure 5/5E/04_24 h_Control_Merge.tif]

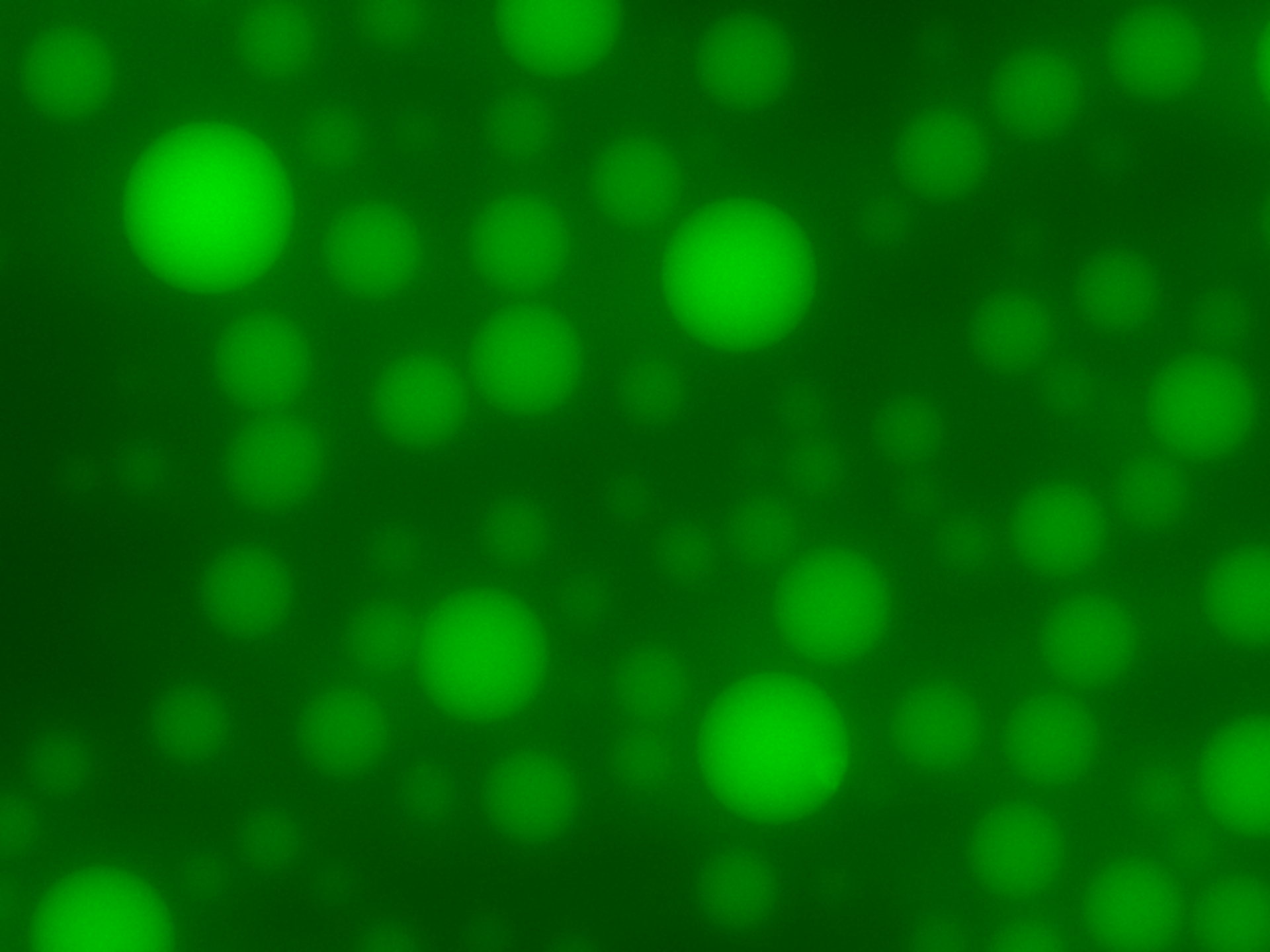

Supplement: Supplementary file 7 — Source data Fig. 5 [file 44318_2025_591_MOESM7_ESM.zip › Figure 5/5E/01_24 h_Control_UBQLN2.tif]

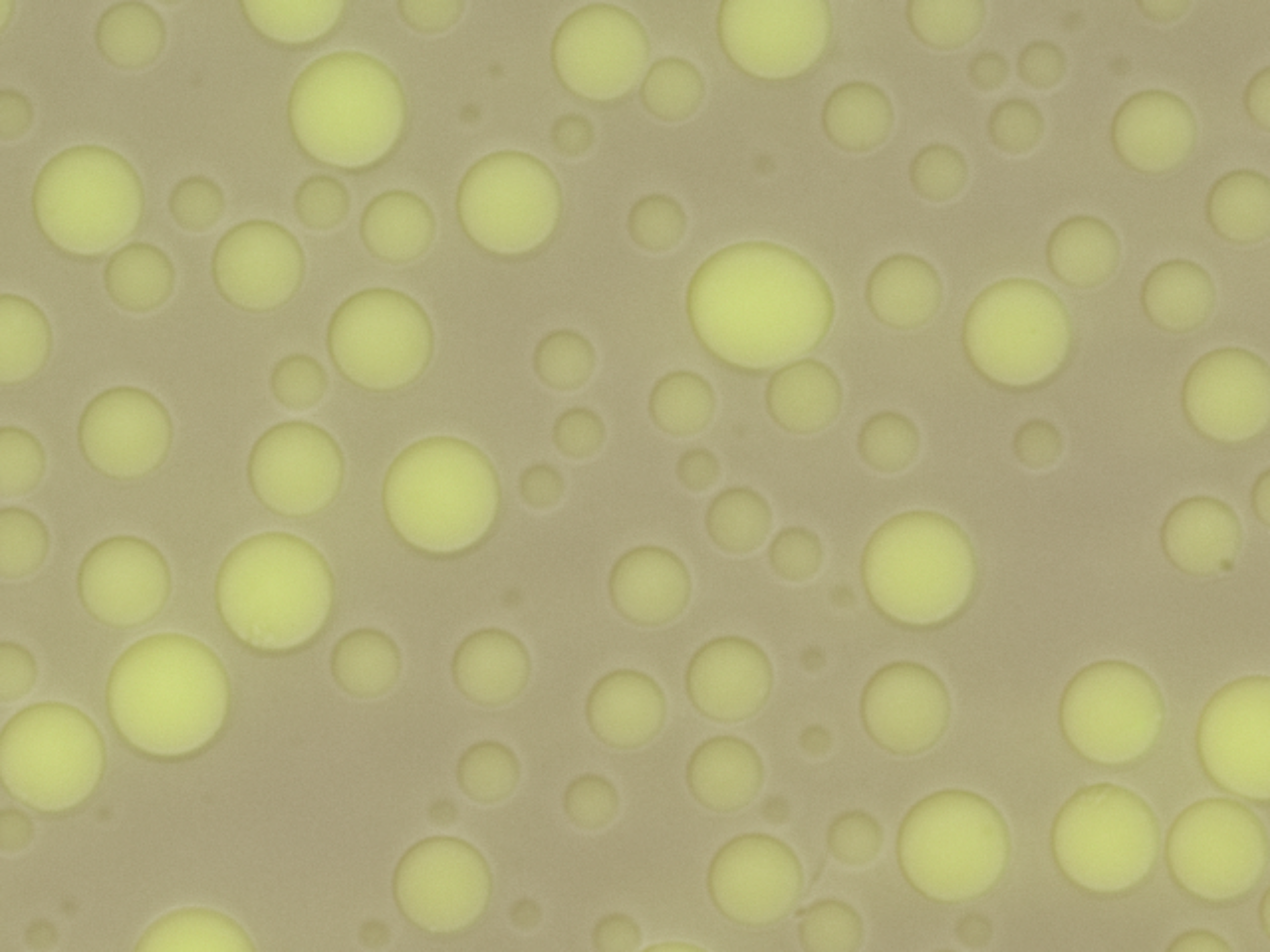

Supplement: Supplementary file 7 — Source data Fig. 5 [file 44318_2025_591_MOESM7_ESM.zip › Figure 5/5E/12_24 h_SO286_Merge.tif]

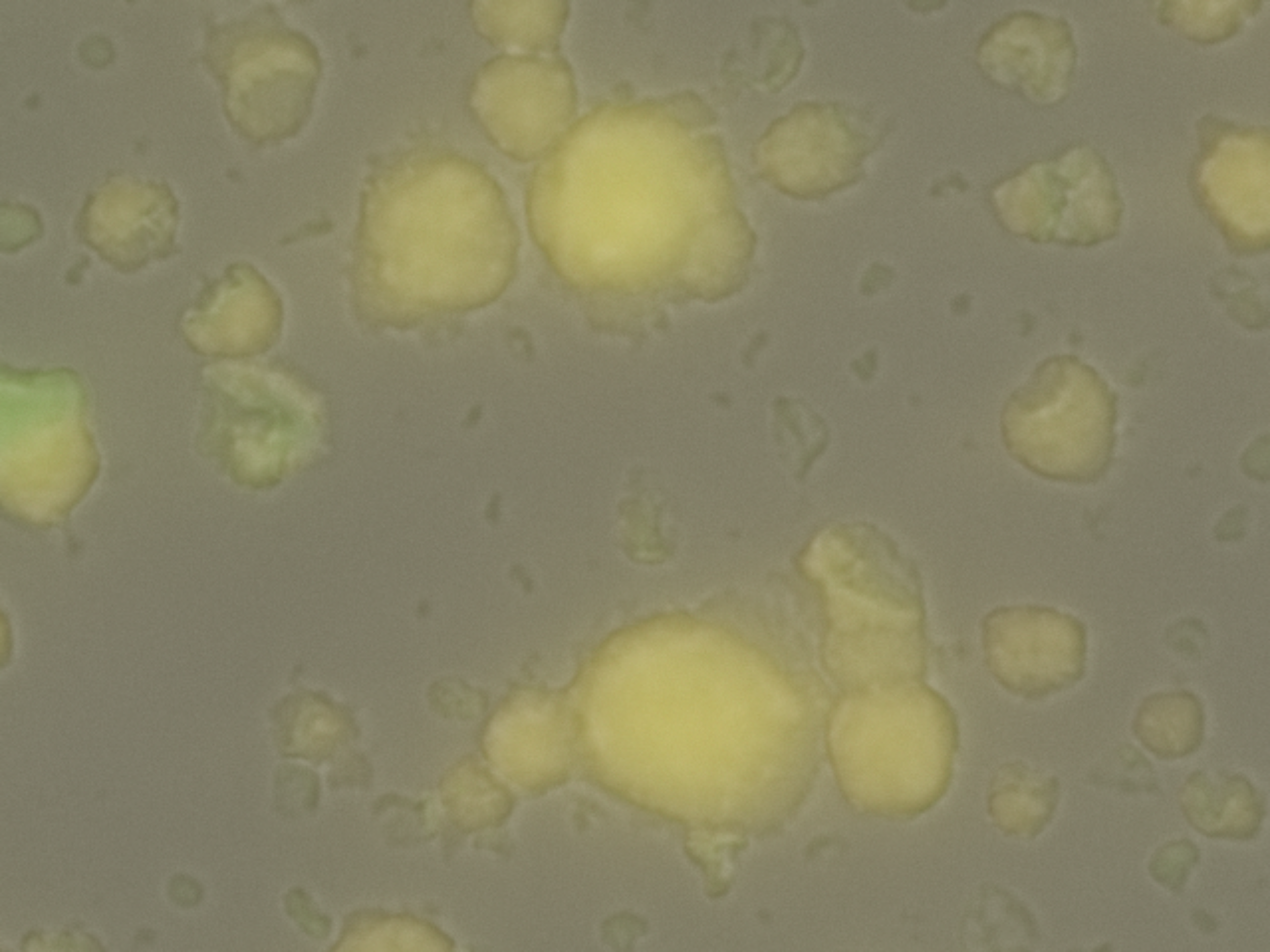

Supplement: Supplementary file 7 — Source data Fig. 5 [file 44318_2025_591_MOESM7_ESM.zip › Figure 5/5E/08_96 h_Control_Merge.tif]

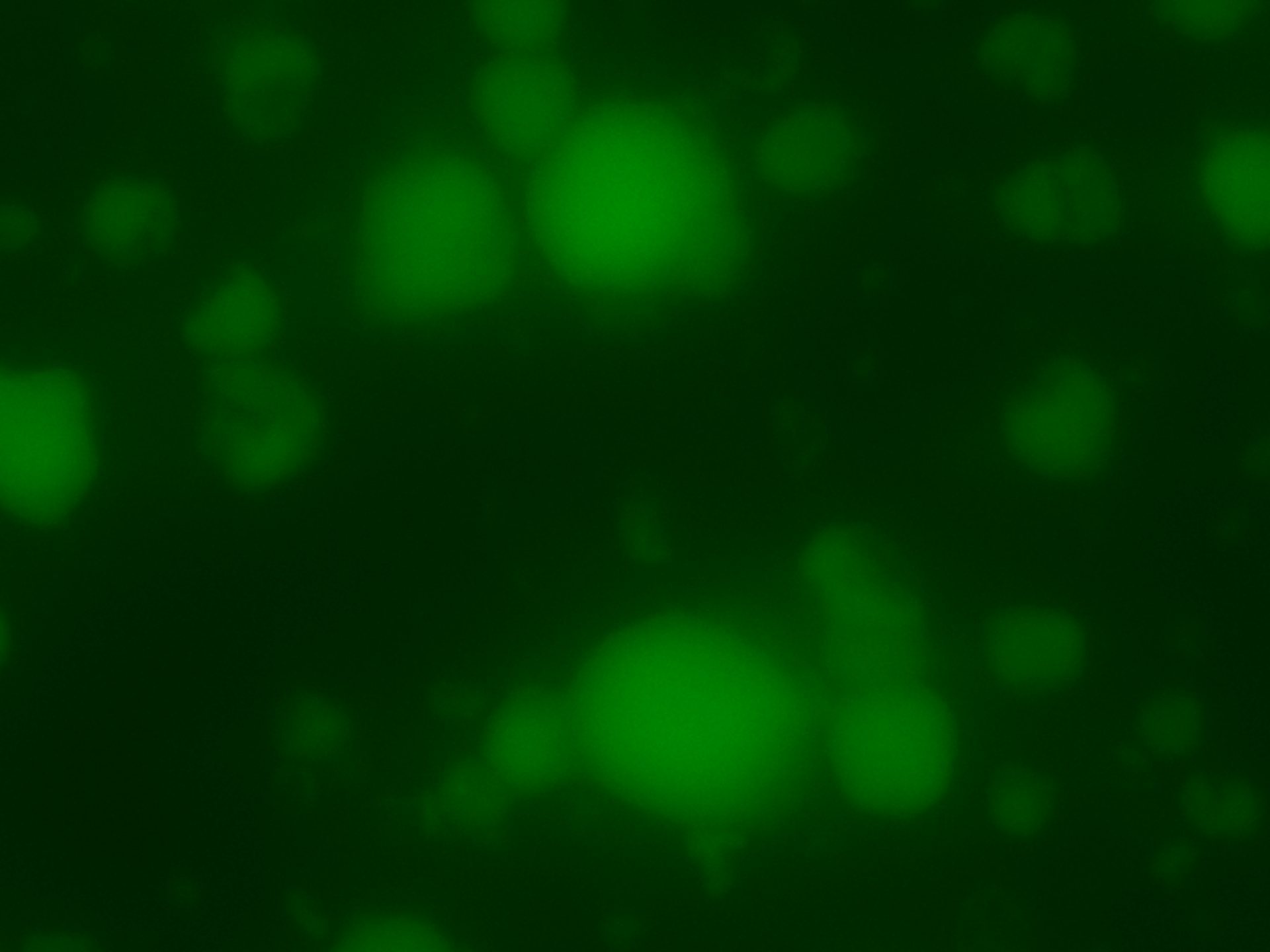

Supplement: Supplementary file 7 — Source data Fig. 5 [file 44318_2025_591_MOESM7_ESM.zip › Figure 5/5E/05_96 h_Control_UBQLN2.tif]

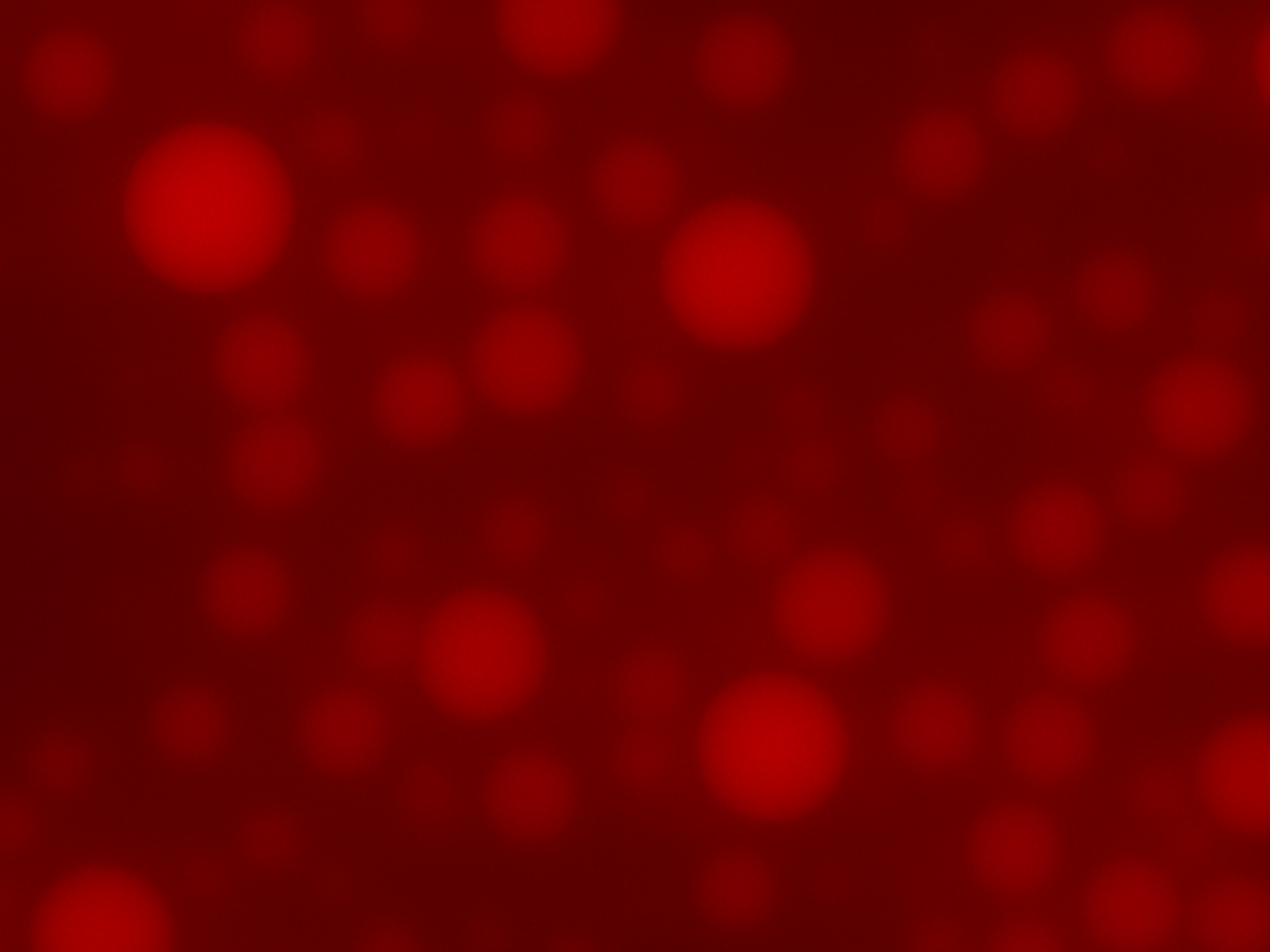

Supplement: Supplementary file 7 — Source data Fig. 5 [file 44318_2025_591_MOESM7_ESM.zip › Figure 5/5E/02_24 h_Control_╬▒-Syn.tif]

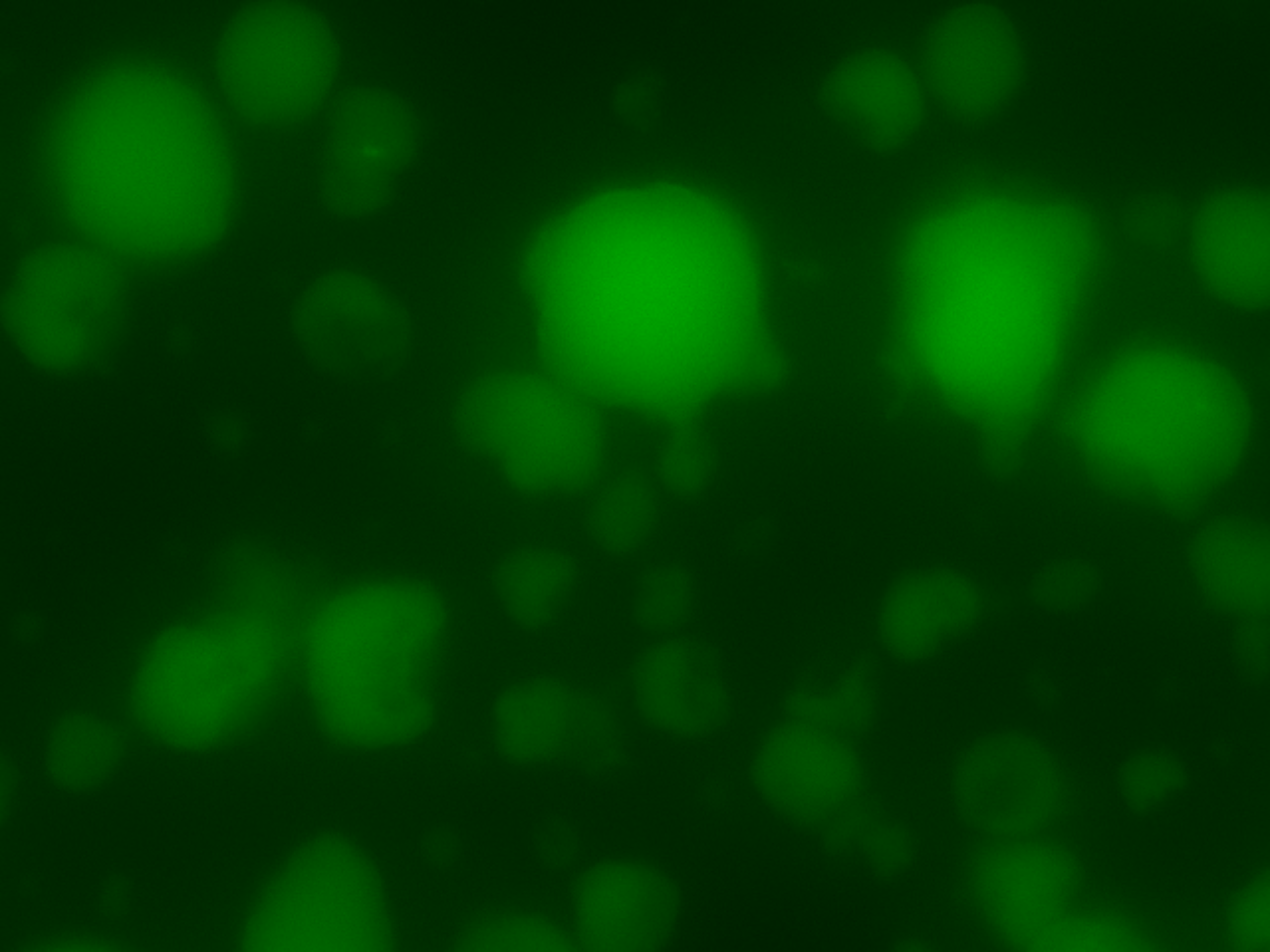

Supplement: Supplementary file 7 — Source data Fig. 5 [file 44318_2025_591_MOESM7_ESM.zip › Figure 5/5B/25_96 h_Control_UBQLN2.tif]

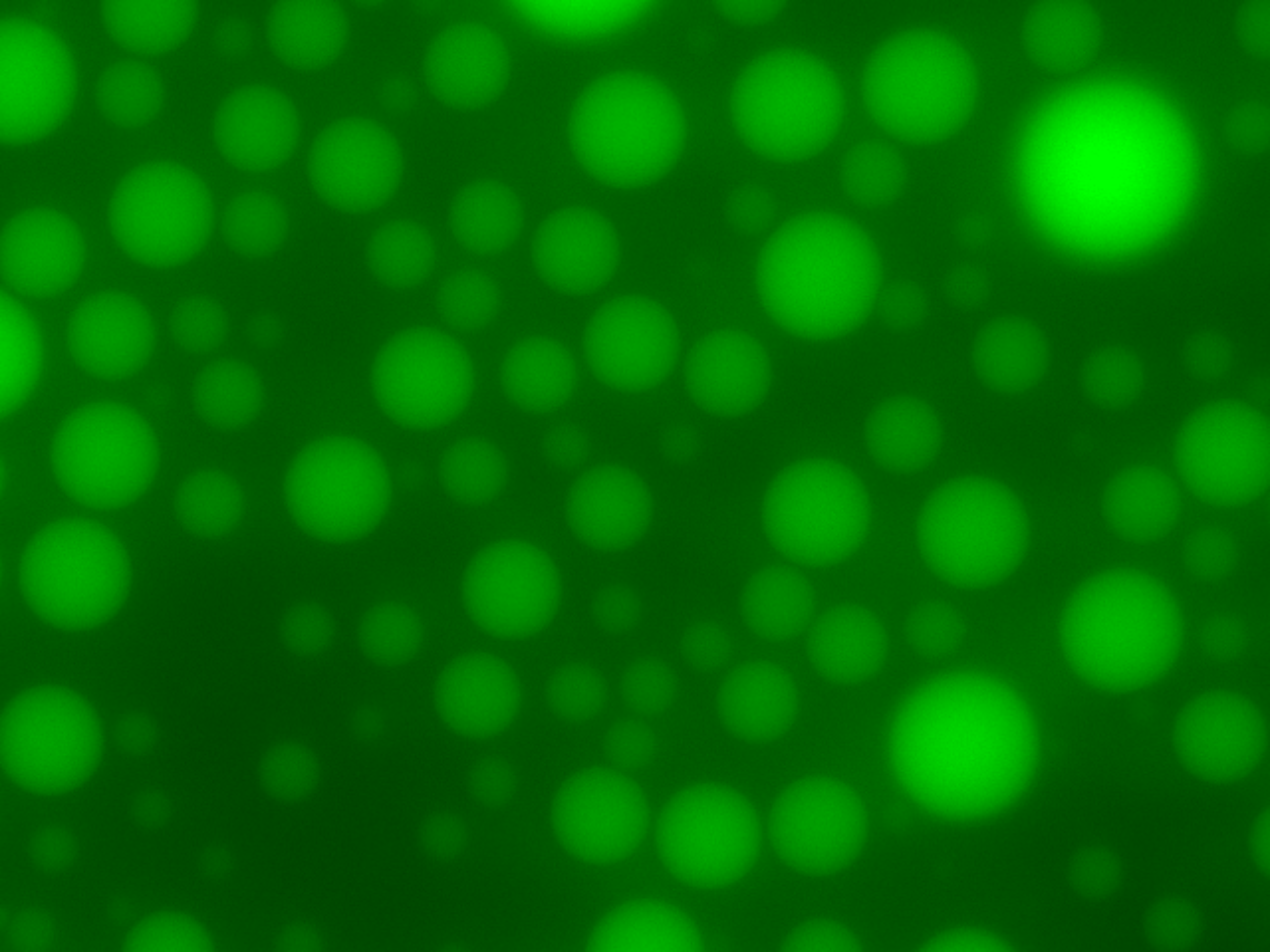

Supplement: Supplementary file 7 — Source data Fig. 5 [file 44318_2025_591_MOESM7_ESM.zip › Figure 5/5B/07_24 h_Control_UBQLN2.tif]

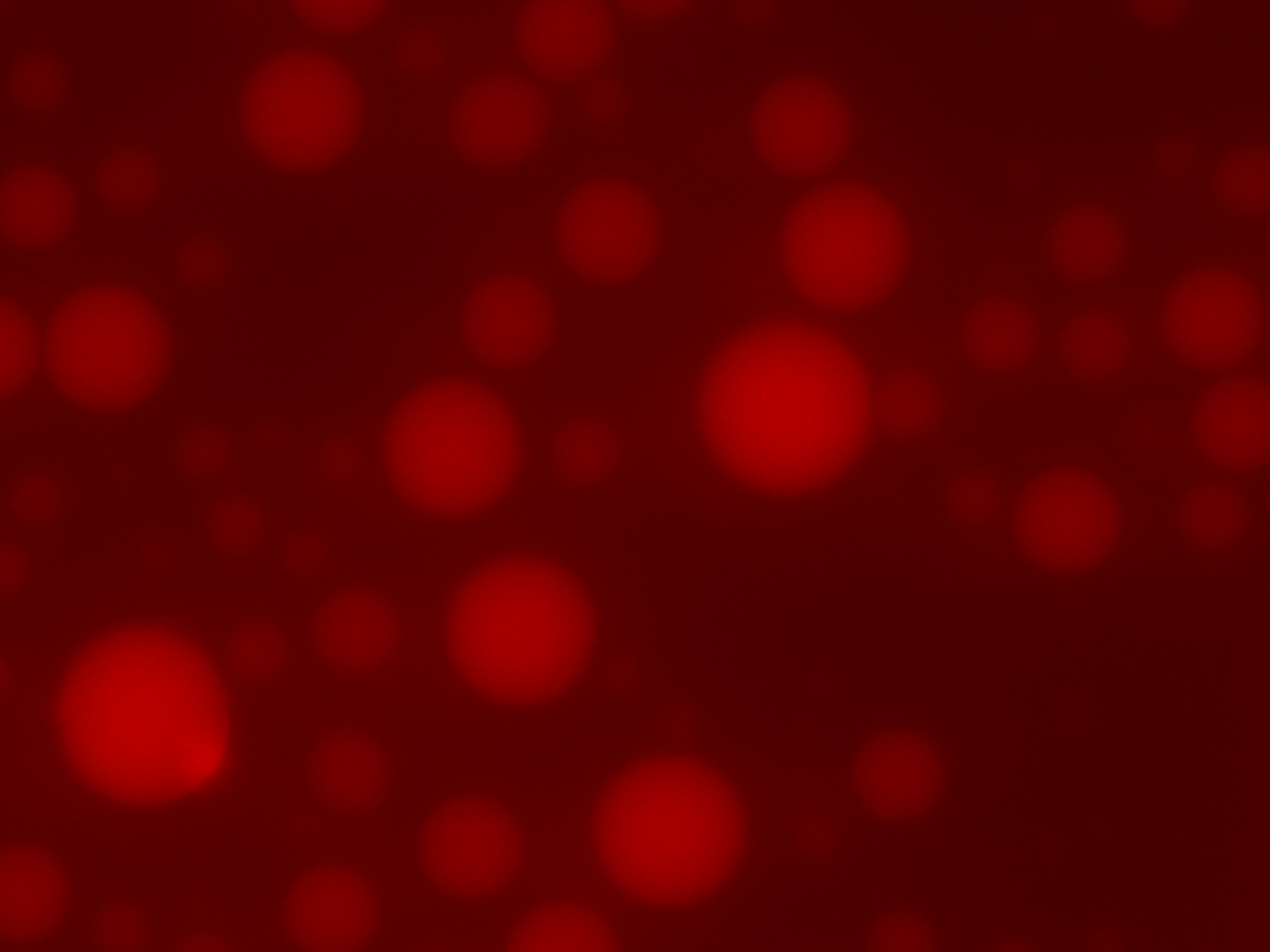

Supplement: Supplementary file 7 — Source data Fig. 5 [file 44318_2025_591_MOESM7_ESM.zip › Figure 5/5B/17_48 h_SO286_╬▒-Syn.tif]

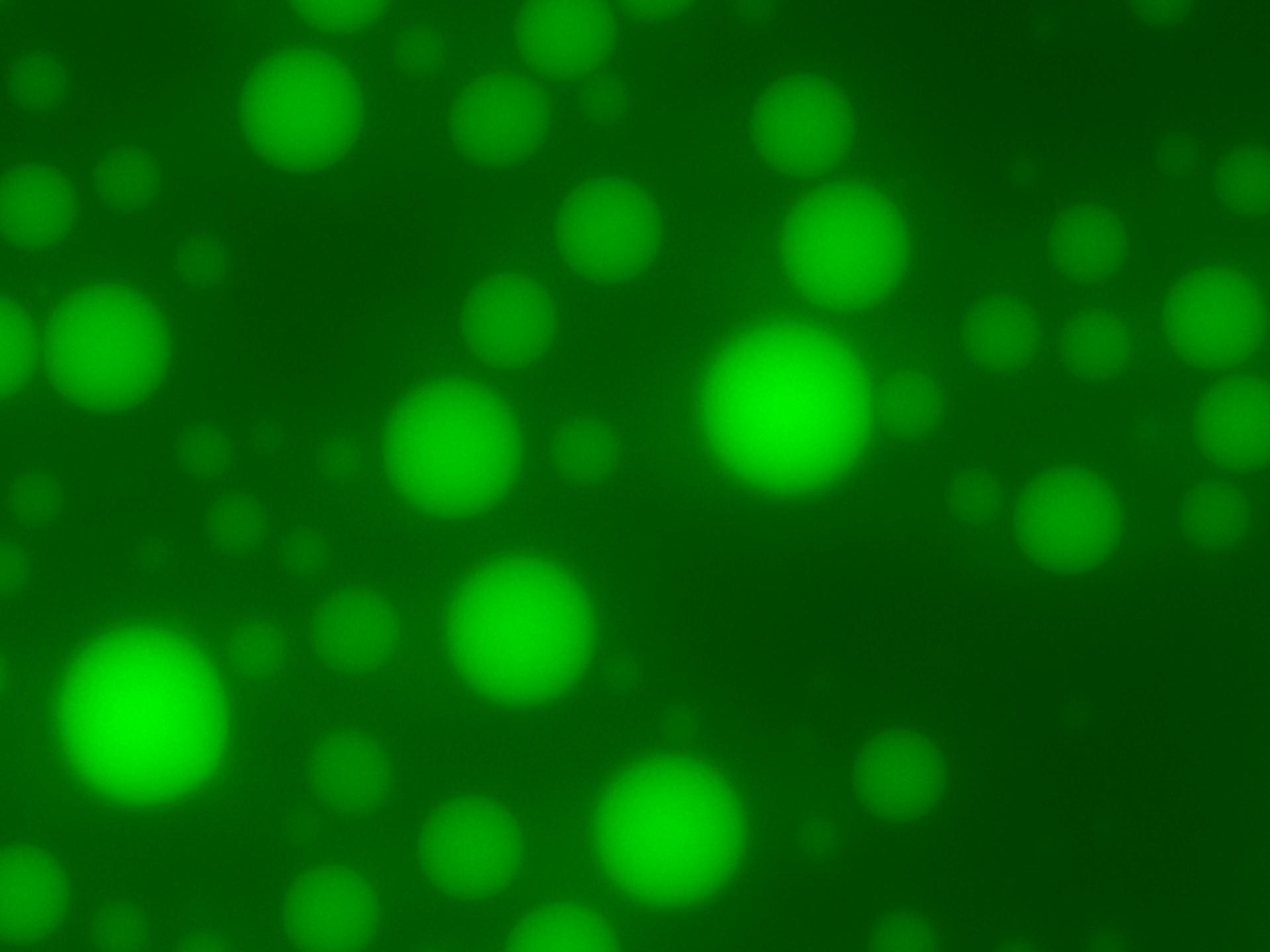

Supplement: Supplementary file 7 — Source data Fig. 5 [file 44318_2025_591_MOESM7_ESM.zip › Figure 5/5B/16_48 h_SO286_UBQLN2.tif]

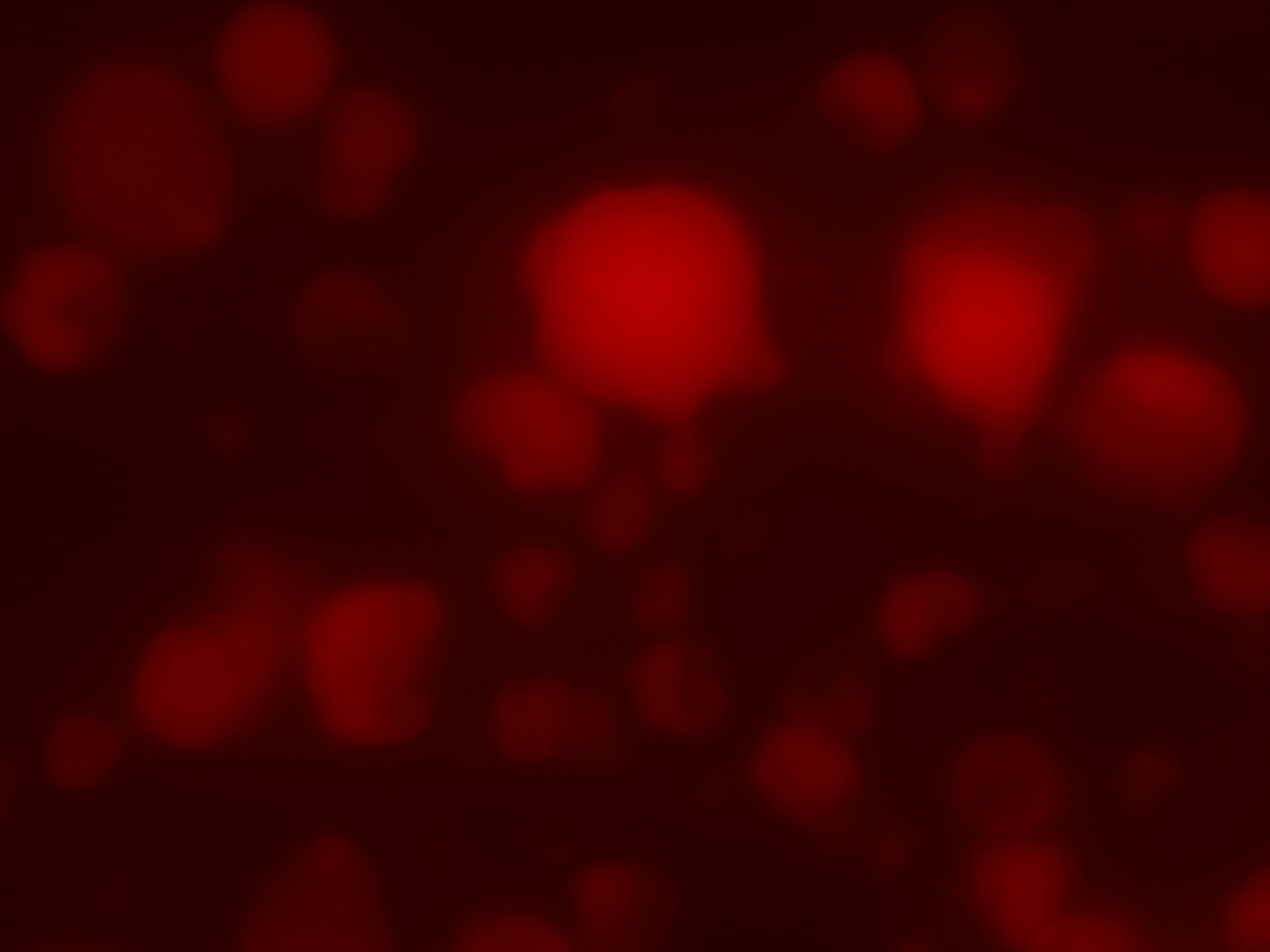

Supplement: Supplementary file 7 — Source data Fig. 5 [file 44318_2025_591_MOESM7_ESM.zip › Figure 5/5B/26_96 h_Control_╬▒-Syn.tif]

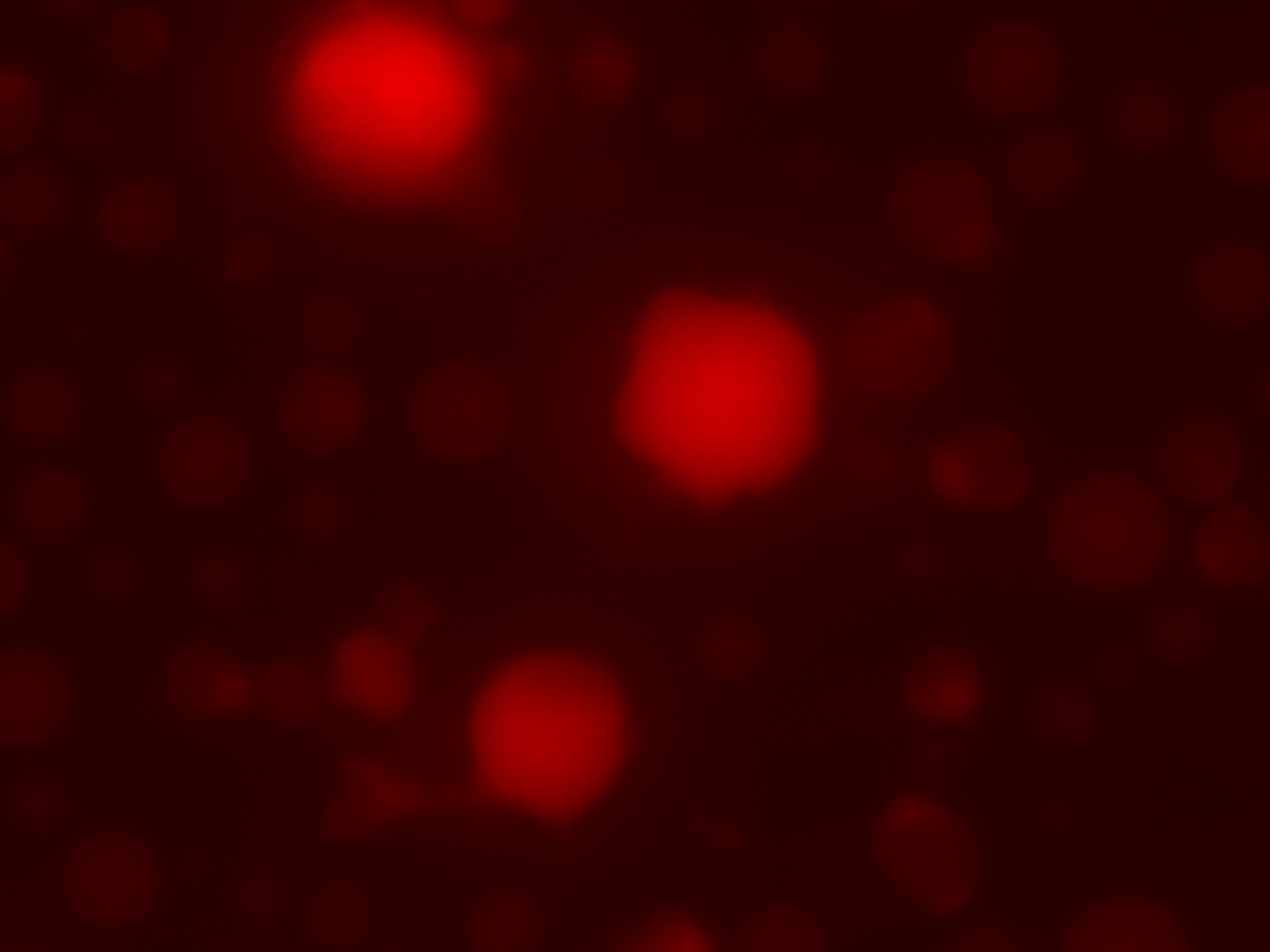

Supplement: Supplementary file 7 — Source data Fig. 5 [file 44318_2025_591_MOESM7_ESM.zip › Figure 5/5B/20_72 h_Control_╬▒-Syn.tif]

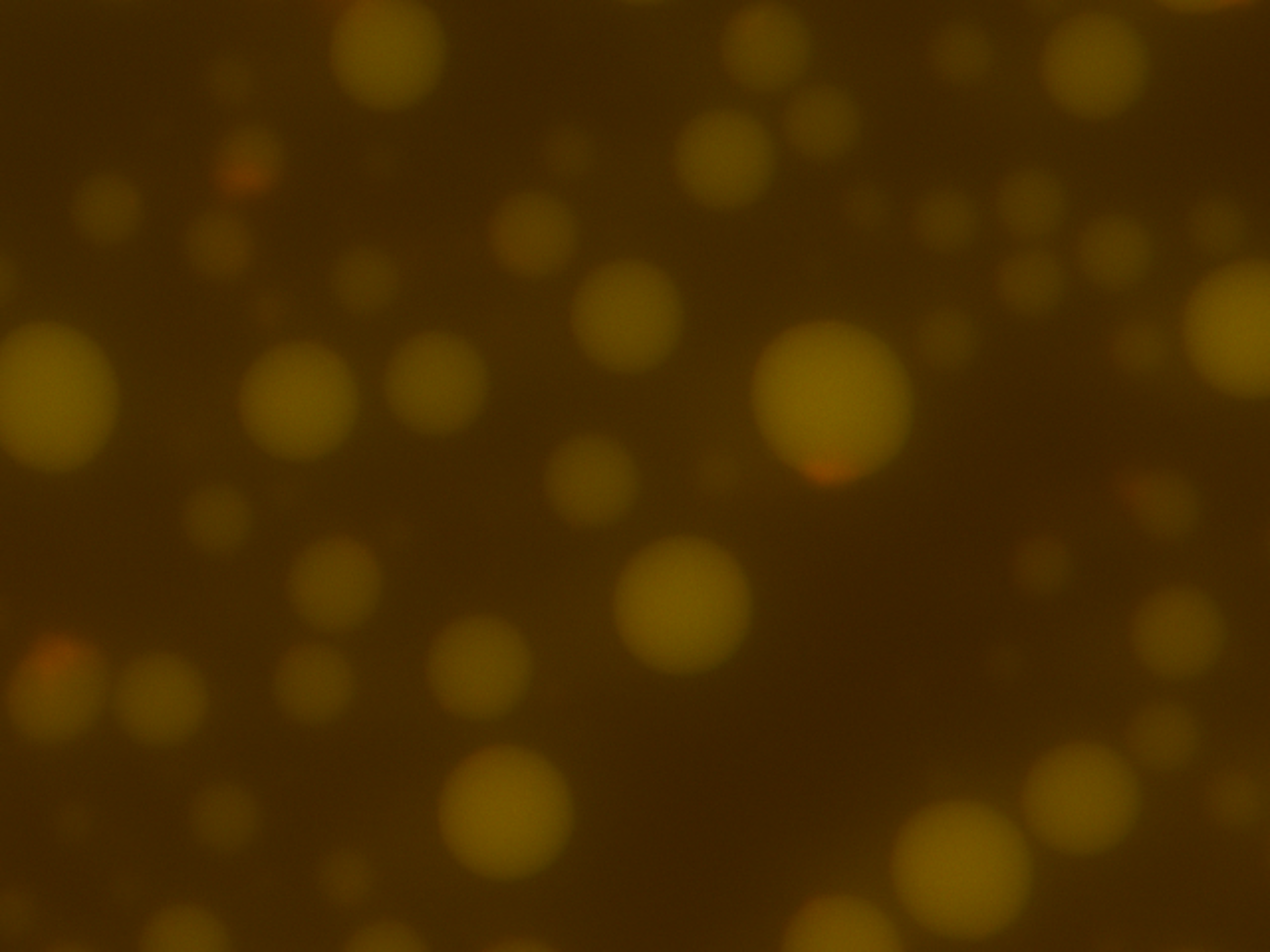

Supplement: Supplementary file 7 — Source data Fig. 5 [file 44318_2025_591_MOESM7_ESM.zip › Figure 5/5B/30_96 h_SO286_Merge.tif]

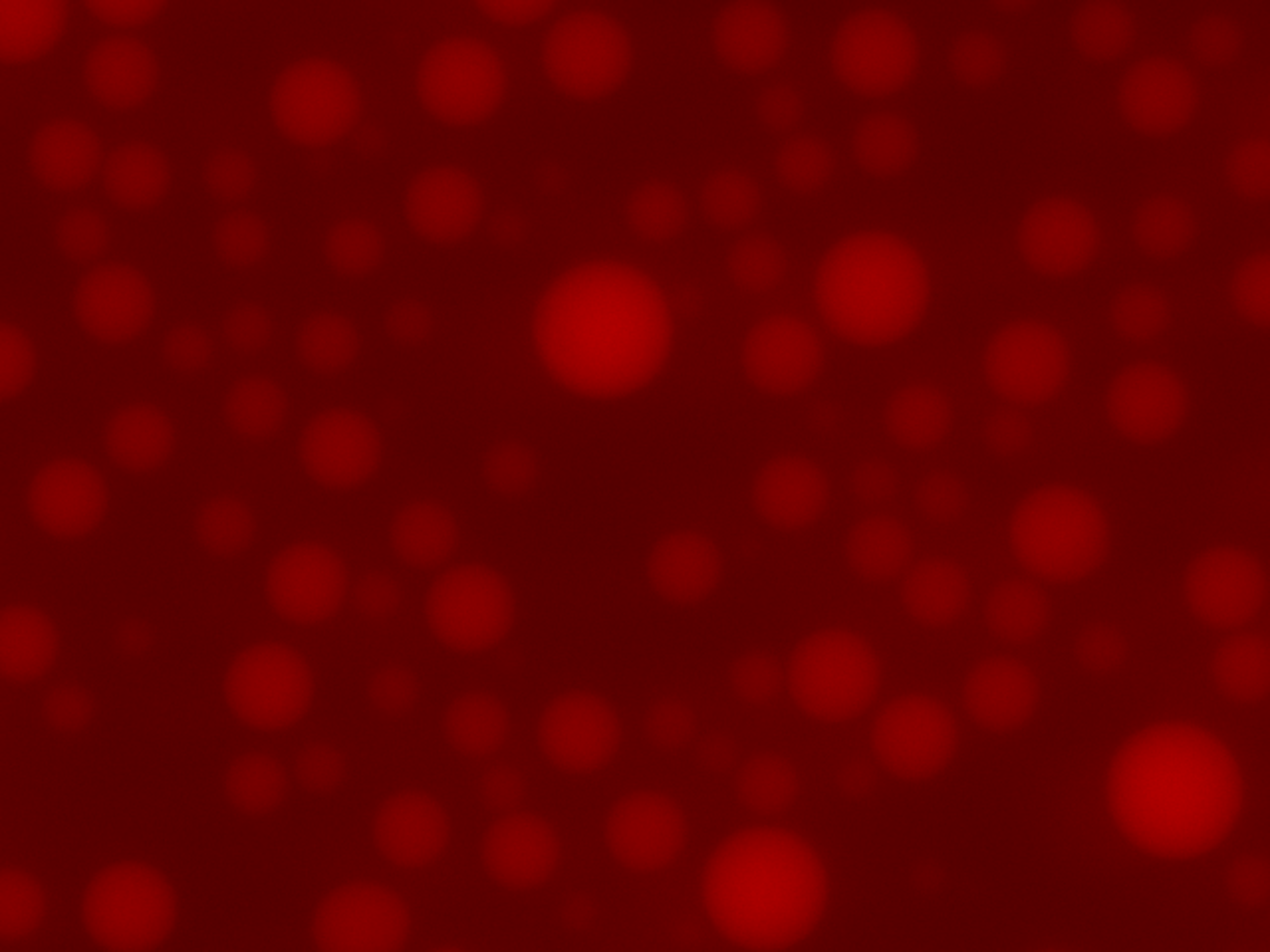

Supplement: Supplementary file 7 — Source data Fig. 5 [file 44318_2025_591_MOESM7_ESM.zip › Figure 5/5B/11_24 h_SO286_╬▒-Syn.tif]

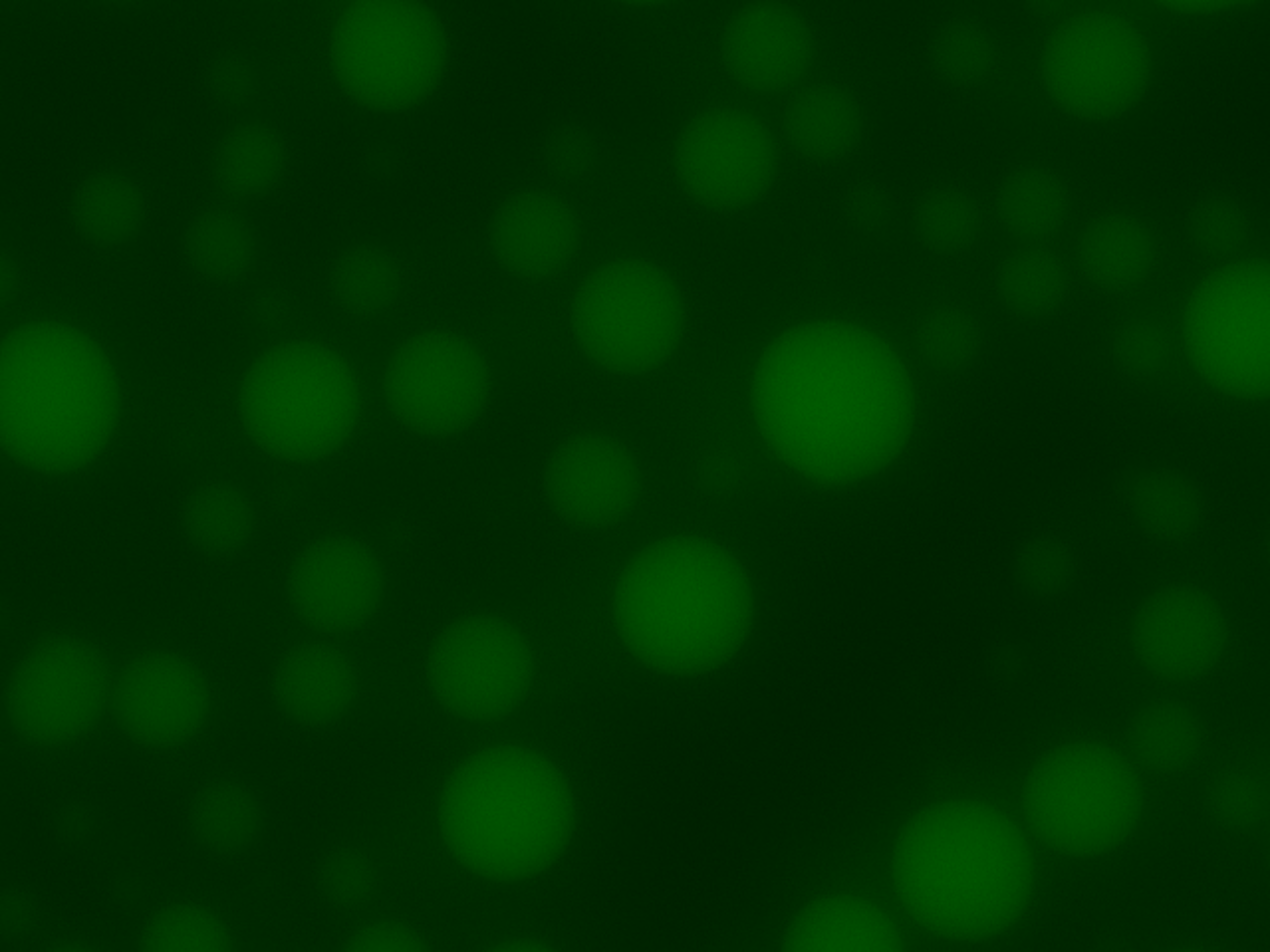

Supplement: Supplementary file 7 — Source data Fig. 5 [file 44318_2025_591_MOESM7_ESM.zip › Figure 5/5B/28_96 h_SO286_UBQLN2.tif]

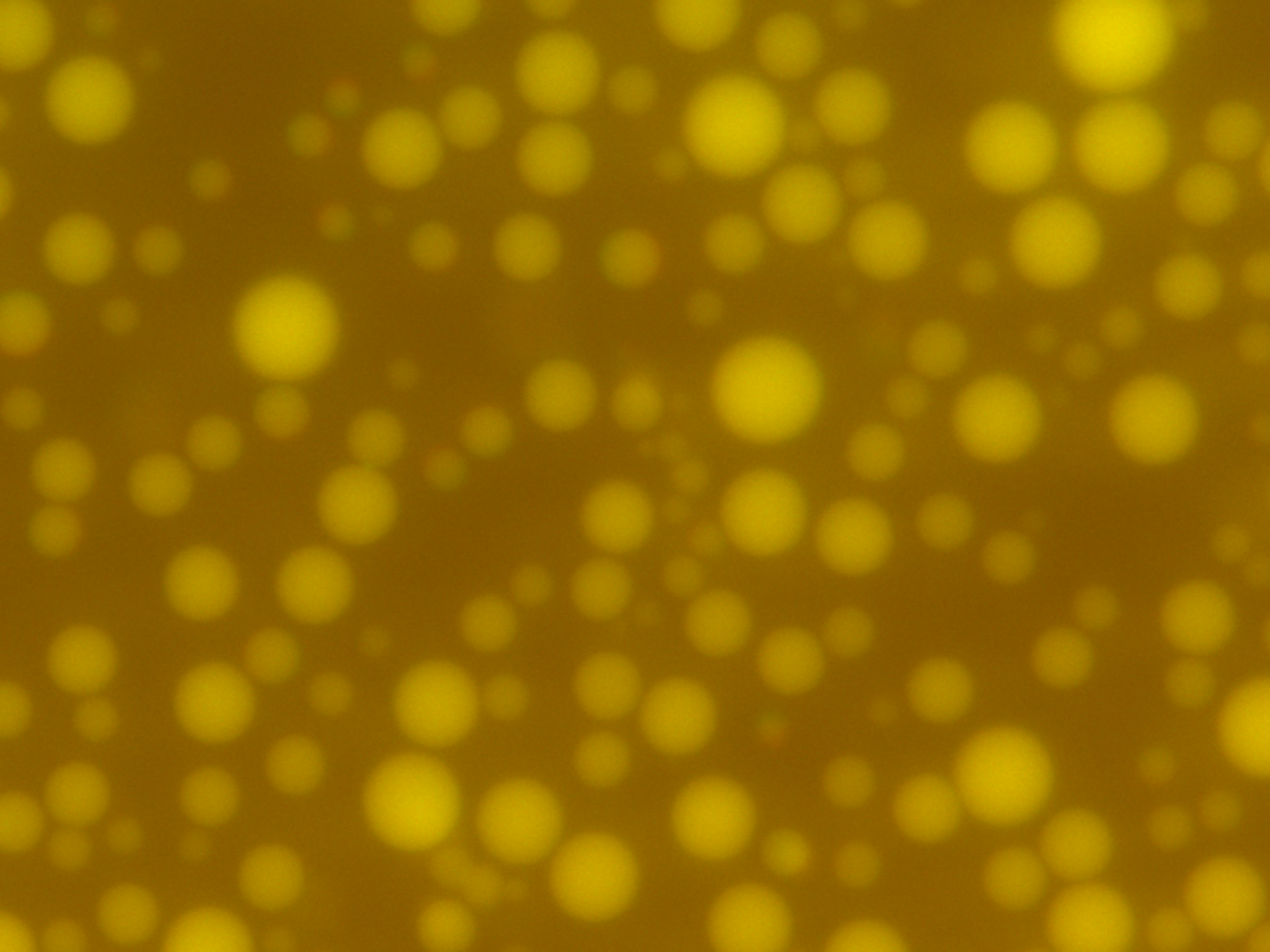

Supplement: Supplementary file 7 — Source data Fig. 5 [file 44318_2025_591_MOESM7_ESM.zip › Figure 5/5B/03_1 h_Control_Merge.tif]

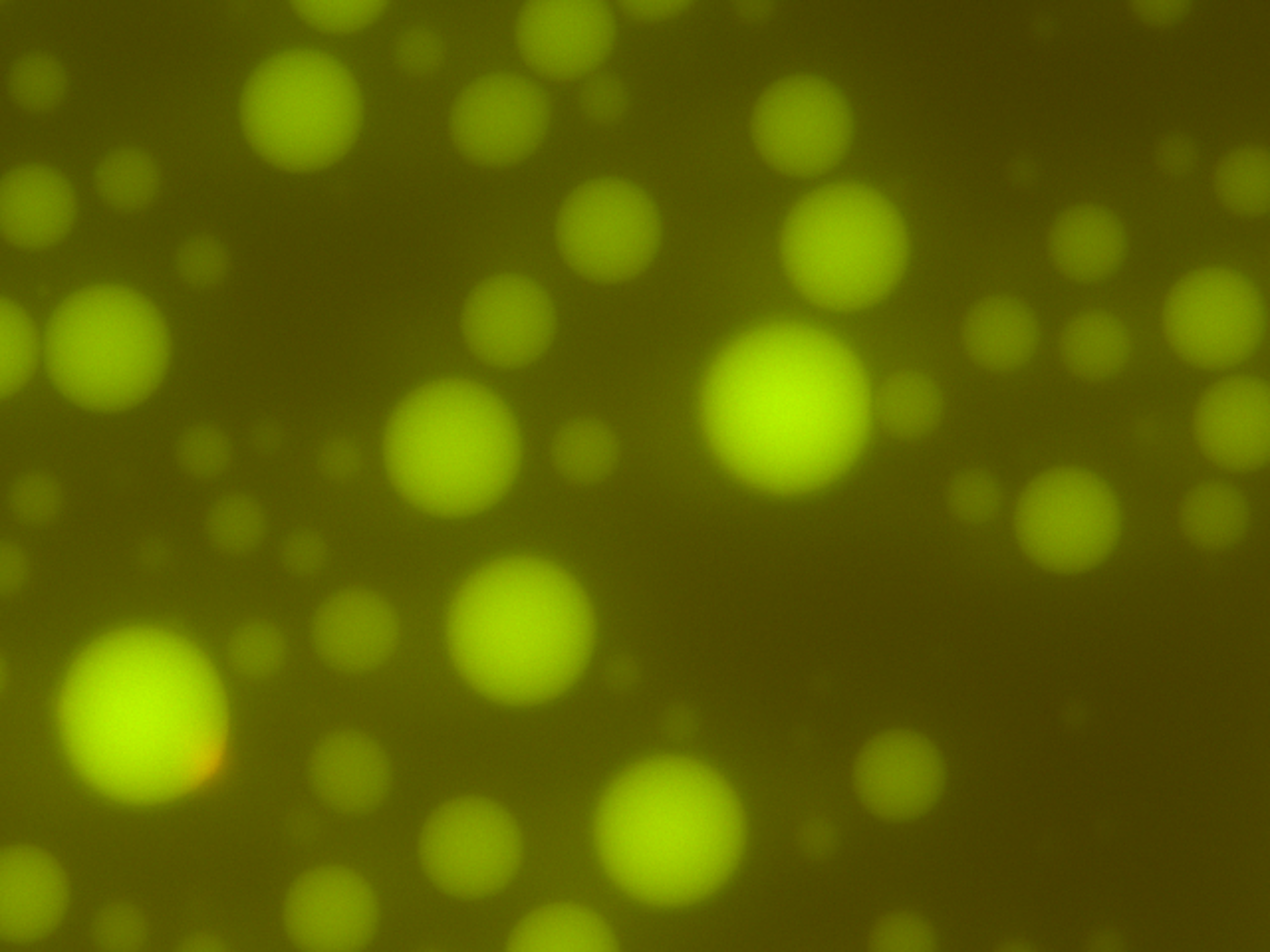

Supplement: Supplementary file 7 — Source data Fig. 5 [file 44318_2025_591_MOESM7_ESM.zip › Figure 5/5B/18_48 h_SO286_Merge.tif]

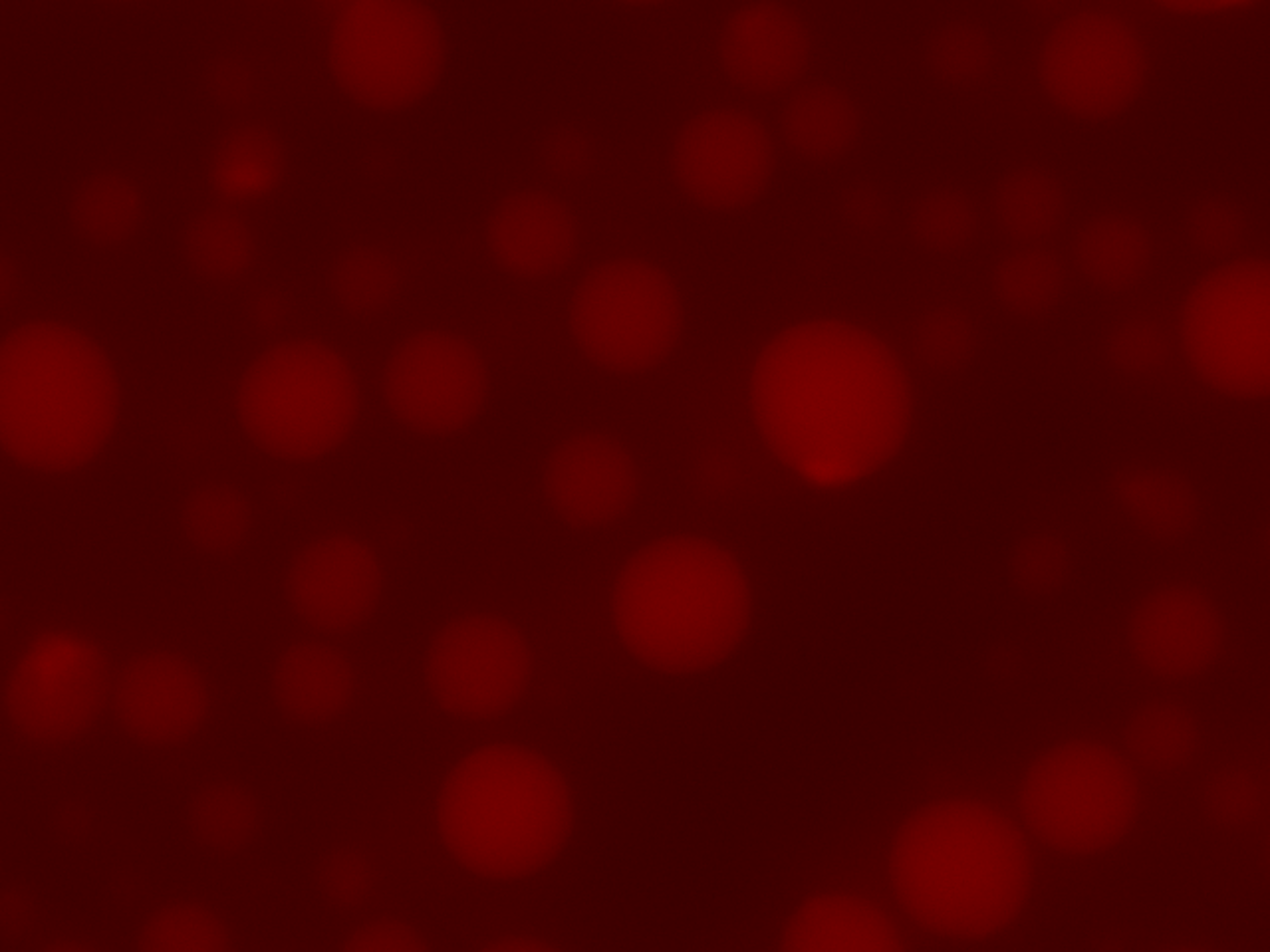

Supplement: Supplementary file 7 — Source data Fig. 5 [file 44318_2025_591_MOESM7_ESM.zip › Figure 5/5B/29_96 h_SO286_╬▒-Syn.tif]

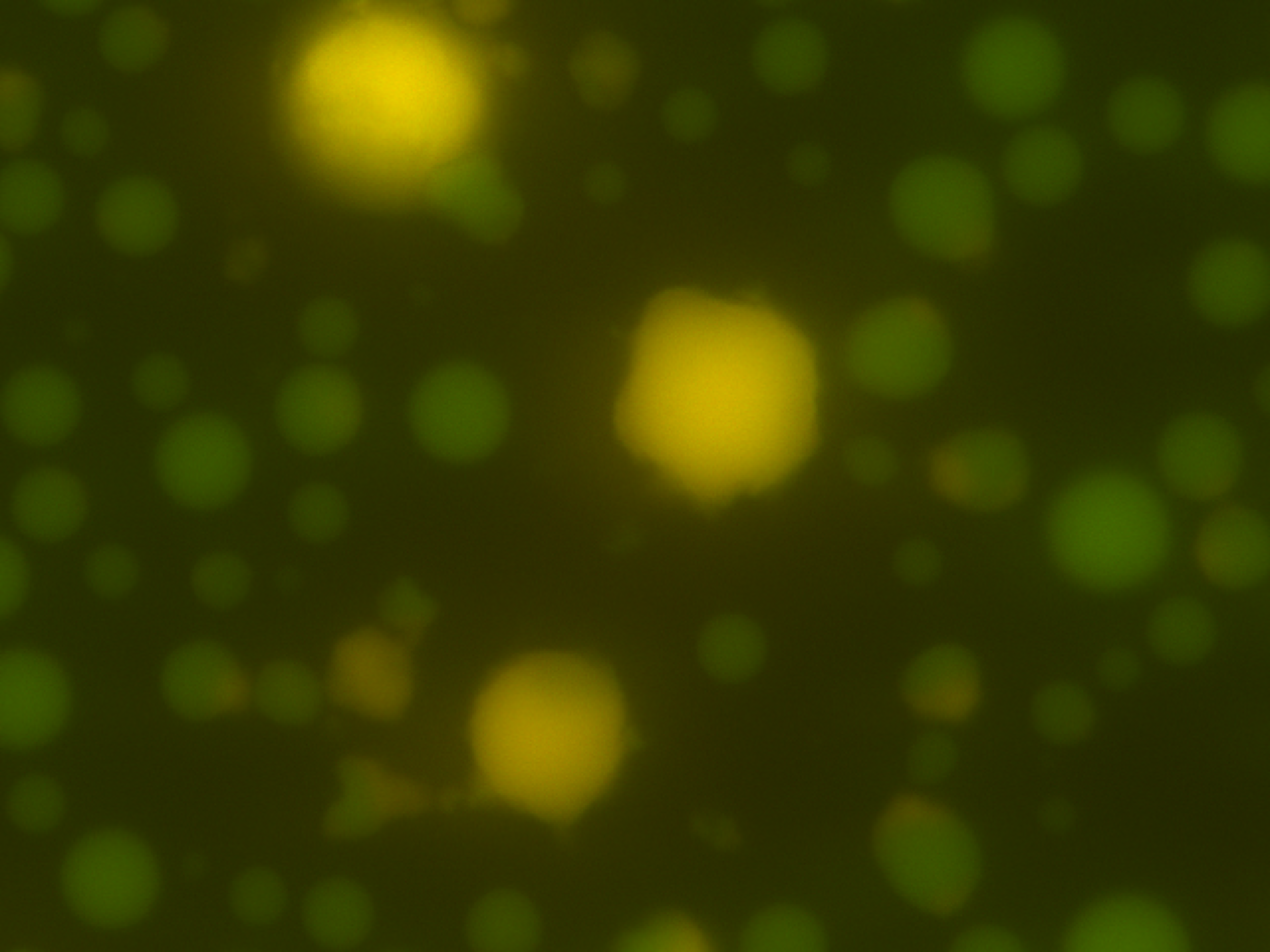

Supplement: Supplementary file 7 — Source data Fig. 5 [file 44318_2025_591_MOESM7_ESM.zip › Figure 5/5B/21_72 h_Control_Merge.tif]

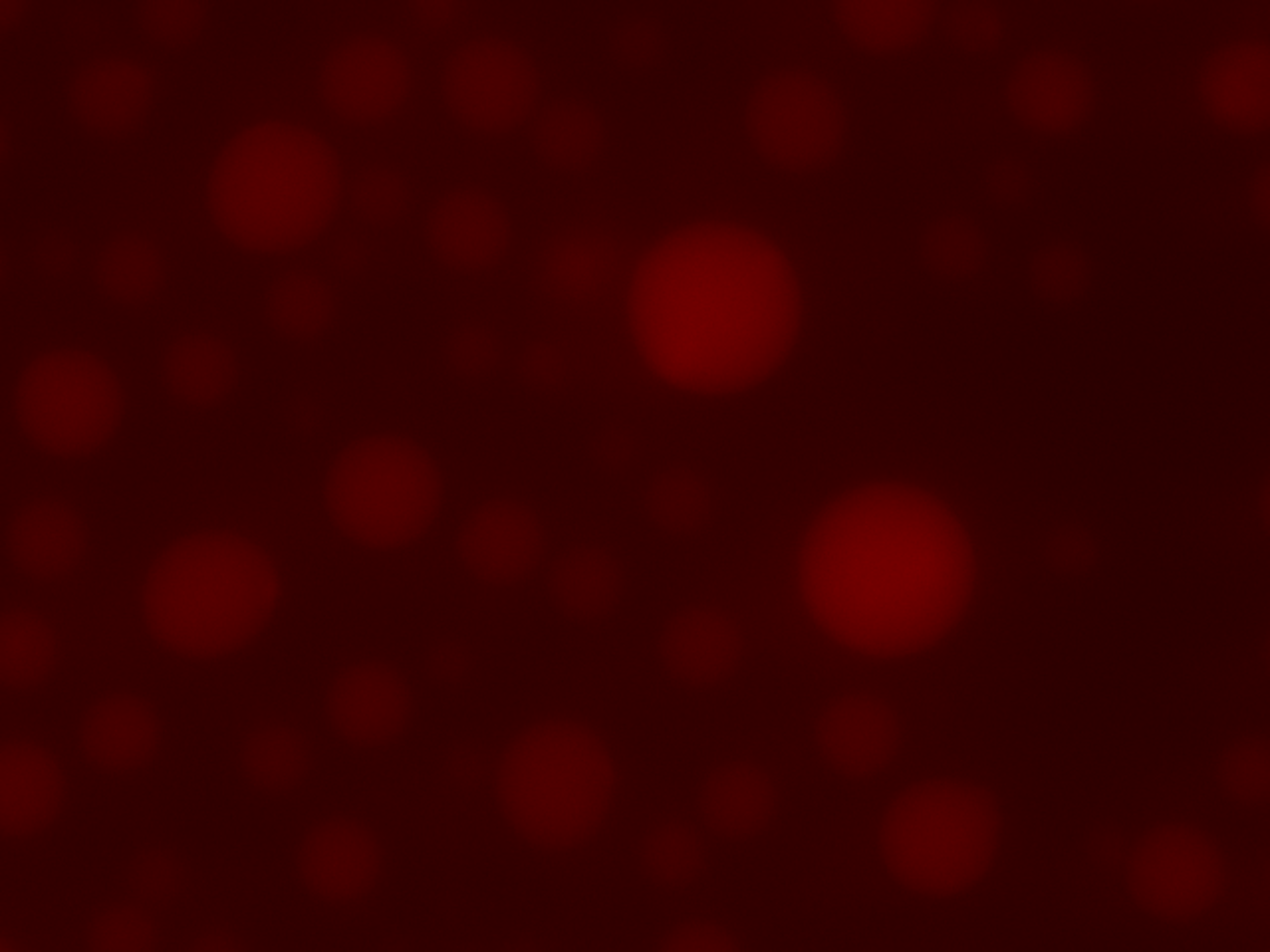

Supplement: Supplementary file 7 — Source data Fig. 5 [file 44318_2025_591_MOESM7_ESM.zip › Figure 5/5B/23_72 h_SO286_╬▒-Syn.tif]

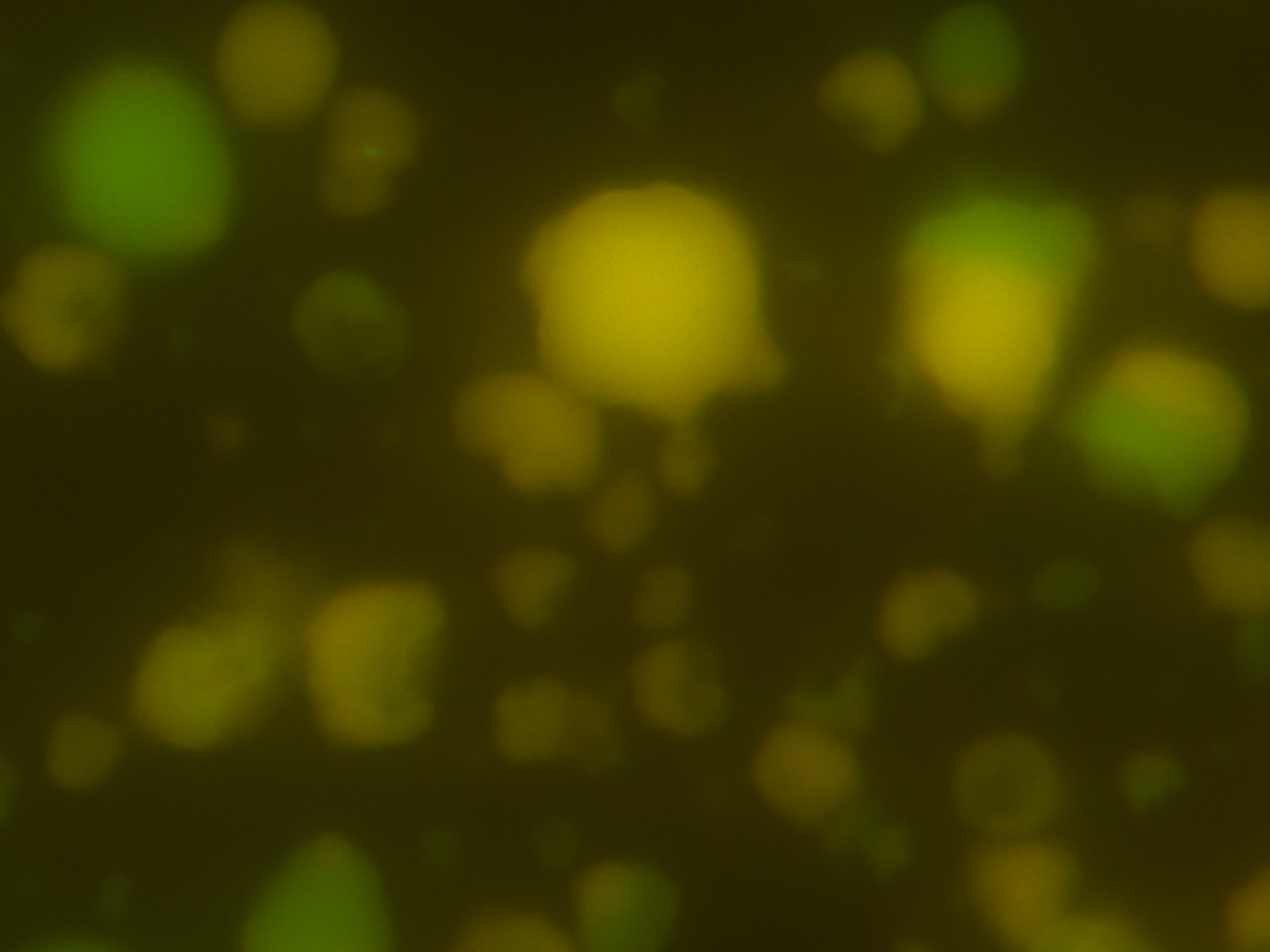

Supplement: Supplementary file 7 — Source data Fig. 5 [file 44318_2025_591_MOESM7_ESM.zip › Figure 5/5B/27_96 h_Control_Merge.tif]

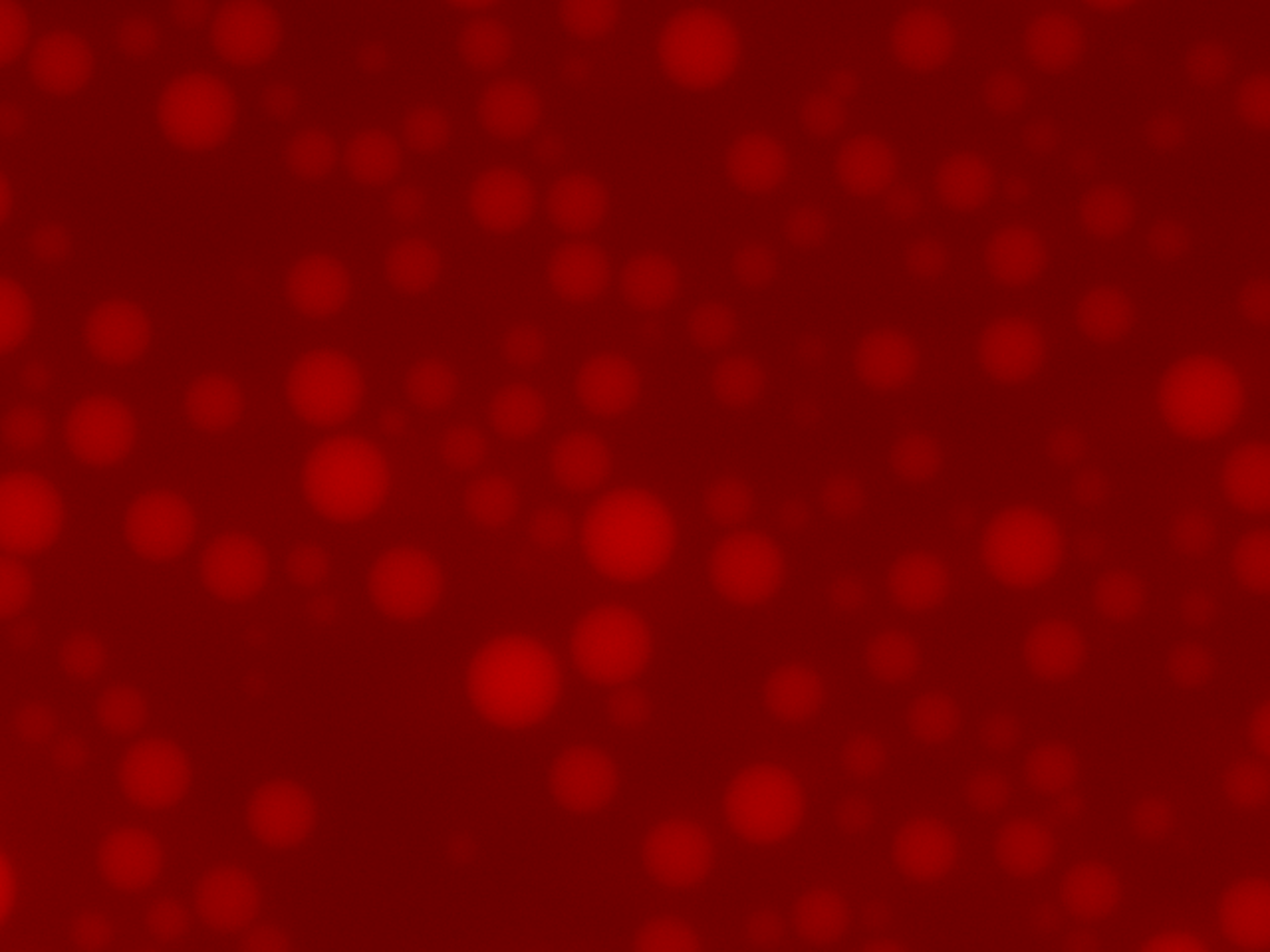

Supplement: Supplementary file 7 — Source data Fig. 5 [file 44318_2025_591_MOESM7_ESM.zip › Figure 5/5B/05_1 h_SO286_╬▒-Syn.tif]

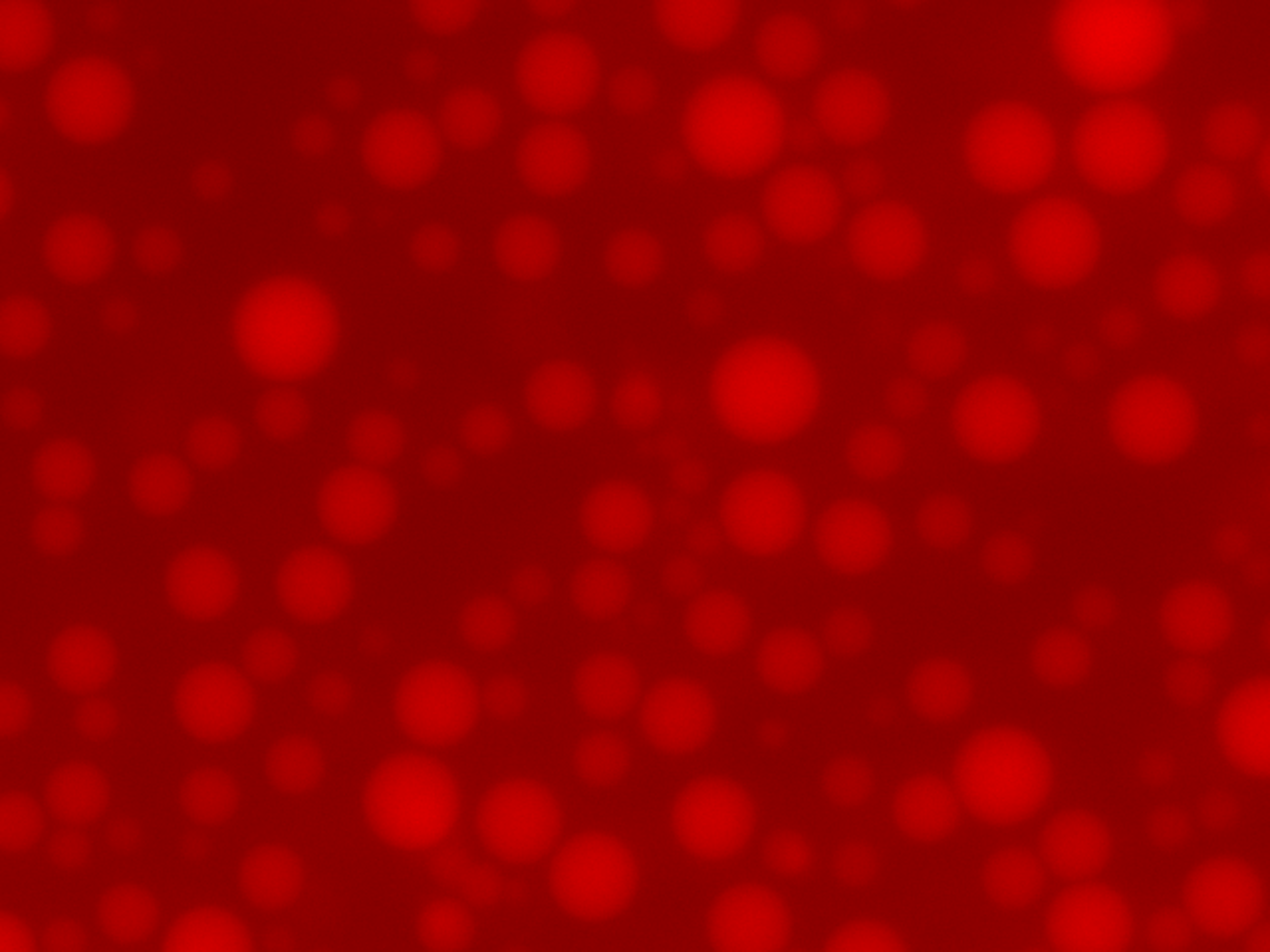

Supplement: Supplementary file 7 — Source data Fig. 5 [file 44318_2025_591_MOESM7_ESM.zip › Figure 5/5B/02_1 h_Control_╬▒-Syn.tif]

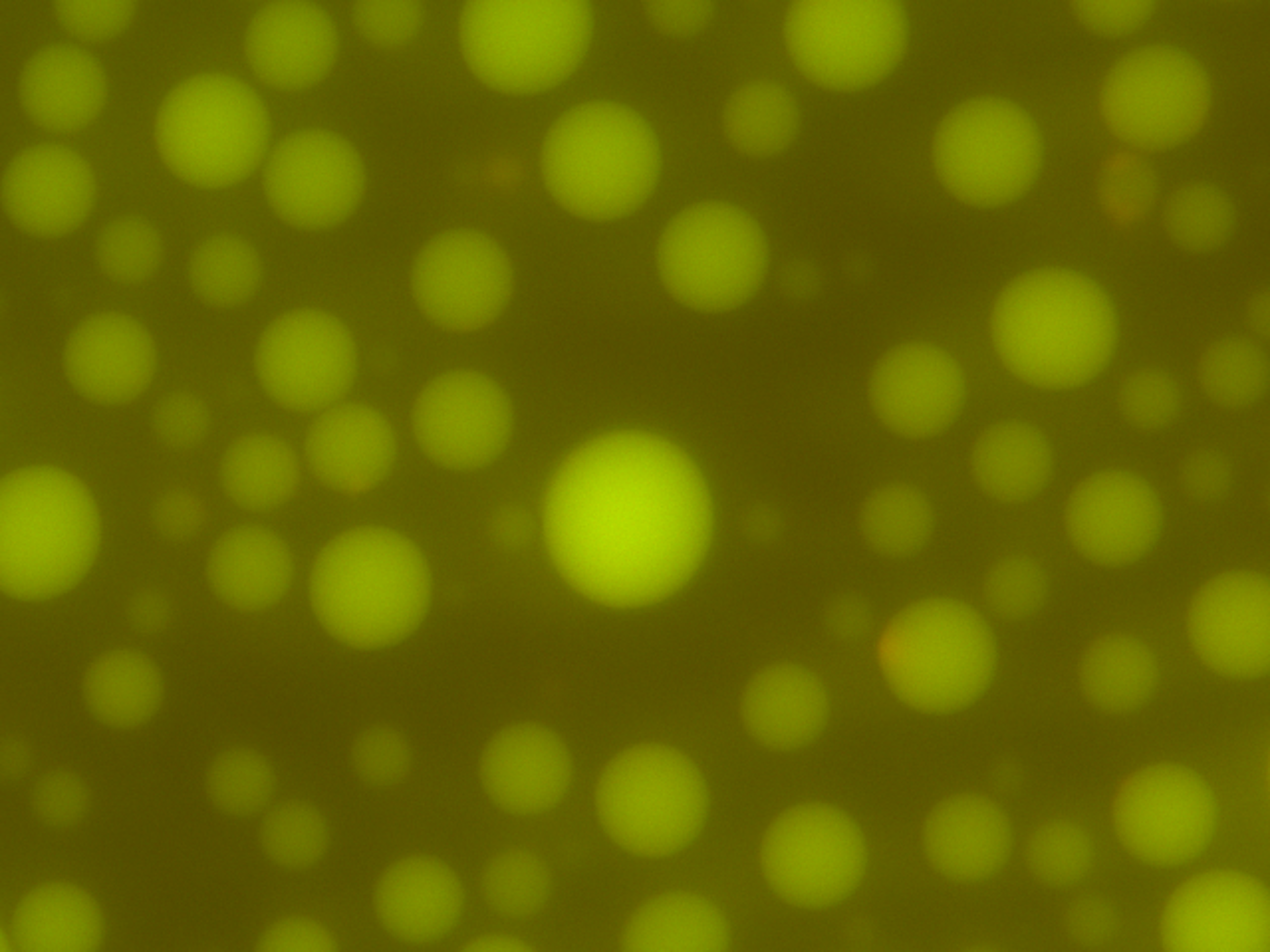

Supplement: Supplementary file 7 — Source data Fig. 5 [file 44318_2025_591_MOESM7_ESM.zip › Figure 5/5B/15_48 h_Control_Merge.tif]

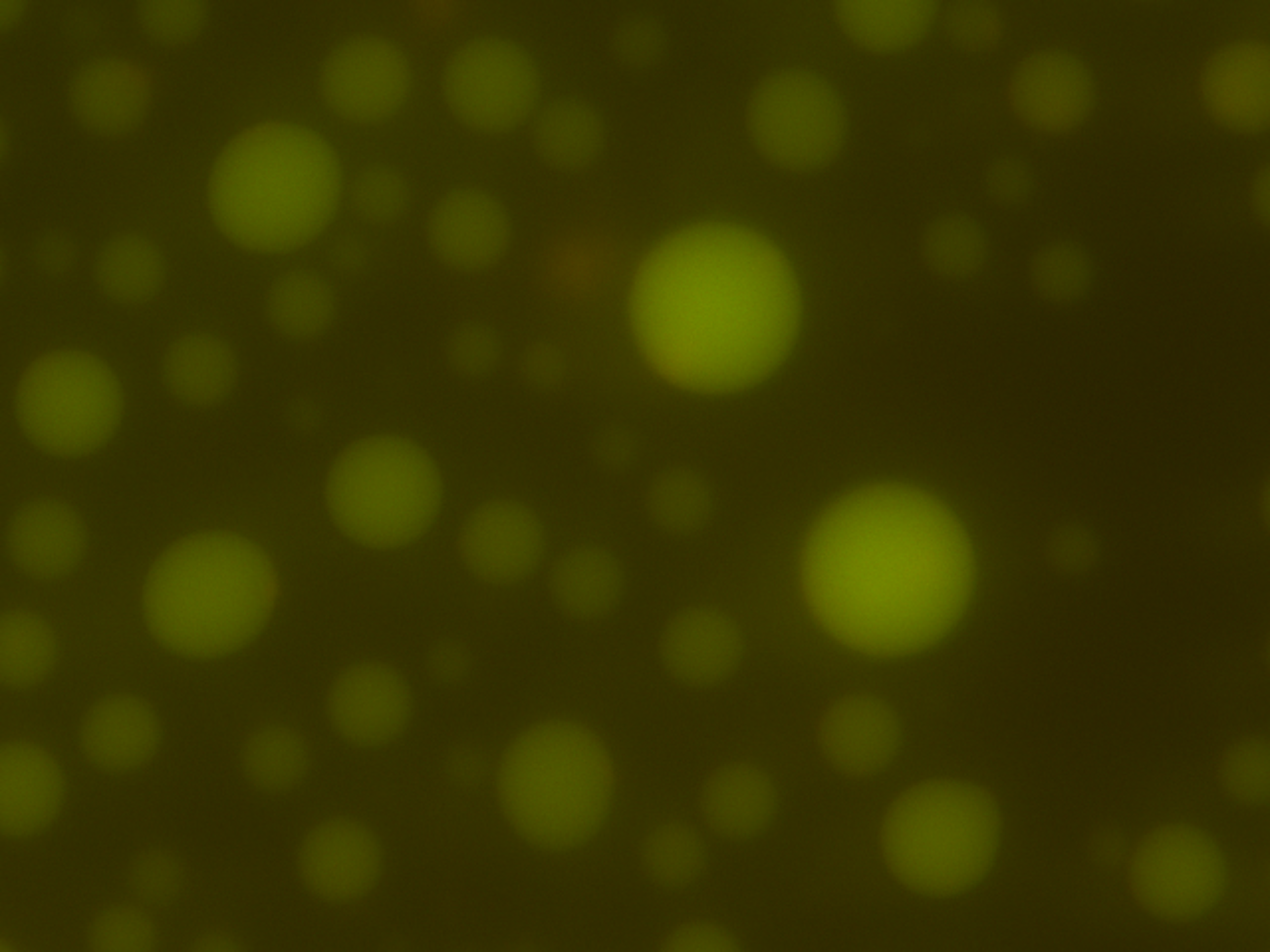

Supplement: Supplementary file 7 — Source data Fig. 5 [file 44318_2025_591_MOESM7_ESM.zip › Figure 5/5B/24_72 h_SO286_Merge.tif]

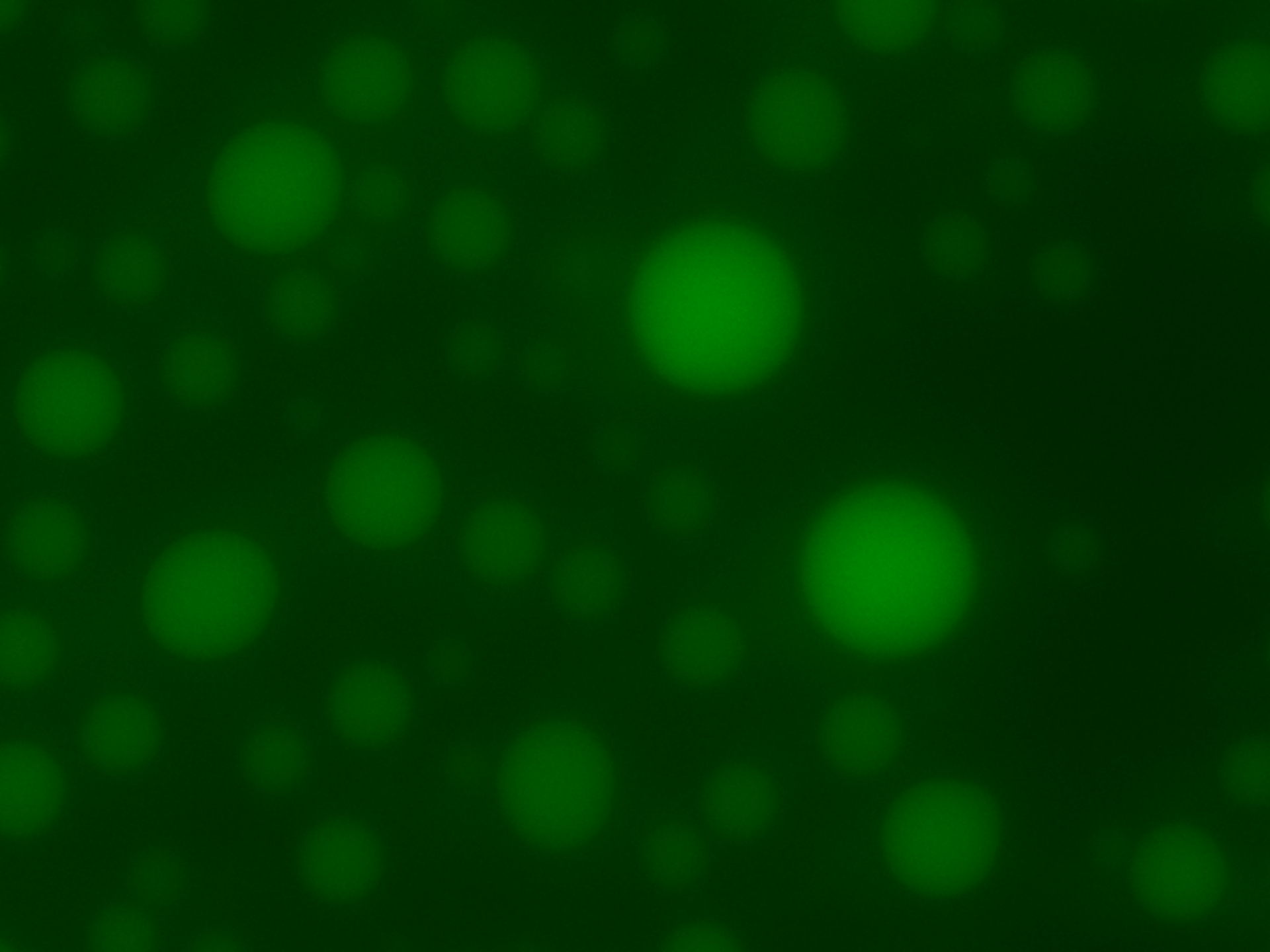

Supplement: Supplementary file 7 — Source data Fig. 5 [file 44318_2025_591_MOESM7_ESM.zip › Figure 5/5B/22_72 h_SO286_UBQLN2.tif]

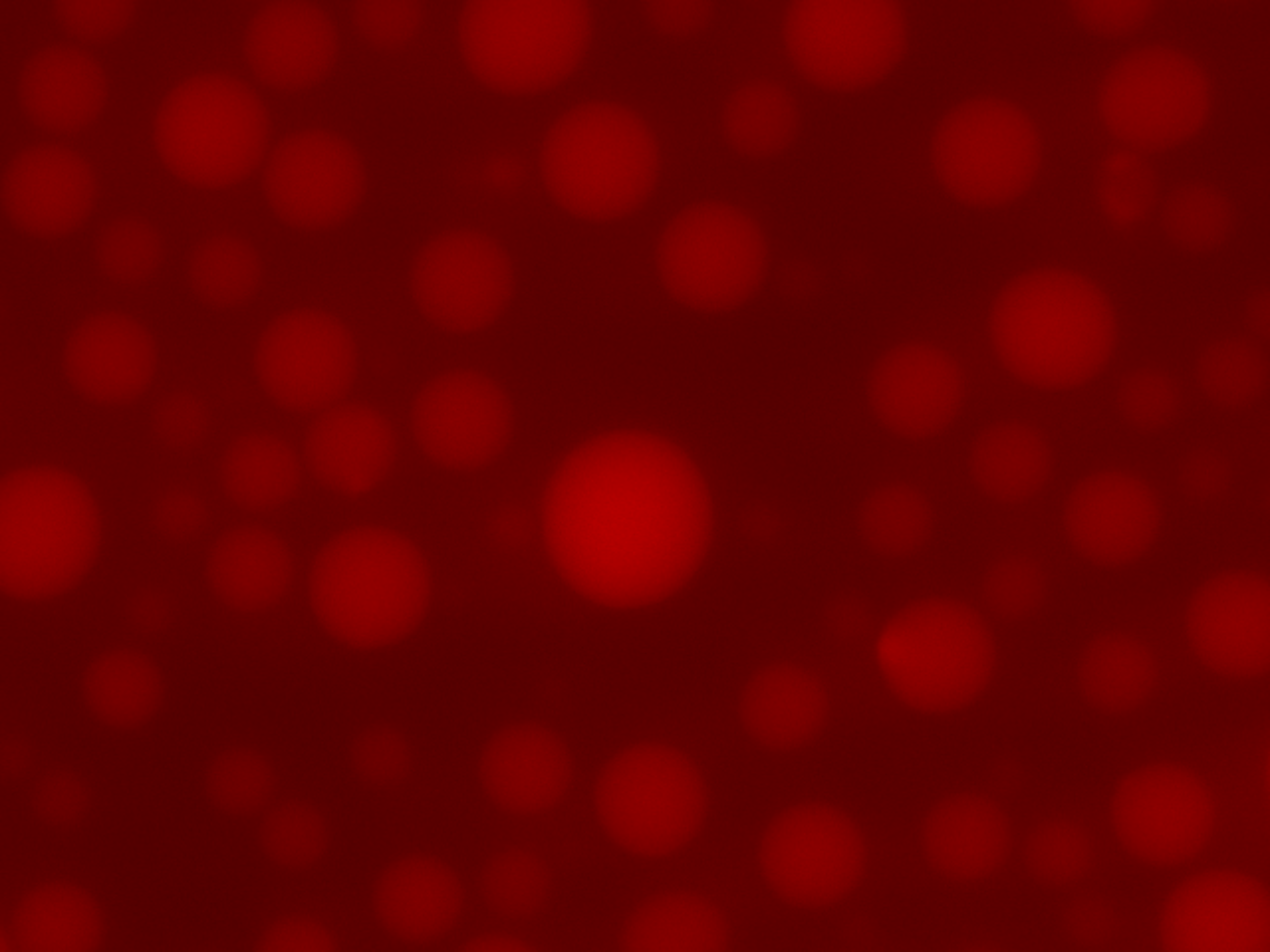

Supplement: Supplementary file 7 — Source data Fig. 5 [file 44318_2025_591_MOESM7_ESM.zip › Figure 5/5B/14_48 h_Control_╬▒-Syn.tif]

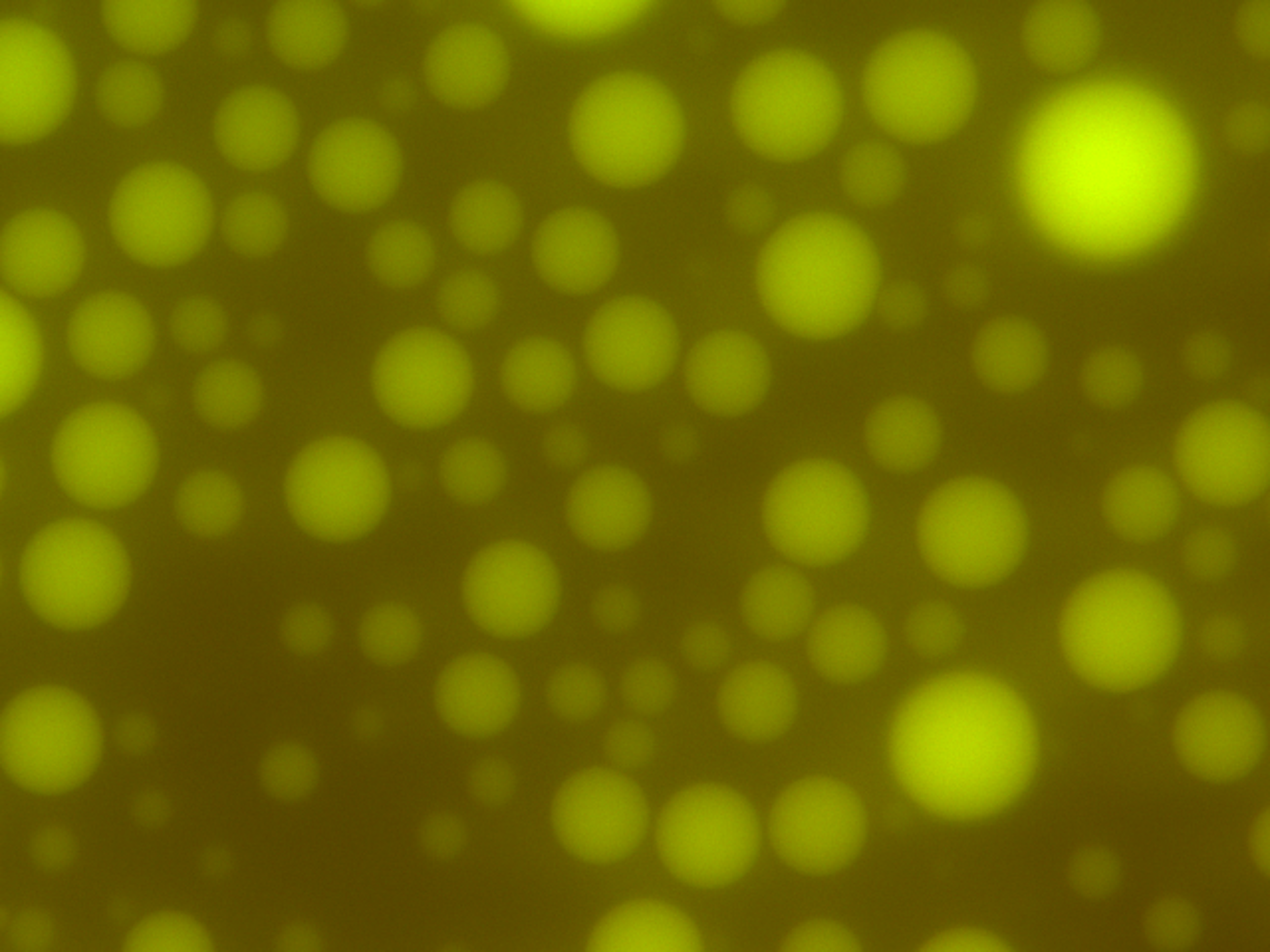

Supplement: Supplementary file 7 — Source data Fig. 5 [file 44318_2025_591_MOESM7_ESM.zip › Figure 5/5B/09_24 h_Control_Merge.tif]

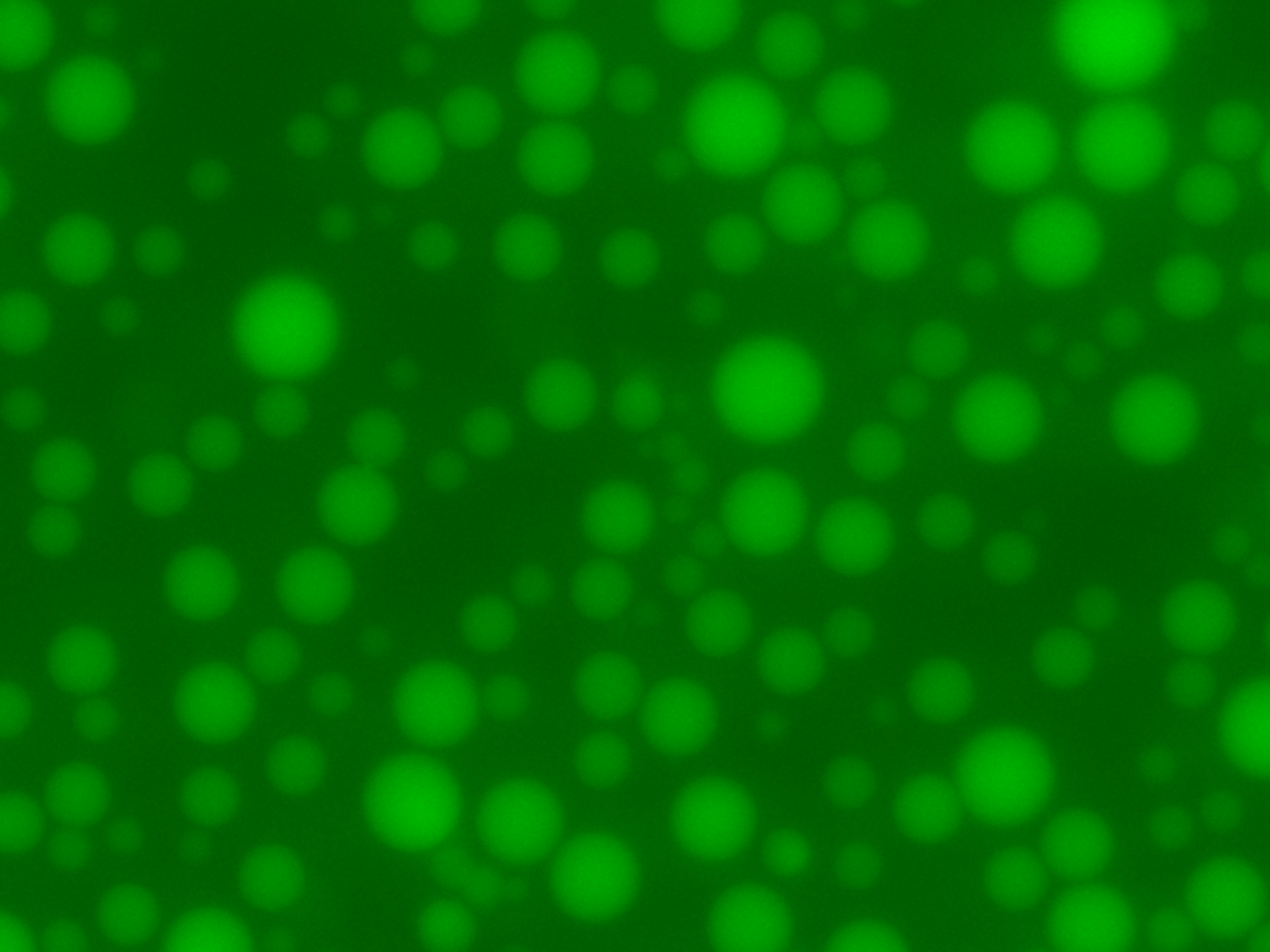

Supplement: Supplementary file 7 — Source data Fig. 5 [file 44318_2025_591_MOESM7_ESM.zip › Figure 5/5B/01_1 h_Control_UBQLN2.tif]

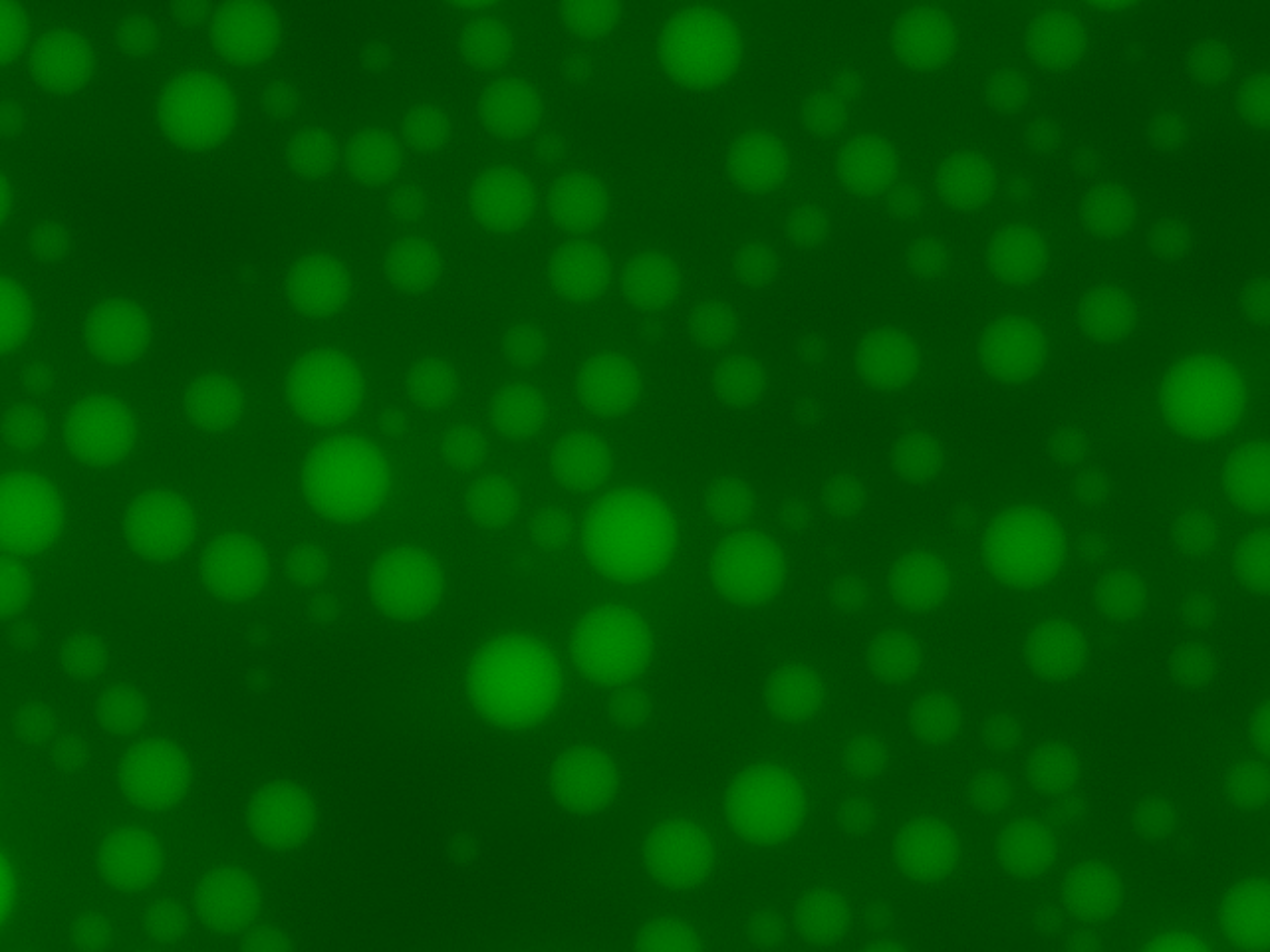

Supplement: Supplementary file 7 — Source data Fig. 5 [file 44318_2025_591_MOESM7_ESM.zip › Figure 5/5B/04_1 h_SO286_UBQLN2.tif]

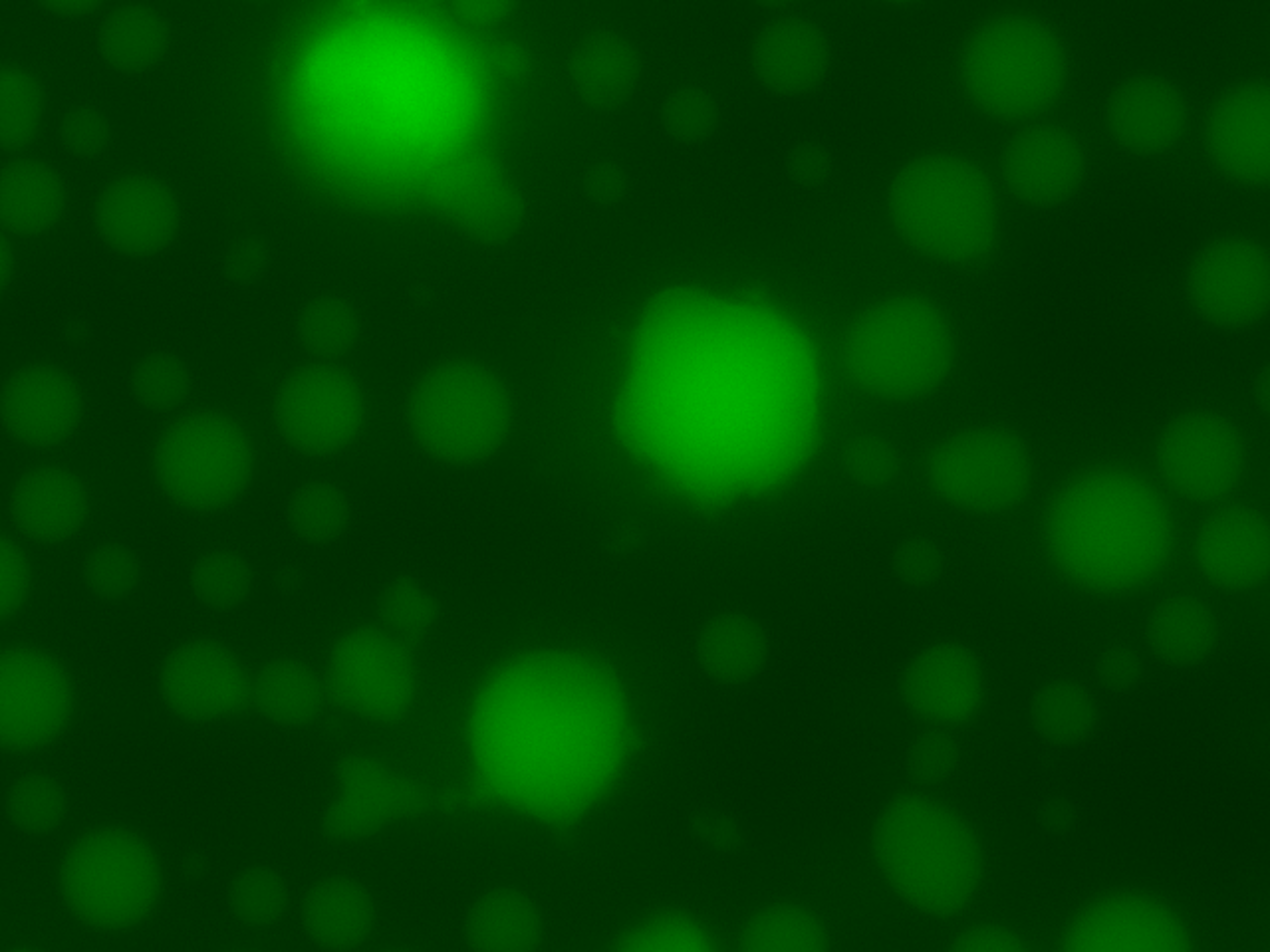

Supplement: Supplementary file 7 — Source data Fig. 5 [file 44318_2025_591_MOESM7_ESM.zip › Figure 5/5B/19_72 h_Control_UBQLN2.tif]

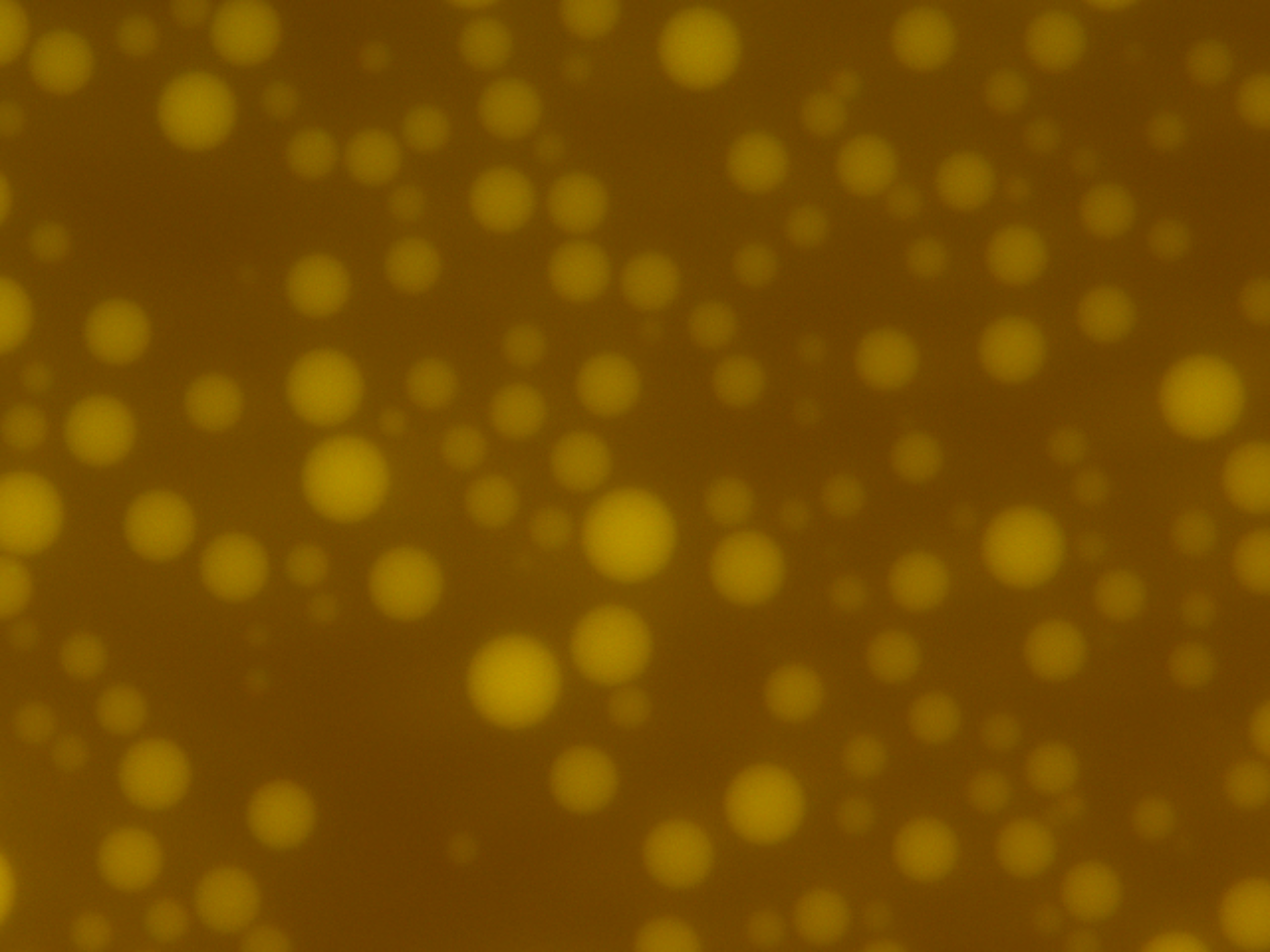

Supplement: Supplementary file 7 — Source data Fig. 5 [file 44318_2025_591_MOESM7_ESM.zip › Figure 5/5B/06_1 h_SO286_Merge.tif]

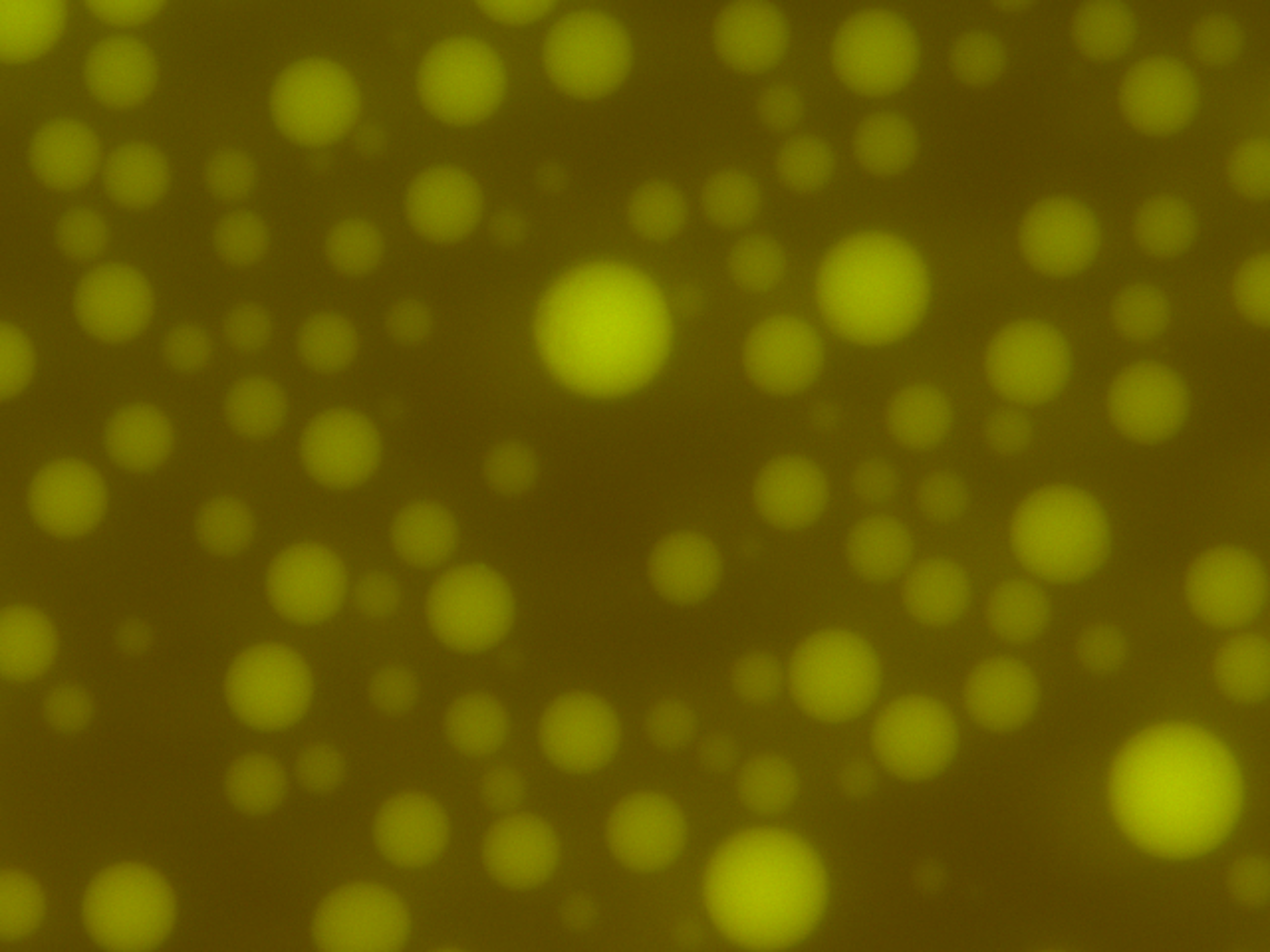

Supplement: Supplementary file 7 — Source data Fig. 5 [file 44318_2025_591_MOESM7_ESM.zip › Figure 5/5B/12_24 h_SO286_Merge.tif]

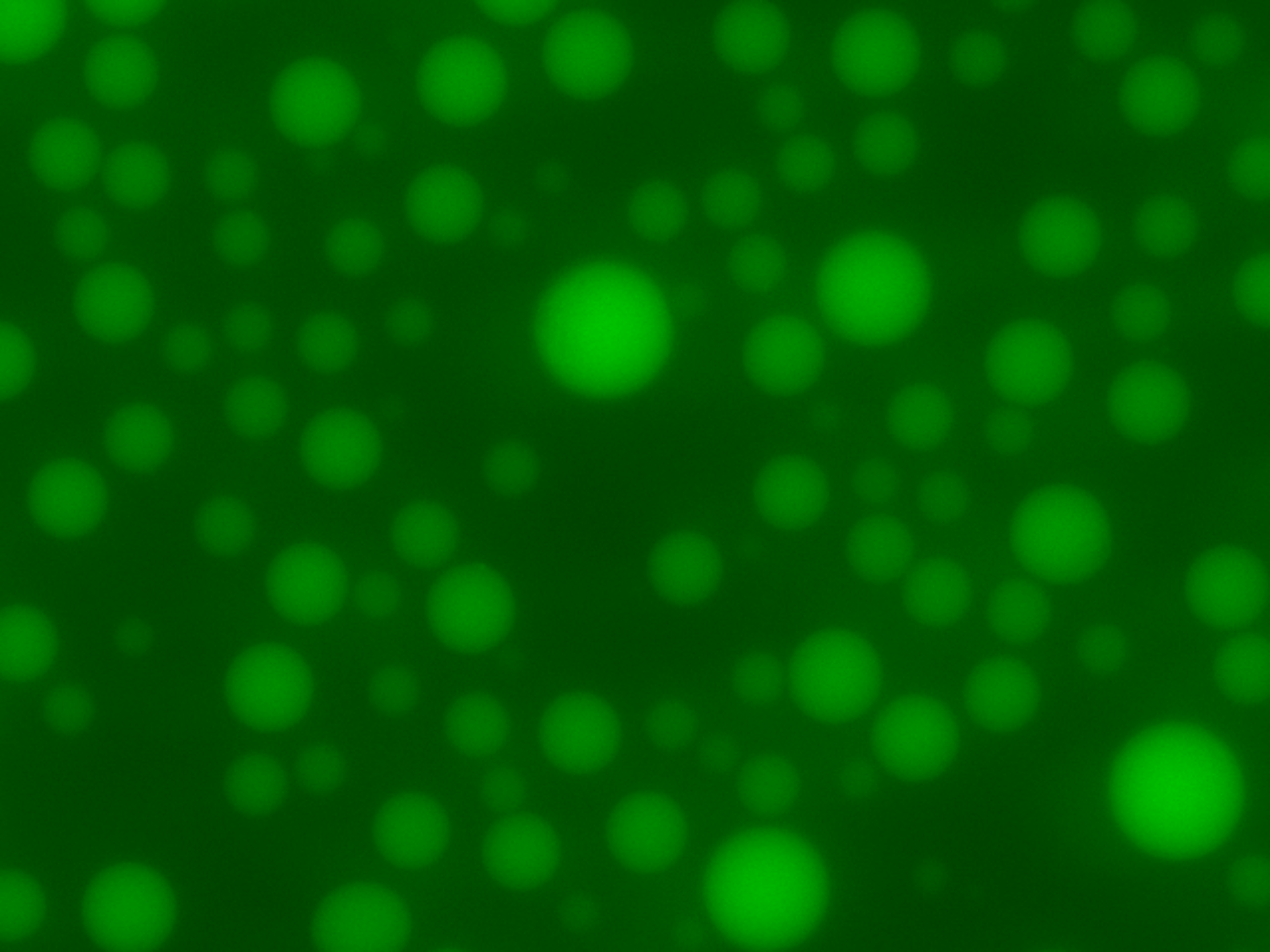

Supplement: Supplementary file 7 — Source data Fig. 5 [file 44318_2025_591_MOESM7_ESM.zip › Figure 5/5B/10_24 h_SO286_UBQLN2.tif]

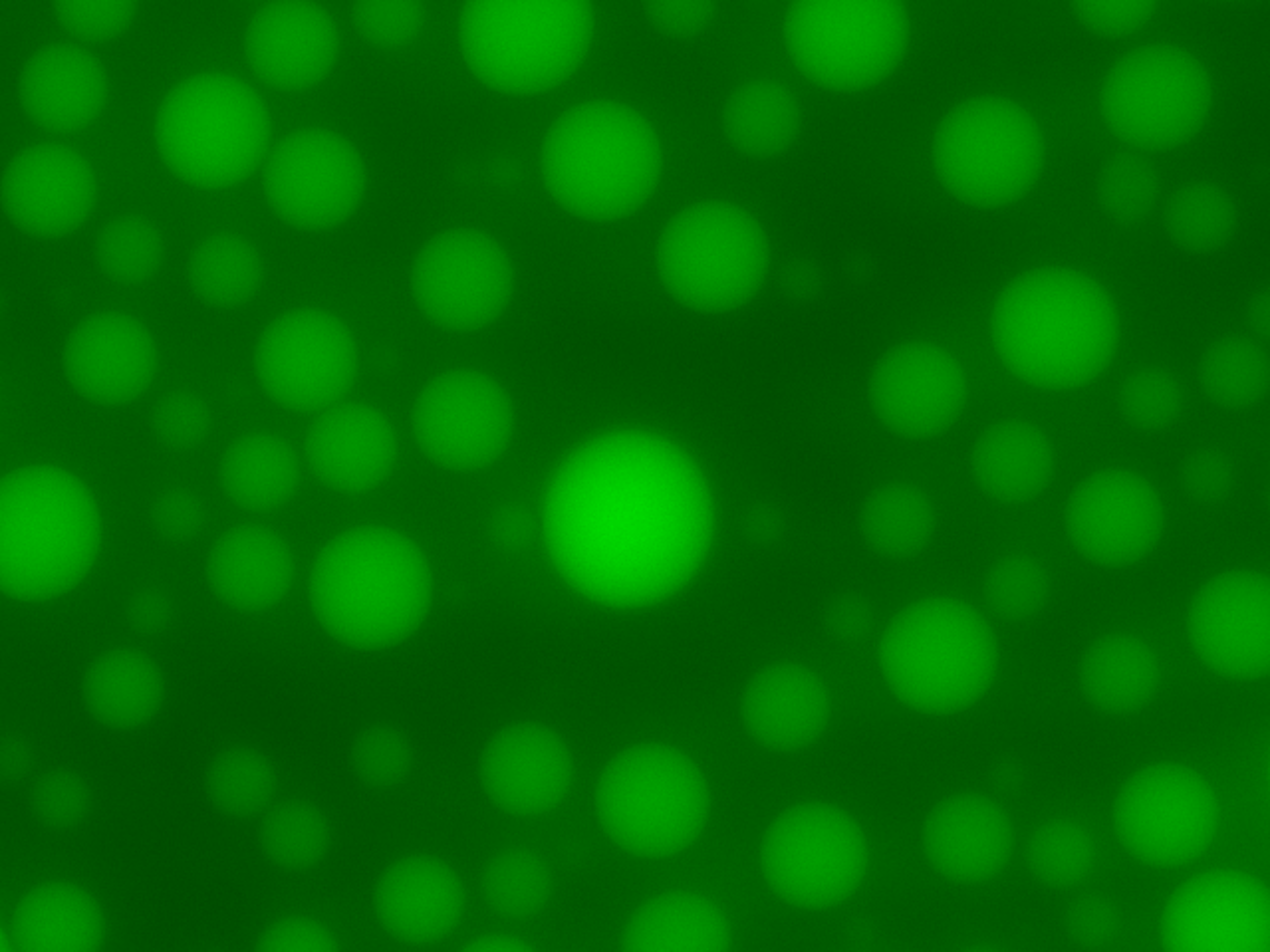

Supplement: Supplementary file 7 — Source data Fig. 5 [file 44318_2025_591_MOESM7_ESM.zip › Figure 5/5B/13_48 h_Control_UBQLN2.tif]

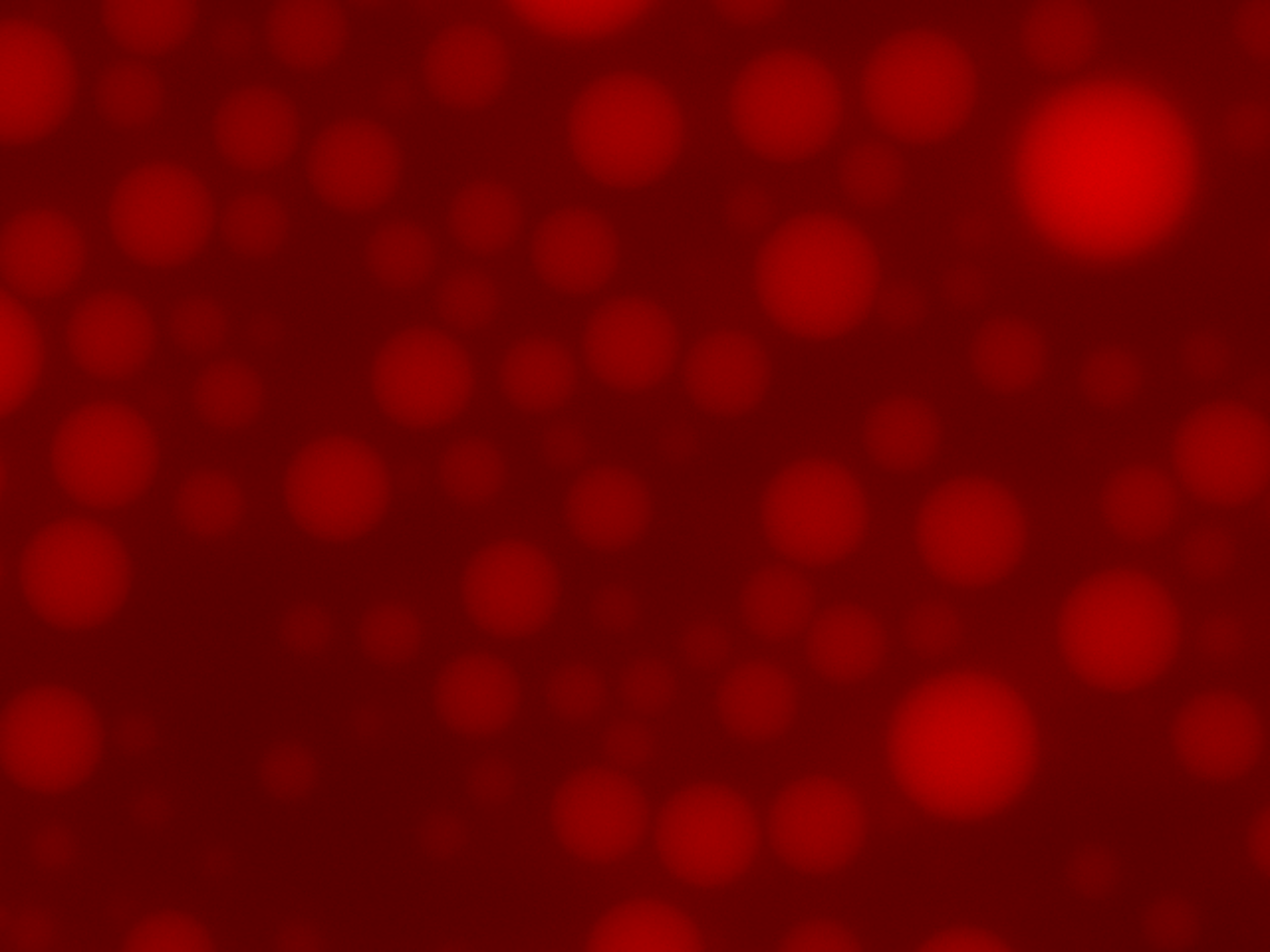

Supplement: Supplementary file 7 — Source data Fig. 5 [file 44318_2025_591_MOESM7_ESM.zip › Figure 5/5B/08_24 h_Control_╬▒-Syn.tif]

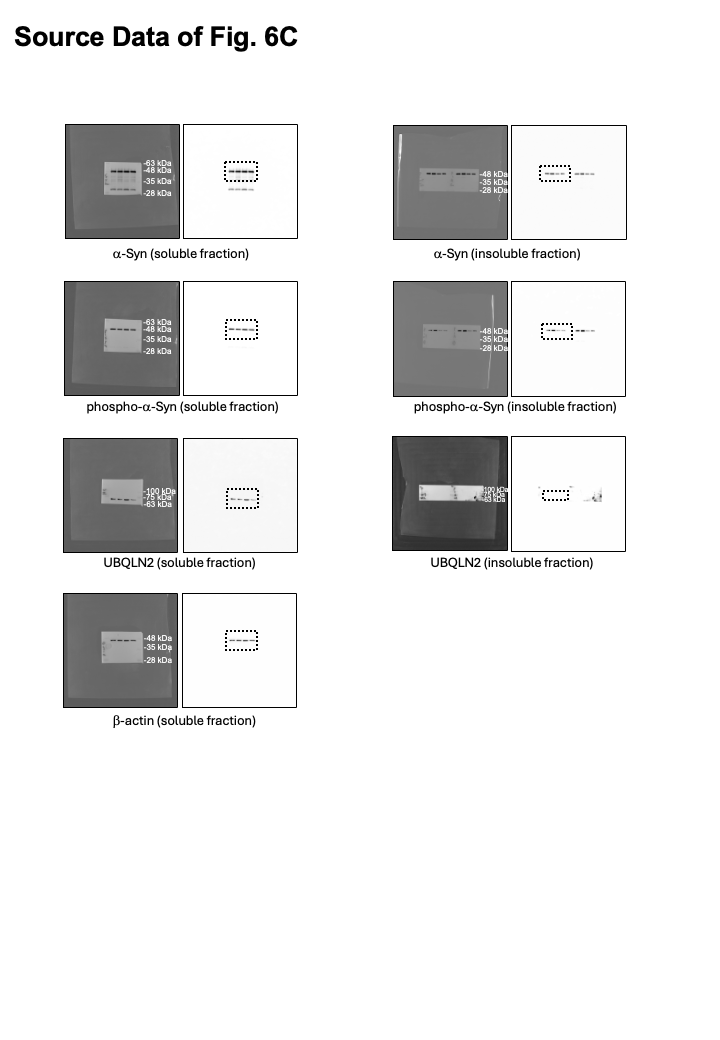

Supplement: Supplementary file 8 — Source data Fig. 6 [file 44318_2025_591_MOESM8_ESM.zip › FIgure 6/6C/08_Highlight of crop area.tiff]

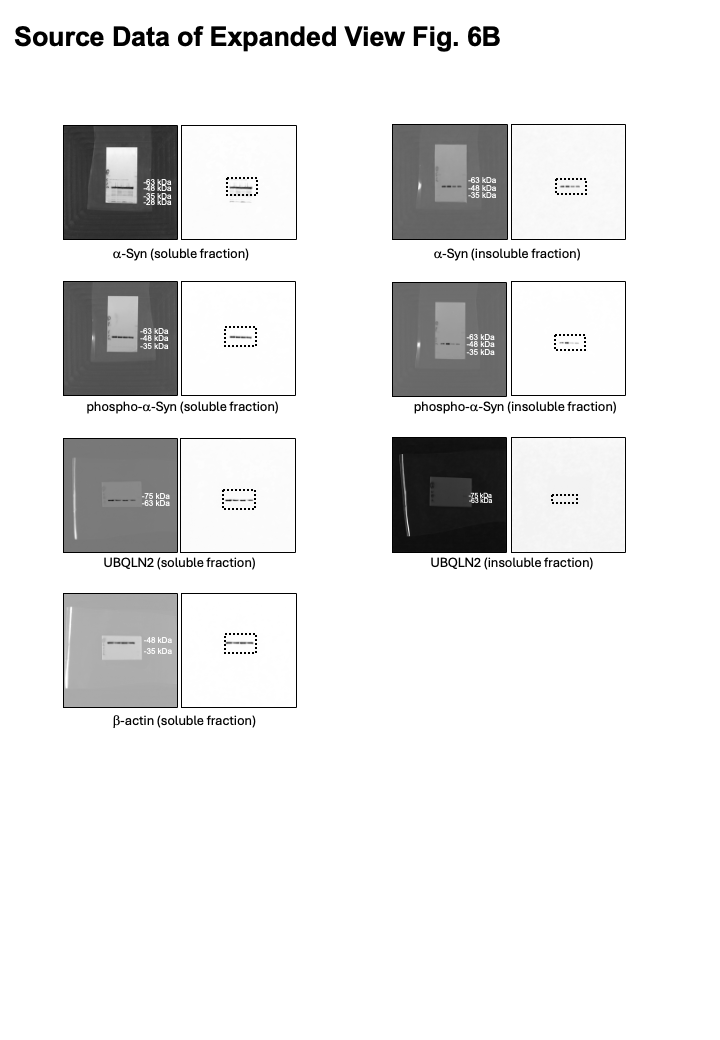

Supplement: Supplementary file 9 — EV Figures Source Data [file 44318_2025_591_MOESM9_ESM.zip › EMBOJ-2025-121908R1_SourceDataForEV/Expanded View Figure 6/EV6B/08_Highlight of crop area.tiff]

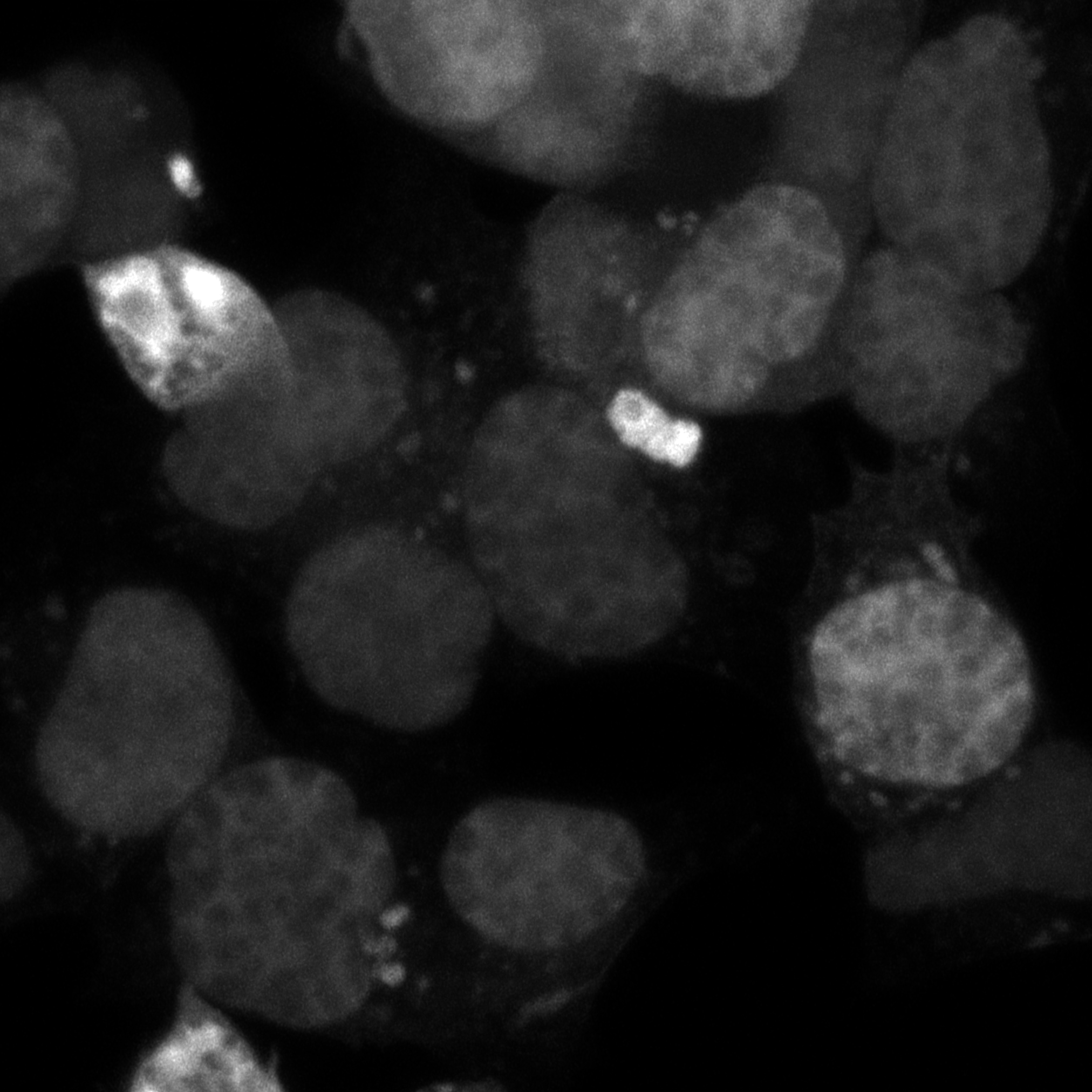

Supplement: Supplementary file 9 — EV Figures Source Data [file 44318_2025_591_MOESM9_ESM.zip › EMBOJ-2025-121908R1_SourceDataForEV/Expanded View Figure 6/EV6A/(a)_04_AsNaO2_╬▒-Syn-EGFP_UBQLN2_DAPI.tif]

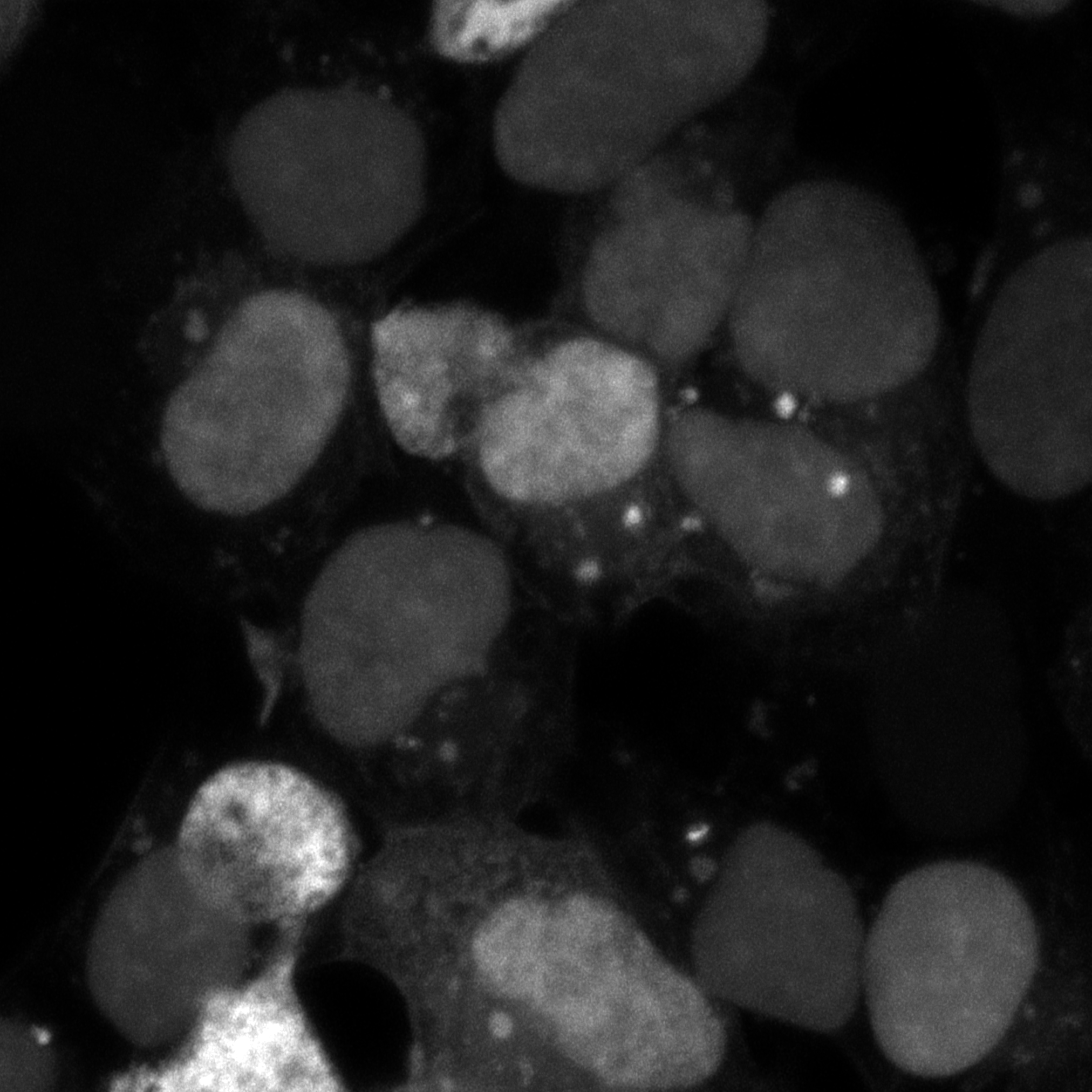

Supplement: Supplementary file 9 — EV Figures Source Data [file 44318_2025_591_MOESM9_ESM.zip › EMBOJ-2025-121908R1_SourceDataForEV/Expanded View Figure 6/EV6A/(a)_05_AsNaO2+SO286_╬▒-Syn-EGFP_UBQLN2_DAPI.tif]

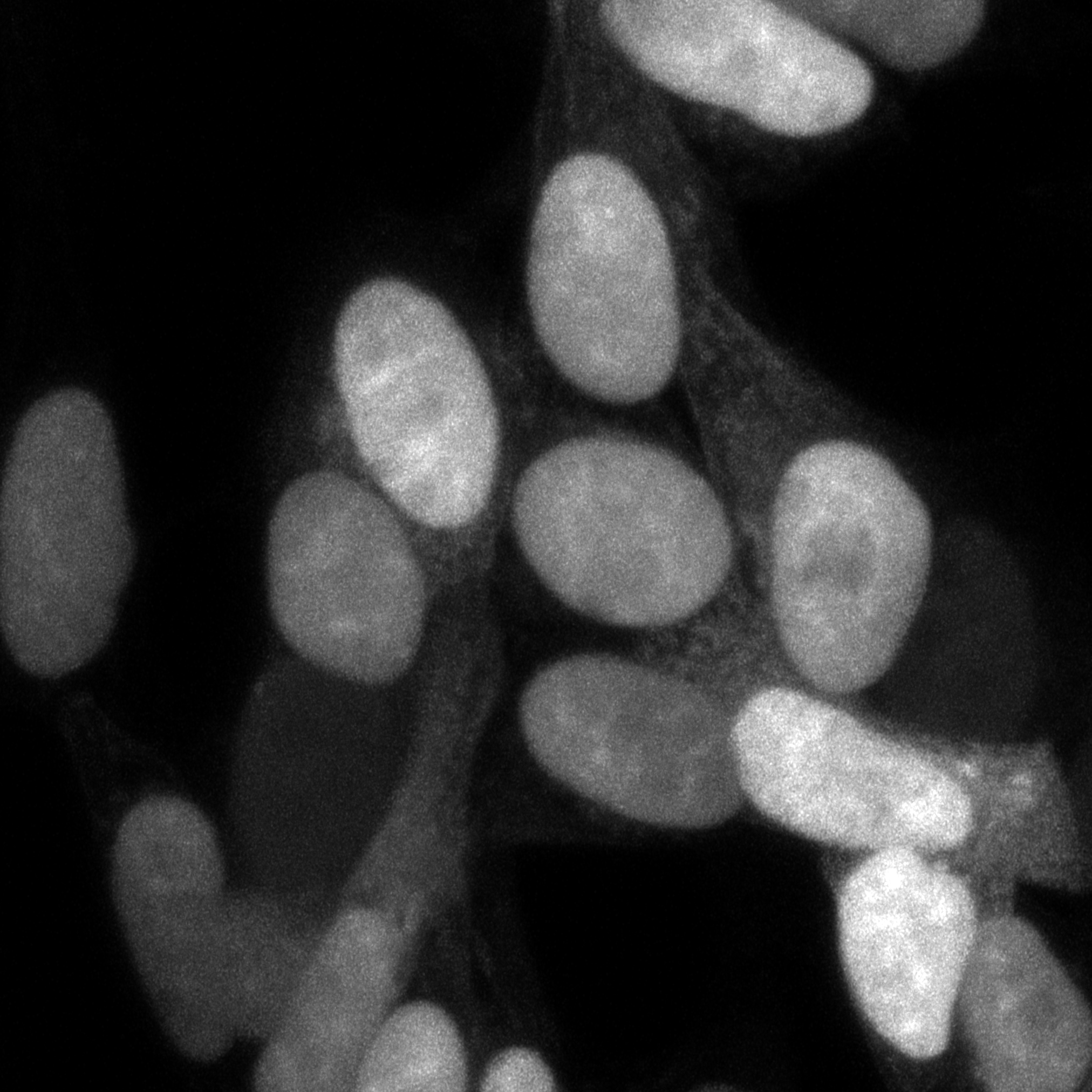

Supplement: Supplementary file 9 — EV Figures Source Data [file 44318_2025_591_MOESM9_ESM.zip › EMBOJ-2025-121908R1_SourceDataForEV/Expanded View Figure 6/EV6A/(a)_01_DMSO_╬▒-Syn-EGFP_UBQLN2_DAPI.tif]

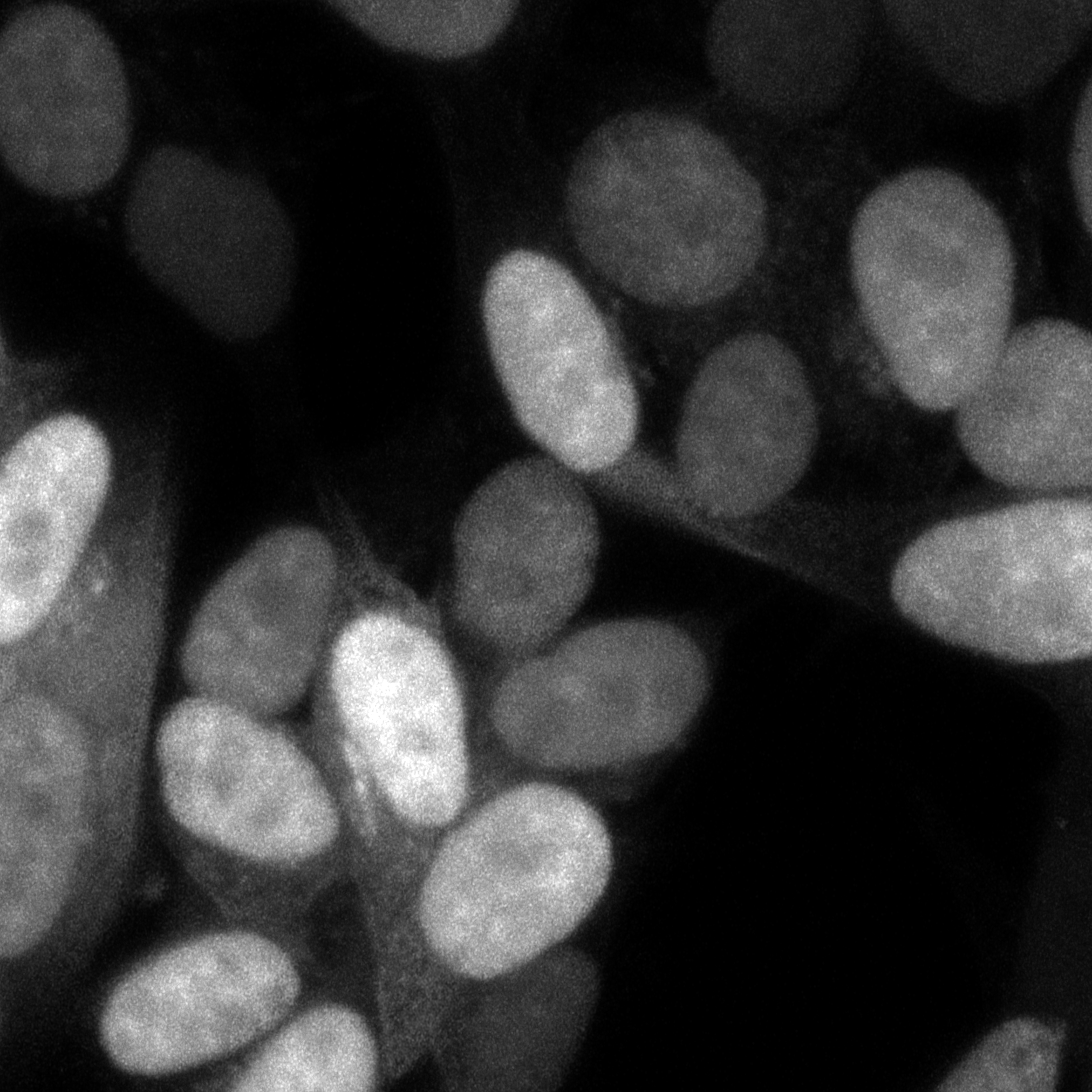

Supplement: Supplementary file 9 — EV Figures Source Data [file 44318_2025_591_MOESM9_ESM.zip › EMBOJ-2025-121908R1_SourceDataForEV/Expanded View Figure 6/EV6A/(a)_02_SO286_╬▒-Syn-EGFP_UBQLN2_DAPI.tif]

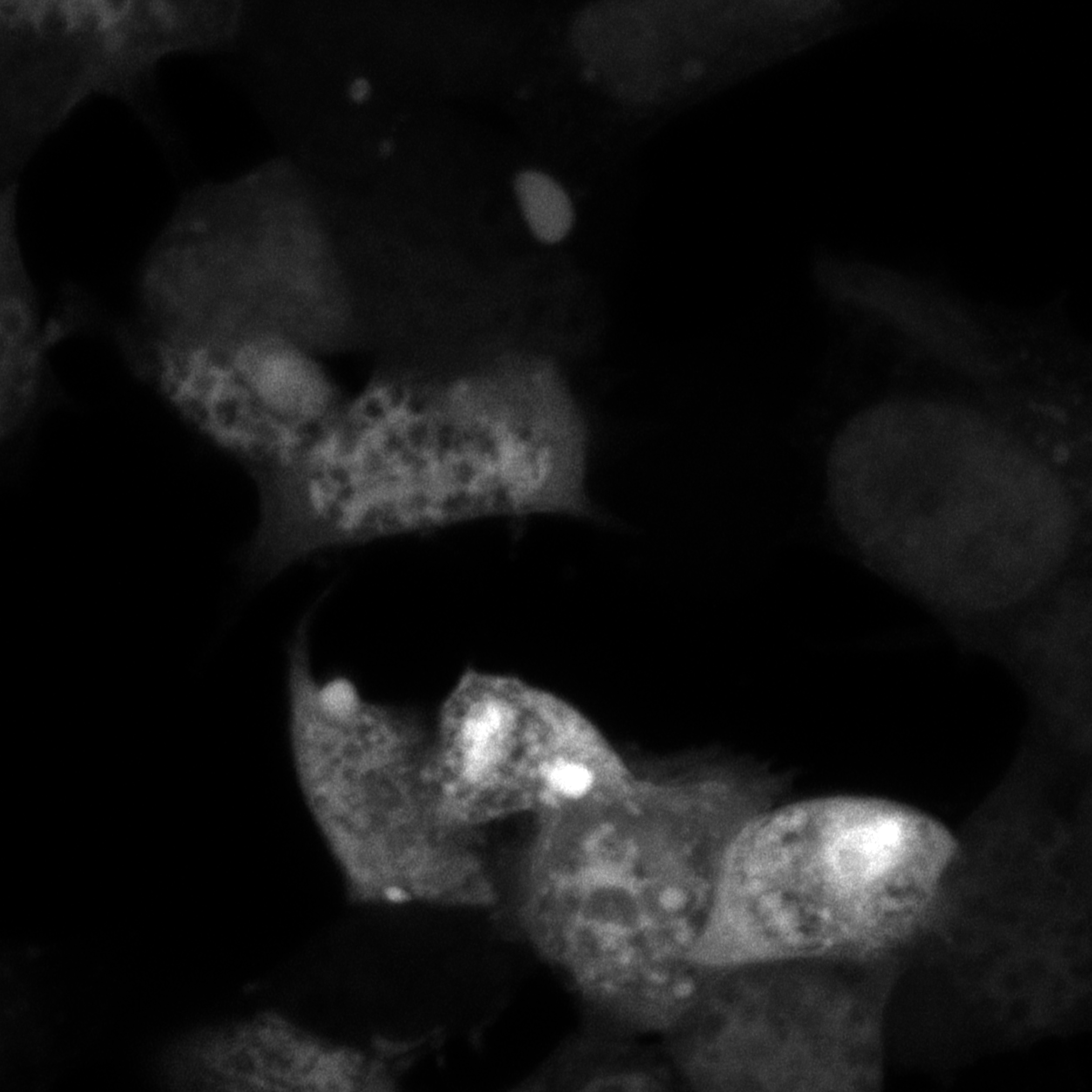

Supplement: Supplementary file 9 — EV Figures Source Data [file 44318_2025_591_MOESM9_ESM.zip › EMBOJ-2025-121908R1_SourceDataForEV/Expanded View Figure 6/EV6A/(a)_06_AsNaO2+SO82_╬▒-Syn-EGFP_UBQLN2_DAPI.tif]

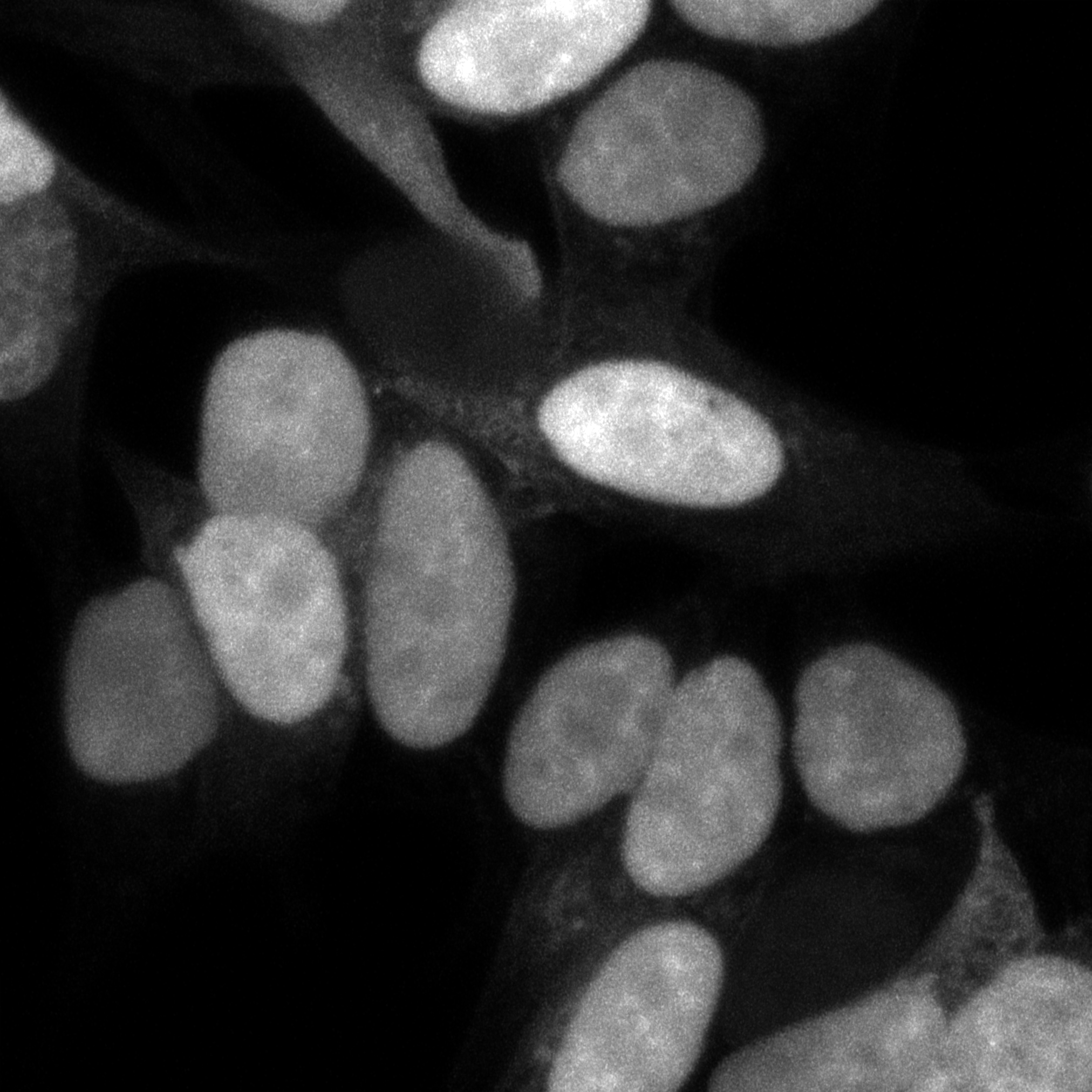

Supplement: Supplementary file 9 — EV Figures Source Data [file 44318_2025_591_MOESM9_ESM.zip › EMBOJ-2025-121908R1_SourceDataForEV/Expanded View Figure 6/EV6A/(a)_03_SO82_╬▒-Syn-EGFP_UBQLN2_DAPI.tif]

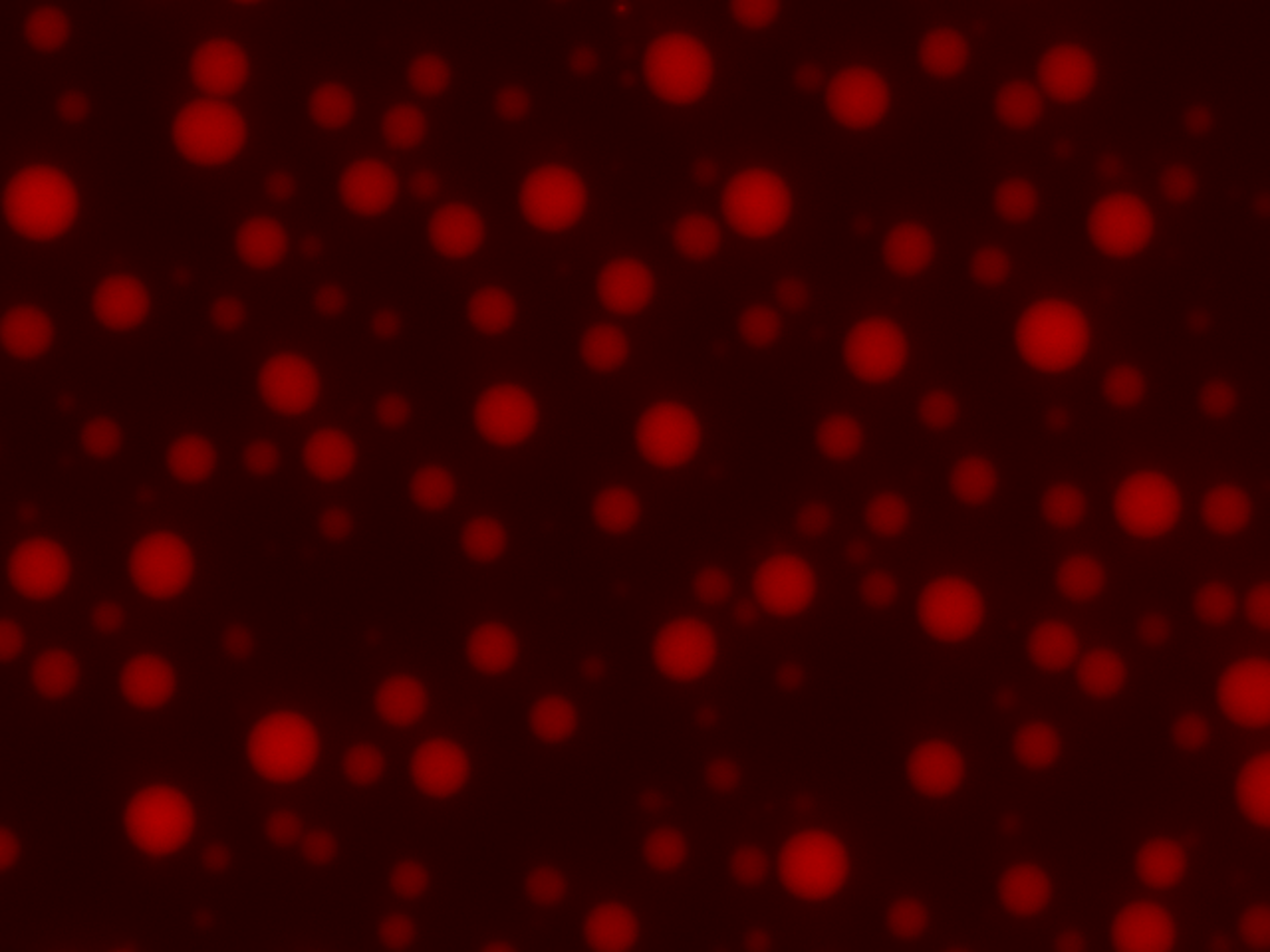

Supplement: Supplementary file 9 — EV Figures Source Data [file 44318_2025_591_MOESM9_ESM.zip › EMBOJ-2025-121908R1_SourceDataForEV/Expanded View Figure 1/EV1C/10_24 h_None_╬▒-Syn(UBQLN1+╬▒-Syn).tif]

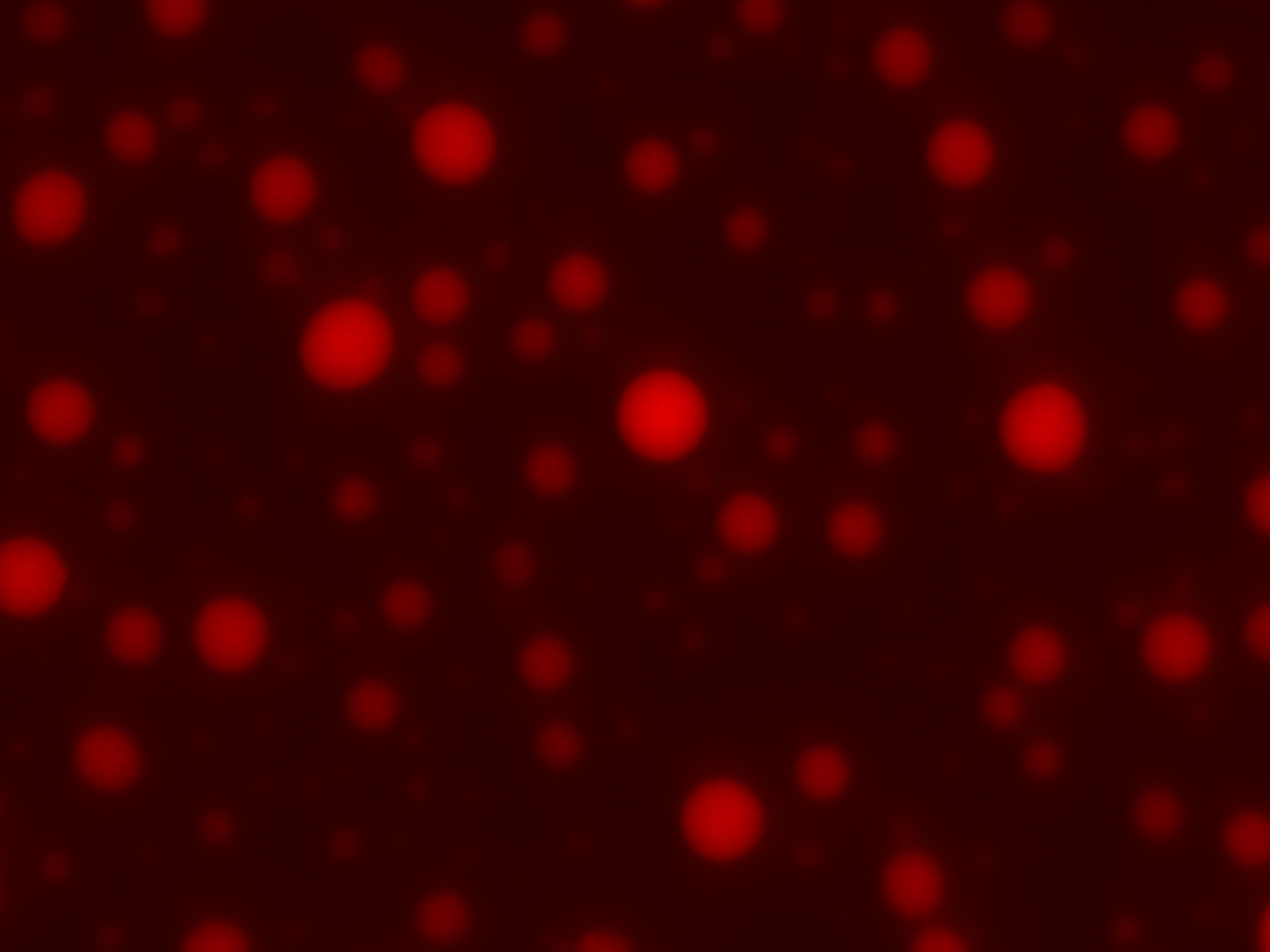

Supplement: Supplementary file 9 — EV Figures Source Data [file 44318_2025_591_MOESM9_ESM.zip › EMBOJ-2025-121908R1_SourceDataForEV/Expanded View Figure 1/EV1C/14_96 h_None_╬▒-Syn(UBQLN1+╬▒-Syn).tif]

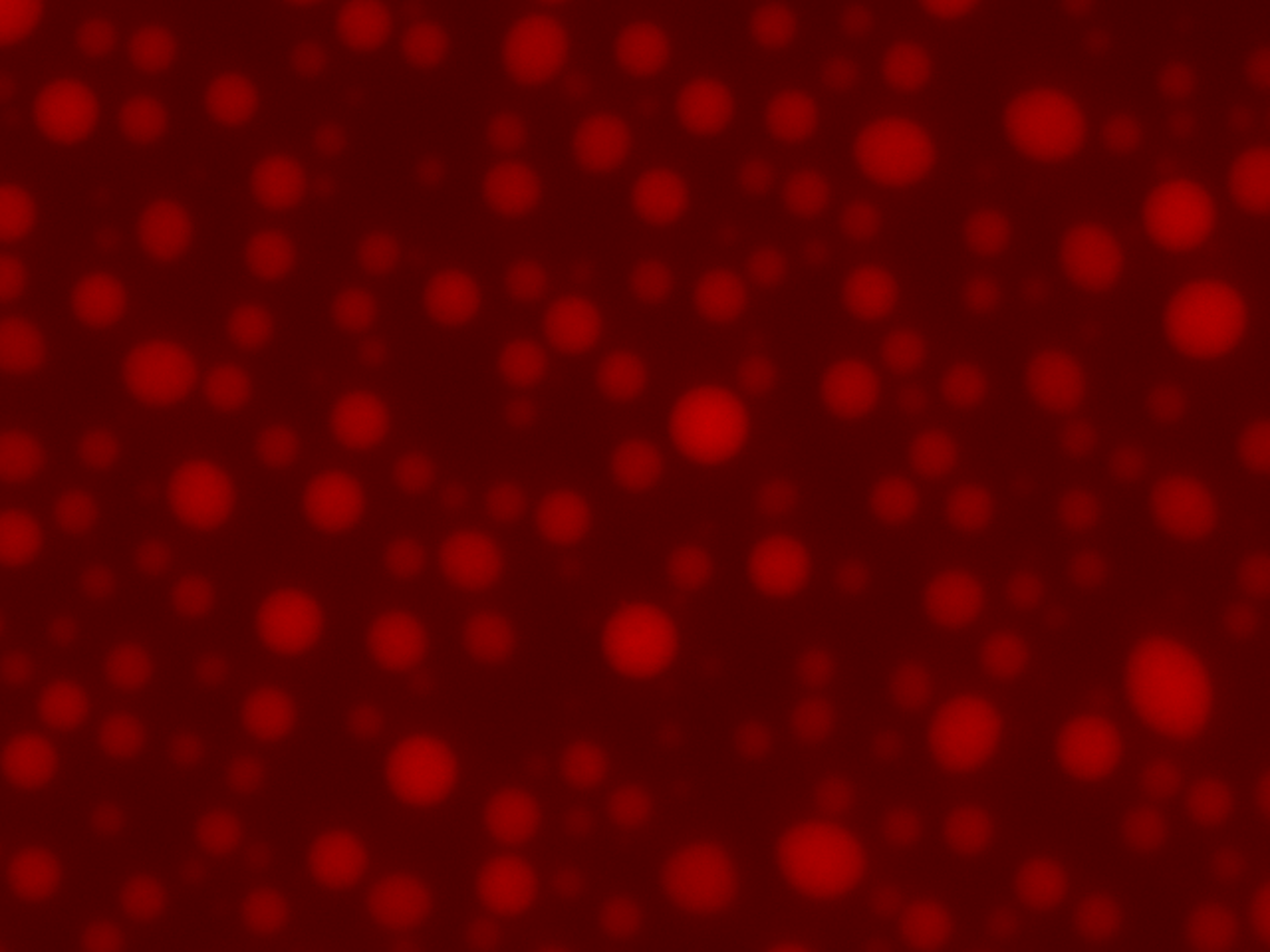

Supplement: Supplementary file 9 — EV Figures Source Data [file 44318_2025_591_MOESM9_ESM.zip › EMBOJ-2025-121908R1_SourceDataForEV/Expanded View Figure 1/EV1C/22_96 h_None_╬▒-Syn(UBQLN4+╬▒-Syn).tif]

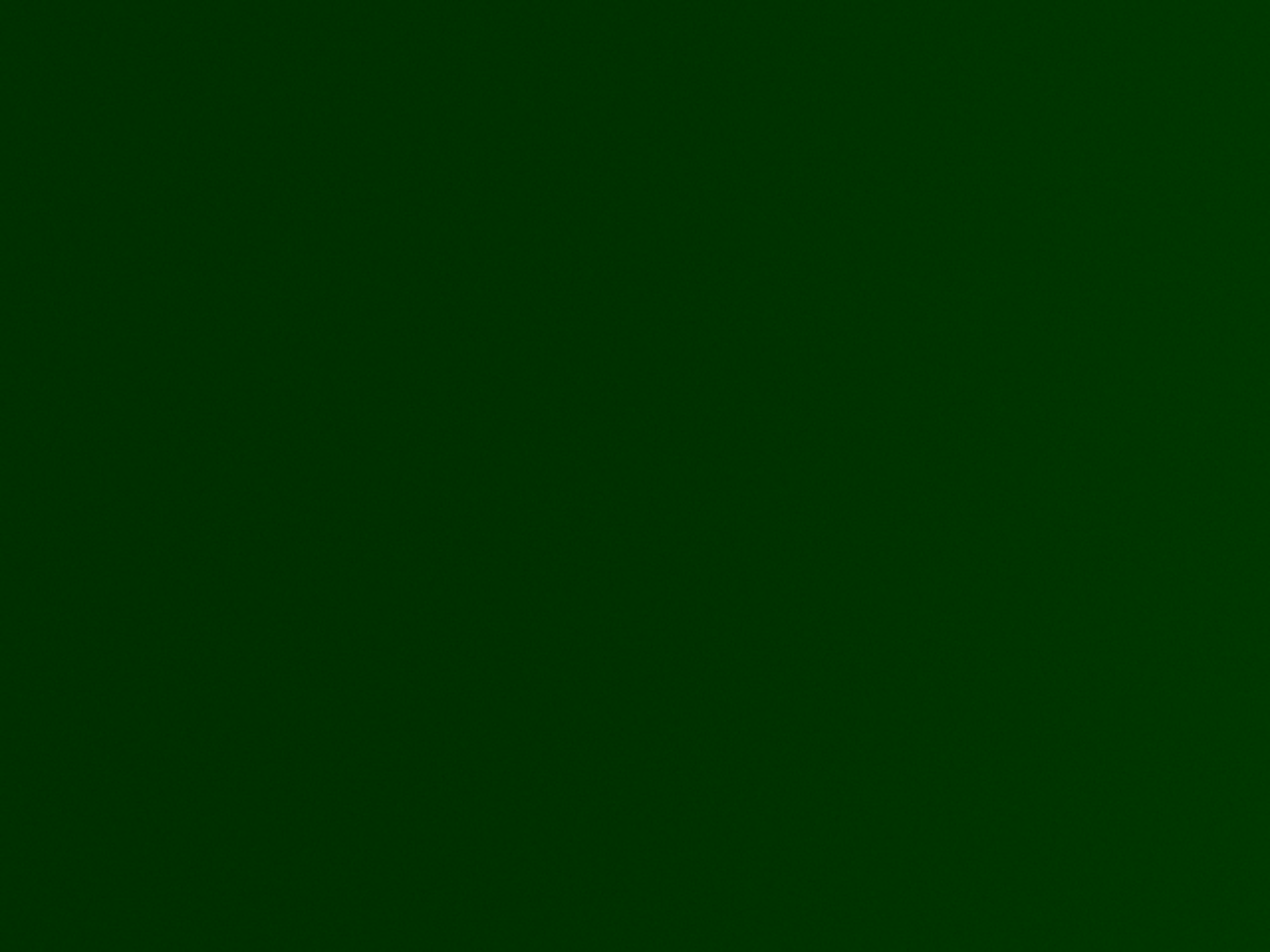

Supplement: Supplementary file 9 — EV Figures Source Data [file 44318_2025_591_MOESM9_ESM.zip › EMBOJ-2025-121908R1_SourceDataForEV/Expanded View Figure 1/EV1C/03_24 h_1,6-HD_UBQLN2(UBQLN2+╬▒-Syn).tif]

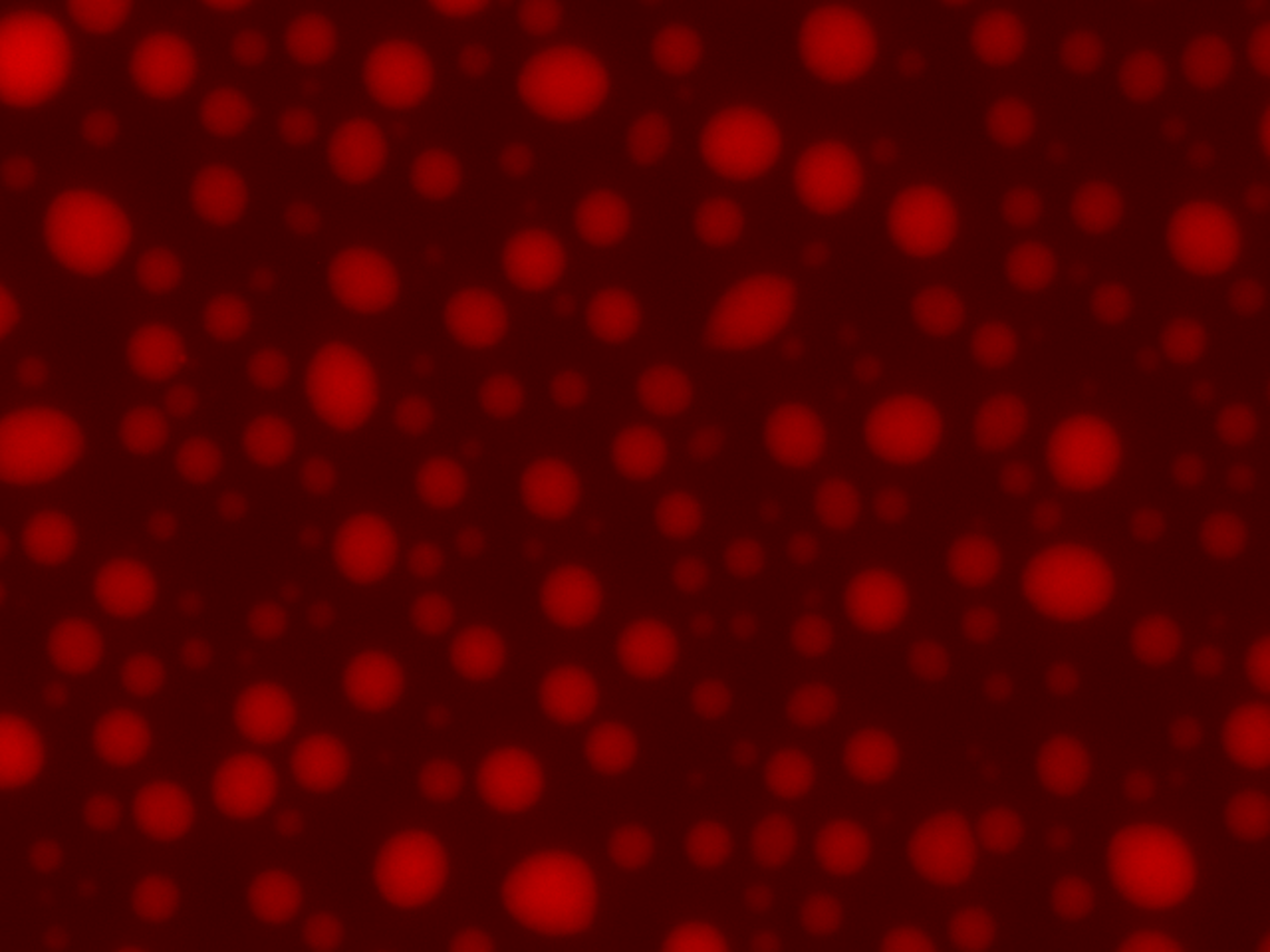

Supplement: Supplementary file 9 — EV Figures Source Data [file 44318_2025_591_MOESM9_ESM.zip › EMBOJ-2025-121908R1_SourceDataForEV/Expanded View Figure 1/EV1C/20_24 h_1,6-HD_╬▒-Syn(UBQLN4+╬▒-Syn).tif]

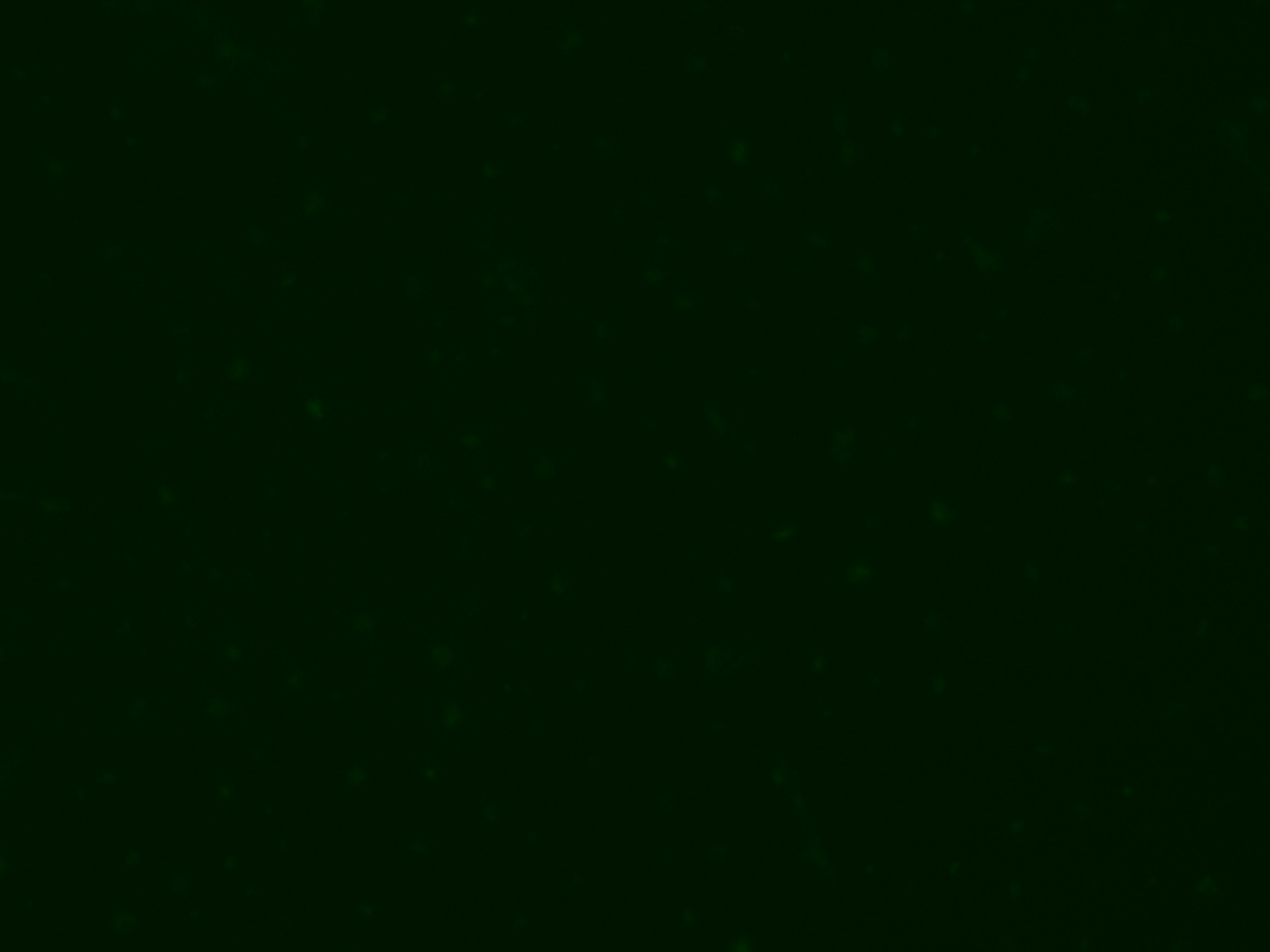

Supplement: Supplementary file 9 — EV Figures Source Data [file 44318_2025_591_MOESM9_ESM.zip › EMBOJ-2025-121908R1_SourceDataForEV/Expanded View Figure 1/EV1C/11_24 h_1,6-HD_UBQLN1(UBQLN1+╬▒-Syn).tif]

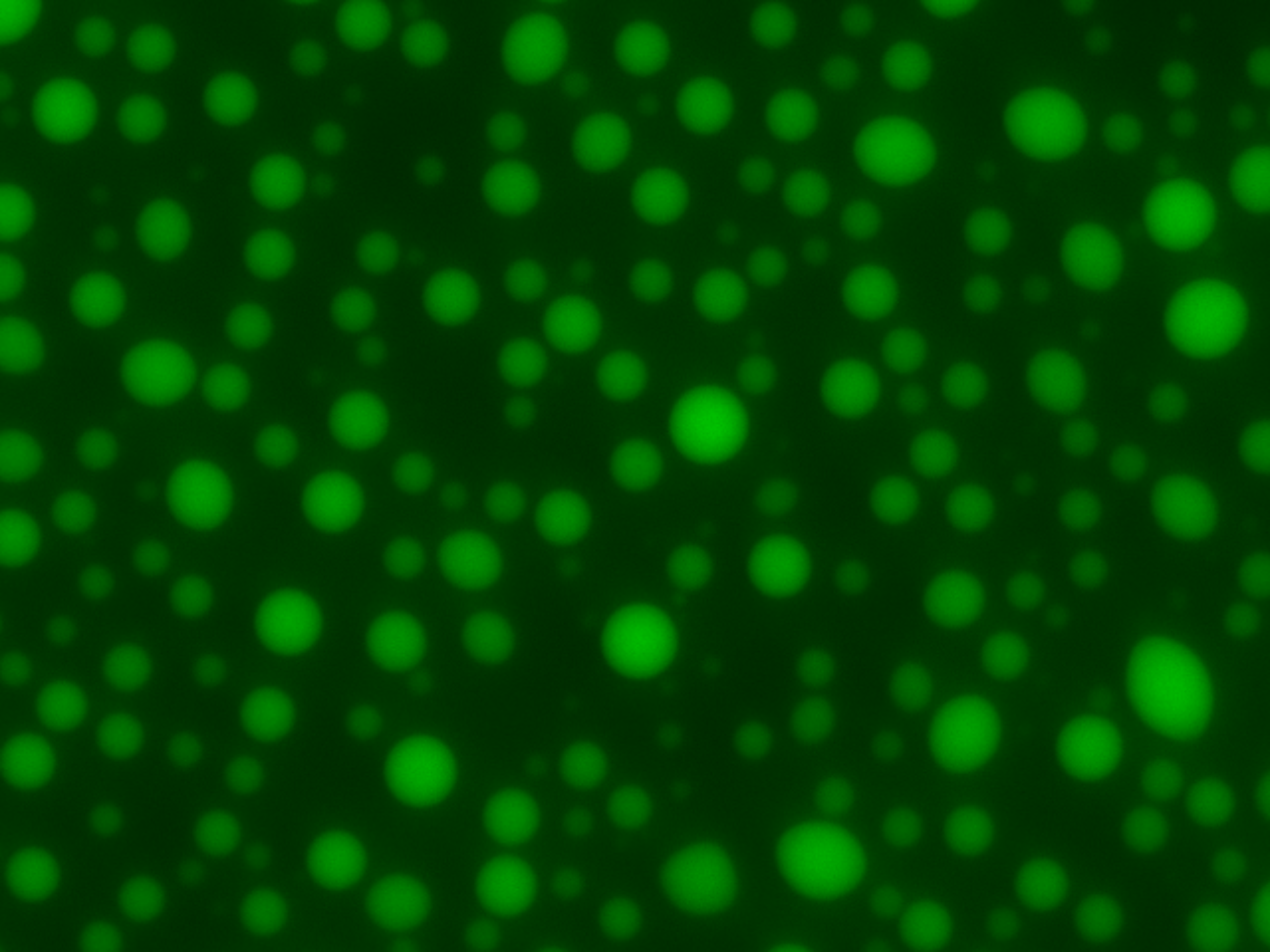

Supplement: Supplementary file 9 — EV Figures Source Data [file 44318_2025_591_MOESM9_ESM.zip › EMBOJ-2025-121908R1_SourceDataForEV/Expanded View Figure 1/EV1C/21_96 h_None_UBQLN4(UBQLN4+╬▒-Syn).tif]

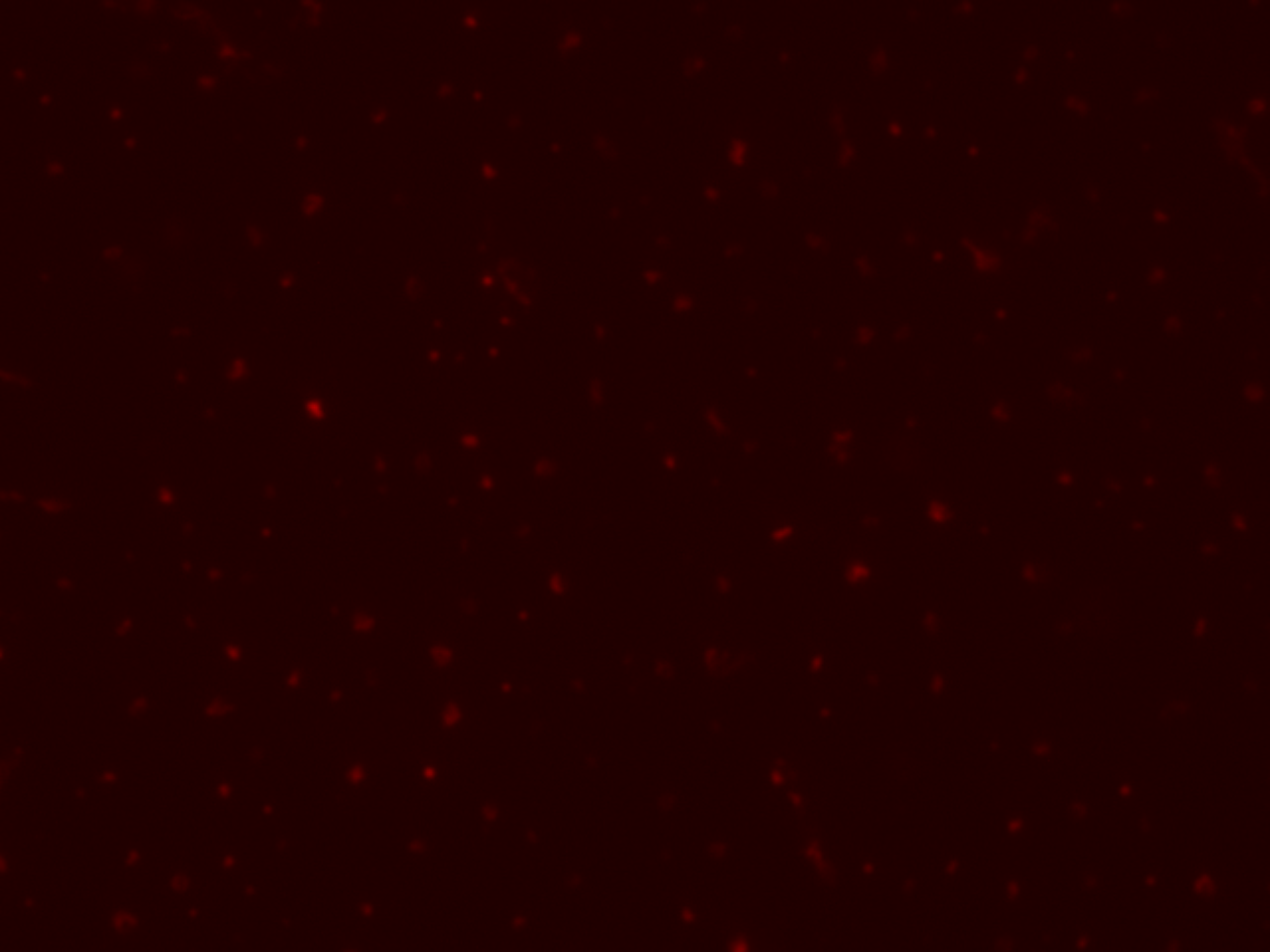

Supplement: Supplementary file 9 — EV Figures Source Data [file 44318_2025_591_MOESM9_ESM.zip › EMBOJ-2025-121908R1_SourceDataForEV/Expanded View Figure 1/EV1C/12_24 h_1,6-HD_╬▒-Syn(UBQLN1+╬▒-Syn).tif]

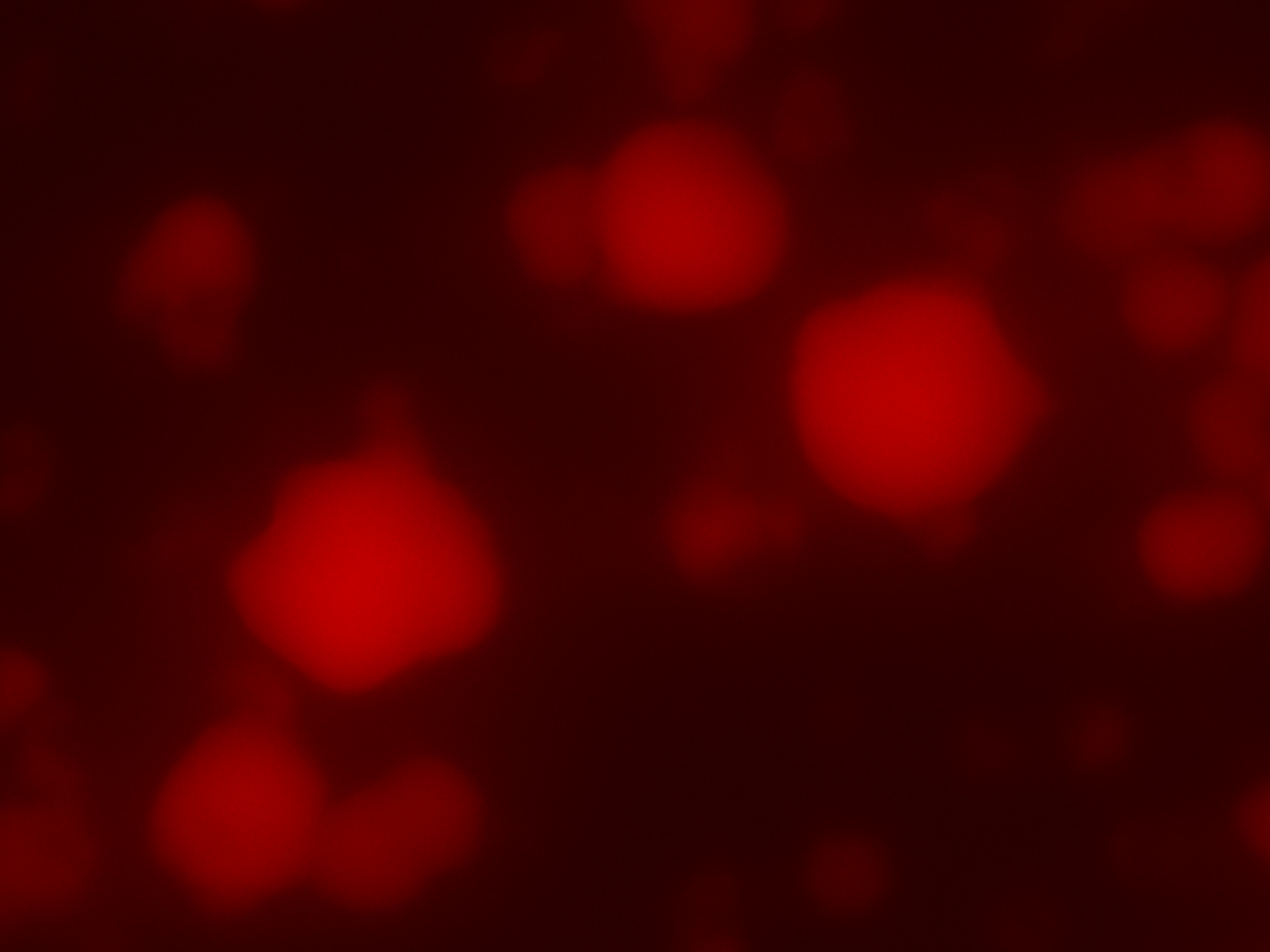

Supplement: Supplementary file 9 — EV Figures Source Data [file 44318_2025_591_MOESM9_ESM.zip › EMBOJ-2025-121908R1_SourceDataForEV/Expanded View Figure 1/EV1C/06_96 h_None_╬▒-Syn(UBQLN2+╬▒-Syn).tif]

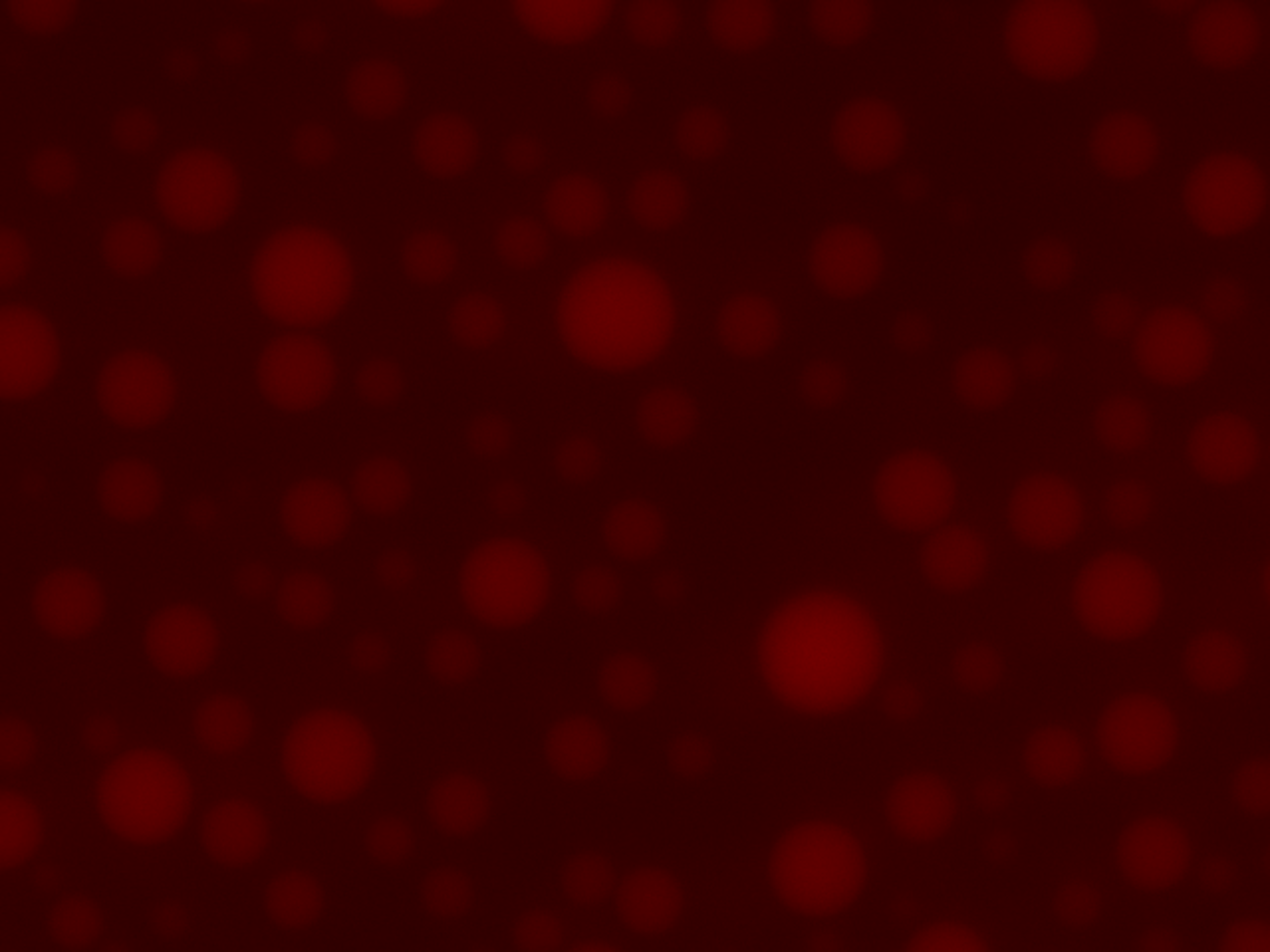

Supplement: Supplementary file 9 — EV Figures Source Data [file 44318_2025_591_MOESM9_ESM.zip › EMBOJ-2025-121908R1_SourceDataForEV/Expanded View Figure 1/EV1C/02_24 h_None_╬▒-Syn(UBQLN2+╬▒-Syn).tif]

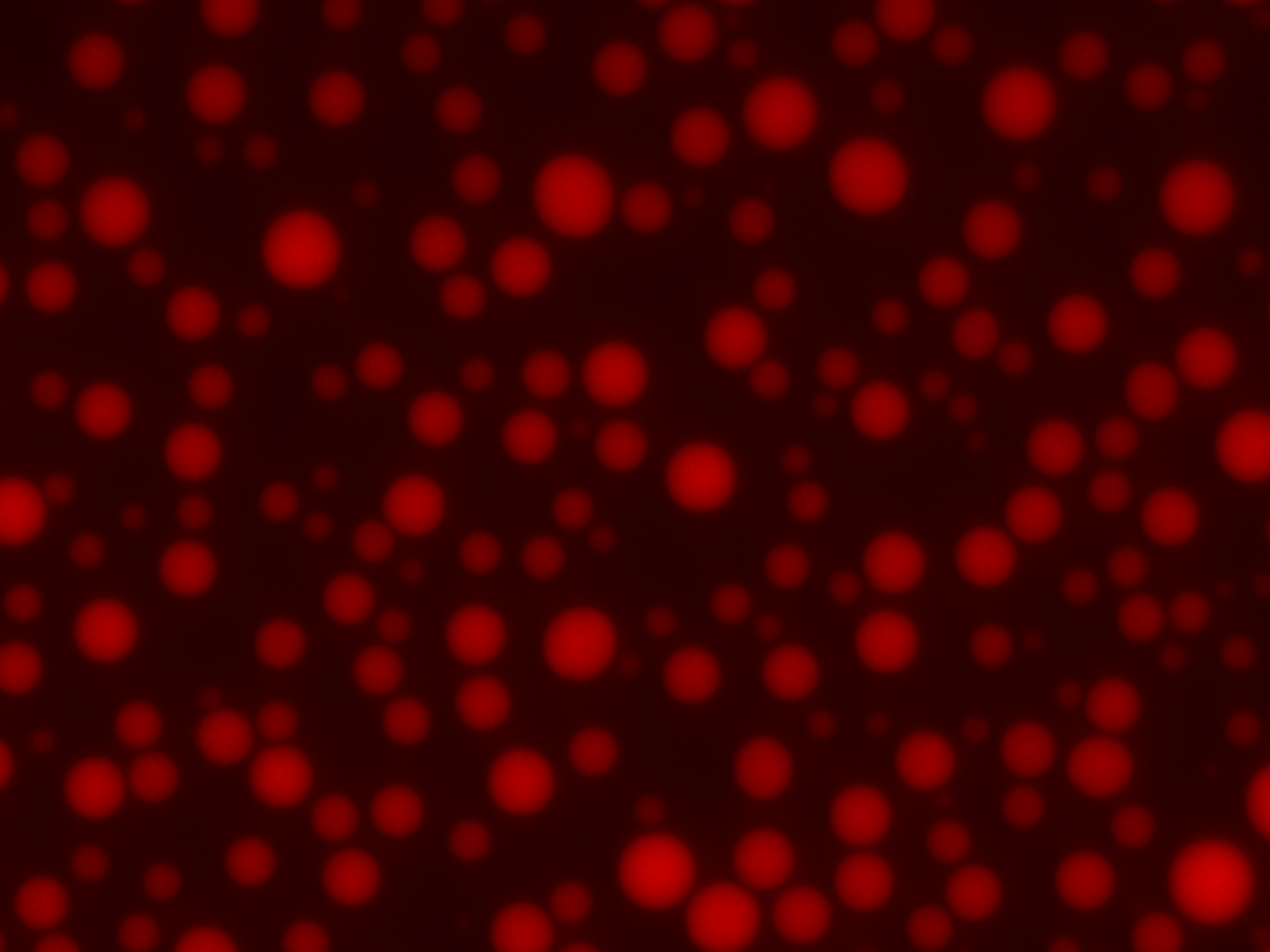

Supplement: Supplementary file 9 — EV Figures Source Data [file 44318_2025_591_MOESM9_ESM.zip › EMBOJ-2025-121908R1_SourceDataForEV/Expanded View Figure 1/EV1C/18_24 h_None_╬▒-Syn(UBQLN4+╬▒-Syn).tif]

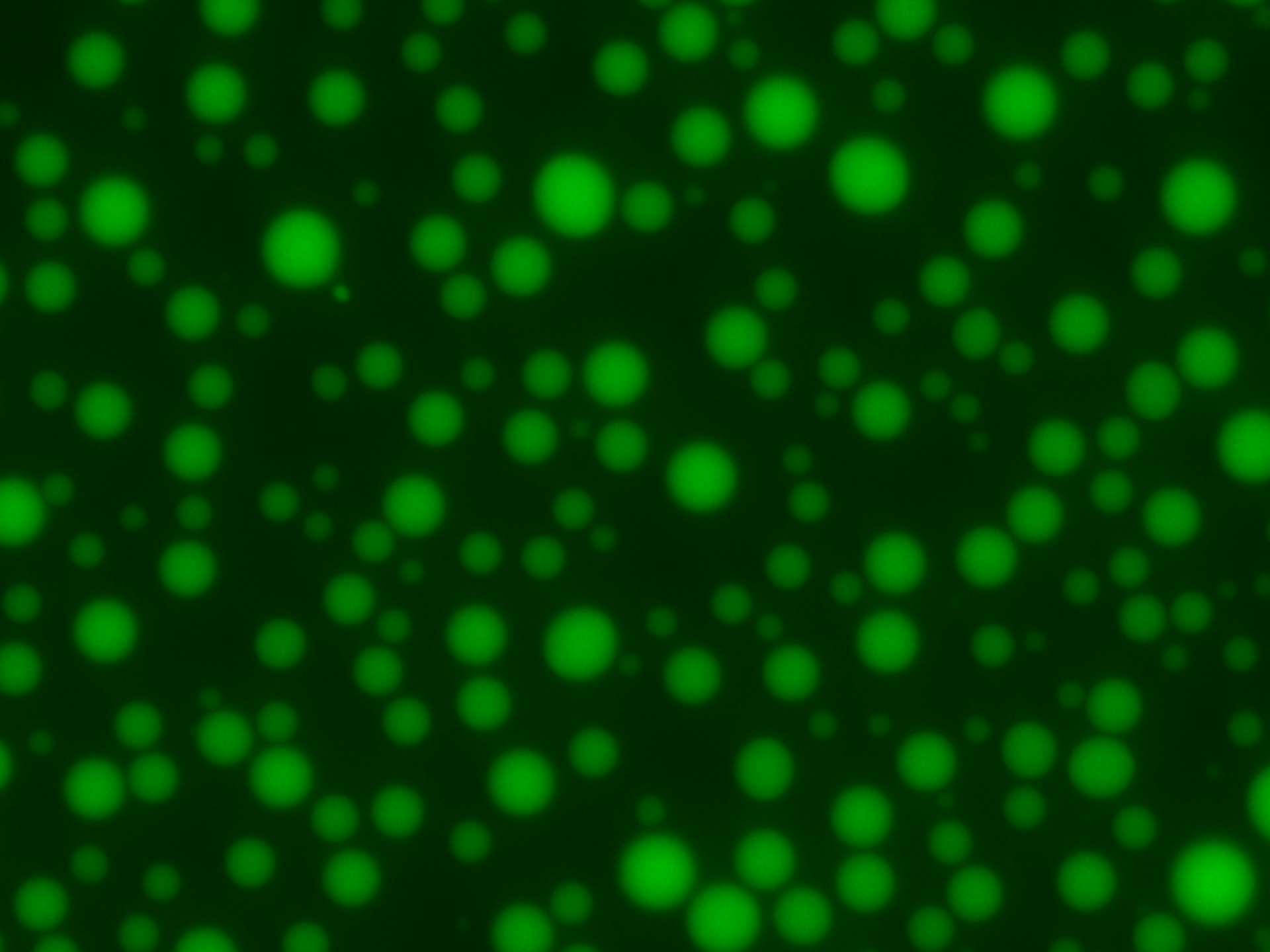

Supplement: Supplementary file 9 — EV Figures Source Data [file 44318_2025_591_MOESM9_ESM.zip › EMBOJ-2025-121908R1_SourceDataForEV/Expanded View Figure 1/EV1C/17_24 h_None_UBQLN4(UBQLN4+╬▒-Syn).tif]

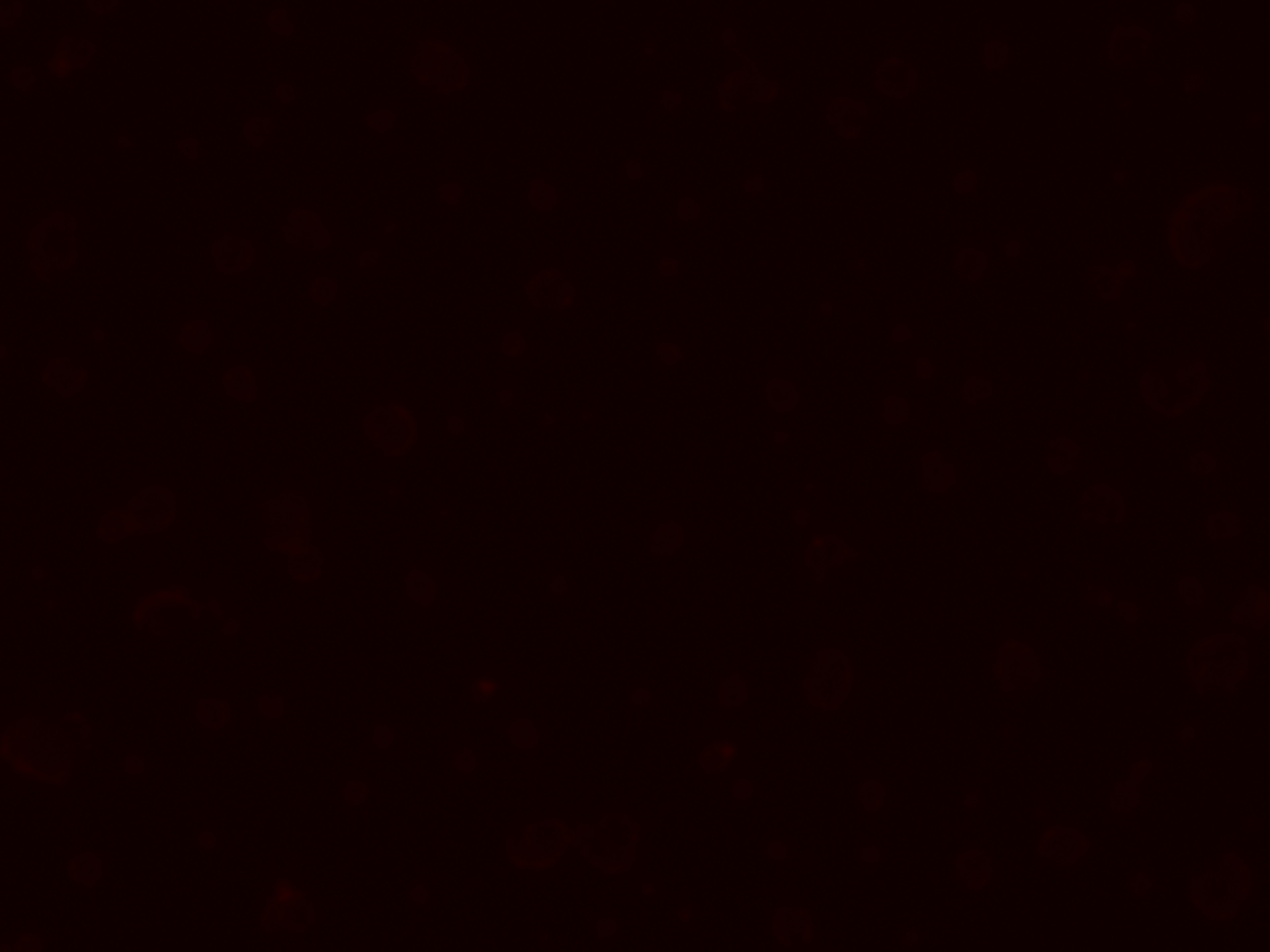

Supplement: Supplementary file 9 — EV Figures Source Data [file 44318_2025_591_MOESM9_ESM.zip › EMBOJ-2025-121908R1_SourceDataForEV/Expanded View Figure 1/EV1C/16_96 h_1,6-HD_╬▒-Syn(UBQLN1+╬▒-Syn).tif]

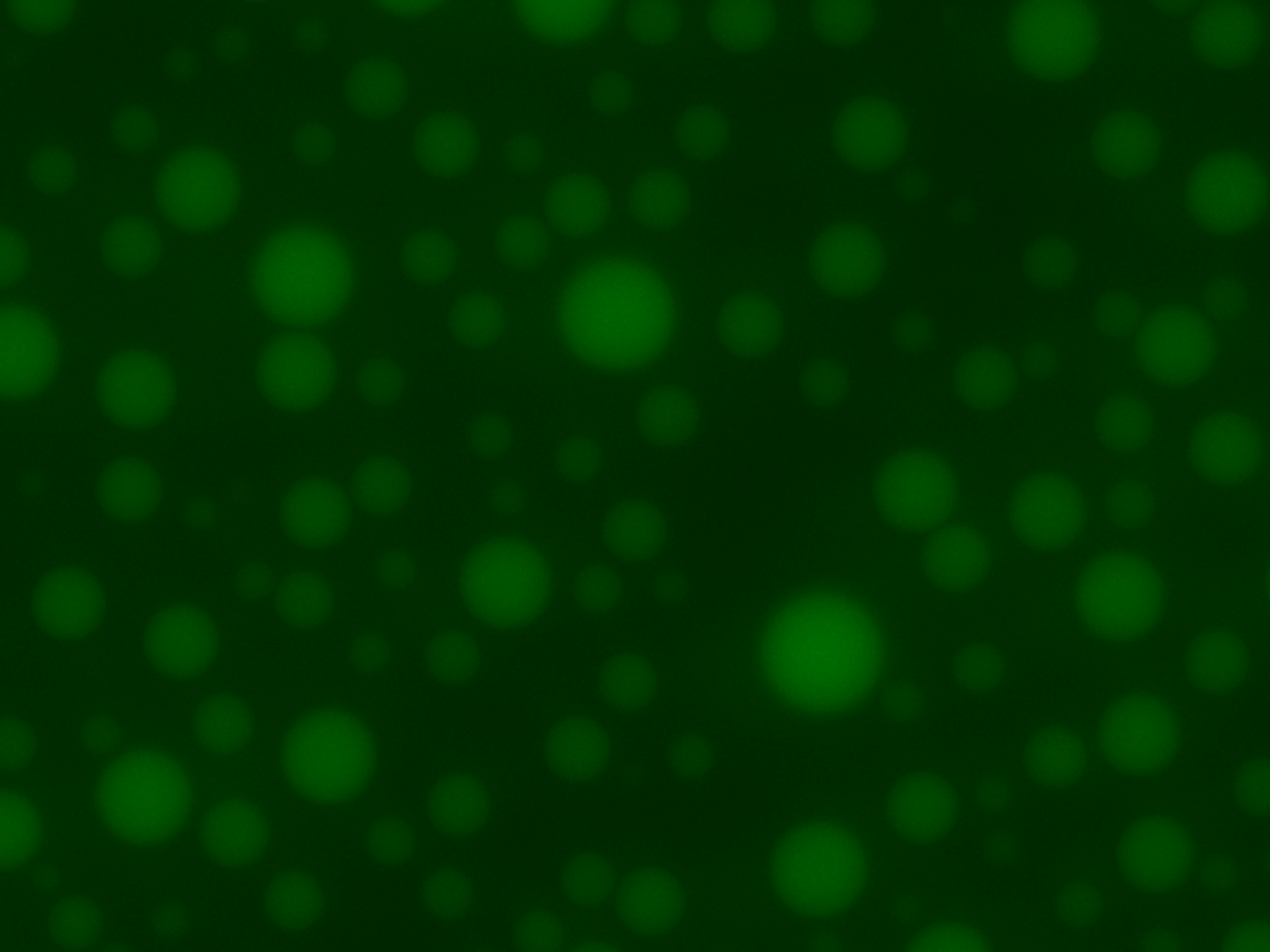

Supplement: Supplementary file 9 — EV Figures Source Data [file 44318_2025_591_MOESM9_ESM.zip › EMBOJ-2025-121908R1_SourceDataForEV/Expanded View Figure 1/EV1C/01_24 h_None_UBQLN2(UBQLN2+╬▒-Syn).tif]

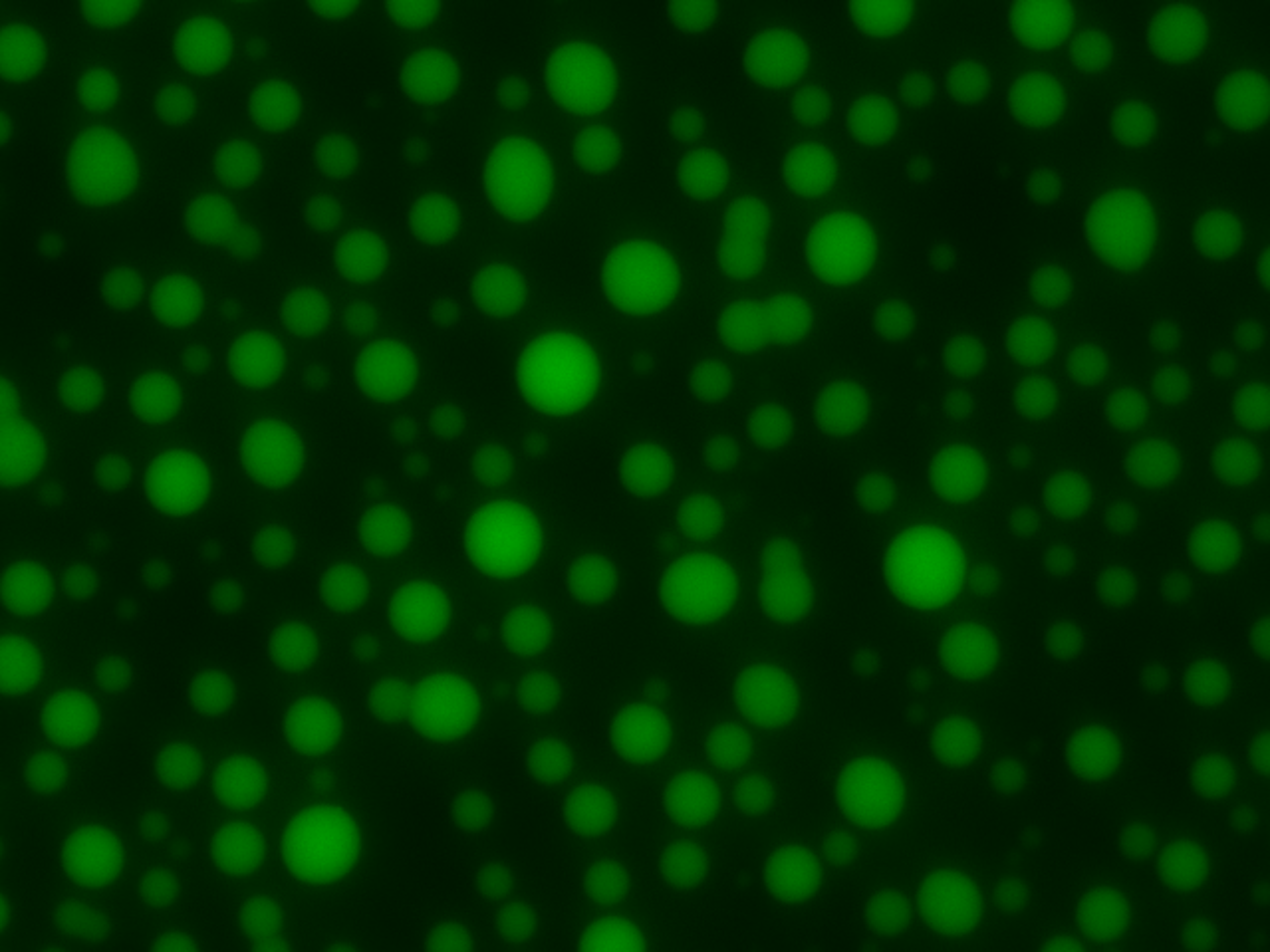

Supplement: Supplementary file 9 — EV Figures Source Data [file 44318_2025_591_MOESM9_ESM.zip › EMBOJ-2025-121908R1_SourceDataForEV/Expanded View Figure 1/EV1C/23_96 h_1,6-HD_UBQLN4(UBQLN4+╬▒-Syn).tif]

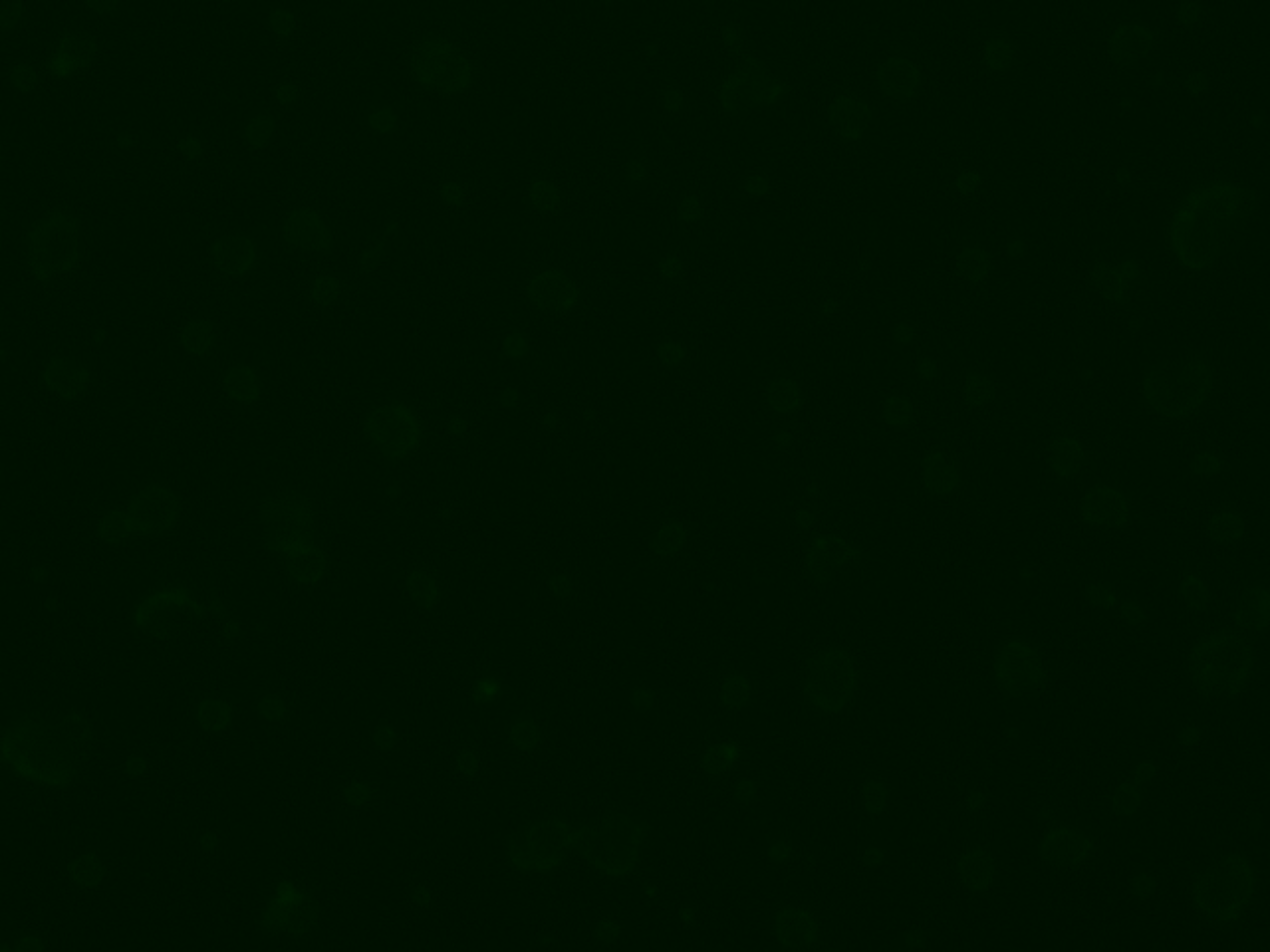

Supplement: Supplementary file 9 — EV Figures Source Data [file 44318_2025_591_MOESM9_ESM.zip › EMBOJ-2025-121908R1_SourceDataForEV/Expanded View Figure 1/EV1C/15_96 h_1,6-HD_UBQLN1(UBQLN1+╬▒-Syn).tif]

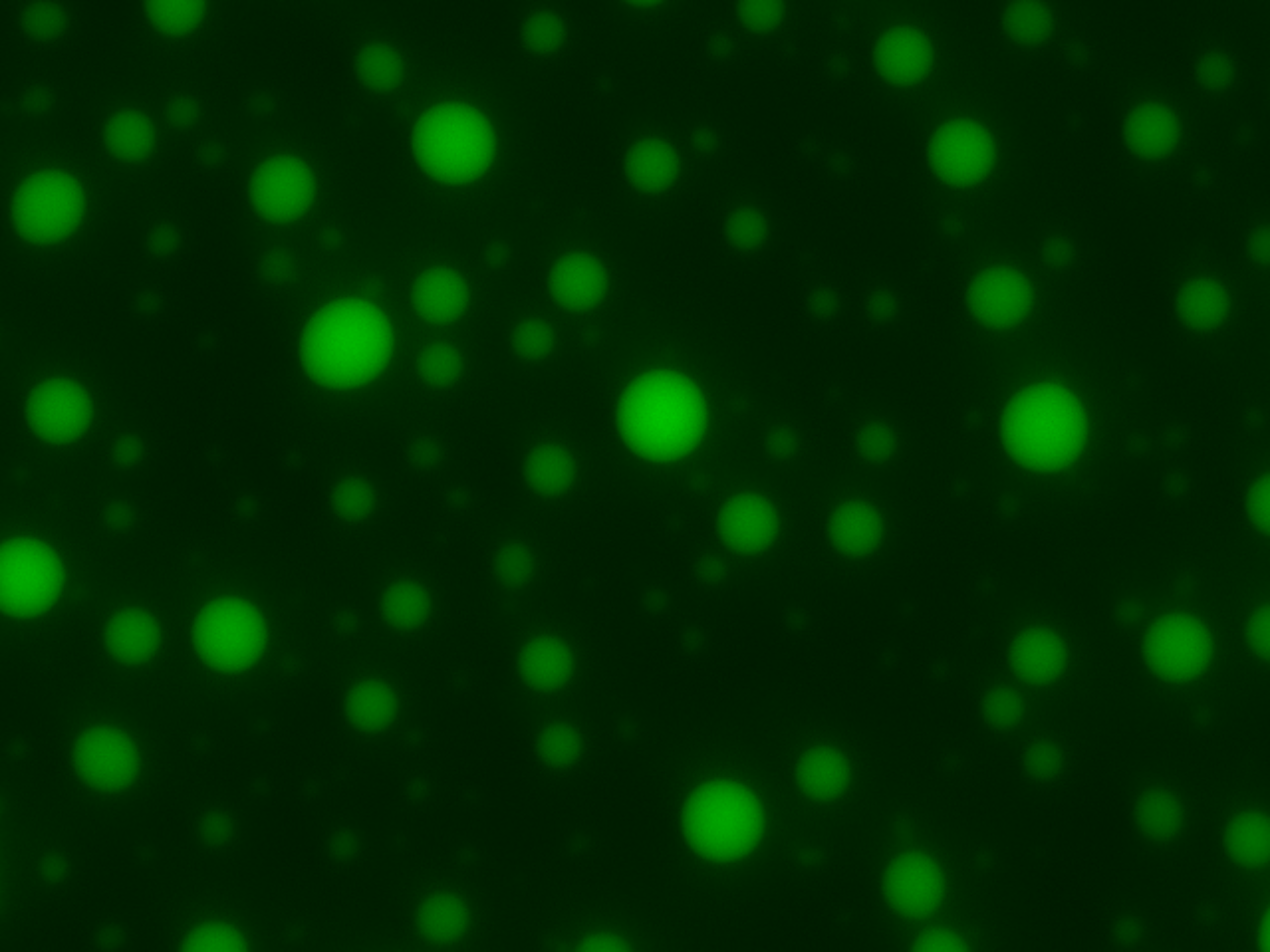

Supplement: Supplementary file 9 — EV Figures Source Data [file 44318_2025_591_MOESM9_ESM.zip › EMBOJ-2025-121908R1_SourceDataForEV/Expanded View Figure 1/EV1C/13_96 h_None_UBQLN1(UBQLN1+╬▒-Syn).tif]

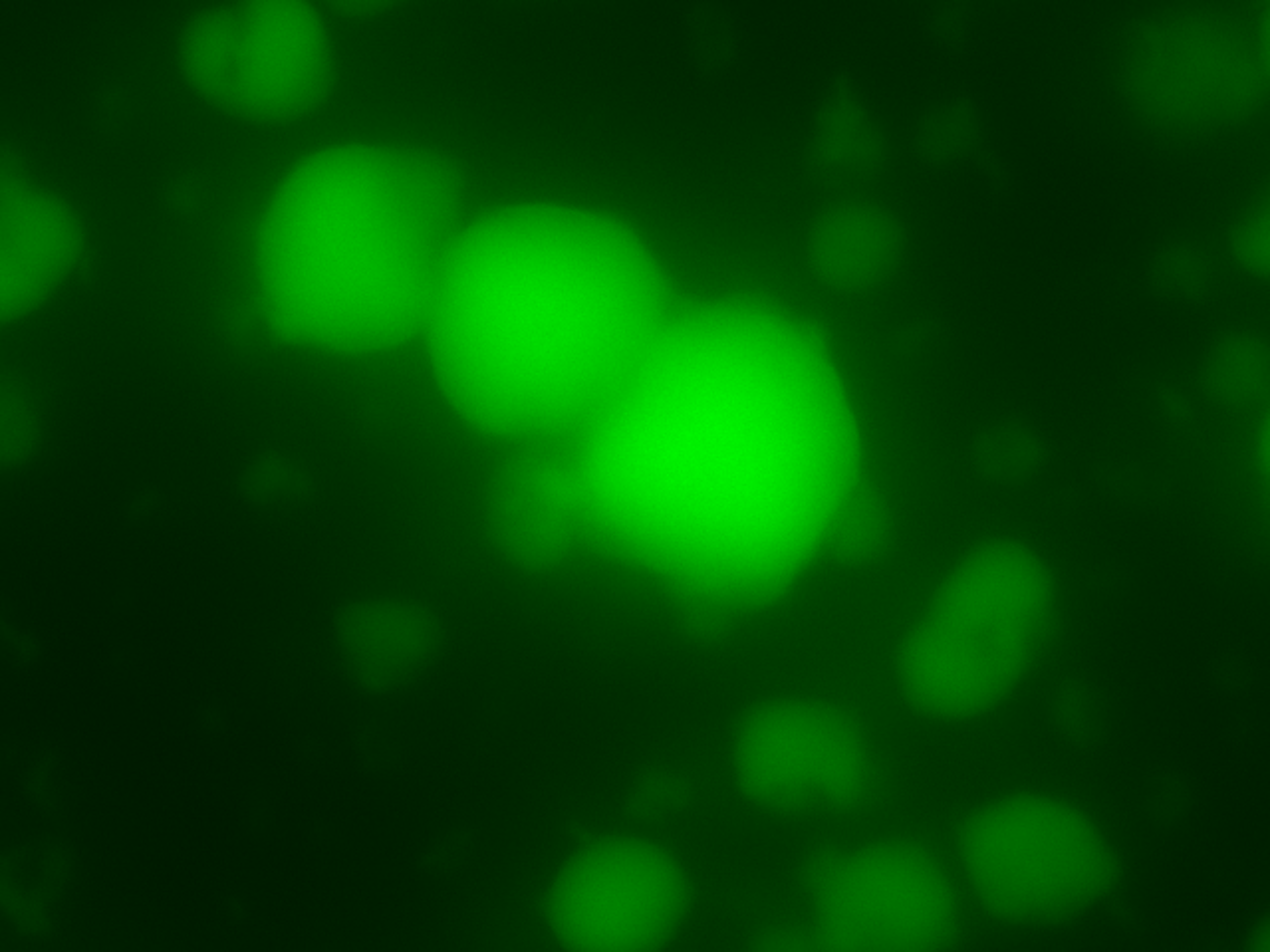

Supplement: Supplementary file 9 — EV Figures Source Data [file 44318_2025_591_MOESM9_ESM.zip › EMBOJ-2025-121908R1_SourceDataForEV/Expanded View Figure 1/EV1C/07_96 h_1,6-HD_UBQLN2(UBQLN2+╬▒-Syn).tif]

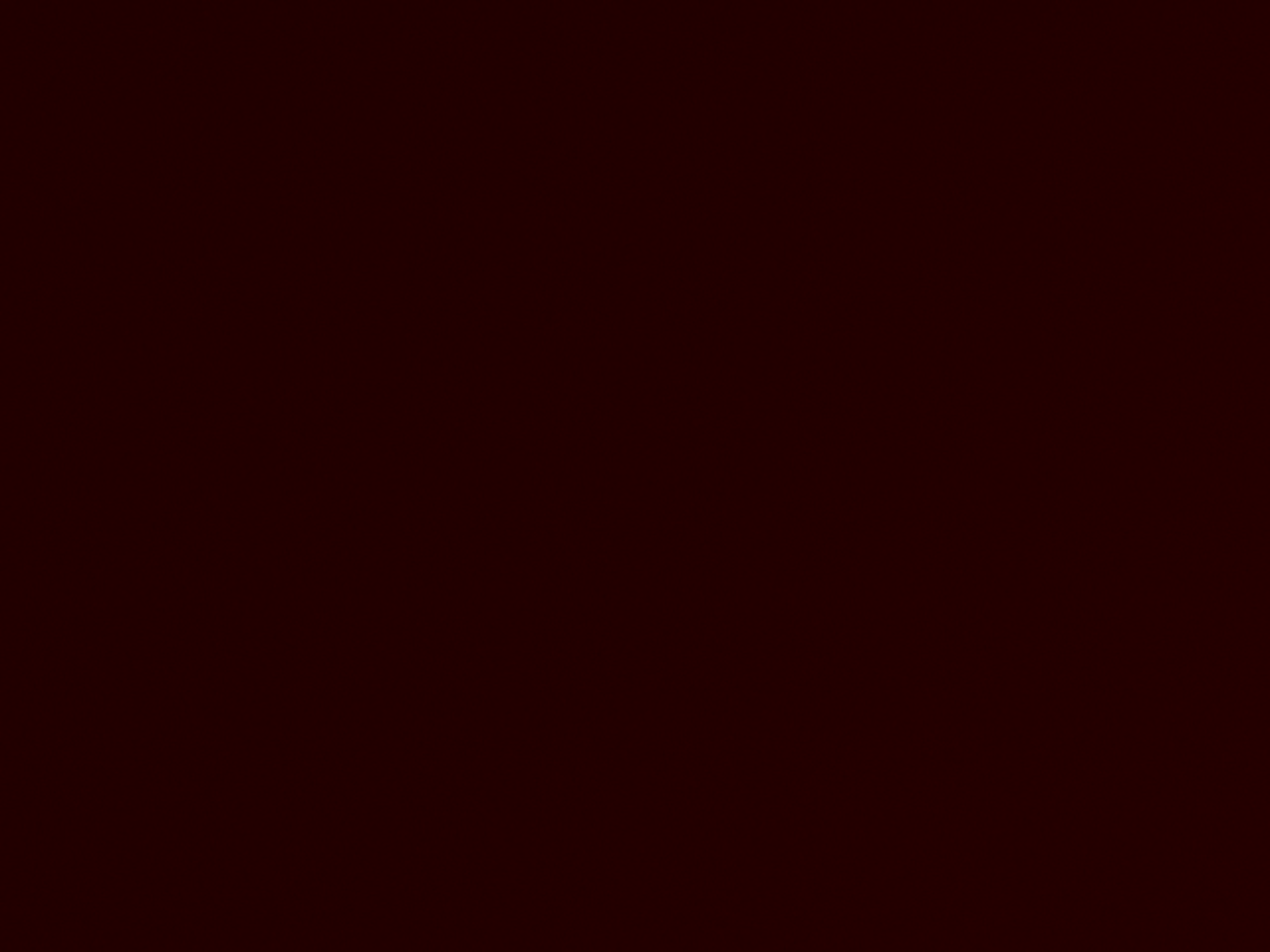

Supplement: Supplementary file 9 — EV Figures Source Data [file 44318_2025_591_MOESM9_ESM.zip › EMBOJ-2025-121908R1_SourceDataForEV/Expanded View Figure 1/EV1C/04_24 h_1,6-HD_╬▒-Syn(UBQLN2+╬▒-Syn).tif]

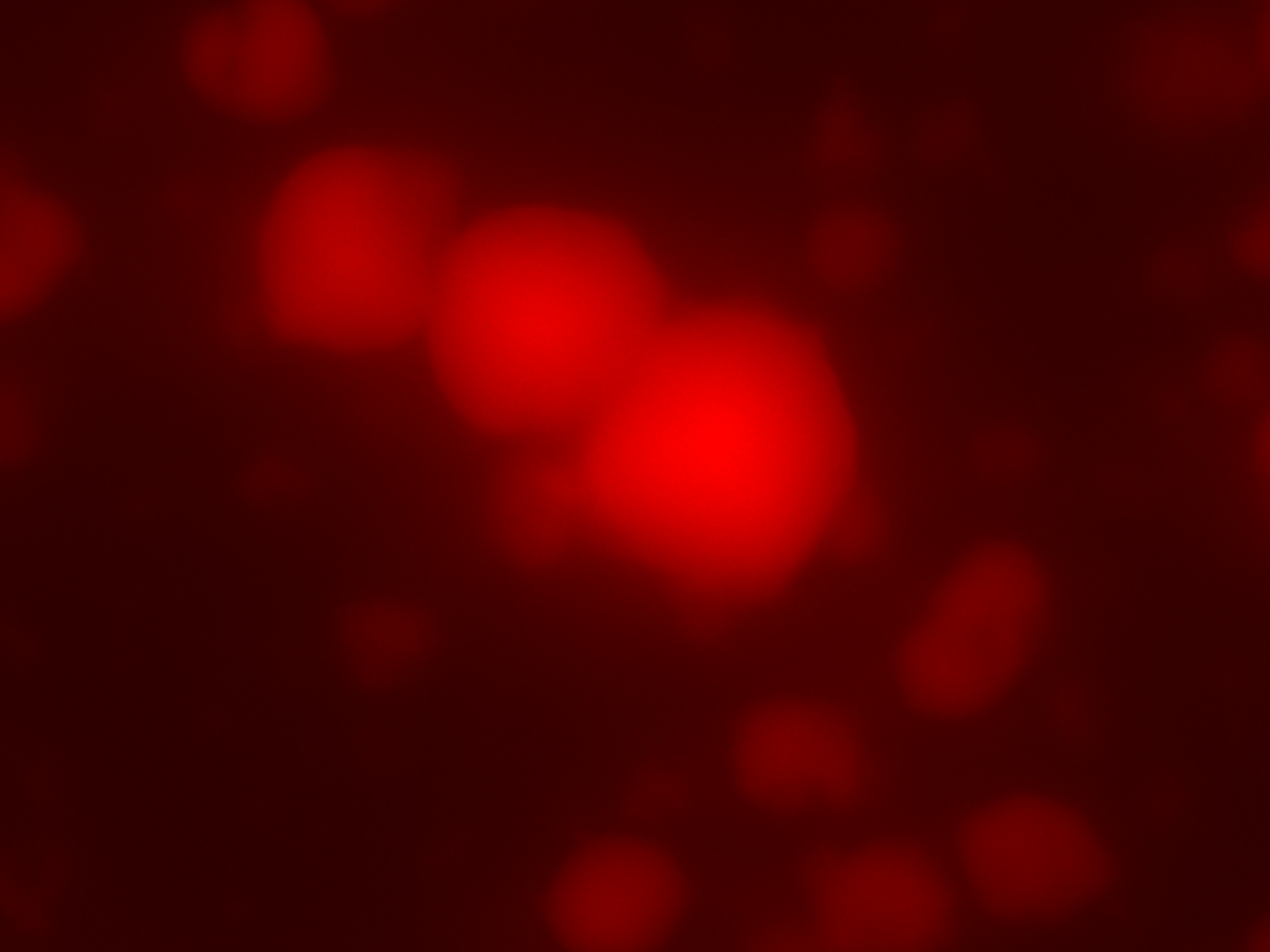

Supplement: Supplementary file 9 — EV Figures Source Data [file 44318_2025_591_MOESM9_ESM.zip › EMBOJ-2025-121908R1_SourceDataForEV/Expanded View Figure 1/EV1C/08_96 h_1,6-HD_╬▒-Syn(UBQLN2+╬▒-Syn).tif]

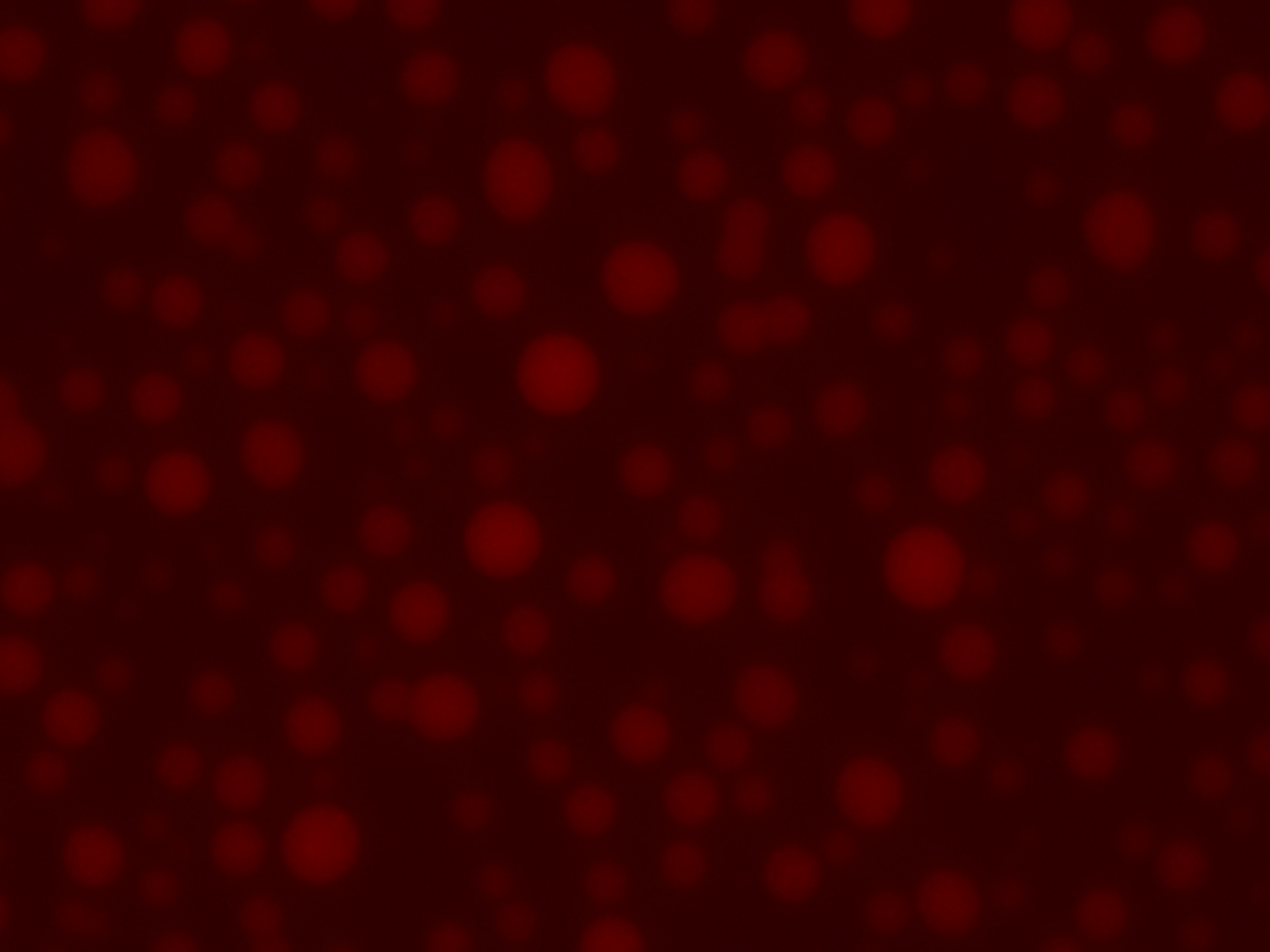

Supplement: Supplementary file 9 — EV Figures Source Data [file 44318_2025_591_MOESM9_ESM.zip › EMBOJ-2025-121908R1_SourceDataForEV/Expanded View Figure 1/EV1C/24_96 h_1,6-HD_╬▒-Syn(UBQLN4+╬▒-Syn).tif]

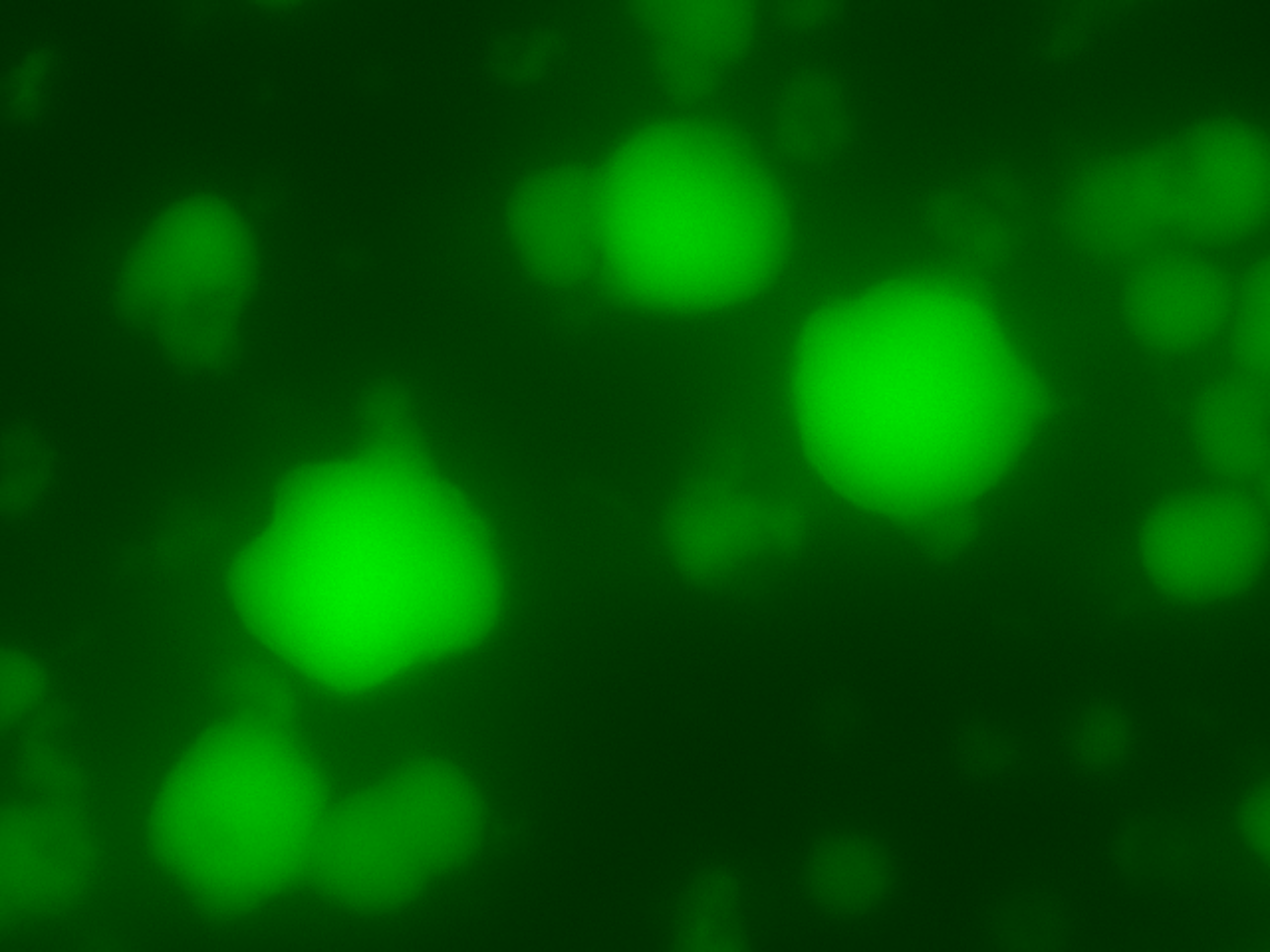

Supplement: Supplementary file 9 — EV Figures Source Data [file 44318_2025_591_MOESM9_ESM.zip › EMBOJ-2025-121908R1_SourceDataForEV/Expanded View Figure 1/EV1C/05_96 h_None_UBQLN2(UBQLN2+╬▒-Syn).tif]

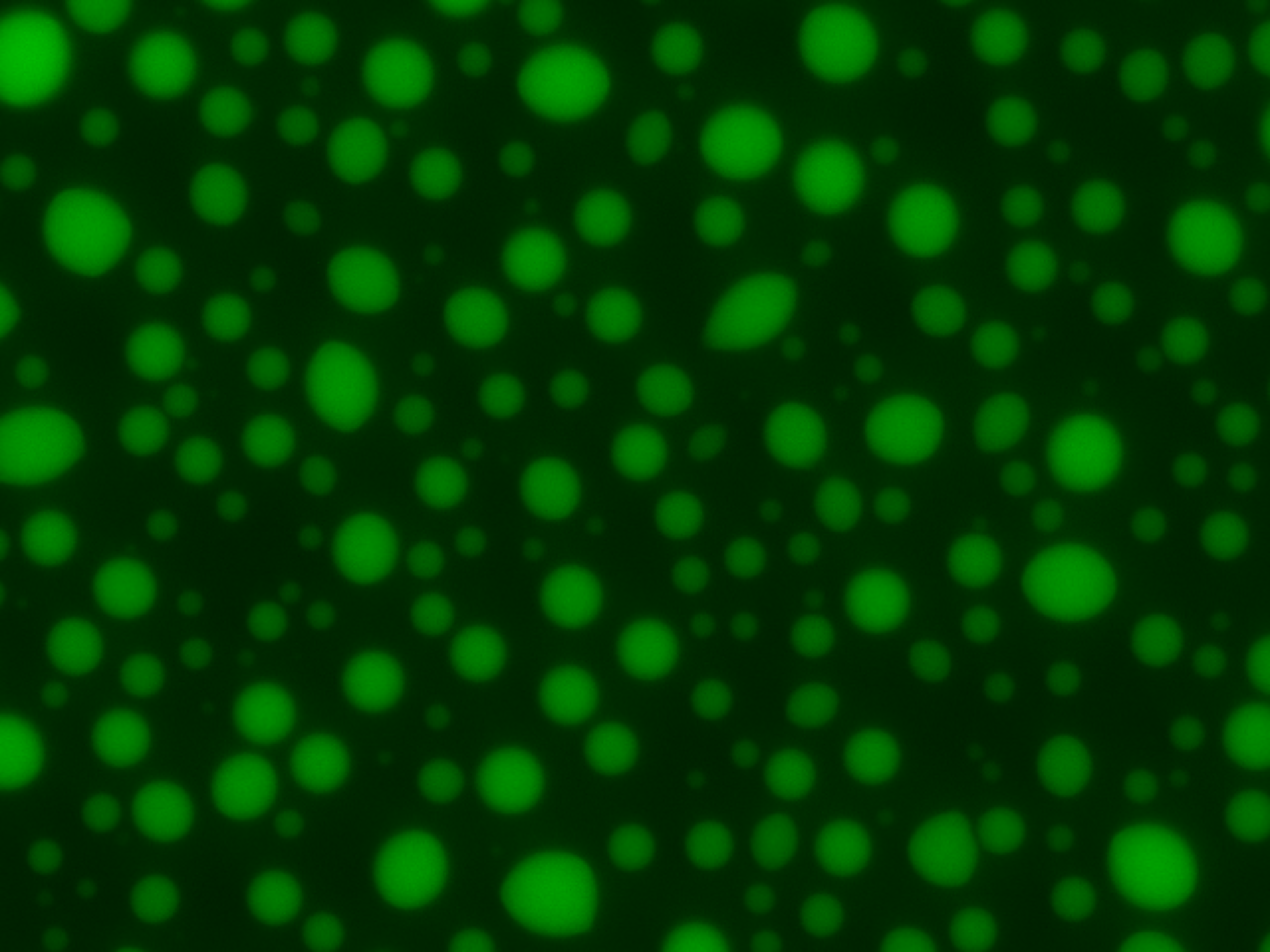

Supplement: Supplementary file 9 — EV Figures Source Data [file 44318_2025_591_MOESM9_ESM.zip › EMBOJ-2025-121908R1_SourceDataForEV/Expanded View Figure 1/EV1C/19_24 h_1,6-HD_UBQLN4(UBQLN4+╬▒-Syn).tif]

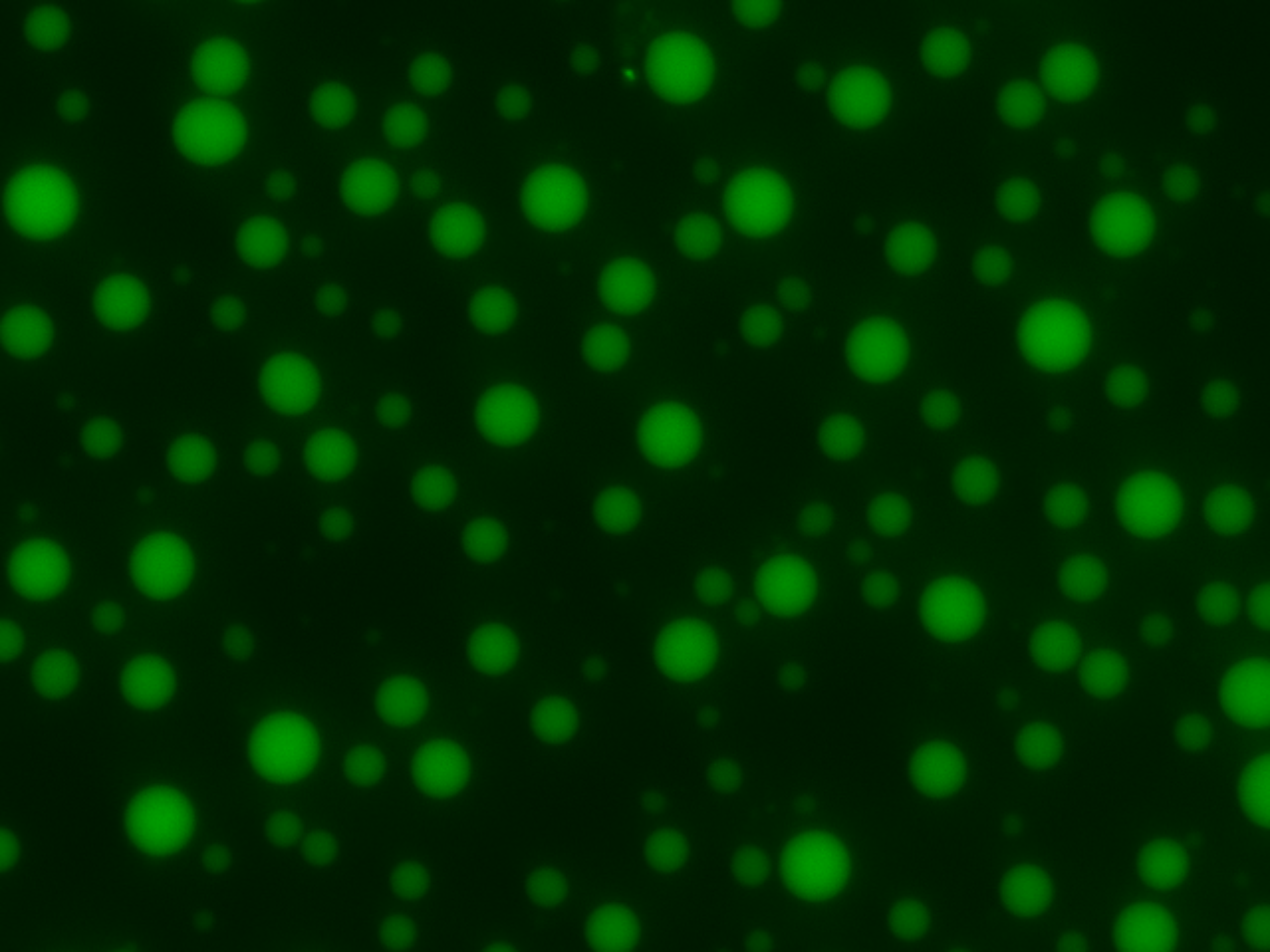

Supplement: Supplementary file 9 — EV Figures Source Data [file 44318_2025_591_MOESM9_ESM.zip › EMBOJ-2025-121908R1_SourceDataForEV/Expanded View Figure 1/EV1C/09_24 h_None_UBQLN1(UBQLN1+╬▒-Syn).tif]

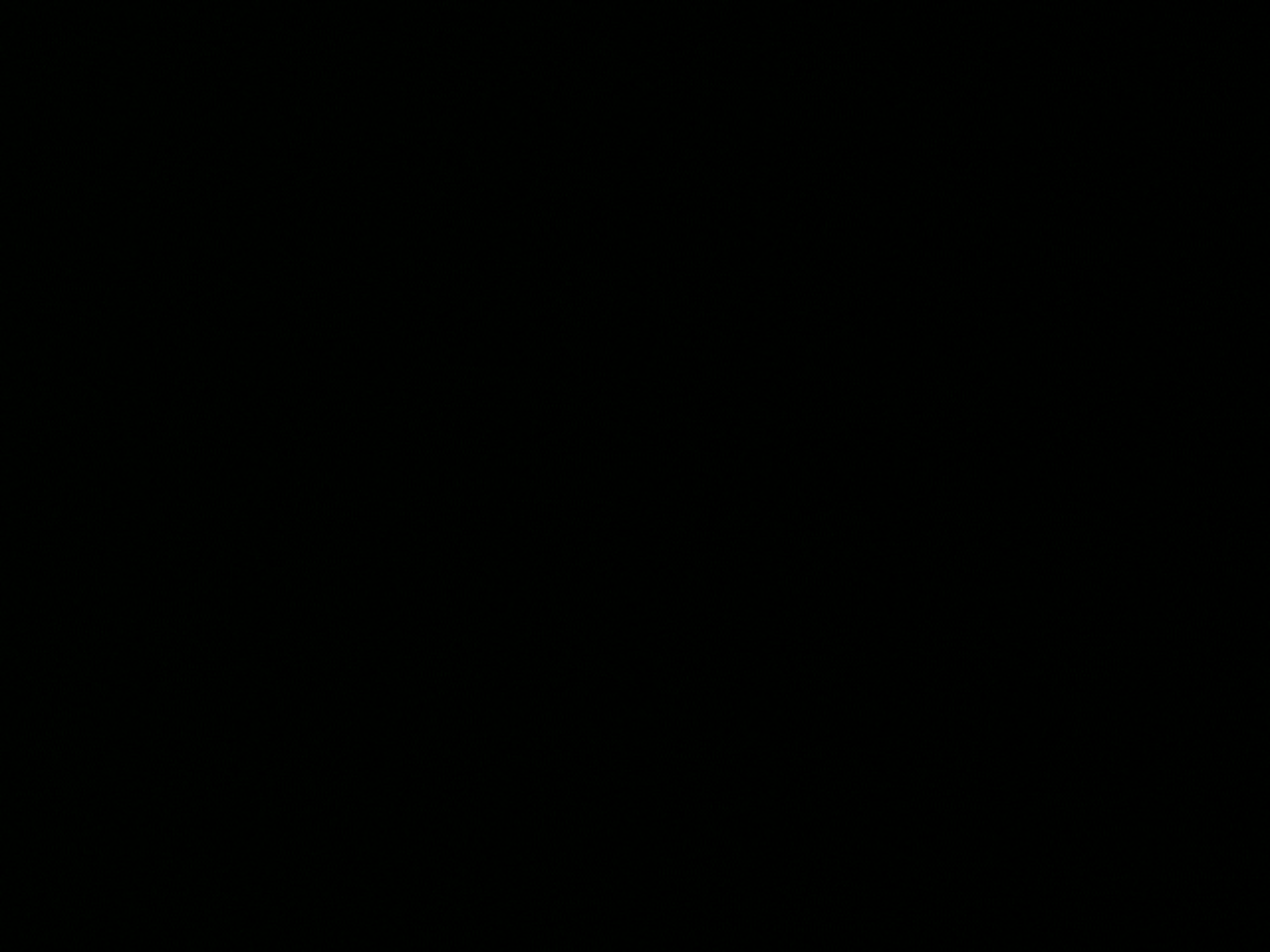

Supplement: Supplementary file 9 — EV Figures Source Data [file 44318_2025_591_MOESM9_ESM.zip › EMBOJ-2025-121908R1_SourceDataForEV/Expanded View Figure 1/EV1D/(a)_02_24h_UBQLN2_16HD_UBQLN2.tif]

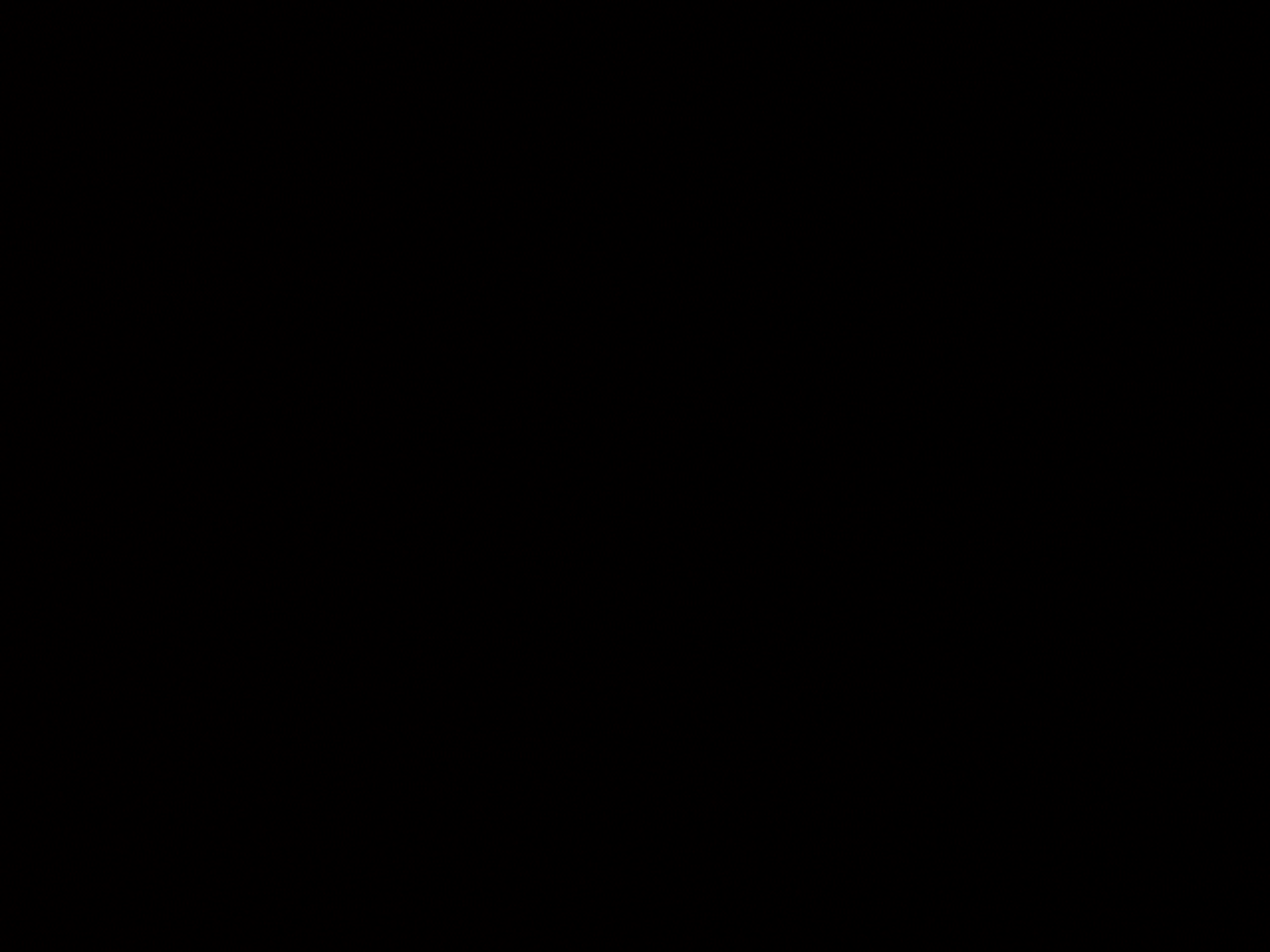

Supplement: Supplementary file 9 — EV Figures Source Data [file 44318_2025_591_MOESM9_ESM.zip › EMBOJ-2025-121908R1_SourceDataForEV/Expanded View Figure 1/EV1D/(a)_04_24h_UBQLN2+aSyn_16HD_aSyn.tif]

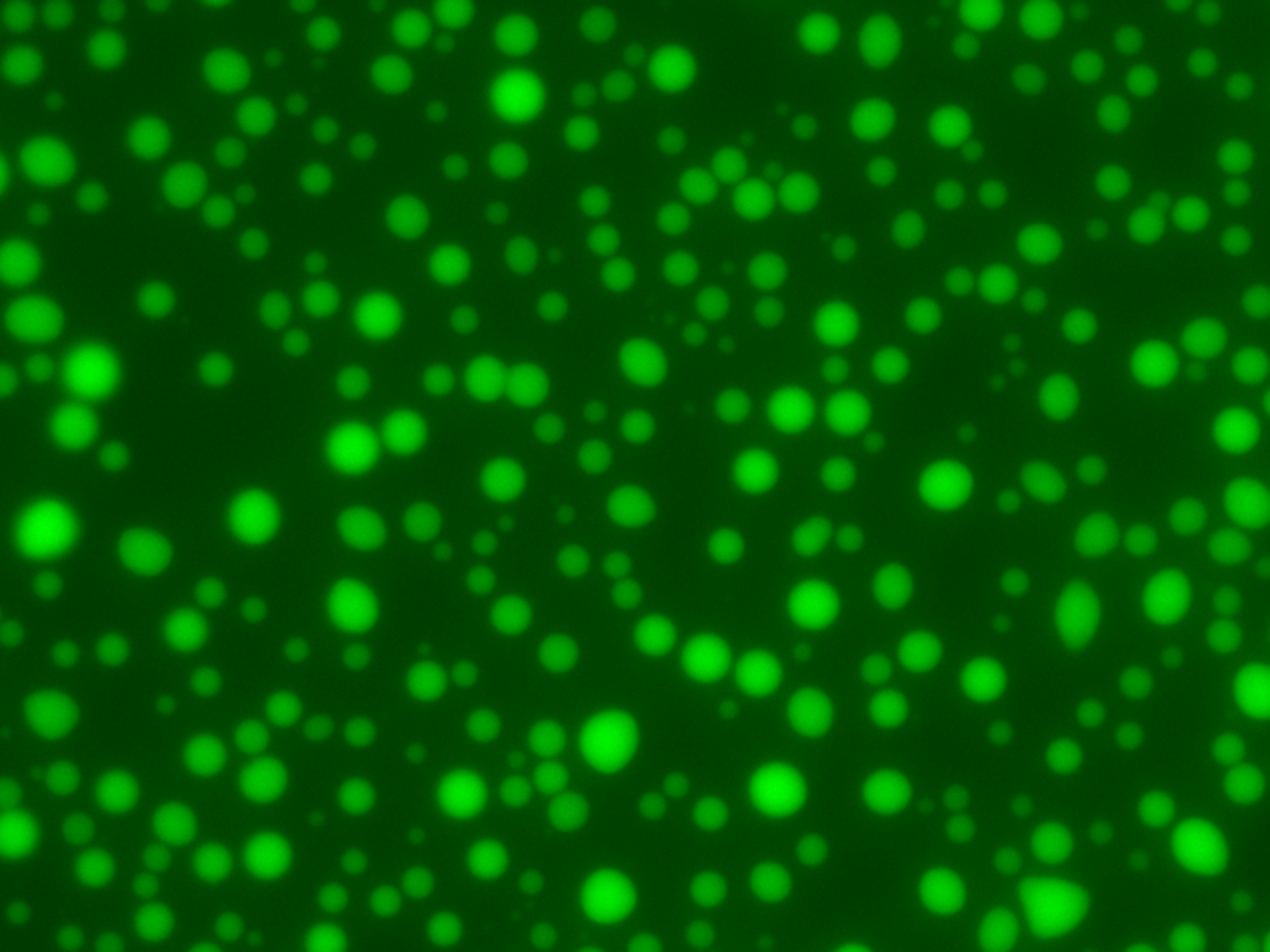

Supplement: Supplementary file 9 — EV Figures Source Data [file 44318_2025_591_MOESM9_ESM.zip › EMBOJ-2025-121908R1_SourceDataForEV/Expanded View Figure 1/EV1D/(a)_21_96h_UBQLN4_None_UBQLN4.tif]

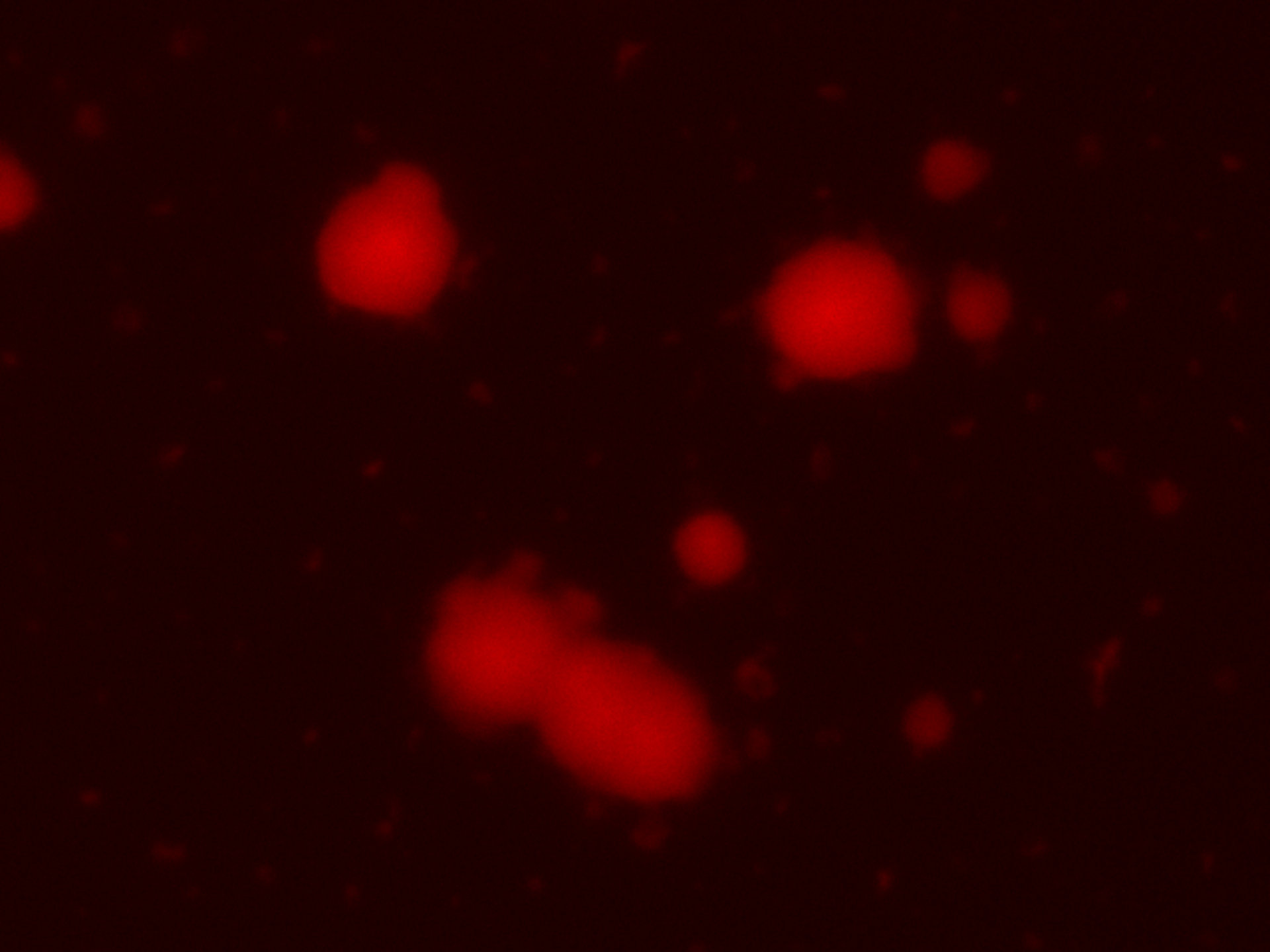

Supplement: Supplementary file 9 — EV Figures Source Data [file 44318_2025_591_MOESM9_ESM.zip › EMBOJ-2025-121908R1_SourceDataForEV/Expanded View Figure 1/EV1D/(a)_15_96h_UBQLN2+aSyn_None_aSyn.tif]

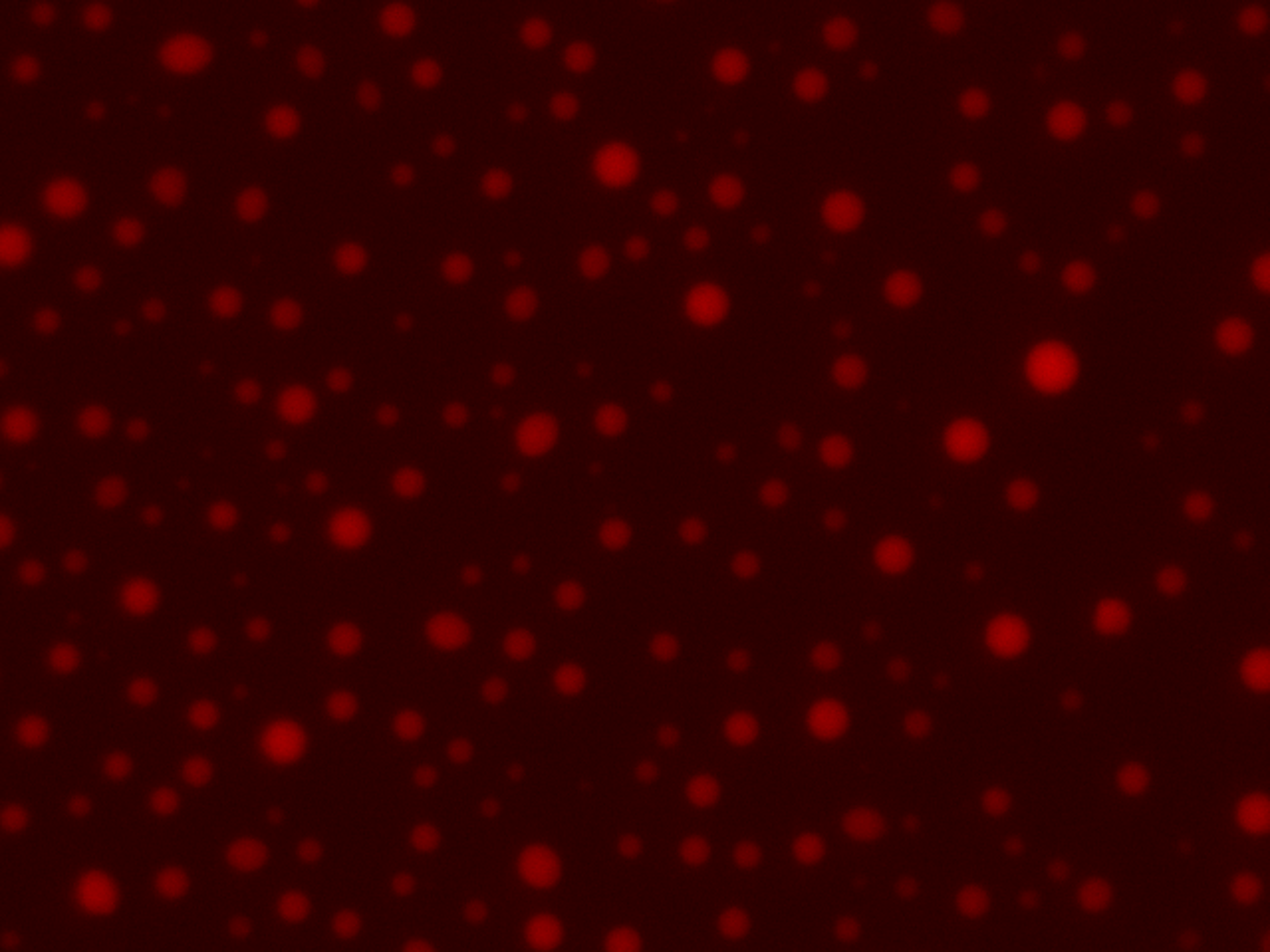

Supplement: Supplementary file 9 — EV Figures Source Data [file 44318_2025_591_MOESM9_ESM.zip › EMBOJ-2025-121908R1_SourceDataForEV/Expanded View Figure 1/EV1D/(a)_11_24h_UBQLN4+aSyn_None_aSyn.tif]

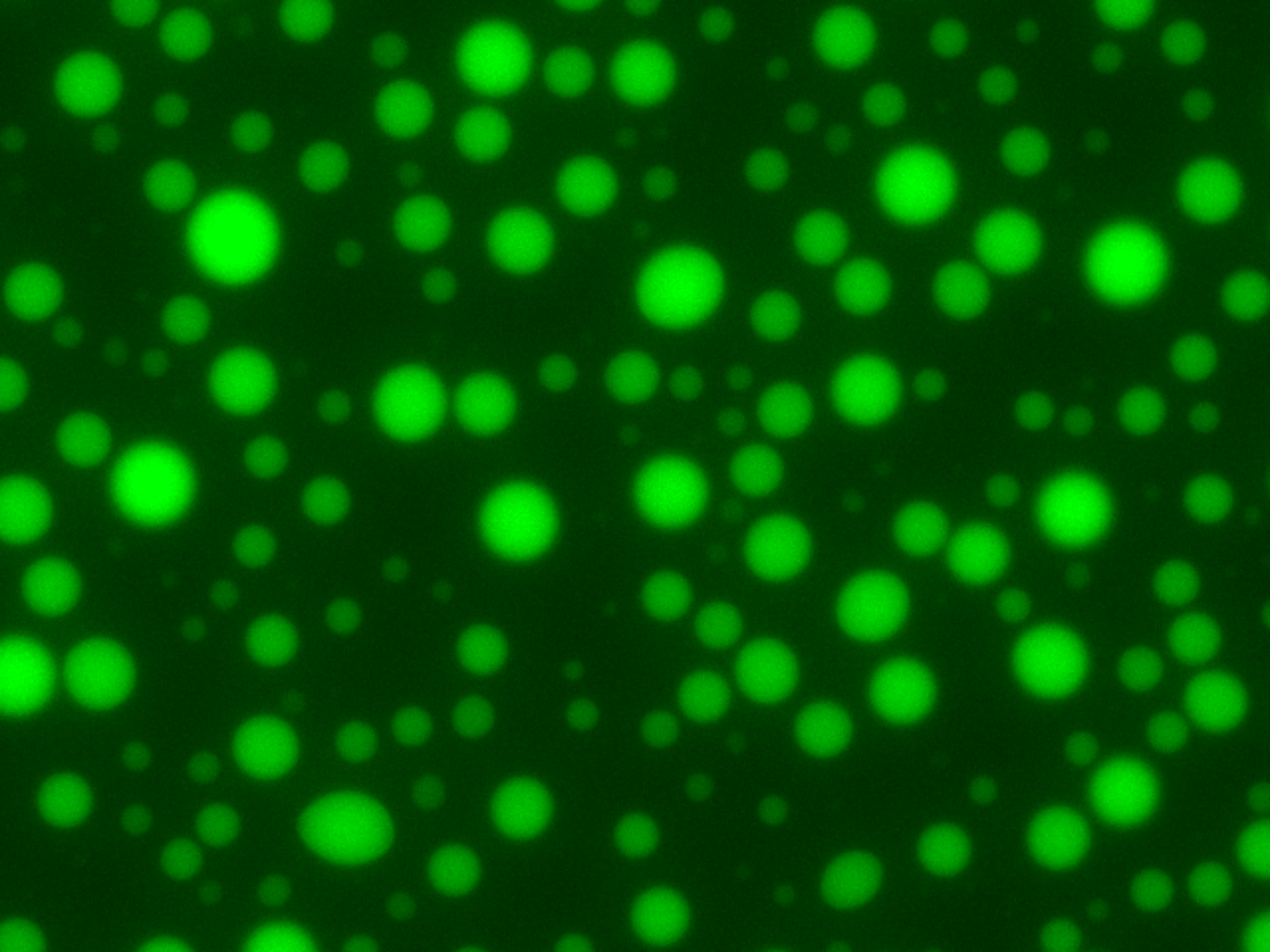

Supplement: Supplementary file 9 — EV Figures Source Data [file 44318_2025_591_MOESM9_ESM.zip › EMBOJ-2025-121908R1_SourceDataForEV/Expanded View Figure 1/EV1D/(a)_03_24h_UBQLN2+aSyn_None_UBQLN2.tif]

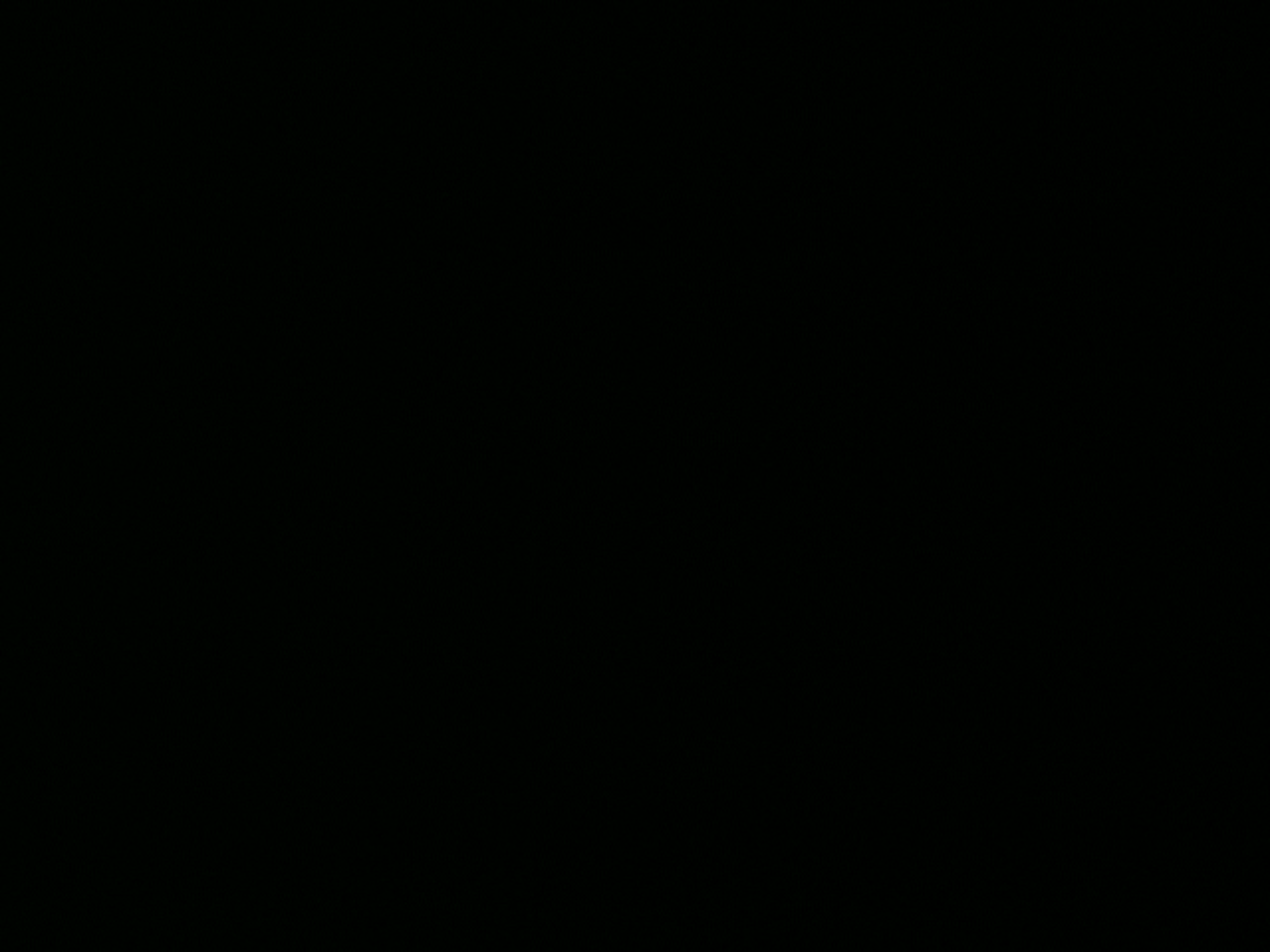

Supplement: Supplementary file 9 — EV Figures Source Data [file 44318_2025_591_MOESM9_ESM.zip › EMBOJ-2025-121908R1_SourceDataForEV/Expanded View Figure 1/EV1D/(a)_06_24h_UBQLN1_16HD_UBQLN1.tif]

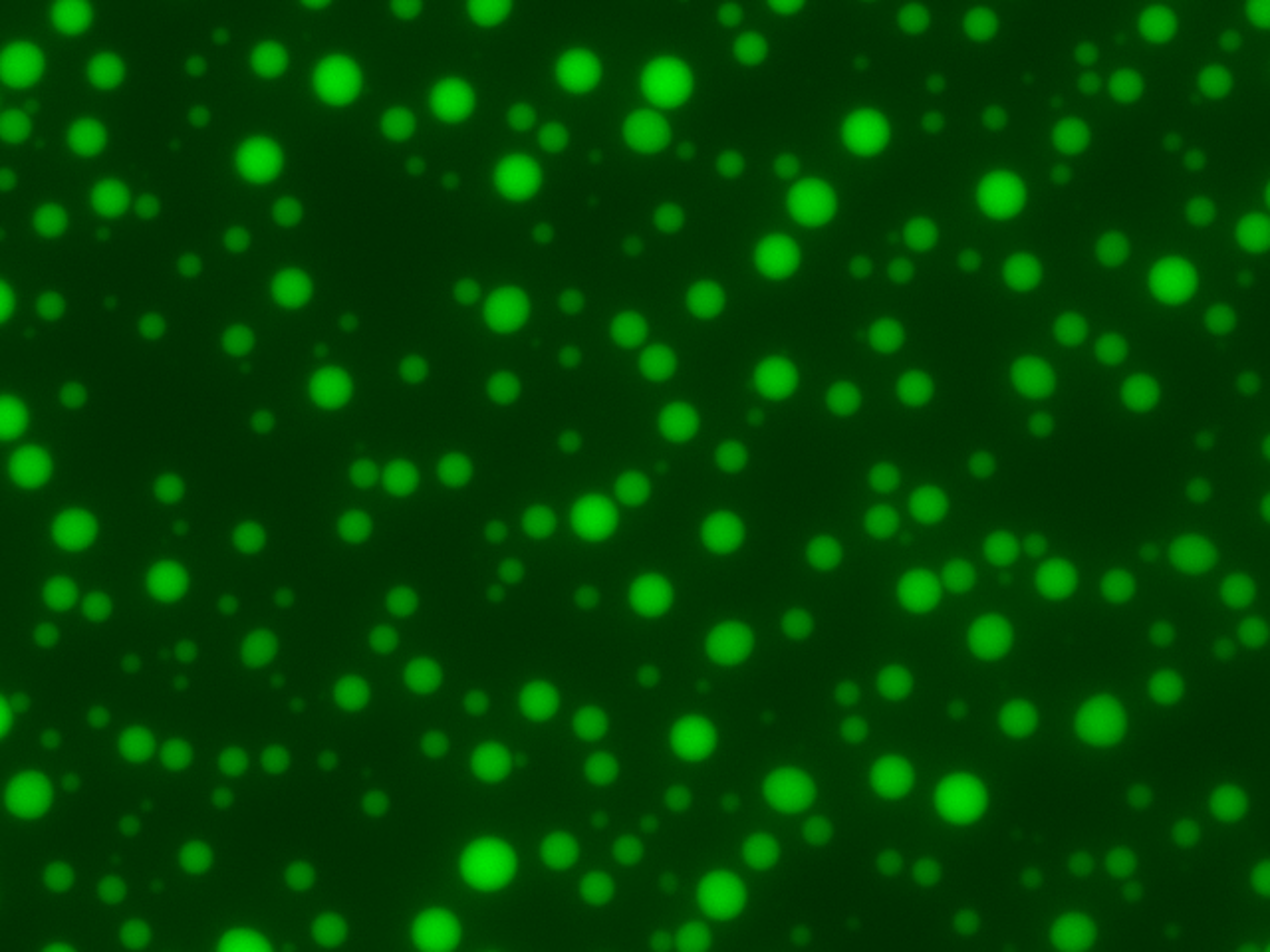

Supplement: Supplementary file 9 — EV Figures Source Data [file 44318_2025_591_MOESM9_ESM.zip › EMBOJ-2025-121908R1_SourceDataForEV/Expanded View Figure 1/EV1D/(a)_23_96h_UBQLN4+aSyn_None_UBQLN4.tif]

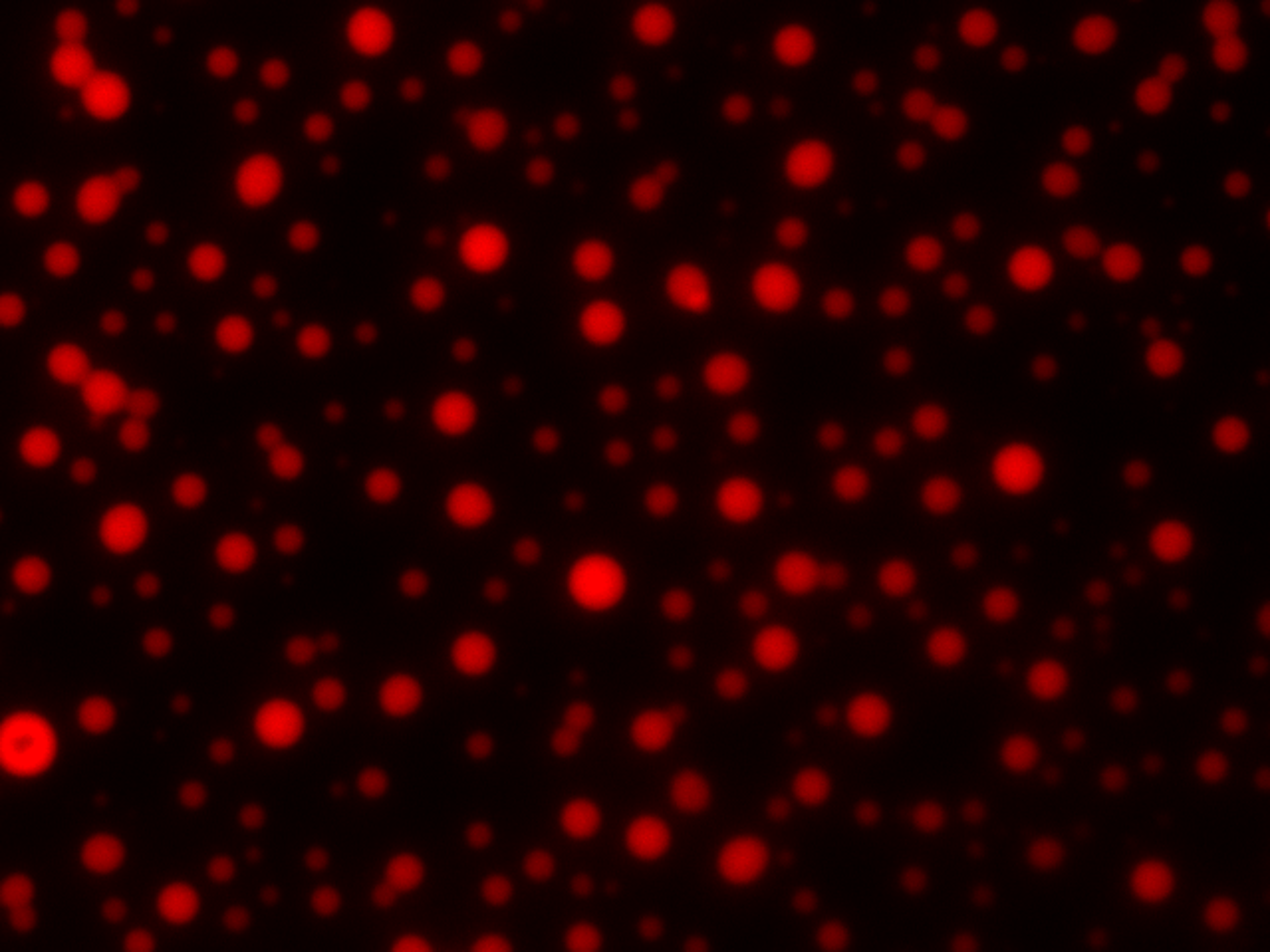

Supplement: Supplementary file 9 — EV Figures Source Data [file 44318_2025_591_MOESM9_ESM.zip › EMBOJ-2025-121908R1_SourceDataForEV/Expanded View Figure 1/EV1D/(a)_24_96h_UBQLN4+aSyn_16HD_aSyn.tif]

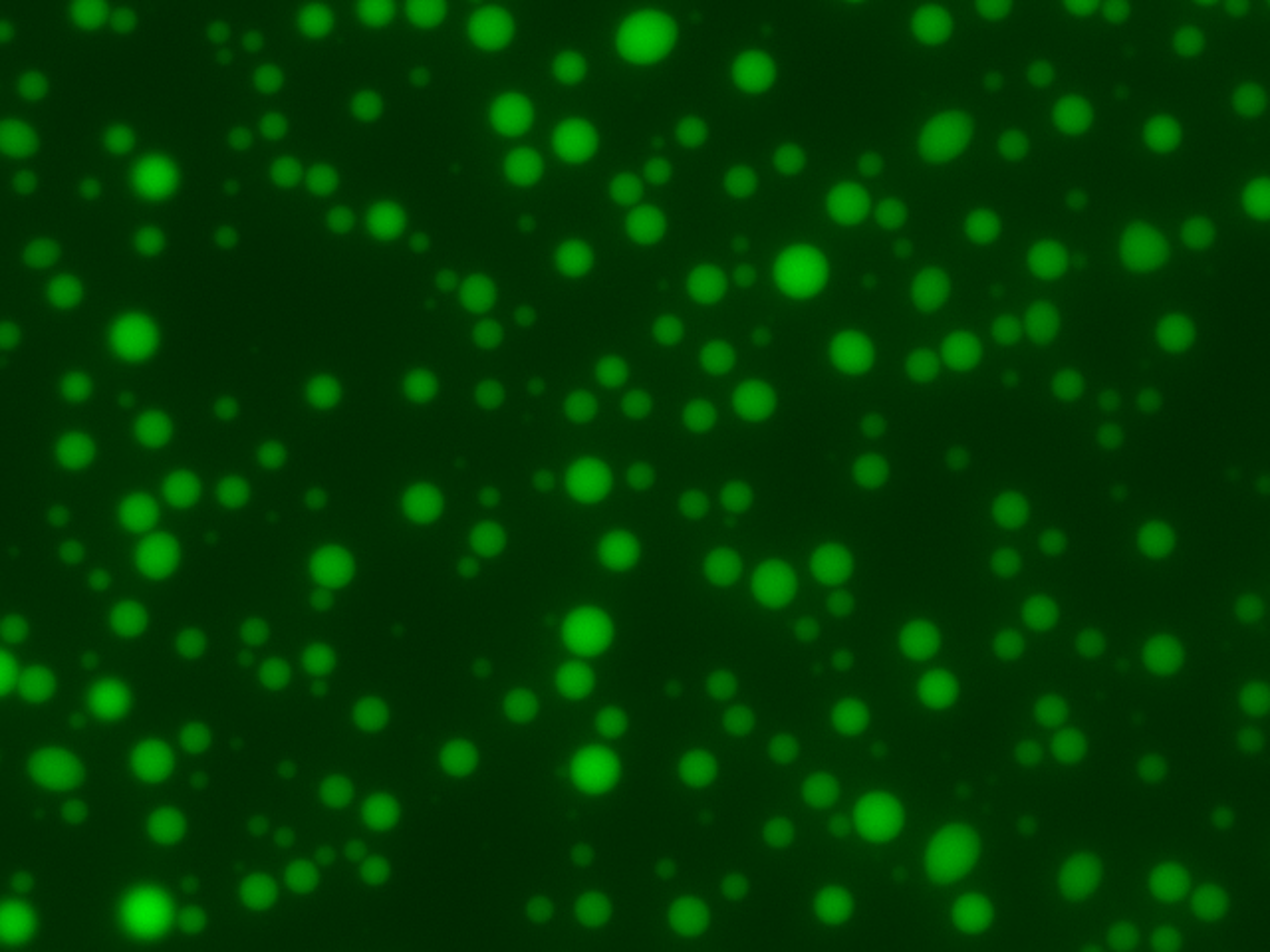

Supplement: Supplementary file 9 — EV Figures Source Data [file 44318_2025_591_MOESM9_ESM.zip › EMBOJ-2025-121908R1_SourceDataForEV/Expanded View Figure 1/EV1D/(a)_09_24h_UBQLN4_None_UBQLN4.tif]

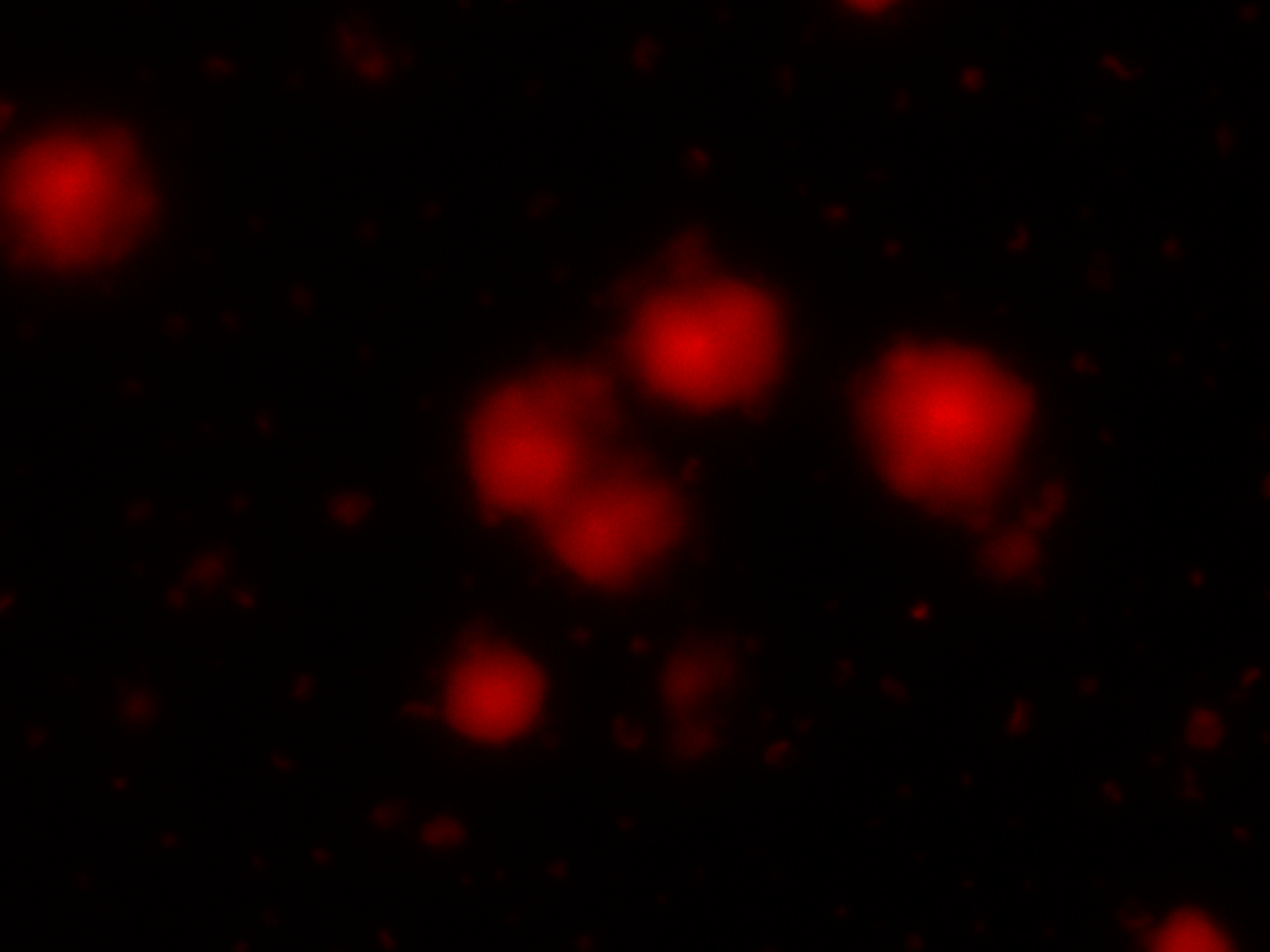

Supplement: Supplementary file 9 — EV Figures Source Data [file 44318_2025_591_MOESM9_ESM.zip › EMBOJ-2025-121908R1_SourceDataForEV/Expanded View Figure 1/EV1D/(a)_16_96h_UBQLN2+aSyn_16HD_aSyn.tif]

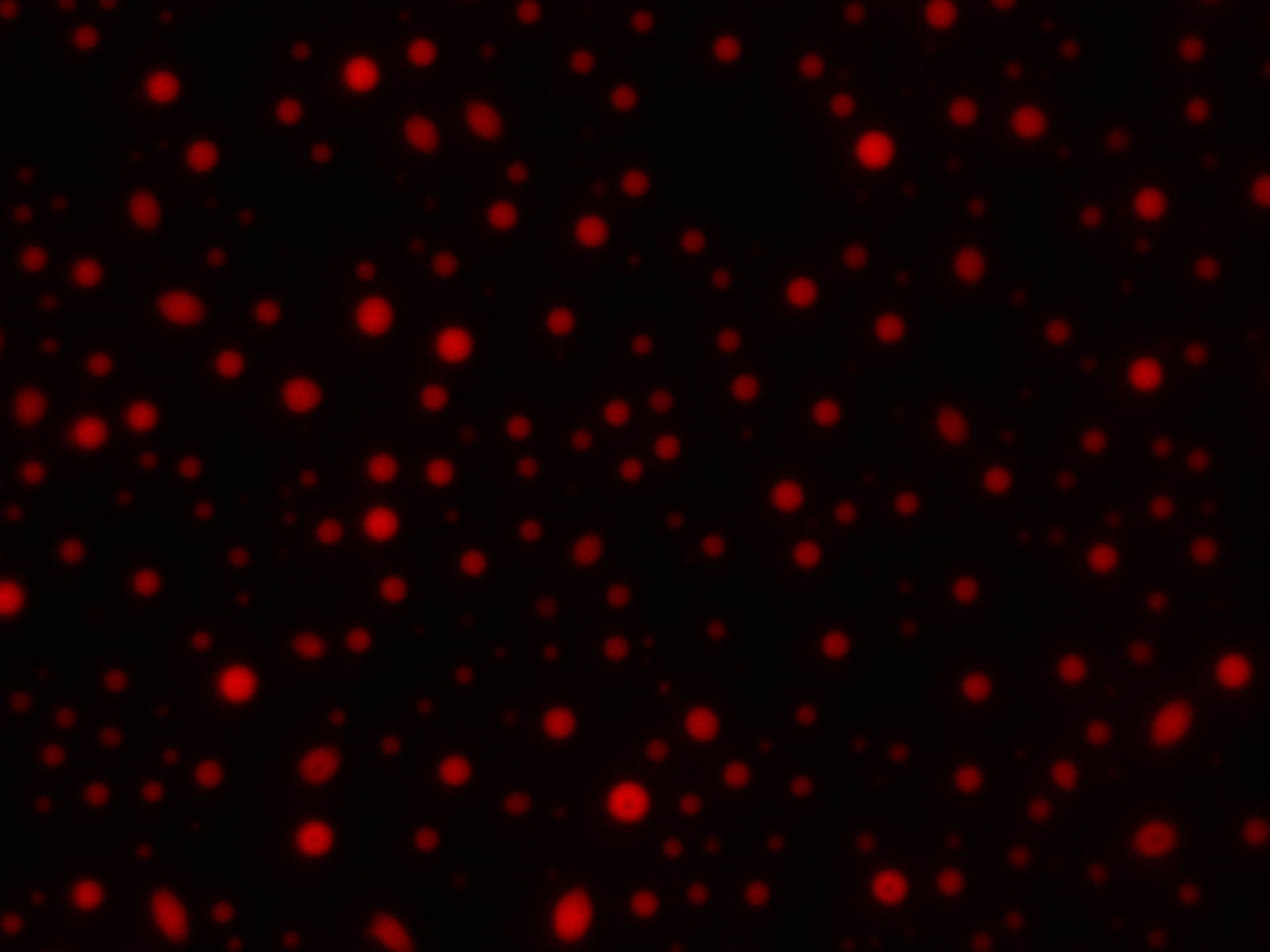

Supplement: Supplementary file 9 — EV Figures Source Data [file 44318_2025_591_MOESM9_ESM.zip › EMBOJ-2025-121908R1_SourceDataForEV/Expanded View Figure 1/EV1D/(a)_12_24h_UBQLN4+aSyn_16HD_aSyn.tif]

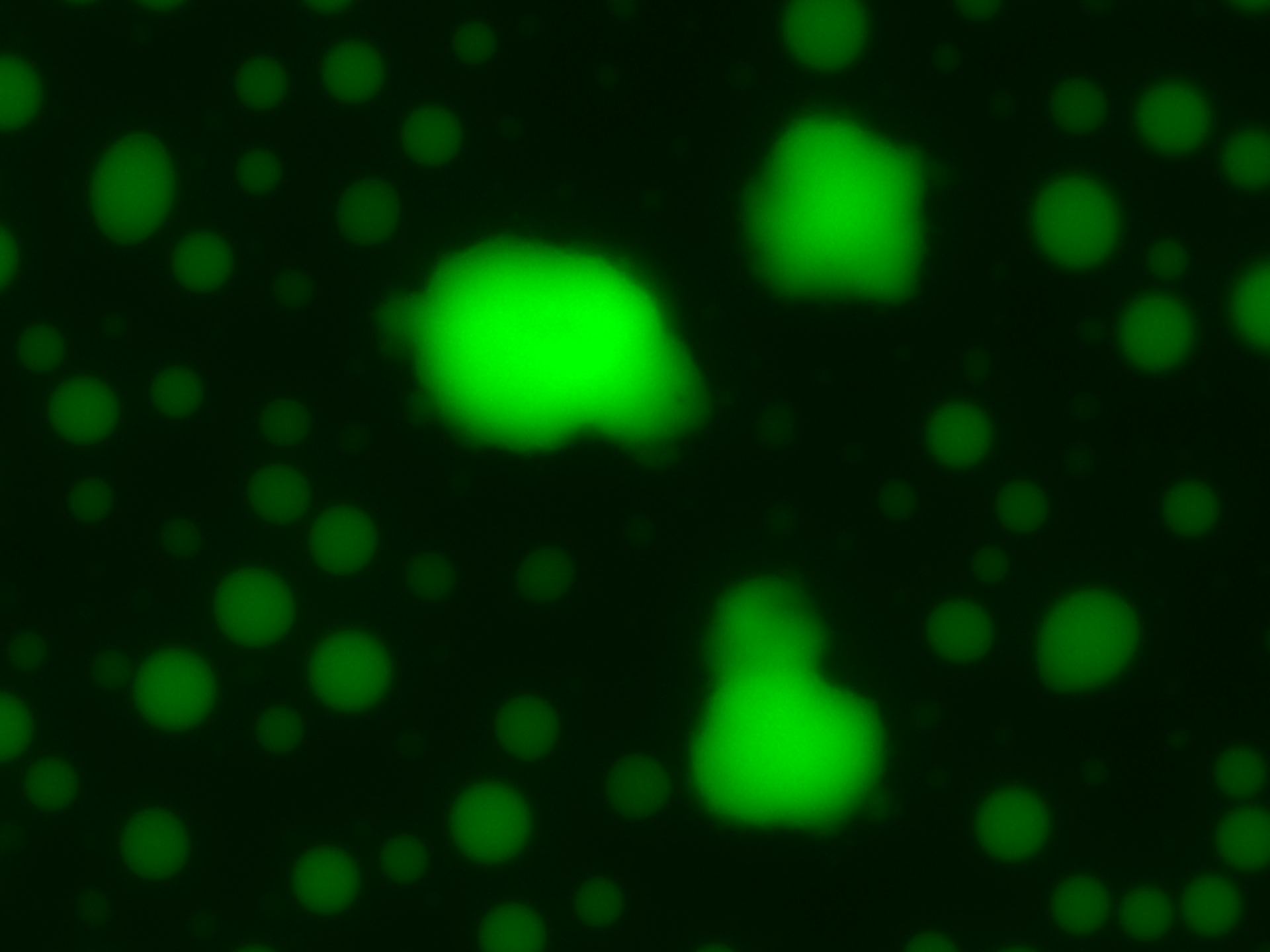

Supplement: Supplementary file 9 — EV Figures Source Data [file 44318_2025_591_MOESM9_ESM.zip › EMBOJ-2025-121908R1_SourceDataForEV/Expanded View Figure 1/EV1D/(a)_13_96h_UBQLN2_None_UBQLN2.tif]

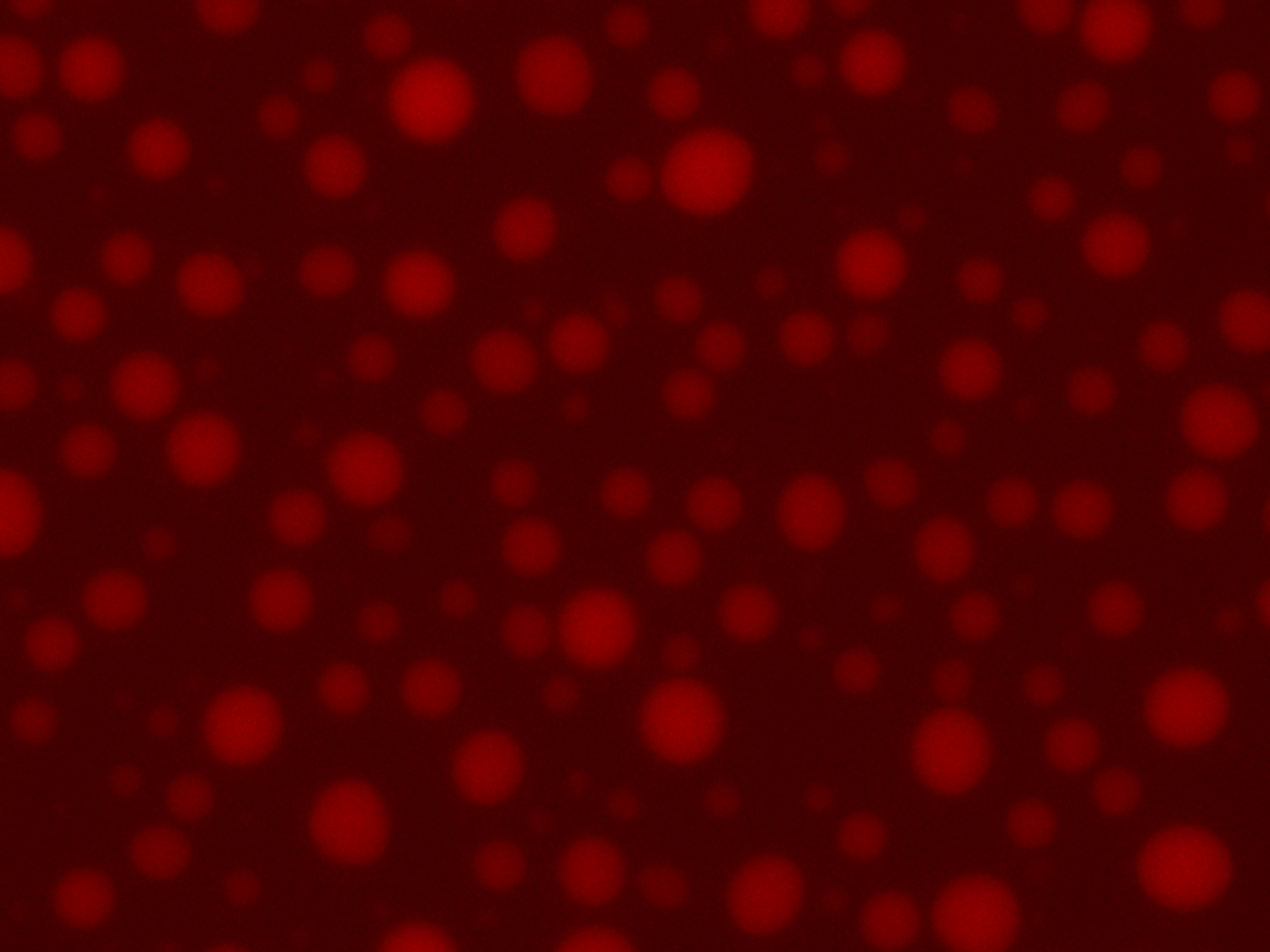

Supplement: Supplementary file 9 — EV Figures Source Data [file 44318_2025_591_MOESM9_ESM.zip › EMBOJ-2025-121908R1_SourceDataForEV/Expanded View Figure 1/EV1D/(a)_07_24h_UBQLN1+aSyn_None_aSyn.tif]

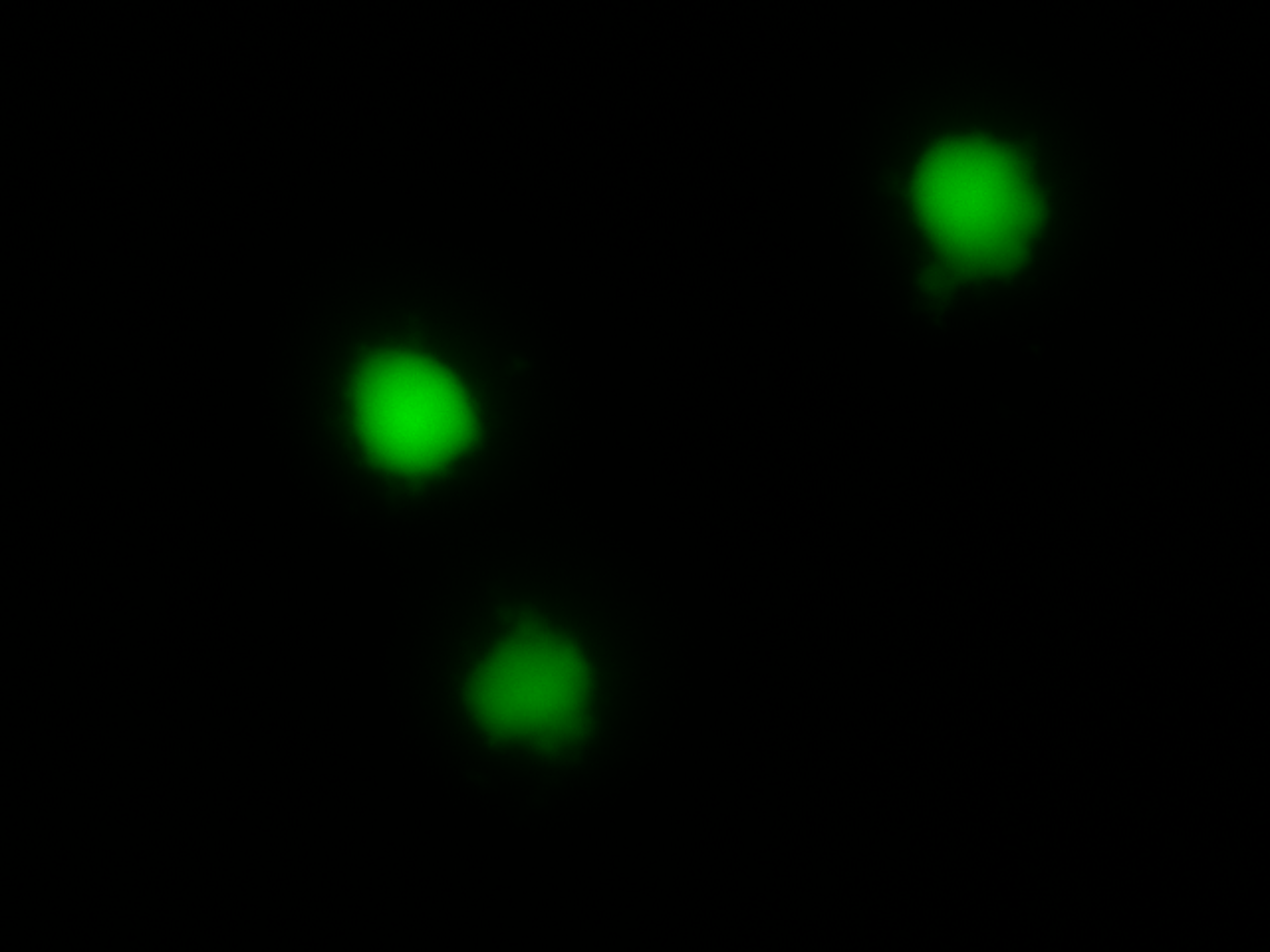

Supplement: Supplementary file 9 — EV Figures Source Data [file 44318_2025_591_MOESM9_ESM.zip › EMBOJ-2025-121908R1_SourceDataForEV/Expanded View Figure 1/EV1D/(a)_14_96h_UBQLN2_16HD_UBQLN2.tif]

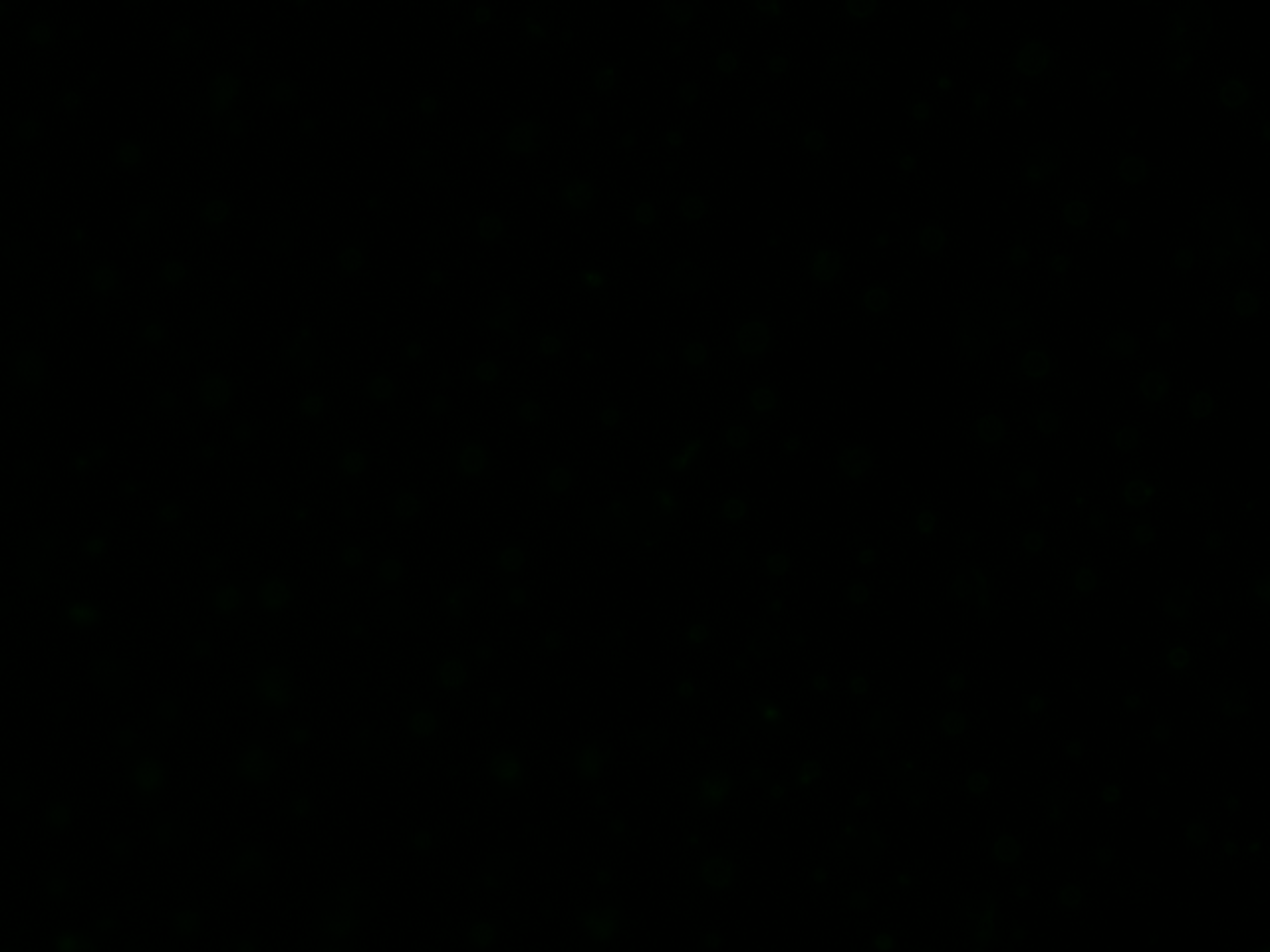

Supplement: Supplementary file 9 — EV Figures Source Data [file 44318_2025_591_MOESM9_ESM.zip › EMBOJ-2025-121908R1_SourceDataForEV/Expanded View Figure 1/EV1D/(a)_20_96h_UBQLN1+aSyn_16HD_UBQLN1.tif]

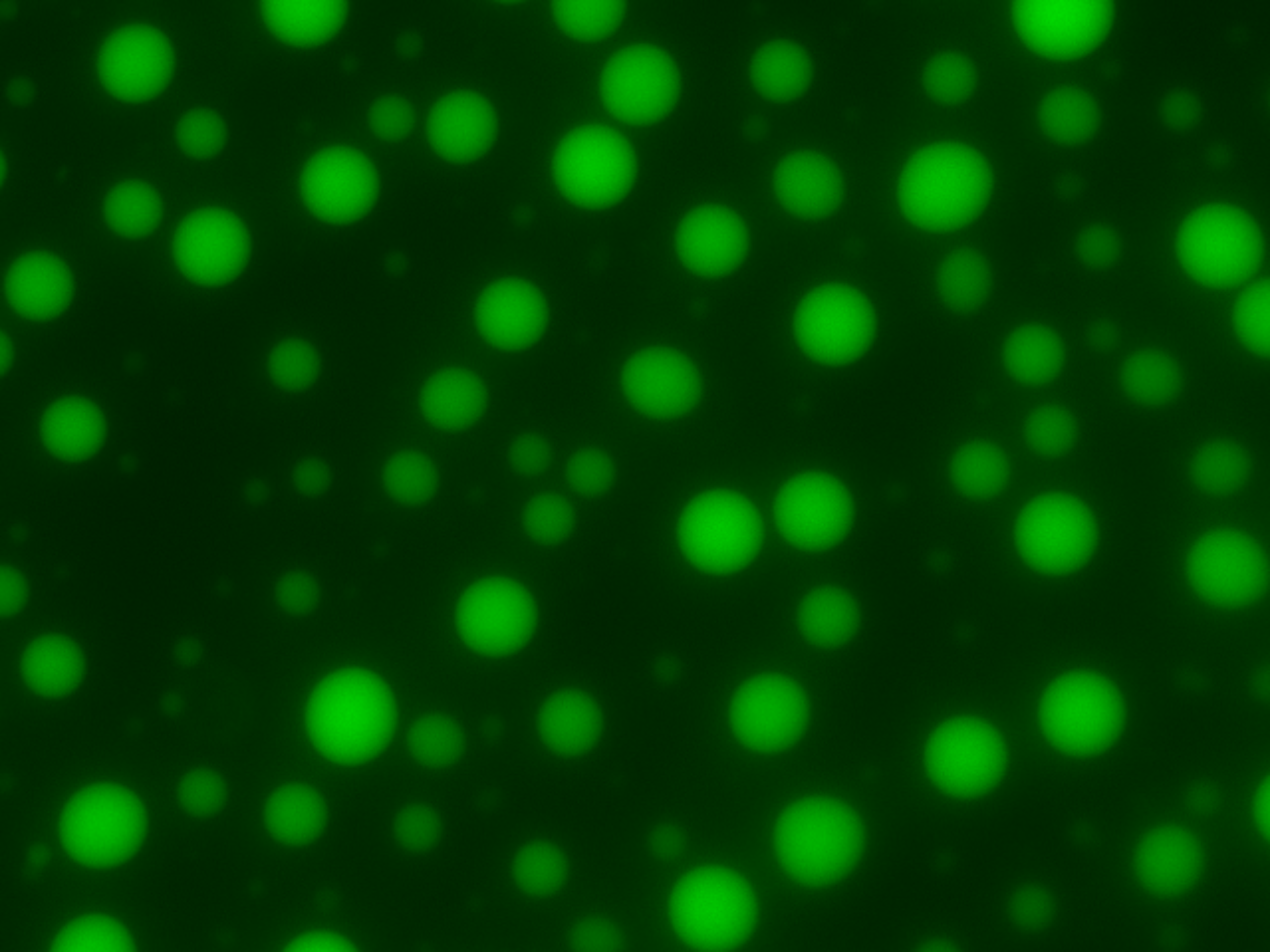

Supplement: Supplementary file 9 — EV Figures Source Data [file 44318_2025_591_MOESM9_ESM.zip › EMBOJ-2025-121908R1_SourceDataForEV/Expanded View Figure 1/EV1D/(a)_17_96h_UBQLN1_None_UBQLN1.tif]

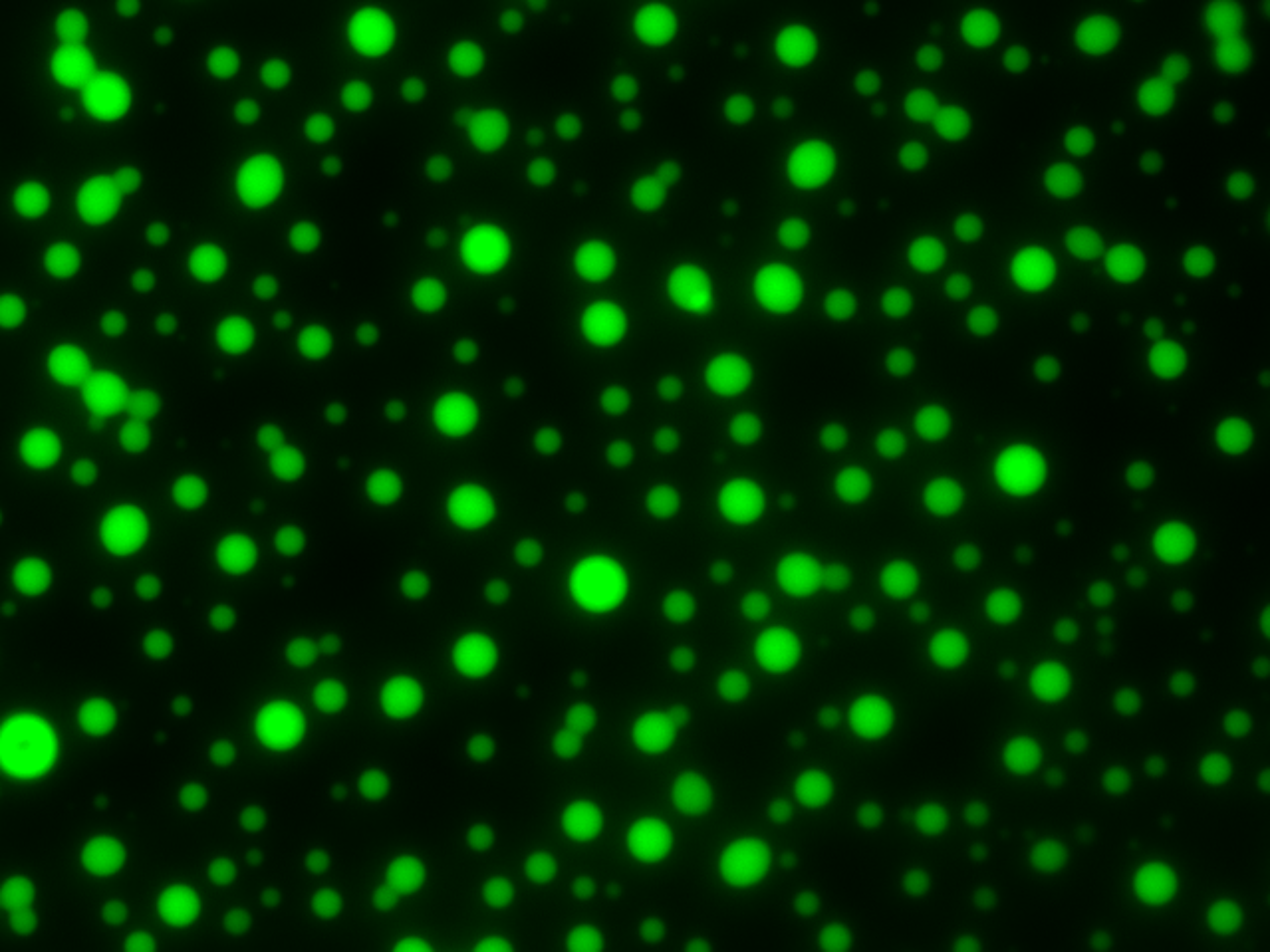

Supplement: Supplementary file 9 — EV Figures Source Data [file 44318_2025_591_MOESM9_ESM.zip › EMBOJ-2025-121908R1_SourceDataForEV/Expanded View Figure 1/EV1D/(a)_24_96h_UBQLN4+aSyn_16HD_UBQLN4.tif]

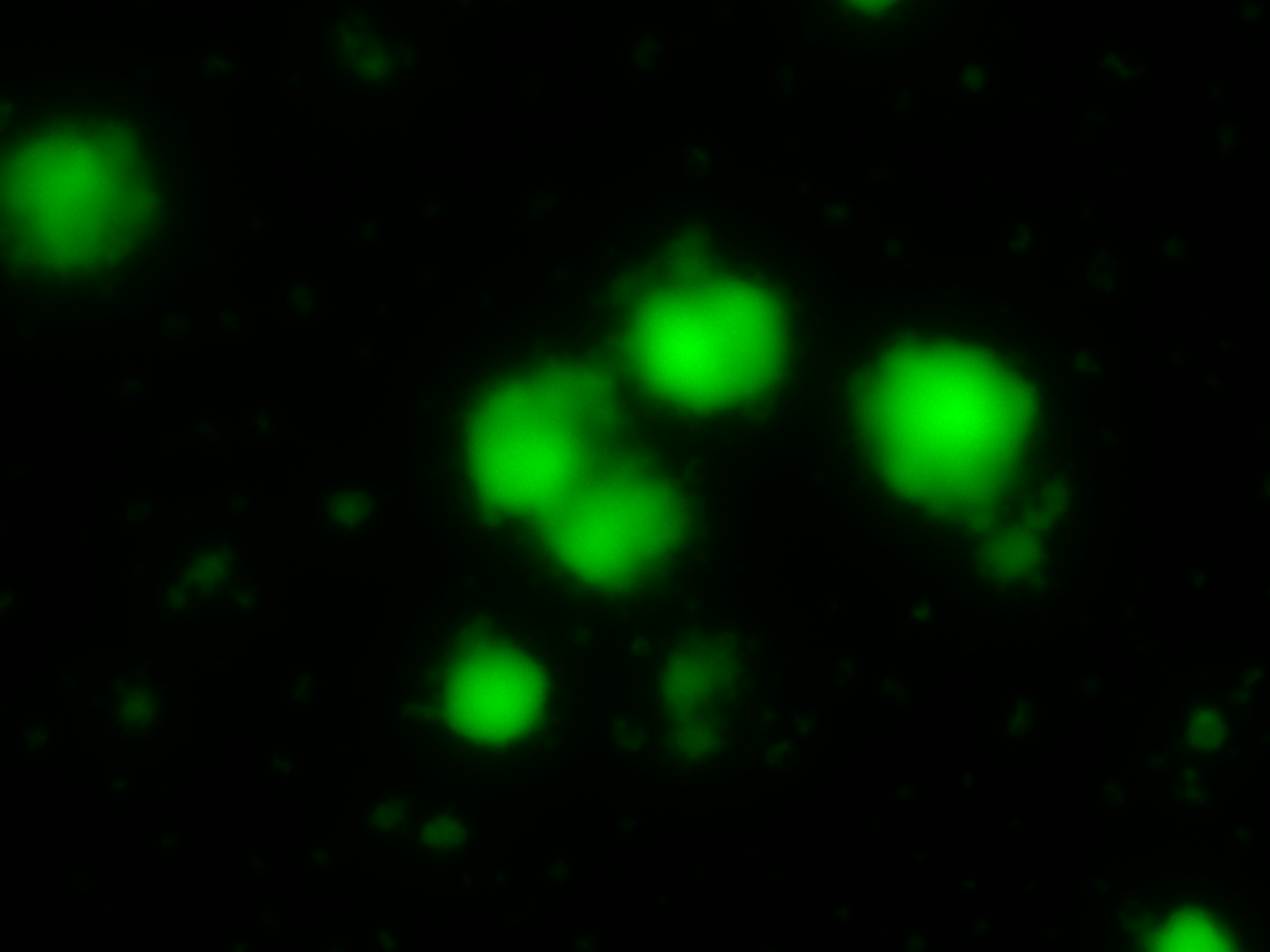

Supplement: Supplementary file 9 — EV Figures Source Data [file 44318_2025_591_MOESM9_ESM.zip › EMBOJ-2025-121908R1_SourceDataForEV/Expanded View Figure 1/EV1D/(a)_16_96h_UBQLN2+aSyn_16HD_UBQLN2.tif]

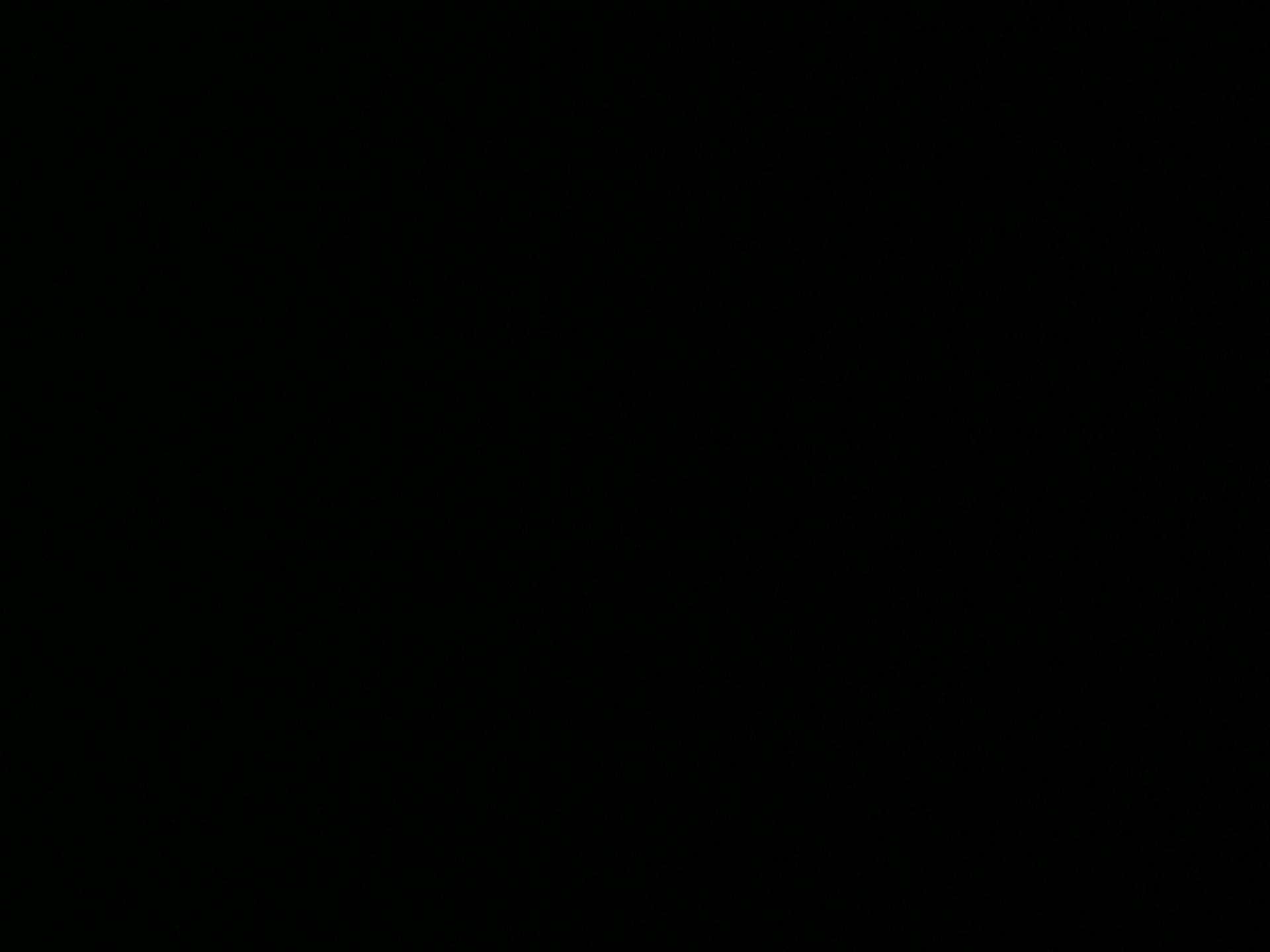

Supplement: Supplementary file 9 — EV Figures Source Data [file 44318_2025_591_MOESM9_ESM.zip › EMBOJ-2025-121908R1_SourceDataForEV/Expanded View Figure 1/EV1D/(a)_04_24h_UBQLN2+aSyn_16HD_UBQLN2.tif]

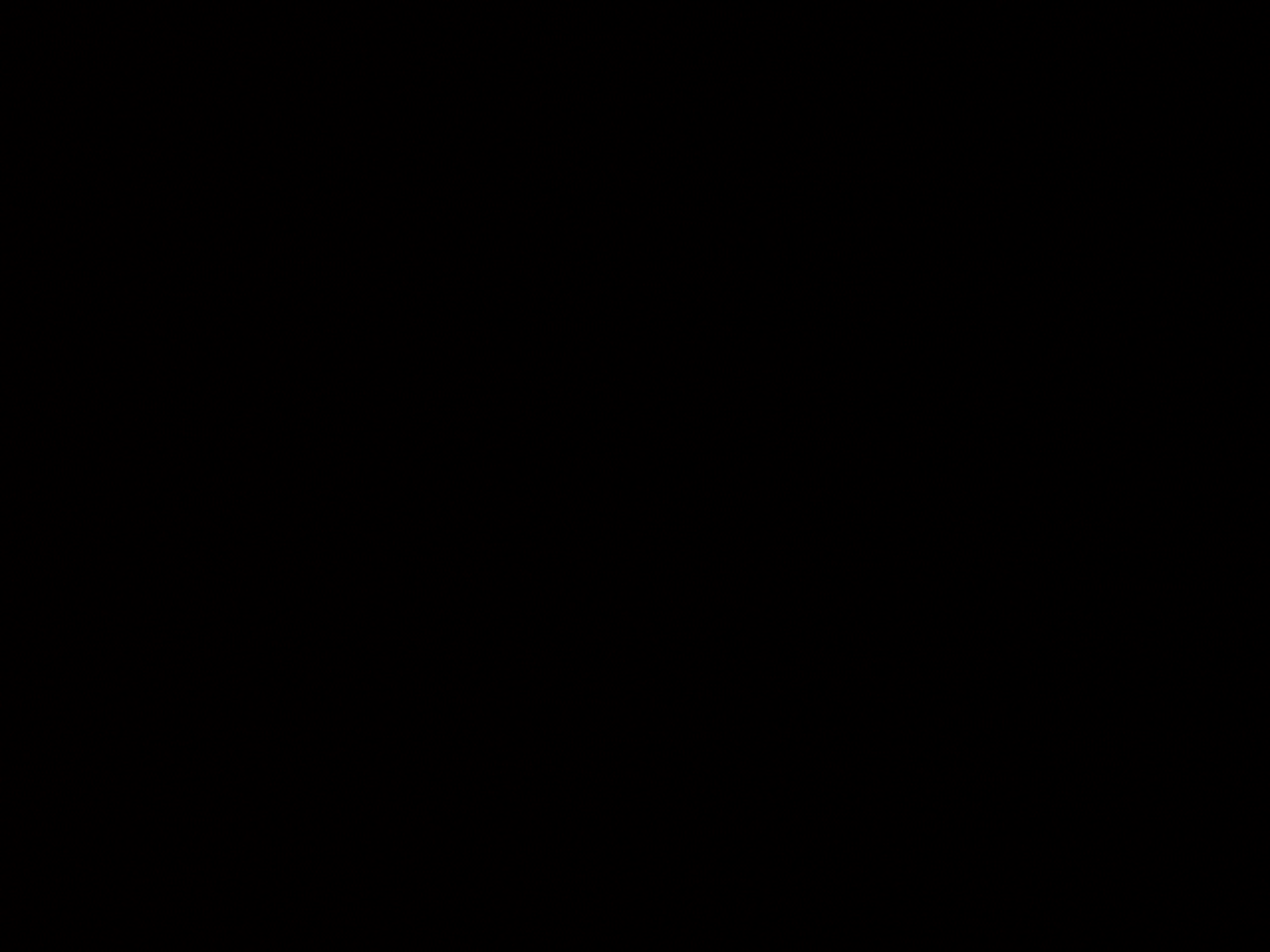

Supplement: Supplementary file 9 — EV Figures Source Data [file 44318_2025_591_MOESM9_ESM.zip › EMBOJ-2025-121908R1_SourceDataForEV/Expanded View Figure 1/EV1D/(a)_08_24h_UBQLN1+aSyn_16HD_UBQLN1.tif]

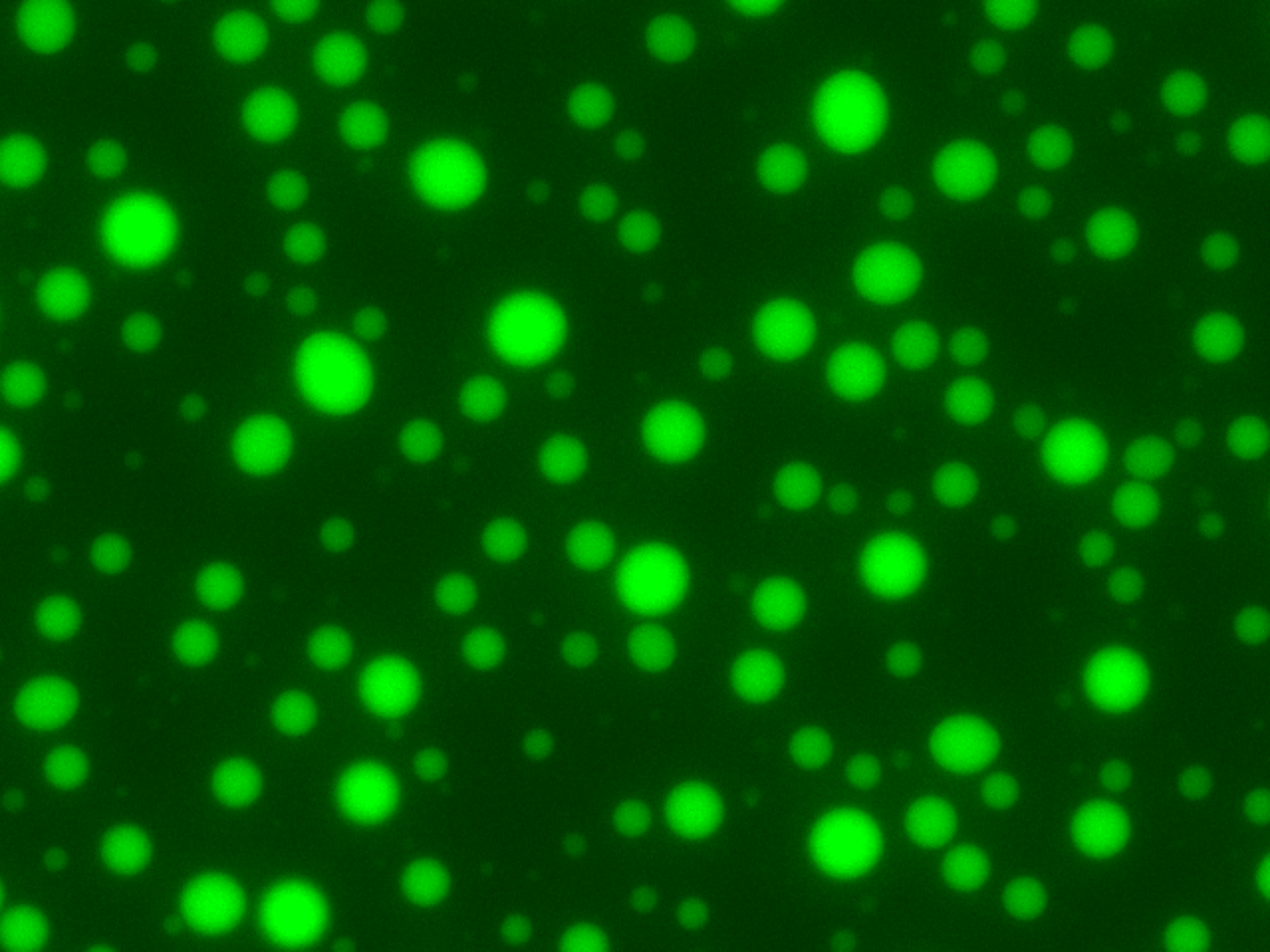

Supplement: Supplementary file 9 — EV Figures Source Data [file 44318_2025_591_MOESM9_ESM.zip › EMBOJ-2025-121908R1_SourceDataForEV/Expanded View Figure 1/EV1D/(a)_05_24h_UBQLN1_None_UBQLN1.tif]

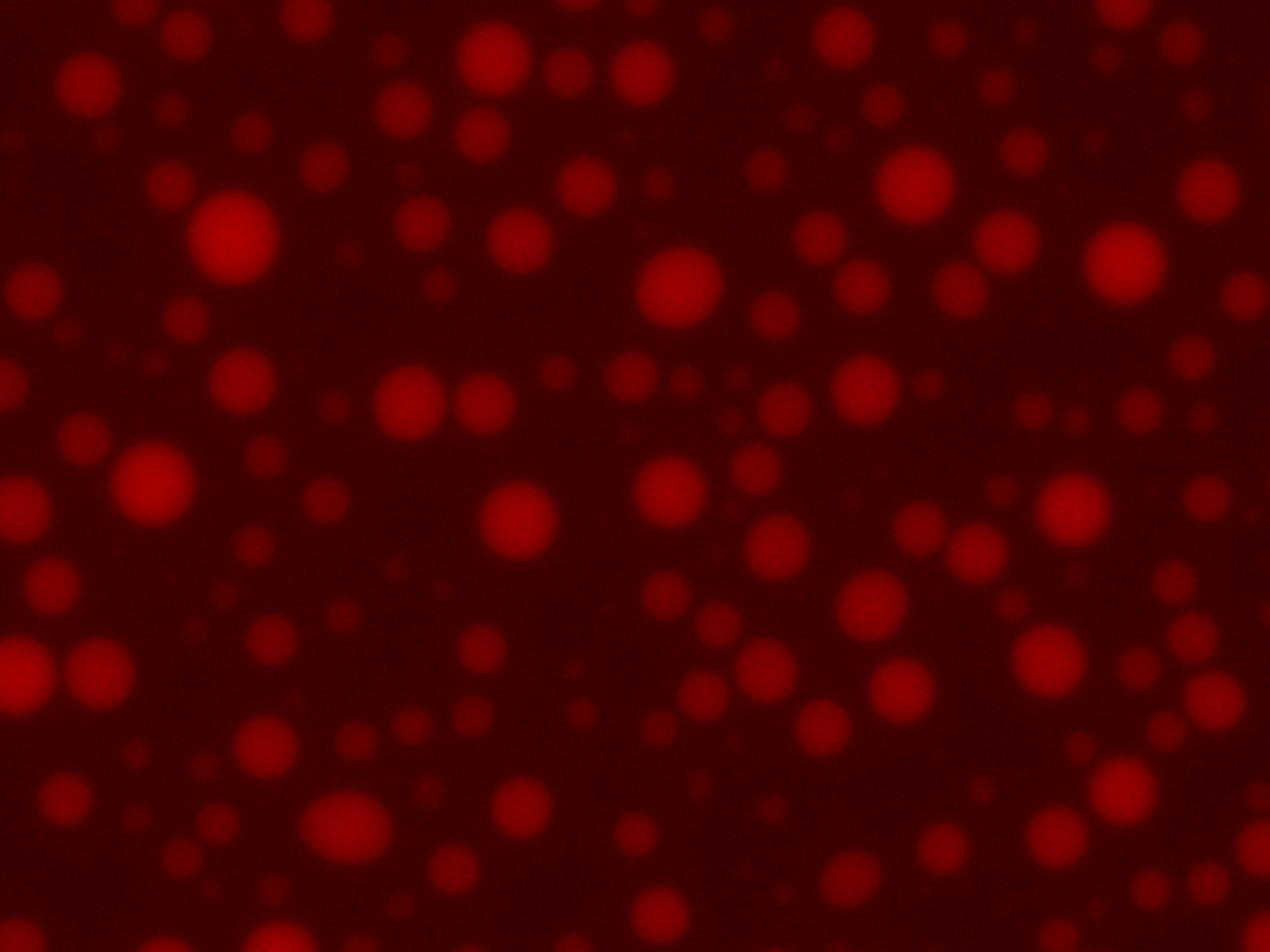

Supplement: Supplementary file 9 — EV Figures Source Data [file 44318_2025_591_MOESM9_ESM.zip › EMBOJ-2025-121908R1_SourceDataForEV/Expanded View Figure 1/EV1D/(a)_03_24h_UBQLN2+aSyn_None_aSyn.tif]
