# Supplementary figures and images for: Ubiquilin-2 liquid droplets catalyze α-synuclein fibril formation (part 3 of 3)
Source: EMBO J. 2025 Oct 14;44(22):6527–55. doi: 10.1038/s44318-025-00591-1 (PMC12623503; doi:10.1038/s44318-025-00591-1)

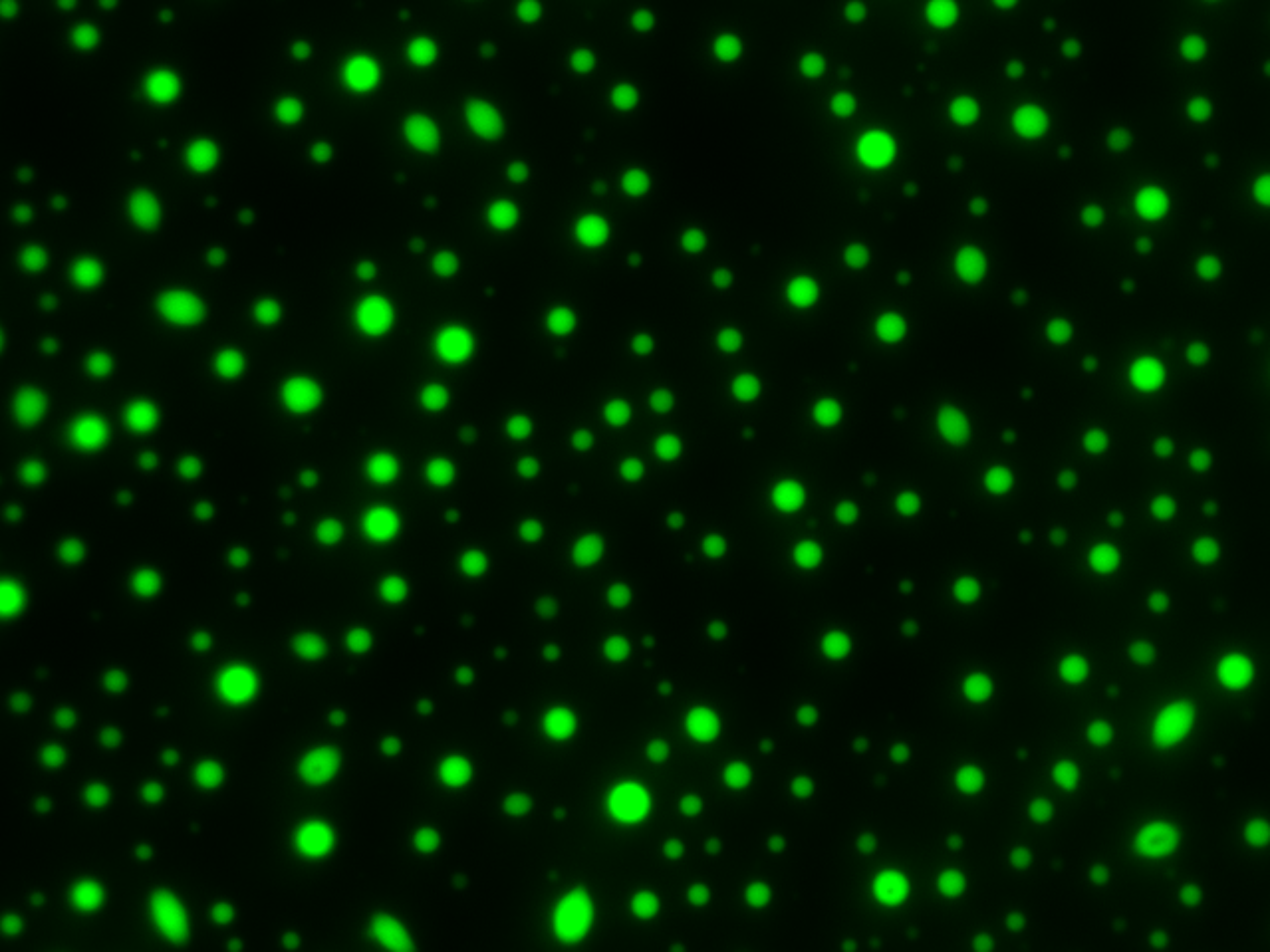

Supplement: Supplementary file 9 — EV Figures Source Data [file 44318_2025_591_MOESM9_ESM.zip › EMBOJ-2025-121908R1_SourceDataForEV/Expanded View Figure 1/EV1D/(a)_12_24h_UBQLN4+aSyn_16HD_UBQLN4.tif]

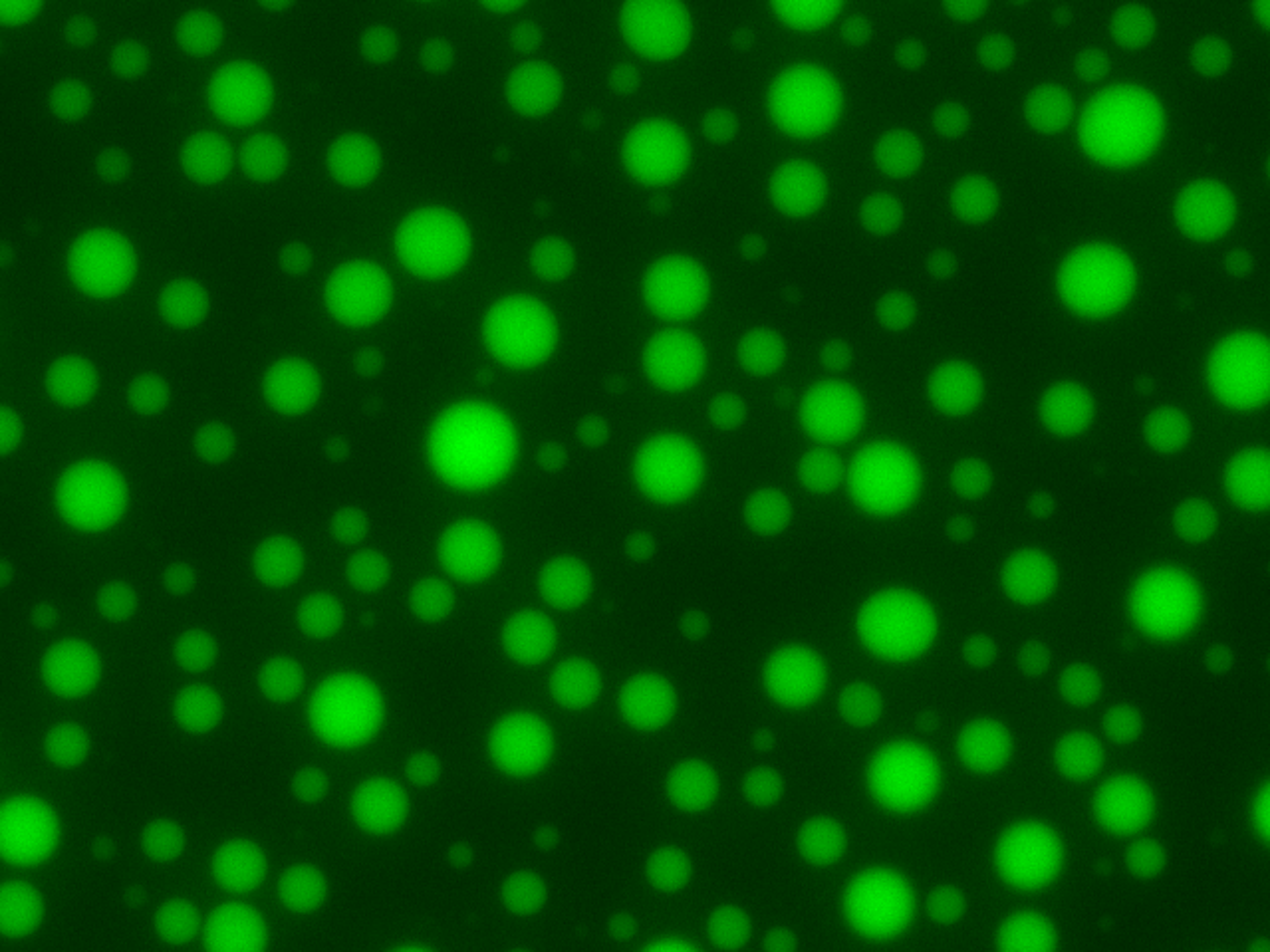

Supplement: Supplementary file 9 — EV Figures Source Data [file 44318_2025_591_MOESM9_ESM.zip › EMBOJ-2025-121908R1_SourceDataForEV/Expanded View Figure 1/EV1D/(a)_01_24h_UBQLN2_None_UBQLN2.tif]

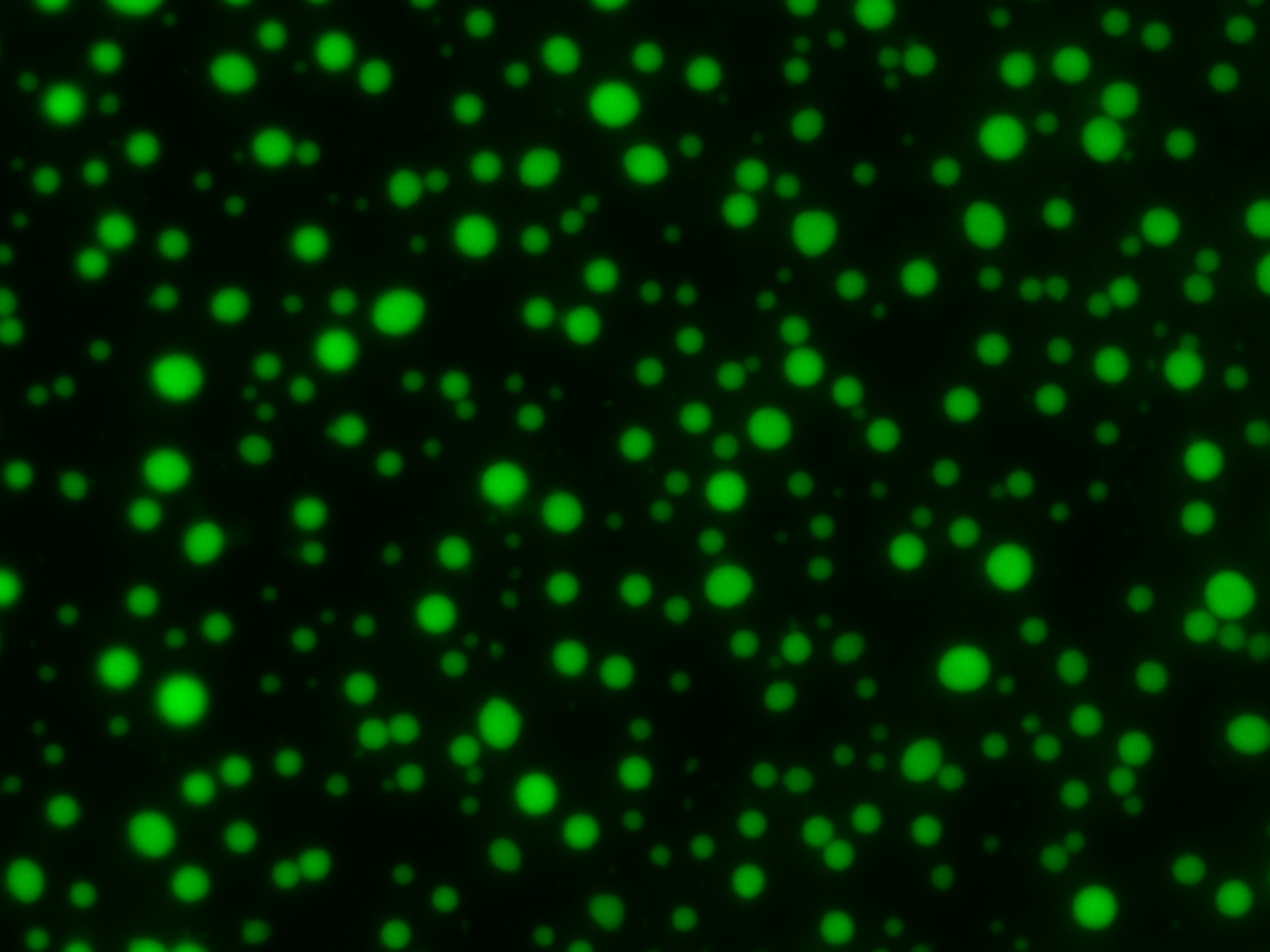

Supplement: Supplementary file 9 — EV Figures Source Data [file 44318_2025_591_MOESM9_ESM.zip › EMBOJ-2025-121908R1_SourceDataForEV/Expanded View Figure 1/EV1D/(a)_22_96h_UBQLN4_16HD_UBQLN4.tif]

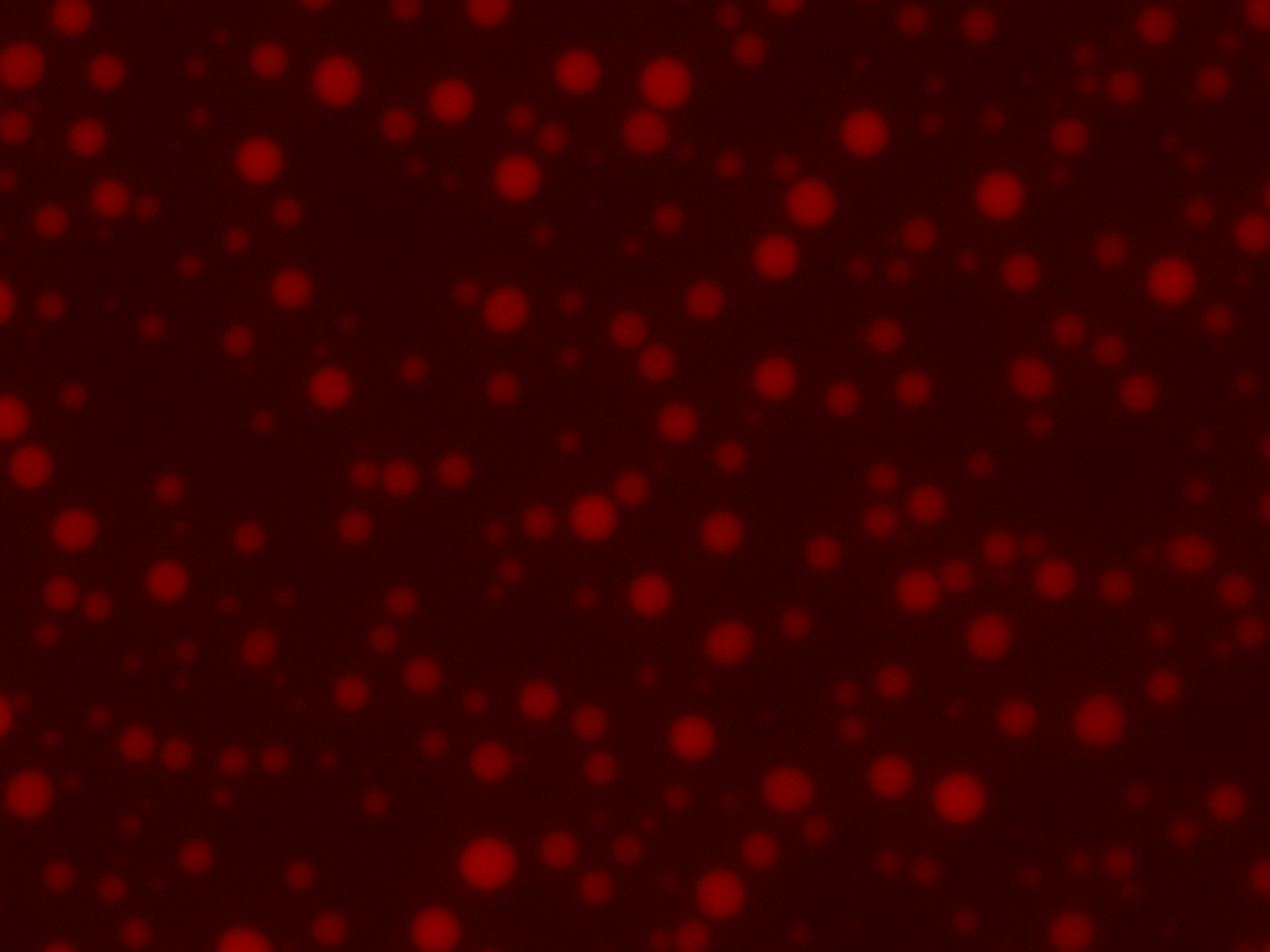

Supplement: Supplementary file 9 — EV Figures Source Data [file 44318_2025_591_MOESM9_ESM.zip › EMBOJ-2025-121908R1_SourceDataForEV/Expanded View Figure 1/EV1D/(a)_23_96h_UBQLN4+aSyn_None_aSyn.tif]

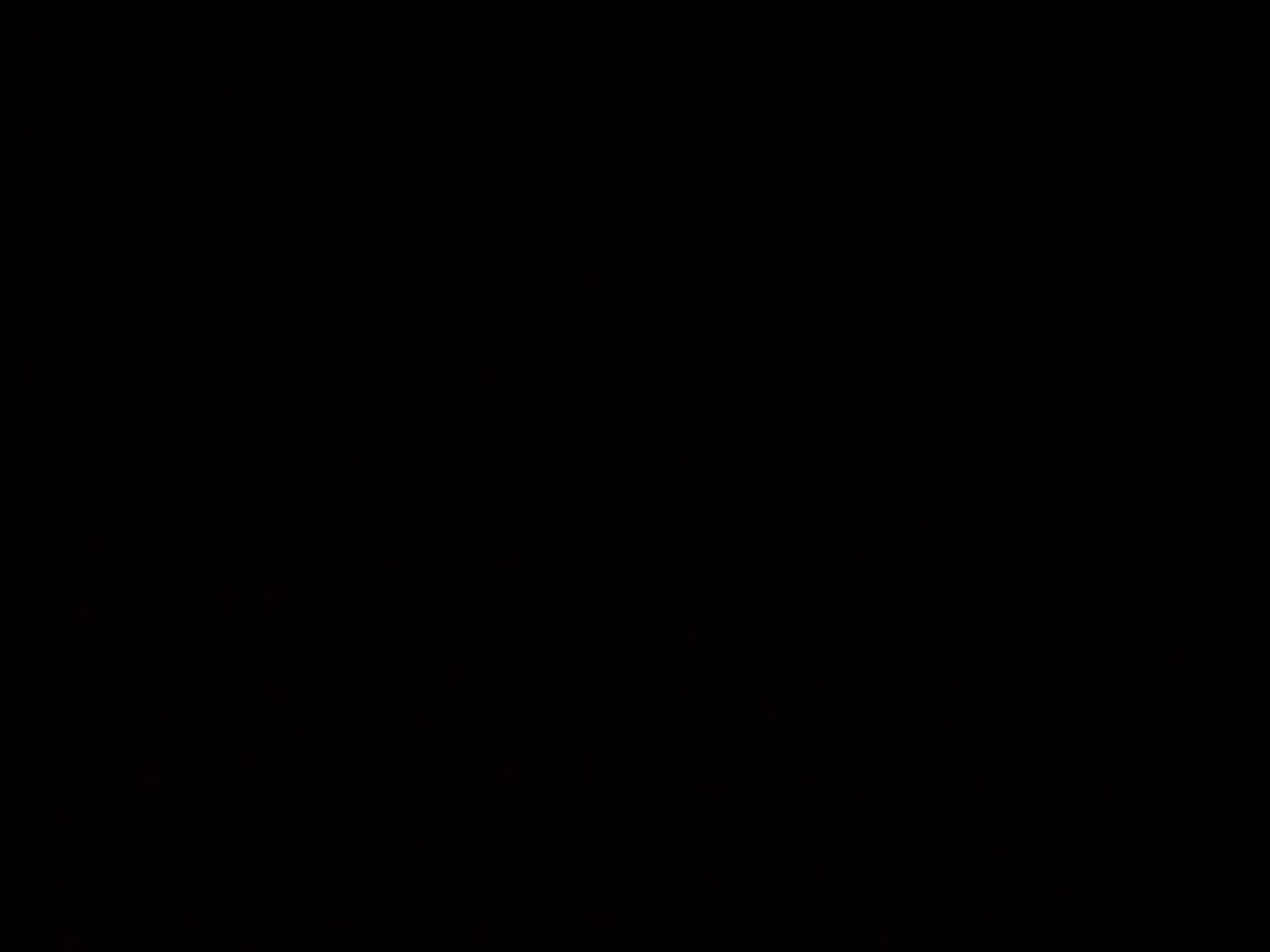

Supplement: Supplementary file 9 — EV Figures Source Data [file 44318_2025_591_MOESM9_ESM.zip › EMBOJ-2025-121908R1_SourceDataForEV/Expanded View Figure 1/EV1D/(a)_20_96h_UBQLN1+aSyn_16HD_aSyn.tif]

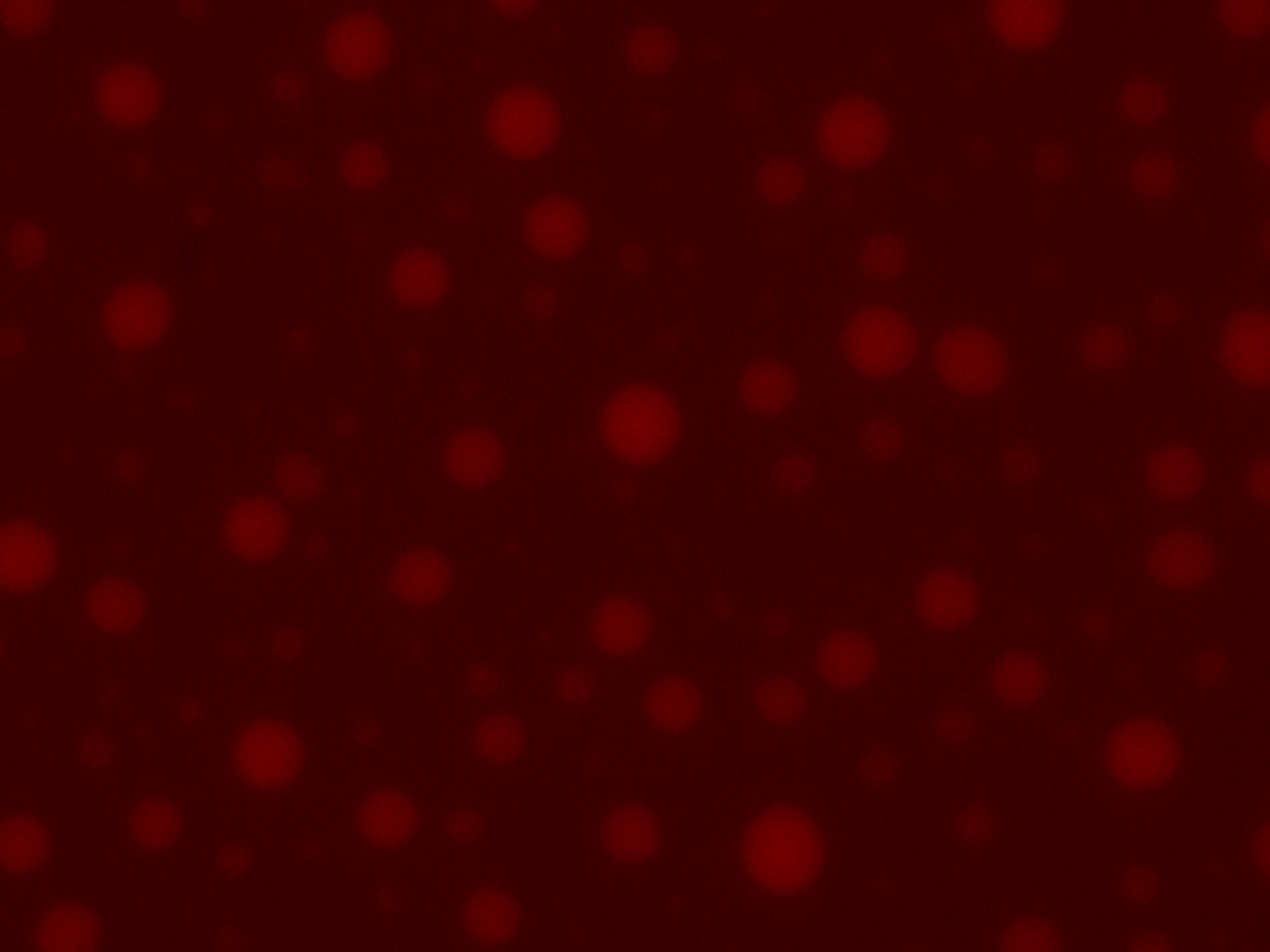

Supplement: Supplementary file 9 — EV Figures Source Data [file 44318_2025_591_MOESM9_ESM.zip › EMBOJ-2025-121908R1_SourceDataForEV/Expanded View Figure 1/EV1D/(a)_19_96h_UBQLN1+aSyn_None_aSyn.tif]

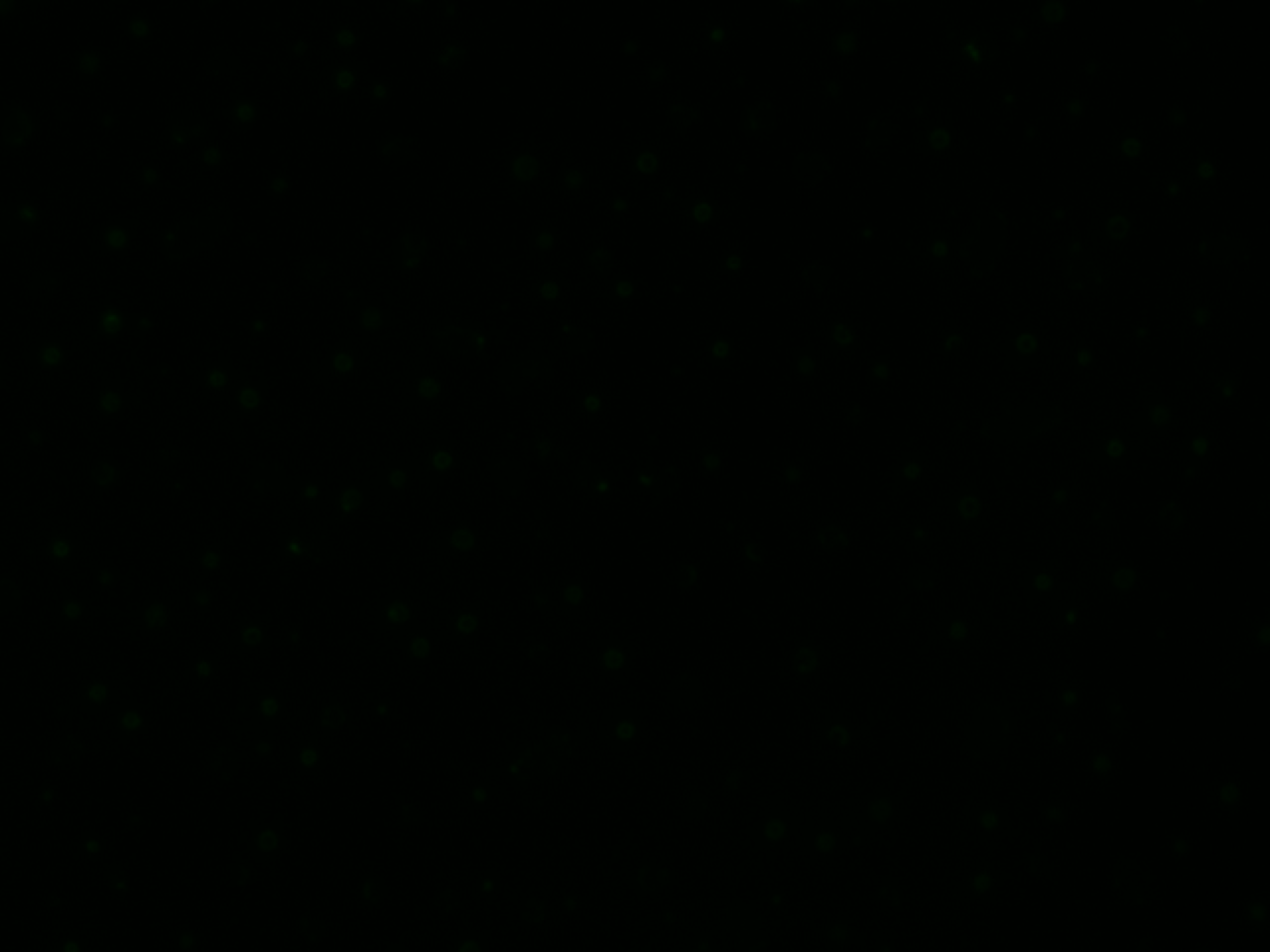

Supplement: Supplementary file 9 — EV Figures Source Data [file 44318_2025_591_MOESM9_ESM.zip › EMBOJ-2025-121908R1_SourceDataForEV/Expanded View Figure 1/EV1D/(a)_18_96h_UBQLN1_16HD_UBQLN1.tif]

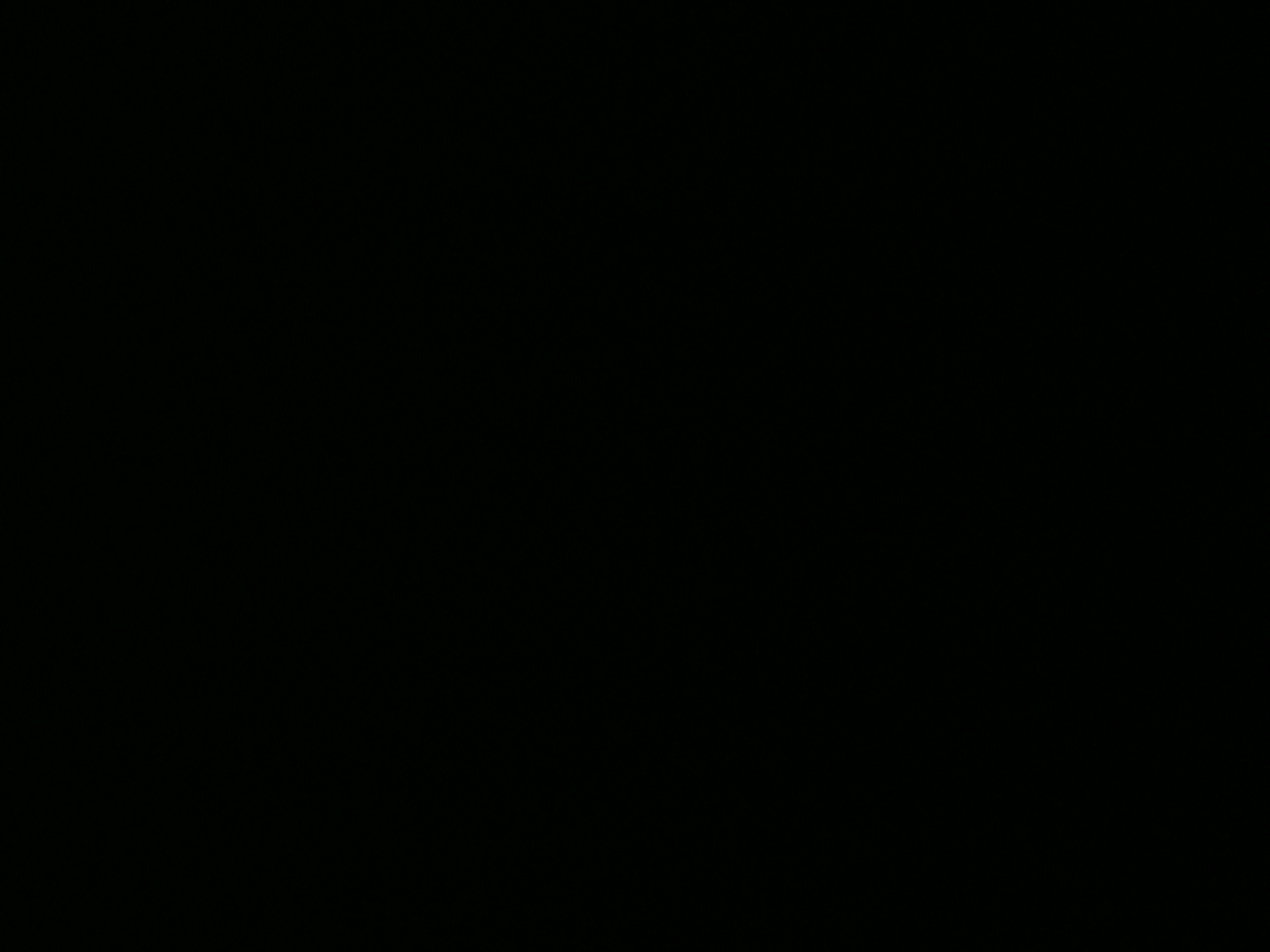

Supplement: Supplementary file 9 — EV Figures Source Data [file 44318_2025_591_MOESM9_ESM.zip › EMBOJ-2025-121908R1_SourceDataForEV/Expanded View Figure 1/EV1D/(a)_08_24h_UBQLN1+aSyn_16HD_aSyn.tif]

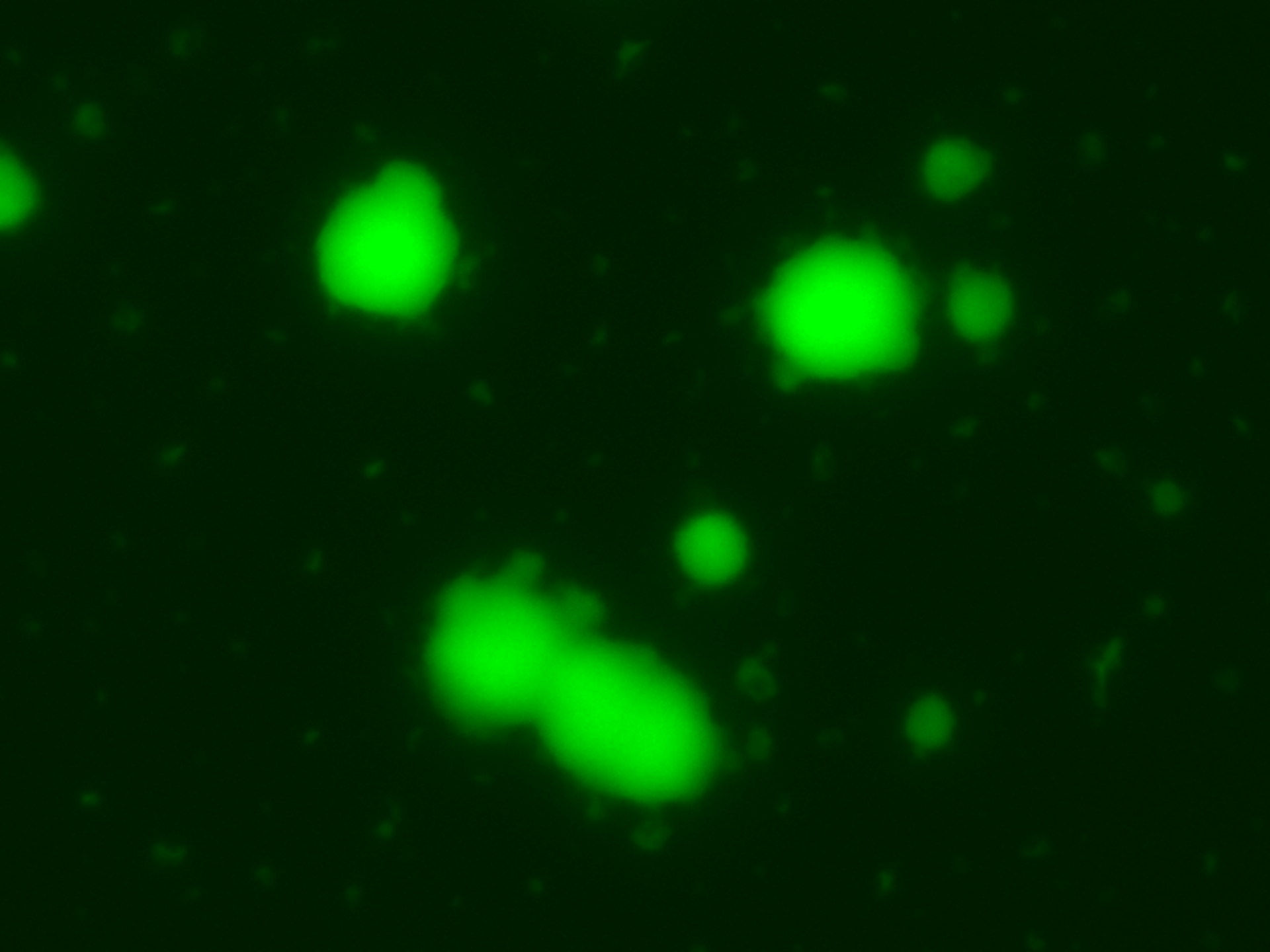

Supplement: Supplementary file 9 — EV Figures Source Data [file 44318_2025_591_MOESM9_ESM.zip › EMBOJ-2025-121908R1_SourceDataForEV/Expanded View Figure 1/EV1D/(a)_15_96h_UBQLN2+aSyn_None_UBQLN2.tif]

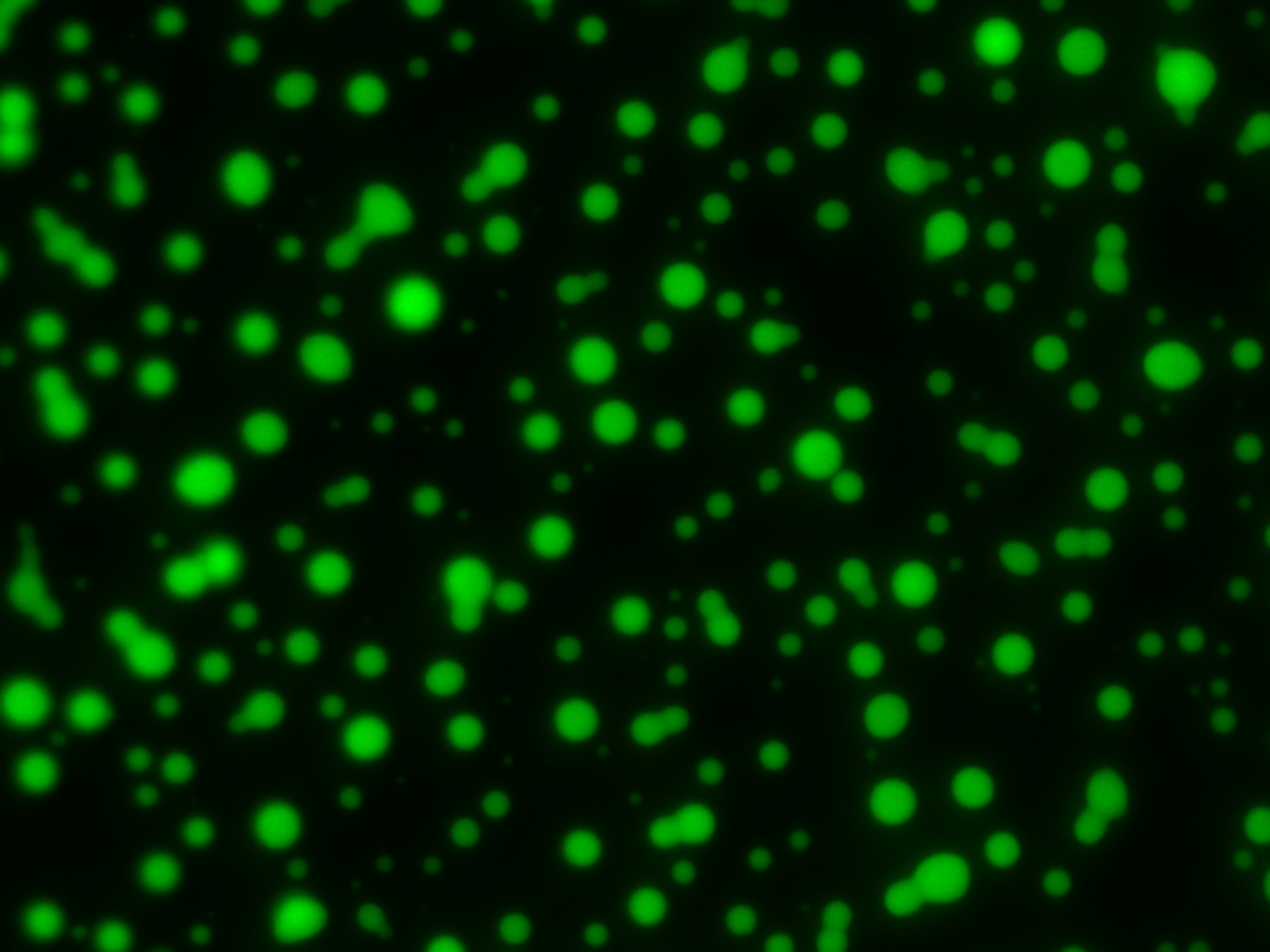

Supplement: Supplementary file 9 — EV Figures Source Data [file 44318_2025_591_MOESM9_ESM.zip › EMBOJ-2025-121908R1_SourceDataForEV/Expanded View Figure 1/EV1D/(a)_10_24h_UBQLN4_16HD_UBQLN4.tif]

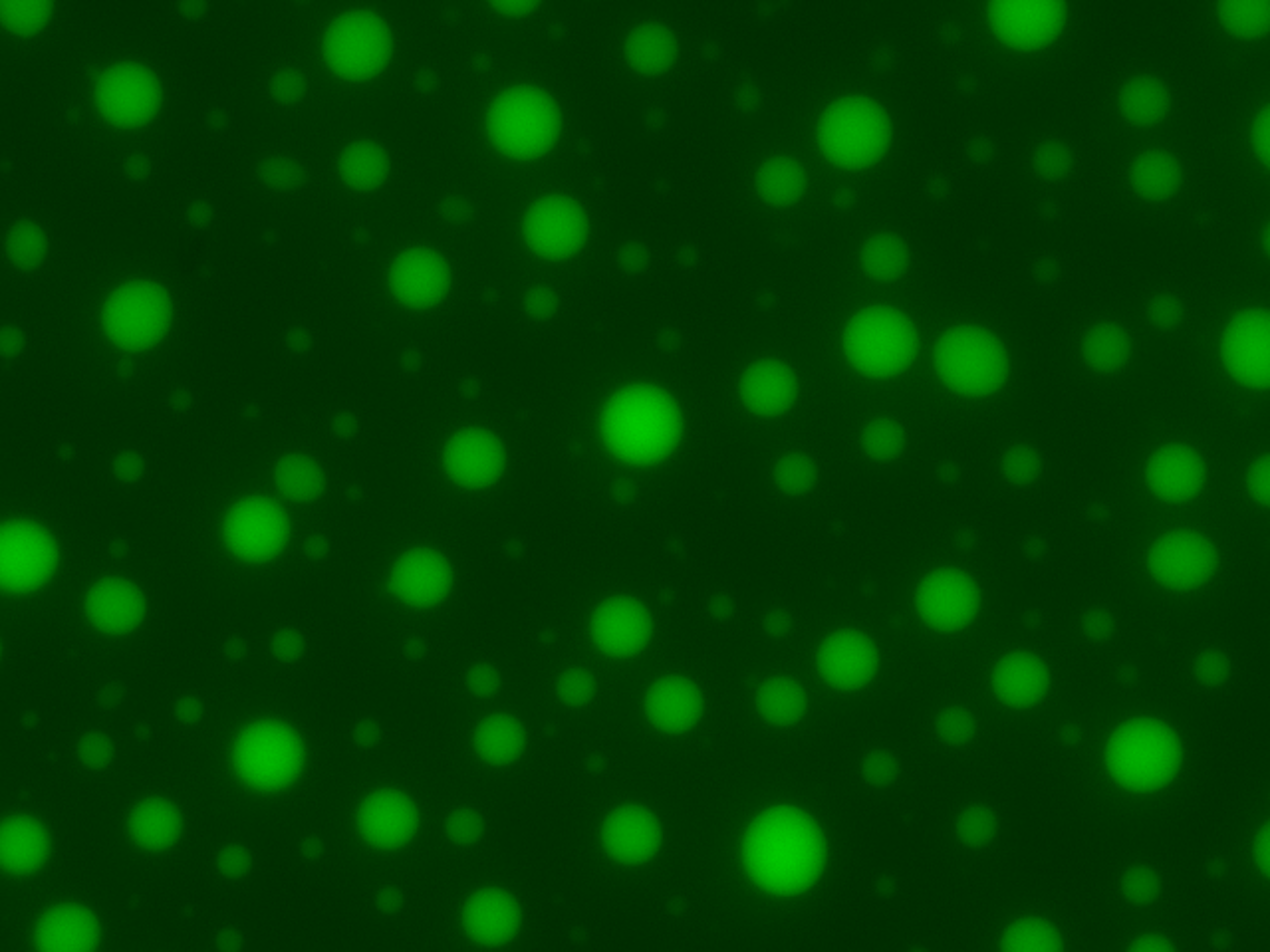

Supplement: Supplementary file 9 — EV Figures Source Data [file 44318_2025_591_MOESM9_ESM.zip › EMBOJ-2025-121908R1_SourceDataForEV/Expanded View Figure 1/EV1D/(a)_19_96h_UBQLN1+aSyn_None_UBQLN1.tif]

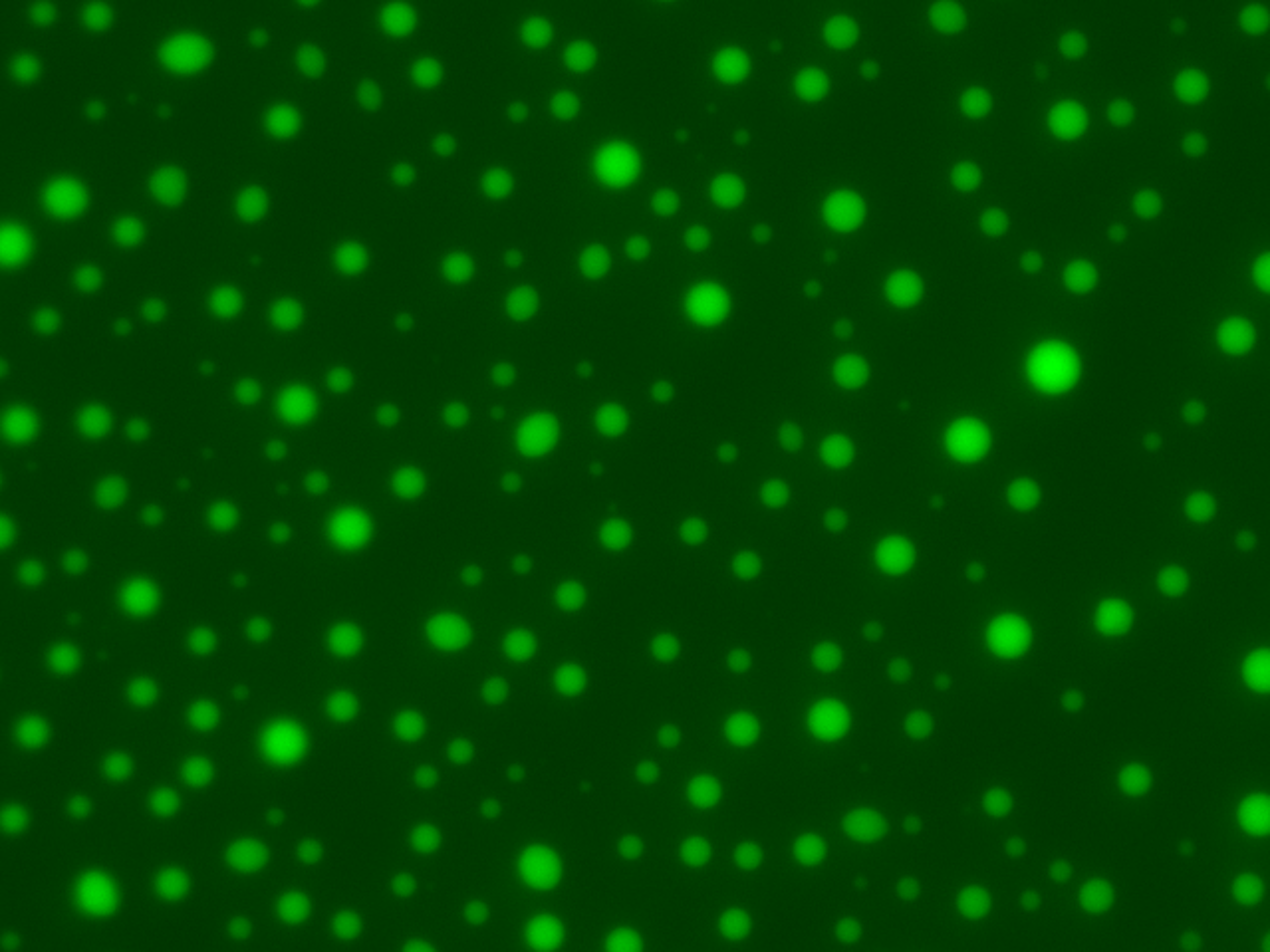

Supplement: Supplementary file 9 — EV Figures Source Data [file 44318_2025_591_MOESM9_ESM.zip › EMBOJ-2025-121908R1_SourceDataForEV/Expanded View Figure 1/EV1D/(a)_11_24h_UBQLN4+aSyn_None_UBQLN4.tif]

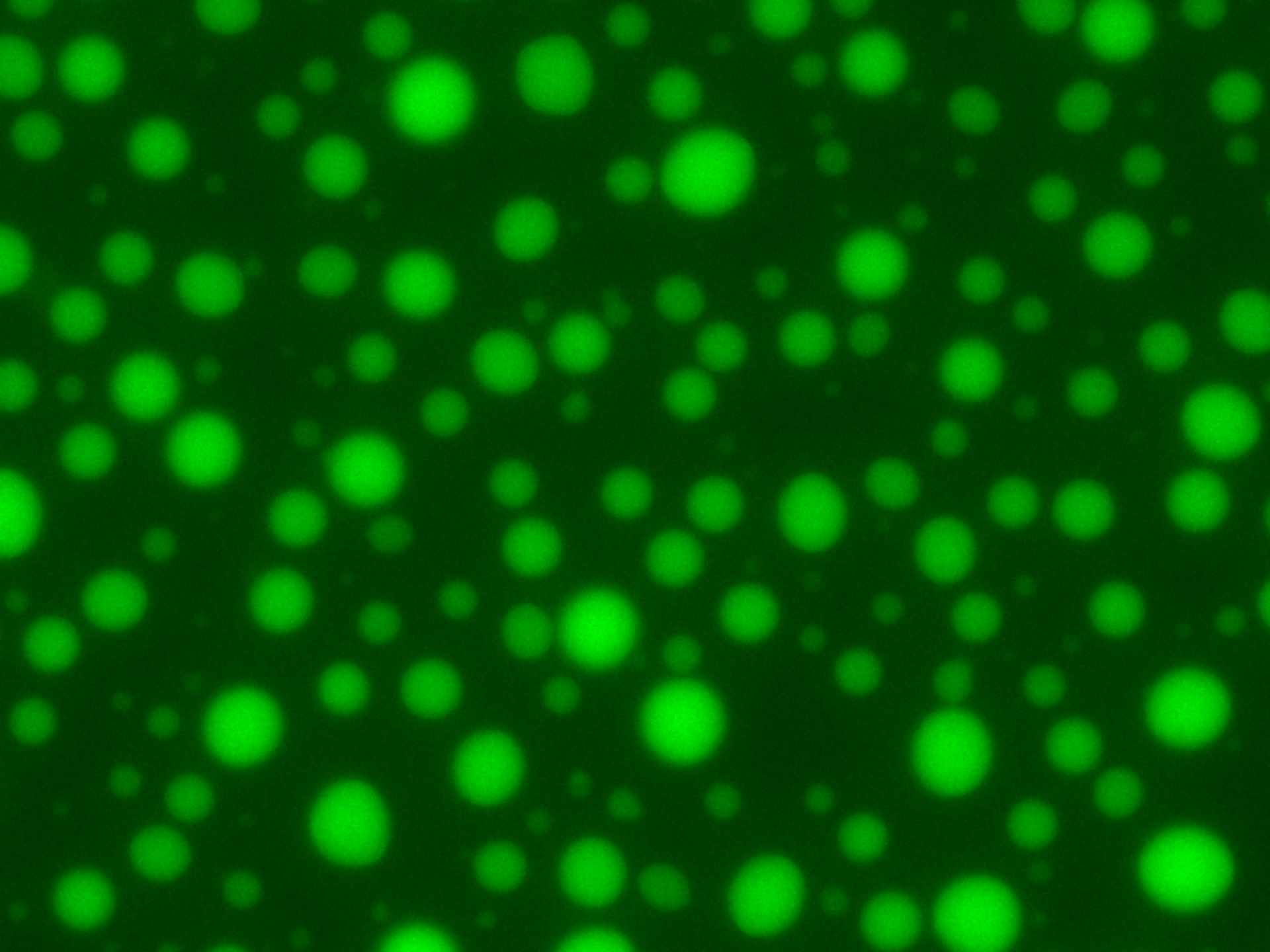

Supplement: Supplementary file 9 — EV Figures Source Data [file 44318_2025_591_MOESM9_ESM.zip › EMBOJ-2025-121908R1_SourceDataForEV/Expanded View Figure 1/EV1D/(a)_07_24h_UBQLN1+aSyn_None_UBQLN 1.tif]

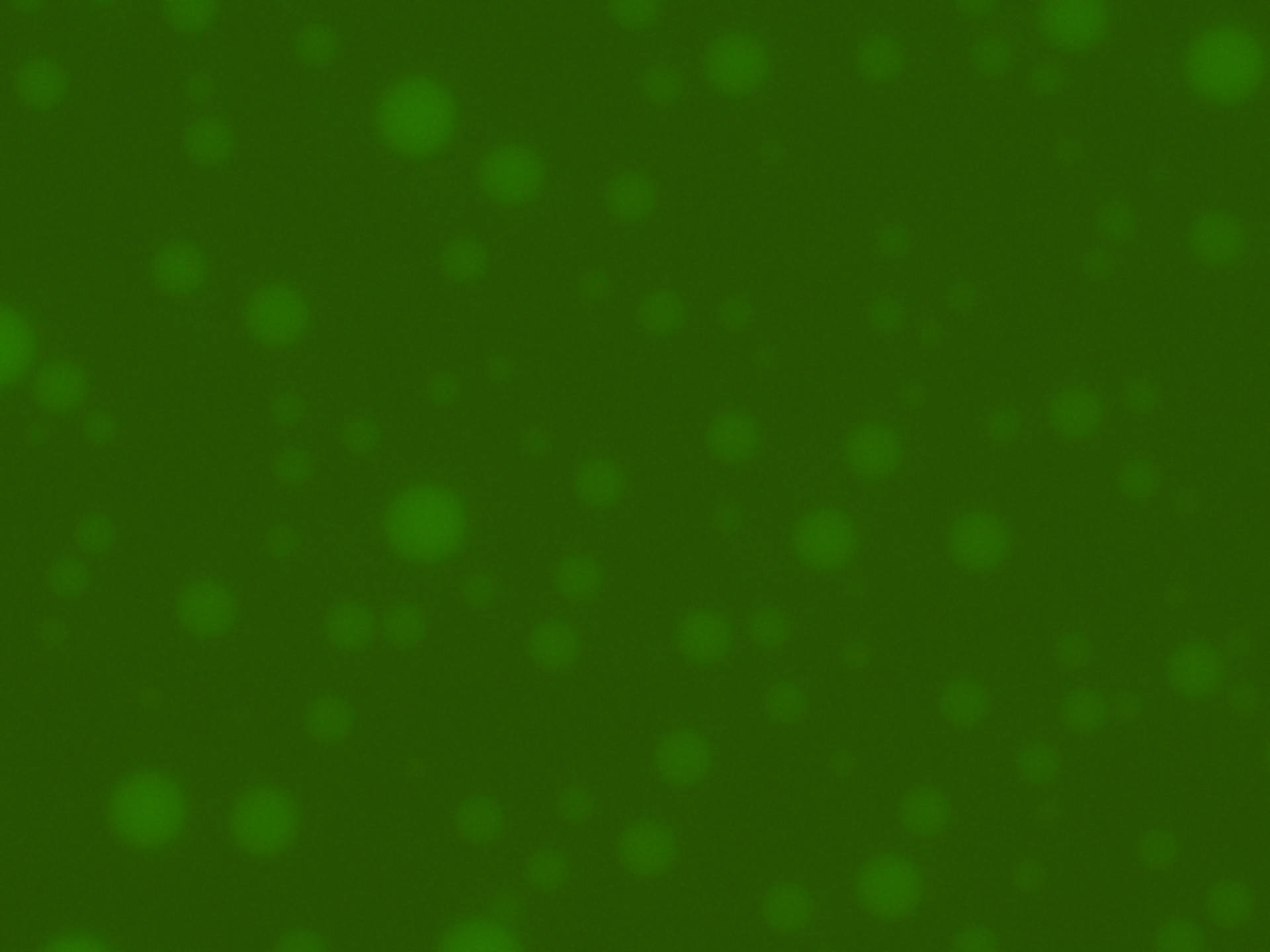

Supplement: Supplementary file 9 — EV Figures Source Data [file 44318_2025_591_MOESM9_ESM.zip › EMBOJ-2025-121908R1_SourceDataForEV/Expanded View Figure 1/EV1B/09_delUBL_Merge.tif]

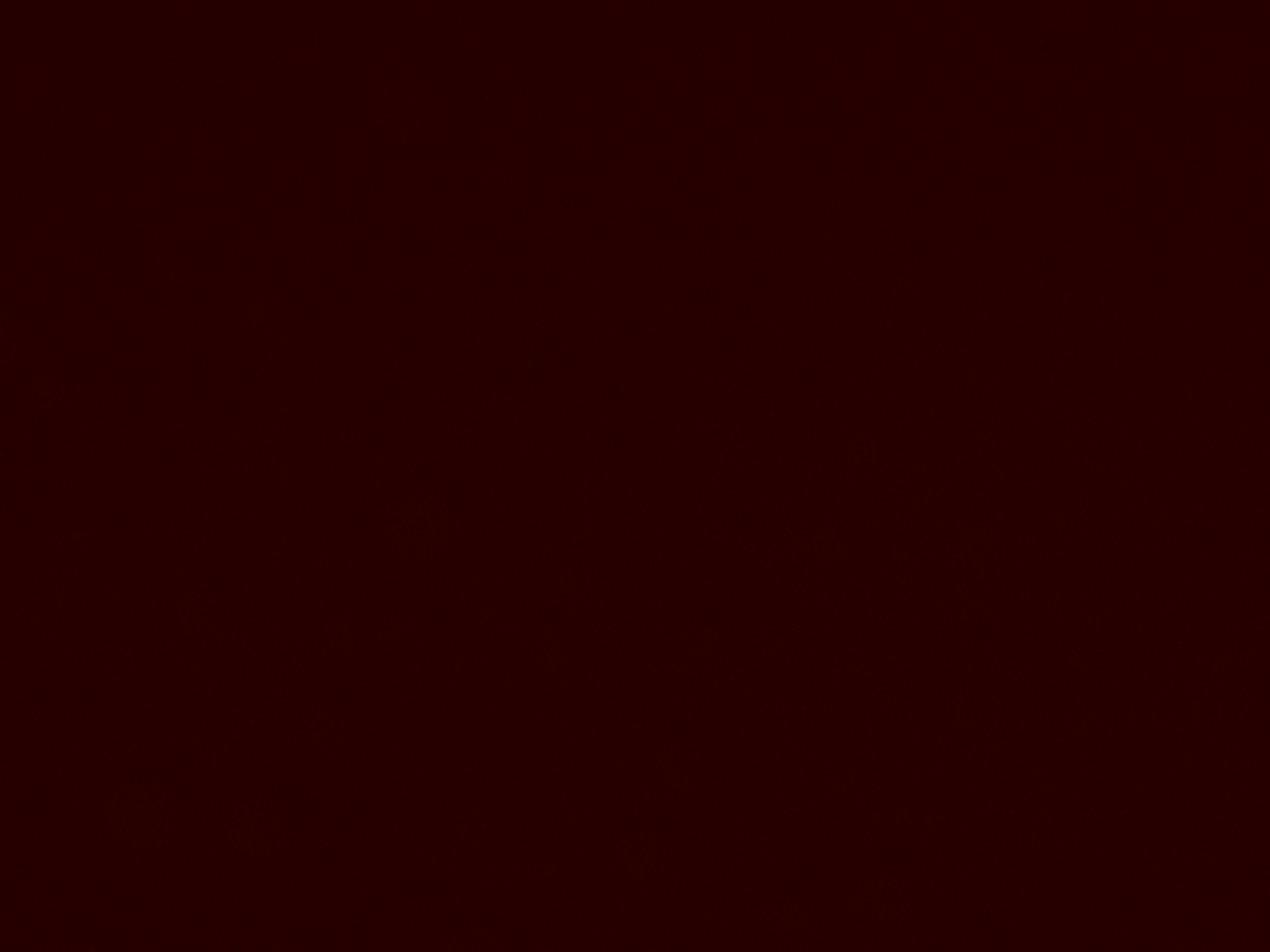

Supplement: Supplementary file 9 — EV Figures Source Data [file 44318_2025_591_MOESM9_ESM.zip › EMBOJ-2025-121908R1_SourceDataForEV/Expanded View Figure 1/EV1B/08_delUBL_╬▒-Syn.tif]

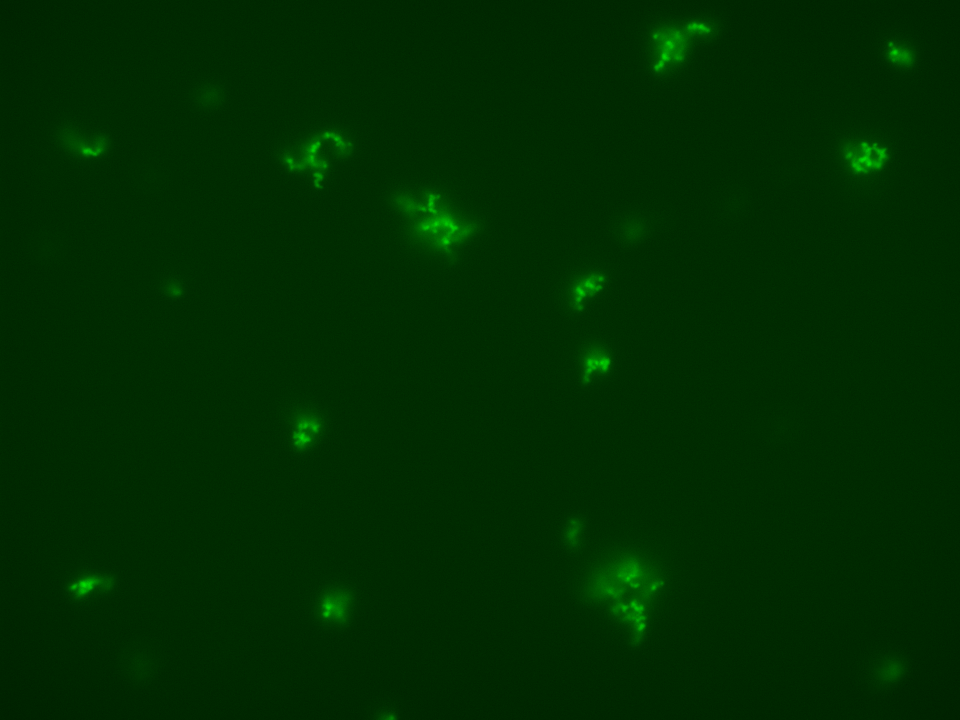

Supplement: Supplementary file 9 — EV Figures Source Data [file 44318_2025_591_MOESM9_ESM.zip › EMBOJ-2025-121908R1_SourceDataForEV/Expanded View Figure 1/EV1B/04_ delSTI1-2_UBQLN2.tif]

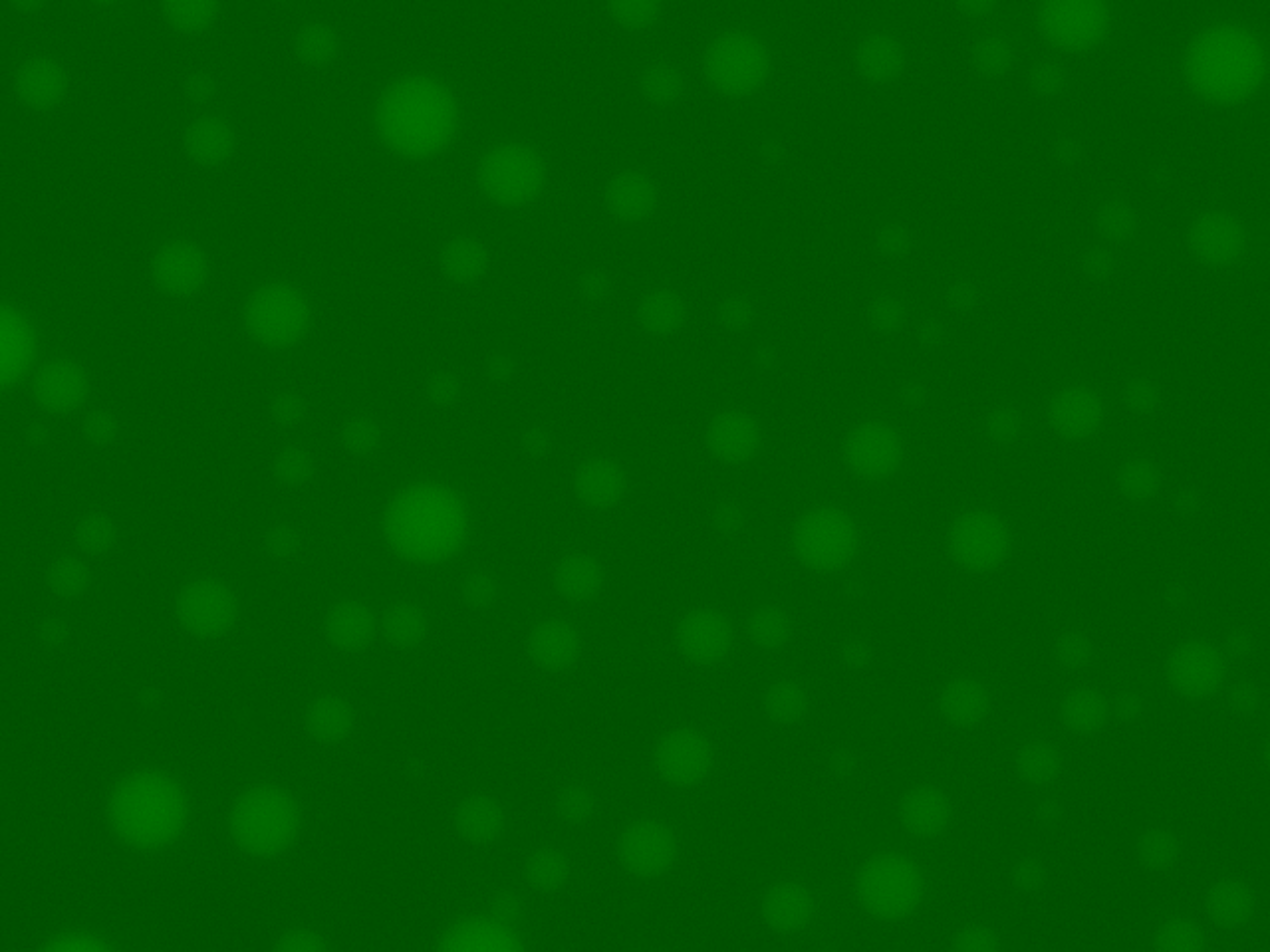

Supplement: Supplementary file 9 — EV Figures Source Data [file 44318_2025_591_MOESM9_ESM.zip › EMBOJ-2025-121908R1_SourceDataForEV/Expanded View Figure 1/EV1B/07_delUBL_UBQLN2.tif]

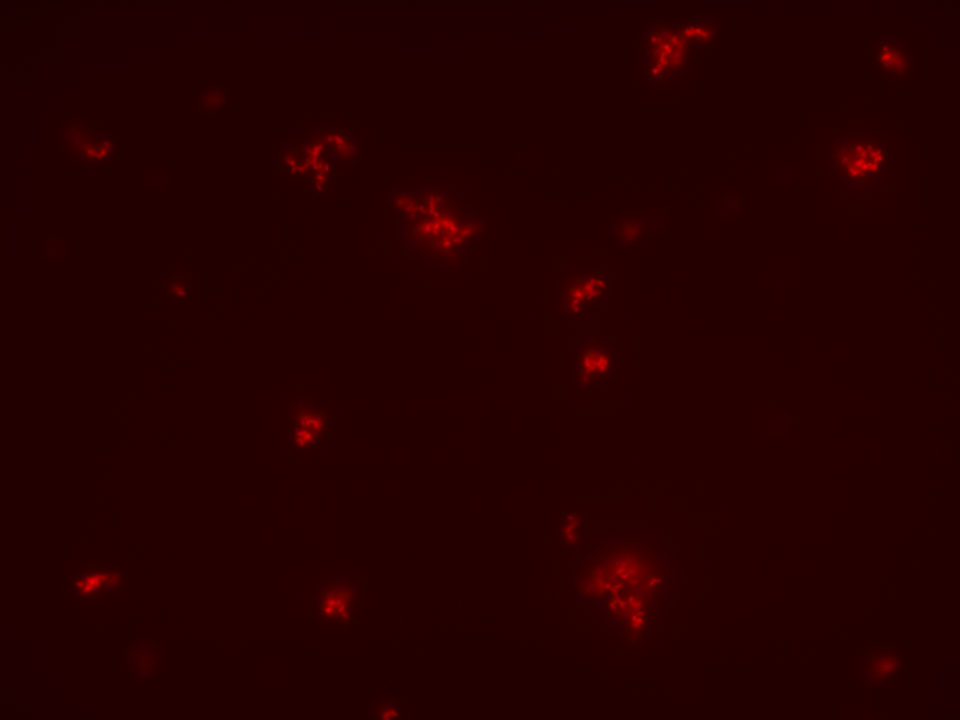

Supplement: Supplementary file 9 — EV Figures Source Data [file 44318_2025_591_MOESM9_ESM.zip › EMBOJ-2025-121908R1_SourceDataForEV/Expanded View Figure 1/EV1B/05_ delSTI1-2_aSyn.tif]

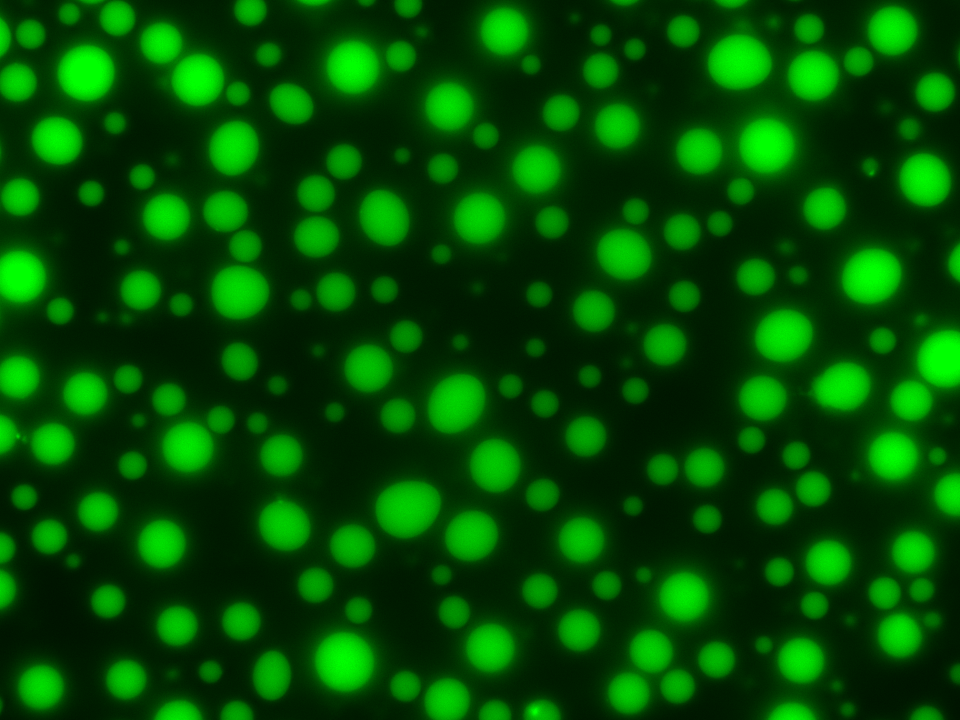

Supplement: Supplementary file 9 — EV Figures Source Data [file 44318_2025_591_MOESM9_ESM.zip › EMBOJ-2025-121908R1_SourceDataForEV/Expanded View Figure 1/EV1B/01_WT_UBQLN2.tif]

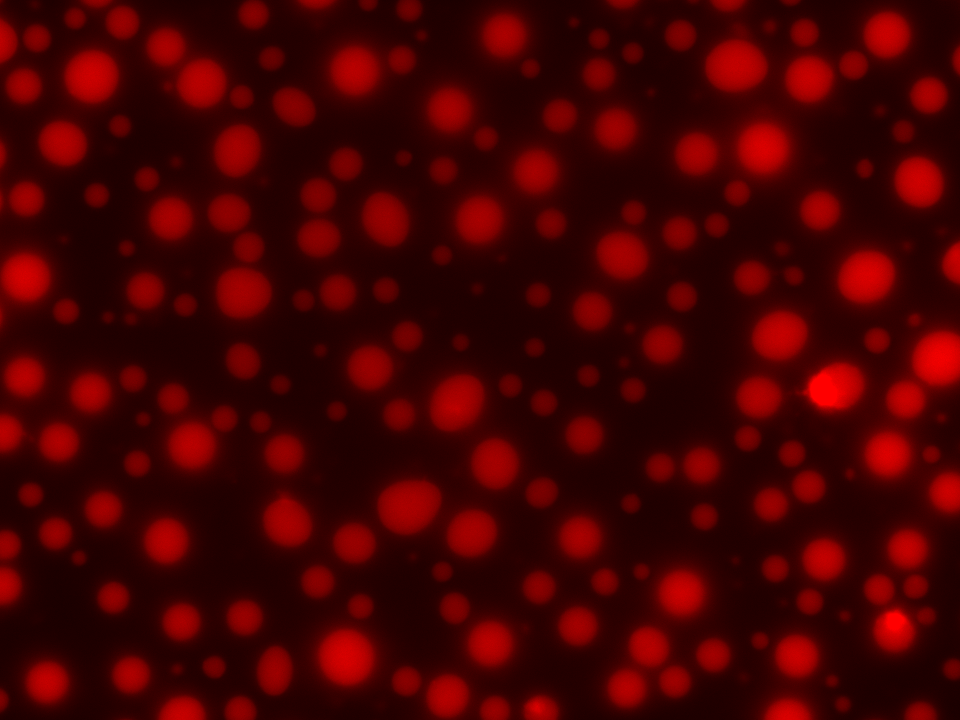

Supplement: Supplementary file 9 — EV Figures Source Data [file 44318_2025_591_MOESM9_ESM.zip › EMBOJ-2025-121908R1_SourceDataForEV/Expanded View Figure 1/EV1B/02_WT_aSyn.tif]

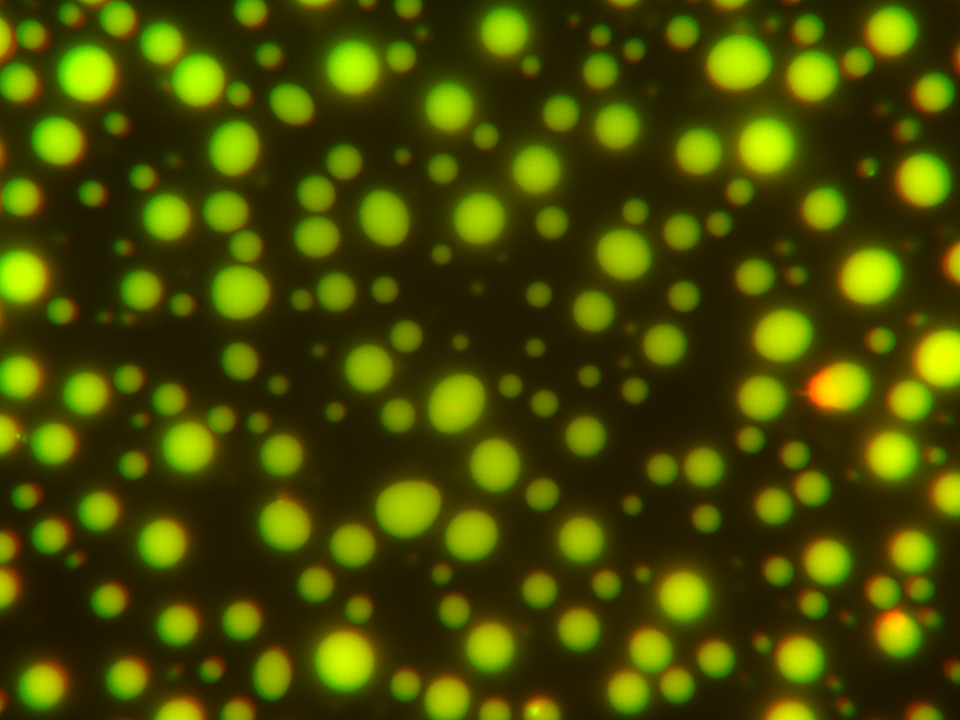

Supplement: Supplementary file 9 — EV Figures Source Data [file 44318_2025_591_MOESM9_ESM.zip › EMBOJ-2025-121908R1_SourceDataForEV/Expanded View Figure 1/EV1B/03_WT_Merge.tif]

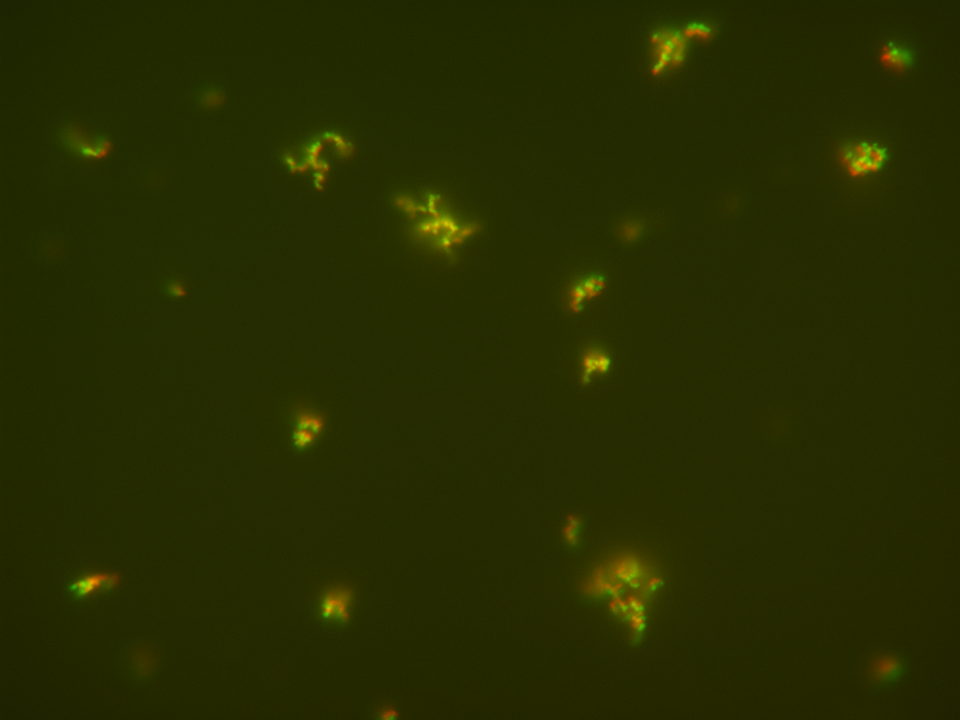

Supplement: Supplementary file 9 — EV Figures Source Data [file 44318_2025_591_MOESM9_ESM.zip › EMBOJ-2025-121908R1_SourceDataForEV/Expanded View Figure 1/EV1B/06_ delSTI1-2_Merge.tif]

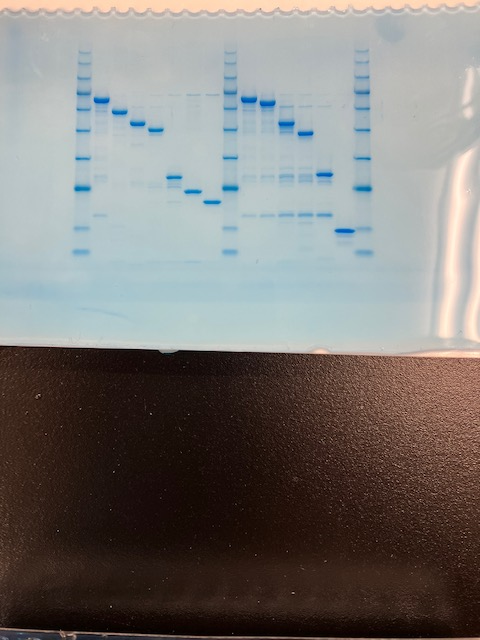

Supplement: Supplementary file 9 — EV Figures Source Data [file 44318_2025_591_MOESM9_ESM.zip › EMBOJ-2025-121908R1_SourceDataForEV/Expanded View Figure 2/EV2B/(b)_CBB.tif]

Source Data of Expanded View Fig. 2B

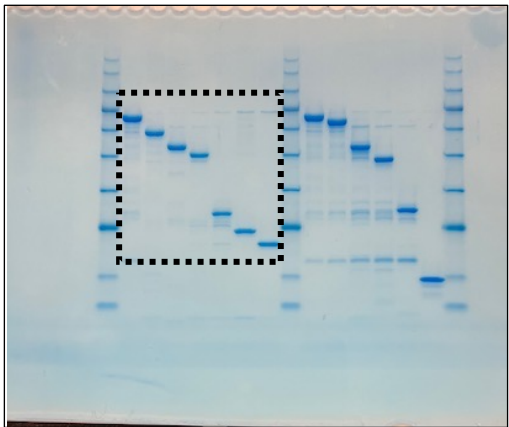

CBB staining

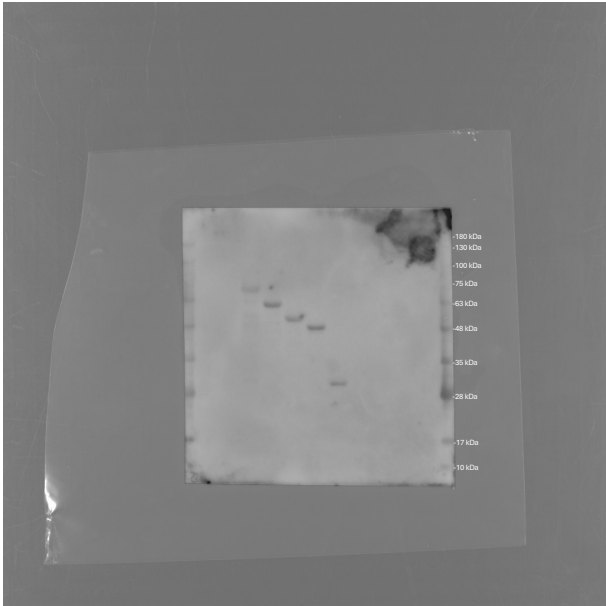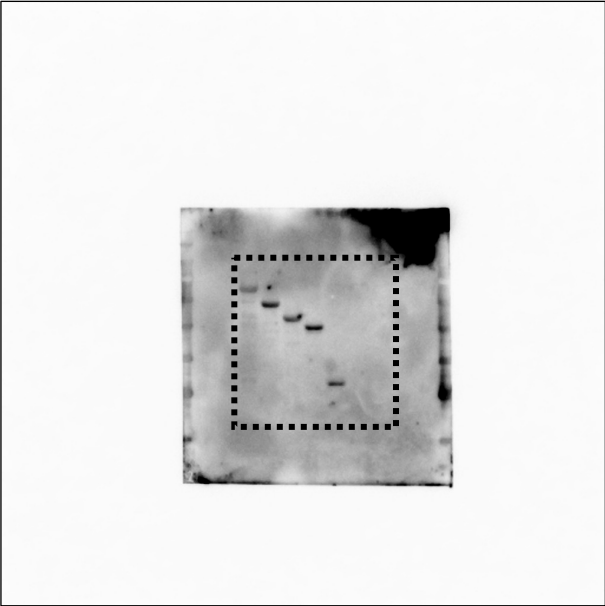

anti- $\alpha$ -Syn

Supplement: Supplementary file 9 — EV Figures Source Data [file 44318_2025_591_MOESM9_ESM.zip › EMBOJ-2025-121908R1_SourceDataForEV/Expanded View Figure 2/EV2B/Highlight of crop area.pdf]

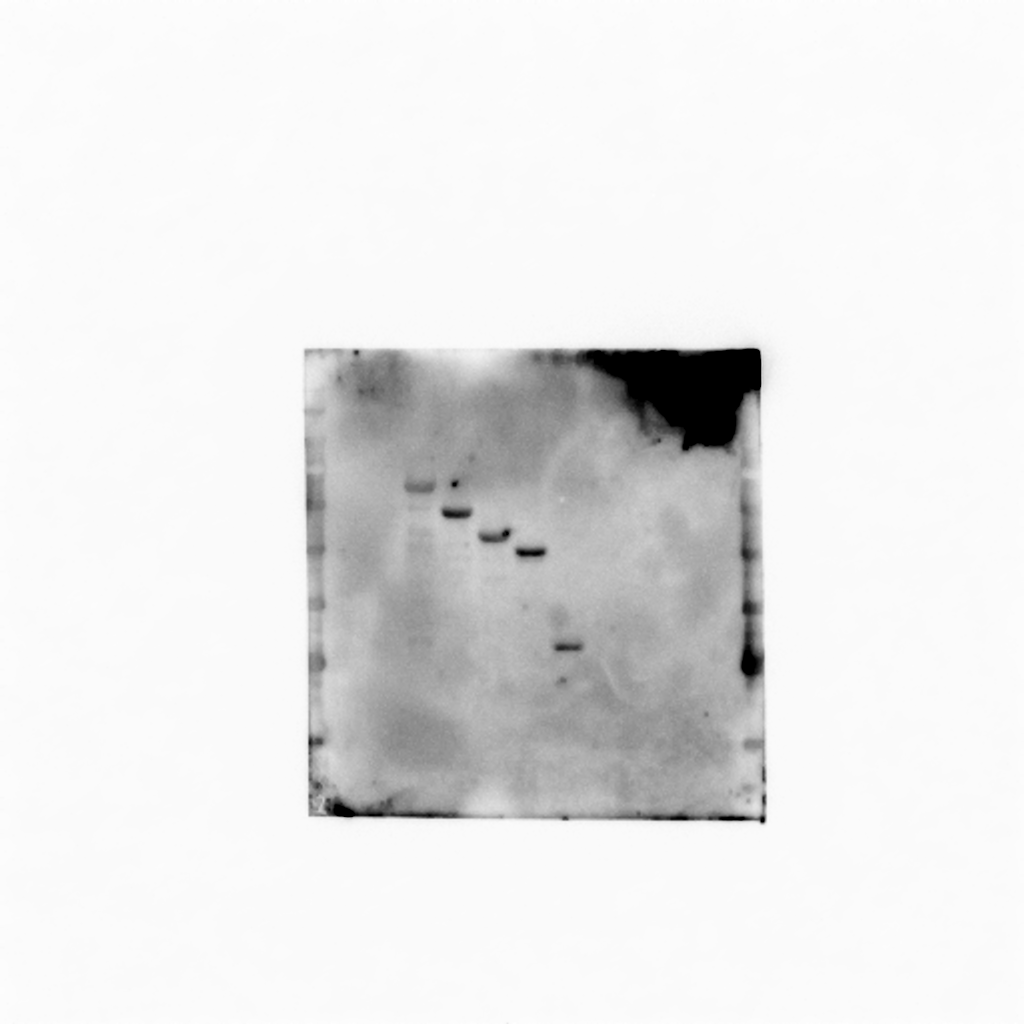

Supplement: Supplementary file 9 — EV Figures Source Data [file 44318_2025_591_MOESM9_ESM.zip › EMBOJ-2025-121908R1_SourceDataForEV/Expanded View Figure 2/EV2B/(c)_╬▒-Syn.tif]

Source Data of Expanded View Fig. 2C

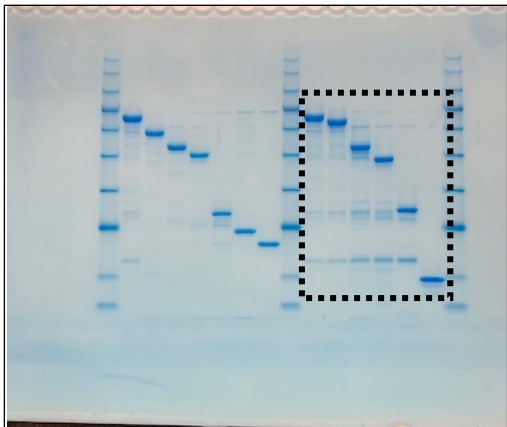

CBB staining

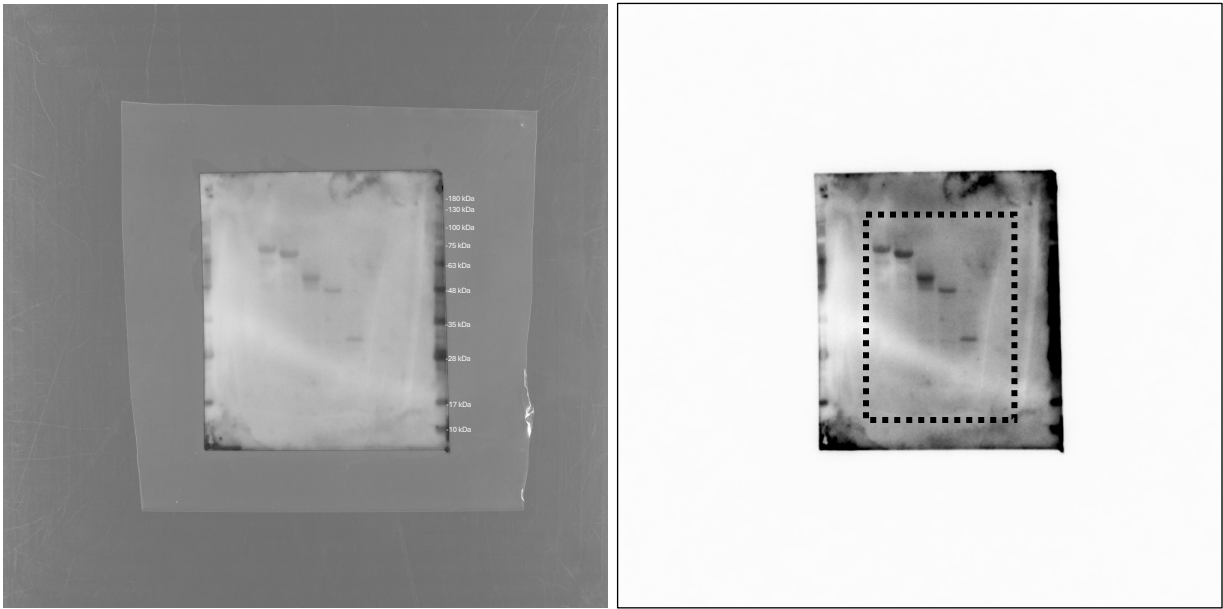

anti- $\alpha$ -Syn

Supplement: Supplementary file 9 — EV Figures Source Data [file 44318_2025_591_MOESM9_ESM.zip › EMBOJ-2025-121908R1_SourceDataForEV/Expanded View Figure 2/EV2C/Highlight of crop area.pdf]

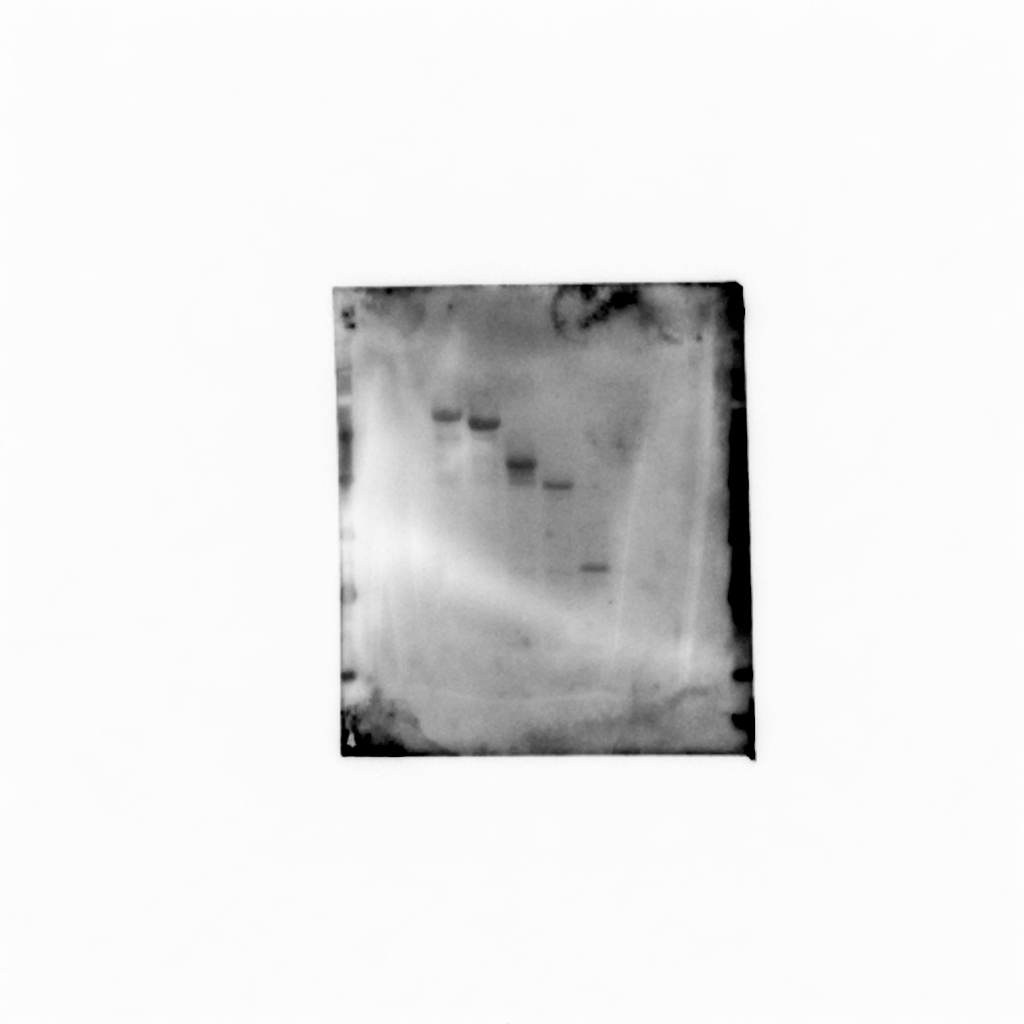

Supplement: Supplementary file 9 — EV Figures Source Data [file 44318_2025_591_MOESM9_ESM.zip › EMBOJ-2025-121908R1_SourceDataForEV/Expanded View Figure 2/EV2C/(c)_╬▒-Syn.tif]

# Source Data of Expanded View Fig. 2A

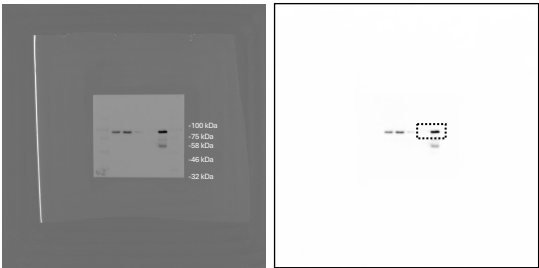

anti-UBQLN2

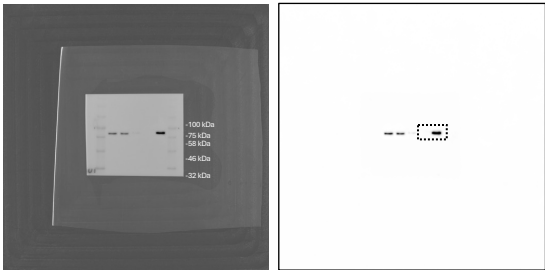

anti-UBQLN1

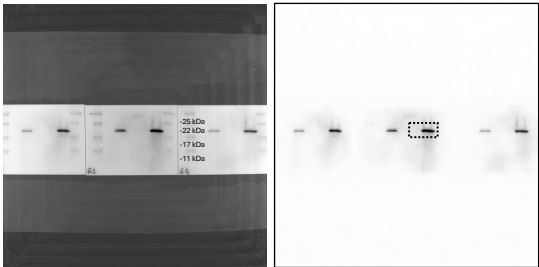

anti-FLAG

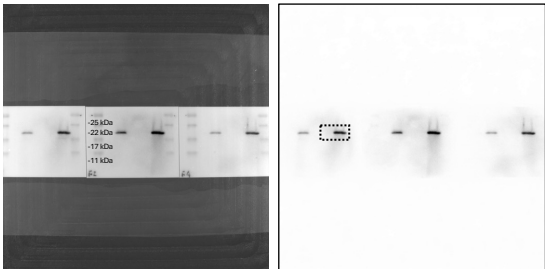

anti-FLAG

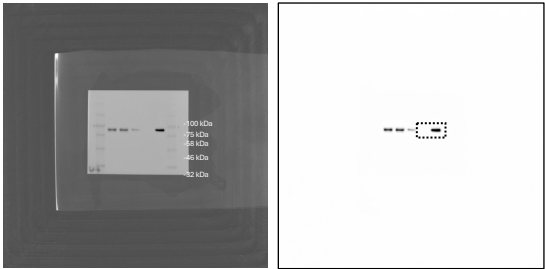

anti-UBQLN4

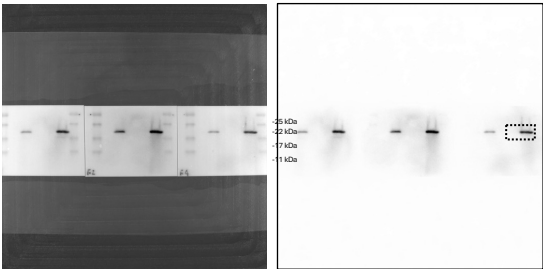

anti-FLAG

Supplement: Supplementary file 9 — EV Figures Source Data [file 44318_2025_591_MOESM9_ESM.zip › EMBOJ-2025-121908R1_SourceDataForEV/Expanded View Figure 2/EV2A/05_Highlight of crop area.pdf]

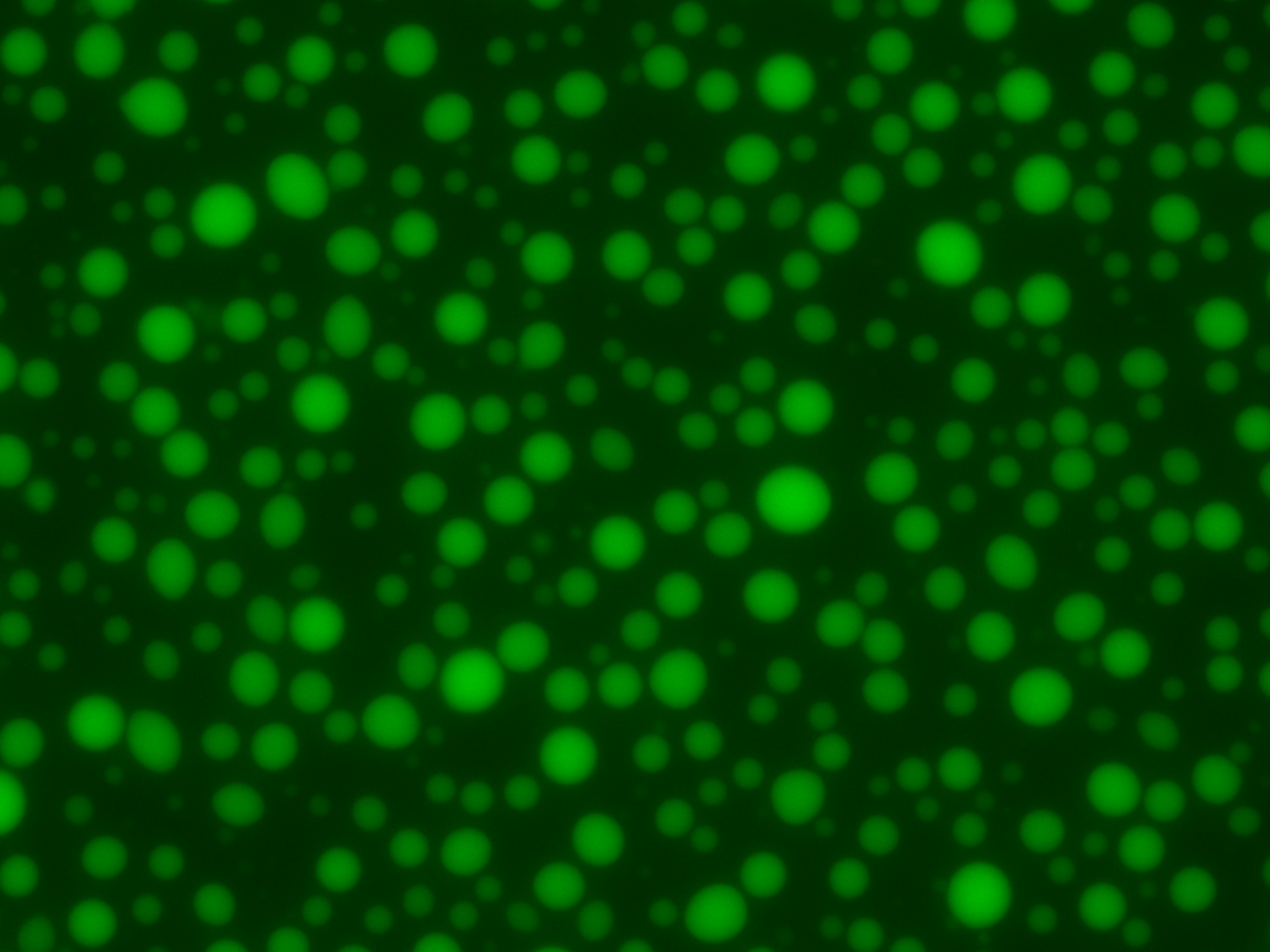

Supplement: Supplementary file 9 — EV Figures Source Data [file 44318_2025_591_MOESM9_ESM.zip › EMBOJ-2025-121908R1_SourceDataForEV/Expanded View Figure 5/EV5B/09_SO82_24h_UBQLN2_x80.tif]

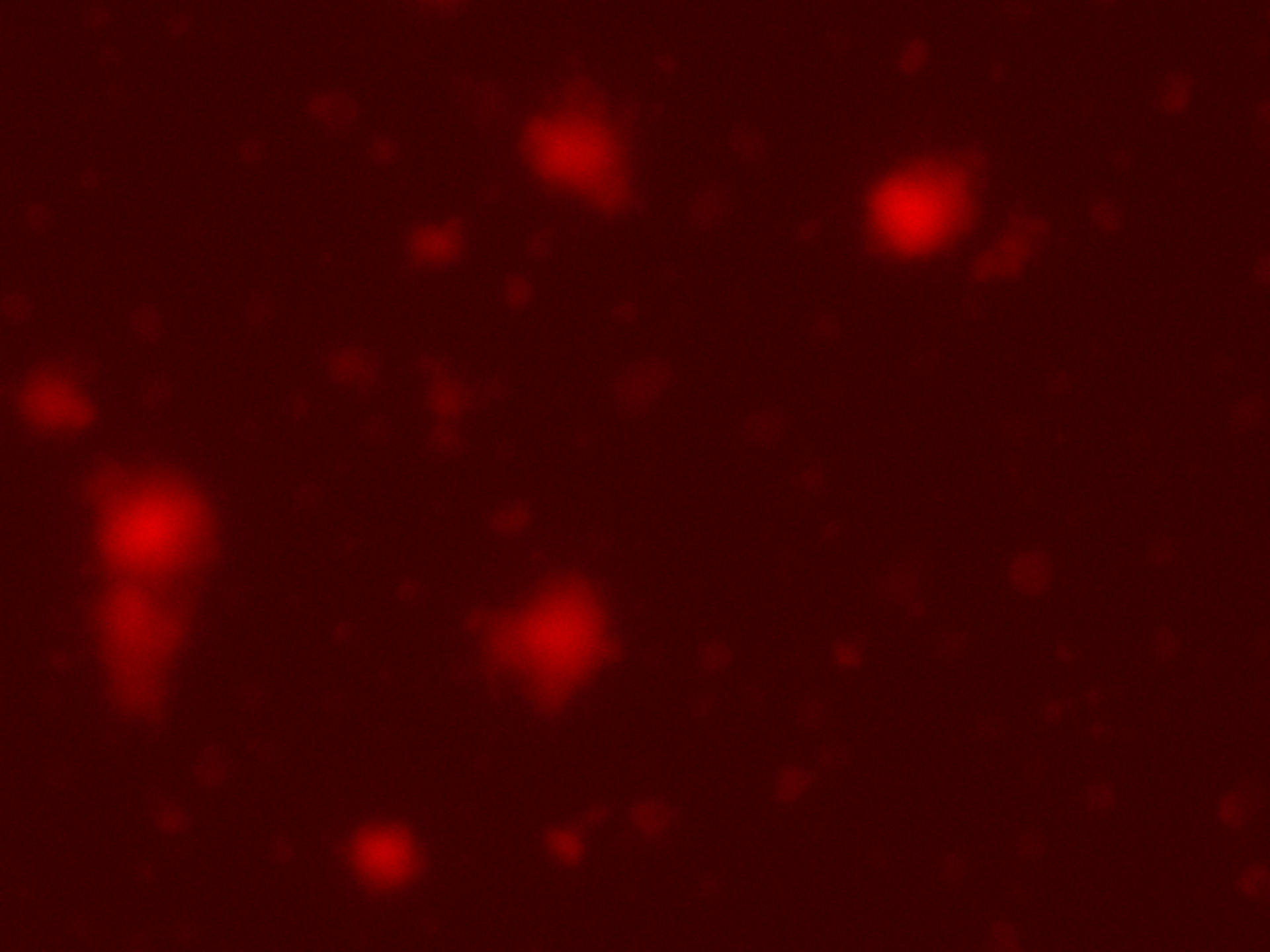

Supplement: Supplementary file 9 — EV Figures Source Data [file 44318_2025_591_MOESM9_ESM.zip › EMBOJ-2025-121908R1_SourceDataForEV/Expanded View Figure 5/EV5B/12_SO82_96h_aSyn_x120.tif]

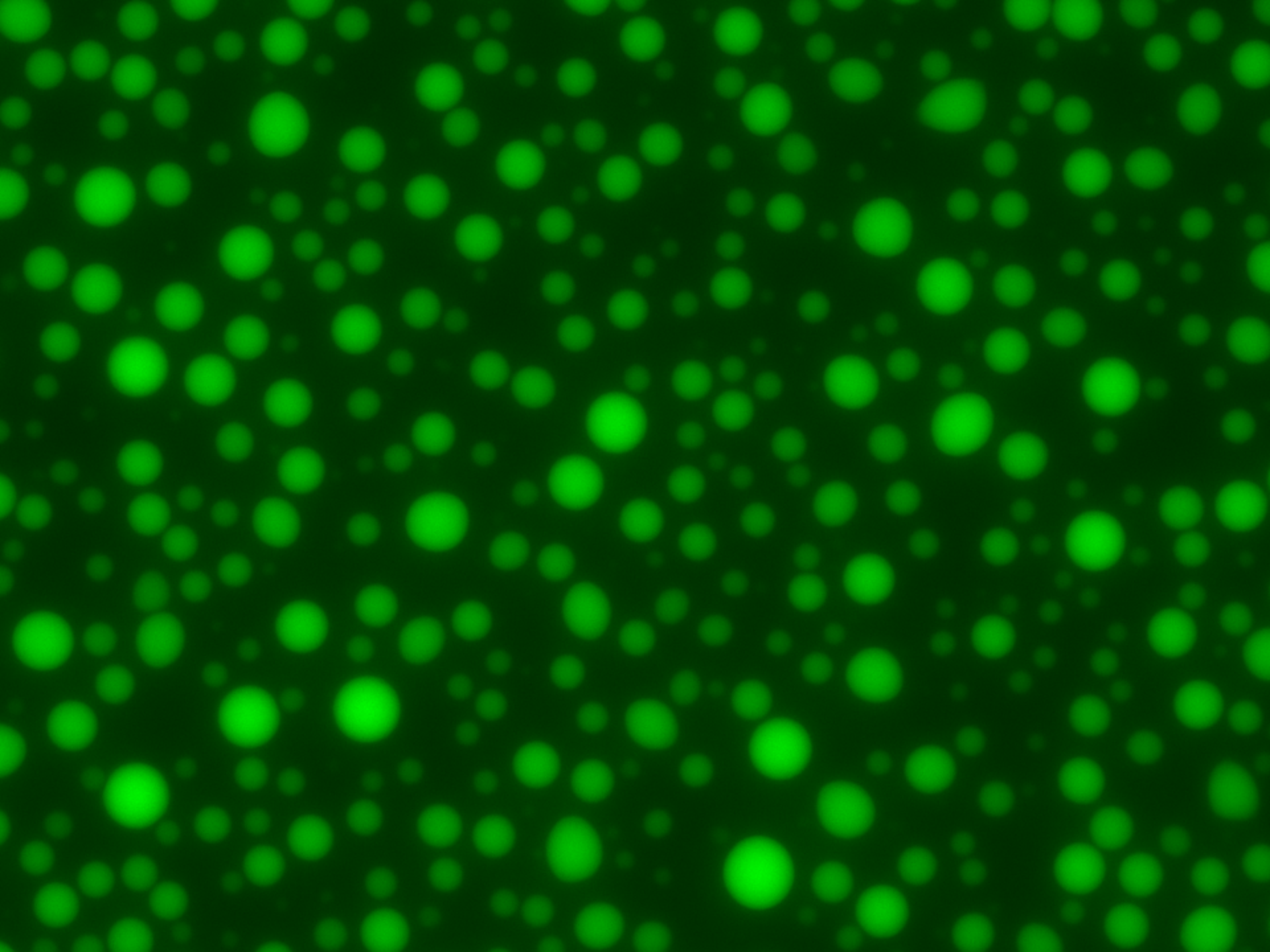

Supplement: Supplementary file 9 — EV Figures Source Data [file 44318_2025_591_MOESM9_ESM.zip › EMBOJ-2025-121908R1_SourceDataForEV/Expanded View Figure 5/EV5B/05_SO286_24h_UBQLN2_x80.tif]

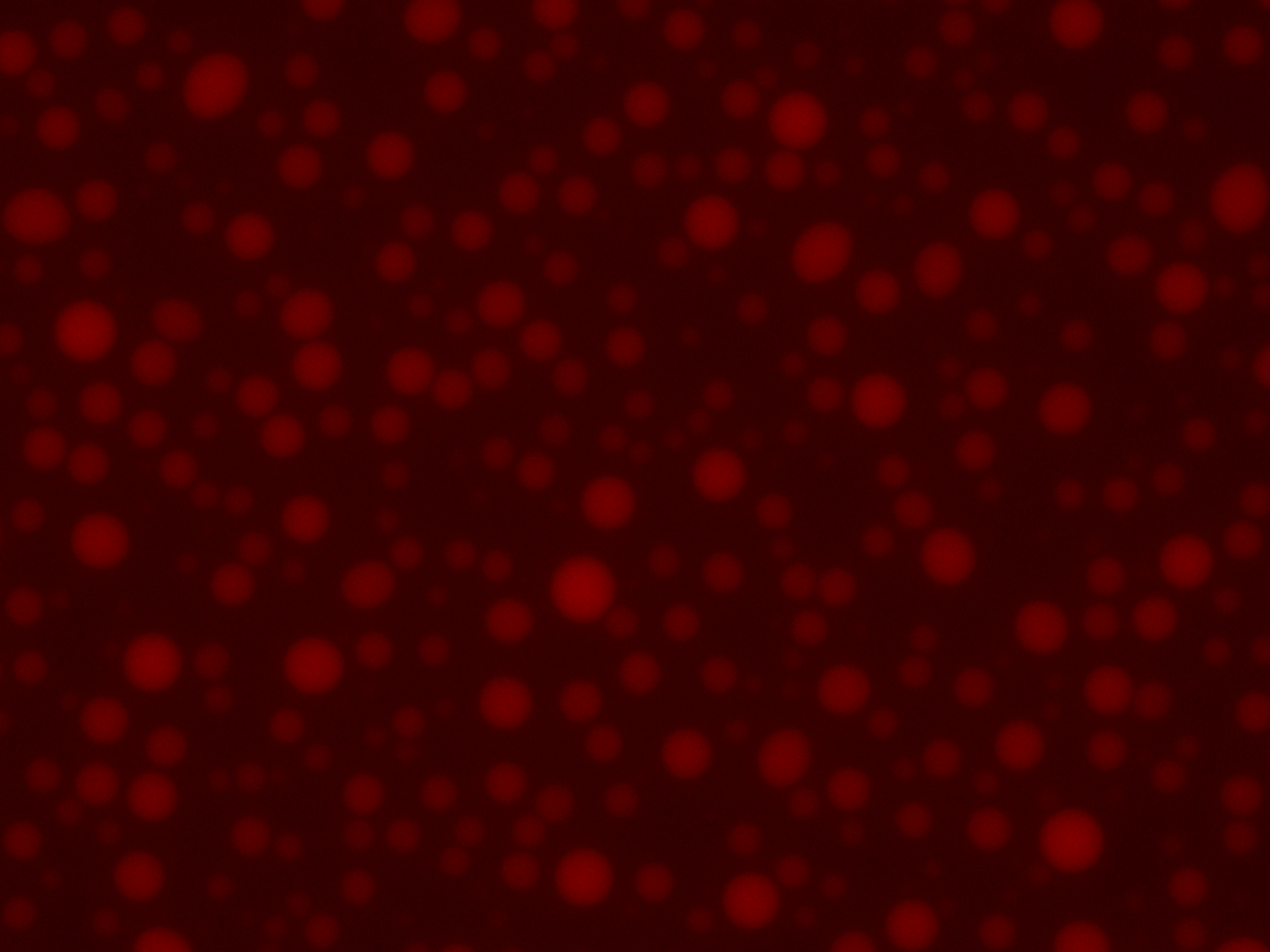

Supplement: Supplementary file 9 — EV Figures Source Data [file 44318_2025_591_MOESM9_ESM.zip › EMBOJ-2025-121908R1_SourceDataForEV/Expanded View Figure 5/EV5B/02_Control_24h_aSyn_x80.tif]

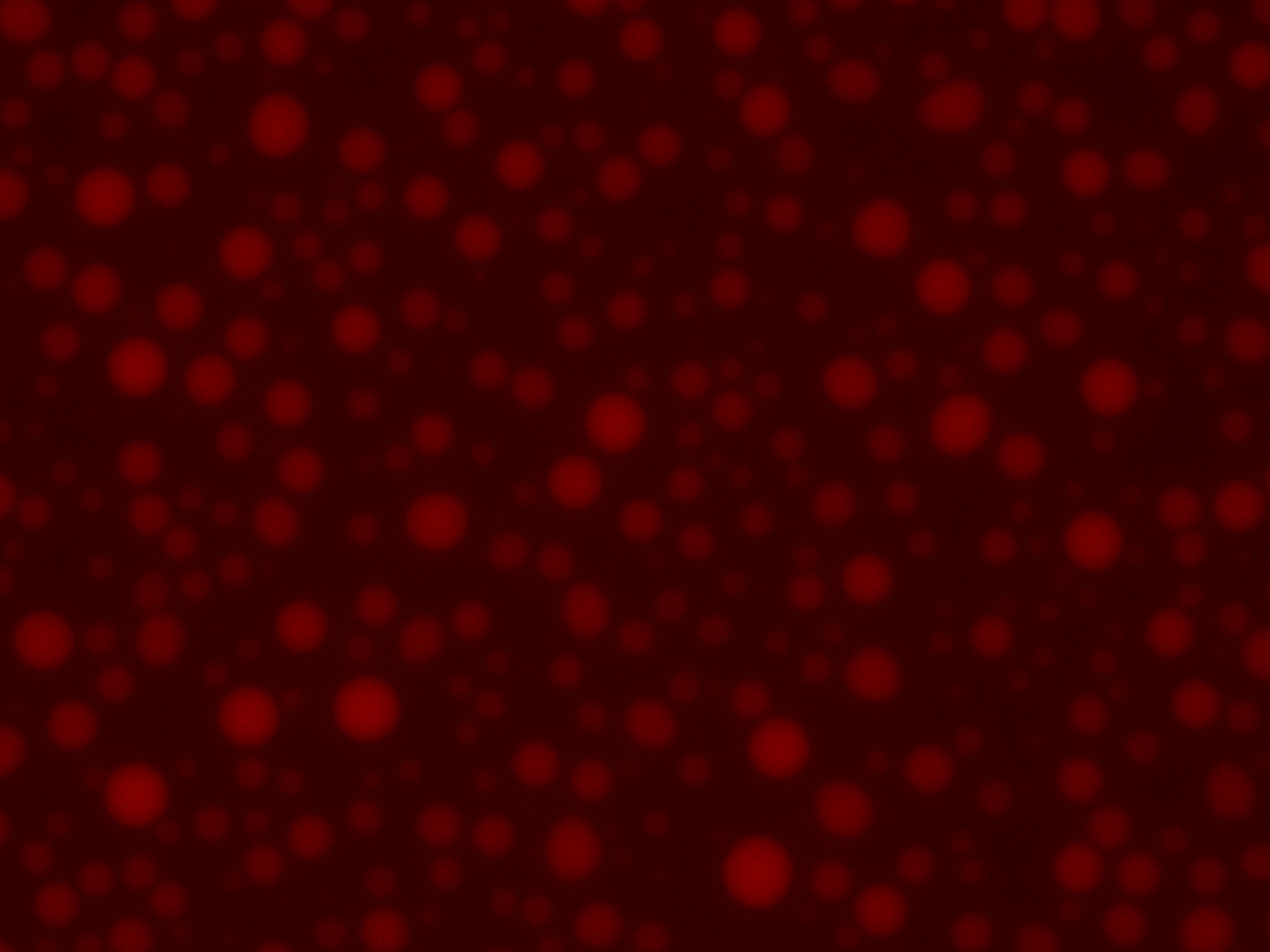

Supplement: Supplementary file 9 — EV Figures Source Data [file 44318_2025_591_MOESM9_ESM.zip › EMBOJ-2025-121908R1_SourceDataForEV/Expanded View Figure 5/EV5B/06_SO286_24h_aSyn_x80.tif]

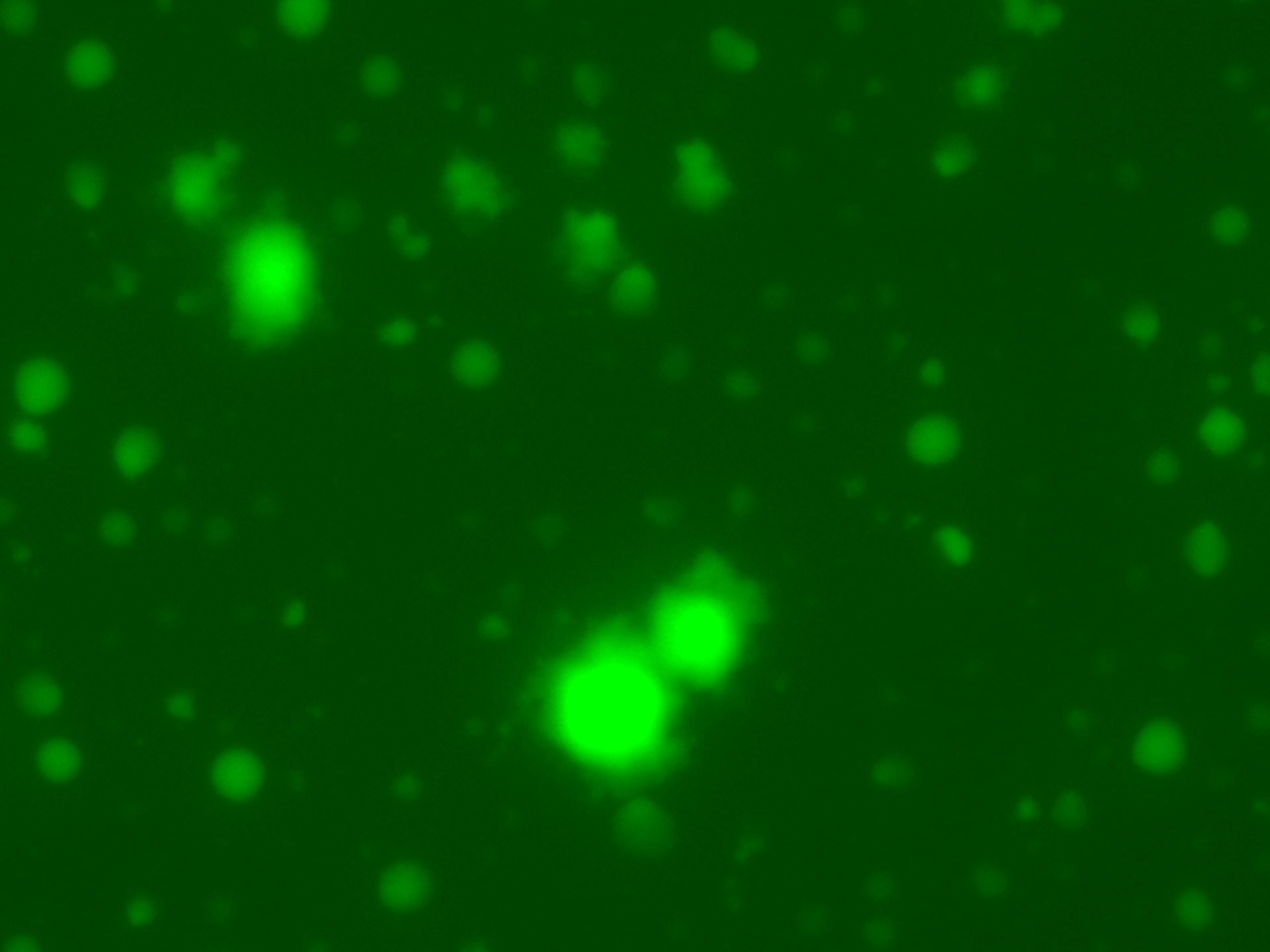

Supplement: Supplementary file 9 — EV Figures Source Data [file 44318_2025_591_MOESM9_ESM.zip › EMBOJ-2025-121908R1_SourceDataForEV/Expanded View Figure 5/EV5B/03_Control_96h_UBQLN2_x120.tif]

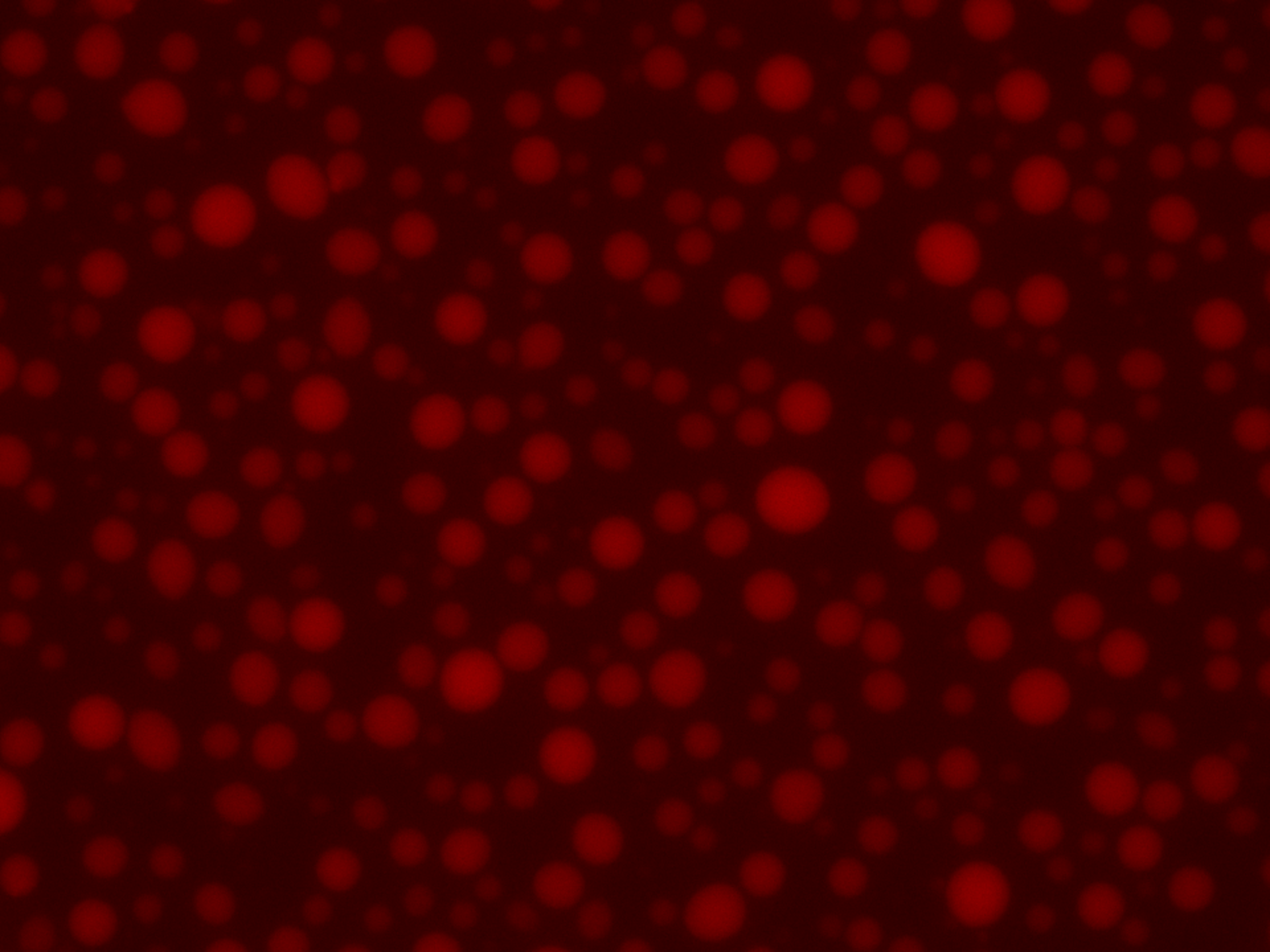

Supplement: Supplementary file 9 — EV Figures Source Data [file 44318_2025_591_MOESM9_ESM.zip › EMBOJ-2025-121908R1_SourceDataForEV/Expanded View Figure 5/EV5B/10_SO82_24h_aSyn_x80.tif]

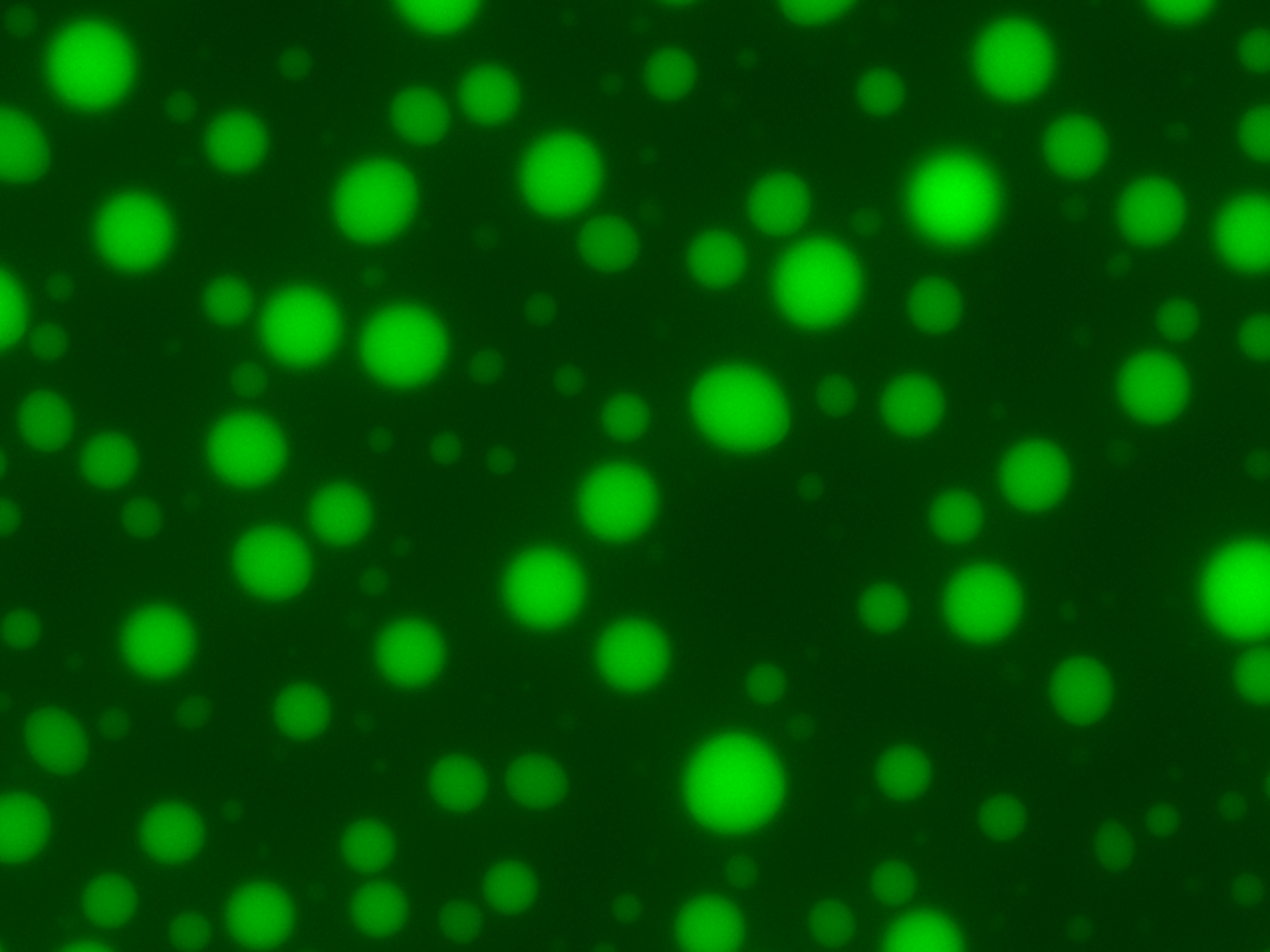

Supplement: Supplementary file 9 — EV Figures Source Data [file 44318_2025_591_MOESM9_ESM.zip › EMBOJ-2025-121908R1_SourceDataForEV/Expanded View Figure 5/EV5B/07_SO286_96h_UBQLN2_x120.tif]

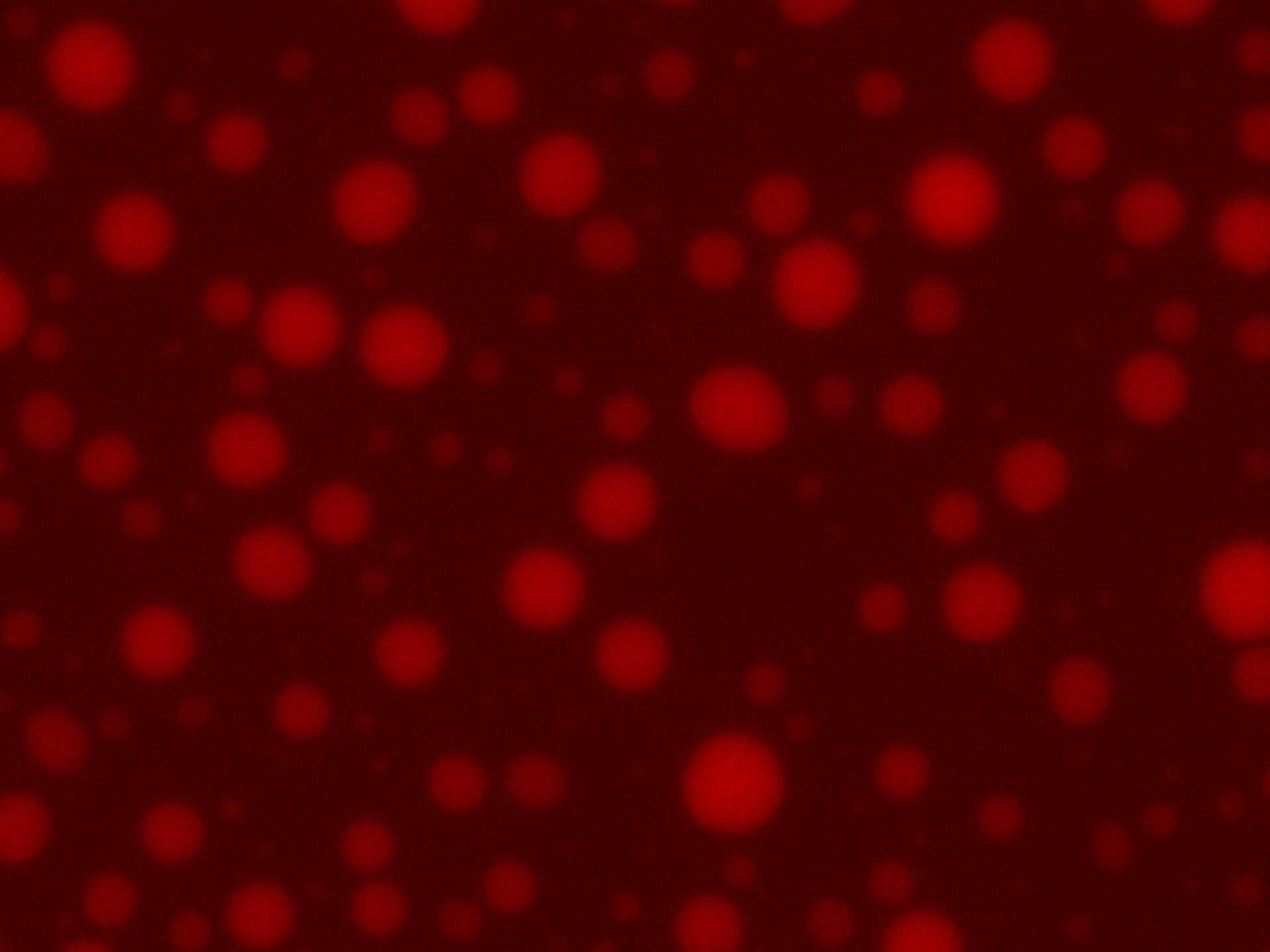

Supplement: Supplementary file 9 — EV Figures Source Data [file 44318_2025_591_MOESM9_ESM.zip › EMBOJ-2025-121908R1_SourceDataForEV/Expanded View Figure 5/EV5B/08_SO286_96h_aSyn_x120.tif]

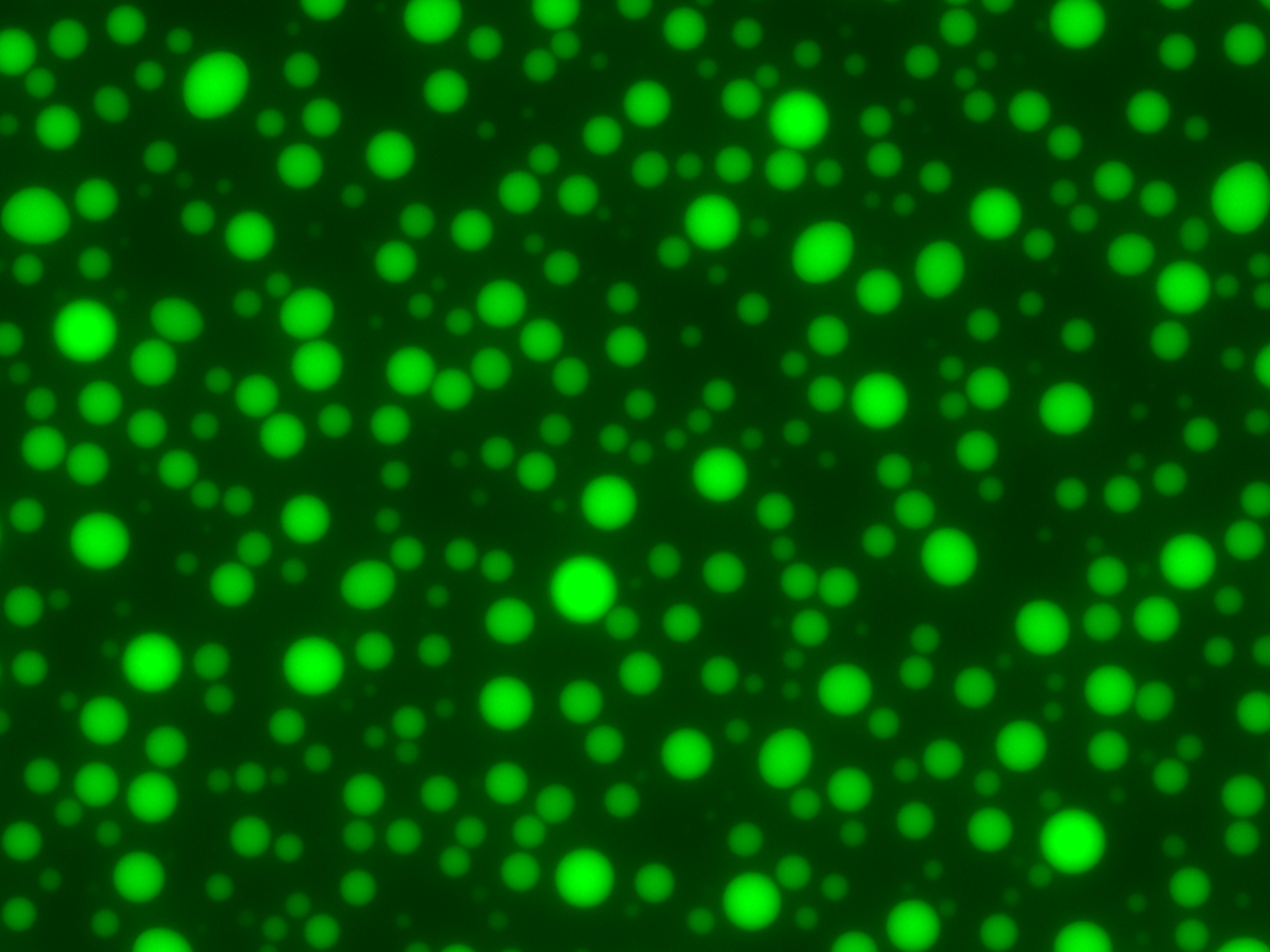

Supplement: Supplementary file 9 — EV Figures Source Data [file 44318_2025_591_MOESM9_ESM.zip › EMBOJ-2025-121908R1_SourceDataForEV/Expanded View Figure 5/EV5B/01_Control_24h_UBQLN2_x80.tif]

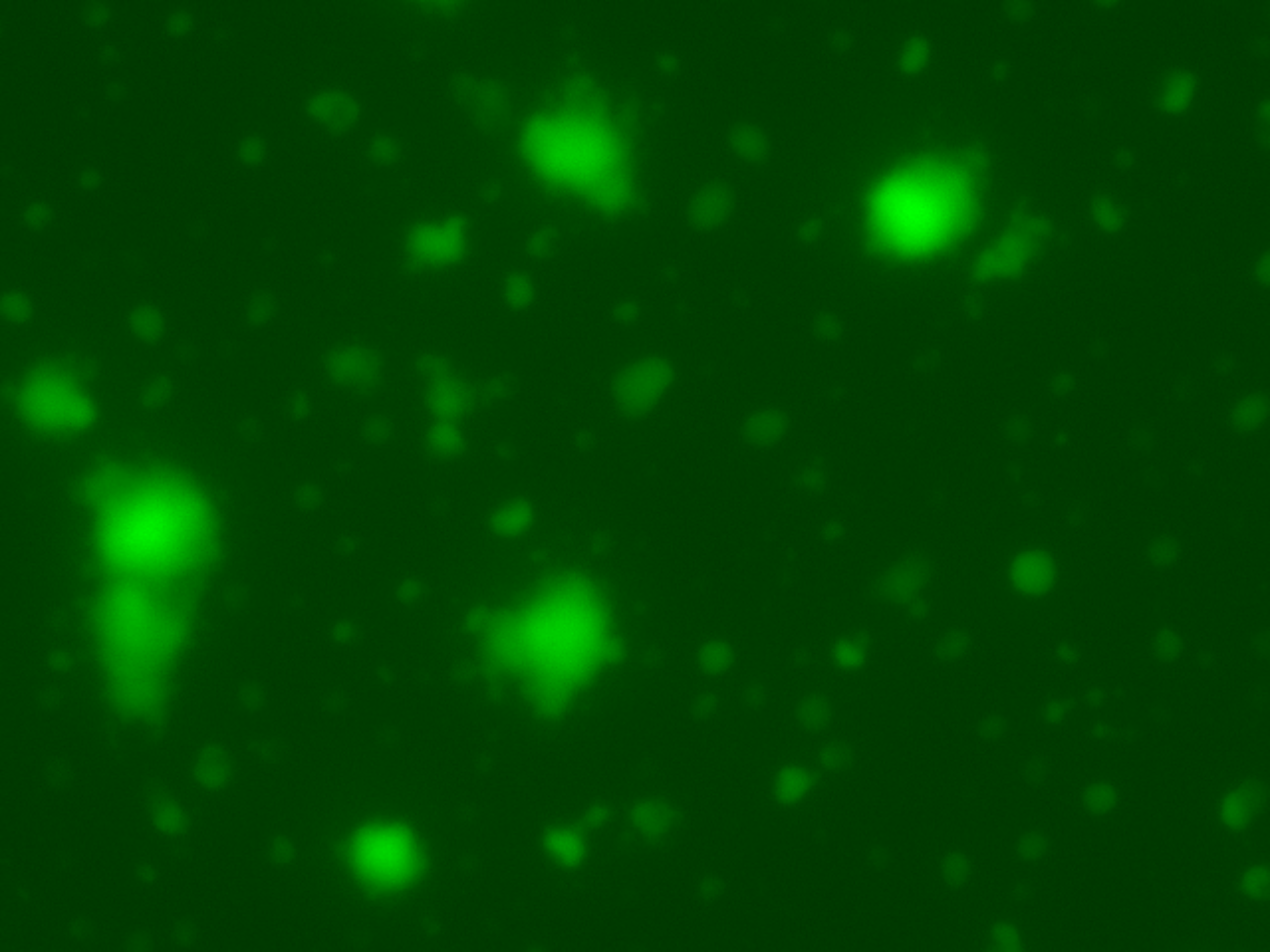

Supplement: Supplementary file 9 — EV Figures Source Data [file 44318_2025_591_MOESM9_ESM.zip › EMBOJ-2025-121908R1_SourceDataForEV/Expanded View Figure 5/EV5B/11_SO82_96h_UBQLN2_x120.tif]

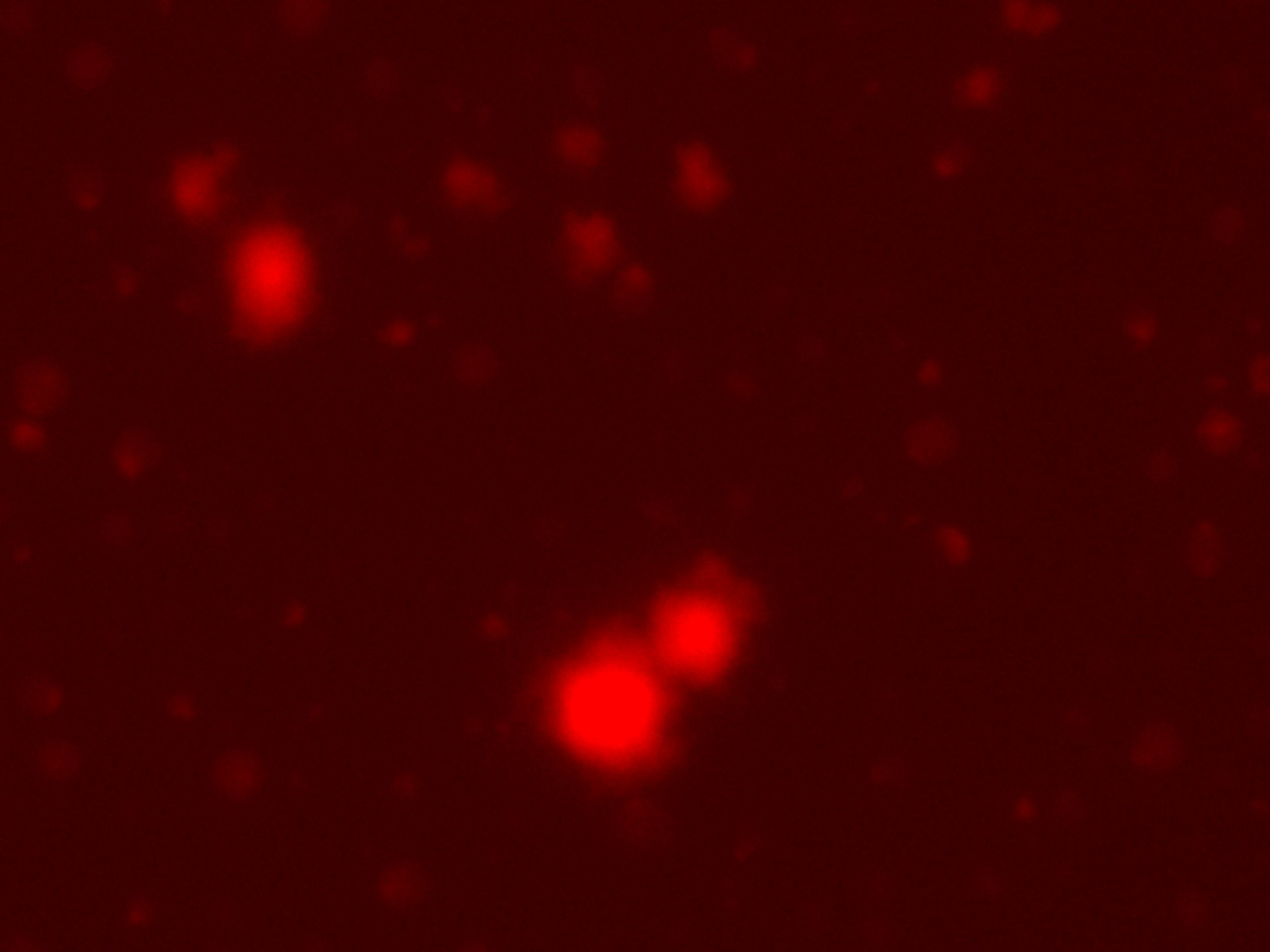

Supplement: Supplementary file 9 — EV Figures Source Data [file 44318_2025_591_MOESM9_ESM.zip › EMBOJ-2025-121908R1_SourceDataForEV/Expanded View Figure 5/EV5B/04_Control_96h_aSyn_x120.tif]

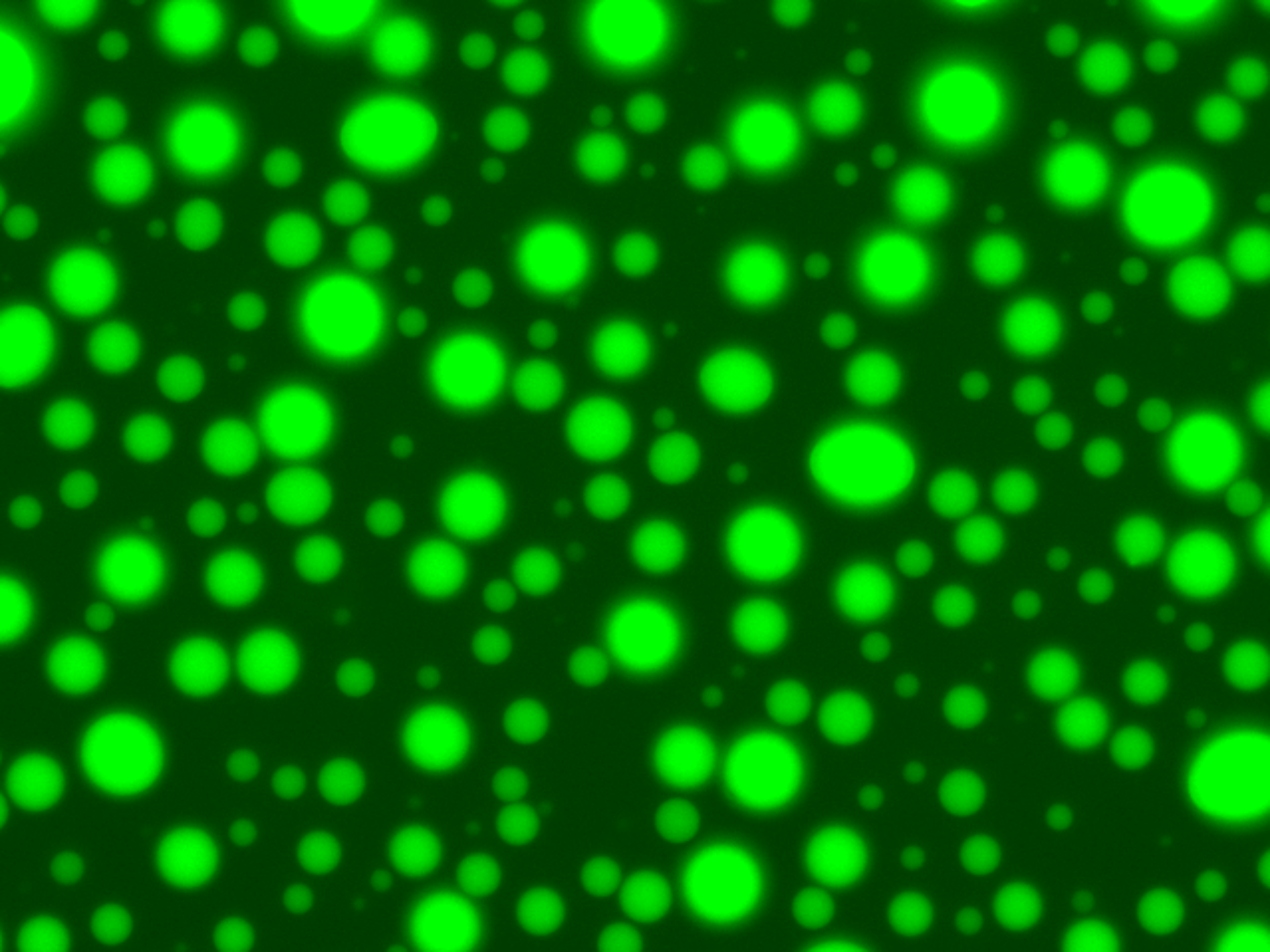

Supplement: Supplementary file 9 — EV Figures Source Data [file 44318_2025_591_MOESM9_ESM.zip › EMBOJ-2025-121908R1_SourceDataForEV/Expanded View Figure 4/EV4B/(a)_01_UBQLN2_SO286(0 ╬╝M).tif]

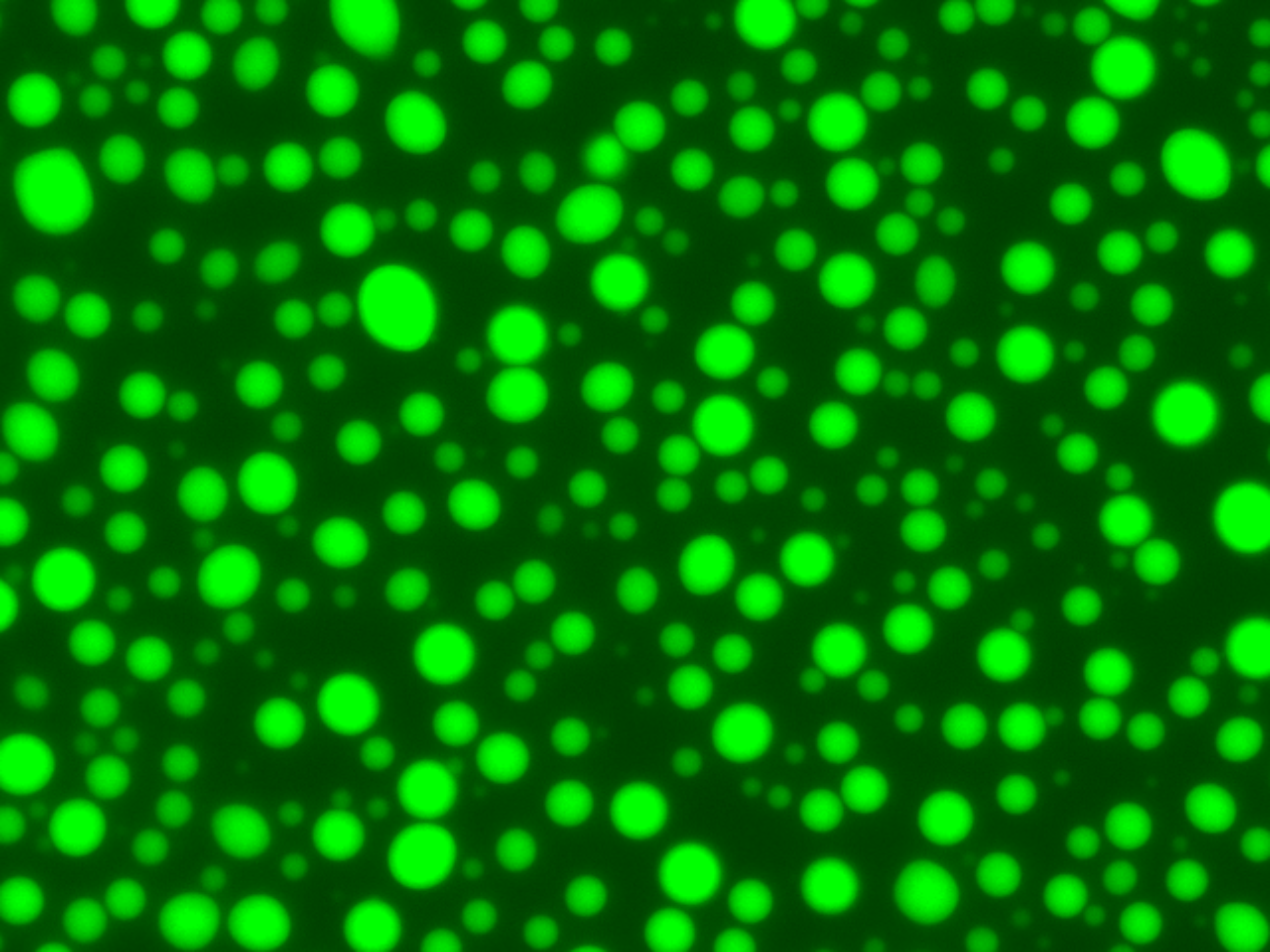

Supplement: Supplementary file 9 — EV Figures Source Data [file 44318_2025_591_MOESM9_ESM.zip › EMBOJ-2025-121908R1_SourceDataForEV/Expanded View Figure 4/EV4B/(a)_09_UBQLN4_SO286(0 ╬╝M).tif]

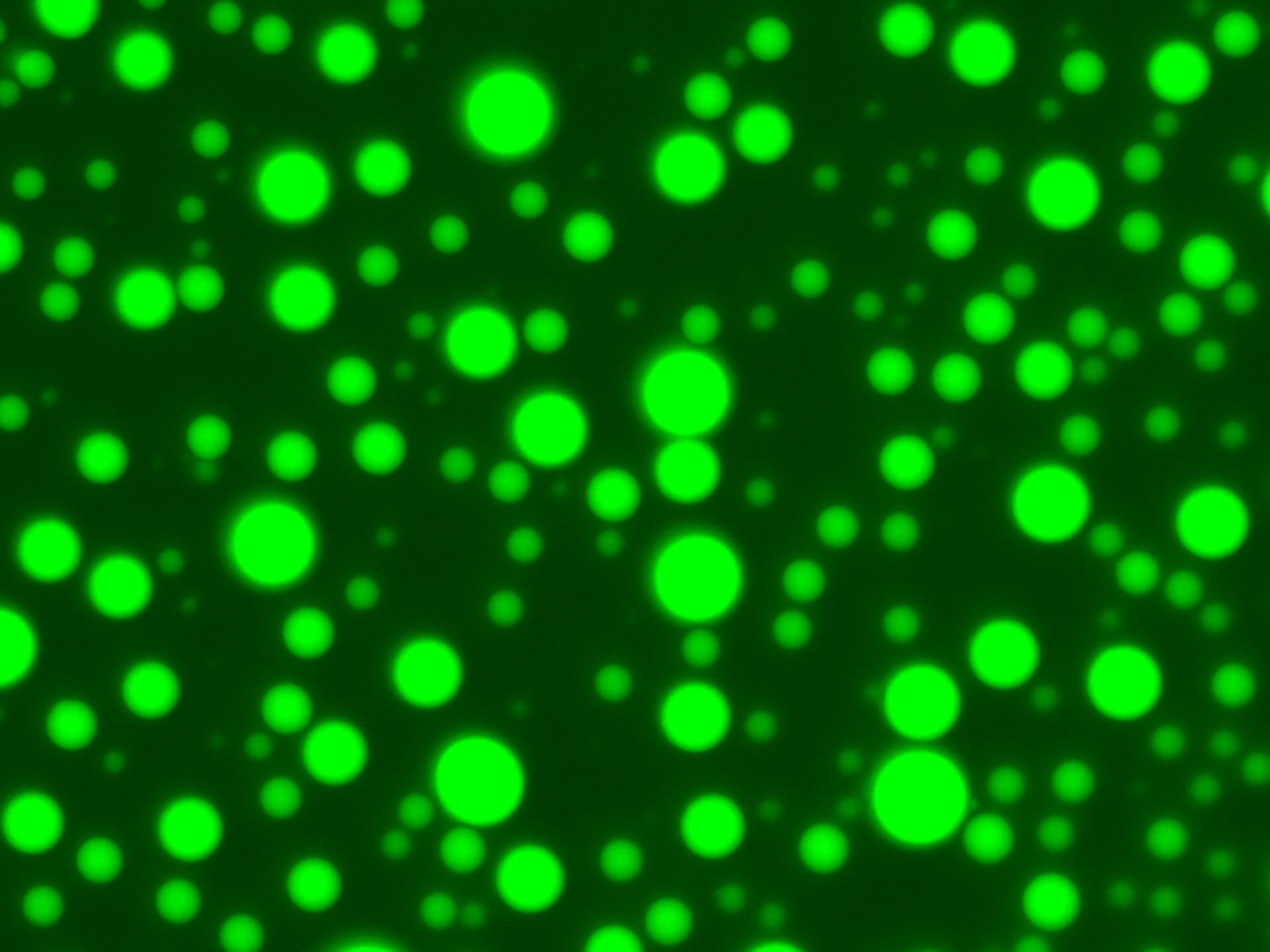

Supplement: Supplementary file 9 — EV Figures Source Data [file 44318_2025_591_MOESM9_ESM.zip › EMBOJ-2025-121908R1_SourceDataForEV/Expanded View Figure 4/EV4B/(a)_04_UBQLN2_SO286(20 ╬╝M).tif]

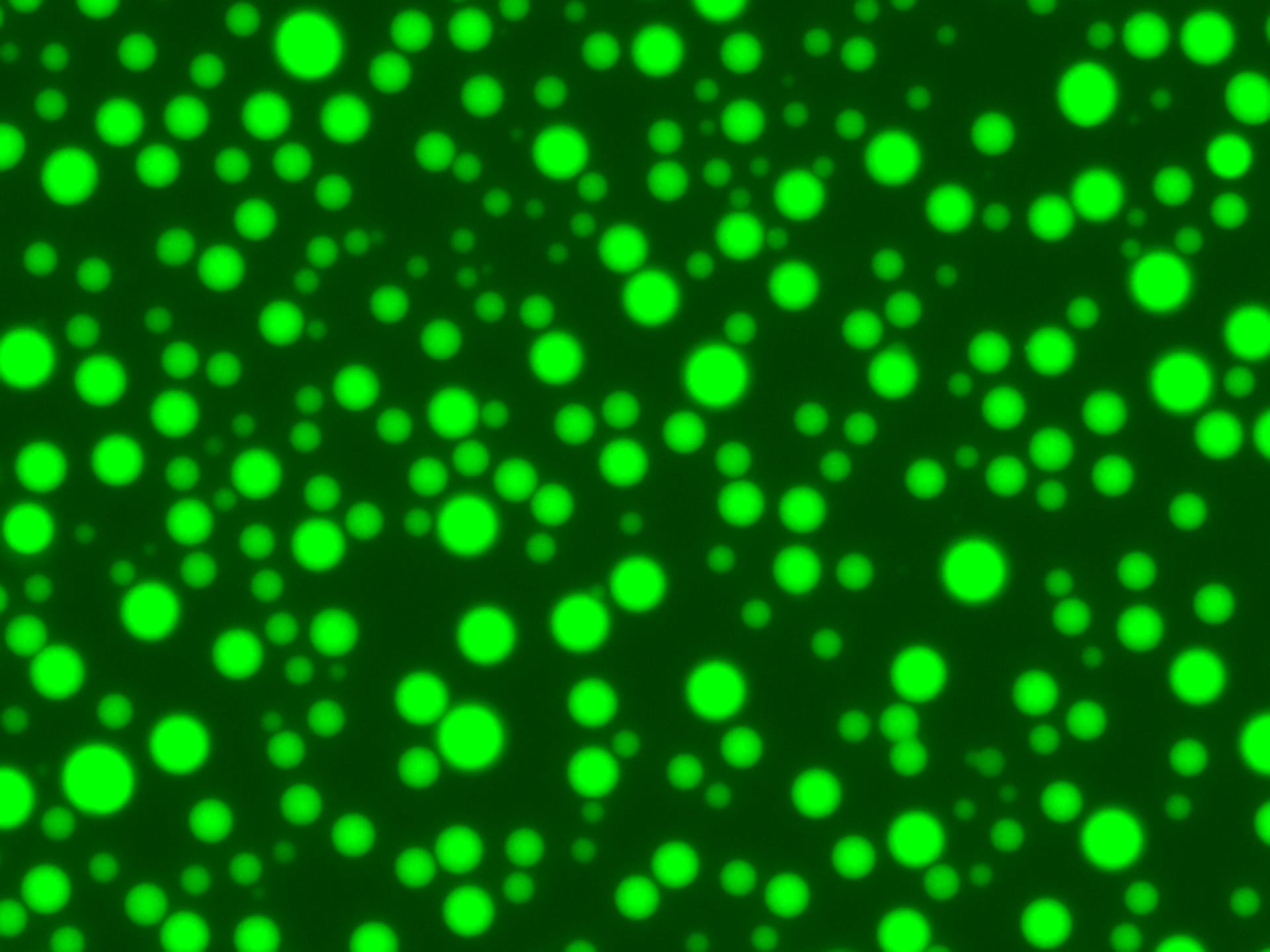

Supplement: Supplementary file 9 — EV Figures Source Data [file 44318_2025_591_MOESM9_ESM.zip › EMBOJ-2025-121908R1_SourceDataForEV/Expanded View Figure 4/EV4B/(a)_11_UBQLN4_SO286(7 ╬╝M).tif]

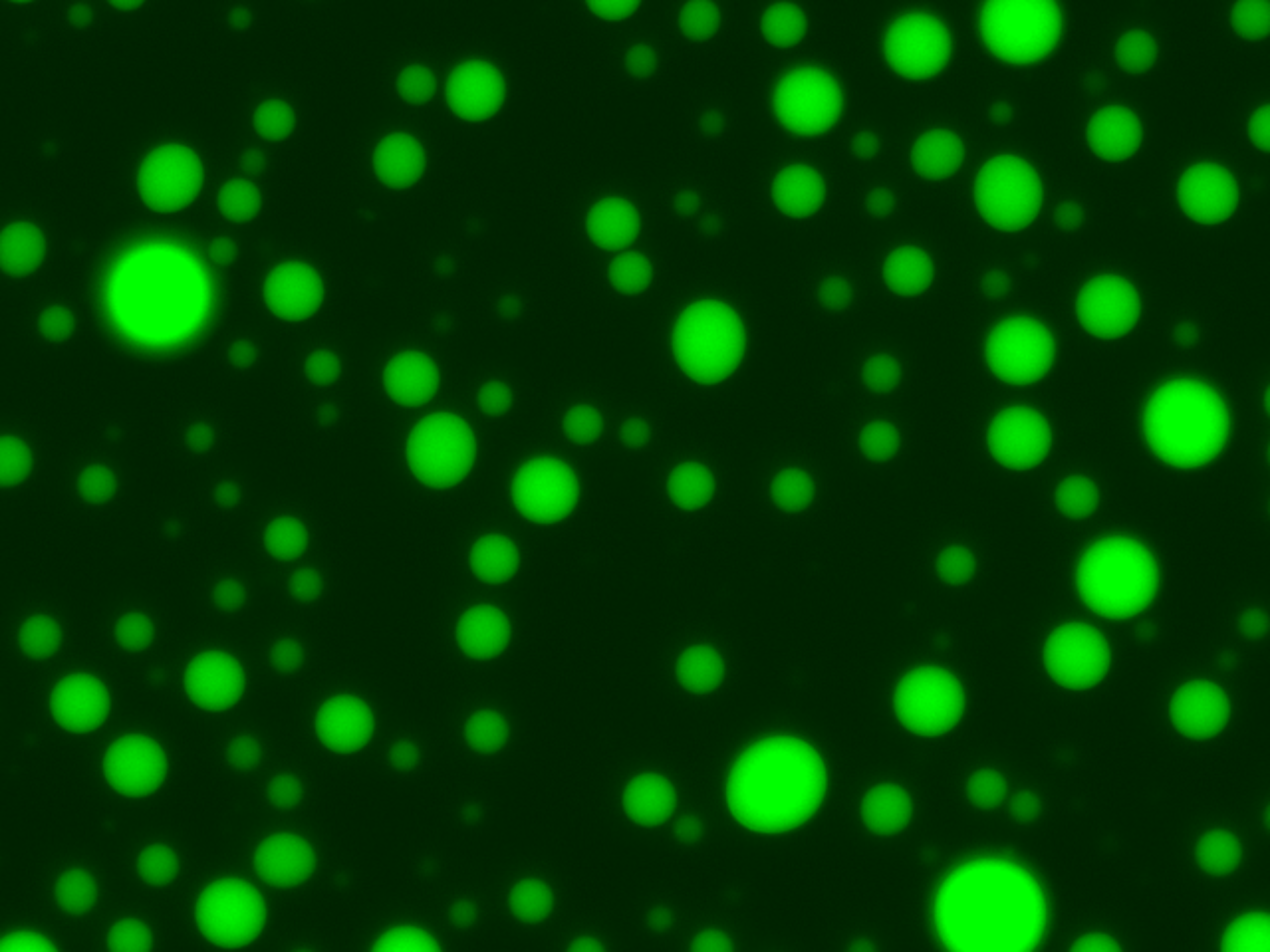

Supplement: Supplementary file 9 — EV Figures Source Data [file 44318_2025_591_MOESM9_ESM.zip › EMBOJ-2025-121908R1_SourceDataForEV/Expanded View Figure 4/EV4B/(a)_07_UBQLN1_SO286(7 ╬╝M).tif]

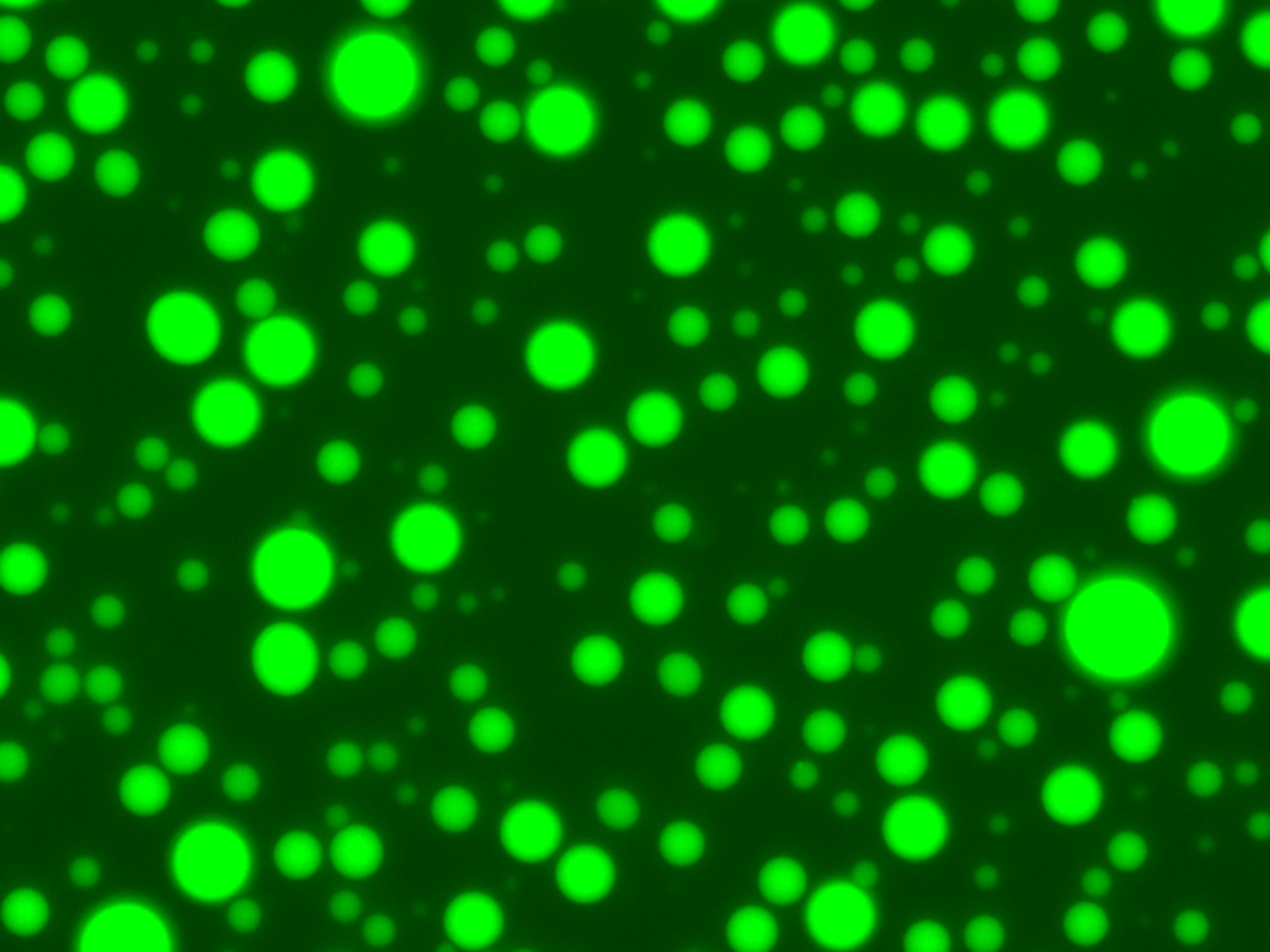

Supplement: Supplementary file 9 — EV Figures Source Data [file 44318_2025_591_MOESM9_ESM.zip › EMBOJ-2025-121908R1_SourceDataForEV/Expanded View Figure 4/EV4B/(a)_02_UBQLN2_SO286(2 ╬╝M).tif]

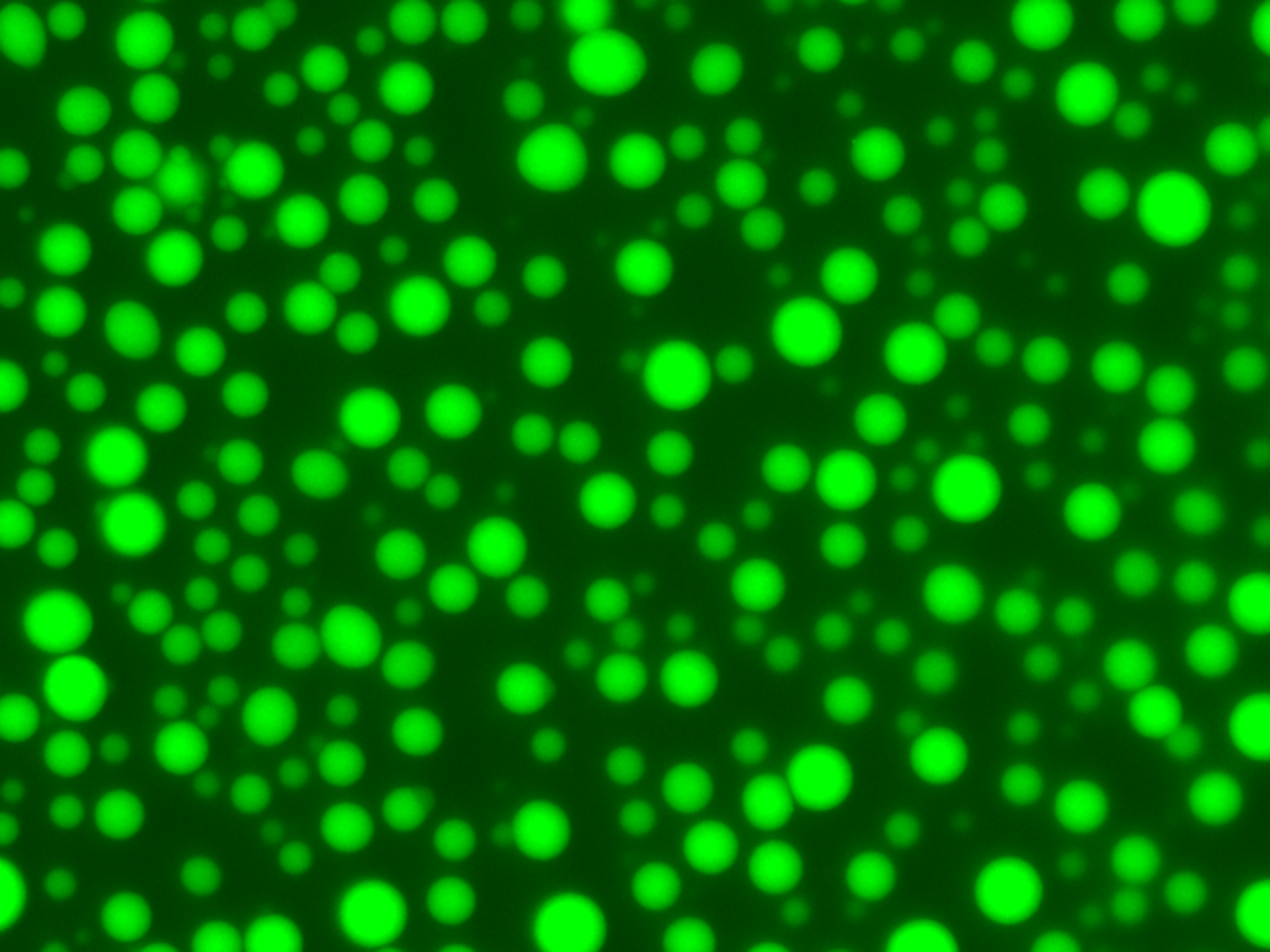

Supplement: Supplementary file 9 — EV Figures Source Data [file 44318_2025_591_MOESM9_ESM.zip › EMBOJ-2025-121908R1_SourceDataForEV/Expanded View Figure 4/EV4B/(a)_12_UBQLN4_SO286(20 ╬╝M).tif]

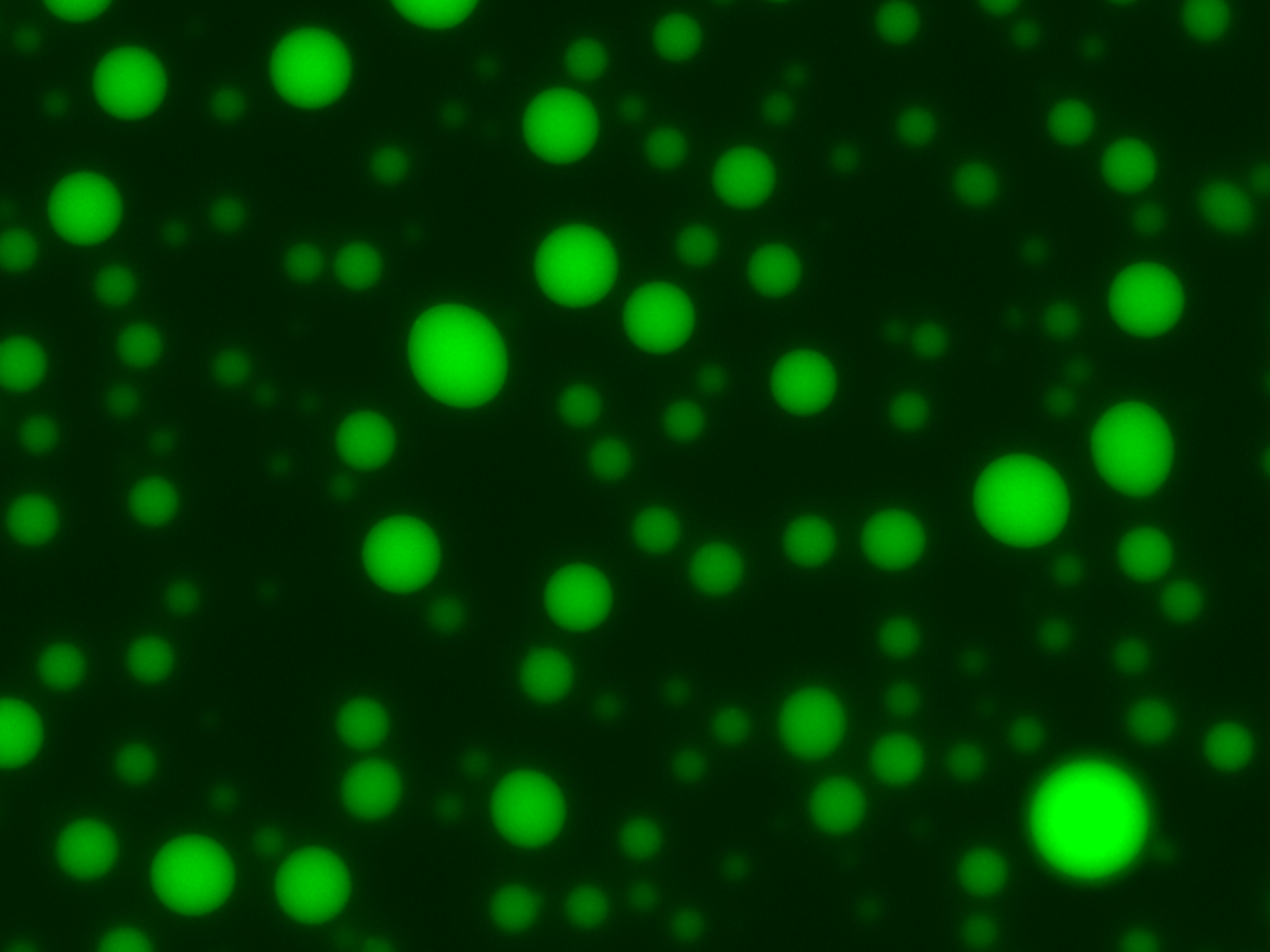

Supplement: Supplementary file 9 — EV Figures Source Data [file 44318_2025_591_MOESM9_ESM.zip › EMBOJ-2025-121908R1_SourceDataForEV/Expanded View Figure 4/EV4B/(a)_05_UBQLN1_SO286(0 ╬╝M).tif]

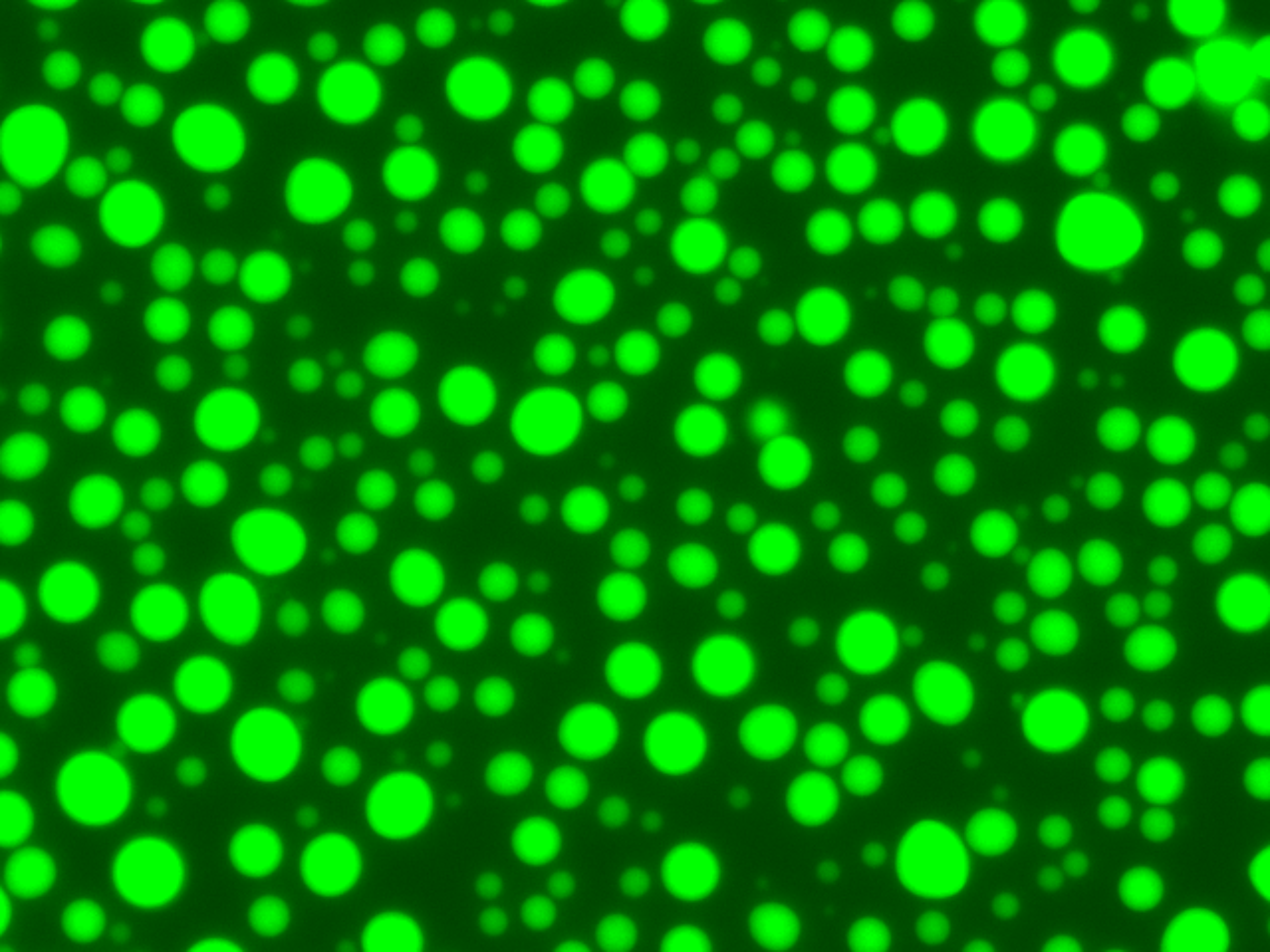

Supplement: Supplementary file 9 — EV Figures Source Data [file 44318_2025_591_MOESM9_ESM.zip › EMBOJ-2025-121908R1_SourceDataForEV/Expanded View Figure 4/EV4B/(a)_10_UBQLN4_SO286(2 ╬╝M).tif]

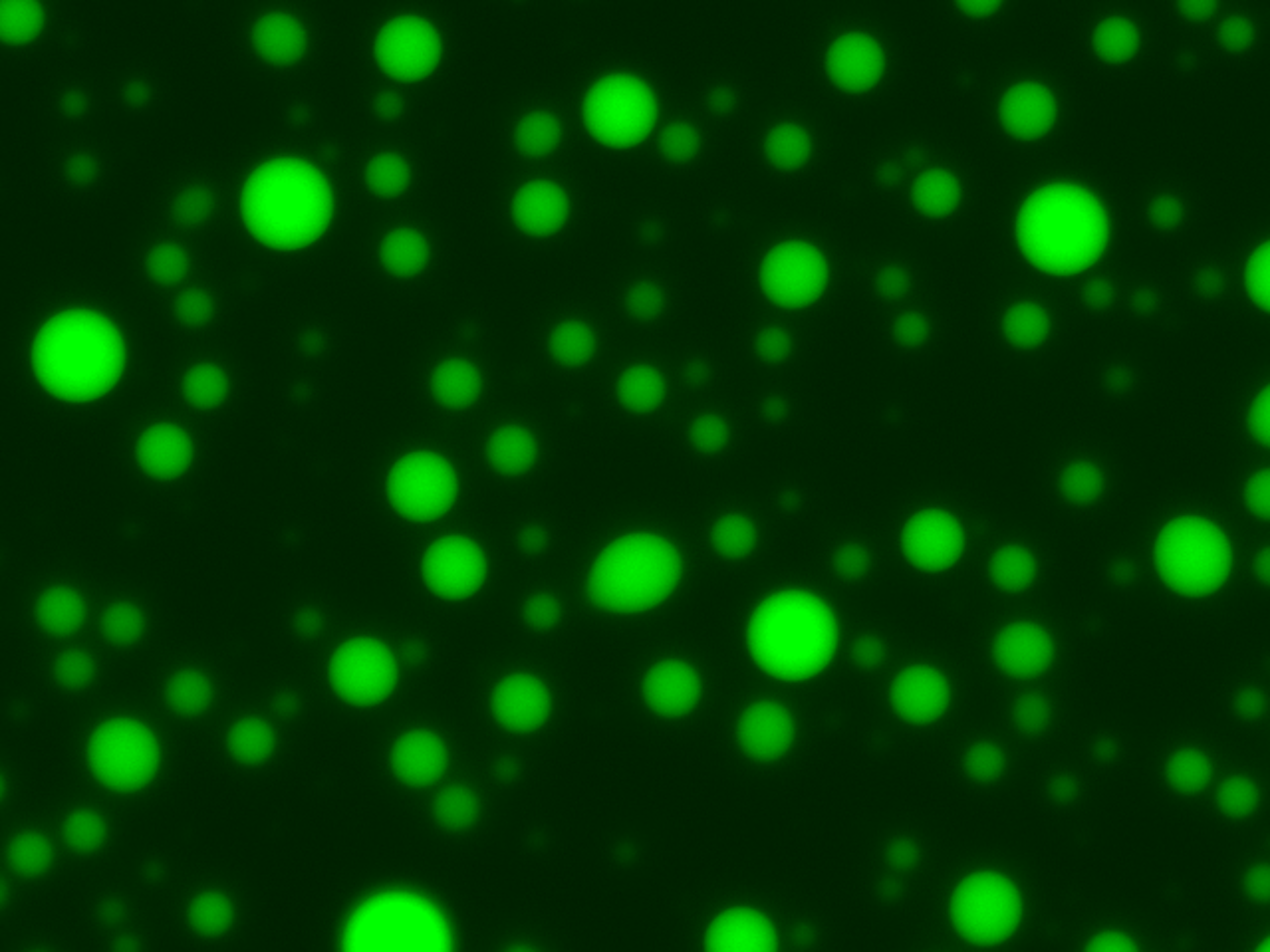

Supplement: Supplementary file 9 — EV Figures Source Data [file 44318_2025_591_MOESM9_ESM.zip › EMBOJ-2025-121908R1_SourceDataForEV/Expanded View Figure 4/EV4B/(a)_06_UBQLN1_SO286(2 ╬╝M).tif]

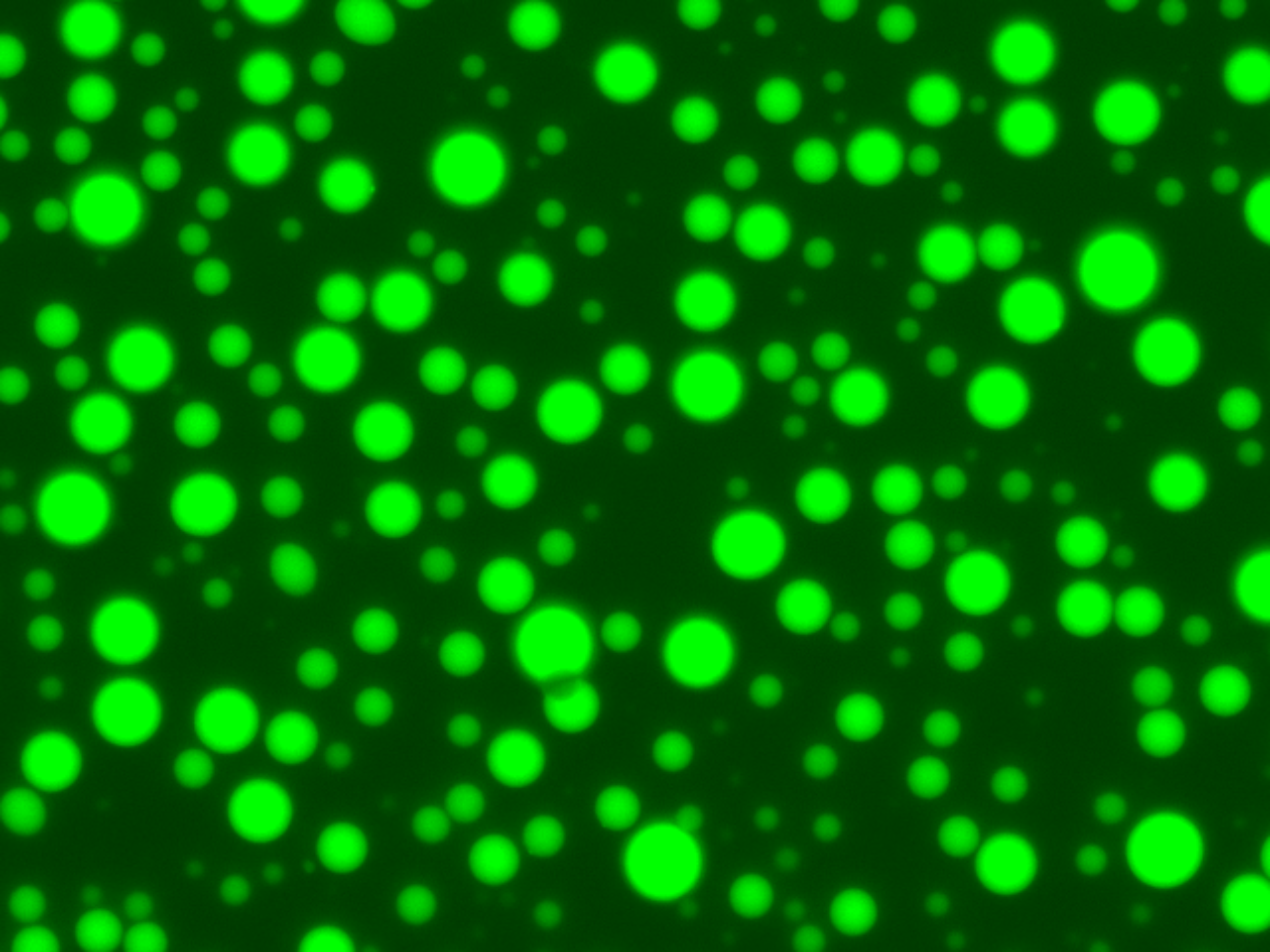

Supplement: Supplementary file 9 — EV Figures Source Data [file 44318_2025_591_MOESM9_ESM.zip › EMBOJ-2025-121908R1_SourceDataForEV/Expanded View Figure 4/EV4B/(a)_03_UBQLN2_SO286(7 ╬╝M).tif]

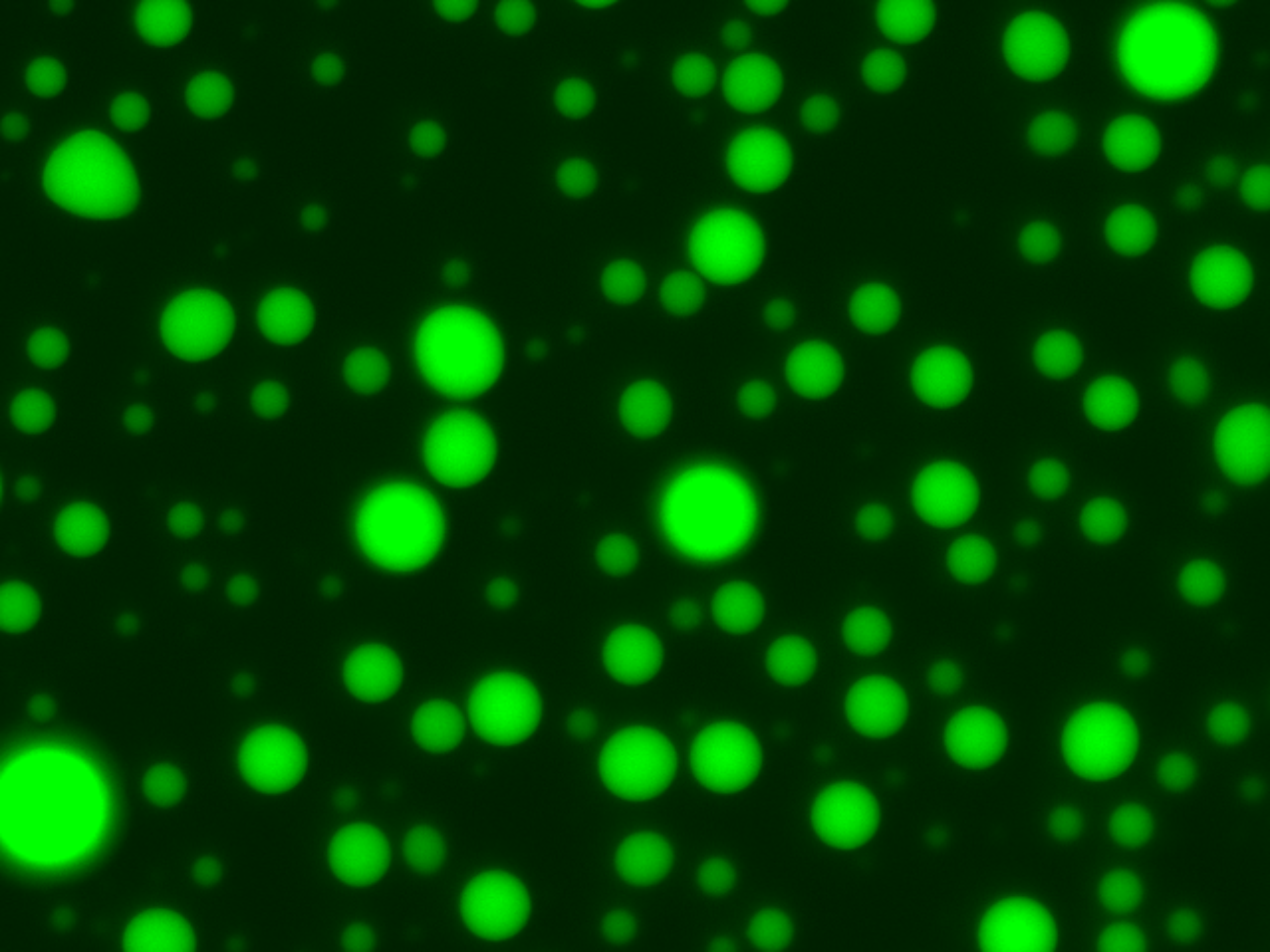

Supplement: Supplementary file 9 — EV Figures Source Data [file 44318_2025_591_MOESM9_ESM.zip › EMBOJ-2025-121908R1_SourceDataForEV/Expanded View Figure 4/EV4B/(a)_08_UBQLN1_SO286(20 ╬╝M).tif]

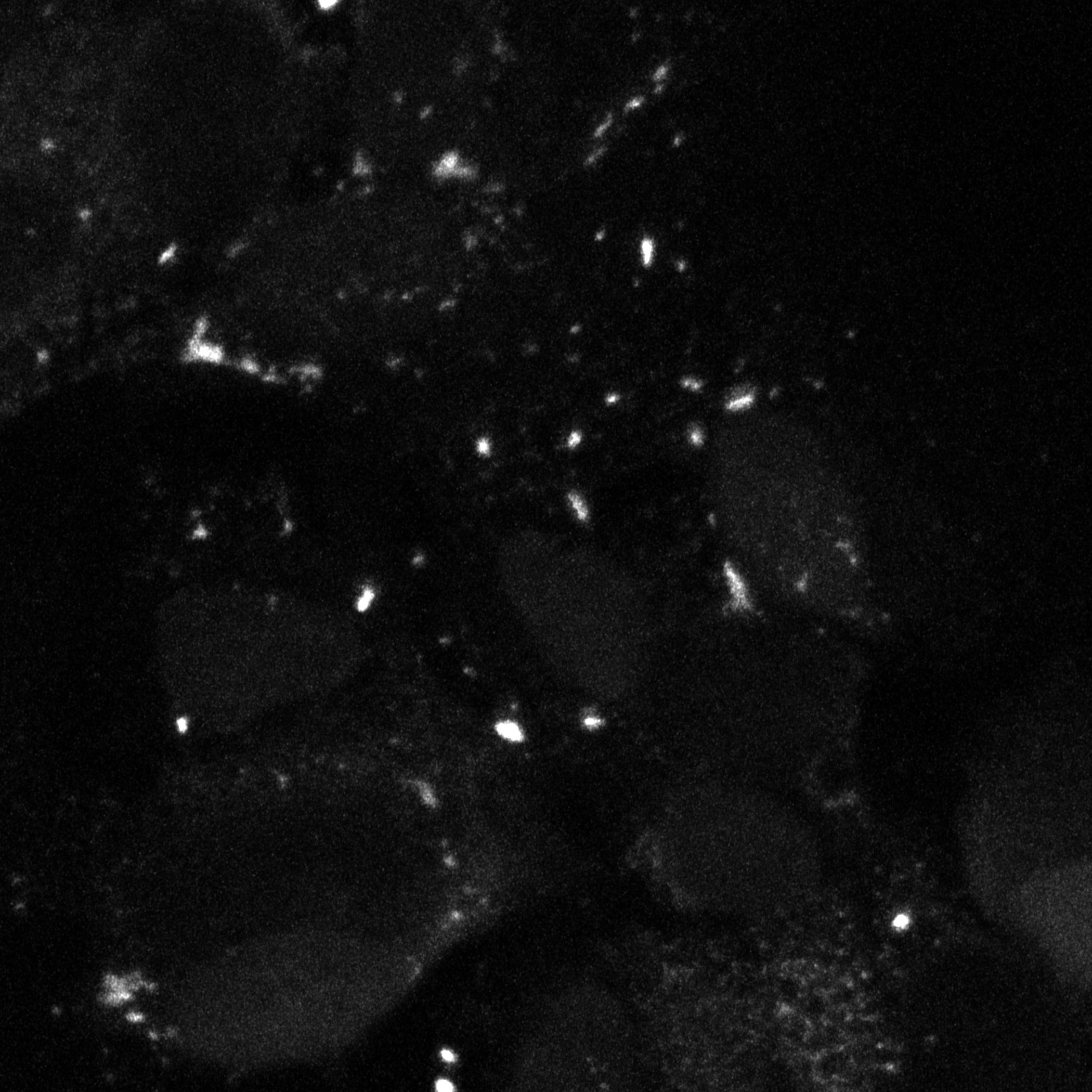

Supplement: Supplementary file 10 — Appendix Fig S1, S2, S3 Source Data [file 44318_2025_591_MOESM10_ESM.zip › Appendix Figure S3/S3C/02_Control KO_As 2 h_G3BP_╬▒-Syn-EGFP_eIF4G1.tif]

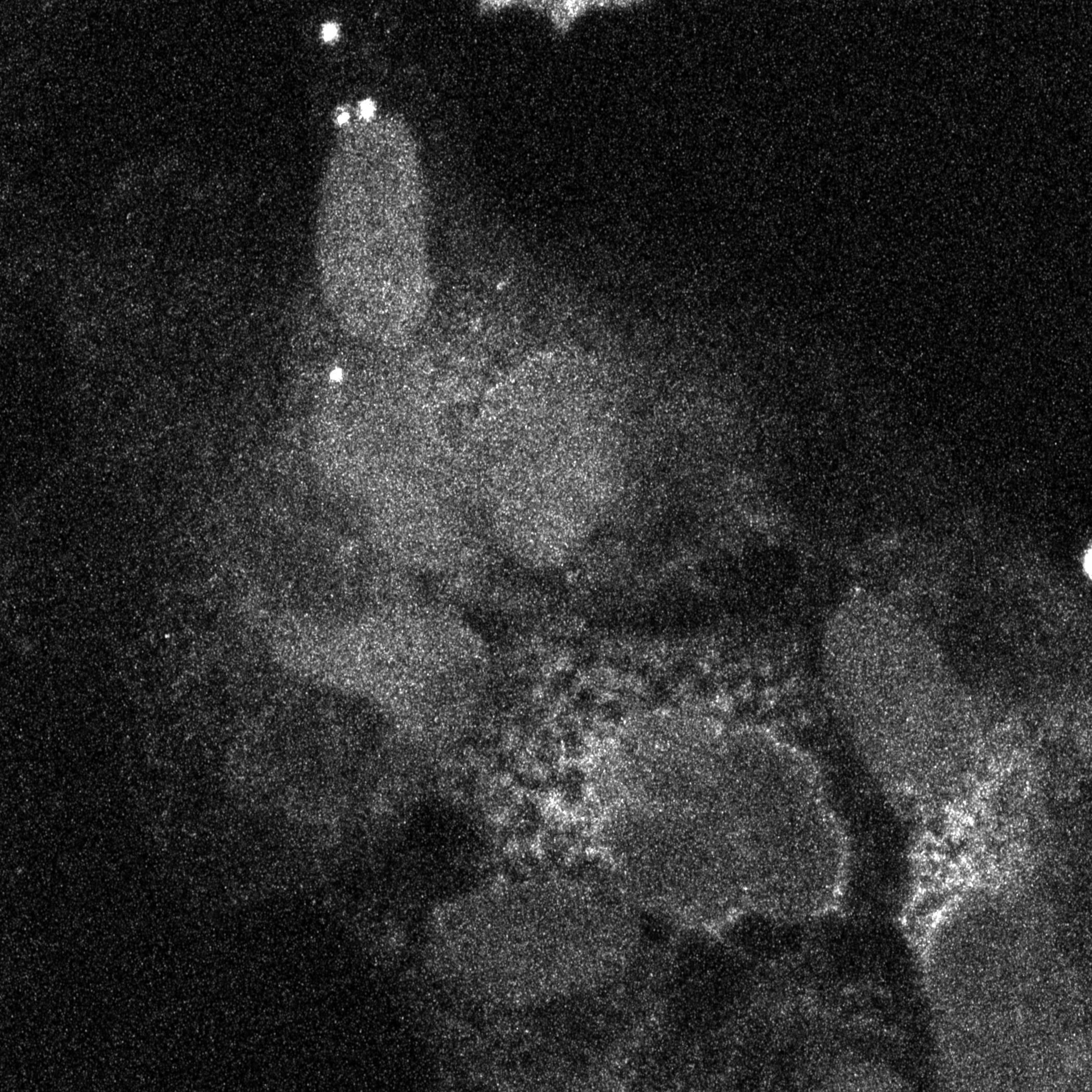

Supplement: Supplementary file 10 — Appendix Fig S1, S2, S3 Source Data [file 44318_2025_591_MOESM10_ESM.zip › Appendix Figure S3/S3C/03_Control KO_As 6 h_G3BP_╬▒-Syn-EGFP_eIF4G1.tif]

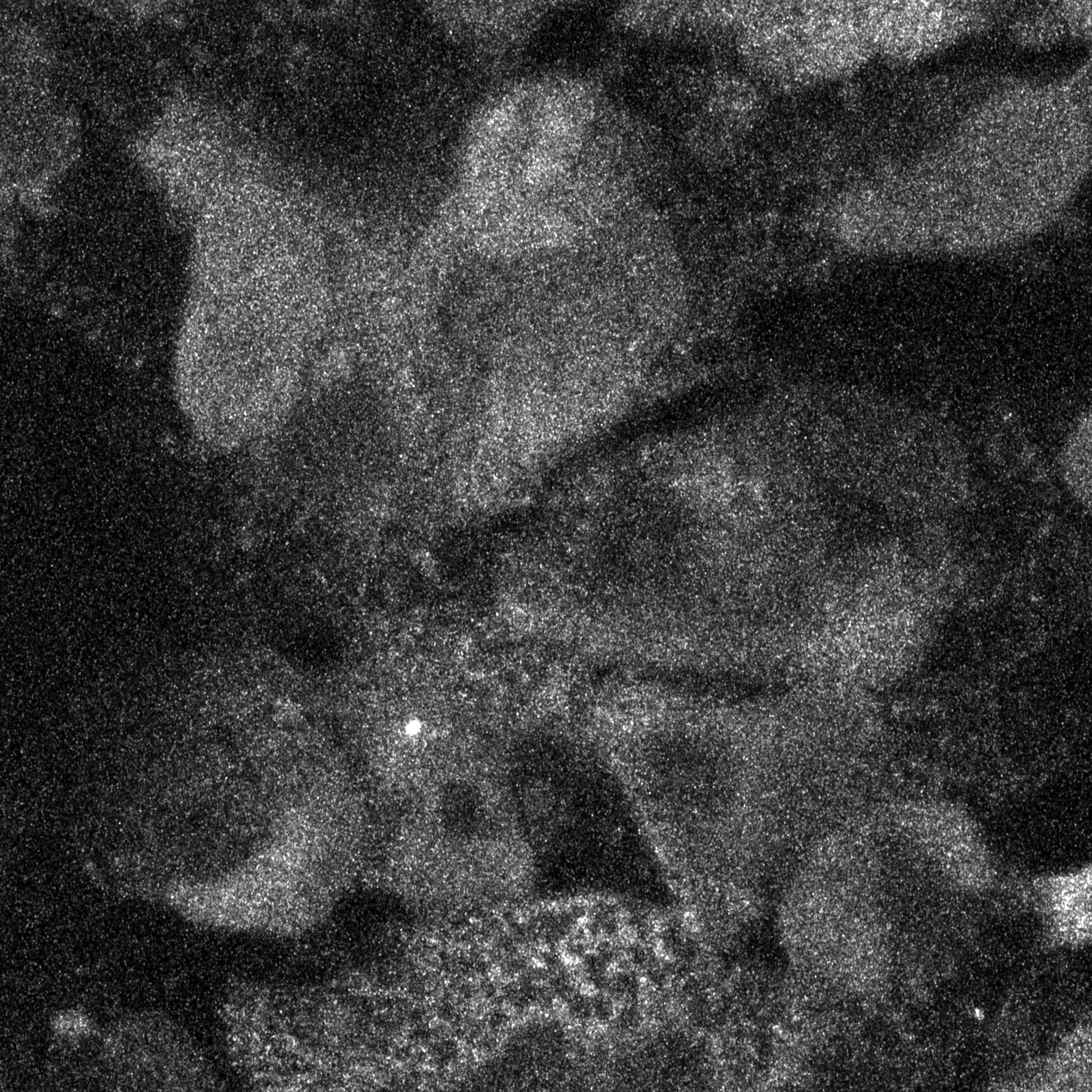

Supplement: Supplementary file 10 — Appendix Fig S1, S2, S3 Source Data [file 44318_2025_591_MOESM10_ESM.zip › Appendix Figure S3/S3C/04_Control KO_As 12 h_G3BP_╬▒-Syn-EGFP_eIF4G1.tif]

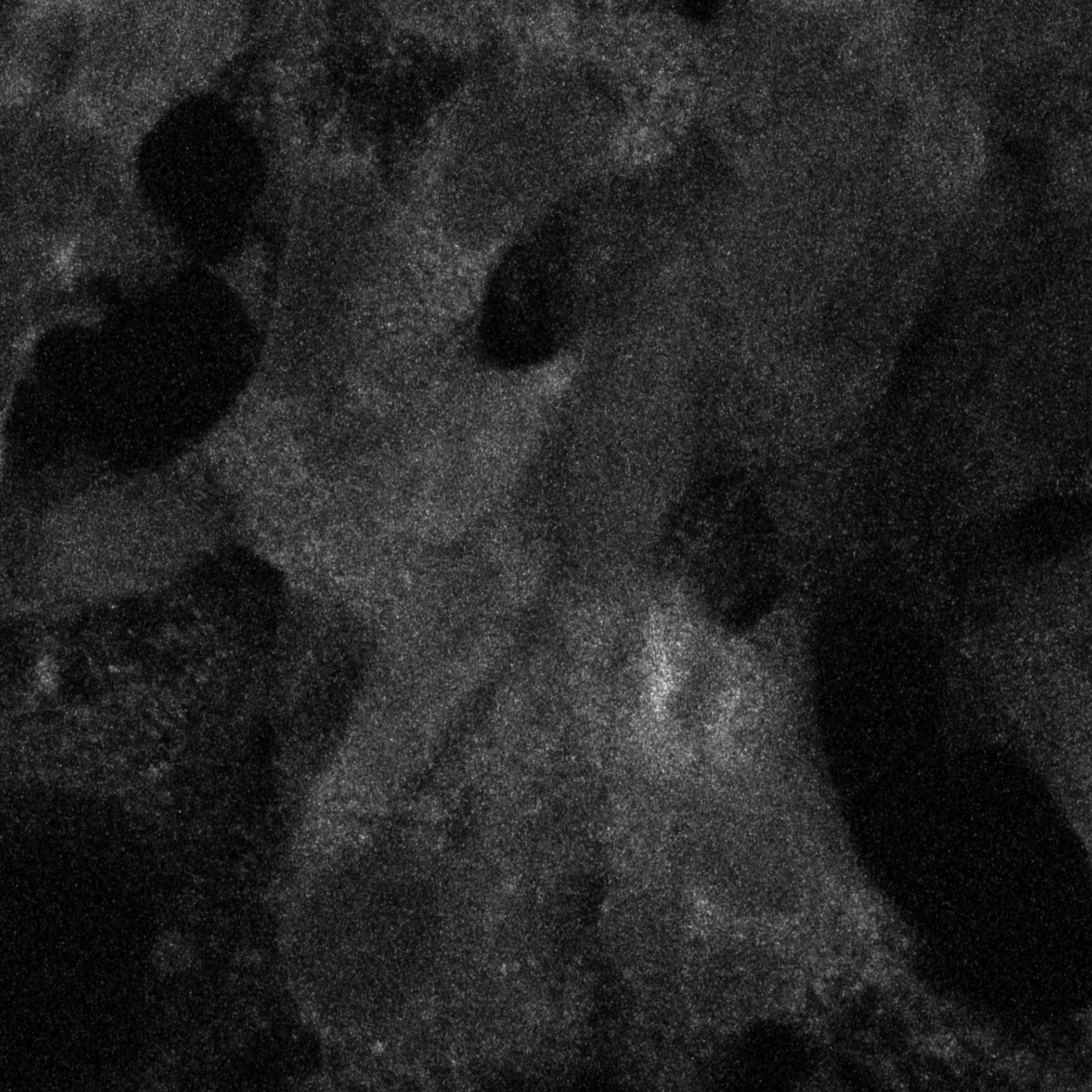

Supplement: Supplementary file 10 — Appendix Fig S1, S2, S3 Source Data [file 44318_2025_591_MOESM10_ESM.zip › Appendix Figure S3/S3C/05_UBQLN2 KO_None_G3BP_╬▒-Syn-EGFP_eIF4G1.tif]

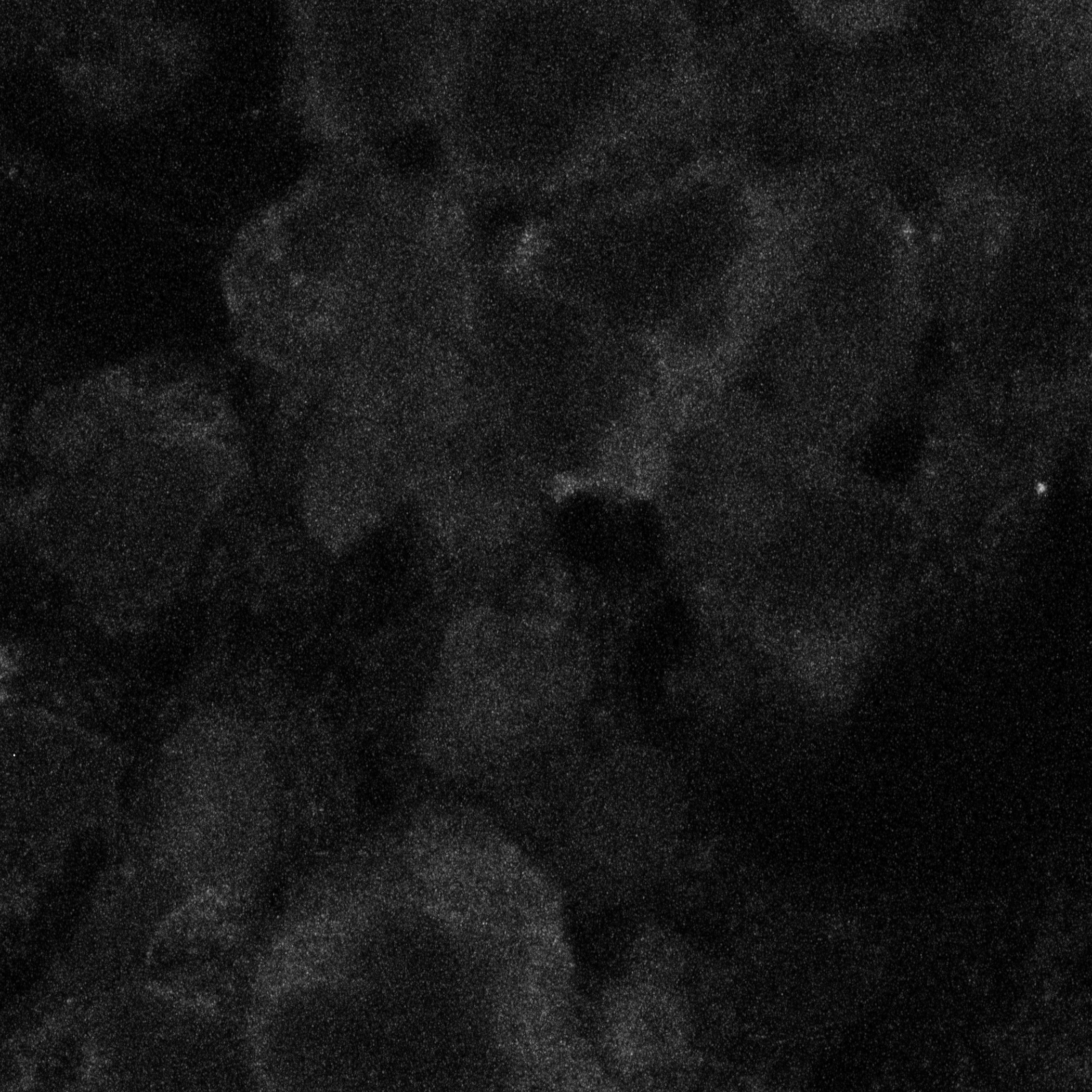

Supplement: Supplementary file 10 — Appendix Fig S1, S2, S3 Source Data [file 44318_2025_591_MOESM10_ESM.zip › Appendix Figure S3/S3C/08_UBQLN2 KO_As 12 h_G3BP_╬▒-Syn-EGFP_eIF4G1.tif]

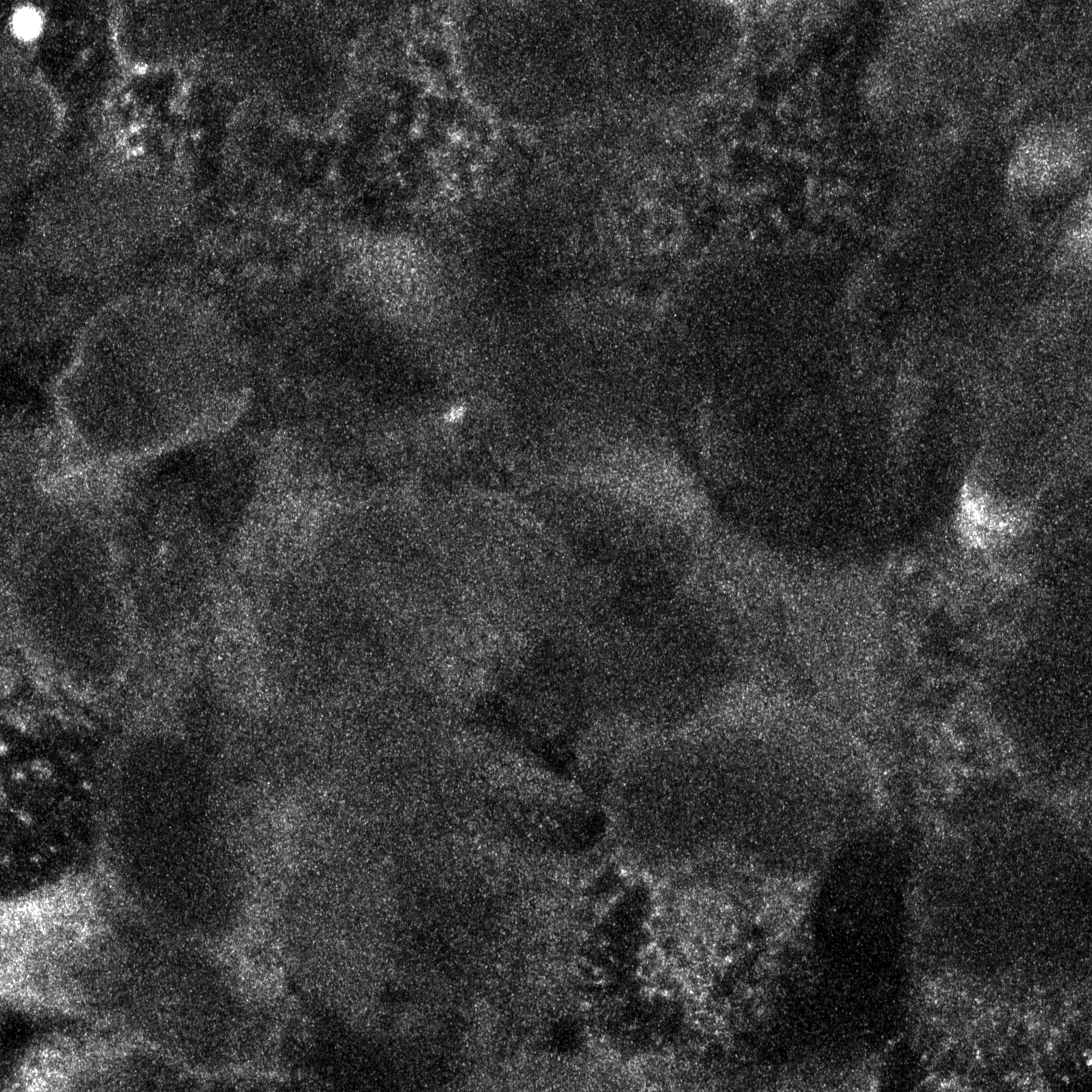

Supplement: Supplementary file 10 — Appendix Fig S1, S2, S3 Source Data [file 44318_2025_591_MOESM10_ESM.zip › Appendix Figure S3/S3C/07_UBQLN2 KO_As 6 h_G3BP_╬▒-Syn-EGFP_eIF4G1.tif]

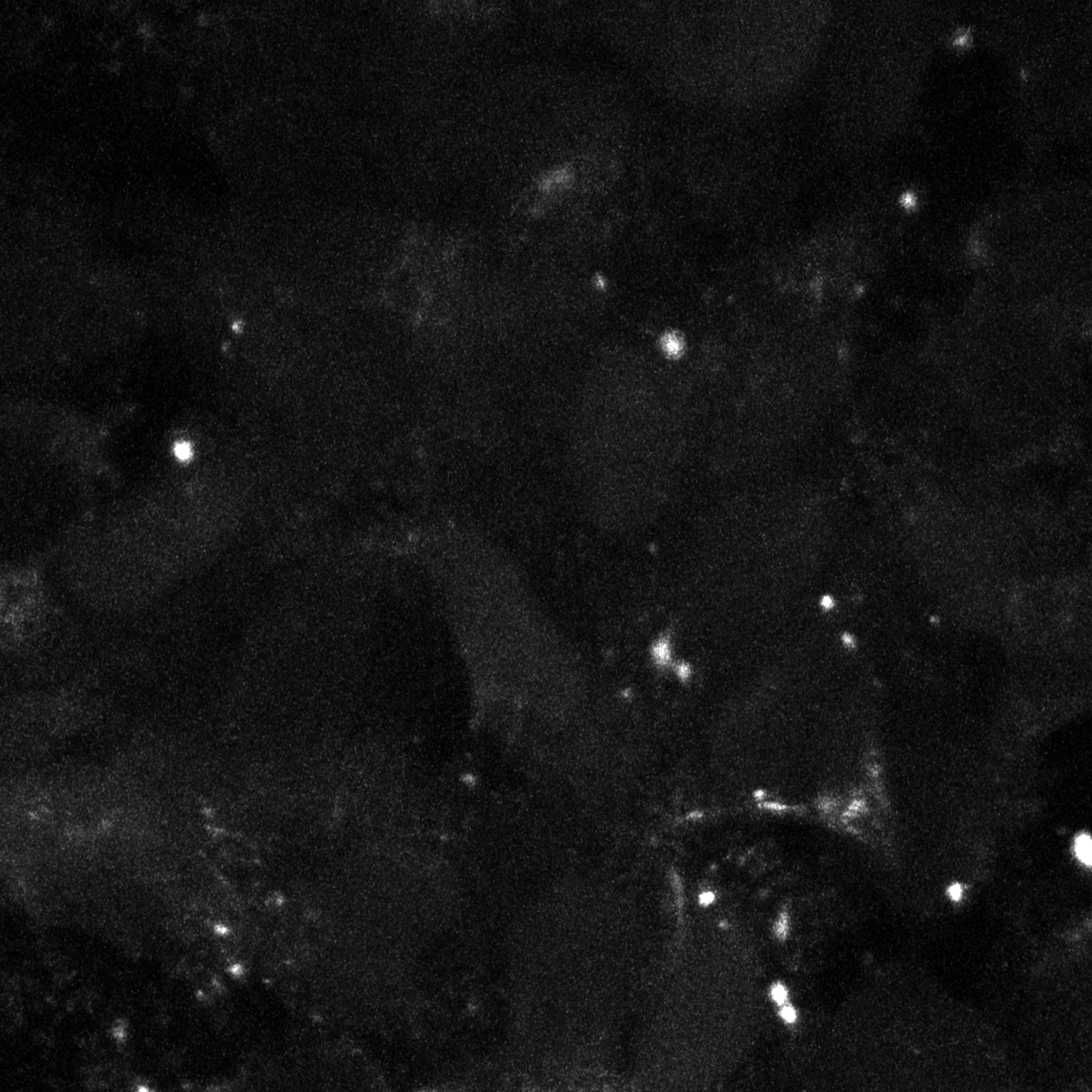

Supplement: Supplementary file 10 — Appendix Fig S1, S2, S3 Source Data [file 44318_2025_591_MOESM10_ESM.zip › Appendix Figure S3/S3C/06_UBQLN2 KO_As 2 h_G3BP_╬▒-Syn-EGFP_eIF4G1.tif]

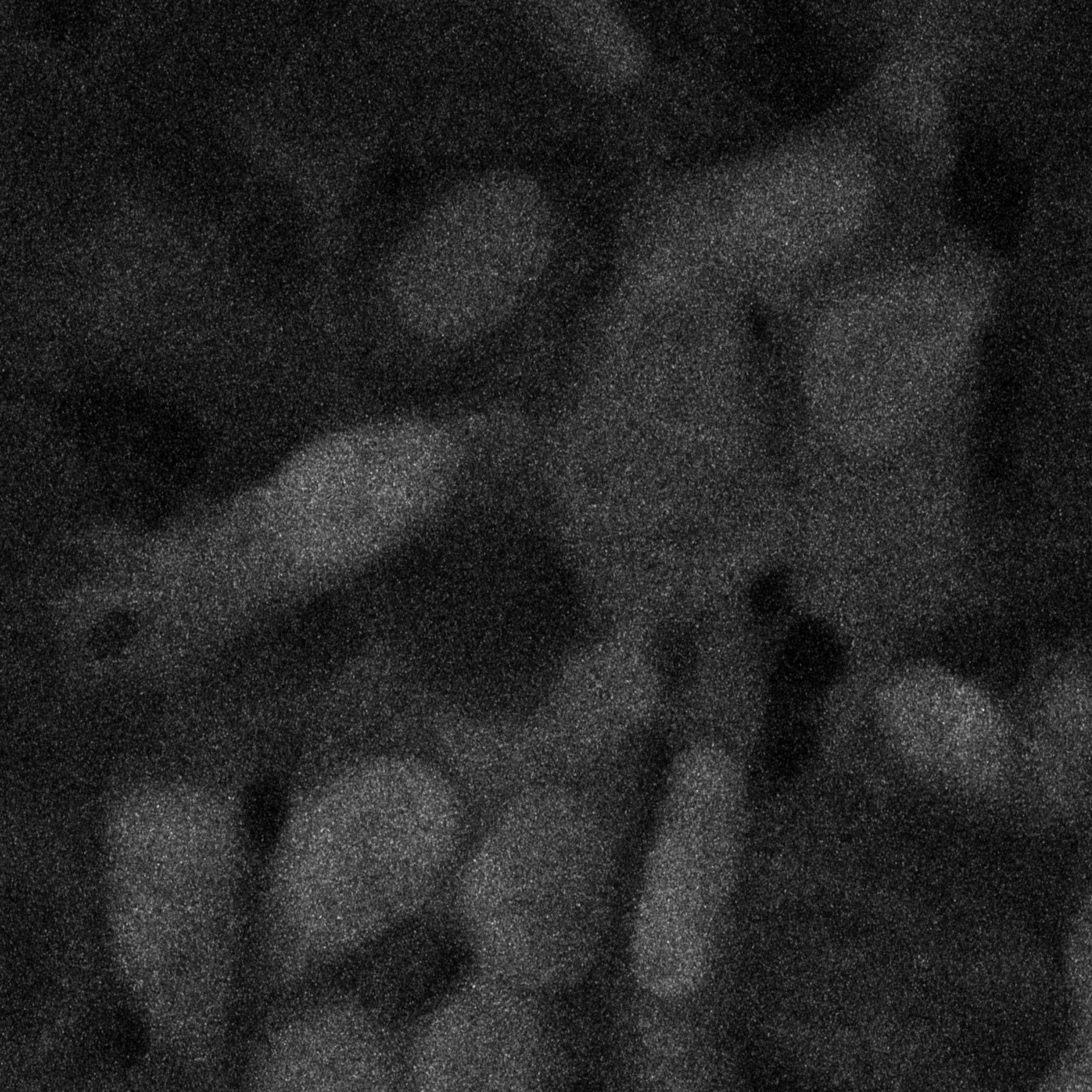

Supplement: Supplementary file 10 — Appendix Fig S1, S2, S3 Source Data [file 44318_2025_591_MOESM10_ESM.zip › Appendix Figure S3/S3C/01_Control KO_None_G3BP_╬▒-Syn-EGFP_eIF4G1.tif]

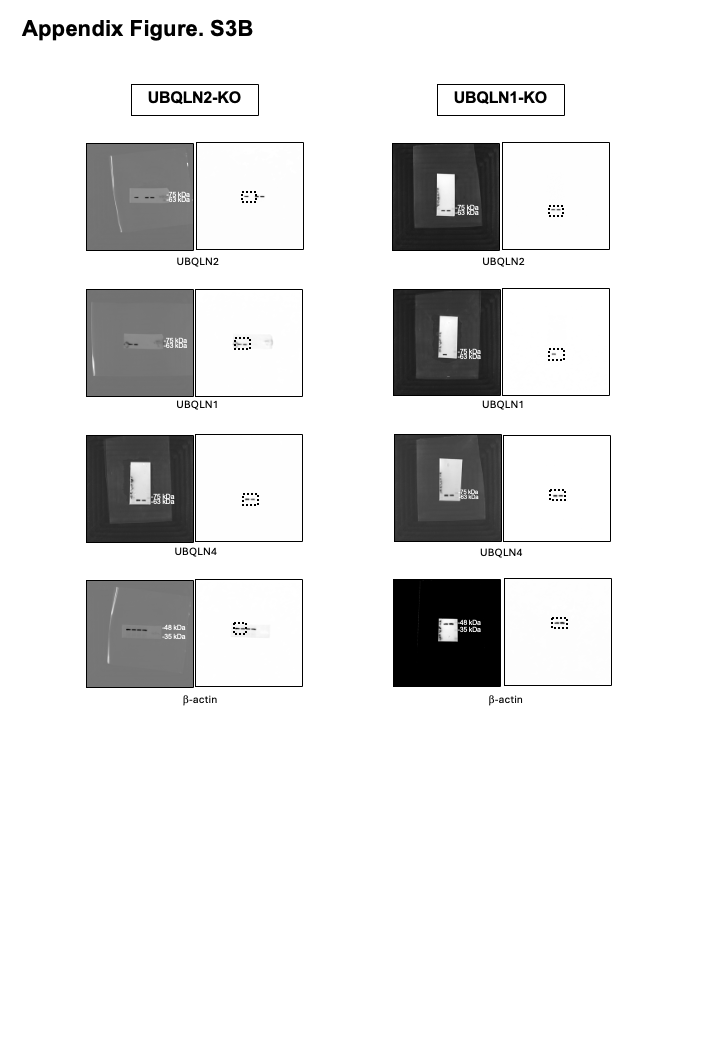

Supplement: Supplementary file 10 — Appendix Fig S1, S2, S3 Source Data [file 44318_2025_591_MOESM10_ESM.zip › Appendix Figure S3/S3B/09_Highlight of crop area.tiff]

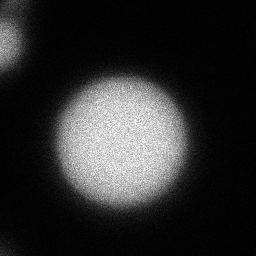

Supplement: Supplementary file 11 — Appendix Fig S4 Source Data [file 44318_2025_591_MOESM11_ESM.zip › Appendix Figure S4/S4/01_24 h_Control.tif]

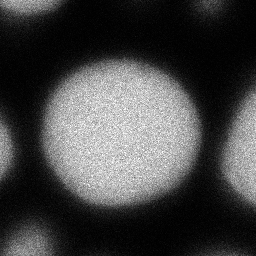

Supplement: Supplementary file 11 — Appendix Fig S4 Source Data [file 44318_2025_591_MOESM11_ESM.zip › Appendix Figure S4/S4/04_72 h_SO286.tif]

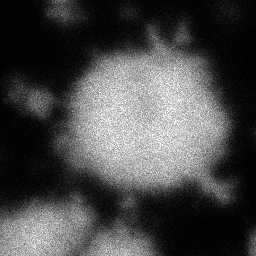

Supplement: Supplementary file 11 — Appendix Fig S4 Source Data [file 44318_2025_591_MOESM11_ESM.zip › Appendix Figure S4/S4/03_72 h_Control.tif]

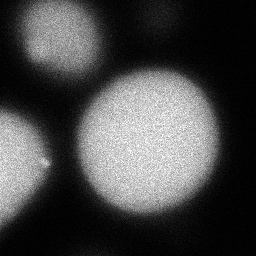

Supplement: Supplementary file 11 — Appendix Fig S4 Source Data [file 44318_2025_591_MOESM11_ESM.zip › Appendix Figure S4/S4/02_24 h_SO286.tif]

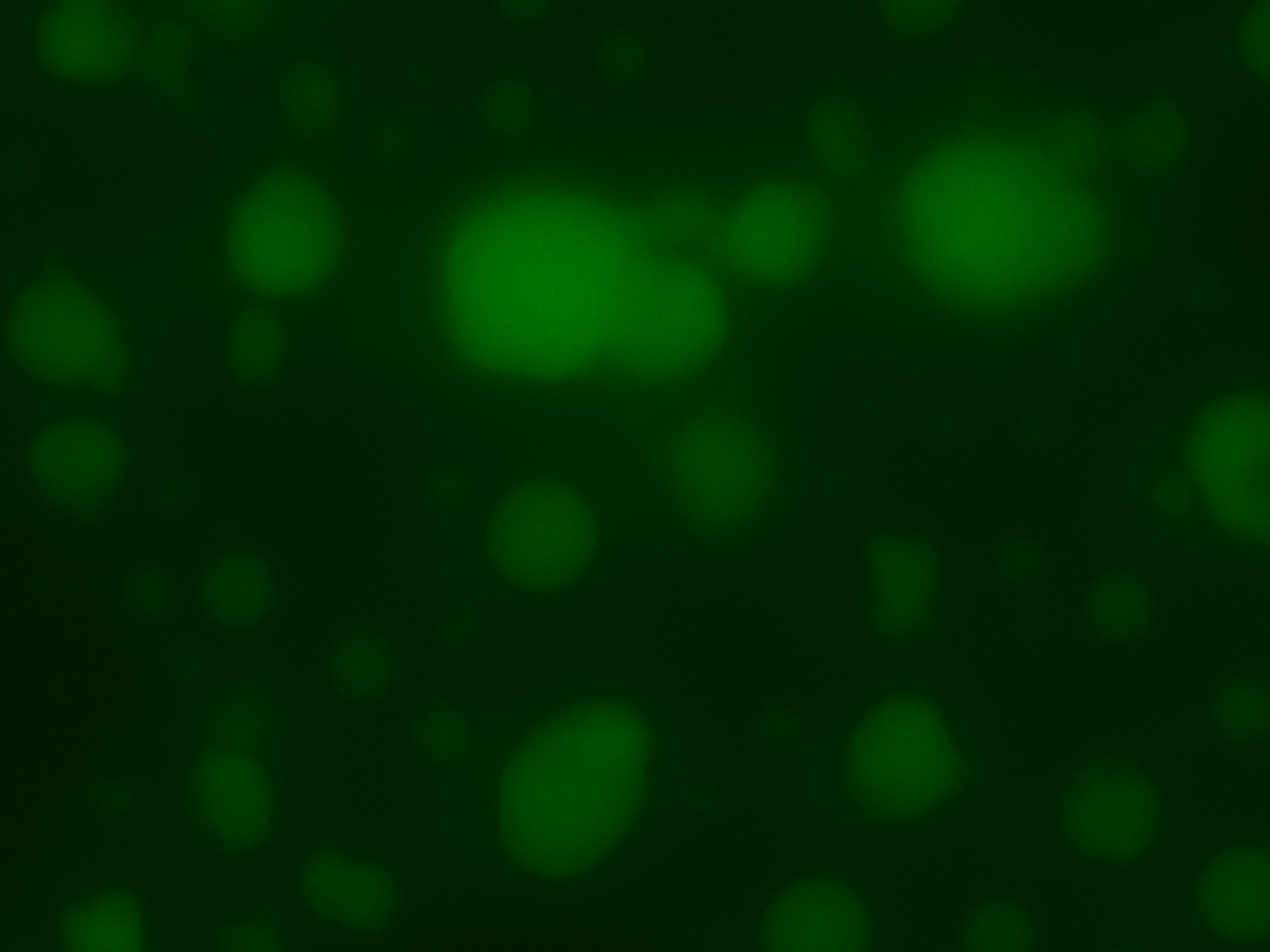

Supplement: Supplementary file 12 — Appendix Fig S5,S6 Source Data [file 44318_2025_591_MOESM12_ESM.zip › Appendix Figure S5/S5A/28_96 h_SO286(7 ╬╝M)_UBQLN2.tif]

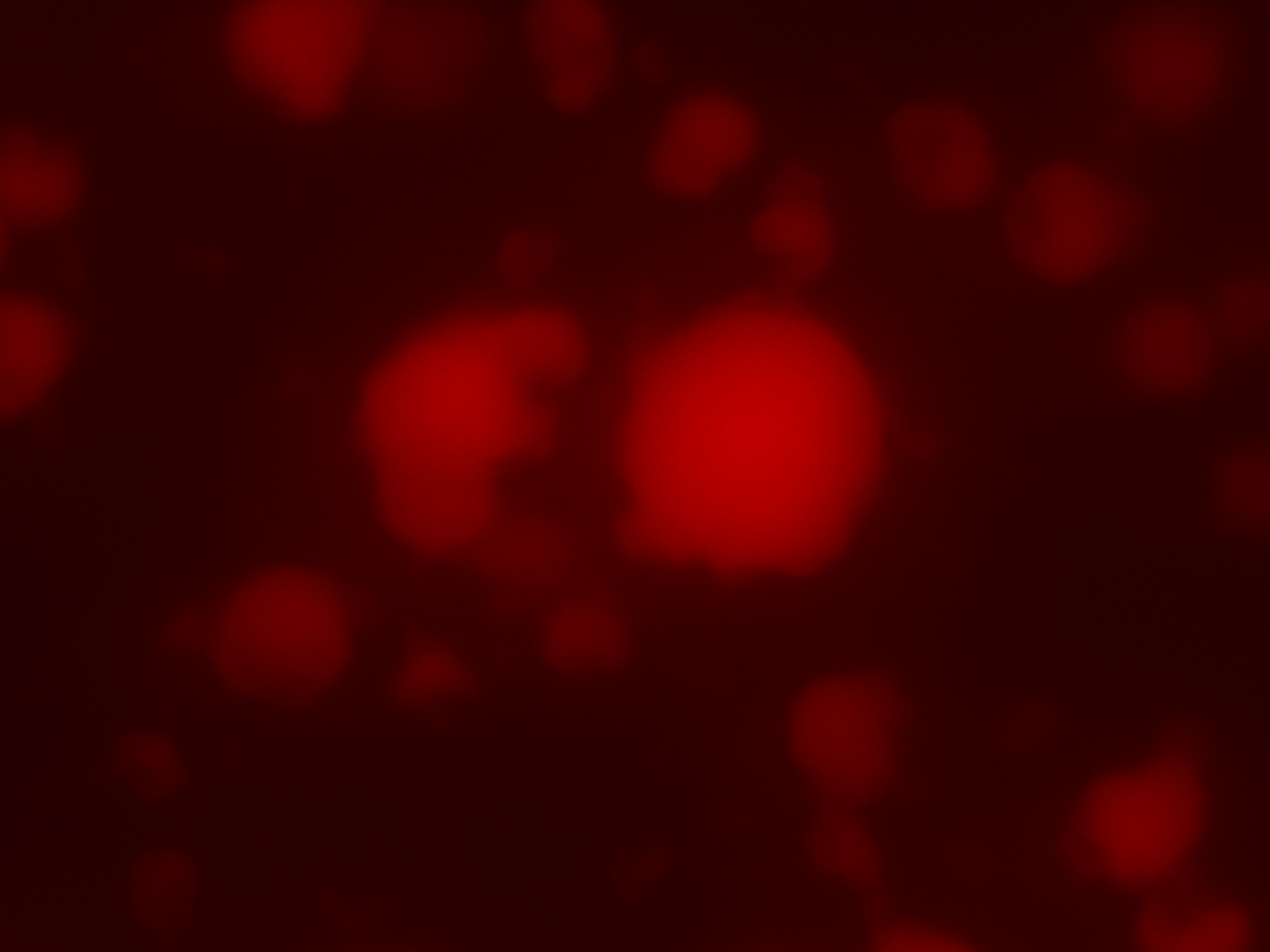

Supplement: Supplementary file 12 — Appendix Fig S5,S6 Source Data [file 44318_2025_591_MOESM12_ESM.zip › Appendix Figure S5/S5A/26_96 h_SO286(2 ╬╝M)_╬▒-Syn.tif]

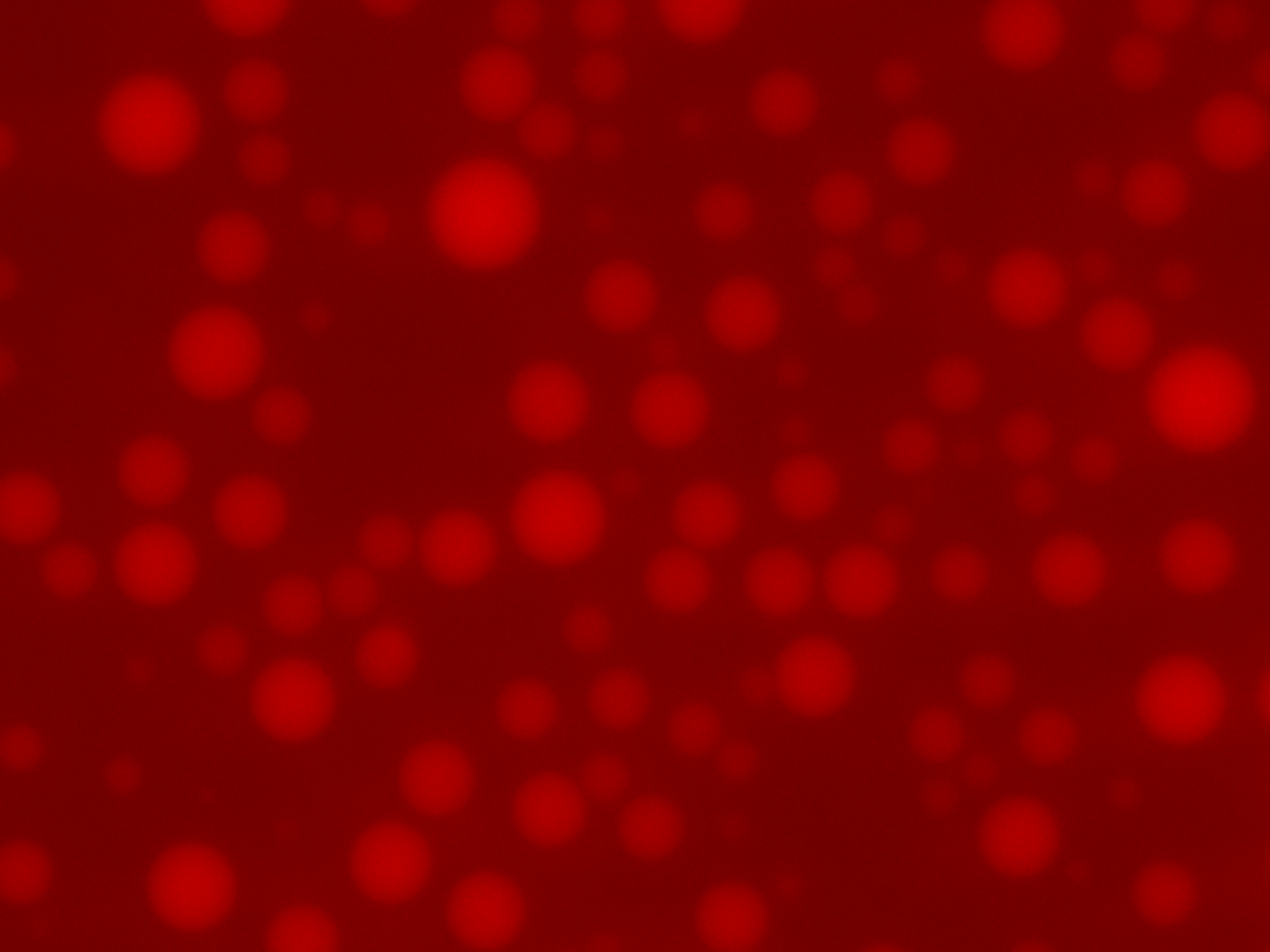

Supplement: Supplementary file 12 — Appendix Fig S5,S6 Source Data [file 44318_2025_591_MOESM12_ESM.zip › Appendix Figure S5/S5A/02_1 h_SO286(2 ╬╝M)_╬▒-Syn.tif]

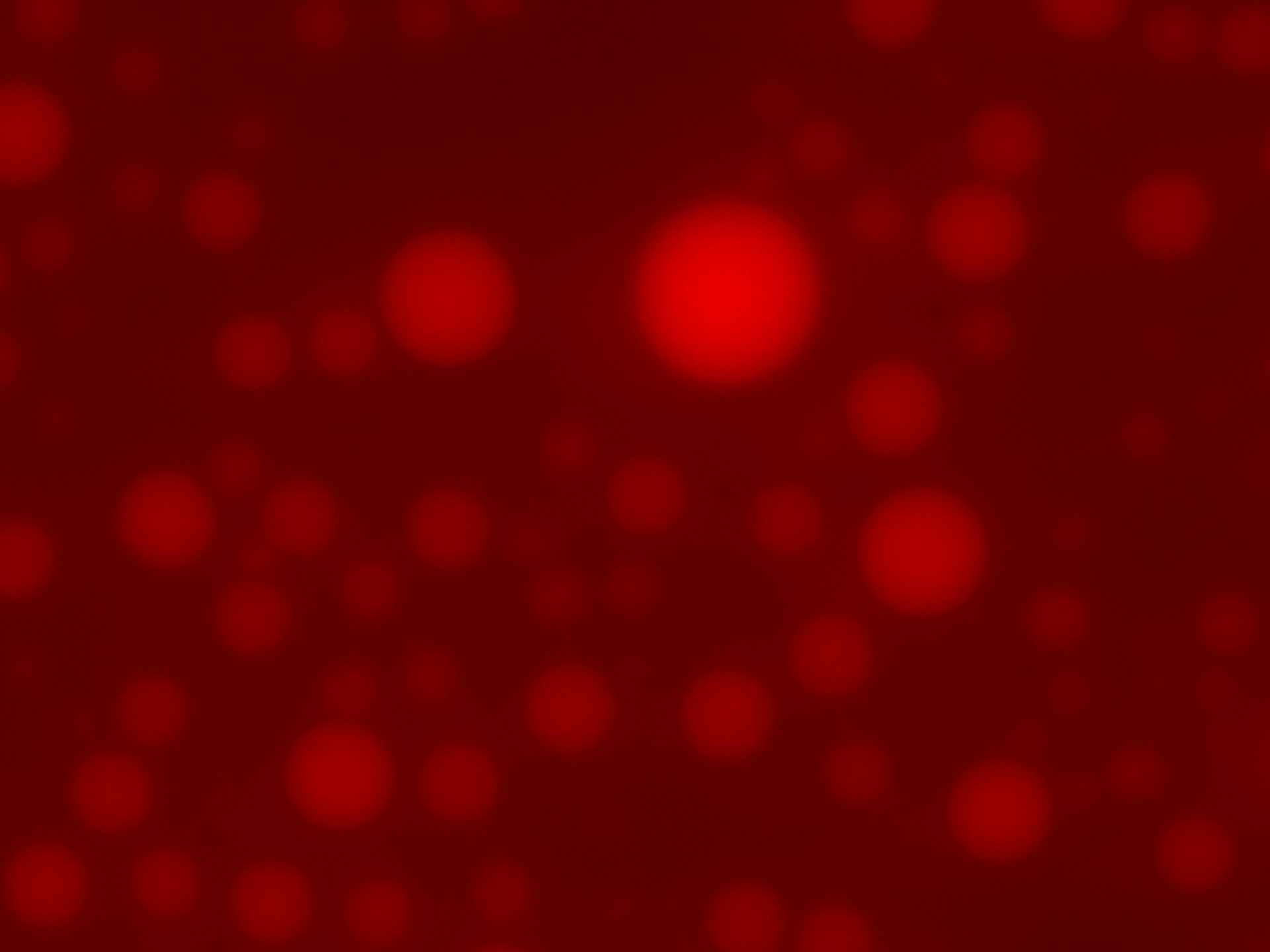

Supplement: Supplementary file 12 — Appendix Fig S5,S6 Source Data [file 44318_2025_591_MOESM12_ESM.zip › Appendix Figure S5/S5A/08_24 h_SO286(2 ╬╝M)_╬▒-Syn.tif]

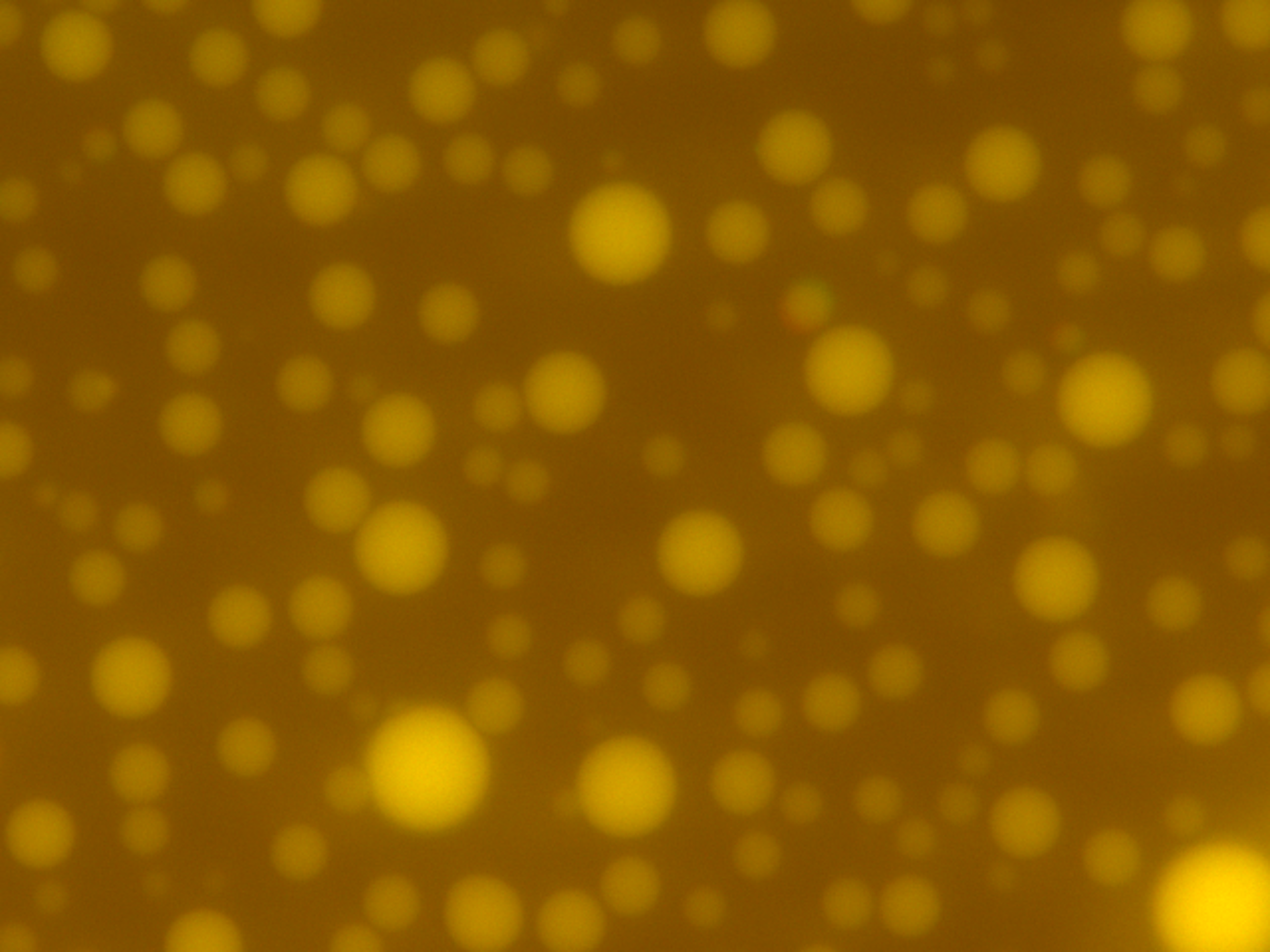

Supplement: Supplementary file 12 — Appendix Fig S5,S6 Source Data [file 44318_2025_591_MOESM12_ESM.zip › Appendix Figure S5/S5A/06_1 h_SO286(7 ╬╝M)_Merge.tif]

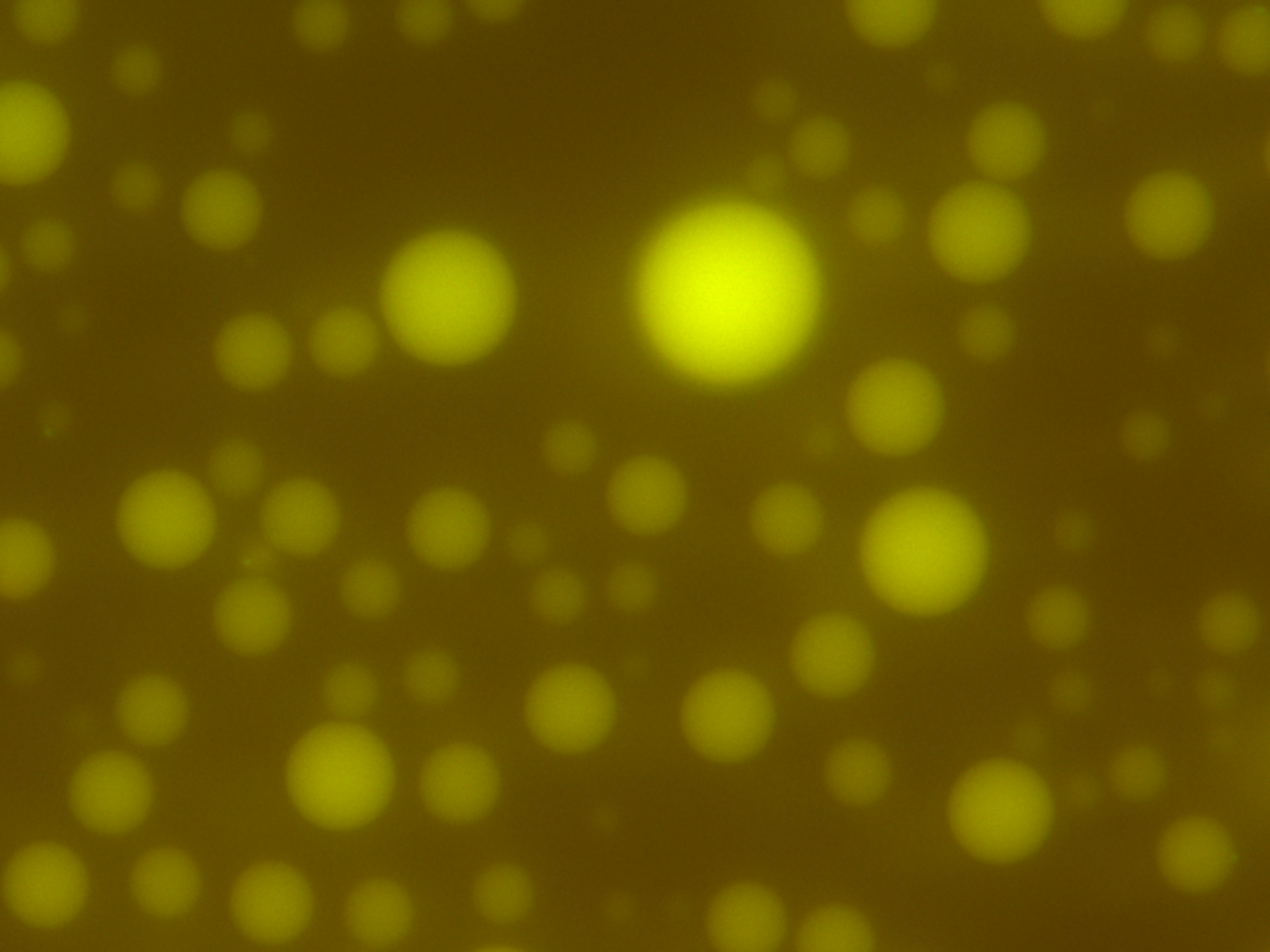

Supplement: Supplementary file 12 — Appendix Fig S5,S6 Source Data [file 44318_2025_591_MOESM12_ESM.zip › Appendix Figure S5/S5A/09_24 h_SO286(2 ╬╝M)_Merge.tif]

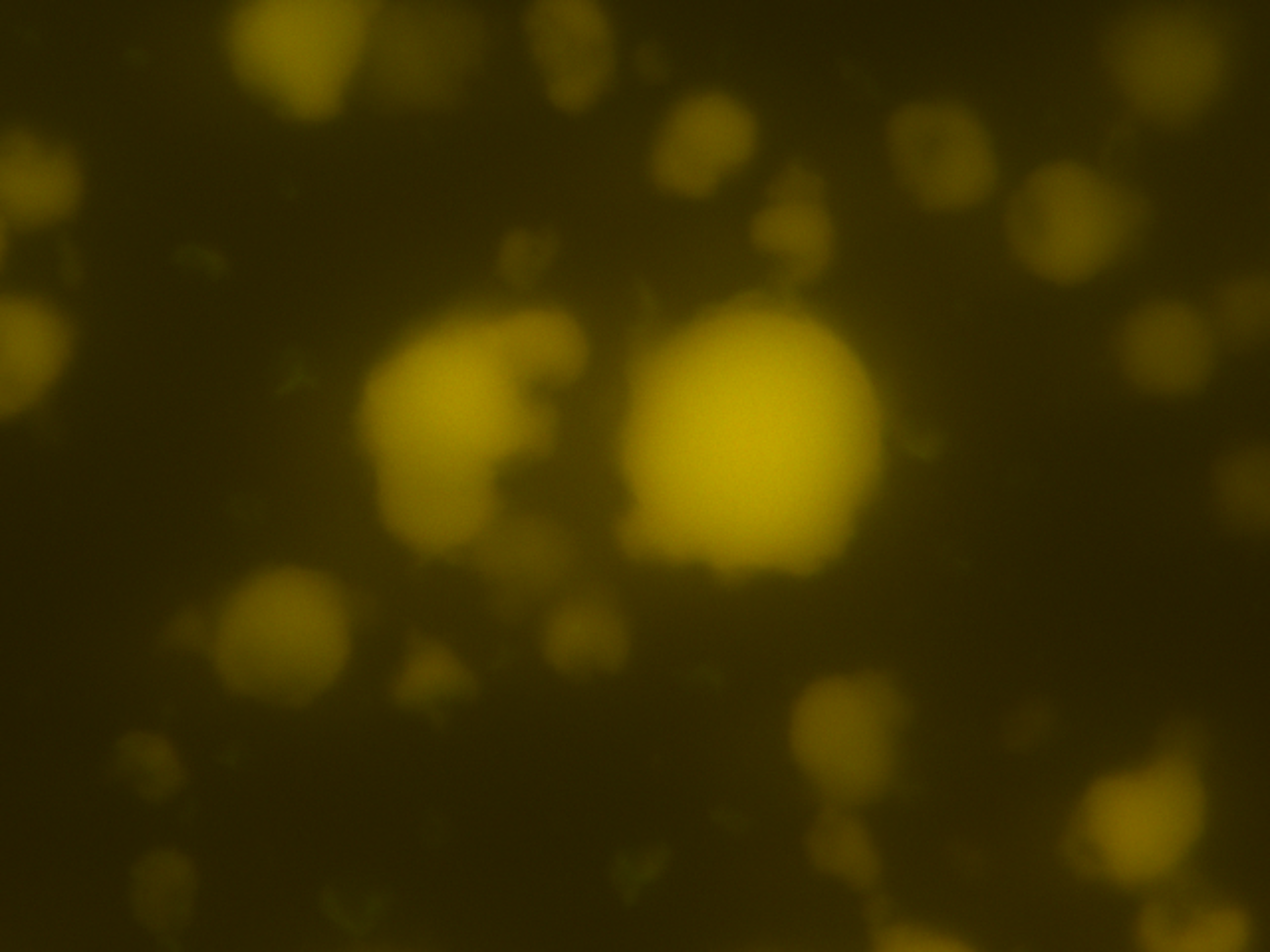

Supplement: Supplementary file 12 — Appendix Fig S5,S6 Source Data [file 44318_2025_591_MOESM12_ESM.zip › Appendix Figure S5/S5A/27_96 h_SO286(2 ╬╝M)_Merge.tif]

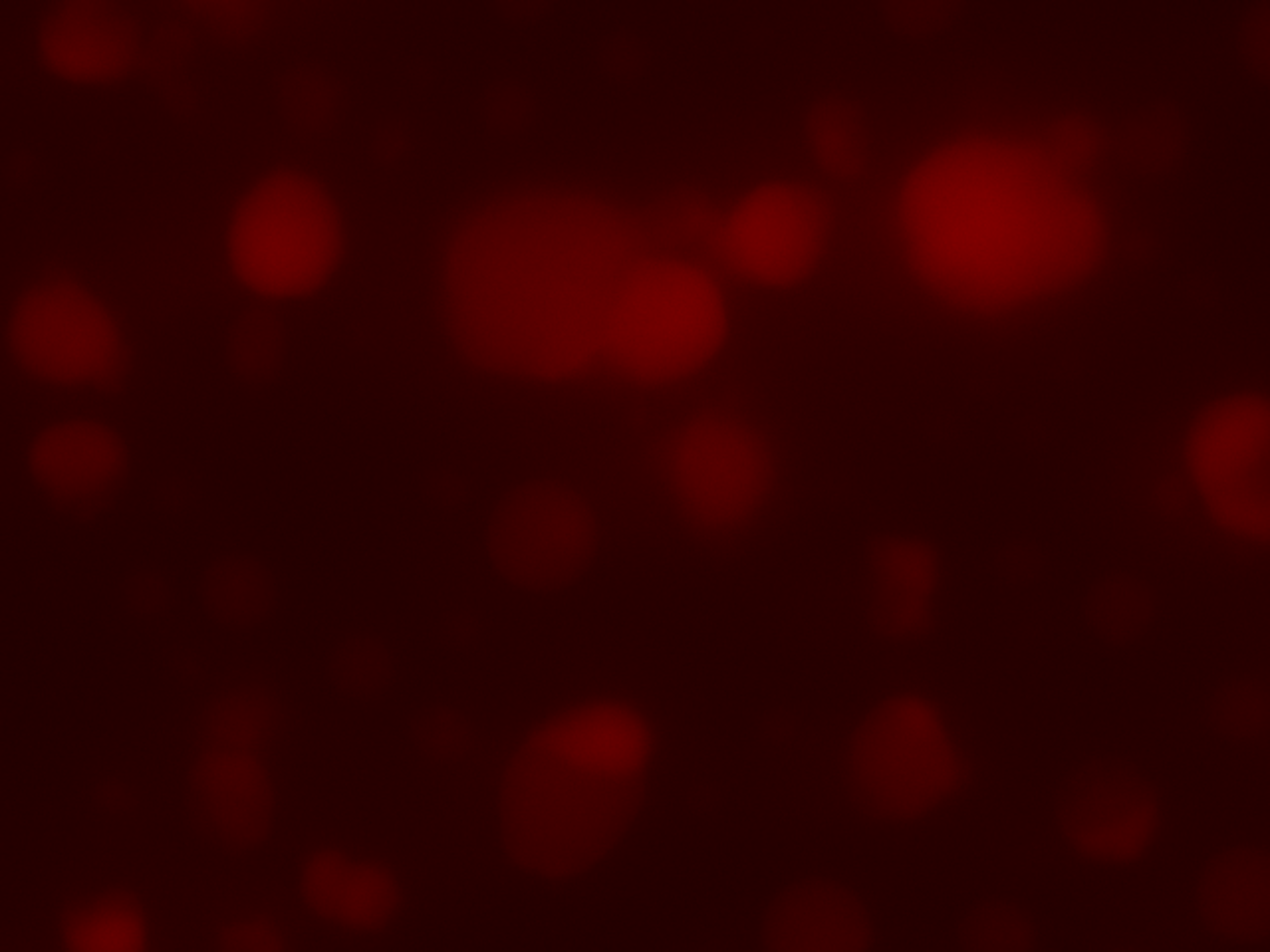

Supplement: Supplementary file 12 — Appendix Fig S5,S6 Source Data [file 44318_2025_591_MOESM12_ESM.zip › Appendix Figure S5/S5A/29_96 h_SO286(7 ╬╝M)_╬▒-Syn.tif]

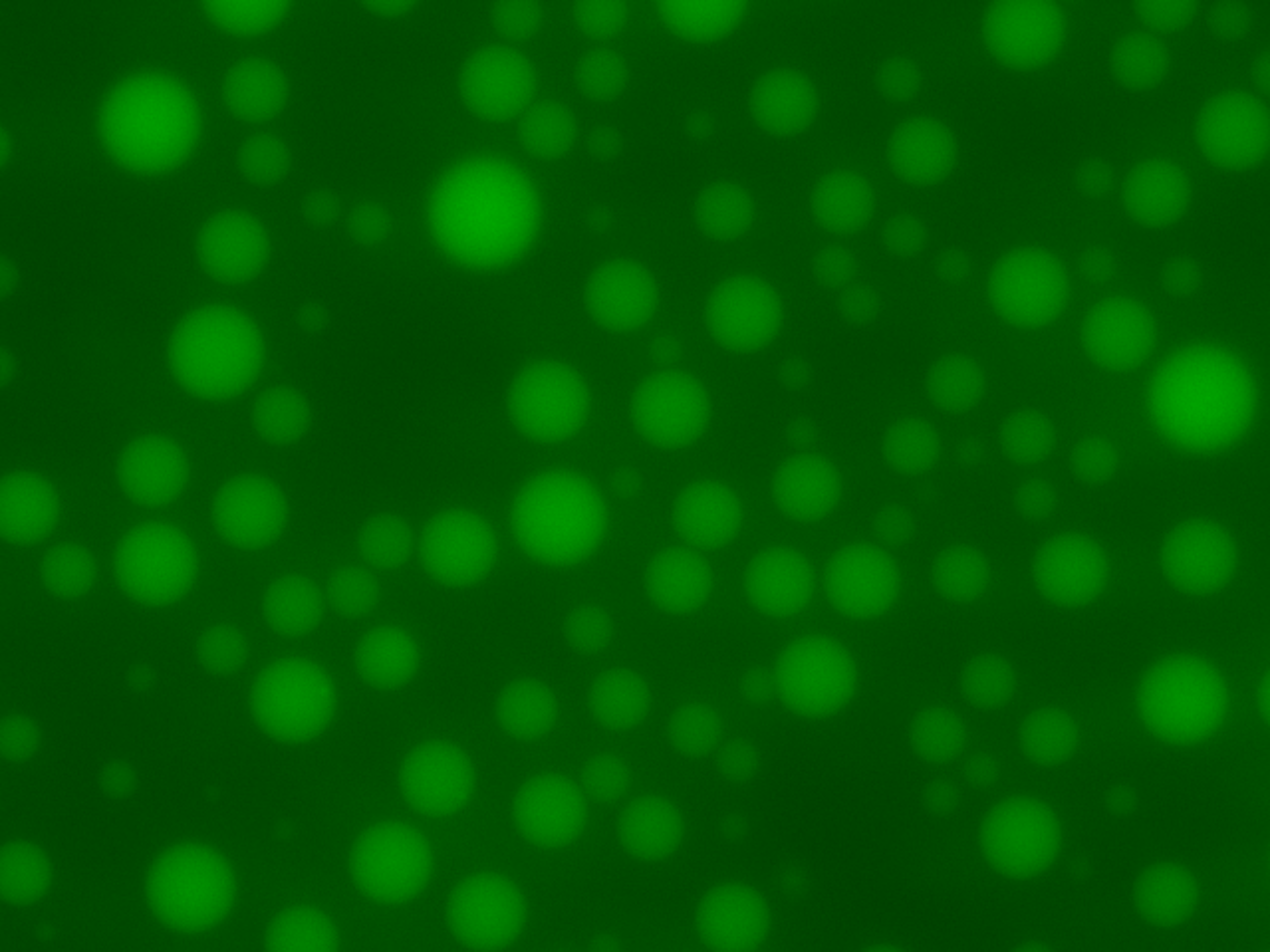

Supplement: Supplementary file 12 — Appendix Fig S5,S6 Source Data [file 44318_2025_591_MOESM12_ESM.zip › Appendix Figure S5/S5A/01_1 h_SO286(2 ╬╝M)_UBQLN2.tif]

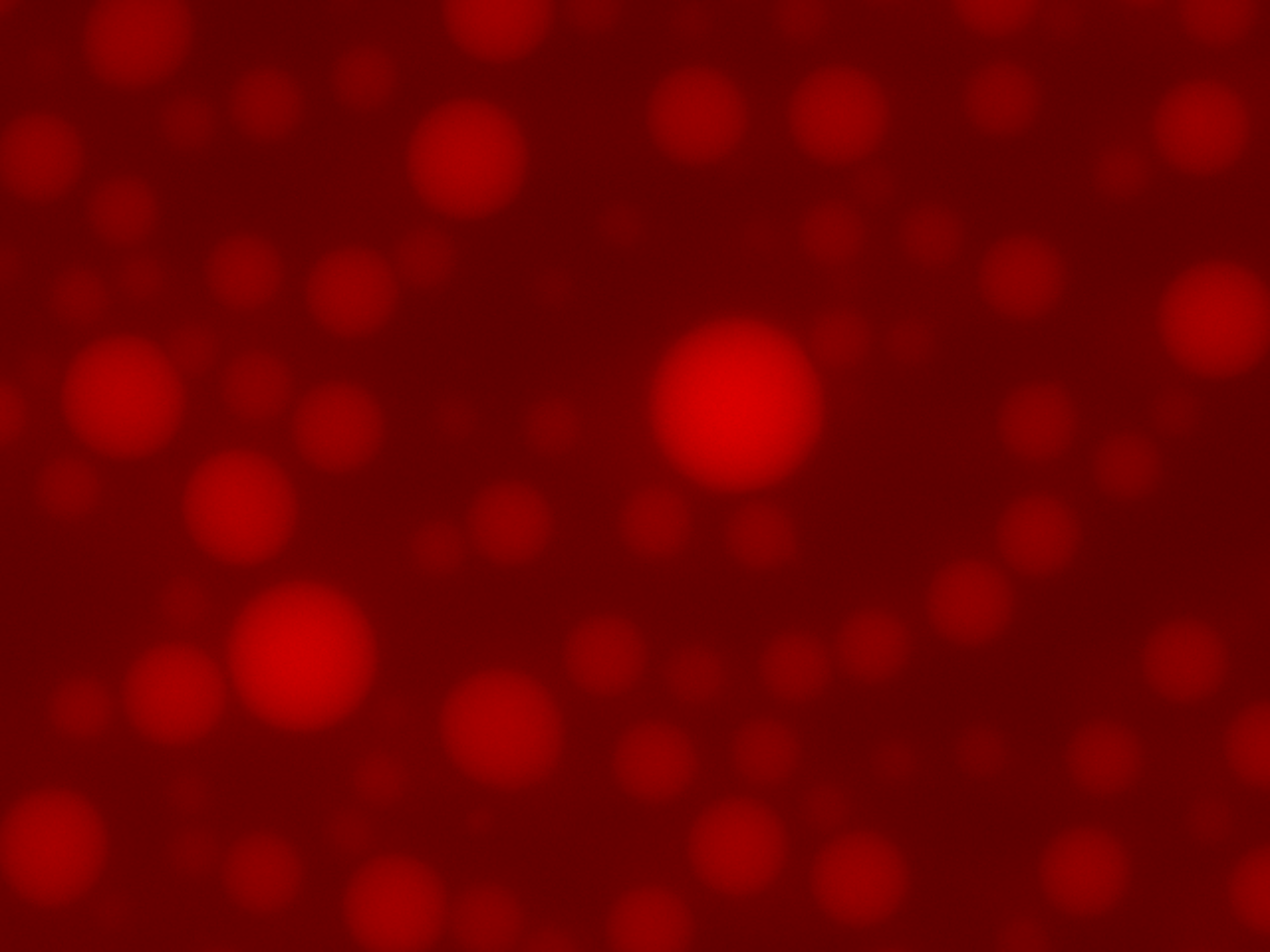

Supplement: Supplementary file 12 — Appendix Fig S5,S6 Source Data [file 44318_2025_591_MOESM12_ESM.zip › Appendix Figure S5/S5A/11_24 h_SO286(7 ╬╝M)_╬▒-Syn.tif]

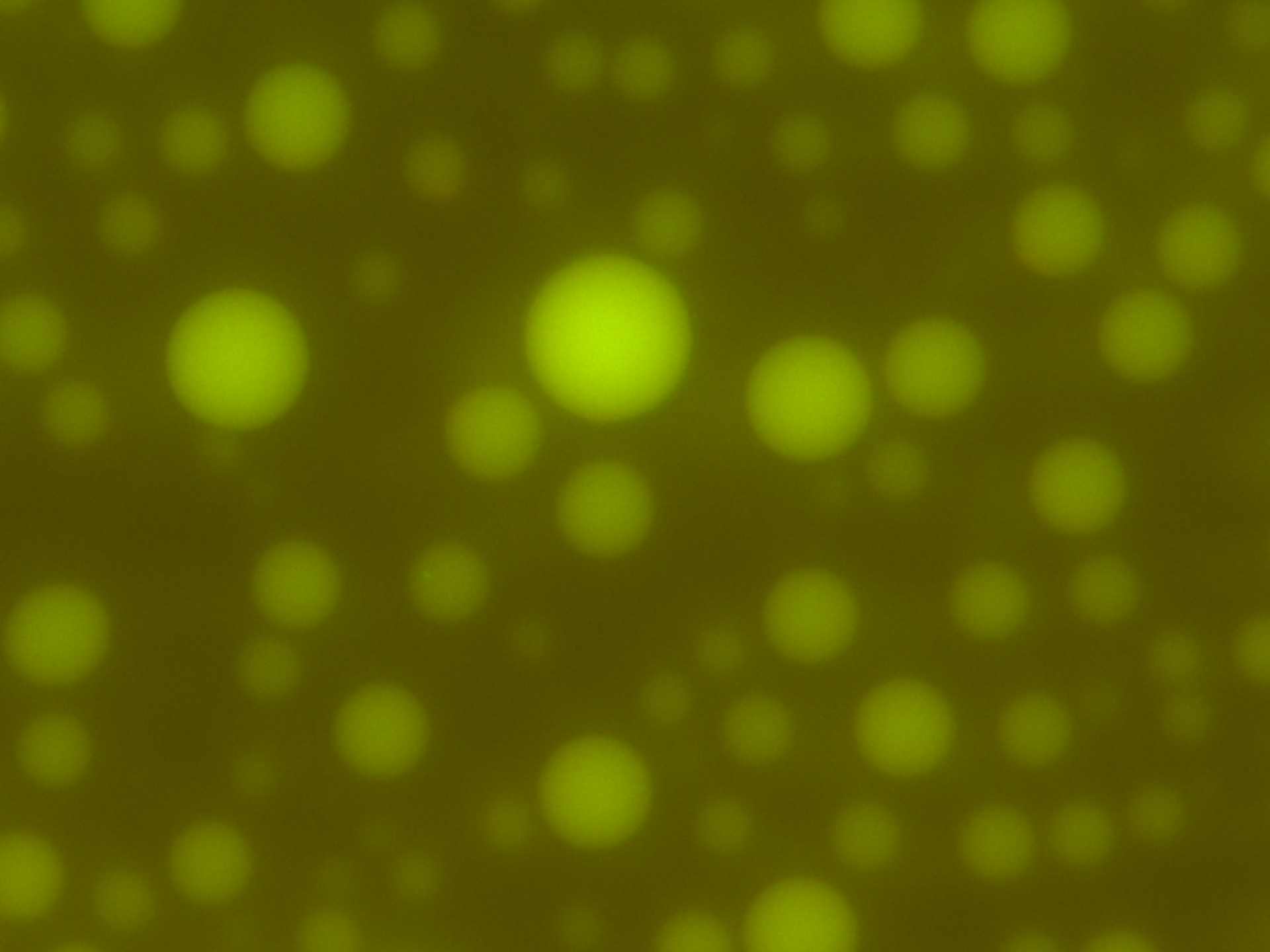

Supplement: Supplementary file 12 — Appendix Fig S5,S6 Source Data [file 44318_2025_591_MOESM12_ESM.zip › Appendix Figure S5/S5A/18_48 h_SO286(7 ╬╝M)_Merge.tif]

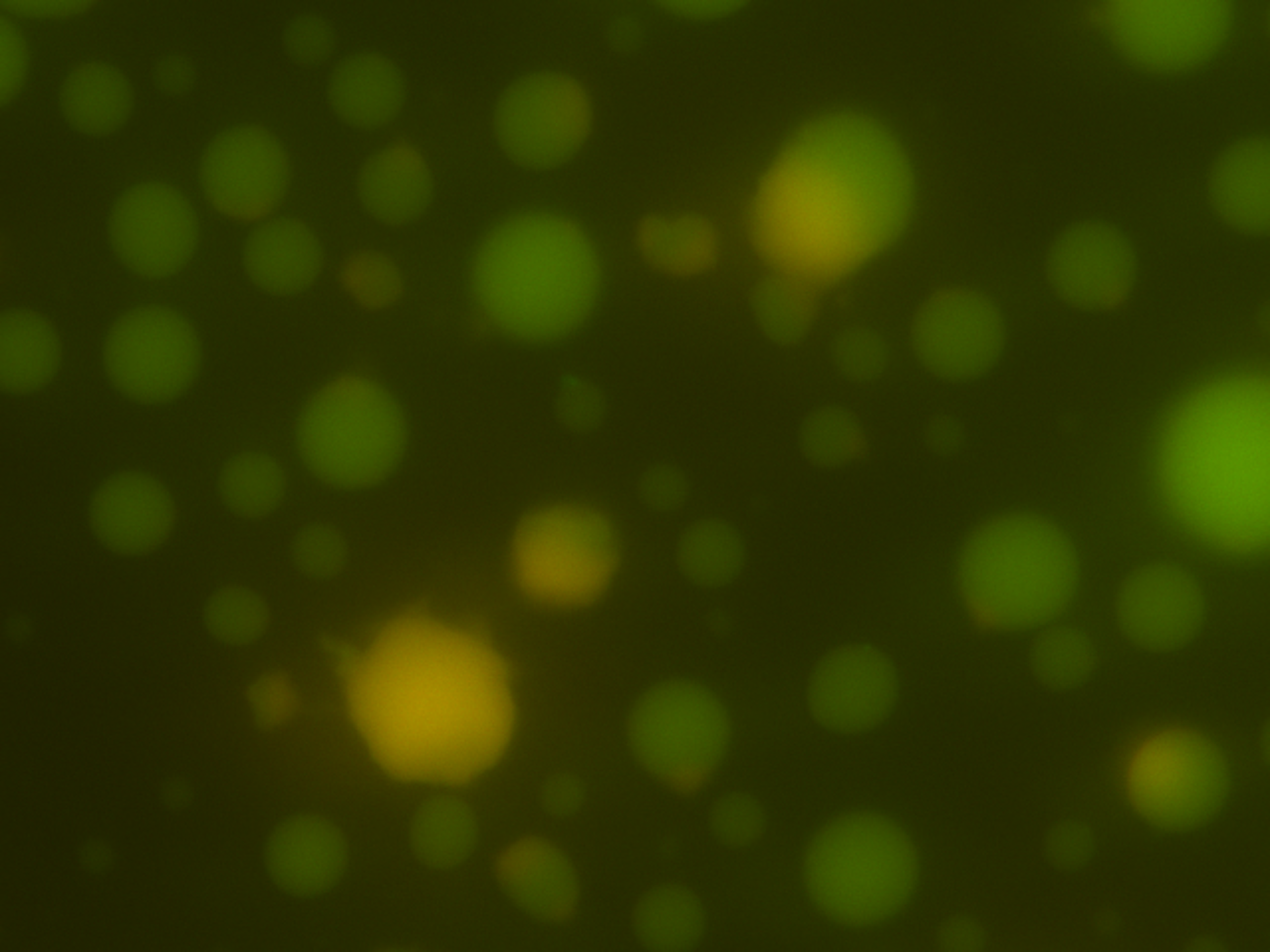

Supplement: Supplementary file 12 — Appendix Fig S5,S6 Source Data [file 44318_2025_591_MOESM12_ESM.zip › Appendix Figure S5/S5A/24_72 h_SO286(7 ╬╝M)_Merge.tif]

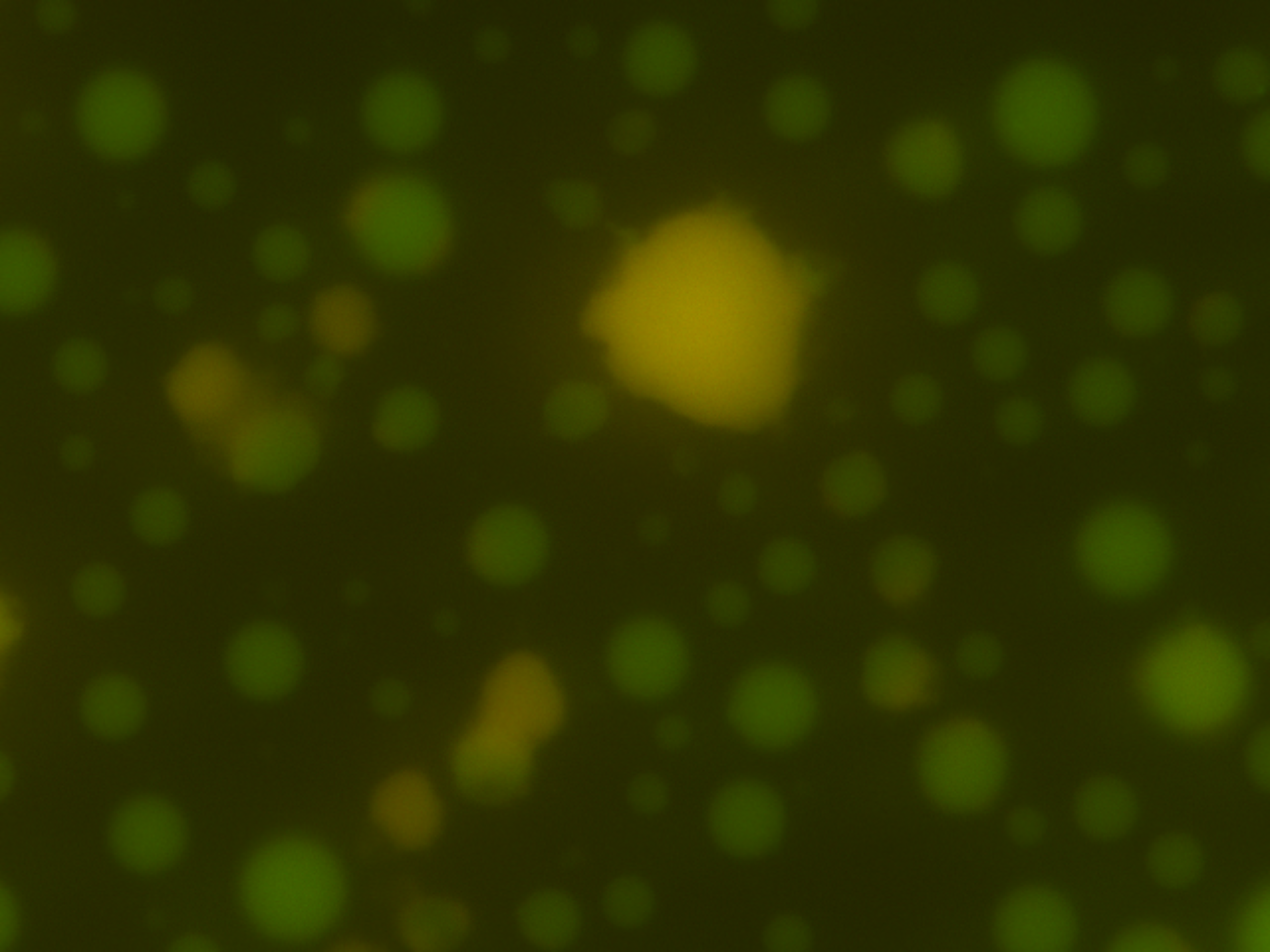

Supplement: Supplementary file 12 — Appendix Fig S5,S6 Source Data [file 44318_2025_591_MOESM12_ESM.zip › Appendix Figure S5/S5A/21_72 h_SO286(2 ╬╝M)_Merge.tif]

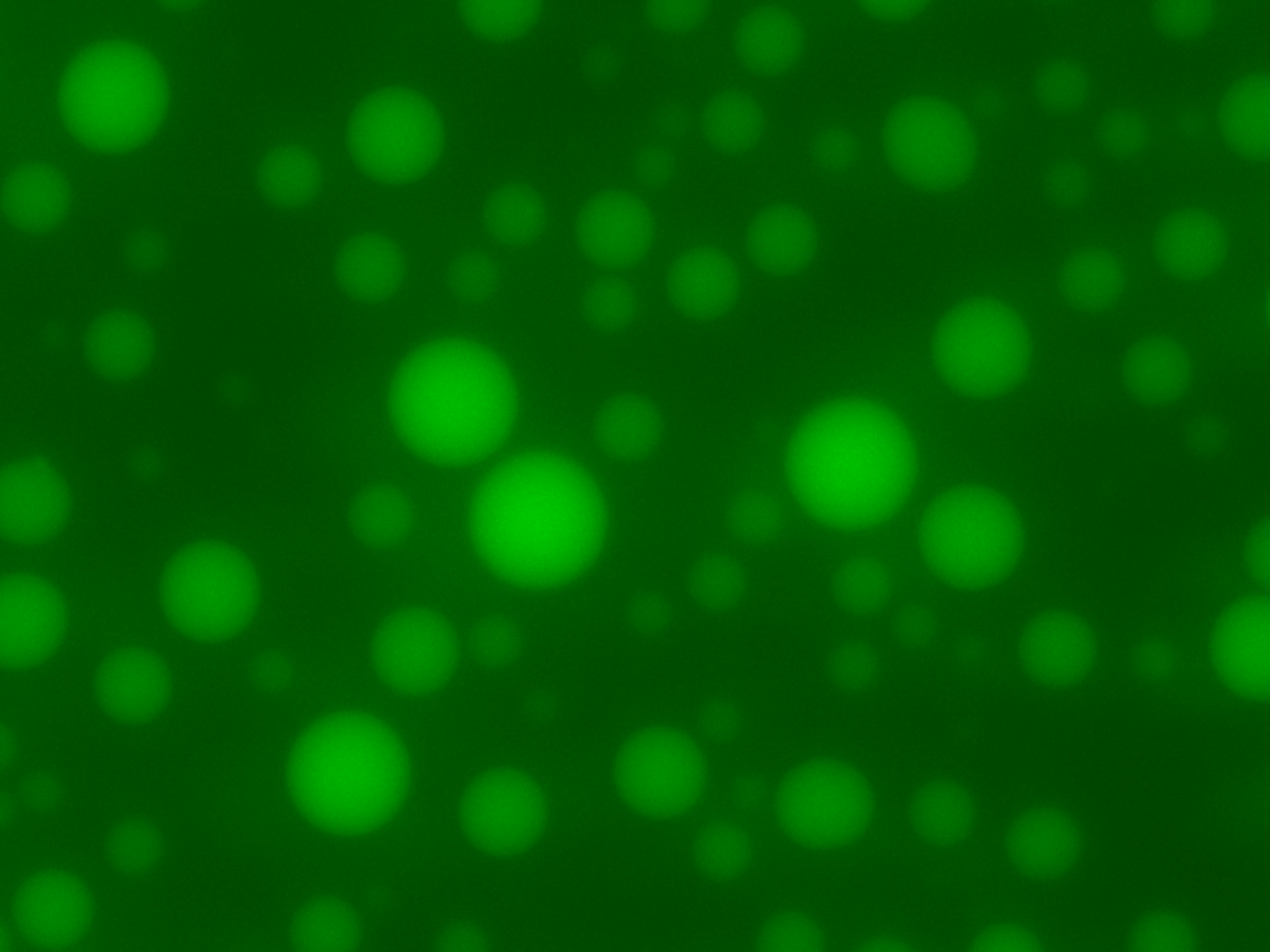

Supplement: Supplementary file 12 — Appendix Fig S5,S6 Source Data [file 44318_2025_591_MOESM12_ESM.zip › Appendix Figure S5/S5A/13_48 h_SO286(2 ╬╝M)_UBQLN2.tif]

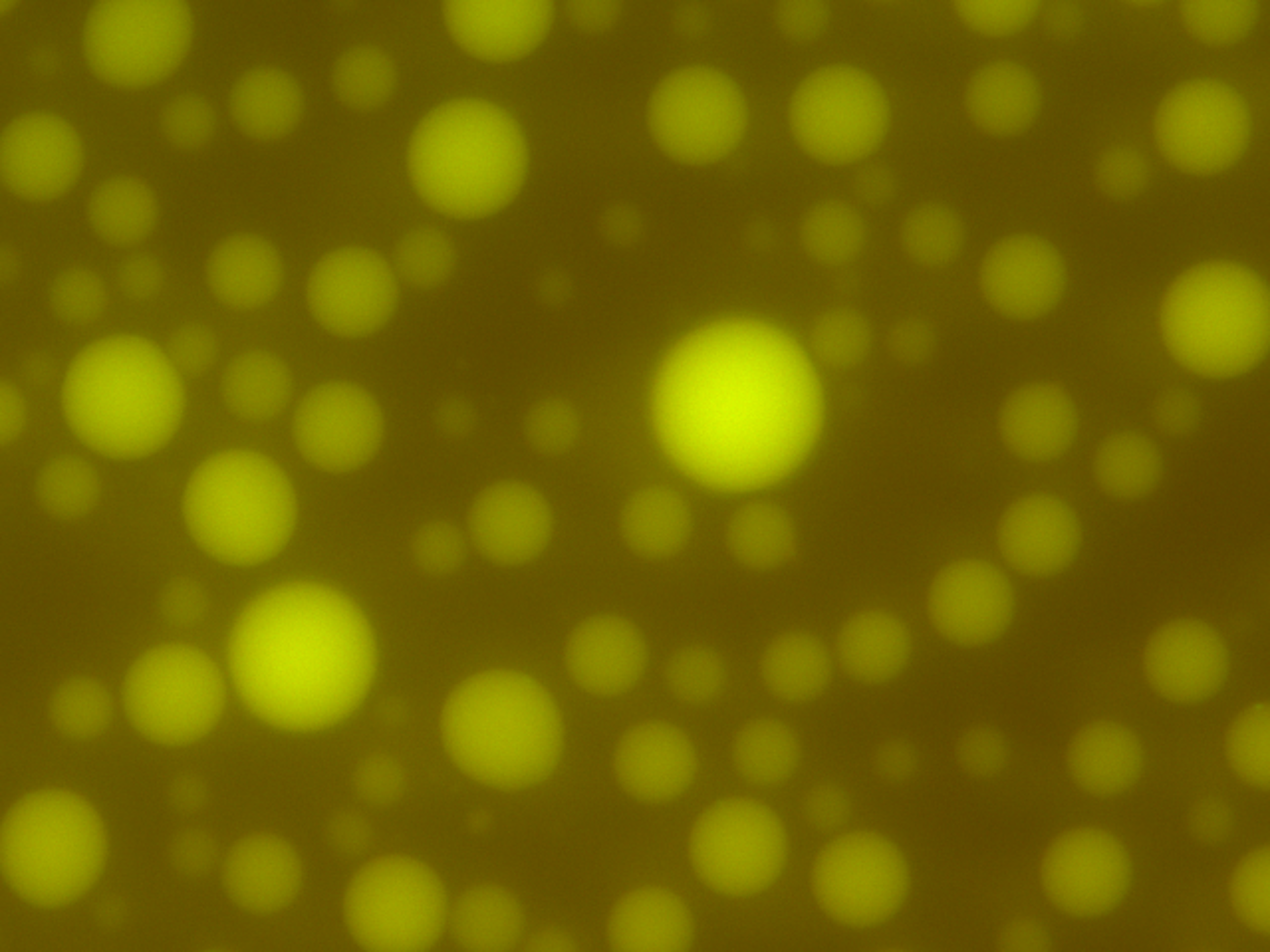

Supplement: Supplementary file 12 — Appendix Fig S5,S6 Source Data [file 44318_2025_591_MOESM12_ESM.zip › Appendix Figure S5/S5A/12_24 h_SO286(7 ╬╝M)_Merge.tif]

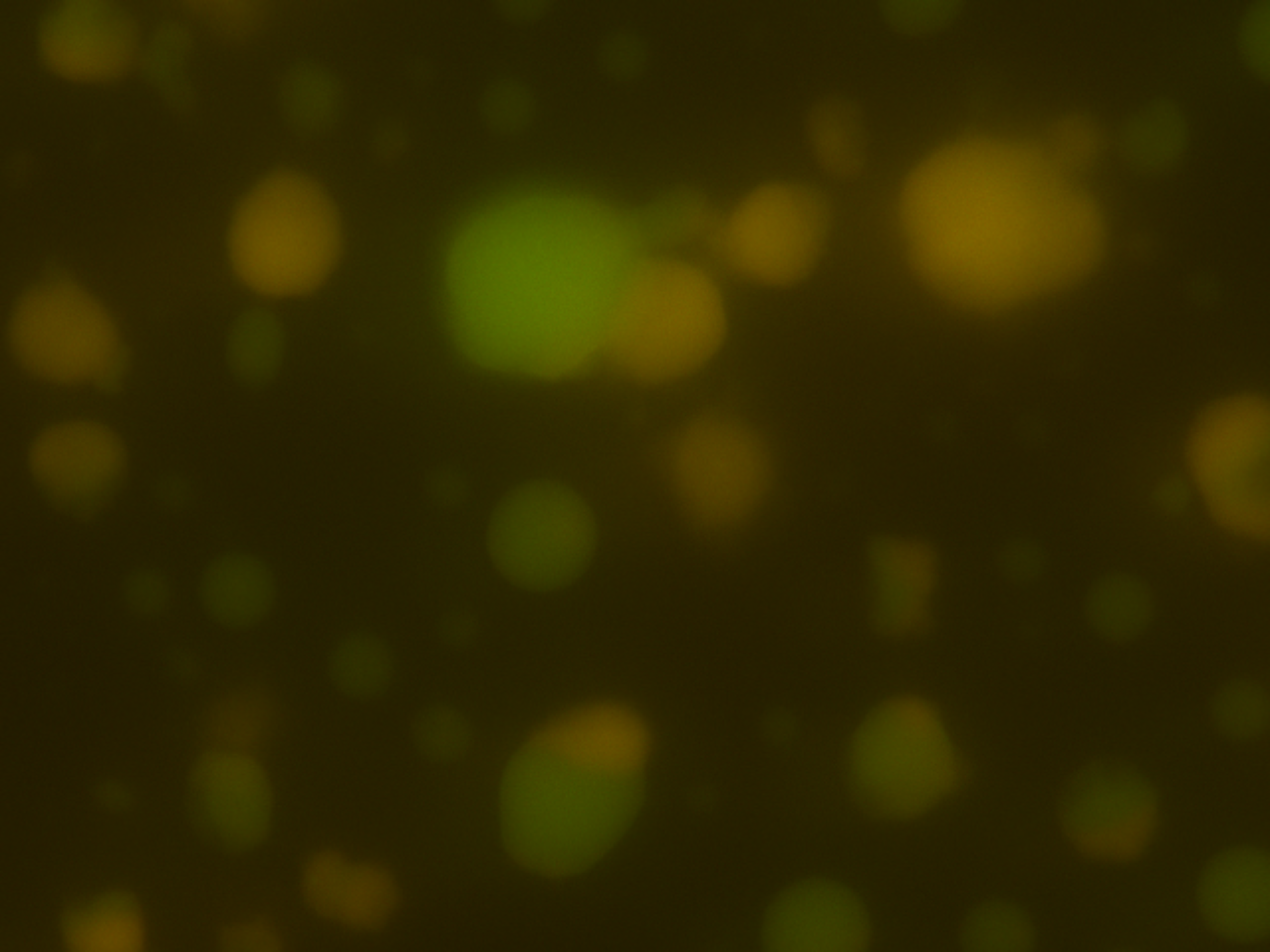

Supplement: Supplementary file 12 — Appendix Fig S5,S6 Source Data [file 44318_2025_591_MOESM12_ESM.zip › Appendix Figure S5/S5A/30_96 h_SO286(7 ╬╝M)_Merge.tif]

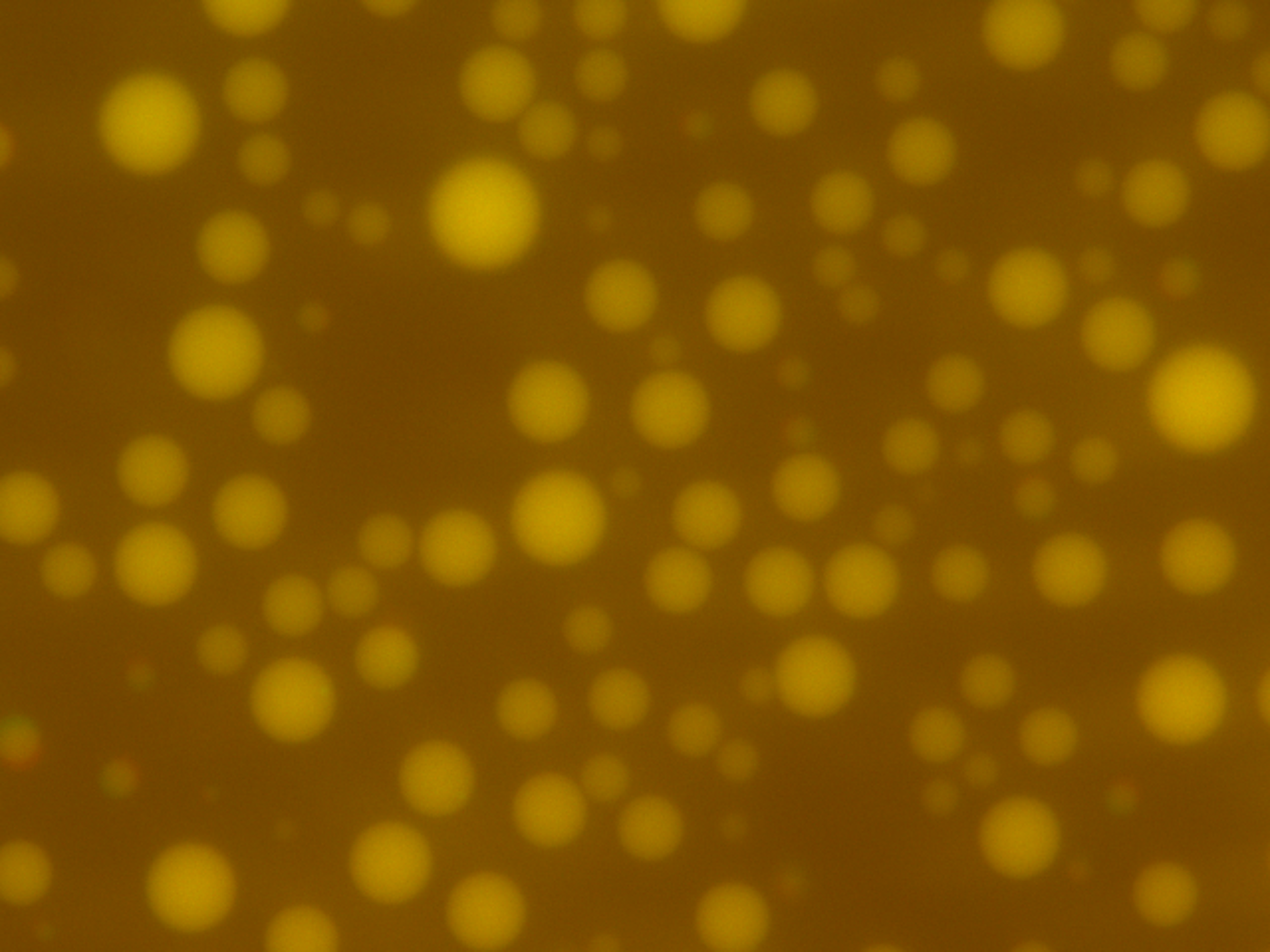

Supplement: Supplementary file 12 — Appendix Fig S5,S6 Source Data [file 44318_2025_591_MOESM12_ESM.zip › Appendix Figure S5/S5A/03_1 h_SO286(2 ╬╝M)_Merge.tif]

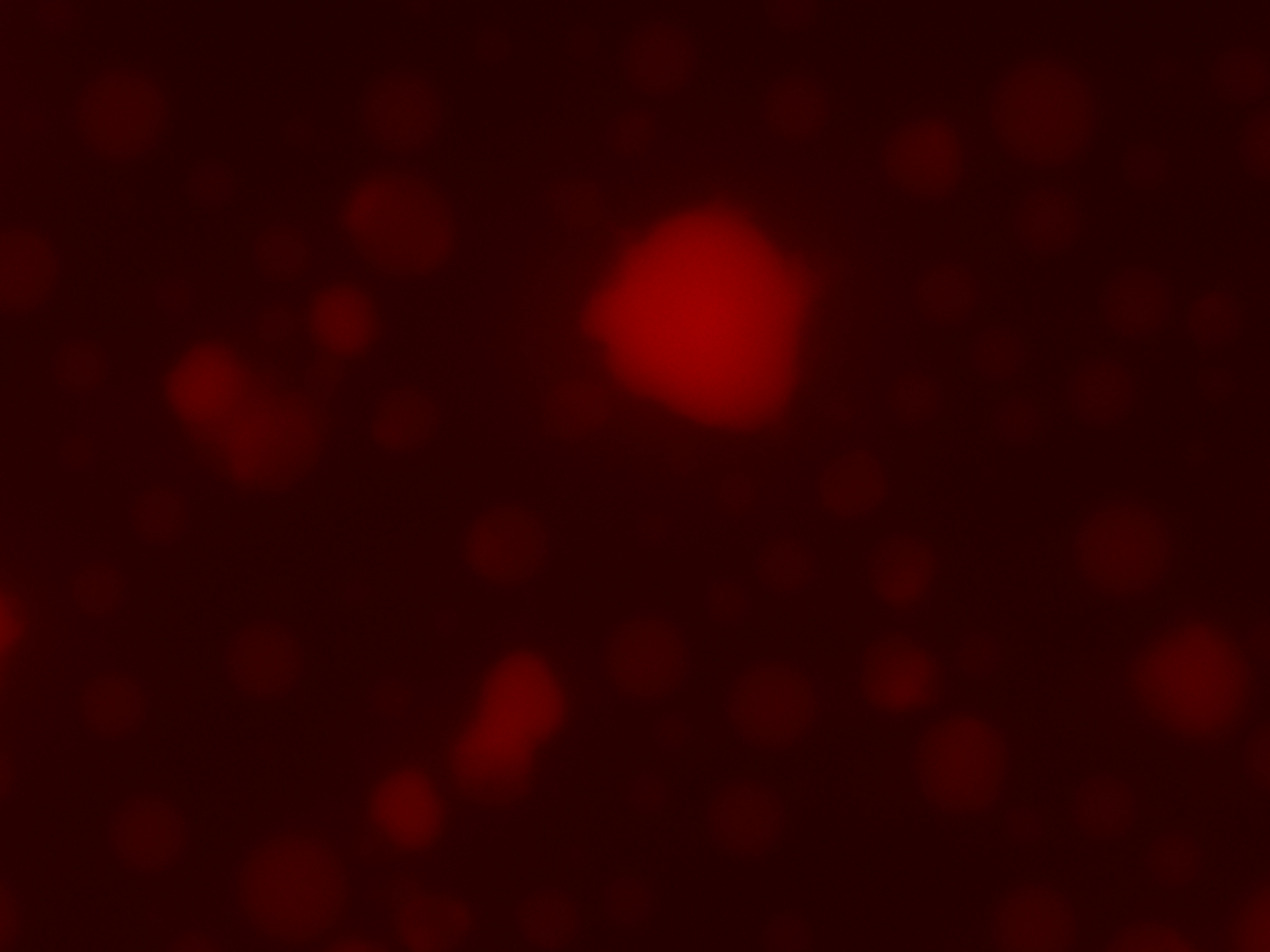

Supplement: Supplementary file 12 — Appendix Fig S5,S6 Source Data [file 44318_2025_591_MOESM12_ESM.zip › Appendix Figure S5/S5A/20_72 h_SO286(2 ╬╝M)_╬▒-Syn.tif]

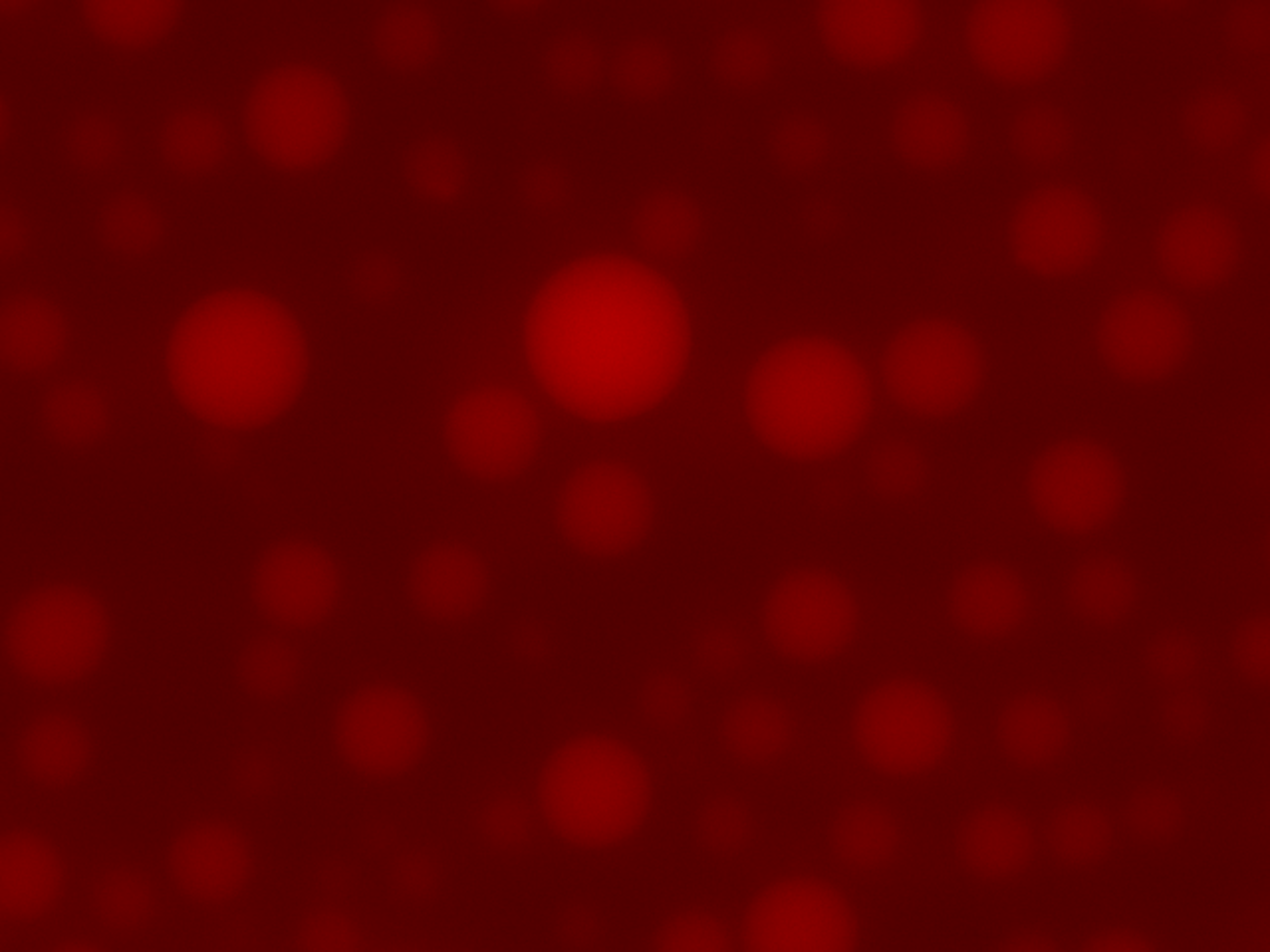

Supplement: Supplementary file 12 — Appendix Fig S5,S6 Source Data [file 44318_2025_591_MOESM12_ESM.zip › Appendix Figure S5/S5A/17_48 h_SO286(7 ╬╝M)_╬▒-Syn.tif]

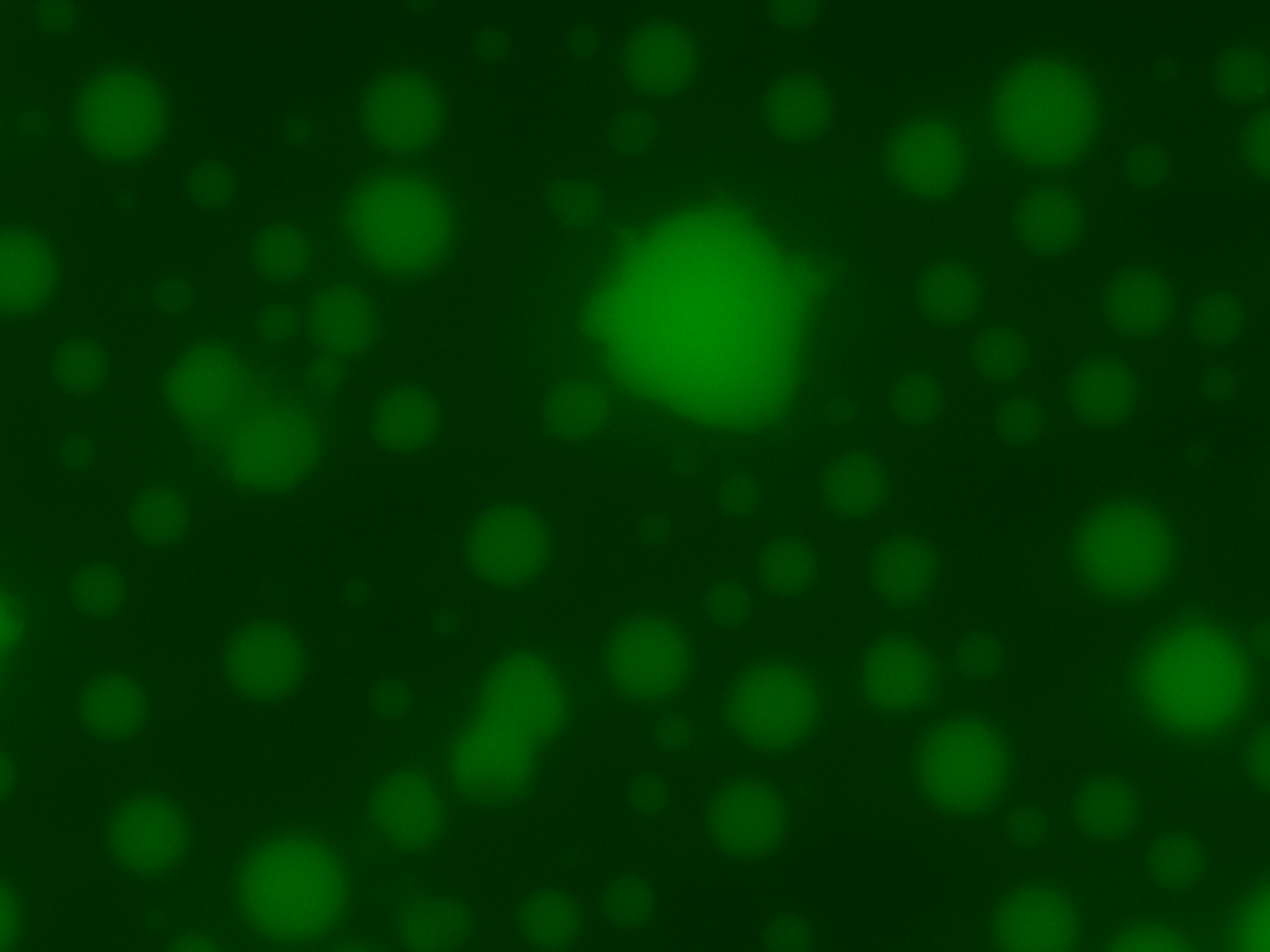

Supplement: Supplementary file 12 — Appendix Fig S5,S6 Source Data [file 44318_2025_591_MOESM12_ESM.zip › Appendix Figure S5/S5A/19_72 h_SO286(2 ╬╝M)_UBQLN2.tif]

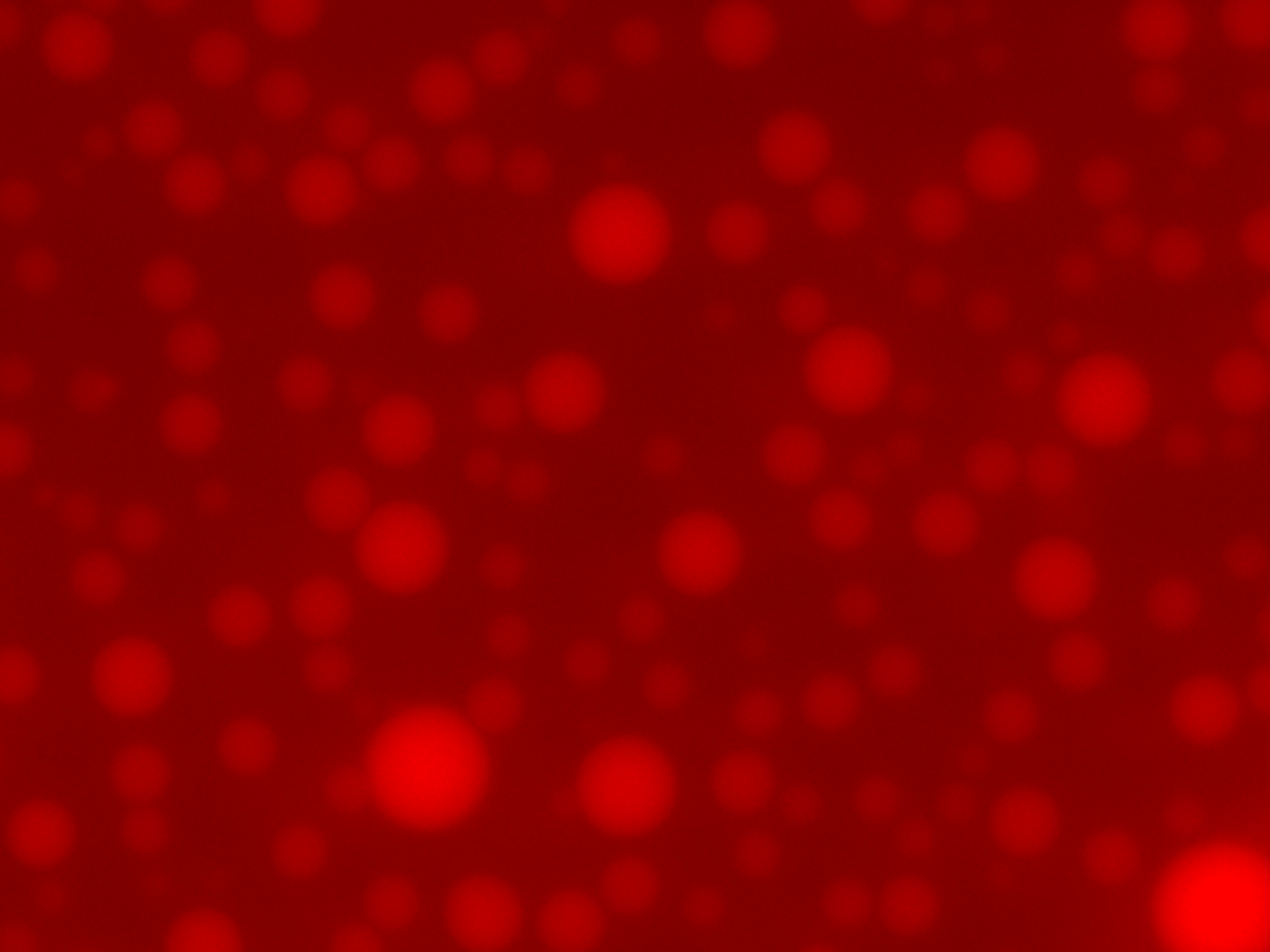

Supplement: Supplementary file 12 — Appendix Fig S5,S6 Source Data [file 44318_2025_591_MOESM12_ESM.zip › Appendix Figure S5/S5A/05_1 h_SO286(7 ╬╝M)_╬▒-Syn.tif]

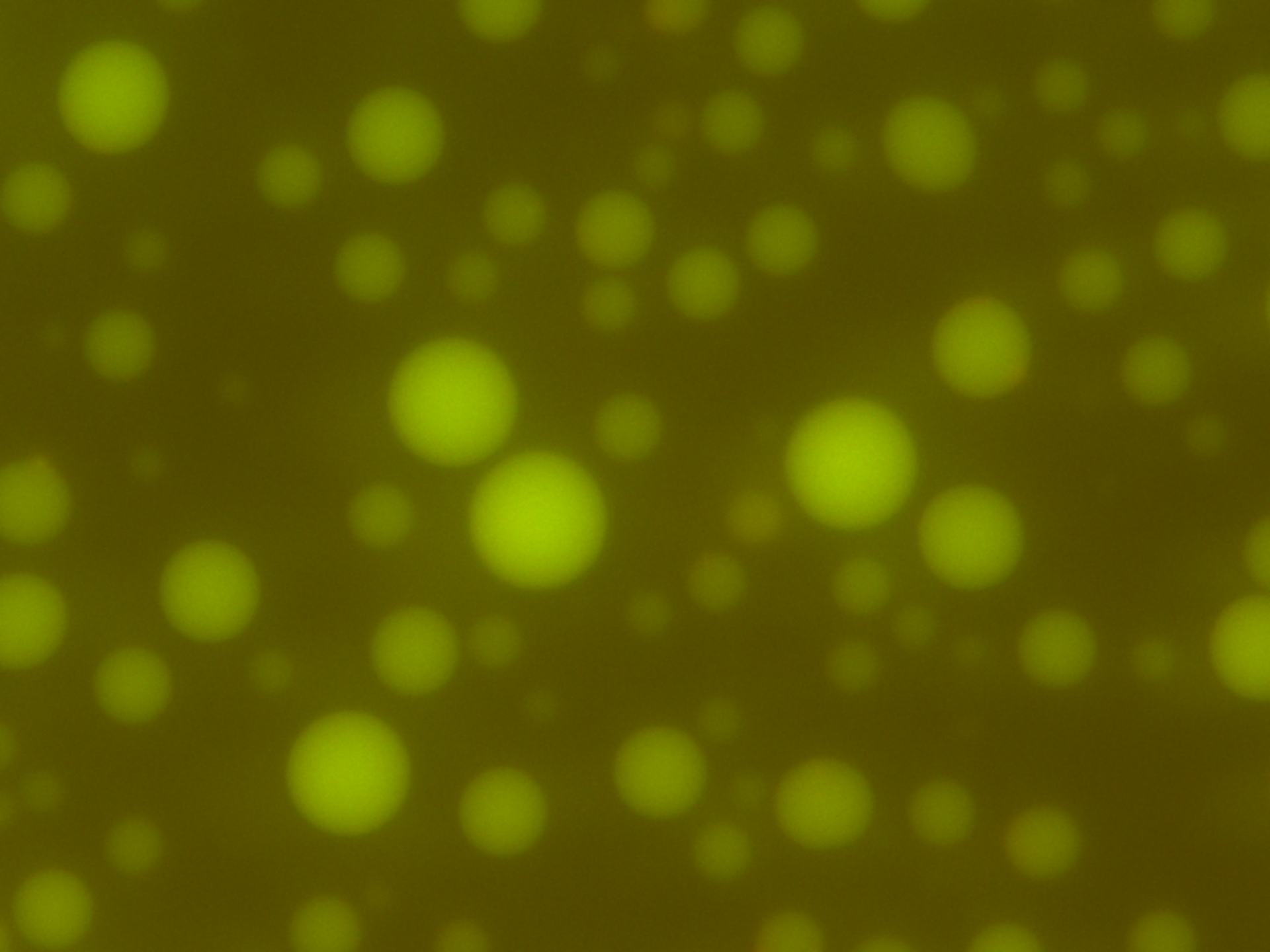

Supplement: Supplementary file 12 — Appendix Fig S5,S6 Source Data [file 44318_2025_591_MOESM12_ESM.zip › Appendix Figure S5/S5A/15_48 h_SO286(2 ╬╝M)_Merge.tif]

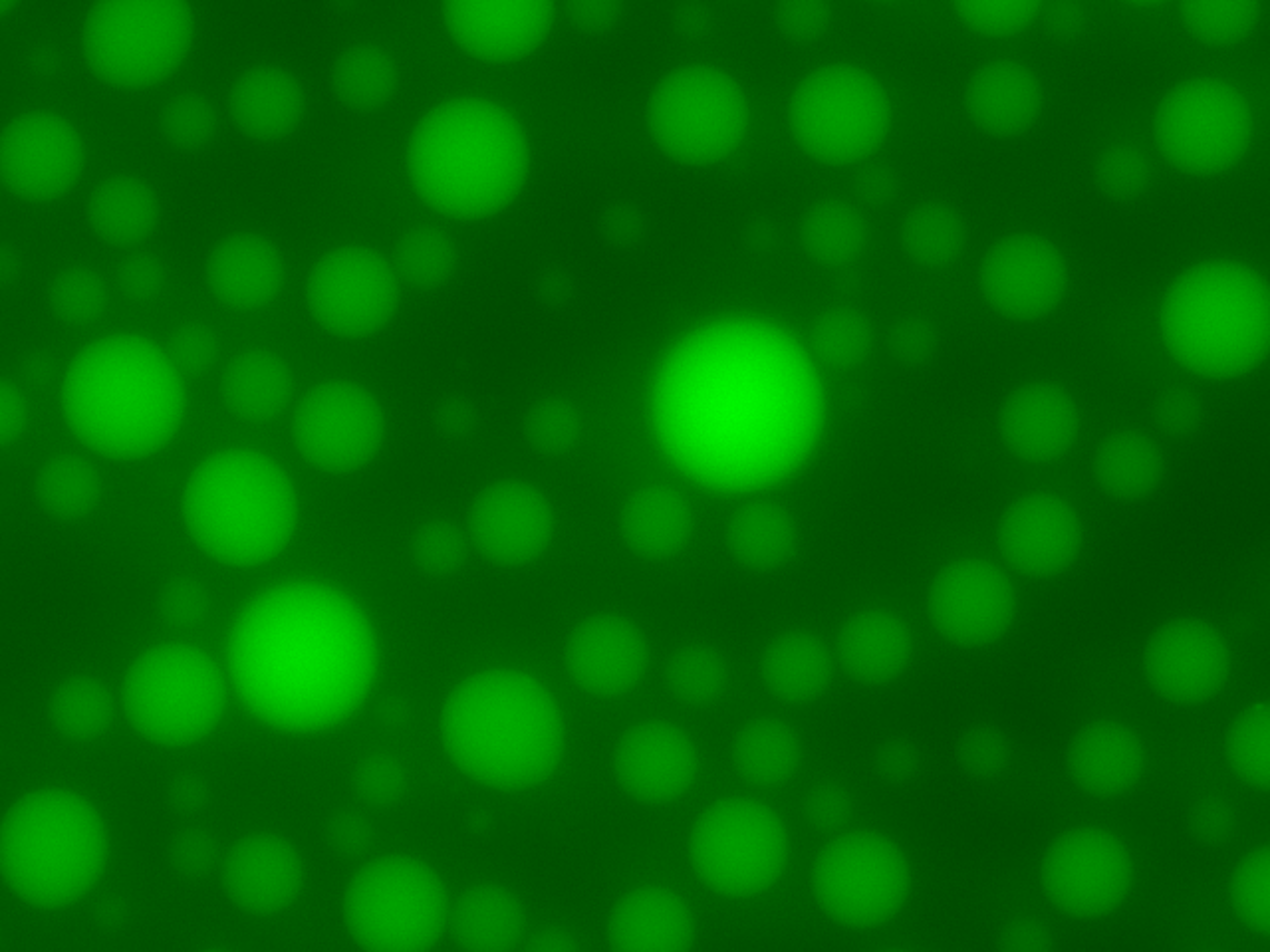

Supplement: Supplementary file 12 — Appendix Fig S5,S6 Source Data [file 44318_2025_591_MOESM12_ESM.zip › Appendix Figure S5/S5A/10_24 h_SO286(7 ╬╝M)_UBQLN2.tif]

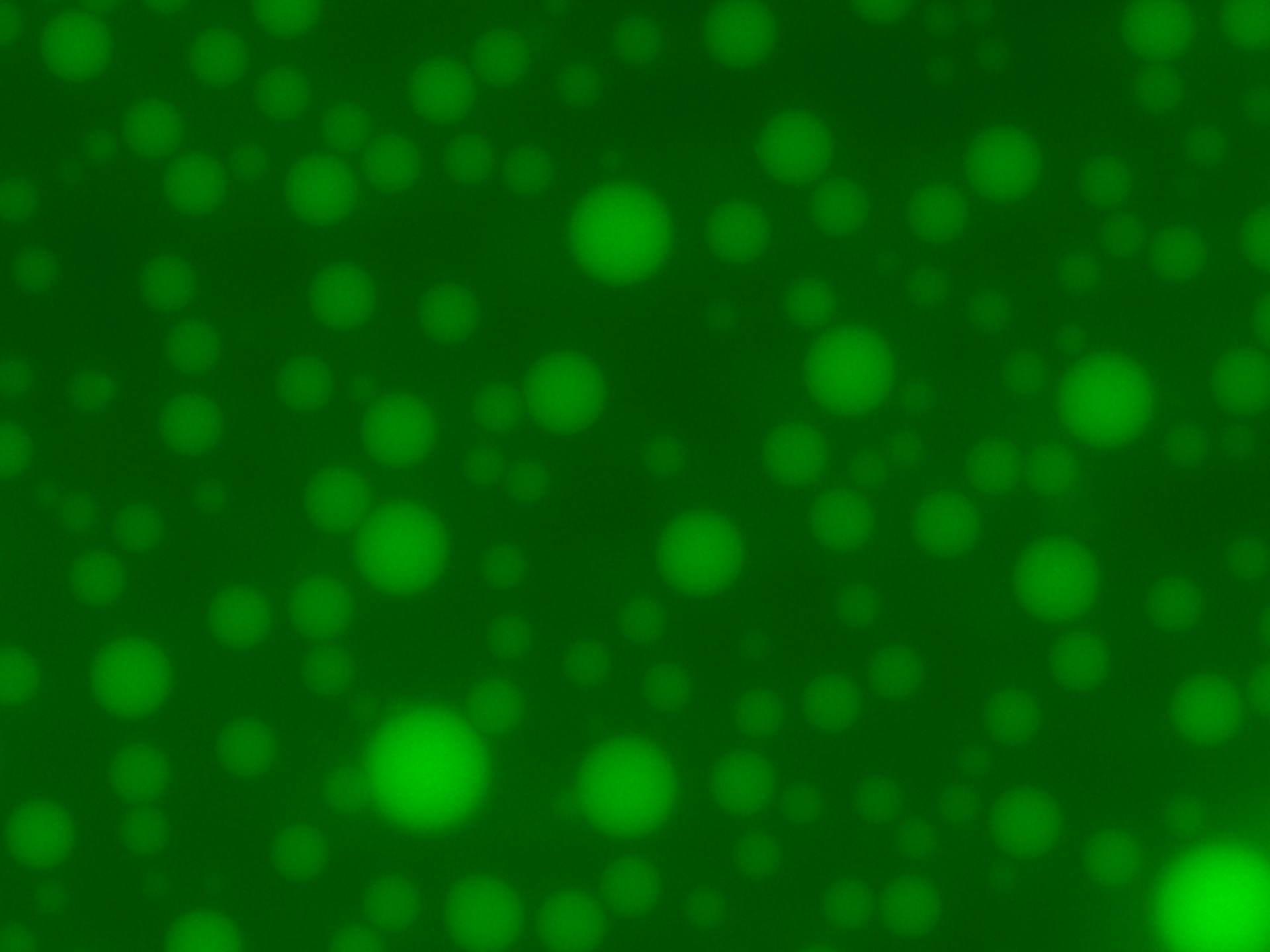

Supplement: Supplementary file 12 — Appendix Fig S5,S6 Source Data [file 44318_2025_591_MOESM12_ESM.zip › Appendix Figure S5/S5A/04_1 h_SO286(7 ╬╝M)_UBQLN2.tif]

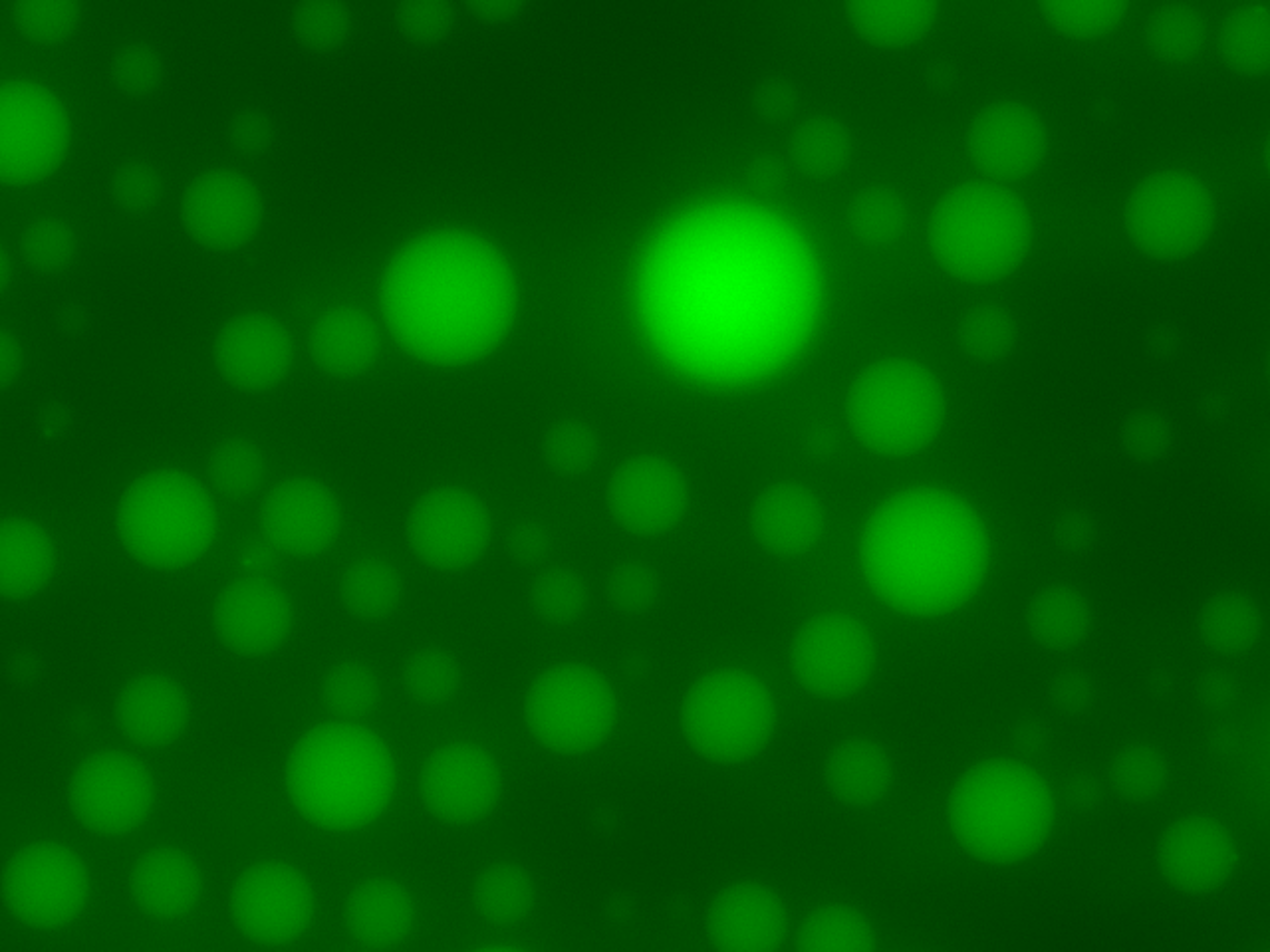

Supplement: Supplementary file 12 — Appendix Fig S5,S6 Source Data [file 44318_2025_591_MOESM12_ESM.zip › Appendix Figure S5/S5A/07_24 h_SO286(2 ╬╝M)_UBQLN2.tif]

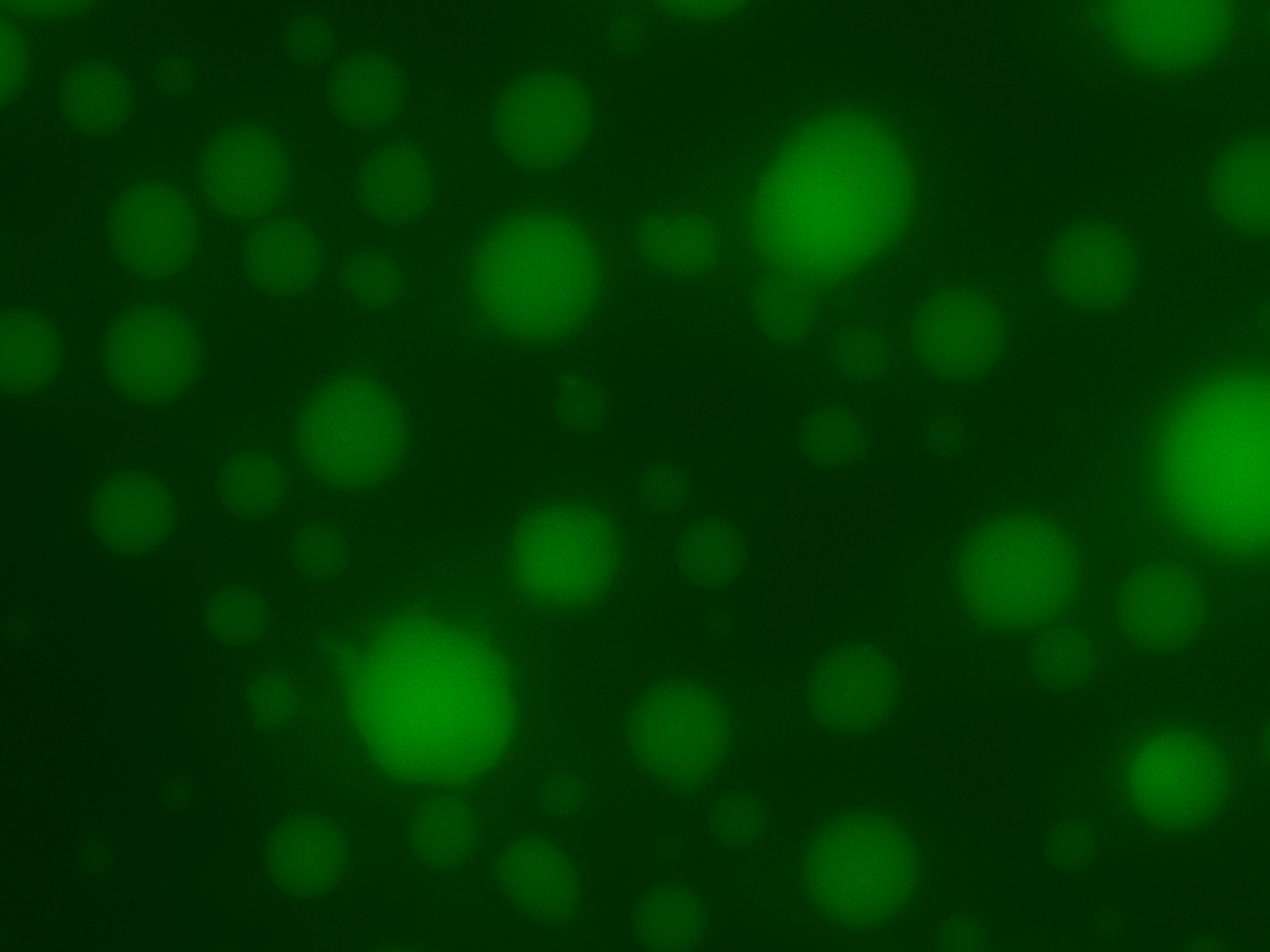

Supplement: Supplementary file 12 — Appendix Fig S5,S6 Source Data [file 44318_2025_591_MOESM12_ESM.zip › Appendix Figure S5/S5A/22_72 h_SO286(7 ╬╝M)_UBQLN2.tif]

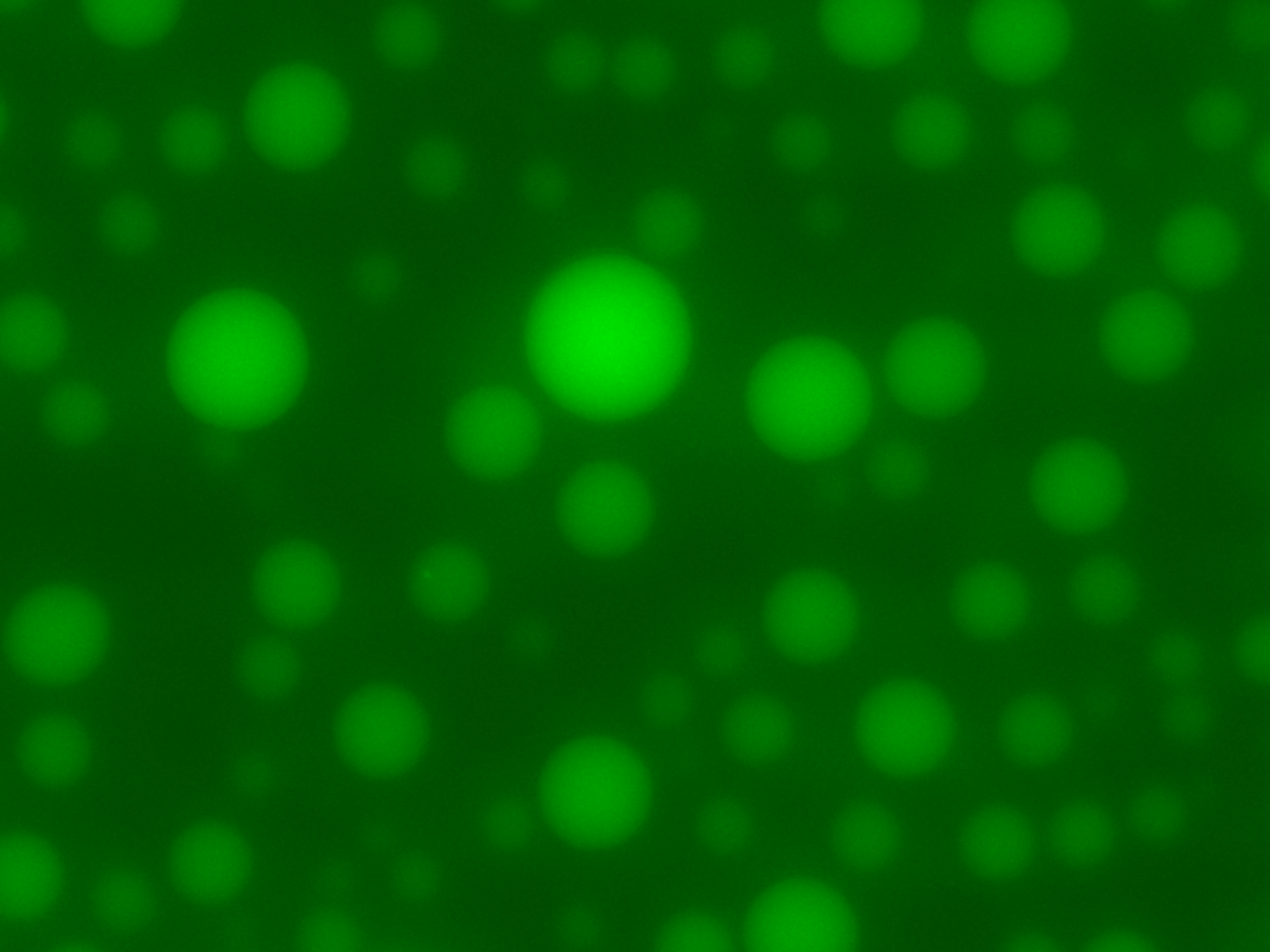

Supplement: Supplementary file 12 — Appendix Fig S5,S6 Source Data [file 44318_2025_591_MOESM12_ESM.zip › Appendix Figure S5/S5A/16_48 h_SO286(7 ╬╝M)_UBQLN2.tif]

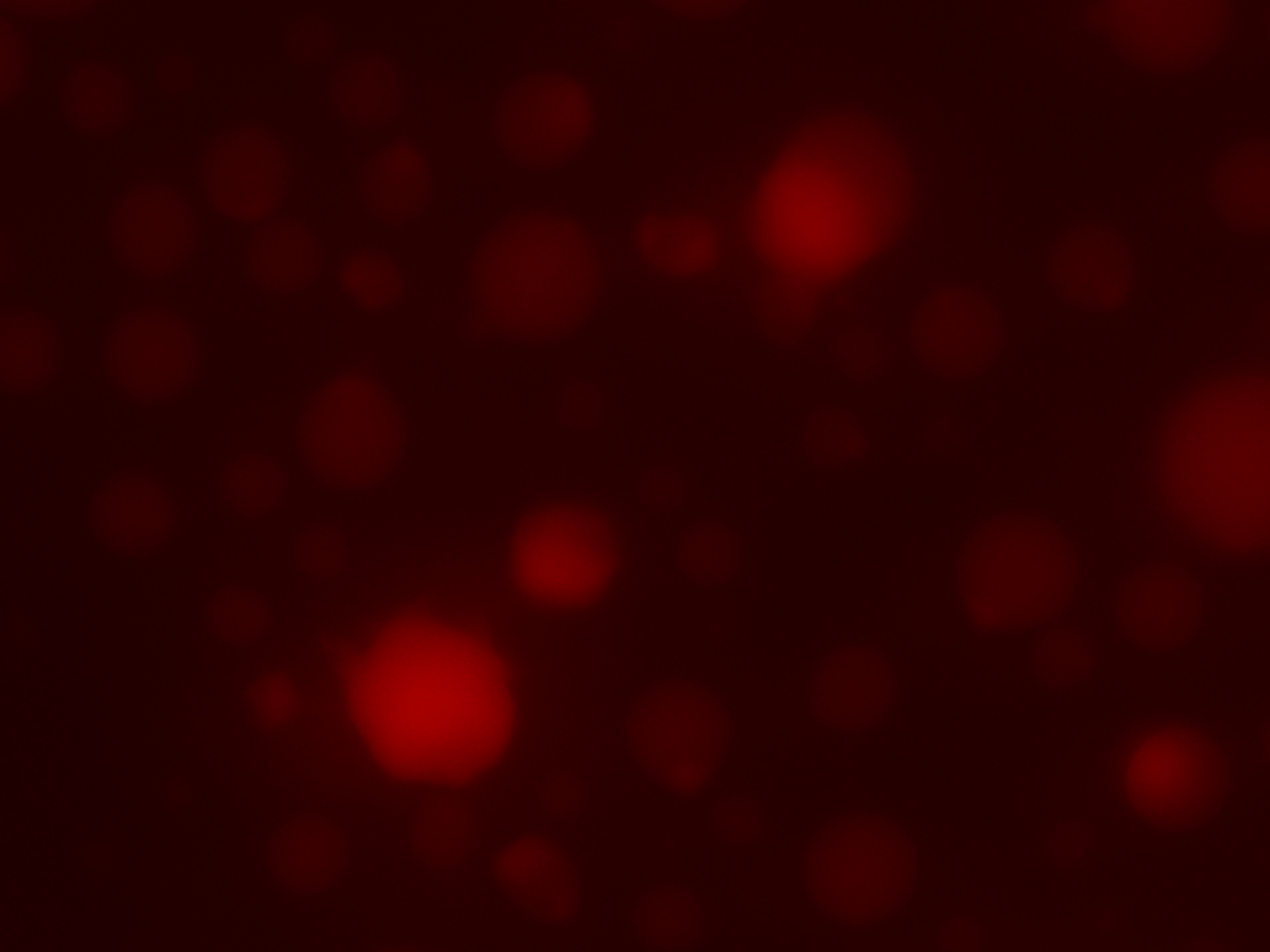

Supplement: Supplementary file 12 — Appendix Fig S5,S6 Source Data [file 44318_2025_591_MOESM12_ESM.zip › Appendix Figure S5/S5A/23_72 h_SO286(7 ╬╝M)_╬▒-Syn.tif]

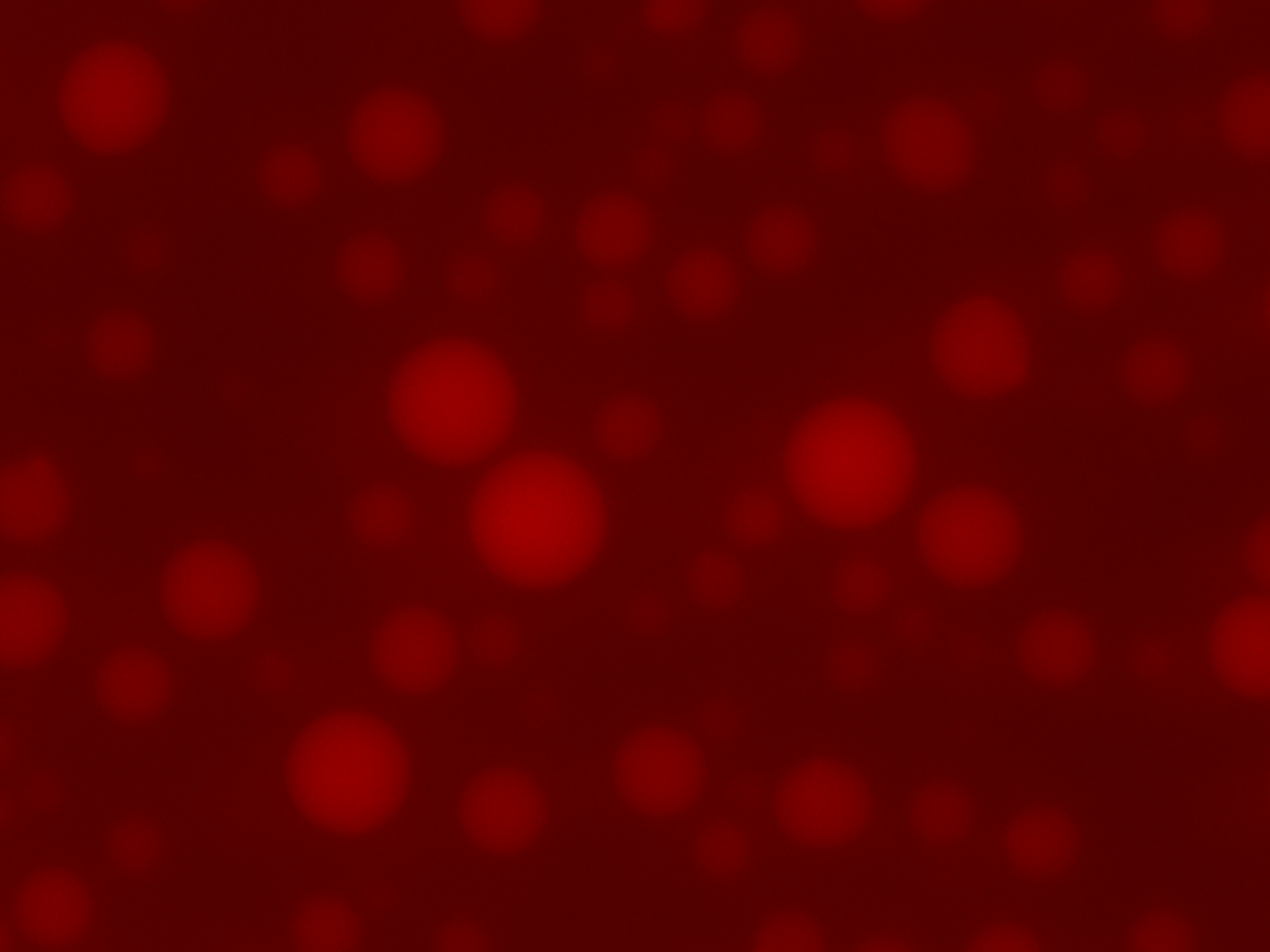

Supplement: Supplementary file 12 — Appendix Fig S5,S6 Source Data [file 44318_2025_591_MOESM12_ESM.zip › Appendix Figure S5/S5A/14_48 h_SO286(2 ╬╝M)_╬▒-Syn.tif]

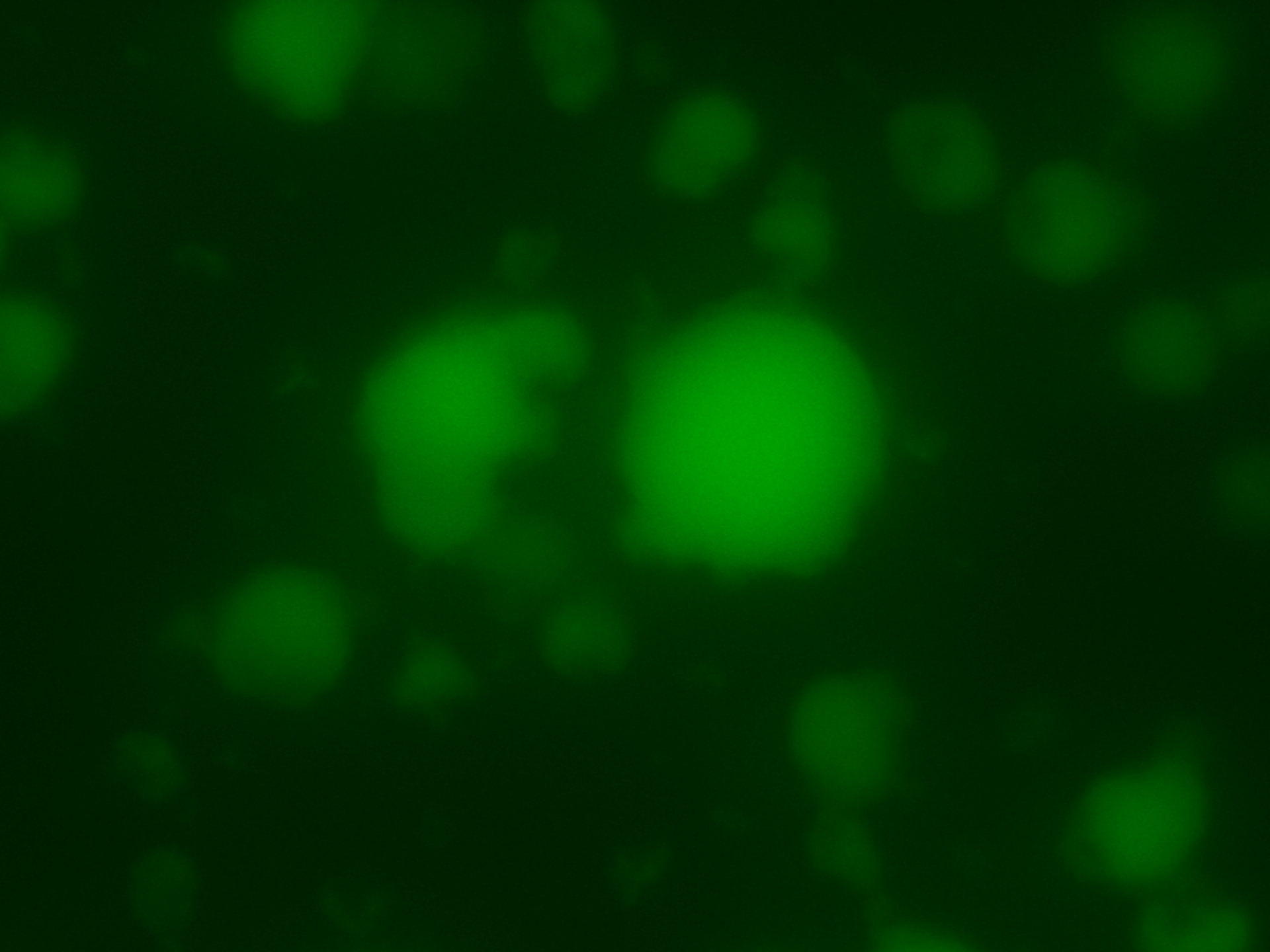

Supplement: Supplementary file 12 — Appendix Fig S5,S6 Source Data [file 44318_2025_591_MOESM12_ESM.zip › Appendix Figure S5/S5A/25_96 h_SO286(2 ╬╝M)_UBQLN2.tif]
