# Supplementary material for: Ubiquilin-2 liquid droplets catalyze α-synuclein fibril formation
Source: EMBO J. 2025 Oct 14;44(22):6527–55. doi: 10.1038/s44318-025-00591-1 (PMC12623503; doi:10.1038/s44318-025-00591-1)
Supplement: Supplementary file 13 — Expanded View Figures [file 44318_2025_591_MOESM13_ESM.pdf]

Expanded View Figures

Figure EV1. α-Syn aggregates within UBQLN2 droplets in vitro.

(**A**) Fluorescence microscopy images of mixed solutions of UBQLN2 (1% DyLight488-labeling) with α-syn (1% DyLight633-labeling) for 24 h at the indicated concentrations. (**B**) Fluorescence microscopy images of mixed solutions of 10 μM UBQLN2, ΔSTI1-2/UBQLN2 or ΔUBL/UBQLN2 (1% DyLight488-labeling) and 10 μM α-syn (1% DyLight633-labeling) after incubation at 37 °C for 24 h. (**C**) Fluorescence microscopy images showing the disassembly of UBQLN droplets incorporating α-syn following the addition of 10% 1,6-HD. (**D**) (a) Fluorescence microscopy images of each 10 μM UBQLN (1% DyLight488-labeling) in the presence or absence of 10 μM α-syn (1% DyLight633-labeling) for 24 h and 96 h. (b) FRAP analysis of droplets of each UBQLN in the presence or absence of 10 μM α-syn for 24 h and 96 h. (c) Quantification of fractional recovery at 70 s after photobleaching from 10 separate droplets. *$P < 0.05$, N.S., nonsignificant (Welch's *t* test). Values are the means ± SDs. UBQLN2 (24 h): $P = 0.0136$, UBQLN2 (96 h): $P = 0.5731$, UBQLN1 (24 h): $P = 0.3975$, UBQLN1 (96 h): $P = 0.0894$, UBQLN4 (24 h): $P = 0.3556$, UBQLN4 (96 h): $P = 0.8157$. Source data are available online for this figure.

© The Author(s)

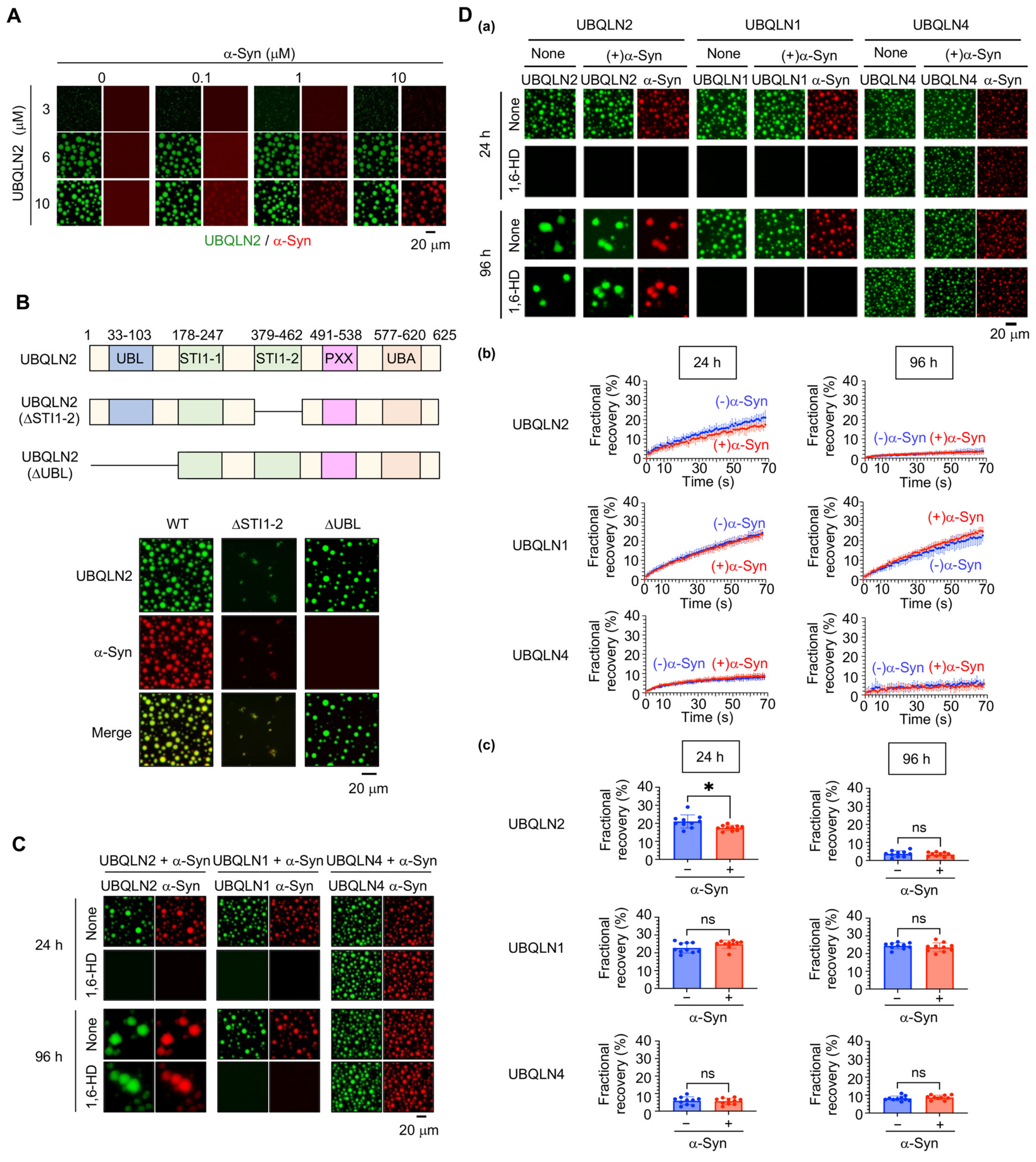
A
α-Syn (μM)
0
0.1
1
10
UBQLN2 (μM)
3
6
10
UBQLN2 / α-Syn
20 μm
B
1
33-103
178-247
379-462
491-538
577-620
625
UBQLN2
UBL
STI1-1
STI1-2
PXX
UBA
UBQLN2 (ΔSTI1-2)
UBQLN2 (ΔUBL)
WT
ΔSTI1-2
ΔUBL
UBQLN2
α-Syn
Merge
20 μm
C
UBQLN2 + α-Syn
UBQLN1 + α-Syn
UBQLN4 + α-Syn
UBQLN2 α-Syn
UBQLN1 α-Syn
UBQLN4 α-Syn
24 h
96 h
None
1,6-HD
20 μm
D
(a)
UBQLN2
UBQLN1
UBQLN4
None
(+)α-Syn
UBQLN2 UBQLN2 α-Syn
UBQLN1 UBQLN1 α-Syn
UBQLN4 UBQLN4 α-Syn
24 h
96 h
None
1,6-HD
20 μm
(b)
24 h
96 h
UBQLN2
UBQLN1
UBQLN4
Fractional recovery (%)
Time (s)
(-)α-Syn
(+)α-Syn
(c)
24 h
96 h
Fractional recovery (%)
*
ns
α-Syn

 © The Author(s)

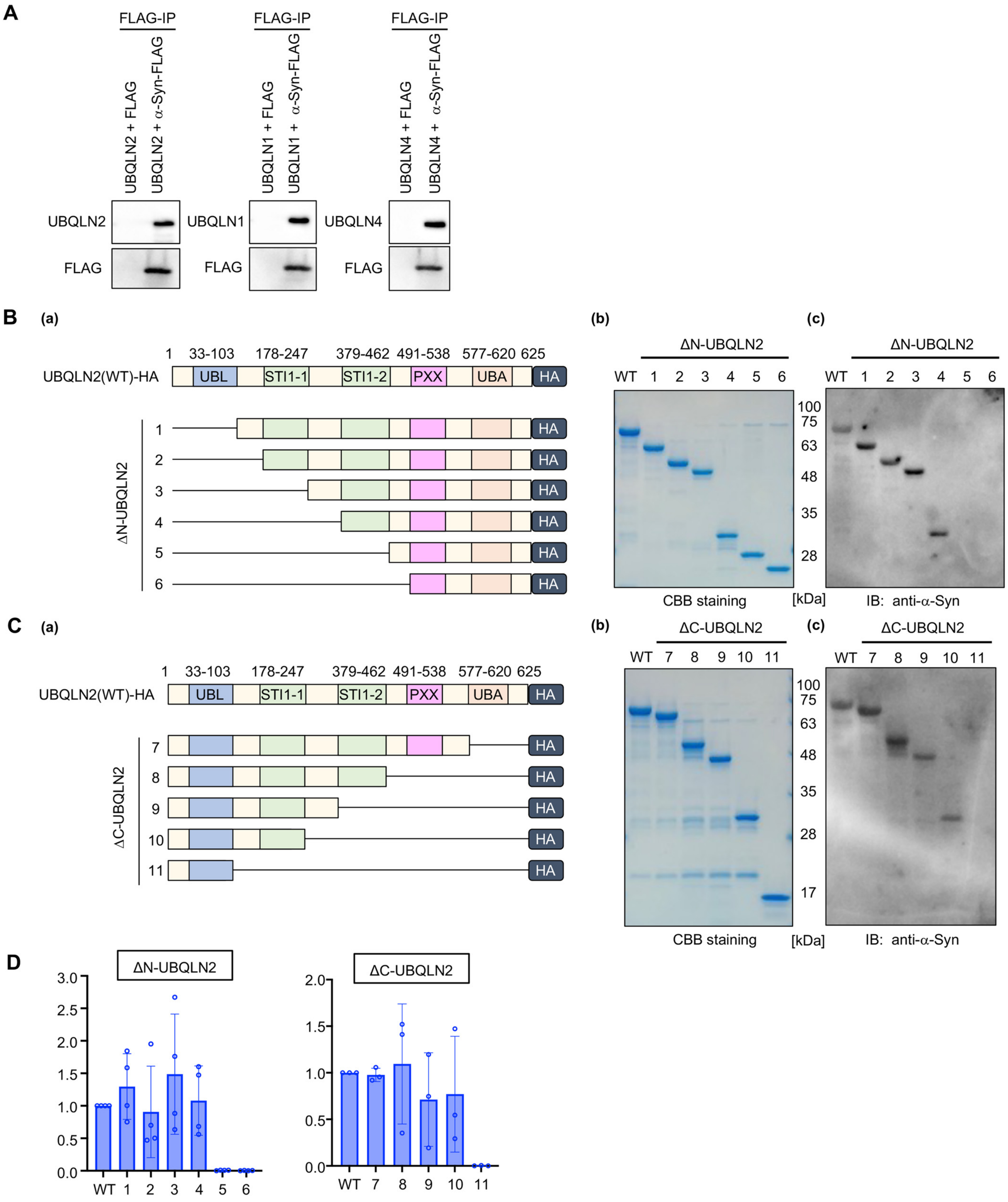
A
FLAG-IP
UBQLN2 + FLAG
UBQLN2 + α-Syn-FLAG
UBQLN2
FLAG
FLAG-IP
UBQLN1 + FLAG
UBQLN1 + α-Syn-FLAG
UBQLN1
FLAG
FLAG-IP
UBQLN4 + FLAG
UBQLN4 + α-Syn-FLAG
UBQLN4
FLAG
B (a)
1 33-103 178-247 379-462 491-538 577-620 625
UBQLN2(WT)-HA
UBL STI1-1 STI1-2 PXX UBA HA
ΔN-UBQLN2
1 2 3 4 5 6
(b)
ΔN-UBQLN2
WT 1 2 3 4 5 6
100 75 63 48 35 28
CBB staining [kDa]
(c)
ΔN-UBQLN2
WT 1 2 3 4 5 6
IB: anti-α-Syn
C (a)
1 33-103 178-247 379-462 491-538 577-620 625
UBQLN2(WT)-HA
UBL STI1-1 STI1-2 PXX UBA HA
ΔC-UBQLN2
7 8 9 10 11
(b)
ΔC-UBQLN2
WT 7 8 9 10 11
100 75 63 48 35 28 17
CBB staining [kDa]
(c)
ΔC-UBQLN2
WT 7 8 9 10 11
IB: anti-α-Syn
D
ΔN-UBQLN2
3.0 2.5 2.0 1.5 1.0 0.5 0.0
WT 1 2 3 4 5 6
ΔC-UBQLN2
2.0 1.5 1.0 0.5 0.0
WT 7 8 9 10 11

© The Author(s)

Figure EV2. α-Syn directly interacts with UBQLN2.

(**A**) Recombinant α-syn-FLAG and each hemagglutinin (HA)-UBQLN were incubated before α-syn-FLAG was pulled down with anti-FLAG beads and α-syn-FLAG was eluted with the FLAG peptide. The eluates were then immunoblotted with the indicated antibodies. Representative blot shown, $n = 2$. (**B**, **C**) (a) Illustrations of the UBQLN2 mutant with successive N-terminal deletions (**B**) or successive C-terminal deletions (**A**). (b) Representative SDS-PAGE images of each HA-tagged UBQLN2 mutant stained with Coomassie Brilliant Blue (CBB). (c) Representative far-western blotting results showing α-syn binding to each UBQLN2 mutant. After SDS-PAGE and membrane transfer, the membranes were incubated with recombinant His-tagged α-syn, followed by detection with an anti-α-syn antibody. (**D**) Quantification of α-syn binding to each UBQLN2 mutant. The binding signal (western blot) was normalized to the amount of HA-UBQLN2 protein (CBB staining). Graphs show the mean ± SD from four independent experiments for ΔN-UBQLN2 mutants and three independent experiments for ΔC-UBQLN2 mutants, corresponding to the representative results shown in (**B**, **C**). Source data are available online for this figure.

© The Author(s)

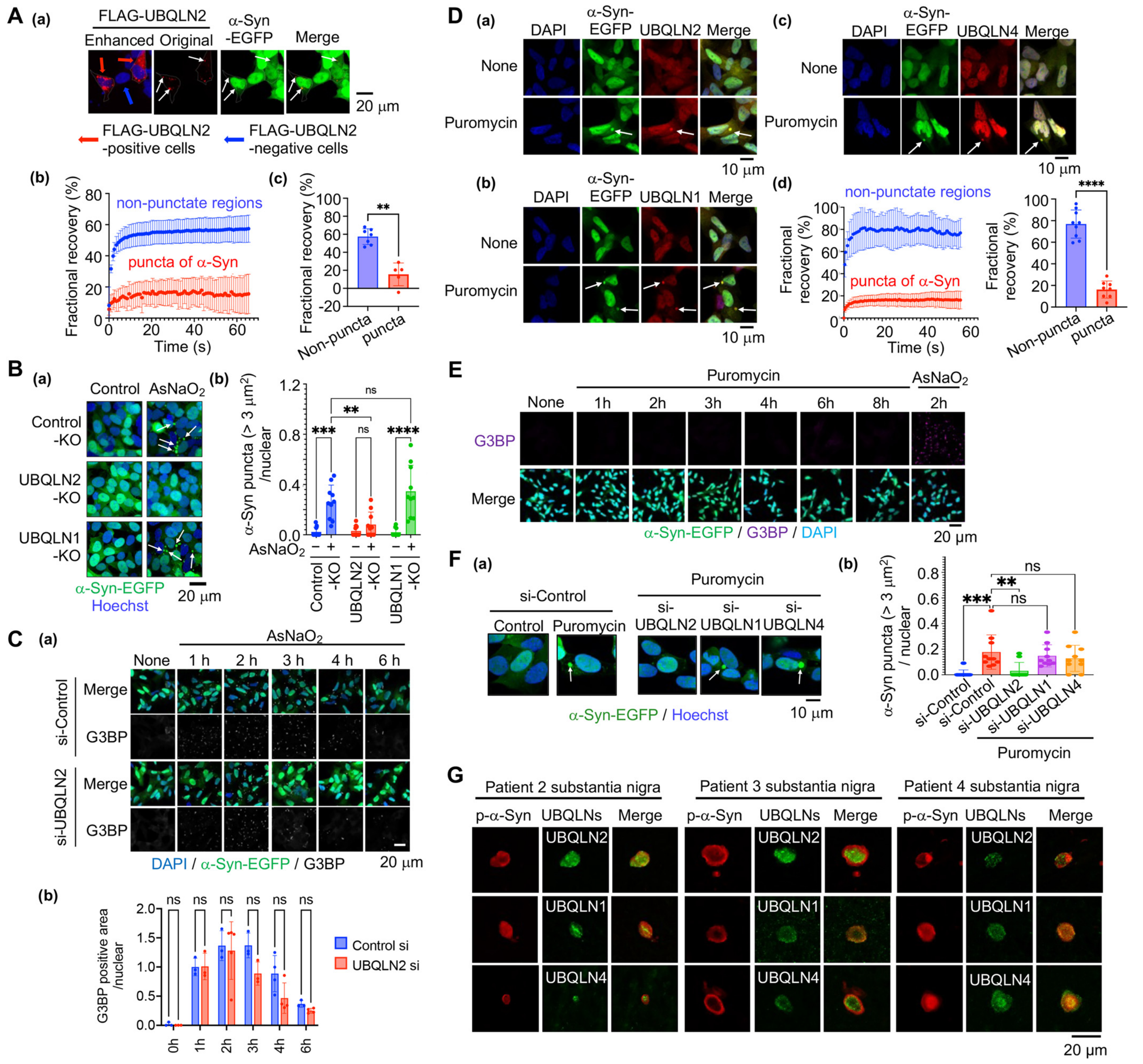

A (a) FLAG-UBQLN2 Enhanced Original α-Syn-EGFP Merge 20 μm
FLAG-UBQLN2-positive cells
FLAG-UBQLN2-negative cells
(b) Fractional recovery (%) non-punctate regions puncta of α-Syn Time (s)
(c) Fractional recovery (%) Non-puncta puncta
B (a) Control AsNaO2 Control-KO UBQLN2-KO UBQLN1-KO α-Syn-EGFP Hoechst 20 μm
(b) α-Syn puncta (> 3 μm²)/nuclear AsNaO2 Control-KO UBQLN2-KO UBQLN1-KO
C (a) None AsNaO2 1 h 2 h 3 h 4 h 6 h si-Control si-UBQLN2 Merge G3BP DAPI / α-Syn-EGFP / G3BP 20 μm
(b) G3BP positive area /nuclear Control si UBQLN2 si 0h 1h 2h 3h 4h 6h
D (a) DAPI α-Syn-EGFP UBQLN2 Merge None Puromycin 10 μm
(b) DAPI α-Syn-EGFP UBQLN1 Merge None Puromycin 10 μm
(c) DAPI α-Syn-EGFP UBQLN4 Merge None Puromycin 10 μm
(d) non-punctate regions puncta of α-Syn Fractional recovery (%) Time (s) Non-puncta puncta
E Puromycin AsNaO2 None 1h 2h 3h 4h 6h 8h 2h G3BP Merge α-Syn-EGFP / G3BP / DAPI 20 μm
F (a) si-Control Control Puromycin si-UBQLN2 si-UBQLN1 si-UBQLN4 α-Syn-EGFP / Hoechst 10 μm
(b) α-Syn puncta (> 3 μm²) / nuclear si-Control si-Control si-UBQLN2 si-UBQLN1 si-UBQLN4 Puromycin
G Patient 2 substantia nigra Patient 3 substantia nigra Patient 4 substantia nigra p-α-Syn UBQLNs Merge UBQLN2 UBQLN1 UBQLN4 20 μm

Figure EV3. α-Syn aggregates within UBQLN2 droplets in cultured cells.

(**A**) (a) α-Syn(WT)-EGFP/SH-SY5Y cells were transiently transfected with FLAG-UBQLN2 for 24 h and stained with anti-FLAG antibody and DAPI both FLAG-UBQLN2-expressing and non-expressing cells were observed under a microscope. Images in the left column were linearly contrast-enhanced using ImageJ for visualization purposes. At least three experiments were replicated. (b) FRAP analysis of droplets of α-syn puncta at 24 h following transfection. Plots show the average FRAP recovery curves from at least 5 separate droplets. Non-puncta regions were photobleached as a control in the FRAP analysis. $^{**}P < 0.01$ (Mann–Whitney test). Values are the means ± SDs. Non-puncta vs puncta: $P = 0.0025$. (**B**) (a) Fluorescence microscopy of these cells after 12 h $AsNaO_2$ (50 μM) treatment, stained with Hoechst. White arrows indicate large α-syn-EGFP condensates. At least three experiments were replicated. (b) Quantification of condensates (> 3 μm²) per cell. $^{**}P < 0.01$, $^{***}P < 0.001$, $^{****}P < 0.0001$, N.S. (two-way ANOVA, Tukey's test). Ten images per condition. Values of mean ± SD. Control-KO/Control vs Control-KO/$AsNaO_2$: $P = 0.0002$, UBQLN2-KO/Control vs UBQLN2-KO/$AsNaO_2$: $P = 0.9047$, UBQLN1-KO/Control vs UBQLN1-KO/$AsNaO_2$: $P < 0.0001$, Control-KO/$AsNaO_2$ vs UBQLN2-KO/$AsNaO_2$: $P = 0.0092$, Control-KO/$AsNaO_2$ vs UBQLN1-KO/$AsNaO_2$: $P = 0.5394$. (**C**) Fluorescence microscopy of α-syn(WT)-EGFP/SH-SY5Y cells with UBQLN2 knockdown. (a) Cells were transfected with si-Control or si-UBQLN2, treated with 50 μM $AsNaO_2$ for the indicated time points post 48 h transfection, and immunostained with anti-G3BP and DAPI. (b) Quantification of the number of G3BP-positive condensates per cell. The values are normalized to the mean of the Ctrl si group at 1 h. N.S., nonsignificant. Statistical comparisons between control si and UBQLN2 si were performed at each time point using the Mann–Whitney test ($n = 3$–5, technical replicates). 0 h: $P > 0.9999$, 1 h: $P > 0.9999$, 2 h: $P = 0.9881$, 3 h: $P = 0.2596$, 4 h: $P = 0.2488$, 6 h: $P = 0.9704$. (**D**) α-Syn (WT)-EGFP/SH-SY5Y cells were treated with 3 μg/ml puromycin for 8 h and subsequently immunostained with anti-UBQLN2 (a), anti-UBQLN1 (b), or anti-UBQLN4 and DAPI. (c) UBQLN2 droplets and large α-syn-EGFP condensates are indicated by white arrows. At least three experiments were replicated. (d) FRAP analysis was performed on α-syn droplets following treatment with 3 μg/ml puromycin for 8 h. Plots show the average FRAP recovery curves from at least 8 separate droplets. Values are the mean ± SD. Quantification of fractional recovery at 70 s after photobleaching from at least 8 separate droplets. Values are the means ± SDs. Non-puncta regions were photobleached as a control in the FRAP analysis. $^{****}P < 0.001$ (Mann–Whitney test). Non-puncta vs puncta: $P < 0.0001$. (**E**) α-Syn(WT)-EGFP/SH-SY5Y cells were treated with 3 μg/ml puromycin for the indicated time points or 50 μM $AsNaO_2$ for 2 h, and subsequently immunostained with anti-G3BP and DAPI. (**F**) (a) Fluorescence microscopy of α-syn(WT)-EGFP/SH-SY5Y cells with UBQLN knockdown. Cells transfected with si-Control, si-UBQLN2, si-UBQLN1, or si-UBQLN4 were treated with puromycin (10 μg/mL, 6 h) and stained with Hoechst. (b) Quantification of condensates (> 3 μm²) per cell. $^{**}P < 0.01$, $^{***}P < 0.001$, N.S. (Dunnett's test, versus si-Control/puromycin). Ten images per condition; mean ± SD. si-Control (Puromycin) vs si-Control: $P = 0.0006$, si-Control(Puromycin) vs si-UBQLN2(Puromycin): $P = 0.0028$, si-Control(Puromycin) vs si-UBQLN1(Puromycin): $P = 0.8805$, si-Control(Puromycin) vs si-UBQLN4(Puromycin): $P = 0.5718$. (**G**) Accumulation of UBQLN2 in LBs of sporadic PD patients (patient 2 ~ 4). Sections from the midbrain of pathologically diagnosed PD cases were stained with each anti-UBQLN antibodies or anti-p-α-Syn antibody.

 © The Author(s)

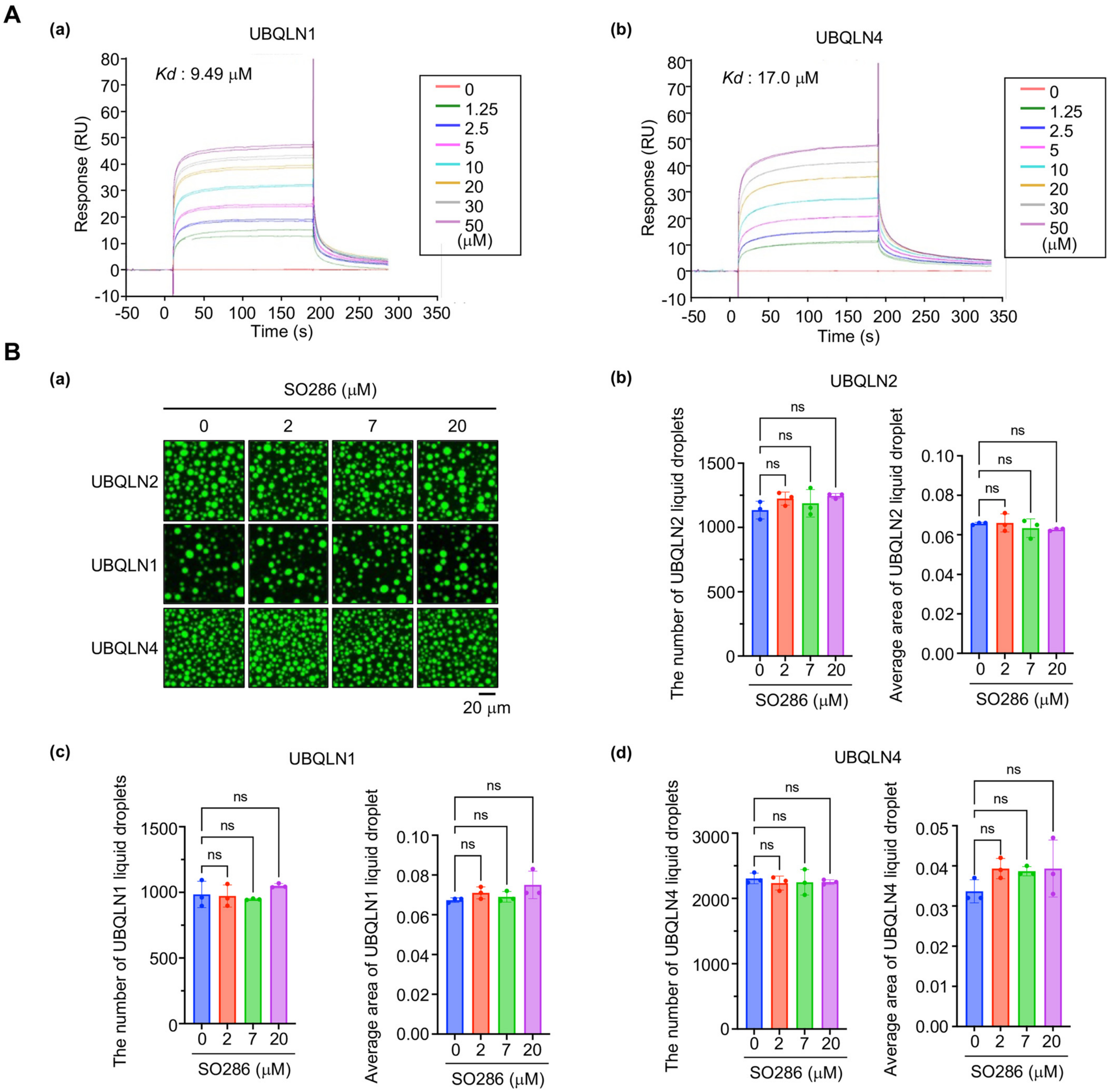

Figure EV4. SO286 binds to the STI1 regions of UBQLN2.

(**A**) Surface plasmon resonance analysis of biotin–SO286 reactivity with UBQLN1 (a) and UBQLN4 (b). The vertical axis represents the amount of binding in the resonance unit (RU). (**B**) (a) Fluorescence microscopy images showing 10 μM solutions of each UBQLN construct (1% DyLight488-labeling) after incubation at 37 °C for 24 h in the presence of SO286. The number and size of the droplets of UBQLN2 (b), UBQLN1 (c), and UBQLN4 (d) were quantified. N.S., nonsignificant (Dunnett's test, versus SO286 [0 μM]). Three images were quantified per condition. Values are the means ± SDs. (b) The number of UBQLN2 liquid droplets; 0 μM vs 2 μM: $P = 0.3283$, 0 μM vs 7 μM: $P = 0.6847$, 0 μM vs 20 μM: $P = 0.1971$. Average area of UBQLN2 liquid droplet; 0 μM vs 2 μM: $P = 0.9986$, 0 μM vs 7 μM: $P = 0.7304$, 0 μM vs 20 μM: $P = 0.5769$. (c) The number of UBQLN1 liquid droplets; 0 μM vs 2 μM: $P = 0.9918$, 0 μM vs 7 μM: $P = 0.8381$, 0 μM vs 20 μM: $P = 0.5056$. Average area of UBQLN1 liquid droplet; 0 μM vs 2 μM: $P = 0.5748$, 0 μM vs 7 μM: $P = 0.9208$, 0 μM vs 20 μM: $P = 0.1141$. (d) The number of UBQLN4 liquid droplets; 0 μM vs 2 μM: $P = 0.8147$, 0 μM vs 7 μM: $P = 0.8762$, 0 μM vs 20 μM: $P = 0.8955$. Average area of UBQLN4 liquid droplet; 0 μM vs 2 μM: $P = 0.2767$, 0 μM vs 7 μM: $P = 0.3611$, 0 μM vs 20 μM: $P = 0.2767$. Source data are available online for this figure.

© The Author(s)

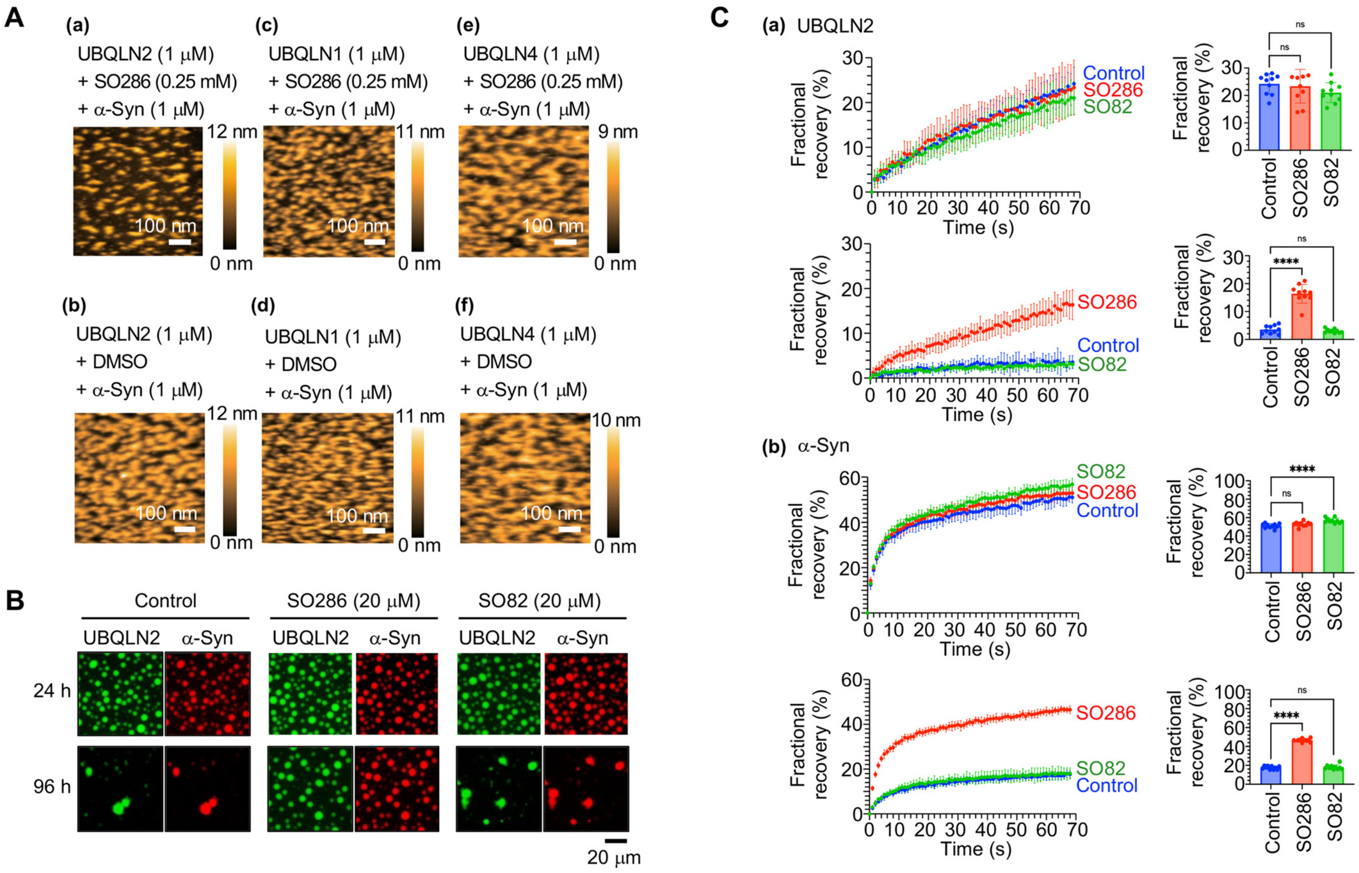

Figure EV5. SO286 inhibits α-syn aggregation within UBQLN2 droplets in vitro.

(**A**) Effects of SO286 on the spinodal decomposition-like patterns of α-syn and UBQLN2 (a, b), UBQLN1 (c, d), or UBQLN4 (e, f). Each UBQLN (1 μM) was premixed with (a, c, e) or without (b, d, f) SO286 (0.25 mM) before being mixed with α-syn (1 μM). (**B**) Fluorescence microscopy images of mixed solutions of 10 μM UBQLN2 construct (1% DyLight488-labeling) and 10 μM α-syn construct (1% DyLight633-labeling) after incubation at 37 °C for 24 and 96 h in the presence or absence of 20 μM SO286 or SO82. (**C**) FRAP analysis of droplets of UBQLN2 incorporating α-syn in the presence or absence of 20 μM SO286 or SO82 and quantification of fractional recovery at 70 s after photobleaching from 10 separate droplets. ****$P < 0.0001$, N.S., nonsignificant (Dunnett's test, versus Control). Values are the means ± SDs. (a) 24 h/Control vs 24 h/SO286: $P = 0.8863$, 24 h/Control vs 24 h/SO82: $P = 0.2308$, 96 h/Control vs 96 h/SO286: $P < 0.0001$, 96 h/Control vs 96 h/SO82: $P = 0.8253$. (b) 24 h/Control vs 24 h/SO286: $P = 0.1874$, 24 h/Control vs 24 h/SO82: $P < 0.0001$, 96 h/Control vs 96 h/SO286: $P < 0.0001$, 96 h/Control vs 96 h/SO82: $P = 0.8330$. Source data are available online for this figure.

 © The Author(s)

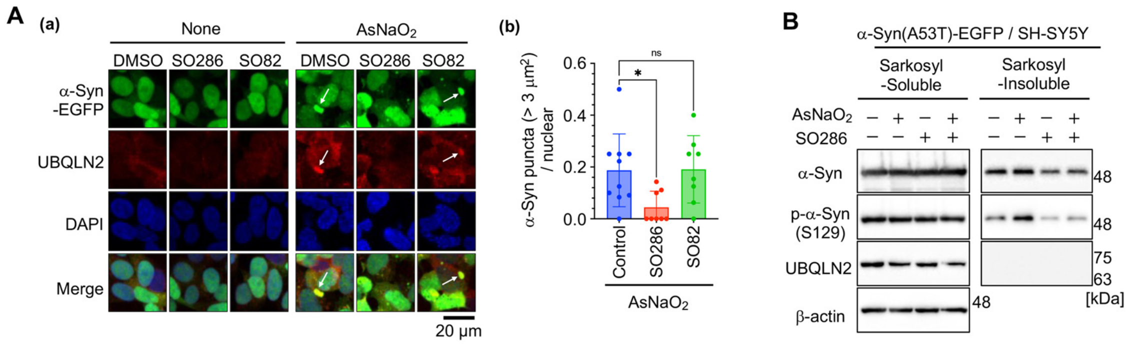

Figure EV6. SO286 inhibits α-syn aggregation within UBQLN2 droplets in cultured cells.

(**A**) (a) a-Syn(WT)-EGFP/SH-SY5Y cells were treated with 50 µM $AsNaO_2$ for the indicated time points in the presence of 20 µM SO286 or SO82 and immunostained with anti-UBQLN2 and DAPI. UBQLN2 droplets and large α-syn-EGFP condensates are indicated by white arrows. Control vs SO286: $P = 0.0341$, Control vs SO82: $P = 0.9969$. (b) Quantification of the number of α-syn-EGFP puncta (> 3 µm^2) per cell. *$P < 0.05$, N.S., nonsignificant (Dunnett's test, versus $AsNaO_2$/DMSO). Nine images were quantified per condition. Values are the means ± SDs. (**B**) α-Syn(A53T)-EGFP/SH-SY5Y cells were treated with 50 µM $AsNaO_2$ for 12 h in the presence of 20 µM SO286. Sarkosyl-soluble and -insoluble fractions were subjected to SDS-PAGE and immunoblotted with the indicated antibodies. Representative blot shown, $n = 2$. Source data are available online for this figure.

© The Author(s)
